# Supplementary material for: A lipid compendium of a metabolically compromised bacterium provides insights into lipid acquisition, biosynthesis, and metabolism
Source: bioRxiv. 2026 May 23:2026.05.22.727245. Preprint. [Version 1] doi: 10.64898/2026.05.22.727245 (PMC13228262; doi:10.64898/2026.05.22.727245)
Supplement: Supplement 1 [file media-1.pdf]

Supplementary Spreadsheet 1: Comparative analysis of lipid species significantly re

Name	lipid_class	Group Area: CM_pos_12022024_Newanalysis
AcHexChE(15:0)	AcHexChE	4754849.891
BisMeLPA(12:0e)	BisMeLPA	16693909.26
BisMeLPA(10:0e)	BisMeLPA	2145335.997
BMP(18:0_18:1)	BMP	10292743.78
CarE(16:1)	CarE	23250441.29
Cer(d18:1_23:0)	Cer	66567694.82
Cer(t17:0_26:0+O)	Cer	28393969.27
Cer(d18:1_24:1)	Cer	45209902.01
Cer(d18:1_22:0)	Cer	43985993.19
Cer(t20:0_25:0+O)	Cer	6251081.394
Cer(t42:0)	Cer	4456662.961
Cer(d18:1_24:2)	Cer	8002096.473
Cer(d18:1_20:0)	Cer	10760299.53
Cer(d18:1_16:0)	Cer	8903255.662
Cer(m36:0)	Cer	4235536.527
Cer(d18:1_24:0)	Cer	2313140.643
Cer(d19:2_31:0+O)	Cer	1670027.682
Cer(m18:1_22:0)	Cer	1711355.254
ChE(18:3)	ChE	592096919.7
ChE(20:5)	ChE	88554223.05
ChE(18:2)	ChE	66016579.57
ChE(20:4)	ChE	15822942.41
CmE(18:3)	CmE	16788578.1
CmE(18:2)	CmE	10284411.74
Co(Q10)	Co	8799182.022
DG(18:1_16:0)	DG	144194289.9
DG(32:2e)	DG	94099416.24
DG(34:3e)	DG	72666614.08
DG(34:4e)	DG	68938875.66

DG(18:2_16:0)	DG	83035866.2
DG(36:2e)	DG	923308186
DG(18:0_16:0)	DG	24530659.76
DG(34:2e)	DG	484143510.9
DG(36:4e)	DG	35940976.92
DG(36:5)	DG	83035866.2
DG(36:5e)	DG	33078450.18
DG(38:2e)	DG	18342256.25
DG(16:0_18:3)	DG	12357206.54
DG(35:2e)	DG	9081236.961
DG(36:6)	DG	2395343.124
DG(18:1_18:1)	DG	10779000.61
DG(36:3e)	DG	10928828.82
DG(36:6e)	DG	9435531.407
DG(18:4_18:1)	DG	5990051.713
DG(18:1_18:2)	DG	154204355.3
DG(18:1_18:3)	DG	8323241.929
DG(16:0_16:1)	DG	4775491.649
DG(18:0_18:1)	DG	38454971.96
DG(28:5)	DG	3792649.136
Hex1Cer(d18:1_16:0)	Hex1Cer	4709144.279
LPC(18:0)	LPC	179369466.1
LPC(16:0)	LPC	205449825.3
LPC(18:2)	LPC	72490370.65
LPC(18:1)	LPC	53022168.68
LPC(20:3)	LPC	20114721.3
LPC(20:4)	LPC	50971693.25
LPC(18:1e)	LPC	20498145.2
LPC(18:0e)	LPC	9324409.353
LPC(18:3)	LPC	8065158.096

LPC(16:0e)	LPC	25751206.26
LPC(16:1)	LPC	19733330.37
LPC(15:0)	LPC	15969773.3
LPC(17:1)	LPC	6550003.59
LPC(19:0)	LPC	30343263.09
LPC(17:0)	LPC	18094312.51
LPC(18:2e)	LPC	1955041.82
LPC(14:0)	LPC	3118045.517
LPC(19:1)	LPC	3899804.56
LPC(22:5)	LPC	4494355.48
LPC(20:2)	LPC	12715397.37
LPC(20:0)	LPC	3202887.295
LPC(20:1)	LPC	1186185.708
LPC(22:4)	LPC	7657502.836
LPC(16:1e)	LPC	15891664.54
LPC(18:4)	LPC	1827795.88
LPC(18:1D7)	LPC	1037178.289
LPC(20:5)	LPC	1656916.303
LPC(31:0)	LPC	2568692.659
LPC(16:2e)	LPC	672779.2774
LPC(20:1e)	LPC	1869035.57
LPC(20:2e)	LPC	348352.4492
LPE(18:0)	LPE	10736796.6
MePC(35:0)	MePC	154673422.3
MePC(14:1e)	MePC	1474300.644
MGDG(19:1_19:1)	MGDG	1903868.151
PC(18:0_18:1)	PC	1337465516
PC(18:1_18:2)	PC	395367558.9
PC(19:1_19:1)	PC	154021498.4
PC(18:1e_20:4)	PC	31778660.35

PC(18:0_16:0)	PC	98592128.24
PC(16:0_18:3)	PC	7444233.464
PC(18:0_22:4)	PC	195387267.2
PC(20:2_18:2)	PC	60043432.42
PC(18:1_18:1)	PC	11741736033
PC(35:3e)	PC	7571577.017
PC(16:0e_18:1)	PC	130349353.3
PC(38:7D5)	PC	182239479.2
PC(21:4)	PC	2570705.572
PC(40:9)	PC	2647353.422
PC(16:0_17:0)	PC	4662525.431
PC(17:0_18:2)	PC	23404118.53
PC(18:0e_18:2)	PC	60992470.75
PC(18:0_18:2)	PC	2468337.394
PC(40:7)	PC	67187826.31
PC(32:3e)	PC	2193902.088
PC(18:3_18:2)	PC	19733188.68
PC(36:2)	PC	11484193.38
PC(16:1e_16:0)	PC	18423515.03
PC(20:0_18:2)	PC	11626906.23
PC(22:5_18:2)	PC	1469881.203
PC(19:0_18:1)	PC	40026385.11
PC(18:0_20:4)	PC	1548569796
PC(17:0_18:1)	PC	137347847
PC(18:0_22:5)	PC	112296724.3
PC(34:2)	PC	1418438.406
PC(34:1)	PC	5343852.992
PC(19:2)	PC	5979774.758
PC(16:0_18:2)	PC	2266312.668
PC(19:1_18:1)	PC	189309592.4

PC(22:3)	PC	2571435.011
PC(18:1_18:3)	PC	1735463.393
PC(16:1_22:6)	PC	1266657.902
PC(20:5_18:2)	PC	1406292.356
PC(38:7)	PC	1255944.116
PC(26:2)	PC	531005.6265
PC(15:0_22:5)	PC	2188536.966
PE(18:0p_18:2)	PE	29482139.05
PE(62:1)	PE	3806842.427
PE(16:0p_22:5)	PE	14133148.23
PE(18:0_20:4)	PE	31389961.83
PE(16:0_18:2)	PE	13581587.76
PE(18:0_18:1)	PE	13272329.91
PE(16:0_20:4)	PE	4380392.943
PE(18:2e)	PE	1024116.13
PE(16:0e)	PE	1391980.662
PE(21:4)	PE	394434.6412
PI(18:0_20:3)	PI	3415821.896
PI(18:0_20:4)	PI	4880165.634
SM(t40:4)	SM	13538933.85
SM(d18:1_24:2)	SM	85495183.96
SM(d42:2)	SM	74399840.63
SM(d34:0)	SM	11982240.08
SM(d42:4)	SM	5342996.376
SM(d18:1_23:1)	SM	39527740.72
SM(d20:1_20:1)	SM	32898716.84
SM(d41:3)	SM	11923037.91
SM(d39:2)	SM	11866471.63
SM(d35:1)	SM	2752230.736
SM(d17:1_21:1)	SM	25625273.25

SM(d44:5)	SM	9092973.819
SPH(d18:1)	SPH	7374550.555
SPH(t18:0)	SPH	2021184.298
TG(18:1_18:1_16:0)	TG	2552811830
TG(16:0_18:1_18:3)	TG	134212209.6
TG(12:1e_6:0_18:3)	TG	82660984.49
TG(16:0_16:0_18:3)	TG	46877977.02
TG(18:1_18:2_20:3)	TG	27957296.59
TG(12:0e_6:0_20:5)	TG	36769913.92
TG(16:0_16:0_18:2)	TG	74107661.35
TG(18:1_18:1_18:3)	TG	48644297.61
TG(20:0e_16:0_18:0)	TG	5165636.531
TG(20:2e_18:3_20:4)	TG	77104076.4
TG(18:0e_18:2_16:0)	TG	5149099.001
TG(18:1e_16:0_16:0)	TG	5658015.756
TG(36:4)	TG	26351842.44
TG(18:0_18:1_22:6)	TG	22153313.8
TG(20:0e_18:0_18:1)	TG	3449872.455
TG(18:2_10:2_10:2)	TG	12439798.59
TG(18:4_16:0_18:2)	TG	19928923.94
TG(8:0_10:2_18:2)	TG	11923544.25
TG(18:3_18:2_18:2)	TG	18454018.82
TG(18:0_18:1_18:3)	TG	7993082.734
TG(6:0_12:2_18:1)	TG	12439798.59
TG(6:0_14:4_18:1)	TG	1973435.269
TG(20:0e_18:0_18:2)	TG	4783997.818
TG(12:0_12:0_14:0)	TG	3076023.251
TG(18:0_18:3_18:3)	TG	6174031.977
TG(14:0_18:2_18:3)	TG	7112587.295
TG(18:3e_18:4_22:3)	TG	8563018.831

TG(18:1_18:2_18:3)	TG	18191741.19
TG(6:0_11:2_18:3)	TG	3048748.858
TG(6:0_14:4_18:2)	TG	5073929.784
TG(16:0_6:0_12:2)	TG	8461888.435
TG(8:0_10:2_18:1)	TG	6285059.372
TG(20:0e_16:0_16:0)	TG	7911119.205
TG(6:0_11:1_14:2)	TG	2297189.461
TG(20:3e_18:1_18:3)	TG	15813622.1
TG(6:0_12:2_18:2)	TG	3809935.047
TG(8:0_10:1_18:1)	TG	3069349.426
TG(20:0e_16:0_17:0)	TG	3045627.992
TG(18:2_18:2_16:0)	TG	5779864.361
TG(20:0e_18:2_16:0)	TG	9356858.575
WE(5:0_15:2)	WE	47727155.77
ZyE(18:3)	ZyE	12483440.36
ZyE()	ZyE	149416174.3
ZyE(0:0)	ZyE	3997474.597

duced in spent media relative to complete media, including peak area quantification and statistical si

Group Area: SM_pos_12022024_Newanalyis	FoldChange_CM_SM	log2FC
556264.8658	8.5741877	3.1
543135.4869	30.69645182	4.94
286357.5009	7.516181994	2.91
473478.5476	21.70566924	4.44
4343146.588	5.351710219	2.42
347634.9058	191.3407038	7.58
296106.2091	95.67035191	6.58
532719.6069	85.0358921	6.41
649714.7414	67.6491546	6.08
302742.6198	20.67764529	4.37
221217.7613	20.11221399	4.33
461679.3612	17.3877578	4.12
683779.6215	15.77972327	3.98
748940.5333	11.87618857	3.57
482874.1689	8.75434961	3.13
305666.1131	7.568461174	2.92
236045.5276	7.06162397	2.82
366326.3953	4.658934346	2.22
511606.1759	1160.073099	10.18
547455.5313	162.016844	7.34
622560.9412	106.1529019	6.73
550212.9304	28.8400148	4.85
479095.4584	35.01739844	5.13
354856.7114	29.04061297	4.86
304643.8749	28.8400148	4.85
1258492.012	114.5632091	6.84
937316.5875	100.4267645	6.65
785824.7705	92.41146851	6.53
812990.3708	85.0358921	6.41

1125362.044	74.02804377	6.21
12622007.3	73.00887782	6.19
421578.8123	58.08122594	5.86
8826864.755	54.94818793	5.78
658900.0384	54.56863307	5.77
1630449.005	50.91433496	5.67
664963.6559	49.8665331	5.64
513058.5965	35.75318842	5.16
553809.7134	22.31589866	4.48
430366.2119	21.11212657	4.4
118200.1874	20.2521055	4.34
582145.7671	18.50701094	4.21
589321.0758	18.50701094	4.21
553391.7157	17.02992292	4.09
440095.7847	13.64215827	3.77
11436611.13	13.45434264	3.75
649102.4726	12.81711804	3.68
698059.8871	6.821079134	2.77
5737373.248	6.680703355	2.74
570481.4159	6.634556367	2.73
144333.0486	32.67238802	5.03
124962.8102	1438.151553	10.49
240452.7873	855.1300295	9.74
165488.5066	436.5490646	8.77
152456.8809	347.2907078	8.44
85628.46588	235.5680386	7.88
221860.2372	229.1264182	7.84
266425.4614	77.17170097	6.27
186688.9579	49.8665331	5.64
168741.1407	47.83517596	5.58

579999.952	44.32350298	5.47
634413.4754	31.12495832	4.96
556788.9407	28.64080227	4.84
230464.3112	28.4429658	4.83
1128302.74	26.90868529	4.75
701452.2912	25.8125363	4.69
93832.29659	20.82146969	4.38
155004.9056	20.11221399	4.33
219364.847	17.75311155	4.15
278436.4802	16.1112888	4.01
846065.8357	15.03236399	3.91
213618.541	15.03236399	3.91
98679.4065	12.04197398	3.59
709216.242	10.77786861	3.43
1639544.212	9.713559075	3.28
212260.1849	8.633825892	3.11
125964.5756	8.224910613	3.04
204777.6927	8.111675838	3.02
377725.3466	6.821079134	2.77
116460.314	5.775716782	2.53
330016.6515	5.656854249	2.5
86232.94787	4.0278222	2.01
122677.5884	87.42657643	6.45
98216.73907	1573.760186	10.62
210830.5495	7.012845771	2.81
389371.0307	4.890561111	2.29
1702239.462	786.8800928	9.62
1927297.384	205.0738887	7.68
2748468.373	56.10276616	5.81
744250.083	42.81368175	5.42

2359871.892	41.64293937	5.38
183651.9958	40.50421101	5.34
5948923.284	32.89964245	5.04
1845717.698	32.44670335	5.02
407872321.7	28.8400148	4.85
278883.9911	27.09585	4.76
5219257.796	24.93326655	4.64
8227254.232	22.16175149	4.47
120933.064	21.25897303	4.41
133574.982	19.8353232	4.31
258740.2403	18.00093576	4.17
1350353.591	17.3877578	4.12
3696420.806	16.44982123	4.04
169220.2821	14.6213032	3.87
4707351.307	14.32040113	3.84
153514.5384	14.32040113	3.84
1399349.406	14.12324794	3.82
834972.6153	13.73704698	3.78
1342182.362	13.73704698	3.78
901040.975	12.90626815	3.69
113537.2042	12.90626815	3.69
3447693.754	11.63178014	3.54
147118556.5	10.55606329	3.4
14782350.8	9.317868692	3.22
13078540.76	8.5741877	3.1
181299.9514	7.835362381	2.97
727928.4849	7.361501205	2.88
819093.0431	7.310651602	2.87
342755.4723	6.634556367	2.73
30952063.56	6.105036836	2.61

421309.0099	6.105036836	2.61
294720.7519	5.897076869	2.56
228285.1951	5.540437872	2.47
274613.6296	5.13370359	2.36
245529.3688	5.098242509	2.35
111068.9737	4.789914818	2.26
528556.2983	4.141059695	2.05
839330.5501	35.01739844	5.13
198723.6099	19.15965927	4.26
839950.4487	16.79546694	4.07
3622163.058	8.6938789	3.12
1658593.525	8.168097006	3.03
1651479.8	8.0556444	3.01
721228.3503	6.062866266	2.6
176577.9386	5.815890069	2.54
243689.7782	5.696200782	2.51
93932.86983	4.198866734	2.07
131871.2749	25.99207668	4.7
583516.9341	8.339726087	3.06
147691.691	91.77313587	6.52
998586.6192	85.62736351	6.42
1449428.785	51.26847217	5.68
270660.432	44.32350298	5.47
123153.4225	43.41133848	5.44
1353986.341	29.24260641	4.87
2091001.154	15.77972327	3.98
891895.1532	13.36140671	3.74
887663.7489	13.36140671	3.74
207695.5744	13.26911273	3.73
2516236.994	10.19648502	3.35

1601516.113	5.696200782	2.51
1127459.323	6.543216468	2.71
458216.9637	4.407620464	2.14
4177550.338	613.1090968	9.26
986004.3944	136.2393834	7.09
740855.0225	111.4304721	6.8
730946.0085	64	6
534943.3553	52.34573175	5.71
771281.0831	47.83517596	5.58
1630907.48	45.56960626	5.51
1543080.557	31.55944654	4.98
174056.9685	29.65081798	4.89
3103167.851	24.7610399	4.63
208745.4208	24.5900029	4.62
232871.5306	24.25146506	4.6
1153358.938	22.78480313	4.51
1059797.092	20.96629446	4.39
166462.1922	20.67764529	4.37
793544.8337	15.67072476	3.97
1287667.636	15.45498126	3.95
808773.0261	14.72300241	3.88
1412411.188	13.08643294	3.71
650210.5517	12.29500145	3.62
1033137.153	12.04197398	3.59
164991.6246	11.95879399	3.58
416051.6702	11.47164198	3.52
317504.5701	9.713559075	3.28
688789.0554	8.938297105	3.16
841039.1836	8.456144324	3.08
1158141.452	7.412704495	2.89

2488852.751	7.310651602	2.87
432015.41	7.06162397	2.82
759895.5514	6.680703355	2.74
1318744.301	6.408559021	2.68
990569.0879	6.36429187	2.67
1364564.068	5.815890069	2.54
399905.3708	5.735820992	2.52
3113069.061	5.063026376	2.34
777678.6775	4.890561111	2.29
670110.5412	4.59479342	2.2
681484.2473	4.469148552	2.16
1289028.558	4.469148552	2.16
2277039.583	4.112455307	2.04
4453806.951	10.70342044	3.42
679030.3142	18.37917368	4.2
8947578.13	16.67945217	4.06
789545.098	5.063026376	2.34

gnificance metrics.

Adj. P-value: (CM_pos_12022024_Newanalysis) / (SM_pos_12022024_Newanalyis)	Significant
0.029971518	TRUE
3.07581E-07	TRUE
0.000769149	TRUE
0.000620209	TRUE
0.000109321	TRUE
3.21958E-11	TRUE
3.21958E-11	TRUE
7.19757E-09	TRUE
5.77401E-10	TRUE
6.1671E-11	TRUE
7.0499E-10	TRUE
1.80399E-06	TRUE
5.81838E-06	TRUE
0.001704921	TRUE
0.000131244	TRUE
7.70933E-06	TRUE
8.21587E-06	TRUE
0.015706223	TRUE
5.05684E-11	TRUE
3.66868E-08	TRUE
3.62528E-08	TRUE
2.03285E-07	TRUE
6.54609E-08	TRUE
1.16917E-05	TRUE
2.44673E-07	TRUE
5.78869E-10	TRUE
3.44703E-05	TRUE
3.21958E-11	TRUE
2.03418E-07	TRUE

2.11062E-10	TRUE
0.012971296	TRUE
3.48733E-05	TRUE
0.001723598	TRUE
0.000527338	TRUE
1.67081E-08	TRUE
0.000242621	TRUE
9.92014E-07	TRUE
1.80571E-10	TRUE
2.47474E-08	TRUE
4.94793E-07	TRUE
1.67811E-07	TRUE
0.00146678	TRUE
2.20289E-08	TRUE
6.83902E-09	TRUE
0.028574293	TRUE
9.49564E-07	TRUE
0.006428085	TRUE
0.002083661	TRUE
0.000218227	TRUE
1.64636E-10	TRUE
2.10258E-05	TRUE
9.82558E-08	TRUE
4.33783E-07	TRUE
2.07031E-09	TRUE
2.03921E-09	TRUE
4.91585E-05	TRUE
3.21957E-08	TRUE
3.21958E-11	TRUE
5.83738E-06	TRUE

1.41511E-05	TRUE
0.002126778	TRUE
8.74477E-05	TRUE
1.35139E-05	TRUE
5.42967E-07	TRUE
0.000104489	TRUE
1.96788E-05	TRUE
0.000156689	TRUE
2.6644E-06	TRUE
0.000218949	TRUE
3.39723E-06	TRUE
3.45541E-07	TRUE
1.66026E-05	TRUE
3.67159E-05	TRUE
0.001717704	TRUE
0.005579733	TRUE
0.038255801	TRUE
0.003843454	TRUE
0.012240684	TRUE
5.04328E-05	TRUE
0.011341514	TRUE
0.008615779	TRUE
6.51812E-06	TRUE
6.85544E-06	TRUE
0.002615311	TRUE
0.040305904	TRUE
2.70781E-09	TRUE
2.77373E-06	TRUE
1.07081E-05	TRUE
0.00037033	TRUE

0.000230207	TRUE
1.43773E-10	TRUE
5.37349E-07	TRUE
0.003190271	TRUE
0.029771282	TRUE
0.000176405	TRUE
0.000551205	TRUE
2.54891E-06	TRUE
0.000122973	TRUE
0.013156242	TRUE
0.000515997	TRUE
0.008398202	TRUE
0.000116159	TRUE
0.014322846	TRUE
3.73298E-06	TRUE
0.011046097	TRUE
4.57006E-06	TRUE
3.35141E-05	TRUE
0.000816137	TRUE
0.00307318	TRUE
0.001973351	TRUE
0.003229801	TRUE
0.015858725	TRUE
0.000164031	TRUE
0.005025985	TRUE
0.031684156	TRUE
0.000174417	TRUE
0.010967885	TRUE
2.62731E-05	TRUE
0.004911986	TRUE

0.000641955	TRUE
5.37213E-05	TRUE
0.022653896	TRUE
0.000198227	TRUE
0.000380772	TRUE
0.016417447	TRUE
0.04417354	TRUE
2.09823E-09	TRUE
0.000162034	TRUE
0.000577809	TRUE
9.48465E-05	TRUE
1.96712E-05	TRUE
0.048894017	TRUE
4.2037E-06	TRUE
0.00073715	TRUE
0.000955815	TRUE
0.018968559	TRUE
7.1973E-06	TRUE
0.003544484	TRUE
0.014531446	TRUE
6.63775E-05	TRUE
0.000153454	TRUE
4.17827E-05	TRUE
0.00012238	TRUE
1.67732E-05	TRUE
0.000139131	TRUE
0.002180119	TRUE
0.002578532	TRUE
0.004200484	TRUE
0.002851188	TRUE

0.003566022	TRUE
0.014800638	TRUE
0.029117655	TRUE
4.31502E-09	TRUE
4.88888E-11	TRUE
1.21359E-10	TRUE
3.21958E-11	TRUE
1.92324E-10	TRUE
4.9916E-11	TRUE
0.000101123	TRUE
5.49754E-05	TRUE
0.000693021	TRUE
4.14816E-06	TRUE
1.15002E-07	TRUE
1.3025E-08	TRUE
3.43884E-11	TRUE
1.43992E-05	TRUE
0.004934026	TRUE
1.76643E-09	TRUE
8.22174E-08	TRUE
7.6992E-11	TRUE
0.000549912	TRUE
5.95667E-06	TRUE
2.52171E-09	TRUE
3.93178E-10	TRUE
0.000401457	TRUE
8.17059E-05	TRUE
0.001313559	TRUE
2.78331E-07	TRUE
0.004609884	TRUE

0.012218122	TRUE
0.002422859	TRUE
1.04491E-05	TRUE
0.009925619	TRUE
0.013126995	TRUE
0.000529542	TRUE
0.000708552	TRUE
0.002750229	TRUE
3.09895E-07	TRUE
0.000732679	TRUE
0.020787141	TRUE
0.006682818	TRUE
0.001000021	TRUE
0.003873774	TRUE
5.91208E-06	TRUE
2.30305E-06	TRUE
0.000763856	TRUE

Reduced_in_SM
TRUE
TRUE
TRUE
TRUE
TRUE
TRUE
TRUE
TRUE
TRUE
TRUE
TRUE
TRUE
TRUE
TRUE
TRUE
TRUE
TRUE
TRUE
TRUE
TRUE
TRUE
TRUE
TRUE
TRUE
TRUE
TRUE
TRUE
TRUE
TRUE

TRUE
TRUE
TRUE
TRUE
TRUE
TRUE
TRUE
TRUE
TRUE
TRUE
TRUE
TRUE
TRUE
TRUE
TRUE
TRUE
TRUE
TRUE
TRUE
TRUE
TRUE
TRUE
TRUE
TRUE
TRUE
TRUE
TRUE
TRUE
TRUE
TRUE

TRUE
TRUE
TRUE
TRUE
TRUE
TRUE
TRUE
TRUE
TRUE
TRUE
TRUE
TRUE
TRUE
TRUE
TRUE
TRUE
TRUE
TRUE
TRUE
TRUE
TRUE
TRUE
TRUE
TRUE
TRUE
TRUE
TRUE
TRUE
TRUE
TRUE

TRUE
TRUE
TRUE
TRUE
TRUE
TRUE
TRUE
TRUE
TRUE
TRUE
TRUE
TRUE
TRUE
TRUE
TRUE
TRUE
TRUE
TRUE
TRUE
TRUE
TRUE
TRUE
TRUE
TRUE
TRUE
TRUE
TRUE
TRUE
TRUE
TRUE

TRUE
TRUE
TRUE
TRUE
TRUE
TRUE
TRUE
TRUE
TRUE
TRUE
TRUE
TRUE
TRUE
TRUE
TRUE
TRUE
TRUE
TRUE
TRUE
TRUE
TRUE
TRUE
TRUE
TRUE
TRUE
TRUE
TRUE
TRUE
TRUE
TRUE

TRUE
TRUE
TRUE
TRUE
TRUE
TRUE
TRUE
TRUE
TRUE
TRUE
TRUE
TRUE
TRUE
TRUE
TRUE
TRUE
TRUE
TRUE
TRUE
TRUE
TRUE
TRUE
TRUE
TRUE
TRUE
TRUE
TRUE
TRUE
TRUE
TRUE

TRUE
TRUE
TRUE
TRUE
TRUE
TRUE
TRUE
TRUE
TRUE
TRUE
TRUE
TRUE
TRUE
TRUE
TRUE
TRUE
TRUE
