# Supplementary material for: A lipid compendium of a metabolically compromised bacterium provides insights into lipid acquisition, biosynthesis, and metabolism
Source: bioRxiv. 2026 May 23:2026.05.22.727245. Preprint. [Version 1] doi: 10.64898/2026.05.22.727245 (PMC13228262; doi:10.64898/2026.05.22.727245)
Supplement: Supplement 3 [file media-3.pdf]

Supplementary spreadsheet 3: Untargeted lipidomics analysis of ESI-neagtive mode data obtained via (

Name	Formula	Annot. DeltaM	Calc. MW	m/z	RT [min]	MS Depth	Reference Ion
WE(47:0)	C47 H94 O2	-3.69	690.72283	689.71555	19.813	1	[M-H]-1
WE(45:0)	C45 H90 O2	-1.97	662.69278	661.6855	19.245	2	[M-H]-1
WE(43:0)	C43 H86 O2	-1.55	634.6618	633.65452	18.836	2	[M-H]-1
WE(26:0)	C26 H52 O2	-1.92	396.39597	395.38869	12.146	2	[M-H]-1
WE(25:0)	C25 H50 O2	-3.16	382.37987	381.37259	8.634	2	[M-H]-1
WE(23:0)	C23 H46 O2	-3.09	354.34868	353.34141	9.742	2	[M-H]-1
ST(d42:2)	C48 H91 N O1	-3.61	889.62807	888.6208	9.727	2	[M-H]-1
ST(d42:1)	C48 H93 N O1	-3.66	891.64367	890.63639	10.702	2	[M-H]-1
ST(d41:1)	C47 H91 N O1	-3.73	877.62801	876.62073	10.209	2	[M-H]-1
ST(d40:1)	C46 H89 N O1	-3.63	863.6125	862.60522	9.746	2	[M-H]-1
ST(d34:1)	C40 H77 N O1	-4.77	779.51801	778.51074	6.895	2	[M-H]-1
SQMG(16:0)	C25 H48 O11	-2.69	556.29024	555.28296	1.865	1	[M-H]-1
SQDG(16:0_18:3)	C43 H76 O12	-2.54	816.50367	815.4964	6.605	2	[M-H]-1
SQDG(16:0_18:2)	C43 H78 O12	-1.46	818.5202	817.51292	7.218	1	[M-H]-1
SQDG(16:0_16:0)	C41 H78 O12	-0.97	794.52063	793.51335	7.795	1	[M-H]-1
SM(d42:2)	C47 H93 N2 C	-0.12	812.67703	871.69086	16.531	2	[M-H+HAc]-1
SM(d41:2)	C46 H91 N2 C	-2.2	798.65972	797.65041	15.978	2	[M-H]-1
PS(18:1e_20:4)	C44 H78 N O9	-1.77	795.54001	794.53274	8.762	2	[M-H]-1
PS(18:1e_18:2)	C42 H78 N O9	-1.63	771.54016	770.53289	8.928	2	[M-H]-1
PI(18:1_20:4)	C47 H81 O13	-2.06	884.53965	883.53238	7.63	2	[M-H]-1
PI(18:1_18:2)	C45 H81 O13	-2.31	860.53949	859.53222	7.792	2	[M-H]-1
PI(18:0_22:6)	C49 H83 O13	-0.19	910.55696	909.54968	8.169	2	[M-H]-1
PI(18:0_22:5)	C49 H85 O13	-1.18	912.57171	911.56443	8.463	2	[M-H]-1
PI(18:0_20:4)	C47 H83 O13	-2.39	886.55501	885.54773	8.102	1	[M-H]-1
PI(18:0_20:3)	C47 H85 O13	-2.23	888.5708	887.56352	9.179	2	[M-H]-1
PI(18:0_20:2)	C47 H87 O13	-3.64	890.58518	889.57791	9.475	2	[M-H]-1
PI(18:0_18:2)	C45 H83 O13	-2.41	862.55505	861.54778	8.565	2	[M-H]-1
PI(18:0_18:1)	C45 H85 O13	-1.64	864.57136	863.56408	9.36	2	[M-H]-1
PI(16:0_22:6)	C47 H79 O13	-1.9	882.52415	881.51687	6.791	1	[M-H]-1
PI(16:0_20:4)	C45 H79 O13	-1.43	858.5246	857.51733	7.391	2	[M-H]-1
PI(16:0_18:2)	C43 H79 O13	-1.78	834.52435	833.51707	7.517	2	[M-H]-1
PI(16:0_18:1)	C43 H81 O13	-1.01	836.54063	835.53335	8.309	2	[M-H]-1
PG(20:0e_16:0)	C42 H85 O9 P	1.71	764.59443	763.58715	13.549	2	[M-H]-1

PG(18:1_18:2)	C42 H77 O10	-1.1	772.52459	771.51731	7.859	2	[M-H]-1
PG(18:1_18:1)	C42 H79 O10	-1.62	774.53983	773.53256	8.375	1	[M-H]-1
PG(18:0_18:1)	C42 H81 O10	-1.23	776.55578	775.54851	9.648	2	[M-H]-1
PG(18:0_16:0)	C40 H79 O10	-1.26	750.54014	749.53284	9.648	2	[M-H]-1
PG(16:1_18:2)	C40 H73 O10	-1.01	744.49338	743.48611	7.139	1	[M-H]-1
PG(16:0_18:2)	C40 H75 O10	-1.66	746.50854	745.50127	7.819	2	[M-H]-1
PG(16:0_18:1)	C40 H77 O10	-1.1	748.52461	747.51733	8.602	2	[M-H]-1
PG(16:0_17:0)	C39 H77 O10	-1.54	736.5243	735.51702	8.832	1	[M-H]-1
PG(16:0_16:1)	C38 H73 O10	-1.26	720.49323	719.48595	7.599	2	[M-H]-1
PG(16:0_16:0)	C38 H75 O10	-1.52	722.50868	721.50141	8.477	2	[M-H]-1
PG(16:0_14:0)	C36 H71 O10	-1.57	694.47739	693.47011	7.452	2	[M-H]-1
PG(15:0_16:0)	C37 H73 O10	-1.65	708.49297	707.48569	7.961	2	[M-H]-1
PEt(34:2e)	C39 H75 O7 P	6.22	686.52931	685.52203	4.137	1	[M-H]-1
PE(24:0_14:4)	C43 H78 N O8	-1.35	767.54547	766.53825	9.155	2	[M-H]-1
PE(22:0_14:4)	C41 H74 N O8	-0.93	739.51452	738.50724	7.425	1	[M-H]-1
PE(20:1e_22:6)	C47 H82 N O7	-1.03	803.58206	802.57479	13.334	2	[M-H]-1
PE(20:1e_18:2)	C43 H82 N O7	-1.66	755.58163	754.57437	14.127	2	[M-H]-1
PE(18:2e_22:6)	C45 H76 N O7	-1.96	773.53442	772.52714	10.824	2	[M-H]-1
PE(18:2e_22:5)	C45 H78 N O7	-2.36	775.54976	774.54248	11.857	2	[M-H]-1
PE(18:2e_20:5)	C43 H74 N O7	-2.01	747.51879	746.51151	9.999	2	[M-H]-1
PE(18:2e_20:4)	C43 H76 N O7	0.02	749.53595	748.52867	11.386	2	[M-H]-1
PE(18:2e_18:2)	C41 H76 N O7	-2.4	725.5342	724.52692	11.495	2	[M-H]-1
PE(18:2_20:4)	C43 H74 N O8	-0.74	763.51464	762.50737	9.354	2	[M-H]-1
PE(18:2_18:2)	C41 H74 N O8	-1.17	739.51434	738.50707	9.612	2	[M-H]-1
PE(18:1e_22:5)	C45 H80 N O7	-2.31	777.56544	776.55817	12	1	[M-H]-1
PE(18:1e_20:4)	C43 H78 N O7	-1.51	751.55046	750.54318	12.293	2	[M-H]-1
PE(18:1e_20:3)	C43 H80 N O7	-1.49	753.56612	752.55884	12.999	2	[M-H]-1
PE(18:1e_18:2)	C41 H78 N O7	-2.61	727.54969	726.54242	12.4	2	[M-H]-1
PE(18:1e_18:1)	C41 H80 N O7	-1.38	729.56623	728.55897	13.745	2	[M-H]-1
PE(18:1_22:6)	C45 H76 N O8	-0.18	789.53072	788.52344	9.987	2	[M-H]-1
PE(18:1_20:4)	C43 H76 N O8	-1.86	765.52943	746.5116	10.515	2	[M-H-H2O]-1
PE(18:1_18:2)	C41 H76 N O8	-1.13	741.53002	740.52274	10.587	2	[M-H]-1
PE(18:1_18:1)	C41 H78 N O8	-1.47	743.54541	742.53847	11.303	2	[M-H]-1
PE(18:0_22:6)	C45 H78 N O8	0.43	791.54685	772.52899	10.797	2	[M-H-H2O]-1
PE(18:0_22:5)	C45 H80 N O8	-0.02	793.56214	792.55207	11.867	2	[M-H]-1

PE(18:0_20:4)	C43 H78 N O8	-1.57	767.5453	766.53802	12.061	1	[M-H]-1
PE(18:0_20:3)	C43 H80 N O8	-2.53	769.56021	768.55296	11.878	2	[M-H]-1
PE(18:0_18:2)	C41 H78 N O8	-1.23	743.54559	742.53831	11.817	2	[M-H]-1
PE(18:0_18:1)	C41 H80 N O8	-0.62	745.56169	744.55206	12.571	2	[M-H]-1
PE(17:1_20:4)	C42 H74 N O8	-1.13	751.51436	750.50699	8.146	2	[M-H]-1
PE(17:1_16:0)	C38 H74 N O8	-0.83	703.51462	702.50734	10.957	1	[M-H]-1
PE(17:0_20:4)	C42 H76 N O8	-1.14	753.52999	752.52272	10.865	2	[M-H]-1
PE(17:0_18:2)	C40 H76 N O8	-0.27	729.53066	728.52338	11.112	2	[M-H]-1
PE(16:1e_22:6)	C43 H74 N O7	-0.51	747.51991	746.51263	10.148	2	[M-H]-1
PE(16:1e_22:4)	C43 H78 N O7	-2.38	751.5498	750.54253	11.258	2	[M-H]-1
PE(16:1e_20:5)	C41 H72 N O7	-1.06	721.50388	720.4966	9.993	2	[M-H]-1
PE(16:1e_20:4)	C41 H74 N O7	-1.1	723.51949	722.51221	10.873	2	[M-H]-1
PE(16:1e_18:2)	C39 H74 N O7	-0.99	699.5196	698.51232	11.192	2	[M-H]-1
PE(16:1e_18:1)	C39 H76 N O7	-1.49	701.53489	700.52762	12.277	2	[M-H]-1
PE(16:0e_18:2)	C39 H76 N O7	-1.29	701.53503	700.52776	11.44	2	[M-H]-1
PE(16:0e_18:1)	C39 H78 N O7	-0.92	703.55094	702.54366	12.537	2	[M-H]-1
PE(16:0_22:6)	C43 H74 N O8	-1.83	763.51381	762.50653	9.897	2	[M-H]-1
PE(16:0_22:4)	C43 H78 N O8	-2	767.54497	766.53832	10.979	2	[M-H]-1
PE(16:0_20:5)	C41 H72 N O8	-0.74	737.49901	736.49173	9.378	2	[M-H]-1
PE(16:0_20:4)	C41 H74 N O8	-1.68	739.51396	738.50682	9.898	2	[M-H]-1
PE(16:0_18:2)	C39 H74 N O8	-0.81	715.51463	714.50735	10.468	2	[M-H]-1
PE(16:0_18:1)	C39 H76 N O8	-1.31	717.52991	716.52263	11.484	2	[M-H]-1
PC(44:6e)	C52 H94 N O7	-4.91	875.67249	874.66521	15.987	1	[M-H]-1
PC(44:5e)	C52 H96 N O7	-4.5	877.68849	876.68121	18.302	1	[M-H]-1
PC(36:6e)	C44 H78 N O7	-3.37	763.54901	808.54755	9.406	2	[M+FA-H]-1
PC(30:1_20:4)	C58 H106 N C	-1.06	975.76457	974.75729	14.702	1	[M-H]-1
PC(20:5_18:2)	C46 H78 N O8	-1.02	803.54569	802.53816	8.971	2	[M-H]-1
PC(18:1_20:4)	C46 H82 N O8	0.11	807.57789	806.57062	9.499	1	[M-H]-1
PC(17:1_20:4)	C45 H80 N O8	-0.91	793.56143	792.55415	9.313	2	[M-H]-1
PC(17:0_20:3)	C45 H84 N O8	-2.8	797.59122	856.6084	12.311	1	[M-H+HAc]-1
PC(17:0_18:2)	C43 H82 N O8	-2	771.57626	816.57449	11.347	2	[M+FA-H]-1
PC(16:0_20:4)	C44 H80 N O8	-0.5	781.56177	780.55449	11.834	1	[M-H]-1
PC(16:0_18:2)	C42 H80 N O8	-0.42	757.56183	756.5543	12.251	1	[M-H]-1
PC(16:0_18:1)	C42 H82 N O8	-0.85	759.57716	758.56989	13.623	1	[M-H]-1
PC(15:0_22:6)	C45 H78 N O8	-0.53	791.54609	790.53873	9.34	2	[M-H]-1

PC(15:0_20:4)	C43 H78 N O8	-3.5	767.54382	766.53224	10.243	2	[M-H]-1
Palmitic acid_putative)			256.16702	255.15975	2.334	2	[M-H]-1
PA(36:1e)	C39 H77 O7 P	-3.61	688.53821	687.53093	4.28	1	[M-H]-1
PA(36:0e)	C39 H79 O7 P	9.33	690.56279	689.55551	6.258	2	[M-H]-1
PA(35:0)	C38 H75 O8 P	1.7	690.52113	689.51385	13.315	1	[M-H]-1
OAHFA(44:9)	C44 H68 O4	7.11	660.51646	659.50918	3.577	2	[M-H]-1
OAHFA(42:10)	C42 H62 O4	-6.16	630.46093	629.45365	3.654	2	[M-H]-1
OAHFA(40:6)	C40 H66 O4	-0.95	610.49553	609.48825	3.88	2	[M-H]-1
OAHFA(38:6)	C38 H62 O4	-6.82	582.46084	581.45356	3.874	2	[M-H]-1
OAHFA(38:4)	C38 H66 O4	-5.97	586.49261	585.48533	4.754	2	[M-H]-1
OAHFA(36:4)	C36 H62 O4	-1.59	558.46392	557.45665	3.643	2	[M-H]-1
OAHFA(36:3)	C36 H64 O4	-0.72	560.48005	559.47278	4.1	1	[M-H]-1
OAHFA(36:1)	C36 H68 O4	-2.93	564.51011	563.50283	4.962	2	[M-H]-1
MGDG(42:4)	C51 H90 O10	-1.29	862.65228	861.64501	13.969	2	[M-H]-1
MGDG(18:1_18:2)	C45 H80 O10	-1.62	780.57388	839.58768	10.866	2	[M-H+HAc]-1
MGDG(18:1_18:1)	C45 H82 O10	0.09	782.59087	841.60468	11.884	2	[M-H+HAc]-1
MGDG(18:1_15:0)	C42 H78 O10	3.69	742.56224	741.55496	10.919	1	[M-H]-1
MGDG(18:0_18:2)	C45 H82 O10	6.31	782.59574	781.58846	13.328	1	[M-H]-1
MGDG(16:0_18:2)	C43 H78 O10	-1.29	754.55853	753.55104	10.707	2	[M-H]-1
MGDG(16:0_18:1)	C43 H80 O10	-1.67	756.57389	815.5877	11.726	2	[M-H+HAc]-1
LPI(18:0)	C27 H53 O12	-3.16	600.32557	599.31829	2.881	2	[M-H]-1
LPE(22:6)	C27 H44 N O7	-1.55	525.28472	524.27745	2.67	2	[M-H]-1
LPE(22:5)	C27 H46 N O7	-1.06	527.30063	526.29335	3.16	2	[M-H]-1
LPE(22:4)	C27 H48 N O7	-1.12	529.31625	528.30897	3.464	2	[M-H]-1
LPE(20:5)	C25 H42 N O7	-1.71	499.26903	498.26176	2.351	2	[M-H]-1
LPE(20:4)	C25 H44 N O7	-2.35	501.28436	500.27708	3.151	1	[M-H]-1
LPE(20:3)	C25 H46 N O7	0.13	503.30125	502.29398	3.159	2	[M-H]-1
LPE(18:2e)	C23 H46 N O6	-0.96	463.30583	462.29855	3.909	2	[M-H]-1
LPE(18:2)	C23 H44 N O7	-1.33	477.2849	476.27763	2.942	2	[M-H]-1
LPE(18:1e)	C23 H48 N O6	-1.37	465.32129	464.31401	4.956	2	[M-H]-1
LPE(18:1)	C23 H46 N O7	-1.25	479.30059	478.29331	3.544	2	[M-H]-1
LPE(18:0)	C23 H48 N O7	-1.84	481.31595	480.30868	4.472	2	[M-H]-1
LPE(16:1e)	C21 H44 N O6	-1.15	437.29012	436.28284	3.768	2	[M-H]-1
LPE(16:0)	C21 H44 N O7	-1.01	453.28508	452.27781	3.41	2	[M-H]-1
LPC(16:1)	C24 H48 N O7	0.95	493.31731	492.31	3.41	1	[M-H]-1

LPC(16:0)	C24 H50 N O7	-0.44	495.33227	494.32504	4.605	1	[M-H]-1
LPC(15:0)	C23 H48 N O7	2.34	481.31797	480.31049	2.819	1	[M-H]-1
LdMePE(18:2)	C25 H48 N O7	-1.16	505.31625	504.30863	3.137	2	[M-H]-1
LdMePE(18:1)	C25 H50 N O7	-2.32	507.33131	506.32404	3.463	1	[M-H]-1
LdMePE(18:0)	C25 H52 N O7	-2.72	509.34675	508.33948	4.613	1	[M-H]-1
LdMePE(16:0)	C23 H48 N O7	-0.96	481.31638	480.30885	3.653	2	[M-H]-1
Hex1Cer(d18:1_24:1)	C48 H91 N O8	-2.84	809.67217	868.68616	13.866	2	[M-H+HAc]-1
FA(24:5)	C24 H38 O2	-1.65	358.28659	357.27931	4.92	2	[M-H]-1
FA(24:4)	C24 H40 O2	-1.8	360.30218	359.29498	5.841	2	[M-H]-1
FA(22:6)	C22 H32 O2	-2.6	328.23938	327.2321	3.57	2	[M-H]-1
FA(22:5)	C22 H34 O2	-1.94	330.25524	329.24796	3.921	2	[M-H]-1
FA(22:4)	C22 H36 O2	-4.21	332.27013	331.26325	5.376	1	[M-H]-1
FA(20:5)	C20 H30 O2	-2.12	302.22394	301.21663	1.875	2	[M-H]-1
FA(20:4)	C20 H32 O2	-2.45	304.23948	303.23221	4.546	2	[M-H]-1
FA(18:4)	C18 H28 O2	-1.41	276.20854	275.20126	2.744	2	[M-H]-1
FA(18:2)	C18 H32 O2	-2.28	280.23959	279.23231	3.873	2	[M-H]-1
FA(16:1)	C16 H30 O2	-6.41	254.22295	253.21567	4.148	2	[M-H]-1
FA(14:1)	C14 H26 O2	-3	226.1926	225.18532	3.085	2	[M-H]-1
dMePE(18:2_20:4)	C45 H78 N O8	-2.37	791.54463	790.53735	8.378	2	[M-H]-1
dMePE(18:0_18:2)	C43 H82 N O8	-2.2	771.57611	770.56668	13.074	2	[M-H]-1
dMePE(17:1_20:4)	C44 H78 N O8	-0.77	779.54591	778.53931	9.333	2	[M-H]-1
dMePE(17:1_18:2)	C42 H78 N O8	-1.69	755.54523	754.53795	9.424	2	[M-H]-1
dMePE(16:1_18:2)	C41 H76 N O8	-1.86	741.52947	740.52233	8.887	1	[M-H]-1
dMePE(16:0e_18:2)	C41 H80 N O7	-1.57	729.5661	728.55882	12.891	2	[M-H]-1
Cer(t18:0_23:0)	C41 H83 N O4	-1.4	653.63129	712.64525	15.789	2	[M-H+HAc]-1
Cer(d19:1_24:1)	C43 H83 N O3	-2.05	661.63594	720.64978	16.223	2	[M-H+HAc]-1
Cer(d19:1_24:0)	C43 H85 N O3	-1.73	663.6518	722.66565	17.277	2	[M-H+HAc]-1
Cer(d18:2_23:0)	C41 H79 N O3	-0.15	633.6059	668.57472	15.348	2	[M+Cl]-1
Cer(d18:2_22:0)	C40 H77 N O3	-2.16	619.58901	618.58037	14.602	1	[M-H]-1
Cer(d18:1_24:2)	C42 H79 N O3	-1.68	645.60491	704.61872	14.569	2	[M-H+HAc]-1
Cer(d18:1_24:1)	C42 H81 N O3	-1.6	647.62061	706.63445	15.787	2	[M-H+HAc]-1
Cer(d18:1_24:0)	C42 H83 N O3	-2.13	649.63591	708.64976	16.983	2	[M-H+HAc]-1
Cer(d18:1_23:0)	C41 H81 N O3	-1.93	635.62042	694.63426	16.481	2	[M-H+HAc]-1
Cer(d18:1_22:0)	C40 H79 N O3	-1.63	621.60498	680.61883	15.62	2	[M-H+HAc]-1
Cer(d18:1_19:0)	C37 H73 N O3	0.07	579.55908	624.55737	13.047	1	[M+FA-H]-1

Cer(d18:1_18:0)	C36 H71 N O3	-2.72	565.54185	624.55565	12.756	2	[M-H+HAc]-1
Cer(d18:1_16:0)	C34 H67 N O3	-1.67	537.5112	596.52505	11.32	3	[M-H+HAc]-1
Cer(d18:0_24:1)	C42 H83 N O3	-1.57	649.63628	694.63446	16.308	2	[M+FA-H]-1
Cer(d18:0_18:0)	C36 H73 N O3	-2.19	567.5578	626.57166	13.329	2	[M-H+HAc]-1
Cer(d18:0_16:0)	C34 H69 N O3	-1.82	539.52676	598.54047	11.827	2	[M-H+HAc]-1
Cer(d17:1_22:0)	C39 H77 N O3	-0.97	607.58975	666.60358	15.096	2	[M-H+HAc]-1
Cer(d16:1_22:0)	C38 H75 N O3	-1.22	593.57397	628.54278	14.343	2	[M+Cl]-1
Cer(d16:1_16:0)	C32 H63 N O3	-1.39	509.48009	568.49393	9.982	2	[M-H+HAc]-1

Orbitrap-IDX

Log2 Fold Change: (Bb_neg_newanalysis_12022024) / (CM_neg_newanalysis_12022024)
-0.64
-0.75
-0.03
-0.25
-0.51
-0.11
-5.44
-3.15
-4.89
-4.82
-4.57
-0.11
1.66
0.23
0.87
-3.94
0.36
-1.57
5.54
-0.76
-0.63
0.7
-0.52
0.85
-1.88
-3.69
-5.52
-3.41
0.34
-1.46
-3.38
-2.94
-2.74

	6.04
	4.19
	7.2
	6.08
	4.42
	8.44
	7.68
	2.6
	6.78
	7.8
	5.43
	4.77
	-1.11
	-0.51
	0.5
	0.42
	-2.83
	1.81
	0.25
	-0.7
	-0.09
	-1.62
	1.27
	-2.39
	-0.79
	-4.13
	-2
	-4.95
	-2.05
	2.31
	0.09
	-3.47
	1.07
	1.22
	3.94

	3.63
	-0.28
	-1.82
	1.24
	0.39
	-0.15
	0.69
	-1.11
	1.52
	-0.04
	0.58
	-1.68
	-3.54
	-1.29
	-0.31
	0.39
	0.23
	-1.22
	0.95
	5.25
	-4.1
	-1.66
	0.56
	0.22
	0.64
	0.87
	1.14
	0.56
	-0.86
	-1.01
	0.92
	-0.19
	0.14
	0.12
	0.2

	0.08
	1.03
	-3.56
	0
	0.52
	-5.13
	-0.77
	-3.34
	-6.04
	-4.76
	-0.71
	0.38
	0.18
	5.79
	11.13
	8.15
	1
	0.59
	4.54
	12.22
	0.98
	-0.29
	-0.96
	-0.56
	0.26
	-0.76
	-0.73
	-2.57
	-1.91
	-3.63
	-1.68
	-3.26
	-2.21
	-3.8
	-0.39

	-1.06
	0.39
	1.28
	-1
	-1.71
	-2.63
	0.64
	-0.39
	-1.43
	-1.93
	-8.56
	0.65
	-1.79
	-0.37
	-2.33
	-4.86
	-1.47
	-3.89
	0.03
	-0.86
	0.08
	-1.06
	-1.14
	-2.3
	-2.46
	0.05
	-3.72
	-0.15
	0.71
	-3.42
	-5.61
	-5.86
	-5.65
	-1.74
	-3.74

-3.03
-2.96
-1.65
-1.09
-0.57
-2.03
-2.31
0.66

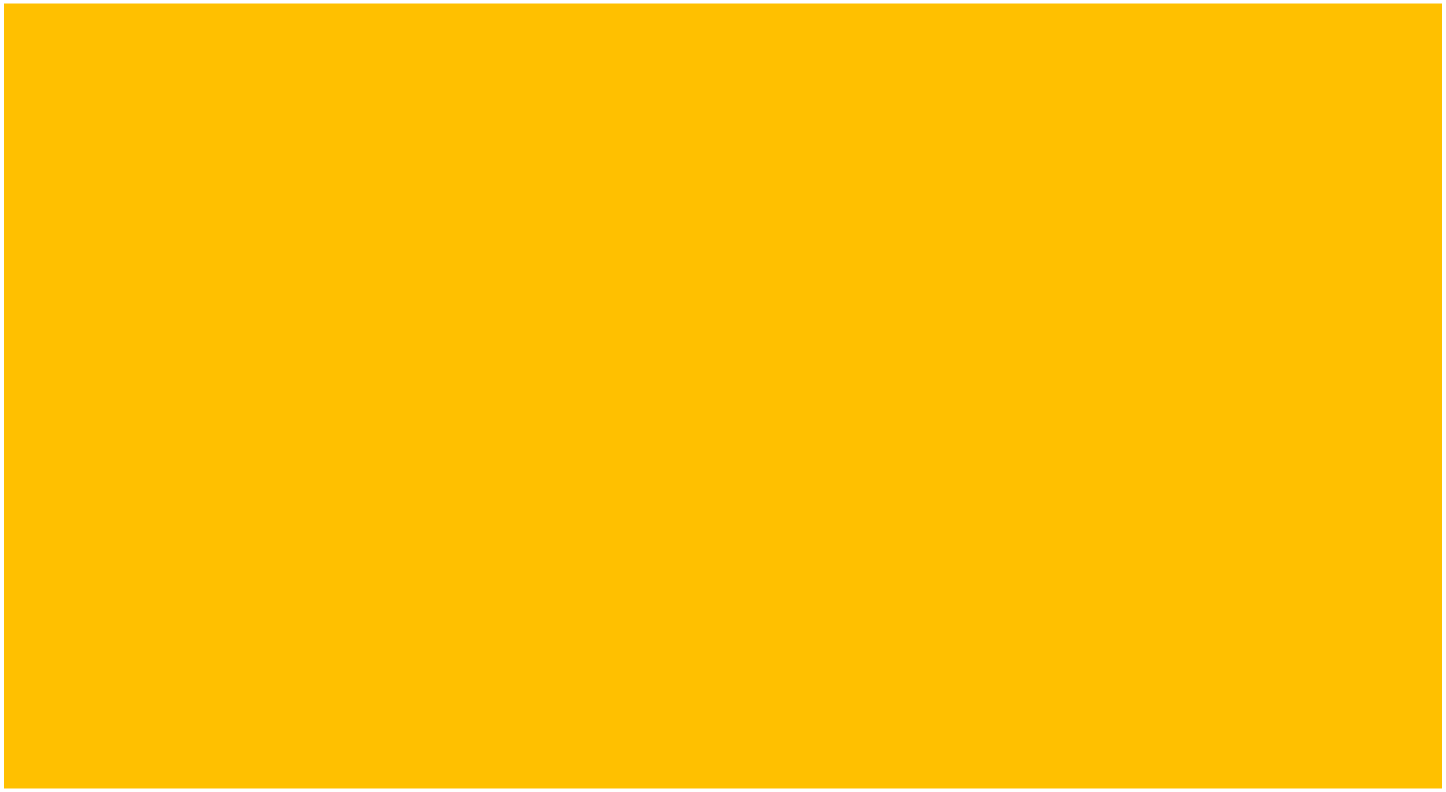

Log2 Fold Change: (Bb_neg_newanalysis_12022024) / (RS_NEG	Log2 Fold Change: (CM_neg_newanalysis_12022024) / (RS_NEG_
2.12	2.76
2.68	3.43
2.2	2.23
0.37	0.62
0.91	1.41
0.14	0.25
-5.08	0.36
-3.32	-0.17
-4.79	0.11
-4.81	0.01
-3.31	1.25
-1.95	-1.84
2.24	0.59
1.17	0.93
1.68	0.81
-6.03	-2.09
-1.4	-1.76
-3.87	-2.3
2.72	-2.81
0.98	1.74
-0.2	0.44
1.62	0.91
0.57	1.09
1.67	0.82
-1.64	0.24
-3.3	0.39
-5.21	0.31
-2.87	0.54
1.43	1.08
-0.81	0.65
-2.5	0.88
-2.17	0.77
-6.29	-3.55

4.36	-1.68
2.84	-1.35
8.63	1.43
5.15	-0.93
5.38	0.97
10.07	1.63
9.91	2.22
3.16	0.56
7.98	1.21
9.92	2.12
6.45	1.02
5.46	0.68
-5.3	-4.2
-1.25	-0.74
0.51	0.02
-0.05	-0.47
-4.52	-1.69
2.34	0.53
0.56	0.31
-2.16	-1.46
-4.04	-3.95
-3.51	-1.88
0.68	-0.59
-3.14	-0.75
3.41	4.21
-5.63	-1.51
-7.85	-5.85
-3.03	1.92
-4.45	-2.4
2.79	0.48
-0.37	-0.46
-4.5	-1.04
-1.23	-2.3
-3.51	-4.73
6.72	2.78

0	-3.63
-1.03	-0.75
-6.36	-4.54
0.69	-0.55
-1.23	-1.63
0.37	0.52
-0.95	-1.63
-2.96	-1.85
1.31	-0.21
-1.55	-1.51
-0.94	-1.53
-3.69	-2.01
-5.12	-1.58
-4.13	-2.84
-3.87	-3.56
-0.33	-0.72
-1.39	-1.62
-2.46	-1.24
1.99	1.04
3.5	-1.75
-5.08	-0.98
-3.73	-2.07
-0.75	-1.31
1.8	1.58
1.6	0.95
-0.25	-1.12
1.84	0.7
1.16	0.59
-0.77	0.09
-2.47	-1.46
-1.44	-2.36
0.03	0.22
1.18	1.04
0.94	0.82
1.1	0.91

-1.81	-1.89
2.64	1.61
-0.28	3.28
0.82	0.82
-1.39	-1.91
-7.75	-2.62
-6.89	-6.12
-7.28	-3.94
-7.56	-1.51
-7.21	-2.45
-7.05	-6.34
-4.76	-5.14
-4.28	-4.46
6.62	0.83
11.75	0.62
11.62	3.47
-1.36	-2.35
-0.06	-0.65
4.88	0.34
13.02	0.79
-2.68	-3.66
1.19	1.48
-0.58	0.39
-1.14	-0.58
1.44	1.17
0.98	1.74
0.13	0.86
-7.08	-4.51
-3.77	-1.86
-6.64	-3.01
-4.35	-2.67
-5.38	-2.12
-4.92	-2.71
-6.22	-2.41
-1.25	-0.86

-1.96	-0.9
1.04	0.66
-1.4	-2.68
-1.51	-0.51
-3.22	-1.51
-6.14	-3.51
-2.68	-3.33
-5.23	-4.84
-5.18	-3.75
-5.94	-4.01
-12.82	-4.26
-1.14	-1.78
-5.44	-3.65
-4.03	-3.66
-5.07	-2.74
-7.69	-2.82
-1.62	-0.15
-2.48	1.41
1.4	1.37
-3.81	-2.95
-1.38	-1.46
-3.1	-2.04
-2.38	-1.24
-4.41	-2.11
-6.23	-3.77
-3.17	-3.22
-6.32	-2.6
-2.1	-1.95
0.85	0.14
-7.31	-3.89
-10.04	-4.44
-9.05	-3.19
-8.91	-3.26
-6.13	-4.38
-3.34	0.4

-6.49	-3.46
-8.43	-5.47
-5.24	-3.59
-5.46	-4.37
-4.25	-3.68
-5.29	-3.25
-4.79	-2.48
-2.05	-2.71

Log2 Fold Change: (SM_neg_newanalysis_12022024) / (RS_NEG	Log2 Fold Change: (Bb_neg_newanalysis_12022024) / (SM_neg_newa
1.57	0.55
2.31	0.37
1.4	0.8
0.3	0.07
0.87	0.04
0.83	-0.69
1.13	-6.2
1.58	-4.9
1.8	-6.59
1.45	-6.26
1.84	-5.16
-1.3	-0.65
1.54	0.7
1.64	-0.47
2.89	-1.21
-1.99	-4.04
-1.91	0.51
-2.36	-1.51
-1.13	3.85
2.91	-1.94
1.26	-1.46
0.95	0.66
1.49	-0.92
1.03	0.64
1.84	-3.48
1.97	-5.27
1.43	-6.64
2.23	-5.1
0.71	0.71
2.19	-3
1.77	-4.28
2.2	-4.37
-3.26	-3.03

-1.93	6.29
-1.58	4.42
1.04	7.59
-1.67	6.81
0.88	4.5
1.38	8.68
0.84	9.06
0.47	2.69
0.98	7
2.51	7.42
0.77	5.68
0.56	4.9
-2.9	-2.4
-1.6	0.35
0.12	0.39
-0.16	0.1
-1.53	-2.98
0.64	1.69
-0.88	1.44
-2.88	0.72
-2.62	-1.42
-1.52	-1.99
-0.85	1.53
-0.46	-2.68
2.71	0.7
-1.37	-4.26
-6.4	-1.46
-1.22	-1.81
-1.51	-2.94
0.24	2.56
-0.96	0.59
-0.65	-3.85
-2.77	1.54
-4.69	1.18
0.23	6.49

-3.12	3.13
-2.07	1.05
-0.91	-5.46
-2.42	3.11
-1.57	0.33
-0.39	0.76
-1.41	0.46
-1.32	-1.65
-0.02	1.33
-2.05	0.5
-1.77	0.82
-1.46	-2.23
-1.32	-3.8
-1.77	-2.36
-3.2	-0.67
-1.81	1.48
-2.56	1.17
-0.13	-2.32
0.8	1.19
-1.71	5.21
-0.94	-4.14
-1.3	-2.43
-1.38	0.63
0.95	0.85
0.98	0.61
-1.08	0.83
0.73	1.11
0.78	0.38
-0.64	-0.12
-2.23	-0.24
-1.61	0.17
-1.67	1.69
0.07	1.11
0.15	0.79
0.67	0.43

-1.57	-0.24
-4.36	7
4.66	-4.94
0.62	0.2
-1.62	0.23
-1.75	-6
-4.25	-2.64
-2.87	-4.41
-0.65	-6.9
-1.73	-5.47
-5.43	-1.62
-5.12	0.36
-4.99	0.71
0.93	5.69
0.6	11.14
0.88	10.75
-2.6	1.24
0.15	-0.21
0.41	4.48
1.64	11.37
-3.06	0.38
0.74	0.45
-1.51	0.94
-1.44	0.3
0.82	0.62
-0.16	1.13
-1.04	1.17
-2.43	-4.66
-2.01	-1.76
-1.71	-4.93
-3.36	-0.98
-1.68	-3.7
-1.05	-3.87
-3.06	-3.15
-1.56	0.3

-0.72	-1.24
-0.1	1.15
-3.8	2.4
1.05	-2.56
-1.87	-1.35
-3.76	-2.38
-2.06	-0.62
-3.4	-1.83
-2.79	-2.39
-3.96	-1.98
-3.2	-9.63
-2.03	0.9
-2.5	-2.94
-2.97	-1.06
-2.24	-2.84
-1.97	-5.72
-0.46	-1.15
1.79	-4.28
0.75	0.65
-2.19	-1.62
-1.79	0.41
-2.12	-0.98
-1.95	-0.42
-1.74	-2.67
-3.21	-3.01
-2.94	-0.23
-2.84	-3.48
-2.99	0.9
0.02	0.83
-3.87	-3.44
-3.95	-6.09
-3.08	-5.97
-3.01	-5.9
-3.85	-2.28
-2.95	-0.39

-3.39	-3.1
-4.47	-3.96
-3.33	-1.91
-3.45	-2.01
-4.25	0
-2.99	-2.3
-1.8	-2.99
-2.95	0.9

Log2 Fold Change: (CM_neg_newanalysis_12022024) / (SM_neg_newar	Log2 Fold Change: (Bb_neg_newanalysis_12022024) / (WATER_NEG_NEWAN
1.19	-1.26
1.12	-1.34
0.83	0.24
0.33	-1.82
0.55	-1.54
-0.58	-0.48
-0.76	-1.46
-1.75	0
-1.69	-1.55
-1.44	-1.42
-0.59	-1.23
-0.54	-2.05
-0.96	-1.38
-0.7	-2.64
-2.08	-3.94
-0.1	-1.9
0.15	-1.25
0.06	-1.44
-1.68	5.23
-1.17	-1.32
-0.82	-1.12
-0.04	-1.18
-0.4	-1.1
-0.21	-0.98
-1.6	-1.64
-1.58	-1.58
-1.12	-2.29
-1.69	-1.36
0.37	-1.32
-1.54	-1.3
-0.89	-1.13
-1.43	-1.67
-0.29	-1.43

0.25	4.68
0.23	2.67
0.39	6.1
0.73	4.96
0.08	2.34
0.24	7.41
1.38	6.8
0.09	0.83
0.23	5.55
-0.38	7.29
0.25	3.76
0.12	2.84
-1.3	-0.69
0.86	-1.63
-0.11	-1.29
-0.31	-1.66
-0.16	-0.86
-0.12	-0.26
1.19	-0.24
1.42	-1
-1.33	-0.39
-0.36	-0.82
0.26	0.82
-0.29	-1.57
1.49	-1.31
-0.13	-1.22
0.55	-1.42
3.14	-1.25
-0.89	-1.4
0.24	0.49
0.5	-1.28
-0.38	-1.25
0.47	2.59
-0.04	-0.76
2.55	5

-0.51	1.32
1.32	-0.1
-3.64	-1.77
1.87	2.28
-0.06	-1.41
0.91	-1.01
-0.22	0.02
-0.54	-0.96
-0.19	-0.42
0.53	-0.72
0.24	-1.12
-0.55	-0.04
-0.26	-1.08
-1.06	-1.22
-0.35	-0.75
1.09	-0.42
0.94	-0.68
-1.11	-1.16
0.24	-0.92
-0.04	3.53
-0.04	-0.91
-0.77	0.2
0.07	-1.21
0.63	-1.19
-0.03	-1.34
-0.04	-1.1
-0.03	-0.71
-0.19	-1.49
0.74	-1.68
0.78	-1.3
-0.75	3.55
1.89	-0.21
0.98	-0.66
0.68	-0.95
0.23	-1.59

-0.32	-1.4
5.97	5.77
-1.38	-0.99
0.2	-1.01
-0.29	-1.45
-0.87	-1.42
-1.87	-1.17
-1.07	-1.16
-0.86	-0.83
-0.71	-0.62
-0.91	-1.17
-0.02	-1.44
0.53	-0.78
-0.1	3.82
0.01	9.2
2.59	9.26
0.25	-0.85
-0.8	-1.06
-0.07	2.52
-0.85	11.25
-0.6	-0.99
0.74	-1.54
1.9	-0.81
0.86	-1.61
0.35	-0.5
1.89	-0.61
1.9	-0.57
-2.08	-1.34
0.15	-1.13
-1.3	-1.08
0.69	-0.21
-0.44	-0.36
-1.66	-0.86
0.65	-1.89
0.7	-0.82

-0.18	-1.12
0.76	-0.9
1.12	1.46
-1.56	-1.61
0.36	-1.1
0.25	-0.75
-1.27	-1.08
-1.44	-1.51
-0.96	-0.59
-0.05	-1.7
-1.06	-3.54
0.25	0.51
-1.15	-1.83
-0.68	-1.32
-0.5	0.12
-0.86	-1.57
0.32	-1.15
-0.39	-1.83
0.62	-1.18
-0.76	0.21
0.33	-1.57
0.08	-1.45
0.71	-1.44
-0.37	-1
-0.55	-1.15
-0.29	0.13
0.24	-1.71
1.05	-1.07
0.13	-1.27
-0.02	-1.45
-0.48	-1.26
-0.1	-2.33
-0.25	-0.85
-0.54	-1.47
3.35	-1.3

-0.07	0.28
-1	0.06
-0.26	-0.52
-0.92	-1.46
0.57	-0.58
-0.26	-1.13
-0.68	-1.38
0.24	-1.16

Log2 Fold Change: (CM_neg_newanalysis_12022024) / (WATER_NEG_NEWANALYSIS_12022024)
-0.62
-0.59
0.28
-1.57
-1.03
-0.37
3.98
3.15
3.34
3.4
3.33
-1.94
-3.04
-2.87
-4.81
2.03
-1.61
0.13
-0.3
-0.56
-0.48
-1.88
-0.58
-1.84
0.24
2.11
3.22
2.06
-1.67
0.17
2.25
1.27
1.31

	-1.36
	-1.52
	-1.09
	-1.12
	-2.08
	-1.03
	-0.89
	-1.77
	-1.22
	-0.51
	-1.67
	-1.93
	0.42
	-1.13
	-1.78
	-2.08
	1.97
	-2.07
	-0.49
	-0.3
	-0.3
	0.81
	-0.45
	0.83
	-0.51
	2.9
	0.59
	3.71
	0.65
	-1.82
	-1.36
	2.21
	1.52
	-1.98
	1.07

	-2.31
	0.17
	0.04
	1.04
	-1.8
	-0.86
	-0.67
	0.15
	-1.94
	-0.68
	-1.7
	1.64
	2.45
	0.07
	-0.44
	-0.81
	-0.91
	0.05
	-1.86
	-1.73
	3.19
	1.86
	-1.76
	-1.41
	-1.98
	-1.97
	-1.85
	-2.05
	-0.82
	-0.28
	2.64
	-0.01
	-0.8
	-1.06
	-1.79

-1.49
4.74
2.58
-1.01
-1.97
3.71
-0.4
2.18
5.21
4.14
-0.46
-1.82
-0.95
-1.97
-1.93
1.11
-1.84
-1.65
-2.02
-0.97
-1.97
-1.25
0.15
-1.05
-0.77
0.15
0.15
1.23
0.78
2.55
1.47
2.9
1.35
1.91
-0.42

	-0.06
	-1.29
	0.18
	-0.61
	0.61
	1.88
	-1.73
	-1.11
	0.84
	0.23
	5.02
	-0.14
	-0.04
	-0.95
	2.46
	3.29
	0.32
	2.06
	-1.21
	1.06
	-1.65
	-0.39
	-0.3
	1.3
	1.31
	0.08
	2.01
	-0.92
	-1.97
	1.98
	4.35
	3.53
	4.79
	0.27
	2.44

3.31
3.02
1.13
-0.37
-0.01
0.9
0.93
-1.82

Log2 Fold Change: (RS_NEG_NEWANALYSIS_12022024) / (WATER_NEG_NE\
-3.38
-4.02
-1.96
-2.19
-2.44
-0.62
3.61
3.32
3.24
3.38
2.08
-0.1
-3.62
-3.8
-5.62
4.13
0.15
2.43
2.51
-2.3
-0.92
-2.8
-1.67
-2.66
0
1.72
2.91
1.52
-2.75
-0.49
1.37
0.5
4.85

	0.32
	-0.17
	-2.53
	-0.19
	-3.05
	-2.66
	-3.11
	-2.33
	-2.43
	-2.63
	-2.69
	-2.62
	4.62
	-0.38
	-1.8
	-1.6
	3.66
	-2.59
	-0.8
	1.16
	3.65
	2.69
	0.15
	1.58
	-4.72
	4.41
	6.44
	1.79
	3.05
	-2.3
	-0.91
	3.25
	3.82
	2.75
	-1.72

	1.32
	0.92
	4.59
	1.59
	-0.17
	-1.38
	0.96
	2.01
	-1.73
	0.84
	-0.18
	3.65
	4.03
	2.91
	3.12
	-0.09
	0.7
	1.29
	-2.9
	0.02
	4.17
	3.92
	-0.45
	-2.99
	-2.93
	-0.85
	-2.55
	-2.65
	-0.91
	1.18
	4.99
	-0.24
	-1.84
	-1.89
	-2.69

	0.4
	3.13
	-0.7
	-1.83
	-0.07
	6.34
	5.72
	6.12
	6.72
	6.58
	5.88
	3.31
	3.5
	-2.8
	-2.54
	-2.37
	0.51
	-1.01
	-2.36
	-1.77
	1.69
	-2.73
	-0.23
	-0.47
	-1.94
	-1.59
	-0.71
	5.74
	2.64
	5.56
	4.14
	5.02
	4.06
	4.32
	0.44

	0.84
	-1.95
	2.86
	-0.1
	2.12
	5.39
	1.6
	3.72
	4.59
	4.24
	9.28
	1.64
	3.61
	2.7
	5.2
	6.12
	0.47
	0.65
	-2.58
	4.02
	-0.19
	1.64
	0.94
	3.41
	5.08
	3.3
	4.61
	1.03
	-2.12
	5.87
	8.78
	6.71
	8.06
	4.65
	2.04

	6.77
	8.49
	4.72
	4
	3.66
	4.16
	3.42
	0.89

Log2 Fold Change: (SM_neg_newanalysis_12022024) / (WATER_NEG_NEWANALYSIS_	P-value: (Bb_neg_newanalysis_12022024) / (CM_
-1.81	0.998887562
-1.71	0.997207754
-0.56	0.999929847
-1.9	0.691706019
-1.57	0.510642703
0.21	0.767955458
4.74	5.20695E-14
4.9	3.04322E-05
5.04	4.98923E-06
4.84	8.21376E-12
3.92	2.73576E-10
-1.4	0.992741652
-2.08	0.375109609
-2.17	0.475617325
-2.73	0.831605454
2.13	0.000877359
-1.76	0.999038506
0.07	0.005353313
1.38	2.44273E-09
0.61	0.7392595
0.34	0.831348992
-1.84	0.968518487
-0.18	0.999933604
-1.63	0.556453232
1.84	0.227919622
3.69	2.73407E-06
4.35	7.16996E-08
3.74	7.08531E-05
-2.04	0.947194982
1.7	0.323812154
3.14	0.009963098
2.7	0.000943023
1.59	5.14729E-06

-1.61	1.59573E-10
-1.75	2.5796E-05
-1.49	1.13191E-07
-1.85	1.13446E-09
-2.16	5.39934E-09
-1.27	6.25056E-14
-2.27	1.19348E-10
-1.86	0.004174598
-1.45	4.85167E-14
-0.12	5.69544E-14
-1.92	3.68279E-10
-2.06	1.35299E-10
1.72	0.326979209
-1.98	0.997355663
-1.68	0.946705208
-1.76	0.997161756
2.13	3.16355E-08
-1.95	0.445698447
-1.68	0.999999509
-1.72	0.920473305
1.03	0.961663234
1.17	0.623966979
-0.71	0.002489766
1.11	8.4713E-07
-2.01	0.917265312
3.04	0.000180934
0.04	0.121888986
0.57	1.18638E-08
1.54	0.000170391
-2.06	0.000545196
-1.86	0.999991493
2.6	8.51129E-11
1.05	0.341149057
-1.94	0.895340855
-1.49	0.0029226

	-1.8	0.000320641
	-1.15	0.946999704
	3.68	0.057979746
	-0.83	0.711401902
	-1.74	0.996716775
	-1.78	0.999878796
	-0.45	0.341264537
	0.69	0.097439819
	-1.75	0.818724359
	-1.21	0.999999293
	-1.95	0.998750662
	2.19	0.009383766
	2.71	1.04229E-06
	1.13	0.001117396
	-0.08	1
	-1.9	0.84340626
	-1.86	0.527507991
	1.16	0.026000951
	-2.1	0.520688518
	-1.69	1.48643E-05
	3.23	2.30249E-12
	2.63	0.003310303
	-1.84	0.966538245
	-2.04	0.991905325
	-1.95	0.999966273
	-1.93	0.961245578
	-1.82	0.40046124
	-1.87	0.957663852
	-1.56	0.940578451
	-1.06	0.596793629
	3.38	0.451875905
	-1.9	0.999982392
	-1.77	0.999512118
	-1.74	0.995697441
	-2.02	0.981697833

	-1.16	0.999722182
	-1.23	0.999996065
	3.96	0.075957875
	-1.21	0.999825949
	-1.68	0.998209024
	4.58	3.18655E-11
	1.47	0.017433527
	3.25	2.74682E-07
	6.07	4.99419E-10
	4.85	1.46764E-05
	0.45	0.089361049
	-1.8	0.99999277
	-1.48	0.961490295
	-1.87	2.49856E-12
	-1.94	4.60743E-14
	-1.49	4.69624E-14
	-2.09	0.768978039
	-0.85	0.999952085
	-1.95	1.78374E-10
	-0.12	4.60743E-14
	-1.37	0.999700727
	-1.99	0.988337559
	-1.75	0.986381699
	-1.91	0.990329985
	-1.12	0.74721748
	-1.74	0.994208262
	-1.75	0.994422861
	3.31	1.44308E-06
	0.63	0.000546281
	3.85	9.3584E-10
	0.77	0.025548659
	3.34	6.96178E-06
	3.01	9.65226E-05
	1.26	5.46892E-07
	-1.12	0.952202916

	0.13	0.098813529
	-2.05	0.22406121
	-0.94	0.282886195
	0.95	0.739411481
	0.25	0.000522498
	1.63	0.000165152
	-0.46	0.989201012
	0.32	0.708841357
	1.8	3.8228E-05
	0.28	0.007726467
	6.08	4.65183E-14
	-0.39	0.999962408
	1.11	0.004883342
	-0.27	0.944601223
	2.96	2.66814E-06
	4.15	1.72318E-12
	0	0.026857003
	2.44	9.99451E-09
	-1.83	0.999999458
	1.82	0.175825086
	-1.98	0.999927445
	-0.47	0.038912472
	-1.01	0.731532807
	1.67	9.80003E-05
	1.87	3.50434E-06
	0.37	0.998999636
	1.77	4.26398E-11
	-1.97	0.995410569
	-2.1	0.922513506
	1.99	2.08161E-08
	4.83	3.55252E-11
	3.63	1.47027E-12
	5.04	5.02931E-14
	0.81	0.027078951
	-0.91	0.008434152

3.38	7.4573E-05
4.02	0.000909207
1.39	0.000335983
0.55	0.469399523
-0.58	0.928580997
1.17	0.001959158
1.61	0.000289853
-2.06	0.992221451

P-value: (Bb_neg_newanalysis_12022024) / (RS	P-value: (CM_neg_newanalysis_12022024) / (RS_NEG_NEWANALYS
0.000706636	0.000248991
6.9329E-06	1.98688E-06
2.13912E-05	1.18163E-05
0.421765486	0.023740456
0.072226931	0.000865663
0.964514708	0.994817208
7.89369E-14	0.923860827
5.15053E-05	0.999962202
1.62574E-05	0.997949172
1.55898E-10	0.746777337
6.98921E-07	0.037395632
0.304107594	0.630694968
0.319916943	0.999997931
0.031003794	0.703804924
0.208782356	0.860825572
1.91956E-06	0.258077779
0.029321762	0.011905026
1.52067E-10	2.84235E-06
0.000465299	0.000941805
0.762593733	0.101673468
0.999993838	0.888323633
0.000168095	0.001444941
0.223749582	0.318378792
0.000332181	0.027493259
0.453105118	0.997509098
1.36151E-06	0.999828248
3.26317E-08	0.999583661
0.000112789	0.999979676
0.008254623	0.069093389
0.514754408	0.999367838
0.00745311	0.999997098
0.002297793	0.999435595
1.10023E-13	8.25717E-08

2.00299E-08	0.34750754
0.001035541	0.763912709
5.94412E-10	0.296003382
8.31403E-09	0.959996621
3.17002E-12	0.0242915
4.65183E-14	0.28185812
9.08051E-13	0.200513106
0.00013721	0.808181529
4.60743E-14	0.006245558
4.60743E-14	0.01106061
3.22509E-12	0.265151103
8.42326E-13	0.167183714
9.65821E-09	2.10362E-06
0.034832446	0.095532572
0.705547682	0.993508948
0.999801308	0.999957083
9.35918E-14	9.00896E-06
0.385766875	0.99999802
0.972888904	0.958601374
0.000124957	0.001854014
8.73797E-11	5.57701E-10
2.71133E-05	0.002055552
0.463831267	0.185249605
7.16691E-10	0.081279077
8.32339E-06	5.68821E-07
1.22868E-08	0.013341167
1.70762E-09	1.35153E-06
3.42266E-05	0.054049397
3.57525E-12	7.47917E-07
1.84793E-06	0.333417804
0.983397534	0.962376842
1.25566E-13	0.017481196
0.022045548	9.24598E-05
0.023682062	0.001547404
1.88151E-07	0.015257985

0.99967474	0.000719911
0.289479649	0.800672274
3.59714E-08	0.000107202
0.951901552	0.992822611
0.067002924	0.022293447
0.971084989	0.912118267
0.105022291	0.000625016
4.06716E-06	0.007249773
0.962748861	0.998228534
0.001848524	0.002325038
0.27412206	0.135681508
1.83964E-08	0.000416215
2.03385E-10	0.019108434
2.81564E-12	9.07552E-08
7.84669E-10	7.7689E-10
0.975993871	0.414368699
0.724708435	0.041088071
2.27981E-06	0.018597423
0.000141035	0.014861373
0.000854629	0.689037054
4.78506E-14	0.005500061
1.21169E-08	0.000786972
0.111623533	0.018419975
0.000341376	0.001652835
0.028622663	0.018259043
0.995957826	0.999461681
9.88922E-05	0.017880258
0.005496312	0.043803624
0.998716181	0.994871616
1.00188E-05	0.000869539
0.200932583	0.002658071
0.992488988	0.998470694
0.881947574	0.72211388
0.477490054	0.7810304
0.001473517	0.009135895

0.154022834	0.086636329
0.705623435	0.774301604
0.88946866	0.491021471
0.167285342	0.264989798
0.115715313	0.046924964
4.62963E-14	1.25793E-06
4.67404E-14	3.62044E-13
4.67404E-14	1.82073E-09
1.91958E-13	0.00551079
6.77092E-12	1.96219E-05
4.65183E-14	8.49321E-14
3.51708E-06	2.43038E-06
0.000791858	8.19254E-05
5.87308E-14	0.080849736
4.60743E-14	0.247182059
4.60743E-14	8.20069E-05
0.024058587	0.000752021
0.928656322	0.855004806
5.05185E-12	0.565726265
4.60743E-14	0.975202587
1.79119E-05	8.08494E-06
0.007454847	0.001422779
0.991433509	0.819409433
0.073878162	0.238731674
0.010407404	0.215110371
0.229707902	0.080685918
0.968580255	0.775829335
4.62963E-14	5.43802E-11
3.61674E-08	0.014412251
4.60743E-14	3.14783E-09
8.79794E-09	5.96982E-05
4.47226E-11	0.000495231
2.26716E-10	0.000270707
8.76743E-13	3.10938E-05
0.981241366	0.631231664

0.000216453	0.187997644
0.012407228	0.772992448
0.657289827	0.010658873
0.004395507	0.117222998
2.98937E-10	6.98505E-05
2.34146E-13	1.53909E-08
1.05474E-06	2.07346E-07
1.12721E-12	2.0013E-11
5.20695E-14	4.87218E-10
7.01939E-12	4.50553E-08
4.60743E-14	4.91737E-09
0.774090568	0.663604306
1.65412E-12	1.13099E-08
3.88393E-06	4.58609E-05
7.4718E-14	4.83882E-08
4.60743E-14	5.72357E-08
0.002440149	0.933495559
1.46182E-05	0.09083046
0.019416048	0.015902958
4.24201E-07	0.000337056
0.920837839	0.835249366
7.6431E-09	3.10978E-05
0.000692851	0.026270503
2.80443E-11	1.92213E-05
4.60743E-14	5.20861E-12
1.64219E-07	4.34158E-07
4.60743E-14	2.76645E-08
0.233934038	0.499515657
0.356590688	0.89935117
4.60743E-14	6.11433E-12
4.60743E-14	9.95598E-11
4.60743E-14	2.95399E-07
4.60743E-14	1.12546E-09
4.9627E-14	1.37212E-12
0.001741854	0.990445147

9.77984E-12	6.37551E-06
1.50879E-13	1.59203E-09
6.42819E-14	3.7479E-10
2.20379E-13	6.11255E-12
6.25343E-09	6.83806E-08
1.95921E-12	3.33329E-08
8.77109E-12	1.46068E-06
7.03557E-05	1.44585E-05

P-value: (SM_neg_newanalysis_12022024) / (RS_NEG_NEWANALYS	P-value: (Bb_neg_newanalysis_12022024) / (SM_neg_nev
0.019503181	0.802748177
0.000102616	0.922706473
0.002416389	0.532891428
0.623563198	0.999425968
0.029668344	0.9987094
0.763637036	0.295349088
0.028630613	4.62963E-14
0.055744794	1.77125E-08
0.129869386	1.69664E-08
0.012798897	1.50657E-13
0.002266859	2.39307E-11
0.961312008	0.7826884
0.631921493	0.994379358
0.331959252	0.841489661
0.014029622	0.819214743
0.267883788	0.000825405
0.000269918	0.493388192
8.04556E-06	0.00194652
0.371797932	1.92621E-06
0.001808263	0.052563374
0.0235568	0.017067923
0.03953496	0.32567323
0.008141009	0.677477387
0.035013584	0.493374431
0.019174666	0.000137777
0.00185842	3.48316E-11
0.189917372	1.09834E-10
0.002723801	1.83894E-09
0.664463131	0.234420049
0.00283641	2.32819E-05
0.072483033	2.86805E-06
0.003184981	3.14028E-08
3.94531E-07	1.0148E-06

0.061333346	2.15251E-11
0.827313829	3.61653E-05
0.577110691	3.19976E-08
0.599156227	1.87416E-10
0.246718562	3.98232E-10
0.482125355	5.4512E-14
0.426244182	4.21035E-11
0.992910143	0.000644707
0.119669242	4.65183E-14
0.0002036	2.00617E-13
0.676889543	7.28254E-11
0.68313086	1.57091E-11
0.000610427	0.003318262
0.002047957	0.872560377
0.999921311	0.569916505
0.9999439	0.996832024
0.000433181	1.04525E-09
0.998895689	0.20962582
0.153047672	0.029699125
1.9041E-05	0.983081887
6.04605E-07	0.012382411
0.045985331	0.085094957
0.210027428	0.002995125
0.430620408	7.24816E-08
0.002835076	0.305955046
0.221349967	5.21022E-06
3.00108E-07	0.33368216
0.907660097	0.000577995
0.003254619	4.47045E-08
0.999303502	4.69902E-06
0.167492729	0.488055281
0.247788957	6.09435E-12
6.94795E-05	0.290383756
0.000544227	0.705223813
0.915403367	2.68604E-06

0.007377096	0.003418126
0.019395546	0.787921944
0.826379999	8.54429E-07
0.02555875	0.002800715
0.001258408	0.617558699
0.949989708	0.999997817
0.005767702	0.82346189
0.111620032	0.006111123
0.867874939	0.400713807
0.000112393	0.908268467
0.002294695	0.322860734
0.02556321	0.000136206
0.285524628	3.60542E-08
0.000102275	8.85886E-07
2.293E-08	0.730875528
0.004254926	0.027246755
0.003618831	0.106256423
0.978595239	1.61687E-05
0.323712618	0.034419197
0.325204937	2.75701E-06
0.047168066	4.62186E-13
0.024852839	8.83177E-05
0.000451039	0.269687728
0.15826886	0.164588499
0.450876927	0.708431296
0.876709885	0.606288718
0.283889604	0.030933774
0.253940008	0.537668851
0.989725263	0.999926008
3.45028E-05	0.997513624
0.075436175	0.996124128
0.005898802	0.02456638
0.999997754	0.920286181
0.985759546	0.854836231
0.322144604	0.207128066

0.117795224	0.999992755
0.342433361	0.017646634
0.060594866	0.004371446
0.553407638	0.968570378
0.028734453	0.988031123
0.000595116	3.96683E-13
8.70292E-11	1.00594E-05
8.26187E-07	6.85745E-10
0.901512008	1.08813E-12
0.079953857	3.80091E-09
8.75855E-13	0.001464996
4.27405E-07	0.968176756
1.55589E-05	0.715504012
0.385279612	5.0393E-13
0.544922574	4.60743E-14
0.270910044	4.60743E-14
2.34888E-06	0.020676092
0.994965849	0.997837094
0.980202484	2.17718E-11
0.166137169	4.60743E-14
8.51052E-06	0.99978434
0.079897271	0.917010829
0.976144983	0.773996143
0.000927047	0.51967107
0.174444423	0.809116413
0.9399216	0.740795711
0.976197201	0.658122871
1.74431E-05	7.84295E-12
0.000594599	0.013342022
0.002528238	7.4496E-14
1.5696E-06	0.363672445
0.039451048	7.7809E-08
0.63359557	8.93052E-09
2.76238E-06	5.90566E-06
0.514002825	0.896177772

0.491422463	0.024409516
0.999980229	0.018777018
8.12349E-05	0.005095262
0.999890538	0.002351989
2.90167E-05	0.001242838
1.39938E-09	0.00247083
0.000182605	0.436465387
2.28539E-08	0.001337621
3.26078E-08	3.39038E-07
8.91642E-08	0.003852231
5.08268E-06	4.60743E-14
0.015514575	0.260251621
9.26219E-06	4.41899E-06
0.000181874	0.731598283
1.74088E-05	8.71377E-09
9.41719E-05	5.67324E-14
0.324843049	0.282782363
0.005503572	6.08485E-10
0.636177823	0.426498011
0.00237799	0.038727054
0.353607005	0.899251861
1.71095E-05	0.063490851
0.002860931	0.994807699
0.000969585	1.98078E-06
5.66636E-10	1.26407E-08
2.83087E-06	0.890067088
4.0292E-09	2.40348E-10
0.010757496	0.727229173
0.999378433	0.205321959
4.38954E-11	2.11256E-09
2.02221E-08	4.21108E-13
2.16124E-06	3.86469E-13
3.61894E-08	4.65183E-14
1.67431E-10	5.84827E-05
0.002531198	0.999991928

5.25732E-05	9.00726E-06
1.58043E-07	5.52197E-06
5.31237E-09	1.4984E-05
5.16991E-09	0.000503456
6.03867E-09	1
3.75876E-07	0.000149844
0.000436096	9.81601E-07
7.21575E-08	0.141349218

P-value: (CM_neg_newanalysis_12022024) / (SM_neg_r	P-value: (Bb_neg_newanalysis_12022024) / (WAT
0.579910706	0.198986235
0.707838209	0.356601204
0.404499993	0.99999637
0.487602381	3.4953E-05
0.748921698	0.000274471
0.963034864	0.188531745
0.221108812	0.029804971
0.084391303	0.91628519
0.283312199	0.806480168
0.248316942	0.296458941
0.875622586	0.480726819
0.975493052	0.260527052
0.696607376	0.966800219
0.988164118	0.790372209
0.177305454	0.050757319
0.999999999	0.158413236
0.719368102	0.356518926
0.998851532	0.116137228
0.126576092	3.99437E-10
0.579033216	0.5839643
0.229603771	0.701101347
0.785176352	0.110259779
0.545412828	0.952943233
0.99999827	0.679795596
0.055041961	0.500548017
0.000922898	0.399860641
0.103950049	0.086948523
0.004247382	0.430205344
0.73102677	0.542835376
0.0068761	0.661783292
0.056469856	0.999861835
0.007541659	0.380101252
0.990305967	0.356313389

0.93935273	4.07391E-09
0.999995796	0.00073543
0.996045917	1.49689E-07
0.968562308	6.58319E-09
0.876841697	8.85427E-06
0.998995031	1.3356E-13
0.996737506	1.93828E-09
0.981509659	0.138276795
0.806738338	1.39555E-13
0.675901672	6.71685E-14
0.977740946	1.20831E-07
0.917268168	6.44E-08
0.336897563	0.922017566
0.626605426	0.15126356
0.970710782	0.290259018
1	0.184258083
0.727866533	0.522692479
0.996449906	0.999972838
0.024561773	0.99999386
0.568081141	0.582430865
0.084516491	0.999986219
0.818565678	0.992390563
0.999999751	0.005325537
0.933609137	0.042649122
0.042959271	0.762600099
0.79215804	0.34198429
0.992594283	0.851691436
0.004358073	0.642752555
0.048268143	0.19996139
0.530686892	0.130061082
0.575019097	0.250516423
0.814433458	0.154795415
0.999998198	3.76834E-05
0.998824688	0.993776934
0.143922432	2.78033E-05

0.950890379	0.438532235
0.27821825	0.999980351
0.002992717	0.627623599
0.091152274	0.0177895
0.875322438	0.094290769
0.999993461	0.847923862
0.960265782	0.925571024
0.850876485	0.772200838
0.979684008	0.993444562
0.876222797	0.993986131
0.545150026	0.446138649
0.626442762	0.999994839
0.789197554	0.856460412
0.129202782	0.479086598
0.72839933	0.999985664
0.303033524	0.999954432
0.923898899	0.982998617
0.095768322	0.368668471
0.686128299	0.847100782
0.989100843	0.001481099
0.950569091	0.150352469
0.770691409	0.48254028
0.72567856	0.313374521
0.423628652	0.23739587
0.594623597	0.543819655
0.969438908	0.561965275
0.776639404	0.593995072
0.951073379	0.210260729
0.86567168	0.139423596
0.849322071	0.356663674
0.751357508	6.05406E-07
0.016503136	0.999996249
0.782484815	0.995849948
0.985894221	0.961829225
0.568762024	0.13554115

0.999988858	0.708821921
0.023680166	0.161112981
0.846067885	0.974157941
0.994802282	0.158860696
0.999937394	0.548296605
0.251578568	0.390591981
0.094153958	0.322847237
0.182962805	0.461468303
0.068524255	0.921422584
0.037557104	0.998857646
0.568953904	0.327529512
0.986172645	0.530538787
0.990299801	0.999870113
0.953196555	8.37813E-10
0.993399977	4.60743E-14
0.028199186	4.60743E-14
0.319044915	0.858132827
0.987524484	0.673469941
0.926863353	1.42553E-07
0.525079657	4.60743E-14
1	0.66808931
0.595120606	0.115486633
0.386718444	0.882362615
0.211830285	0.048963155
0.999996859	0.9936251
0.415309354	0.925514658
0.341904334	0.916803541
0.000252522	0.28269867
0.832168596	0.068728474
0.000227232	0.286097522
0.769490192	0.999807439
0.551182215	0.787242112
0.016999445	0.287997638
0.948262022	0.065506527
0.999963759	0.993390041

0.988780039	0.112168111
0.855616877	0.998040921
0.479957714	0.400770095
0.071506667	0.442989468
0.999530262	0.041366943
0.918446311	0.970570676
0.15981906	0.515783964
0.050489453	0.295830437
0.518894265	0.274312126
0.999797945	0.008063498
0.110619559	1.9755E-06
0.351614546	0.975345442
0.140800347	0.043272345
0.99595109	0.43609928
0.265644398	0.999971772
0.095175559	0.062444398
0.859970849	0.169471553
0.847873512	0.000908021
0.380868764	0.584065024
0.9785461	0.973156653
0.960086167	0.813688031
0.999928277	0.129897265
0.951107317	0.414087516
0.716949901	0.575060585
0.288511369	0.174308213
0.980729717	0.938441556
0.96897079	0.01994637
0.416782798	0.957456984
0.739189394	0.37670583
0.934222253	0.198016218
0.245871993	0.476610259
0.974486181	0.008315134
0.715195411	0.651355659
0.226657855	0.181209603
0.012014596	0.945571566

0.971559714	0.932508585
0.453153312	0.765861035
0.868651787	0.947273847
0.054222812	0.196174794
0.924286141	0.99662526
0.933871399	0.897315219
0.330331869	0.396620481
0.376386858	0.282023781

P-value: (CM_neg_newanalysis_12022024) / (WATER_NEG_NE	P-value: (RS_NEG_NEWANALYSIS_12022024) / (WATER_NEG_NE
0.37024739	1.03901E-06
0.630421151	3.32516E-08
0.99939645	2.96887E-05
0.001954467	2.03459E-07
0.02781414	1.10658E-07
0.888367008	0.607758363
2.46636E-12	1.79222E-11
1.9893E-06	3.32896E-06
0.000160361	0.000526326
9.14842E-10	2.52248E-08
2.02315E-08	9.69245E-05
0.571508065	0.999998804
0.089754991	0.071552107
0.044345354	0.001119738
0.002481696	0.000108636
0.299810405	0.001834057
0.193858869	0.807041003
0.783608071	9.07337E-08
0.970175157	0.000111635
0.99983626	0.058702982
0.999880918	0.776333045
0.018660641	1.17626E-07
0.888173652	0.037660858
0.038425125	5.54362E-06
0.994667643	0.999999528
0.000577386	0.000283518
0.000135441	5.74411E-05
0.011635205	0.017675914
0.132526693	8.1735E-05
0.992109814	0.999887996
0.018481733	0.013955054
0.12289431	0.228017152
0.001353861	3.00948E-12

0.733379876	0.986337091
0.828871909	0.999995212
0.999997738	0.247713511
0.976243751	0.999998779
0.078423862	1.15336E-05
0.933782145	0.043326484
0.828980248	0.013943402
0.678831324	0.098257629
0.311531331	1.96218E-05
0.99877277	0.004069459
0.198727657	0.000996326
0.136568371	0.000283022
0.878008845	1.14106E-07
0.332650483	0.9826782
0.050517678	0.013564906
0.072865645	0.109848994
3.00903E-06	1.2389E-12
0.553943668	0.489223036
0.999999938	0.946814815
0.985485725	0.010374944
0.984531771	1.23213E-10
0.913869226	0.00013131
0.999708557	0.303504045
0.004248339	1.84733E-06
0.99927585	2.28092E-07
0.039042088	2.52921E-06
0.68761357	2.93377E-08
6.23899E-07	0.00237518
0.076607689	6.03286E-10
0.266519994	0.002318798
0.195679188	0.627471728
3.21353E-08	1.03956E-11
0.009847291	5.05064E-09
0.6061542	0.082212355
0.547538311	0.452545941

0.042064206	0.618941298
0.896088357	0.218465081
0.719514495	2.18759E-06
0.339082446	0.125445892
0.032650822	0.999984118
0.935889733	0.401617476
0.884534296	0.011149218
0.717238027	0.000156015
0.492879904	0.74758734
0.997313431	0.007704095
0.247759753	0.999449134
0.012948206	2.53024E-08
2.41095E-05	2.96866E-09
0.098383983	1.26955E-10
0.999983604	1.13086E-09
0.740841076	0.994121264
0.898595974	0.323225193
0.768397413	0.000561149
0.067750331	5.5323E-06
0.563970509	0.99994849
5.12536E-10	1.94844E-13
2.36281E-05	1.72978E-10
0.069297734	0.992414194
0.07707358	6.69427E-07
0.659300544	0.000329061
0.159235293	0.283570352
0.014670799	1.16276E-06
0.036498078	8.99475E-06
0.575268277	0.281436165
0.998463133	0.002599831
9.53926E-05	1.55936E-09
0.999734128	0.982213917
0.999971067	0.6126808
0.771451906	0.122472107
0.029885559	1.24231E-06

0.533258319	0.886702906
0.201388503	0.89761948
0.311319133	0.9994822
0.094653762	0.000351477
0.310398218	0.926424078
2.72881E-09	4.87388E-14
0.725342724	7.02771E-14
3.97977E-05	6.13953E-14
5.07494E-09	9.73333E-13
4.20569E-05	1.49166E-11
0.980109807	5.71765E-14
0.447988383	0.000407158
0.991856516	0.000405333
0.107226507	7.46922E-05
0.214508611	0.000998975
0.561911821	0.007653239
0.165000108	0.2640655
0.549763236	0.994226287
0.093839725	0.001538551
0.850166001	0.419705699
0.488999785	0.001129108
0.350468881	5.25E-06
0.997648262	0.560539196
0.171004382	0.999966416
0.415351102	0.002488708
0.997961905	0.030545565
0.997127924	0.496756961
0.000564634	5.20695E-14
0.423881079	8.70831E-05
1.98527E-07	4.69624E-14
0.013352235	4.61493E-09
0.000248928	8.21923E-10
0.029669214	4.01814E-08
0.001647149	4.01381E-10
0.718027801	0.999990712

0.999999861	0.16764027
0.098790012	0.004133184
0.999903566	0.018904079
0.996340322	0.285526543
0.550551414	6.90511E-07
0.001373837	8.54317E-13
0.204003098	0.000126695
0.97953537	9.29771E-11
0.014128075	3.30513E-13
1	4.31938E-08
1.06762E-10	4.60743E-14
0.993486646	0.337178856
0.948731311	1.34917E-09
0.920864903	0.000691747
1.64405E-06	6.69464E-14
9.68136E-10	4.62963E-14
0.955106626	0.488126677
0.002338828	0.671932482
0.634532376	0.000252332
0.035449297	5.95987E-08
0.693204875	0.999818535
0.993089066	6.6975E-06
0.99487795	0.085916538
0.008550682	1.14123E-09
0.002902598	4.87388E-14
0.788482295	1.57349E-08
1.53969E-07	4.94049E-14
0.999420978	0.703747193
0.060812553	0.004707089
1.10471E-05	4.62963E-14
2.16105E-09	4.60743E-14
5.87812E-09	4.78506E-14
1.0425E-13	4.60743E-14
0.947647726	2.91211E-13
0.071389583	0.017719819

5.53876E-06	1.49269E-12
2.28297E-05	5.60663E-14
0.003722185	1.34448E-13
0.99294296	1.95831E-11
0.996625795	2.03822E-08
0.028839153	1.65047E-11
0.045756892	6.28697E-10
0.096825419	0.023283173

P-value: (SM_neg_newanalysis_12022024) / (WATER_NEG_
0.012085947
0.056029499
0.606824443
1.40312E-05
0.000803873
0.999819076
7.78266E-14
1.57373E-09
4.29105E-07
6.58795E-12
1.37329E-09
0.93941126
0.769791375
0.165900825
0.477319499
0.289249264
0.007933212
0.555523918
0.022588252
0.734733158
0.339381916
0.000613404
0.98849014
0.030229389
0.015788933
2.89617E-09
8.93165E-08
2.05074E-07
0.004970232
0.001502199
5.62029E-06
5.78272E-06
0.000261159

0.223739397
0.882505382
0.990148871
0.658261293
0.005478112
0.779235832
0.553026642
0.280029087
0.023696918
0.87776844
0.044538328
0.014477416
0.038630725
0.012184141
0.00775287
0.071170784
7.40805E-08
0.283879055
0.021636968
0.220034252
0.01820996
0.253554322
0.999924902
0.000334059
0.018827484
0.001475759
0.943202256
0.031908234
2.51023E-05
0.005754485
0.004356029
1.54427E-09
0.012777345
0.376540515
0.955143787

0.246797116
0.867547305
6.18814E-05
0.978417185
0.001936554
0.892428678
0.999833091
0.134093792
0.160146282
0.631202687
0.005460563
0.000193777
7.20123E-07
0.000124939
0.815402656
0.01686267
0.357871889
0.003886478
0.001725354
0.233435284
7.19229E-11
6.44367E-07
0.002122883
0.000630831
0.04095288
0.029650661
0.000449462
0.00413267
0.089565357
0.623597053
2.26808E-06
0.032649649
0.679036985
0.381340351
0.000386346

0.62585504
0.915422073
0.028775548
0.029205856
0.218424762
1.74741E-11
0.003109077
6.03686E-08
7.47169E-12
9.62385E-09
0.202397686
0.155311165
0.844738132
0.014889722
0.071769524
0.593173447
0.001035942
0.895058724
0.009821914
0.992637391
0.500393481
0.011701817
0.187042059
0.000557895
0.483231446
0.210008234
0.154211653
9.29993E-10
0.981274601
1.6408E-12
0.238372138
2.37294E-06
2.39889E-06
0.01638269
0.603103448

0.98283767
0.006391103
0.349632675
0.190041129
0.743749899
0.018069471
0.999993826
0.214004736
0.00013225
0.999728328
4.81726E-13
0.680530193
0.019861394
0.996404393
5.62188E-09
2.51899E-12
0.999688797
9.4218E-05
0.015798545
0.005978109
0.231961488
0.99932905
0.732416072
0.000186125
7.71099E-06
0.372756346
1.21242E-06
0.250505345
0.001927243
8.69765E-07
1.38359E-11
9.88814E-10
5.09592E-14
0.036338726
0.9732553

7.01898E-07
1.56687E-07
0.000174403
0.172732342
0.996122471
0.002652085
0.00020561
0.00067285

Adj. P-value: (Bb_neg_newanalysis_12022024) / (CM_neg_newanalysis_12022024)
1
1
1
1
1
1
1.40686E-11
0.000674715
0.000130363
8.59889E-10
1.81009E-08
1
1
1
1
0.013387352
1
0.064797216
1.29146E-07
1
1
1
1
1
1
7.56922E-05
2.69864E-06
0.0014378
1
1
0.109299501
0.014320181
0.00013383

	1.15184E-08
	0.000579238
	4.07592E-06
	6.45091E-08
	2.61818E-07
	1.45745E-11
	8.85754E-09
	0.052085271
	1.40686E-11
	1.4291E-11
	2.35698E-08
	9.90187E-09
	1
	1
	1
	1
	1.29558E-06
	1
	1
	1
	1
	1
	0.033354426
	2.60028E-05
	1
	0.003318108
	0.781354863
	5.29782E-07
	0.003154026
	0.008803333
	1
	6.69386E-09
	1
	1
	0.038372476

0.005545615
1
0.452837858
1
1
1
1
0.663362905
1
1
1
0.103805855
3.12649E-05
0.016508214
1
1
1
0.241995776
1
0.000351232
2.79983E-10
0.042648138
1
1
1
1
1
1
1
1
1
1
1
1
1

1
1
0.554859454
1
1
2.87846E-09
0.173544836
9.16072E-06
3.04819E-08
0.000347832
0.624776408
1
1
3.01505E-10
1.40686E-11
1.40686E-11
1
1
1.25484E-08
1.40686E-11
1
1
1
1
1
1
1
4.19343E-05
0.008811852
5.41896E-08
0.238695747
0.000178655
0.00191687
1.73949E-05
1

	0.670981216
	1
	1
	1
	0.008497581
	0.003067825
	1
	1
	0.000831236
	0.088187715
	1.40686E-11
	1
	0.059841765
	1
	7.39964E-05
	2.21463E-10
	0.248278075
	4.54004E-07
	1
	0.974901073
	1
	0.335037231
	1
	0.001943774
	9.43724E-05
	1
	3.62392E-09
	1
	1
	8.82124E-07
	3.10267E-09
	1.96966E-10
	1.40686E-11
	0.250037412
	0.095097771

0.001509411
0.013846576
0.005773065
1
1
0.026884008
0.005068582
1

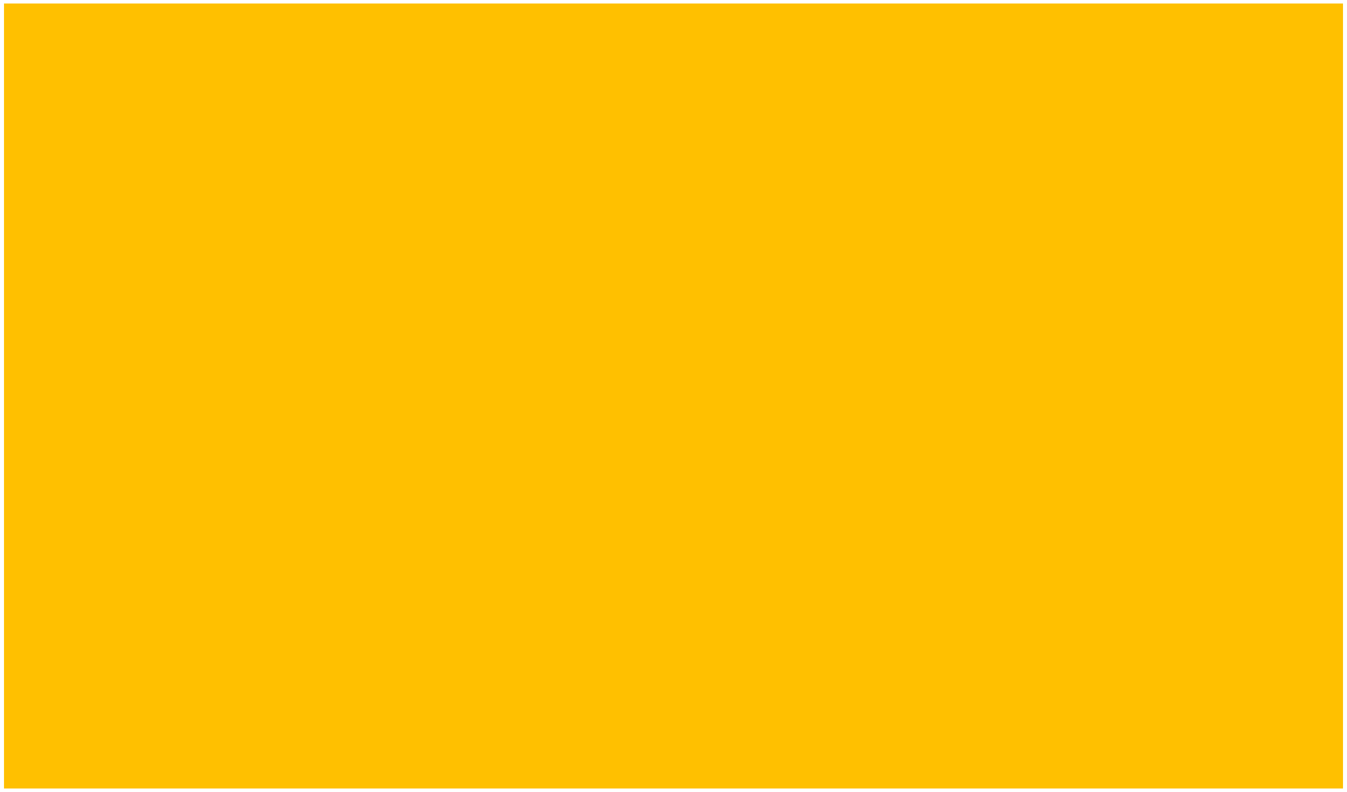

Adj. P-value: (Bb_neg_newanalysis_12022024) / (RS_NEG_I	Adj. P-value: (CM_neg_newanalysis_12022024) / (RS_NEG
0.002803841	0.001930382
5.72E-05	3.3882E-05
0.00014989	0.000152235
0.639853052	0.072296115
0.143545804	0.005287634
1	1
5.0932E-12	1
0.000321436	1
0.000117889	1
3.77401E-09	1
7.72086E-06	0.104259285
0.488252371	0.942613789
0.509392328	1
0.068828525	1
0.356726274	1
1.87542E-05	0.486024961
0.06562628	0.041741493
3.68692E-09	4.53858E-05
0.001986348	0.005658775
1	0.233341199
1	1
0.000869802	0.00797836
0.378054318	0.569787382
0.001513727	0.081236157
0.678348869	1
1.38767E-05	1
4.88024E-07	1
0.000620251	1
0.022105552	0.170314719
0.753036126	1
0.020324093	1
0.007529749	1
6.6894E-12	2.35188E-06

3.11953E-07	0.609564935
0.003835481	1
1.26297E-08	0.539640349
1.3893E-07	1
1.15199E-10	0.073602468
4.24286E-12	0.519120722
3.90067E-11	0.399157685
0.00073253	1
4.24286E-12	0.025071044
4.24286E-12	0.039415265
1.16664E-10	0.496565411
3.67831E-11	0.345153473
1.58708E-07	3.54143E-05
0.075970102	0.221693907
0.954088773	1
1	1
5.87103E-12	0.000121269
0.595236971	1
1	1
0.000675783	0.009762907
2.24967E-09	4.02563E-08
0.000185304	0.010625952
0.69191702	0.374431115
1.48876E-08	0.193911809
6.6756E-05	1.15133E-05
1.98194E-07	0.045589531
3.28795E-08	2.44171E-05
0.000226572	0.139884229
1.27292E-10	1.45964E-05
1.81216E-05	0.590022237
1	1
7.50363E-12	0.05651181
0.051408176	0.000856743
0.054739879	0.008449521
2.36618E-06	0.050702602

1	0.004550323
0.46910244	1
5.33429E-07	0.000965061
1	1
0.134499331	0.068563192
1	1
0.198231926	0.004056347
3.60189E-05	0.028061805
1	1
0.006270488	0.011746311
0.448073779	0.293413582
2.8879E-07	0.00291001
4.82025E-09	0.060680219
1.05723E-10	2.53026E-06
1.61933E-08	5.11711E-08
1	0.696030219
0.971934414	0.112276616
2.17892E-05	0.059355554
0.000749155	0.04975441
0.003278062	0.996579855
4.24286E-12	0.022766421
1.95653E-07	0.00489589
0.20906929	0.058872417
0.001547591	0.008935712
0.064298288	0.058499989
1	1
0.000555342	0.057542979
0.015672023	0.118004036
1	1
7.80951E-05	0.005307211
0.345592674	0.013008914
1	1
1	1
0.708614606	1
0.005159104	0.03371941

0.276429718	0.204501582
0.954109594	1
1	0.790434563
0.296130648	0.496380936
0.215812608	0.124754427
4.24286E-12	2.29624E-05
4.24286E-12	1.5061E-10
4.24286E-12	9.8967E-08
1.0573E-11	0.022781006
2.26289E-10	0.00023322
4.24286E-12	5.11562E-11
3.17159E-05	4.01037E-05
0.003079652	0.000777343
4.30496E-12	0.193238091
4.24286E-12	0.469928321
4.24286E-12	0.000777343
0.055399584	0.004721187
1	1
1.75516E-10	0.876163495
4.24286E-12	1
0.000128588	0.000111136
0.020325322	0.007880621
1	1
0.146385808	0.457106383
0.026913175	0.421789226
0.386504051	0.192914729
1	1
4.24286E-12	7.10448E-09
5.3565E-07	0.048525849
4.24286E-12	1.54537E-07
1.45784E-07	0.000594343
1.21892E-09	0.003352021
5.32529E-09	0.002066317
3.79653E-11	0.000342813
1	0.942881049

0.001070651	0.379016293
0.03140145	1
0.906353593	0.038294422
0.013033985	0.261172607
6.81904E-09	0.000677421
1.25708E-11	5.82057E-07
1.10934E-05	4.91412E-06
4.72651E-11	3.47279E-09
4.24286E-12	3.59904E-08
2.32626E-10	1.41316E-06
4.24286E-12	2.24664E-07
1	0.974840337
6.58649E-11	4.48089E-07
3.459E-05	0.000472908
4.90101E-12	1.49107E-06
4.24286E-12	1.71391E-06
0.007907724	1
0.000107883	0.212749727
0.045940562	0.052473106
4.93797E-06	0.002470179
1	1
1.28946E-07	0.000342813
0.002755642	0.078237398
8.07514E-10	0.000229148
4.24286E-12	1.21085E-09
2.0986E-06	9.16311E-06
4.24286E-12	9.34446E-07
0.391982723	0.801496499
0.55817265	1
4.24286E-12	1.38079E-09
4.24286E-12	1.05627E-08
4.24286E-12	6.58627E-06
4.24286E-12	6.65562E-08
4.24286E-12	4.33811E-10
0.00596517	1

3.09819E-10	9.03083E-05
8.70474E-12	8.83044E-08
4.41419E-12	2.87606E-08
1.19307E-11	1.38079E-09
1.0745E-07	2.0073E-06
7.62837E-11	1.10236E-06
2.83545E-10	2.61499E-05
0.000418586	0.000179263

Adj. P-value: (SM_neg_newanalysis_12022024) / (RS_NEG_NEWAN	Adj. P-value: (Bb_neg_newanalysis_12022024) / (SM_neg_r
0.086809259	1
0.001055402	1
0.015863071	0.896981247
1	1
0.121627898	1
1	0.654546248
0.118324897	9.72174E-12
0.194700331	5.77317E-07
0.356543114	5.57599E-07
0.062499525	2.05955E-11
0.015050192	1.5504E-09
1	1
1	1
0.679152447	1
0.067287701	1
0.587745582	0.0087161
0.002446594	0.86061647
0.000117546	0.017209248
0.732657904	4.04914E-05
0.012476789	0.216950867
0.101274379	0.093586447
0.150594857	0.690988331
0.043127707	1
0.137793118	0.86061647
0.085625175	0.001903821
0.012745297	2.15928E-09
0.461417177	5.86571E-09
0.017453525	7.304E-08
1	0.573462107
0.018057981	0.000388228
0.236591324	5.81259E-05
0.019908334	9.81058E-07
8.68627E-06	2.23425E-05

0.208148889	1.42372E-09
1	0.000571638
0.980226261	9.97669E-07
1	9.3875E-09
0.555573652	1.78843E-08
0.870351558	9.72174E-12
0.805218455	2.5599E-09
1	0.007102113
0.337080261	9.72174E-12
0.001929558	2.51695E-11
1	4.11151E-09
1	1.08916E-09
0.004925796	0.026162141
0.01382918	1
1	0.927067309
1	1
0.003659926	4.29176E-08
1	0.539855587
0.398908095	0.142397259
0.000246318	1
1.25428E-05	0.07290173
0.1688988	0.304064439
0.496354251	0.024122449
0.80990451	2.09086E-06
0.018056763	0.666684921
0.515207136	9.94723E-05
6.80646E-06	0.699953236
1	0.006457209
0.02023958	1.35641E-06
1	9.08095E-05
0.423365057	0.856480671
0.557269574	4.69948E-10
0.000759566	0.648176562
0.004469166	1
1	5.493E-05

0.039761868	0.026762622
0.086440594	1
1	1.91831E-05
0.108077888	0.022875009
0.009213946	0.965901645
1	1
0.032446914	1
0.321987128	0.041639925
1	0.772644093
0.001142579	1
0.015196707	0.688026756
0.108077888	0.001888205
0.61384106	1.111E-06
0.001052582	1.97519E-05
7.2933E-07	1
0.025324502	0.133555566
0.022106824	0.353621377
1	0.000278729
0.668222651	0.1587219
0.67007816	5.61634E-05
0.172320959	4.87082E-11
0.105724886	0.001276167
0.003788532	0.620646907
0.408542478	0.469811302
0.834108201	1
1	0.957081292
0.611740303	0.146759032
0.566726681	0.900775927
1	1
0.000410399	1
0.24436374	1
0.033043323	0.123546784
1	1
1	1
0.666413336	0.536135575

0.334476393	1
0.693602582	0.095894807
0.206484942	0.032111437
0.954321801	1
0.11862999	1
0.004831841	4.35469E-11
7.31786E-09	0.000180089
1.65531E-05	2.93774E-08
1	1.00007E-10
0.254615346	1.41376E-07
1.73069E-10	0.013846179
9.3192E-06	1
0.000208614	1
0.749600013	5.20662E-11
0.944843265	9.72174E-12
0.592648116	9.72174E-12
4.10288E-05	0.108227703
1	1
1	1.42809E-09
0.421075826	9.72174E-12
0.000123313	1
0.254486412	1
1	1
0.007076179	0.884091062
0.43500357	1
1	1
1	0.99689597
0.000229119	5.79352E-10
0.004830125	0.07725666
0.016440307	1.18953E-11
2.88514E-05	0.73508552
0.150311442	2.22424E-06
1	3.0824E-07
4.72084E-05	0.000111007
0.909486978	1

0.881564938	0.122926293
1	0.100755976
0.000869019	0.036200405
1	0.019995579
0.000355854	0.012187832
7.22921E-08	0.02086479
0.001749462	0.810776629
7.28375E-07	0.012932791
9.82309E-07	8.44018E-06
2.36494E-06	0.029201008
7.93943E-05	9.72174E-12
0.072775786	0.608677136
0.000131907	8.59231E-05
0.001744574	1
0.00022895	3.01416E-07
0.000983919	9.8938E-12
0.669768999	0.638985357
0.031182962	2.65716E-08
1	0.799354546
0.015649984	0.173123883
0.708322051	1
0.000225389	0.247146854
0.018170186	1
0.007346941	4.15833E-05
3.38015E-08	4.26974E-07
4.81704E-05	1
1.71681E-07	1.16547E-08
0.054209275	1
1	0.533135599
3.98793E-09	8.30729E-08
6.59116E-07	4.5595E-11
3.8088E-05	4.27224E-11
1.0794E-06	9.72174E-12
1.24847E-08	0.00087713
0.016452785	1

0.000595755	0.000163288
3.92821E-06	0.000104917
2.14745E-07	0.000260867
2.09889E-07	0.005788093
2.38485E-07	1
8.31567E-06	0.002042015
0.003678654	2.17217E-05
1.97006E-06	0.42715512

Adj. P-value: (CM_neg_newanalysis_12022024) / (SM_neg_new	Adj. P-value: (Bb_neg_newanalysis_12022024) / (WATER_NEG_NEV
1	0.840837853
1	1
1	1
1	0.001898751
1	0.012222059
1	0.827988817
1	0.445749271
1	1
1	1
1	0.947517779
1	1
1	0.907731863
1	1
1	1
1	0.589237952
1	0.79266544
1	1
1	0.747822983
1	6.64662E-08
1	1
1	1
1	0.747822983
1	1
1	1
1	1
0.124693722	1
1	0.717954028
0.349701127	1
1	1
0.431338875	1
1	1
0.4450931	1
1	1

1	5.19357E-07
1	0.027358822
1	1.40017E-05
1	8.00516E-07
1	0.000566673
1	5.27828E-11
1	2.6414E-07
1	0.772989589
1	5.3807E-11
1	2.94944E-11
1	1.17011E-05
1	6.6976E-06
1	1
1	0.782873861
1	0.9392865
1	0.824036842
1	1
1	1
0.803813347	1
1	1
1	1
1	1
1	0.130117606
1	0.540222214
1	1
1	0.993766112
1	1
0.349865373	1
1	0.842009346
1	0.762383042
1	0.897224176
1	0.788180989
1	0.002023419
1	1
1	0.001553052

1	1
1	1
0.270336405	1
1	0.31956411
1	0.725642831
1	1
1	1
1	1
1	1
1	1
1	1
1	1
1	1
1	1
1	1
1	1
1	1
1	1
1	1
1	0.04768475
1	0.782260932
1	1
1	0.967237017
1	0.884185576
1	1
1	1
1	1
1	0.852321508
1	0.77316027
1	1
1	4.98451E-05
0.660459677	1
1	1
1	1
1	0.770170684

1	1
0.791408165	0.795568956
1	1
1	0.793181028
1	1
1	1
1	0.976105918
1	1
1	1
1	1
1	0.980279903
1	1
1	1
1	1.24945E-07
1	2.65677E-11
0.857255267	2.65677E-11
1	1
1	1
1	1.35317E-05
1	2.65677E-11
1	1
1	0.747822983
1	1
1	0.579782433
1	1
1	1
1	1
0.049282221	0.93082703
1	0.672629034
0.046052278	0.935393927
1	1
1	1
0.671243329	0.937040814
1	0.657822441
1	1

1	0.747822983
1	1
1	1
1	1
1	0.531791267
1	1
1	1
1	0.945891494
1	0.921869934
1	0.179027766
1	0.000148004
1	1
1	0.546297177
1	1
1	1
1	0.640427919
1	0.806861921
1	0.032475097
1	1
1	1
1	1
1	0.762383042
1	1
1	1
1	0.815170383
1	1
1	0.348026736
1	1
1	1
1	0.839202174
1	1
1	0.183840061
1	1
1	0.819913047
0.552112614	1

1	1
1	1
1	1
1	0.837991924
1	1
1	1
1	1
1	0.929867706

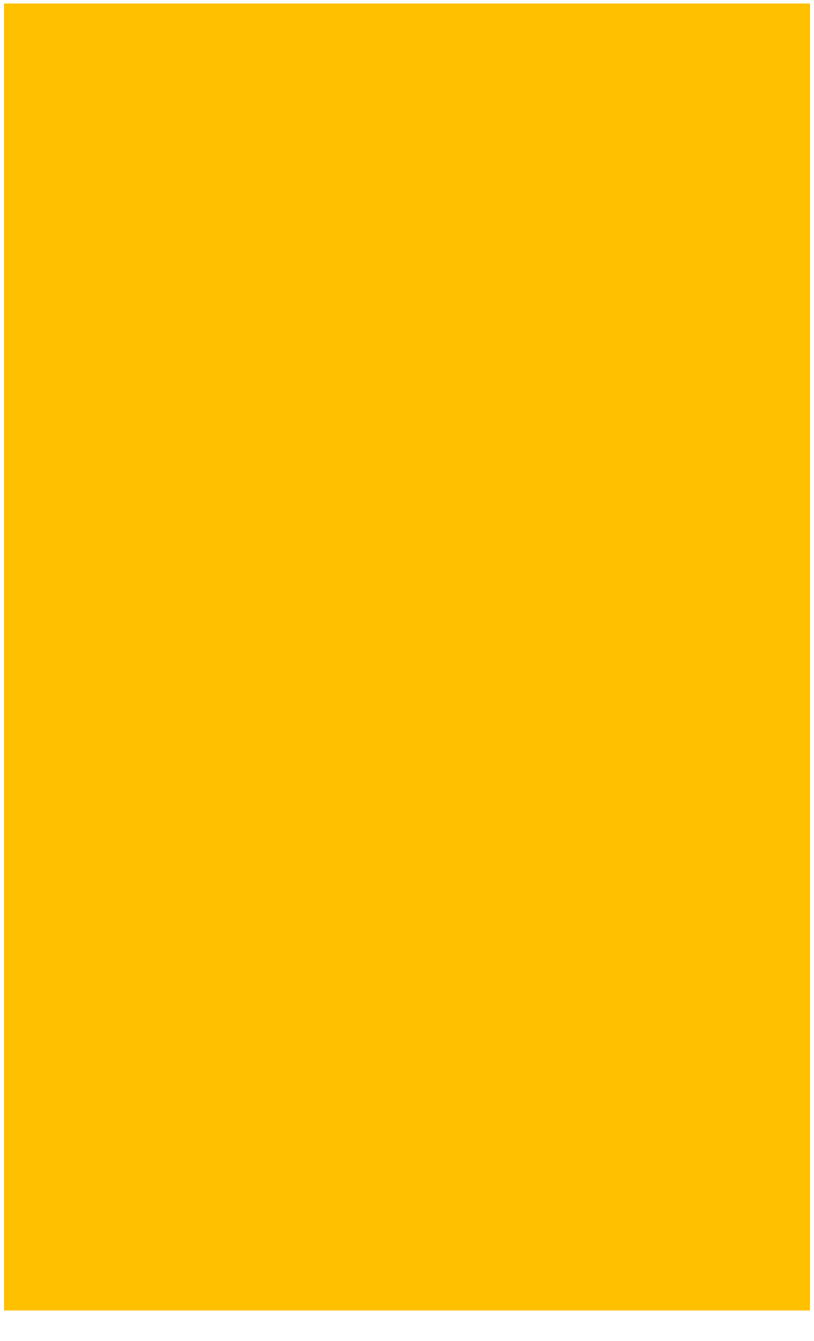

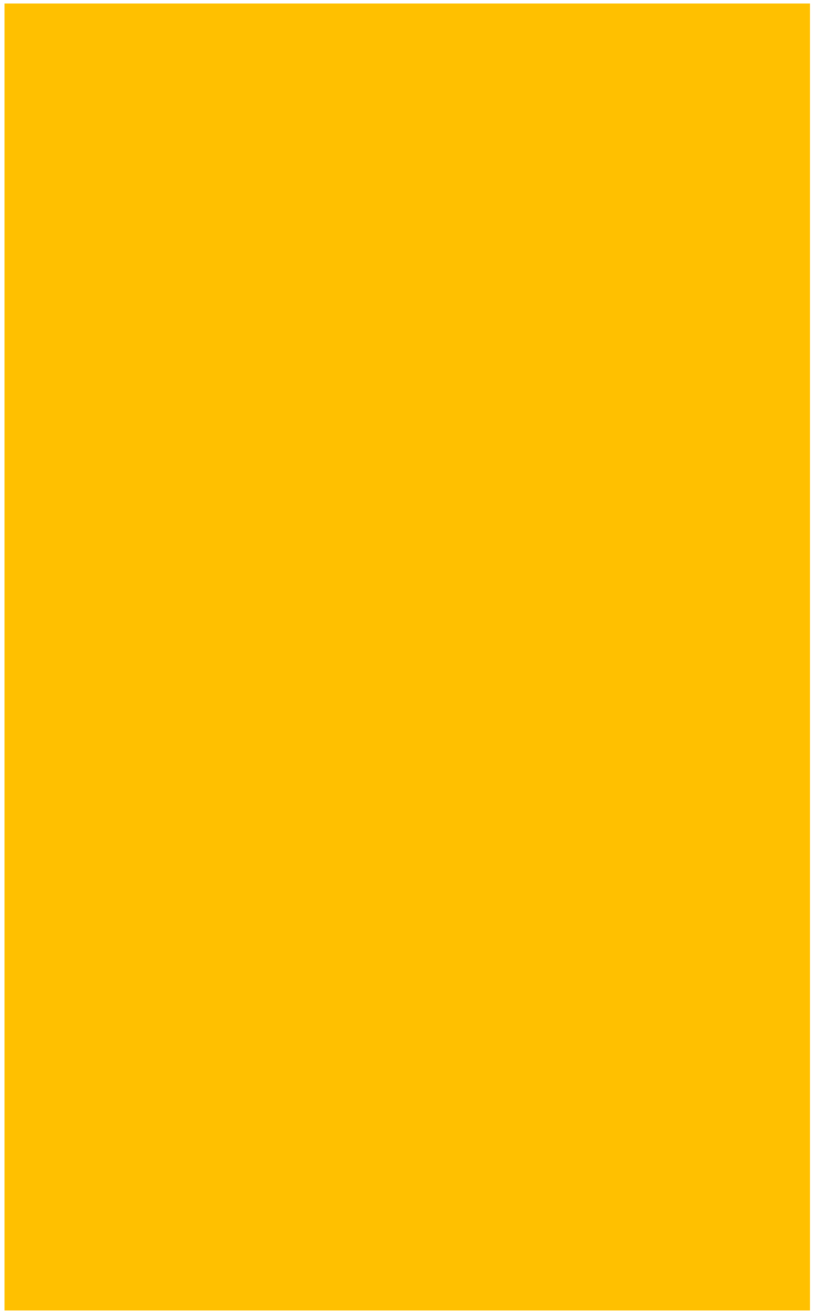

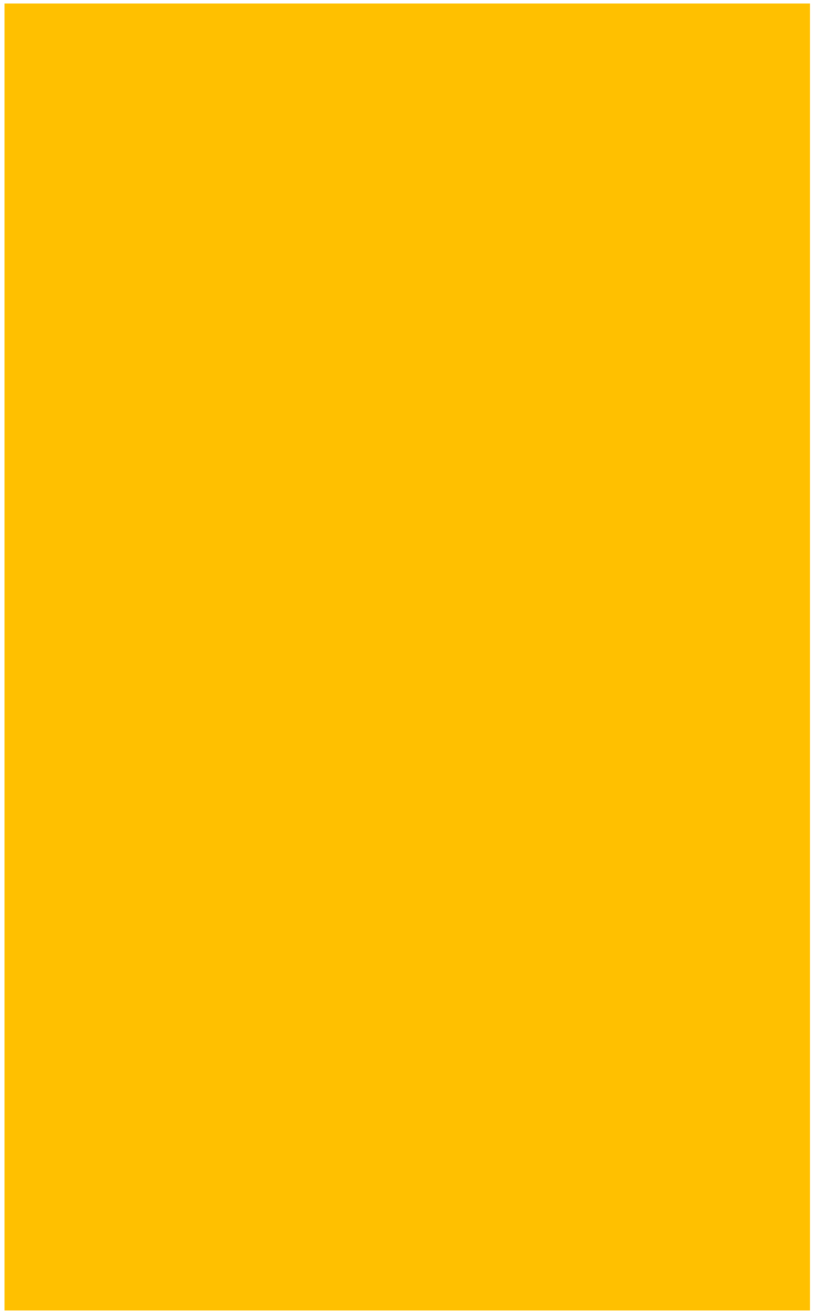

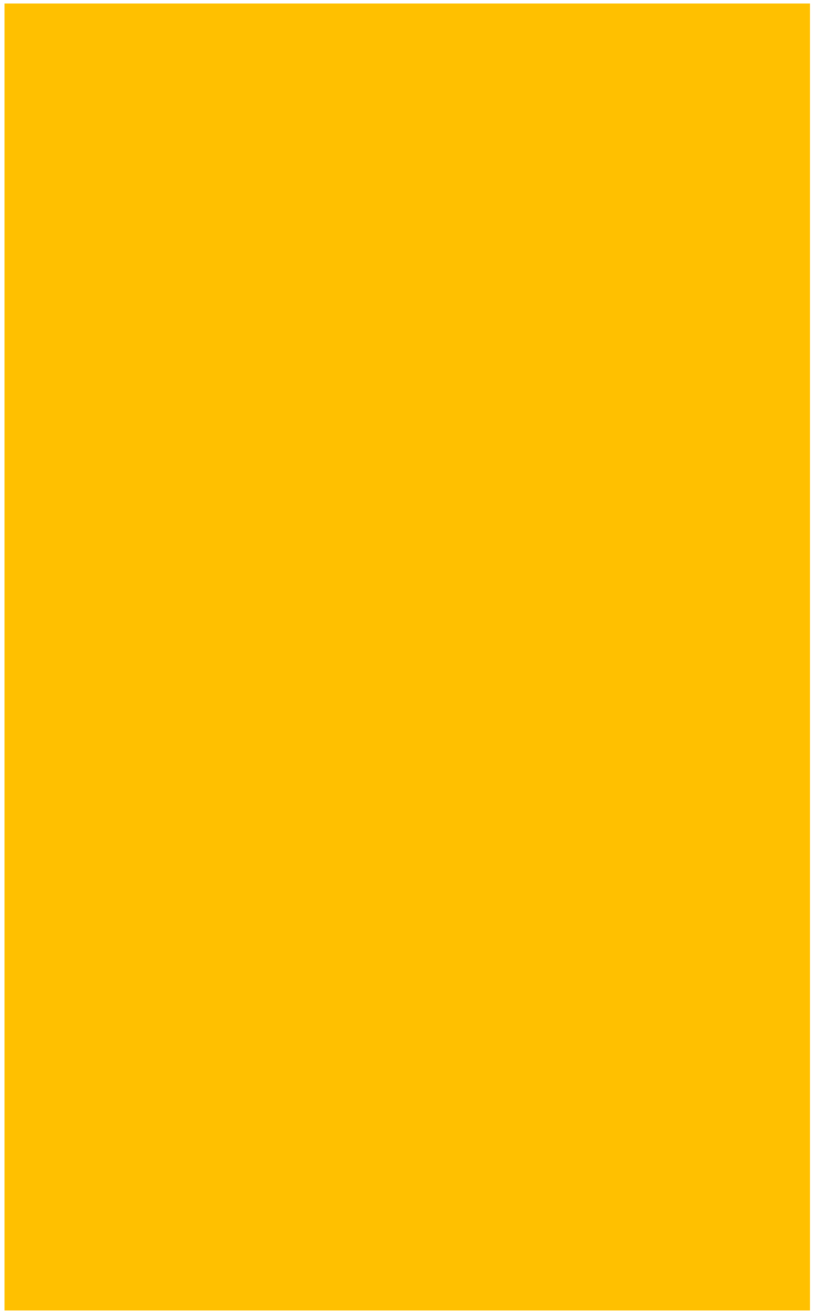

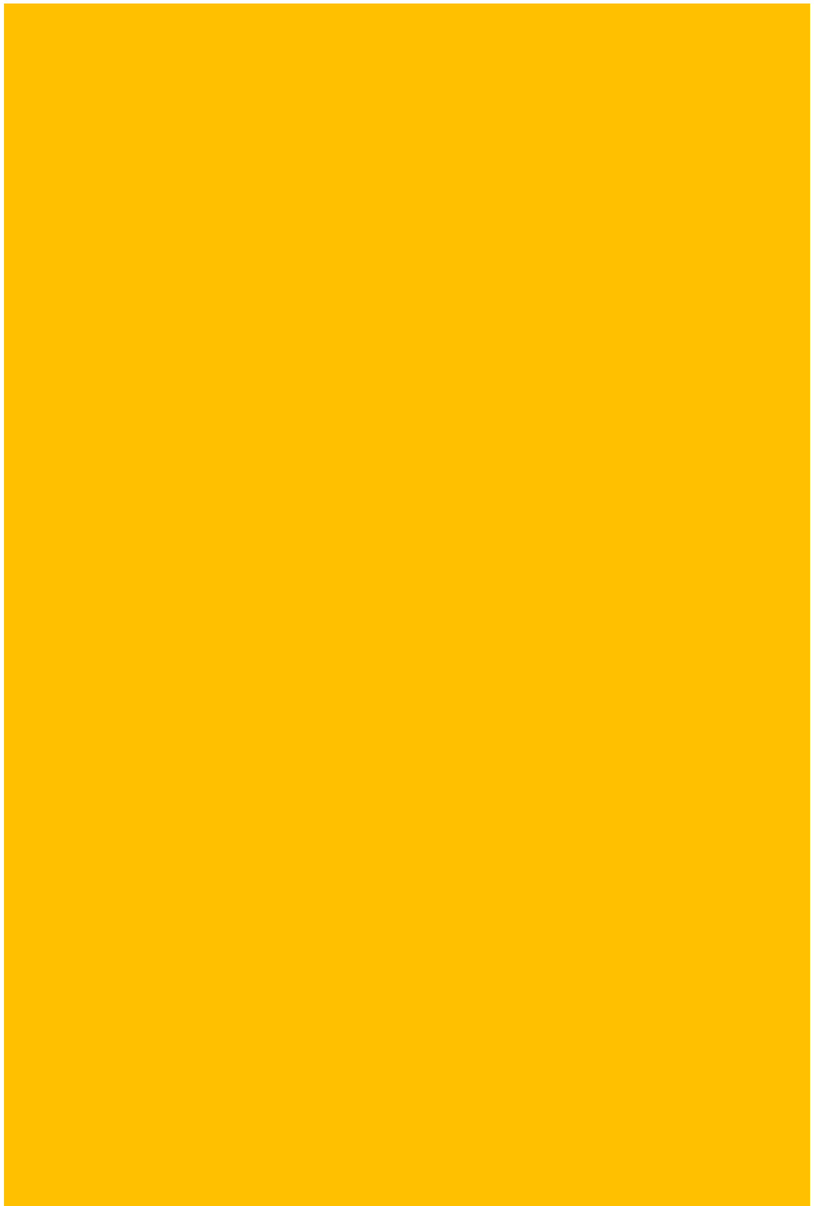

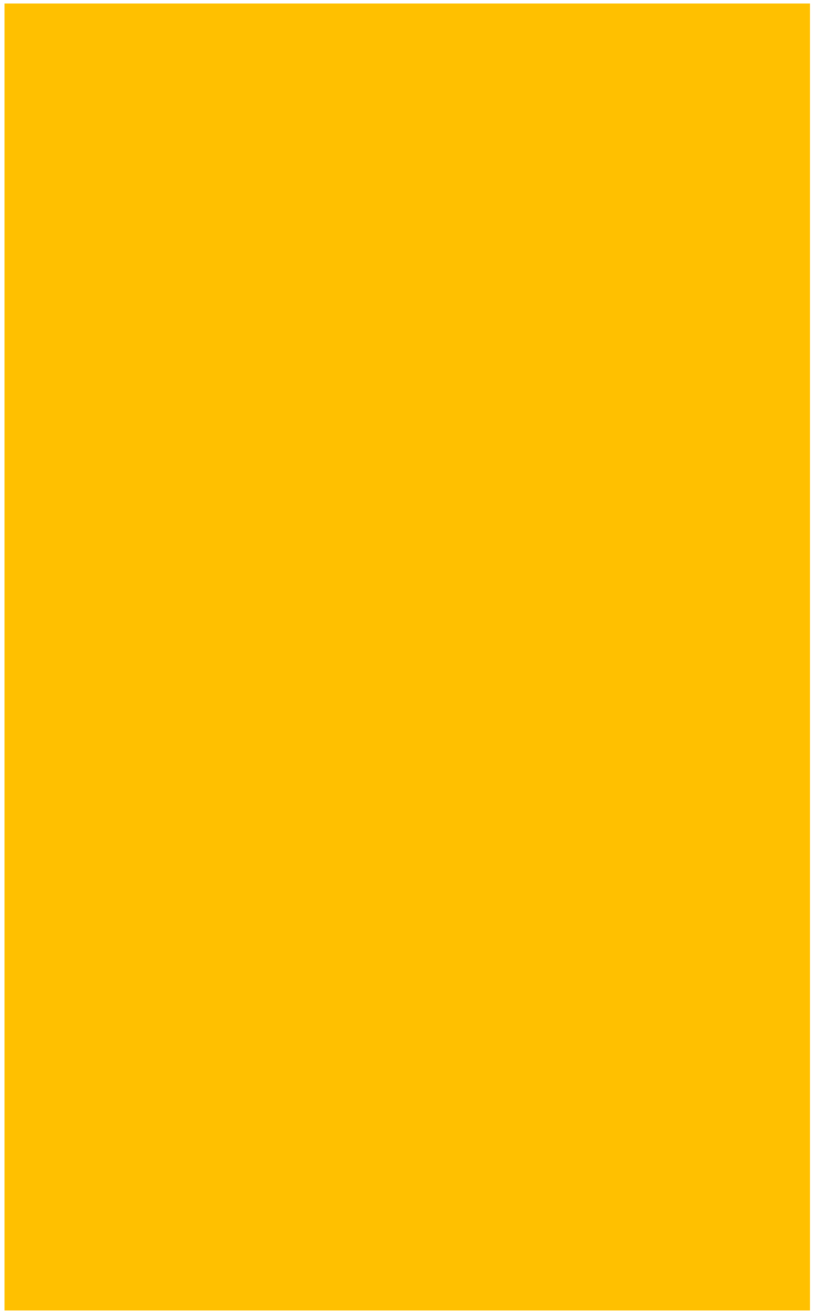

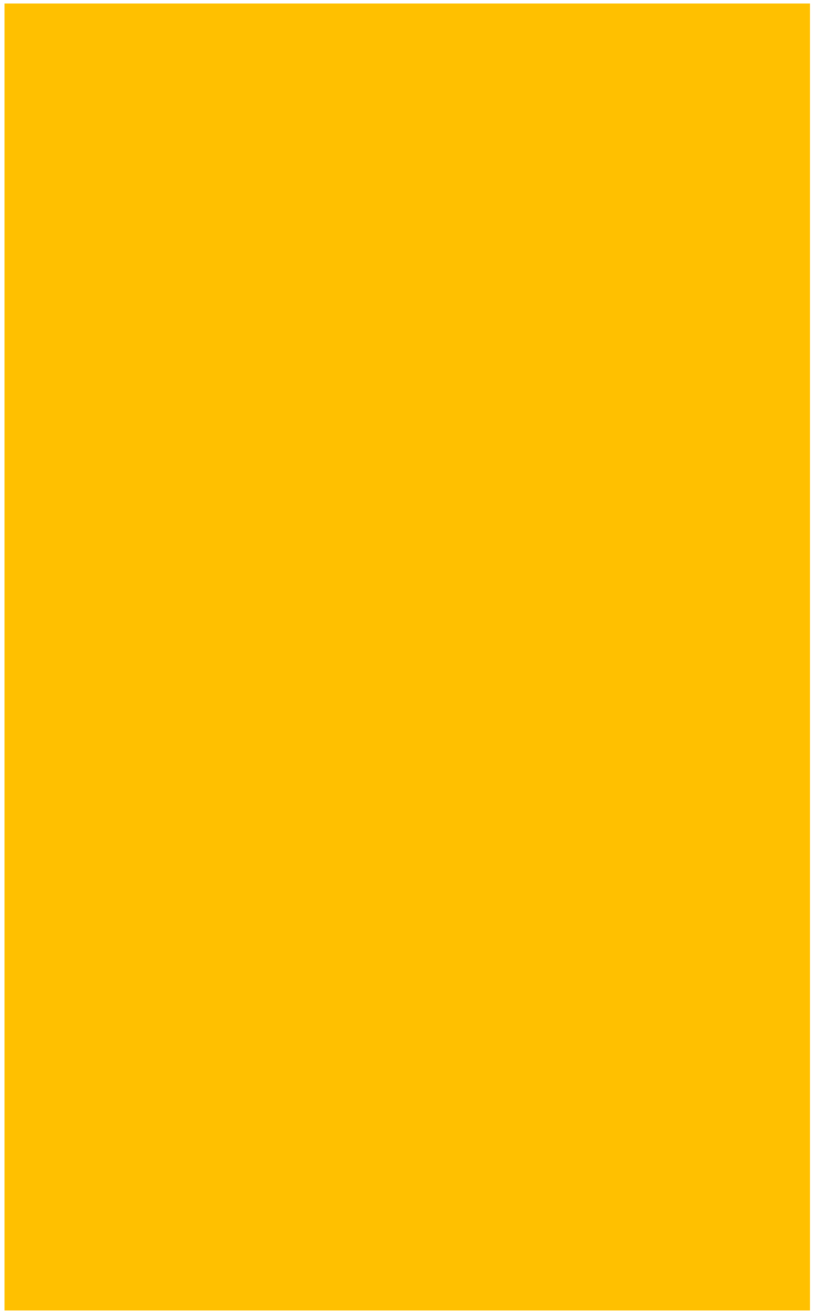

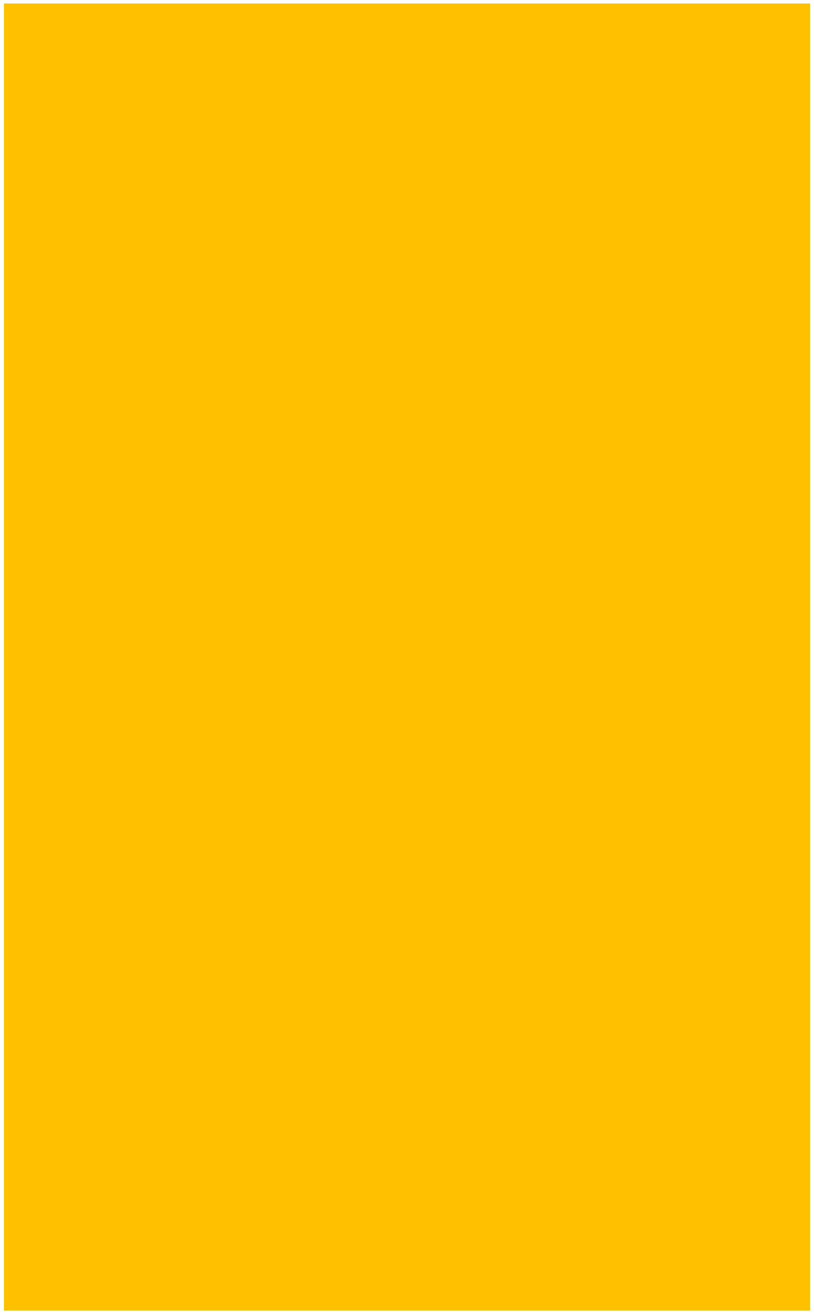

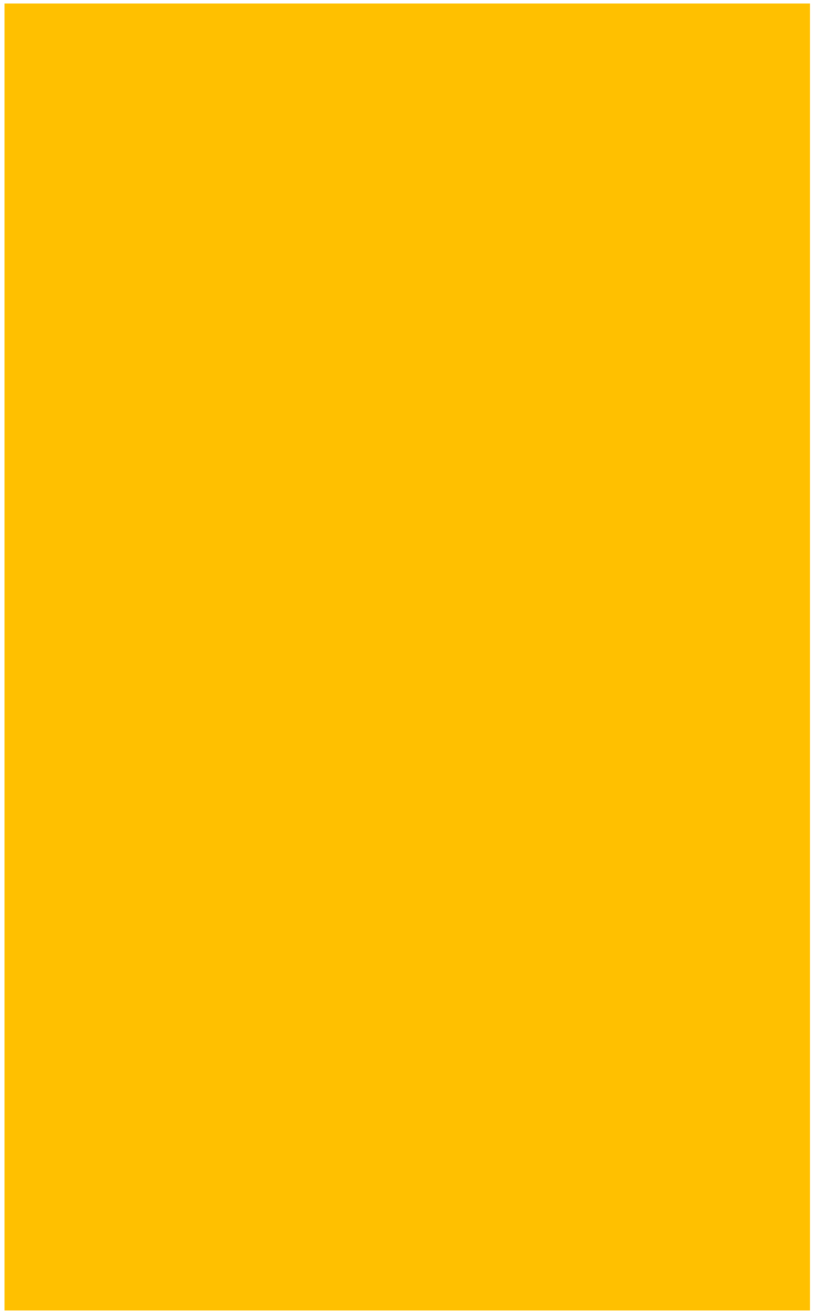

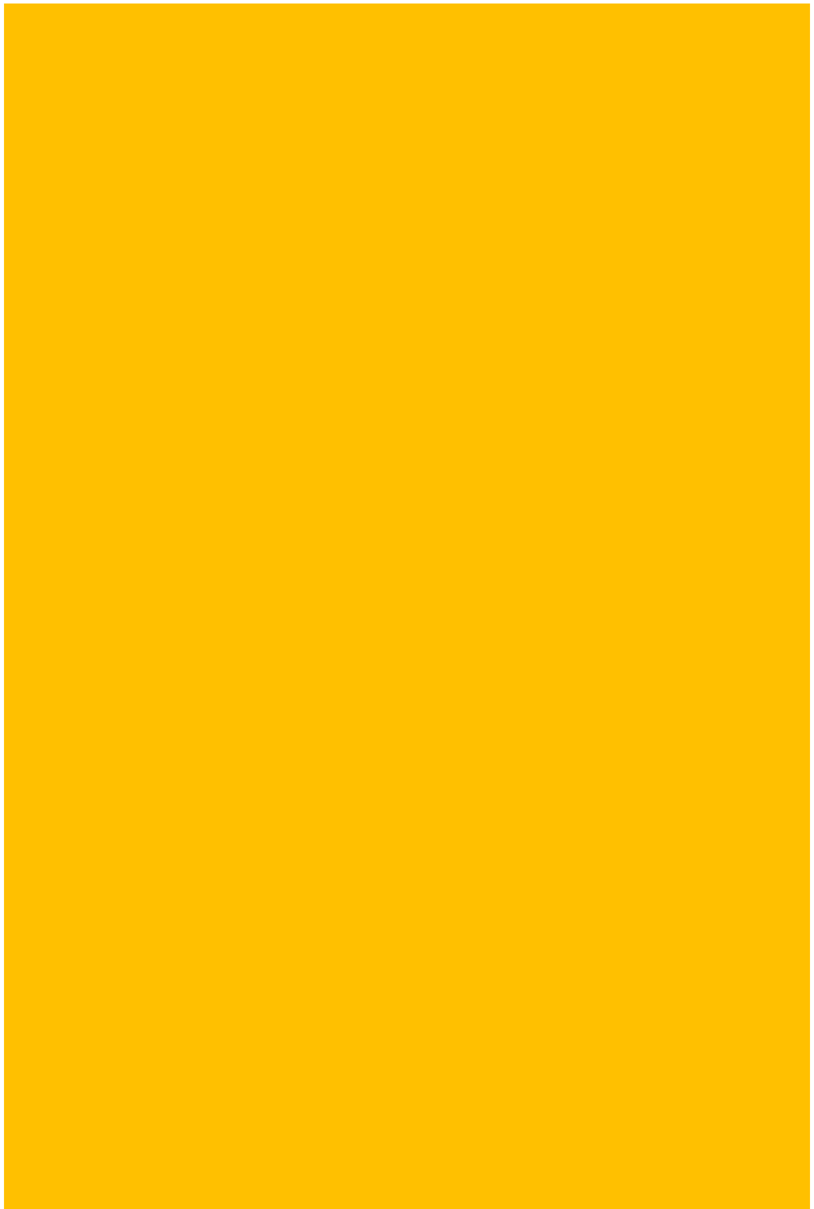

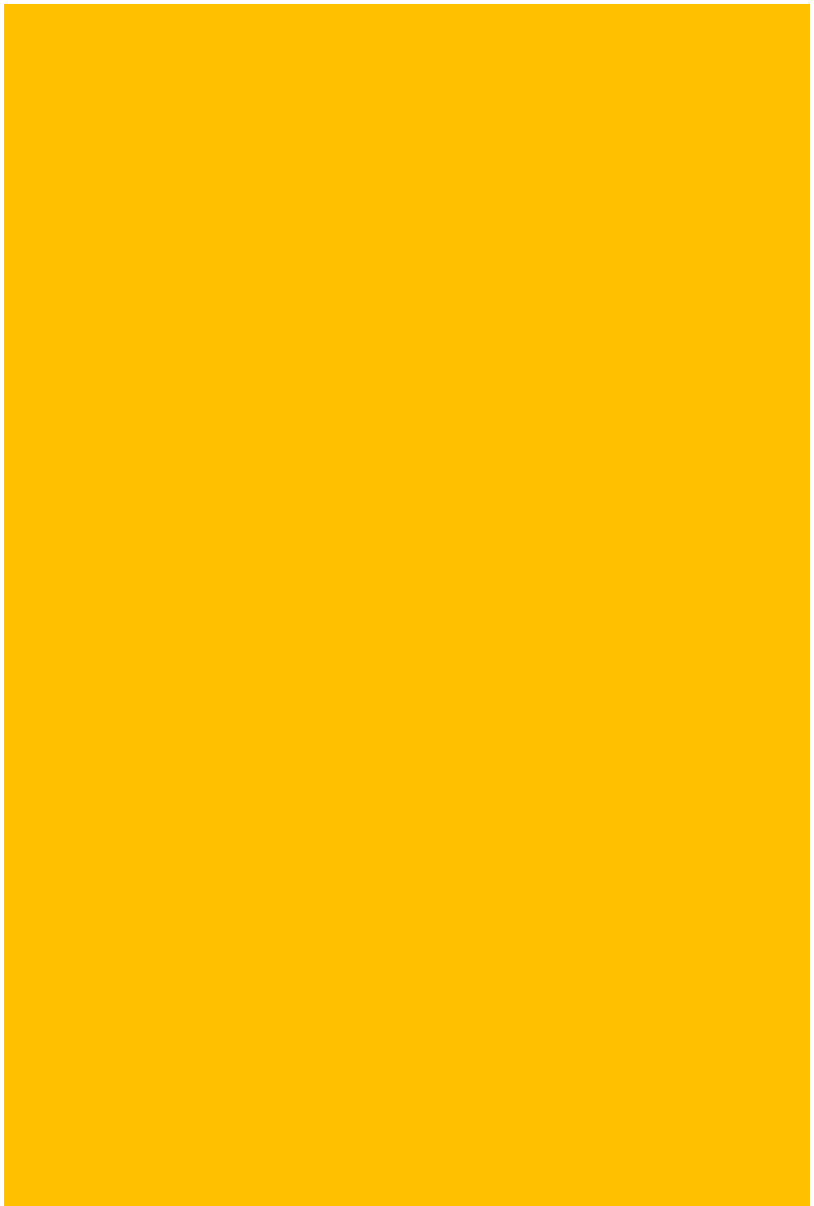

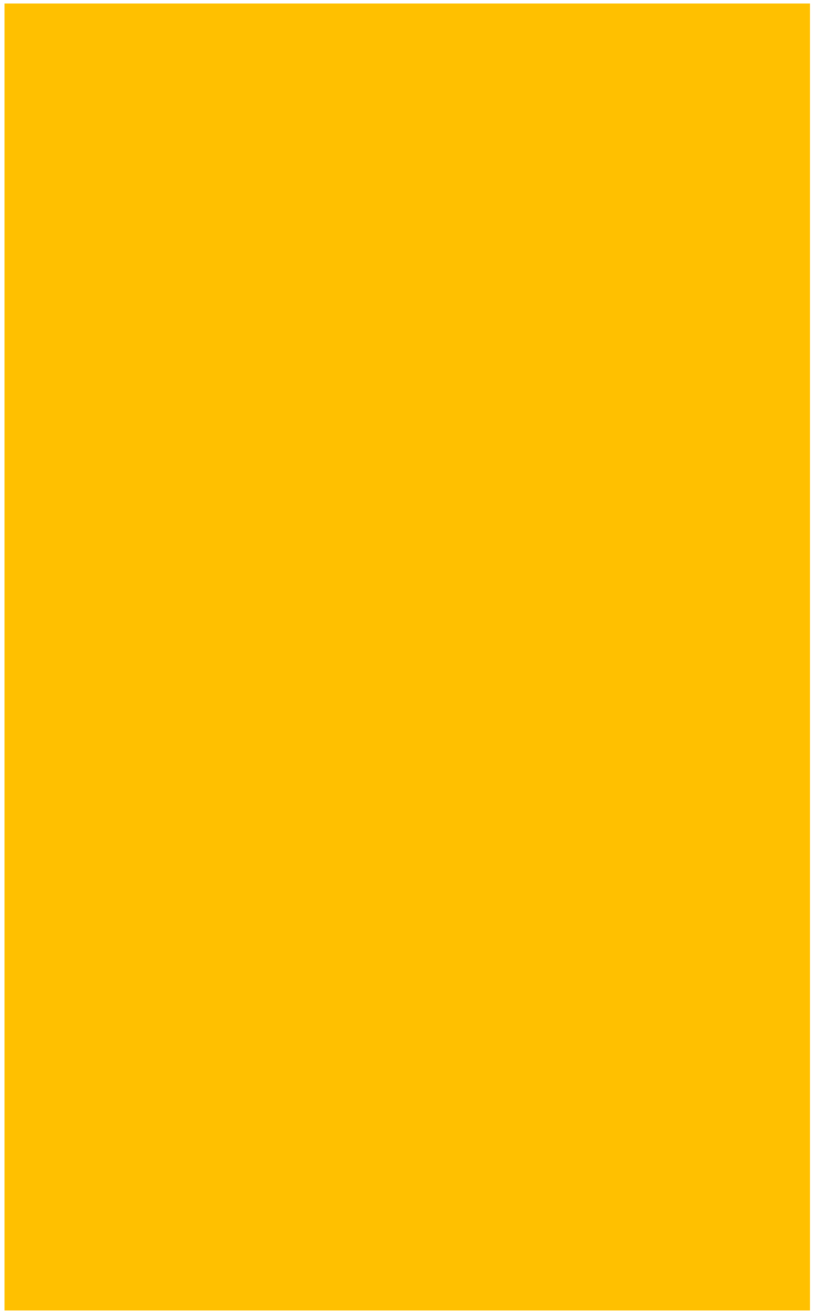

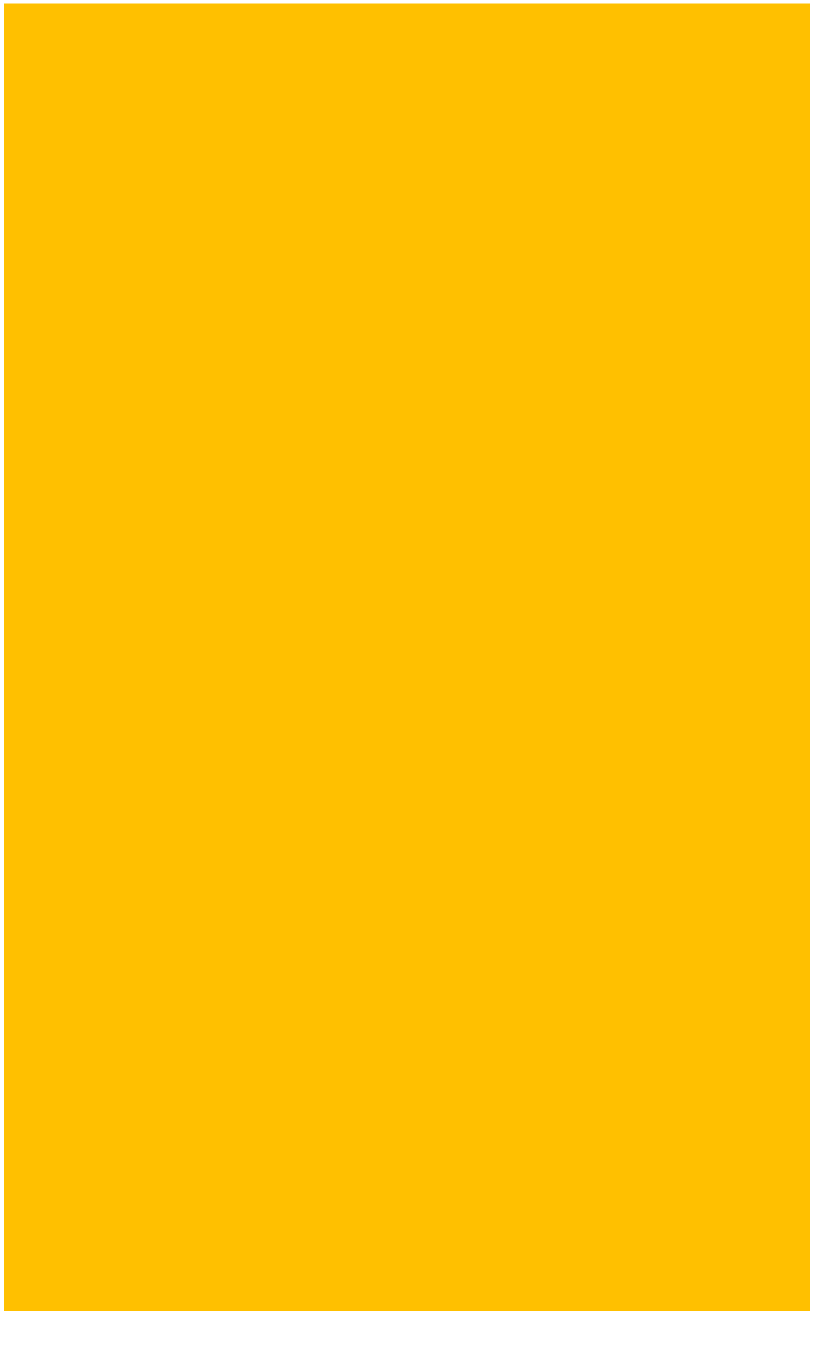

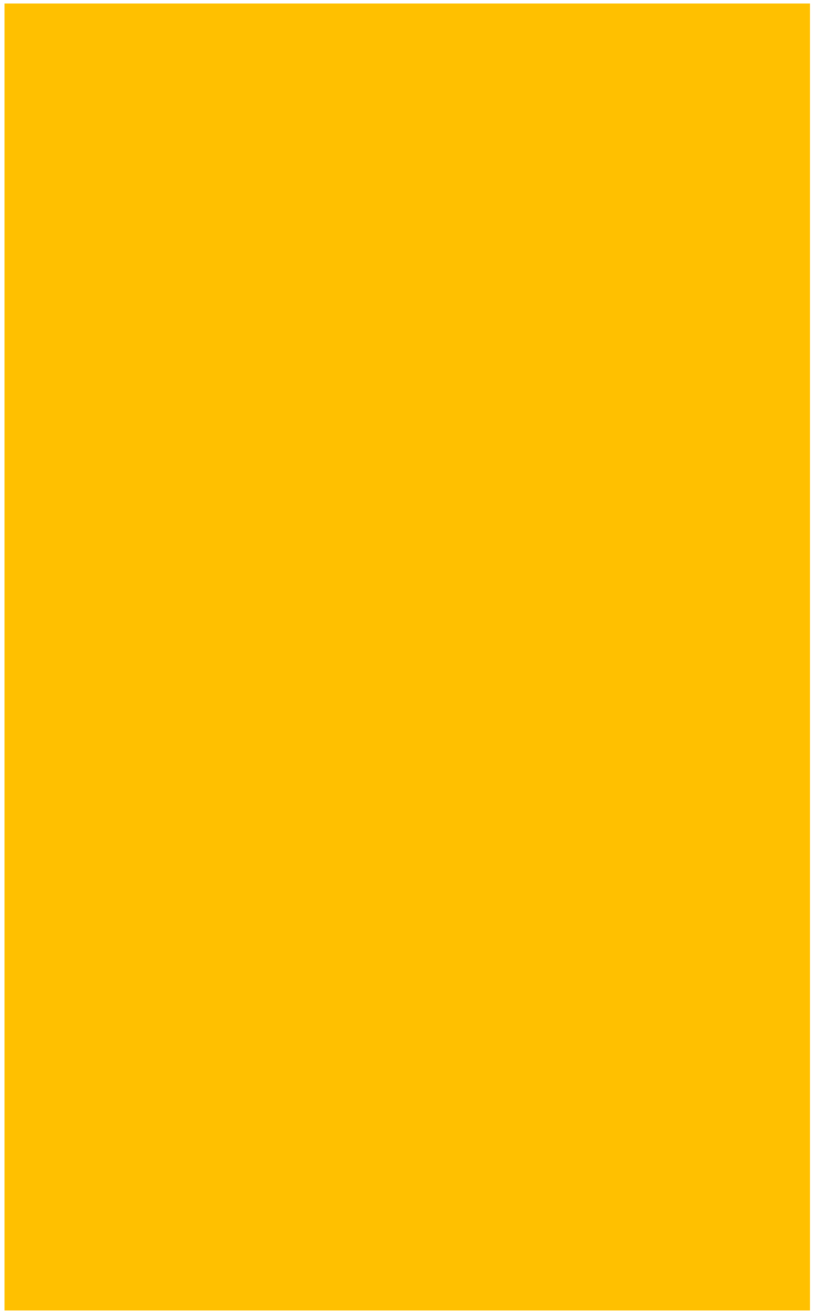

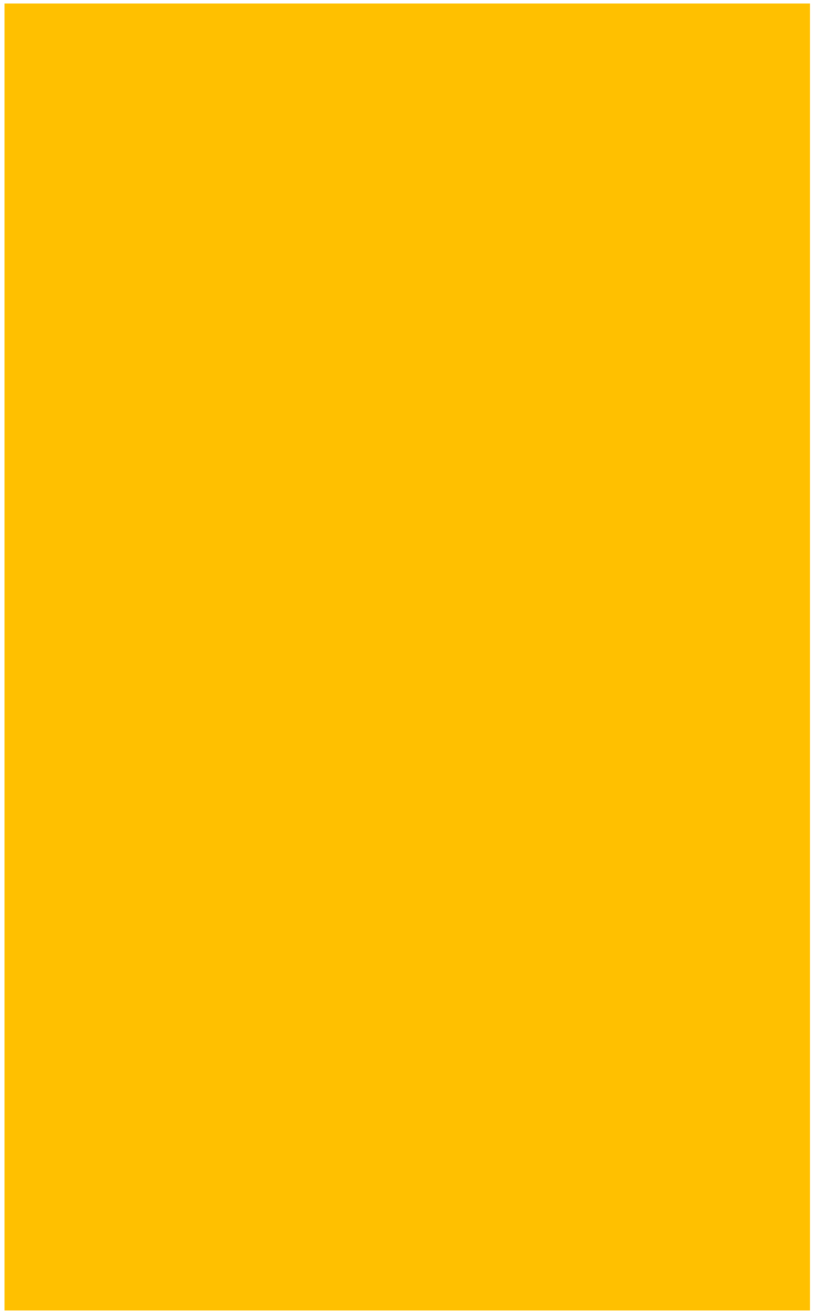

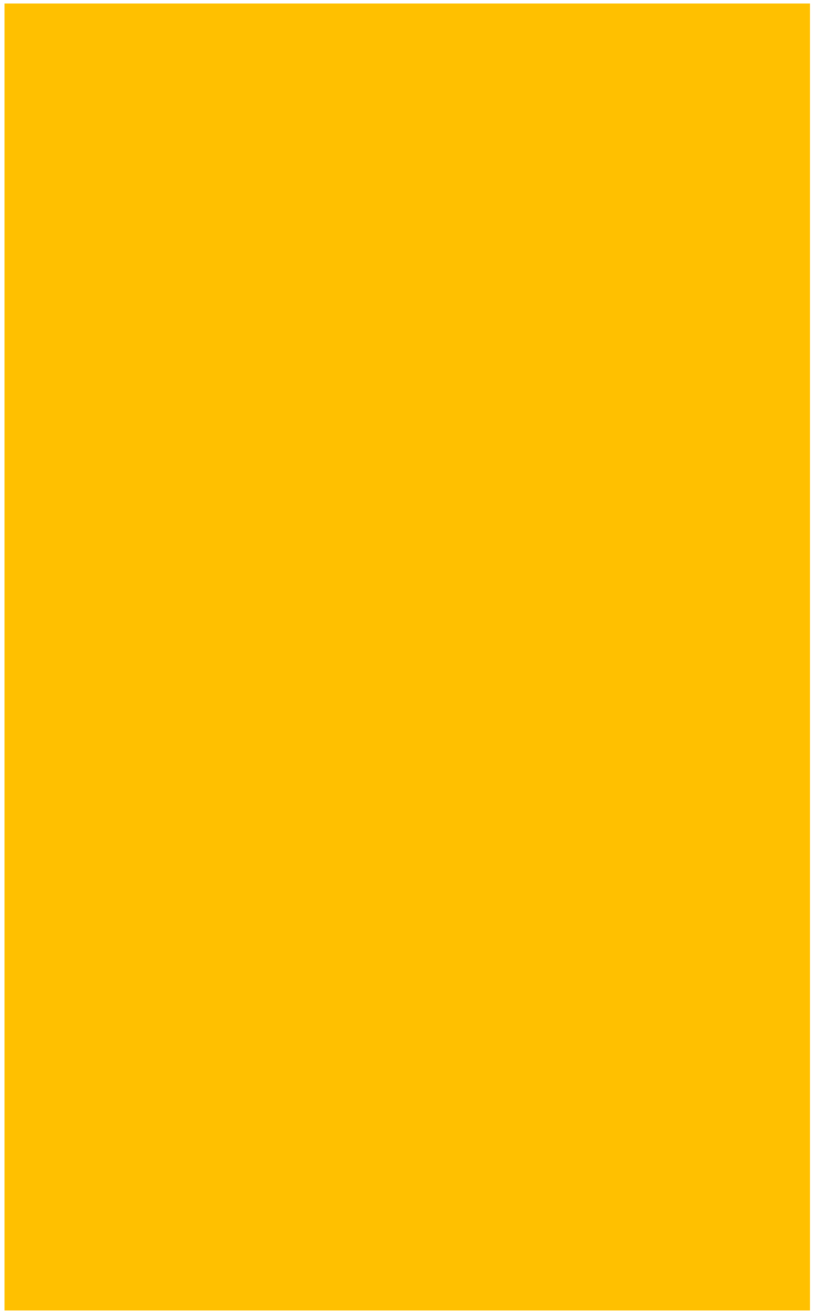

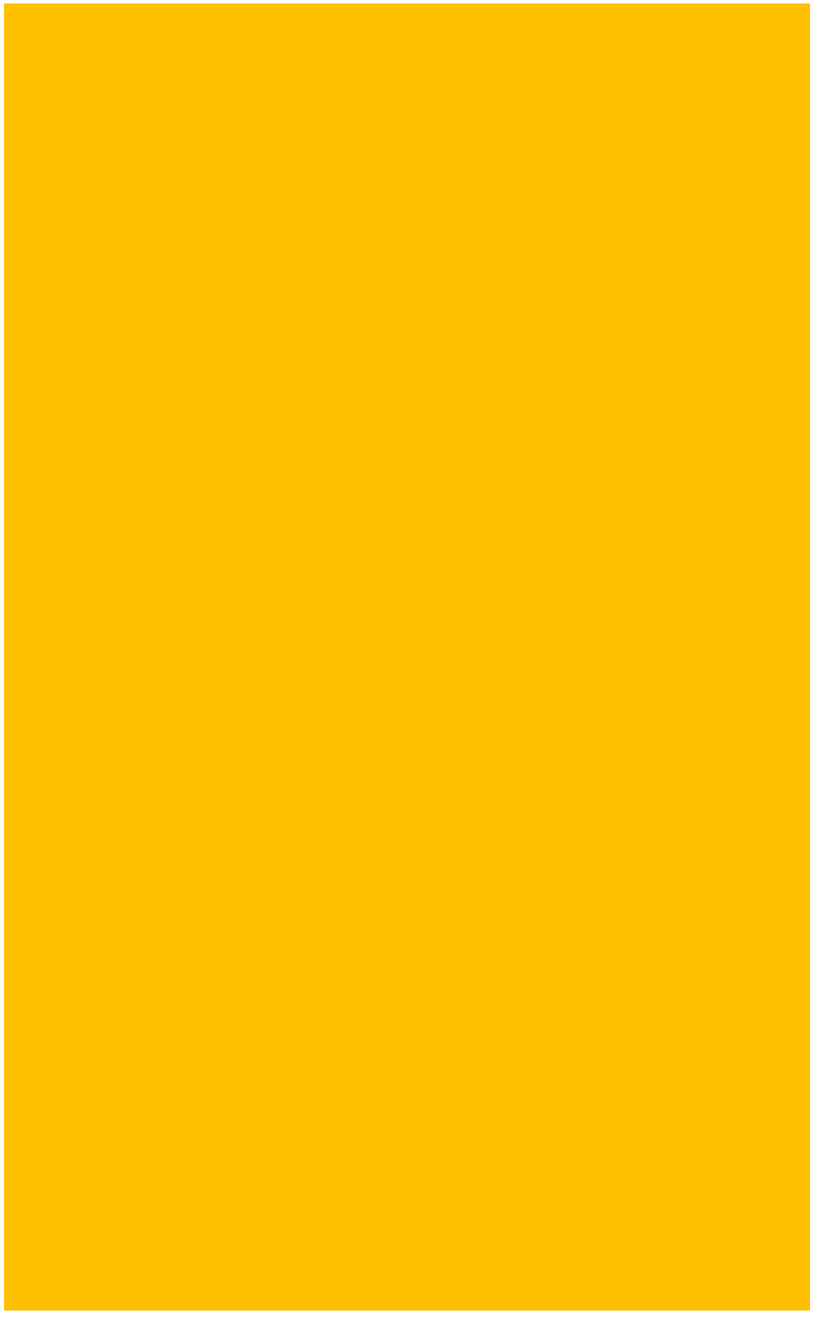

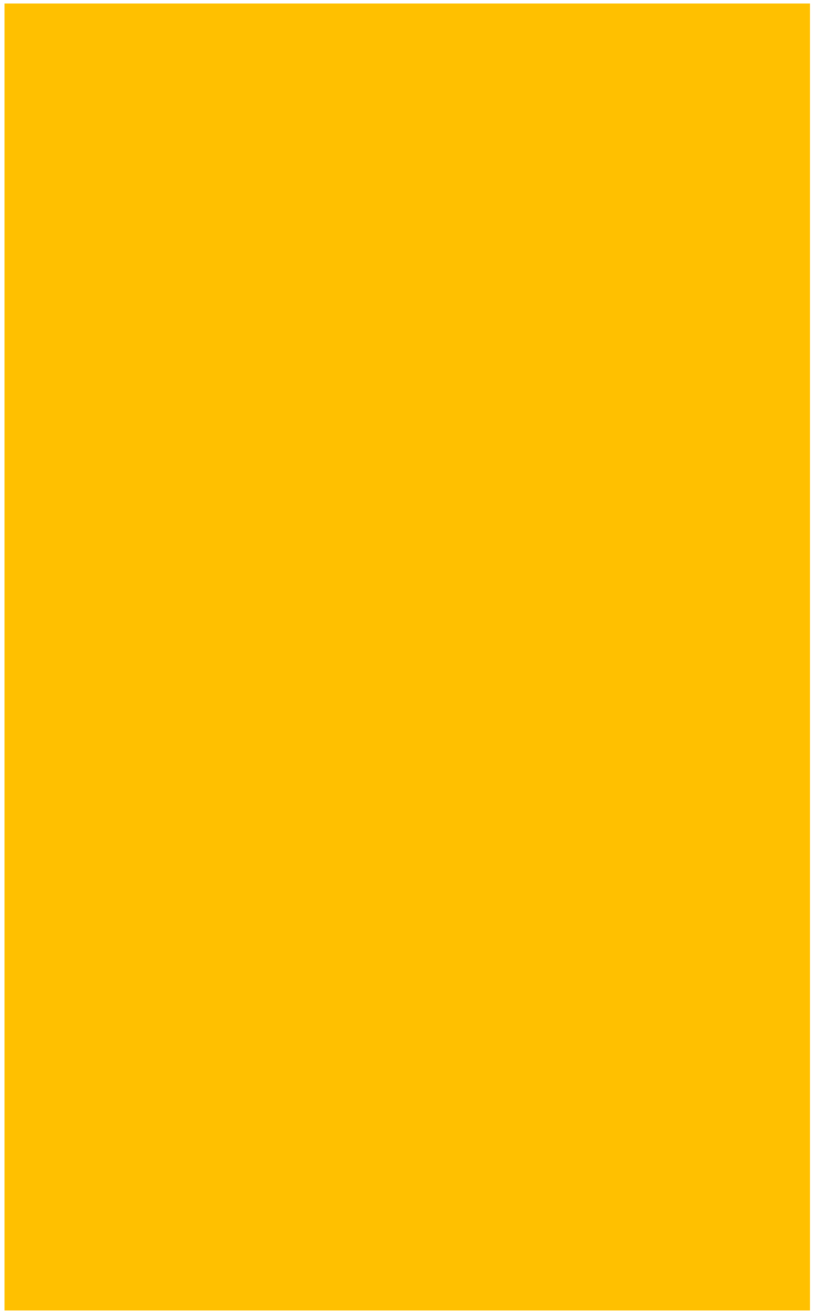

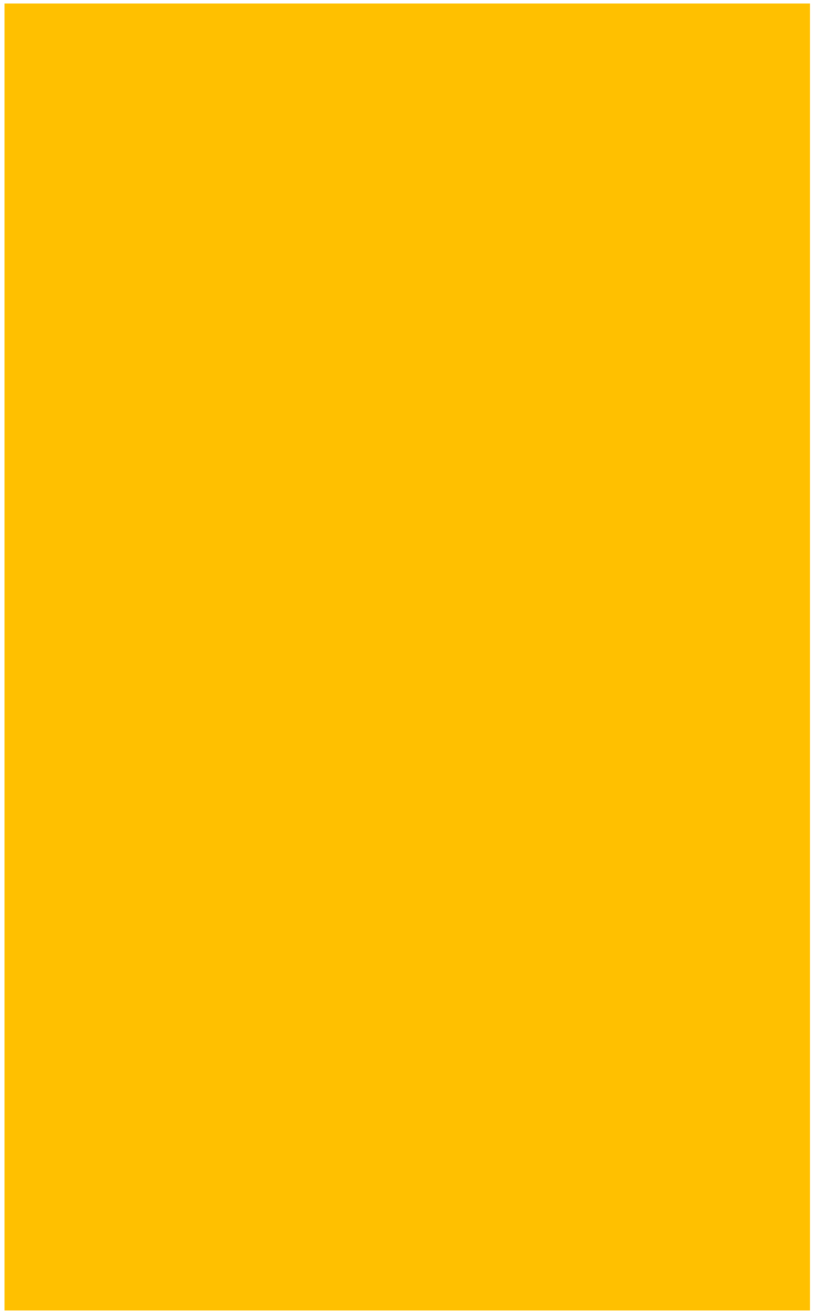

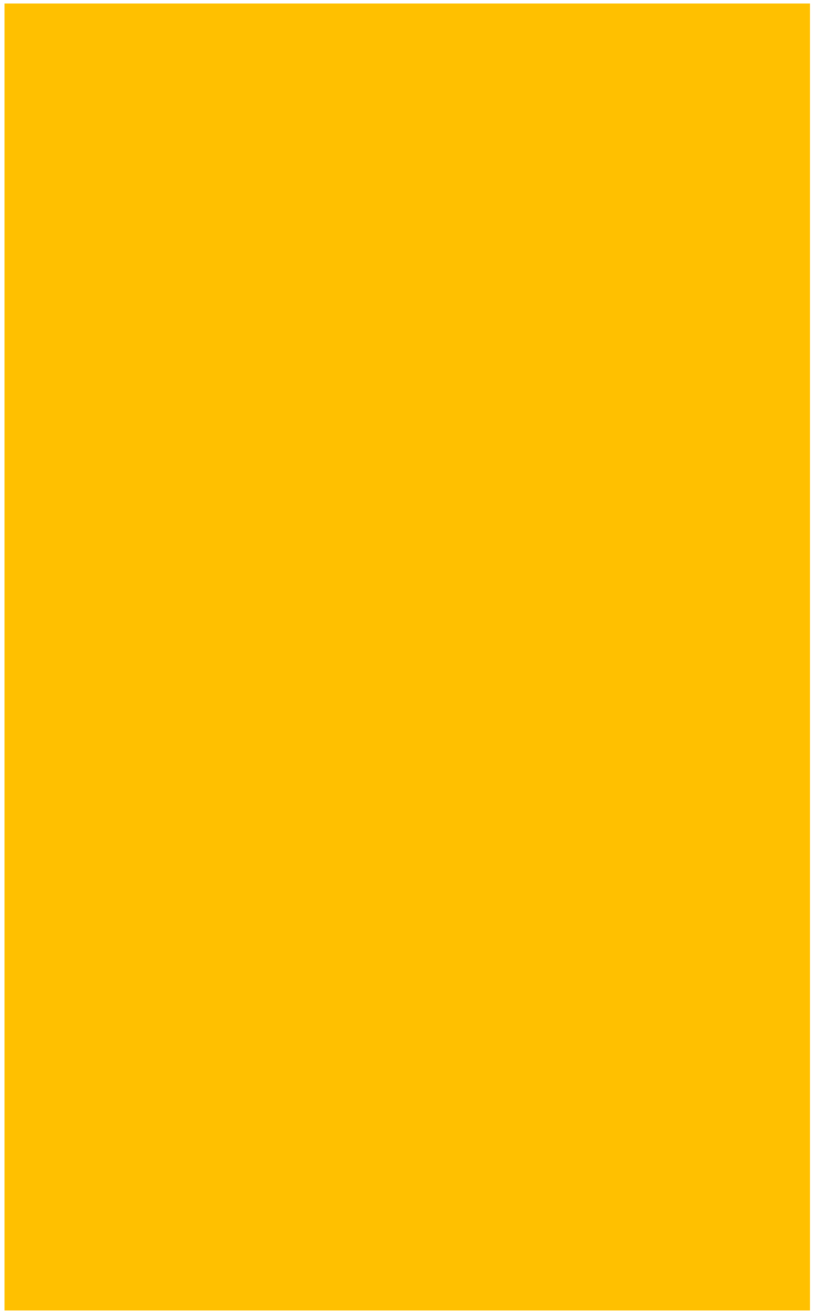

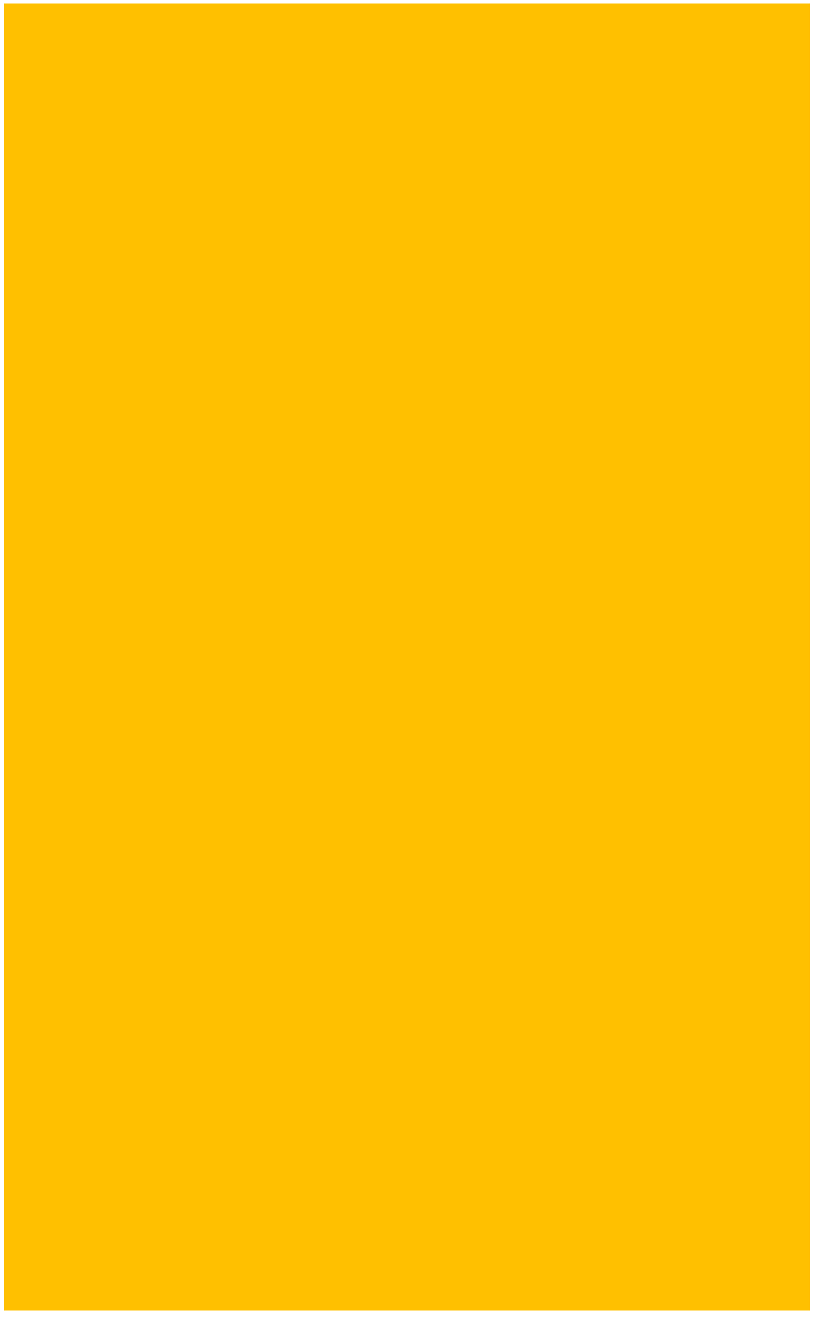

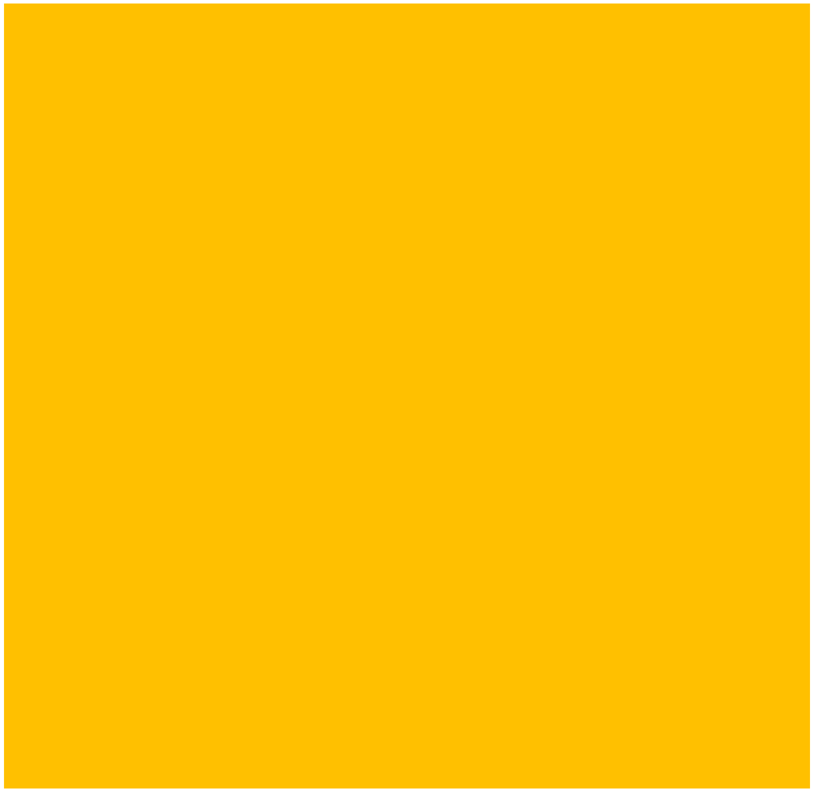

Adj. P-value: (CM_neg_newanalysis_12022024) / (WATER_NE(	Adj. P-value: (RS_NEG_NEWANALYSIS_12022024) / (WATER_NEG_N
0.735100571	7.35869E-06
1	4.86255E-07
1	0.000122891
0.04000326	2.07636E-06
0.181213954	1.2948E-06
1	0.802425808
2.29343E-09	9.25864E-10
0.000142293	1.88888E-05
0.005756991	0.001524677
1.9543E-07	3.93637E-07
2.5002E-06	0.00034499
0.985320045	1
0.310503681	0.125999299
0.222520365	0.002999122
0.047322862	0.000380444
0.638561423	0.004649258
0.479956303	0.991193629
1	1.10163E-06
1	0.000389735
1	0.105547855
1	0.962804149
0.154419344	1.36058E-06
1	0.070571698
0.208665194	2.91098E-05
1	1
0.015763938	0.000876388
0.004979188	0.000218644
0.127016152	0.035823185
0.386153686	0.0002973
1	1
0.154419344	0.029041798
0.369466983	0.351075791
0.030314213	2.0506E-10

1	1
1	1
1	0.377547438
1	1
0.29069512	5.45879E-05
1	0.080030973
1	0.029021368
1	0.1672094
0.654704505	8.62021E-05
1	0.009572918
0.487657064	0.002701993
0.392408075	0.000875197
1	1.32826E-06
0.68468713	1
0.235534834	0.02829398
0.280804514	0.184615448
0.000205917	9.99209E-11
0.966634453	0.675286262
1	1
1	0.022238253
1	4.75061E-09
1	0.000448812
1	0.449736778
0.069665705	1.17185E-05
1	2.26488E-06
0.21013211	1.51217E-05
1	4.41266E-07
5.27412E-05	0.005868528
0.287925427	1.86629E-08
0.590159415	0.005743584
0.48254113	0.821863707
3.6028E-06	5.93261E-10
0.117507281	1.06454E-07
1	0.142532673
0.958950323	0.632940528

0.218303009	0.813724554
1	0.338202443
1	1.34663E-05
0.693430181	0.207649074
0.19455115	1
1	0.572532306
1	0.023746543
1	0.000523184
0.89381264	0.935864609
1	0.017033055
0.564085455	1
0.132054994	3.94458E-07
0.001213766	6.9627E-08
0.32413078	4.88001E-09
1	3.1584E-08
1	1
1	0.474515933
1	0.001615489
0.273353045	2.90643E-05
0.976880318	1
1.2276E-07	2.0953E-11
0.001193332	6.35914E-09
0.275795211	1
0.28860693	5.1621E-06
1	0.000999577
0.427474292	0.4247778
0.139205281	8.02661E-06
0.203512793	4.39805E-05
0.989428889	0.4220608
1	0.006372792
0.003714204	4.06101E-08
1	1
1	0.80723938
1	0.203237357
0.186216363	8.46846E-06

0.943136045	1
0.491591947	1
0.65443256	1
0.318058771	0.001059042
0.653383498	1
4.68881E-07	8.54557E-12
1	9.18133E-12
0.001742721	8.58883E-12
7.86516E-07	8.09813E-11
0.00181154	7.99329E-10
1	8.54557E-12
0.838380532	0.001207503
1	0.001202383
0.341672369	0.000275387
0.512063429	0.002708249
0.97537082	0.016927719
0.43734435	0.399420862
0.961781455	1
0.316563887	0.003975385
1	0.594507858
0.889745466	0.00302217
0.70828587	2.7803E-05
1	0.753614868
0.44785243	1
0.796249118	0.006121286
1	0.058479385
1	0.683797809
0.015523013	8.54557E-12
0.807606617	0.000314223
1.87923E-05	8.54557E-12
0.13342107	9.92556E-08
0.008180991	2.39281E-08
0.185837406	5.64166E-07
0.035044582	1.30556E-08
1	1

1	0.267984254
0.32467204	0.00969829
1	0.038082794
1	0.426979813
0.962734154	5.29626E-06
0.030595297	7.22194E-11
0.496670411	0.000435706
1	3.71157E-09
0.137599611	3.16652E-11
1	5.96684E-07
3.37539E-08	8.54557E-12
1	0.492435639
1	3.62431E-08
1	0.001948528
0.000123171	9.02805E-12
1.98757E-07	8.54557E-12
1	0.67408985
0.045531556	0.865676608
1	0.000791912
0.201277563	7.737E-07
1	1
1	3.39885E-05
1	0.148329451
0.109358472	3.18175E-08
0.053249789	8.54557E-12
1	2.70661E-07
1.50243E-05	8.54557E-12
1	0.89579158
0.259187068	0.010904113
0.000621466	8.54557E-12
3.88203E-07	8.54557E-12
8.68424E-07	8.54557E-12
1.83109E-10	8.54557E-12
1	2.84165E-11
0.278304937	0.03589836

0.000353051	1.1579E-10
0.001160427	8.54557E-12
0.06333724	1.55135E-11
1	1.00184E-09
1	3.33888E-07
0.184282302	8.66798E-10
0.225844471	1.92951E-08
0.321558031	0.045812845

Adj. P-value: (SM_neg_newanalysis_12022024) / (WATER_NEG_NEWANALYSIS_12022024)
0.036975934
0.120325272
0.790301597
0.000434061
0.005817218
1
7.19567E-11
1.72761E-07
2.35531E-05
1.50931E-09
1.53964E-07
1
0.941935293
0.289338068
0.65973932
0.445214305
0.027355279
0.740386316
0.058556097
0.911311241
0.504177176
0.005013725
1
0.073450073
0.044649635
2.97289E-07
5.82393E-06
1.21416E-05
0.019716293
0.008623282
0.000201464
0.000205886
0.003268729

	0.364738826
	1
	1
	0.837758194
	0.021045548
	0.94957543
	0.737679956
	0.433777541
	0.06076251
	1
	0.099912281
	0.041930926
	0.08896773
	0.037204345
	0.026900213
	0.145697714
	4.89985E-06
	0.438623801
	0.056703777
	0.360333725
	0.049623004
	0.40146101
	1
	0.003661942
	0.050945714
	0.008526608
	1
	0.076669002
	0.000679524
	0.021705296
	0.017965068
	1.70712E-07
	0.038396827
	0.546890157
	1

	0.393124628
	1
	0.001325503
	1
	0.010217501
	1
	1
	0.243734008
	0.281144232
	0.811817759
	0.020996478
	0.002797461
	3.61387E-05
	0.002076806
	0.978285154
	0.046889199
	0.525814557
	0.016493276
	0.009434245
	0.376622267
	1.14844E-08
	3.27529E-05
	0.010899169
	0.005085252
	0.093323213
	0.072411192
	0.004231742
	0.01725548
	0.175228857
	0.804917303
	9.49097E-05
	0.078047128
	0.85681806
	0.552288435
	0.003936184

	0.806975243
	1
	0.070579342
	0.071490582
	0.358366232
	3.45289E-09
	0.01401318
	4.11246E-06
	1.68732E-09
	8.69336E-07
	0.33817806
	0.274016228
	0.999775061
	0.042733609
	0.146675194
	0.776939751
	0.00680639
	1
	0.031960646
	1
	0.683624591
	0.036102451
	0.317999663
	0.004756853
	0.665653787
	0.348024968
	0.272377409
	1.10536E-07
	1
	5.4037E-10
	0.382712447
	9.79411E-05
	9.84978E-05
	0.045909868
	0.786557157

1
0.023435525
0.516535989
0.322059408
0.919393056
0.049325195
1
0.353167018
0.002161772
1
2.05814E-10
0.858428127
0.053152008
1
5.41894E-07
7.24002E-10
1
0.001763891
0.044660836
0.02234617
0.374824984
1
0.909157194
0.00271177
0.000266729
0.542690395
5.70417E-05
0.397908812
0.010202897
4.26995E-05
2.87787E-09
1.14935E-07
7.19567E-11
0.084876268
1

	3.53363E-05
	9.50557E-06
	0.002605939
	0.298551592
	1
	0.012608771
	0.002884013
	0.005255148
