# Supplementary material for: A lipid compendium of a metabolically compromised bacterium provides insights into lipid acquisition, biosynthesis, and metabolism
Source: bioRxiv. 2026 May 23:2026.05.22.727245. Preprint. [Version 1] doi: 10.64898/2026.05.22.727245 (PMC13228262; doi:10.64898/2026.05.22.727245)
Supplement: Supplement 4 [file media-4.pdf]

Supplementary Spreadsheet 4: MRM (mass reaction monitoring) parameters fo

›r targeted lipid analysis including transitions, collision energies, and instrumenta

l settings in triple quadrupole

Compound group	Compound name	Compound formula	Ion speci	CAS	z	Monoisot	ISTD?
AcylCarnitine(12:0)	AcylCarnitine(12:0)	C42H82NO10P	[M+H]+		1		FALSE
AcylCarnitine(14:0)	AcylCarnitine(14:0)	C42H82NO10P	[M+H]+		1		FALSE
AcylCarnitine(14:1)	AcylCarnitine(14:1)	C42H82NO10P	[M+H]+		1		FALSE
AcylCarnitine(14:2)	AcylCarnitine(14:2)	C42H82NO10P	[M+H]+		1		FALSE
AcylCarnitine(16:0)	AcylCarnitine(16:0)	C42H82NO10P	[M+H]+		1		FALSE
AcylCarnitine(16:1)	AcylCarnitine(16:1)	C42H82NO10P	[M+H]+		1		FALSE
AcylCarnitine(18:0)	AcylCarnitine(18:0)	C42H82NO10P	[M+H]+		1		FALSE
AcylCarnitine(18:1)	AcylCarnitine(18:1)	C42H82NO10P	[M+H]+		1		FALSE
AcylCarnitine(18:2)	AcylCarnitine(18:2)	C42H82NO10P	[M+H]+		1		FALSE
AcylCarnitine(20:0)	AcylCarnitine(20:0)	C42H82NO10P	[M+H]+		1		FALSE
AcHexCmE(18:1)_a	AcHexCmE(18:1)_a	C52H91O7	[M+H]+		1		FALSE
AcHexCmE(18:1)_b	AcHexCmE(18:1)_b	C52H91O7	[M+NH4]+		1		FALSE
C-Gal(a)	C-Gal(a)	$C_{33}H_{56}O_{6}$	[M+Na]+		1		FALSE
C-Gal(b)	C-Gal(b)	$C_{33}H_{56}O_{6}$	[M+Na]+		1		FALSE
BbGL-1(a)	BbGL-1(a)	$C_{49}H_{86}O_{7}$	[M+NH4]+		1		FALSE
BbGL-1(b)	BbGL-1(b)	$C_{49}H_{86}O_{7}$	[M+NH4]+		1		FALSE
CE(16:0)	CE(16:0)	C42H82NO10P	[M+NH4]+		1		FALSE
CE(16:1)	CE(16:1)	C42H82NO10P	[M+NH4]+		1		FALSE
CE(16:2)	CE(16:2)	C42H82NO10P	[M+NH4]+		1		FALSE
CE(17:0)	CE(17:0)	C42H82NO10P	[M+NH4]+		1		FALSE
CE(17:1)	CE(17:1)	C42H82NO10P	[M+NH4]+		1		FALSE
CE(18:0)	CE(18:0)	C42H82NO10P	[M+NH4]+		1		FALSE
CE(18:1)	CE(18:1)	C42H82NO10P	[M+NH4]+		1		FALSE
CE(18:2)	CE(18:2)	C42H82NO10P	[M+NH4]+		1		FALSE
CE(18:3)	CE(18:3)	C42H82NO10P	[M+NH4]+		1		FALSE
CE(20:1)	CE(20:1)	C42H82NO10P	[M+NH4]+		1		FALSE
CE(20:2)	CE(20:2)	C42H82NO10P	[M+NH4]+		1		FALSE
CE(20:3)	CE(20:3)	C42H82NO10P	[M+NH4]+		1		FALSE
CE(20:4)	CE(20:4)	C42H82NO10P	[M+NH4]+		1		FALSE
CE(20:5)	CE(20:5)	C42H82NO10P	[M+NH4]+		1		FALSE
CE(22:0)	CE(22:0)	C42H82NO10P	[M+NH4]+		1		FALSE
CE(22:1)	CE(22:1)	C42H82NO10P	[M+NH4]+		1		FALSE
CE(22:4)	CE(22:4)	C42H82NO10P	[M+NH4]+		1		FALSE
CE(22:5) (n3)	CE(22:5) (n3)	C42H82NO10P	[M+NH4]+		1		FALSE
CE(22:6)	CE(22:6)	C42H82NO10P	[M+NH4]+		1		FALSE
CE(24:0)	CE(24:0)	C42H82NO10P	[M+NH4]+		1		FALSE
CE(24:1)	CE(24:1)	C42H82NO10P	[M+NH4]+		1		FALSE

CE(24:4)	CE(24:4)	C42H82NO10P	[M+NH4]+	1	FALSE
CE(24:5)	CE(24:5)	C42H82NO10P	[M+NH4]+	1	FALSE
CE(24:6)	CE(24:6)	C42H82NO10P	[M+NH4]+	1	FALSE
CmE(18:3)	CmE(18:3)	$C_{46}H_{76}O_2$	[M+NH4]+	1	FALSE
Cer(d16:1/16:0)	Cer(d16:1/16:0)		[M+H]+	1	FALSE
Cer(d16:1/18:0)	Cer(d16:1/18:0)		[M+H]+	1	FALSE
Cer(d16:1/20:0)	Cer(d16:1/20:0)		[M+H]+	1	FALSE
Cer(d16:1/22:0)	Cer(d16:1/22:0)		[M+H]+	1	FALSE
Cer(d16:1/23:0)	Cer(d16:1/23:0)		[M+H]+	1	FALSE
Cer(d16:1/24:0)	Cer(d16:1/24:0)		[M+H]+	1	FALSE
Cer(d16:1/24:1)	Cer(d16:1/24:1)		[M+H]+	1	FALSE
Cer(d18:1/14:0)	Cer(d18:1/14:0)		[M+H]+	1	FALSE
Cer(d18:1/16:0)	Cer(d18:1/16:0)		[M+H]+	1	FALSE
Cer(d18:1/18:0)	Cer(d18:1/18:0)		[M+H]+	1	FALSE
Cer(d18:1/19:0)	Cer(d18:1/19:0)		[M+H]+	1	FALSE
Cer(d18:1/20:0)	Cer(d18:1/20:0)		[M+H]+	1	FALSE
Cer(d18:1/21:0)	Cer(d18:1/21:0)		[M+H]+	1	FALSE
Cer(d18:1/22:0)	Cer(d18:1/22:0)		[M+H]+	1	FALSE
Cer(d18:1/23:0)	Cer(d18:1/23:0)		[M+H]+	1	FALSE
Cer(d18:1/24:0)	Cer(d18:1/24:0)		[M+H]+	1	FALSE
Cer(d18:1/24:1)	Cer(d18:1/24:1)		[M+H]+	1	FALSE
Cer(d18:1/26:0)	Cer(d18:1/26:0)		[M+H]+	1	FALSE
Cer(d18:2/14:0)	Cer(d18:2/14:0)		[M+H]+	1	FALSE
Cer(d18:2/16:0)	Cer(d18:2/16:0)		[M+H]+	1	FALSE
Cer(d18:2/18:0)	Cer(d18:2/18:0)		[M+H]+	1	FALSE
Cer(d18:2/20:0)	Cer(d18:2/20:0)		[M+H]+	1	FALSE
Cer(d18:2/21:0)	Cer(d18:2/21:0)		[M+H]+	1	FALSE
Cer(d18:2/22:0)	Cer(d18:2/22:0)		[M+H]+	1	FALSE
Cer(d18:2/23:0)	Cer(d18:2/23:0)		[M+H]+	1	FALSE
Cer(d18:2/24:0)	Cer(d18:2/24:0)		[M+H]+	1	FALSE
Cer(d18:2/24:1)	Cer(d18:2/24:1)		[M+H]+	1	FALSE
Cer(d18:2/26:0)	Cer(d18:2/26:0)		[M+H]+	1	FALSE
Cer(d20:1/22:0)	Cer(d20:1/22:0)		[M+H]+	1	FALSE
Cer(d20:1/23:0)	Cer(d20:1/23:0)		[M+H]+	1	FALSE
Cer(d20:1/24:0)	Cer(d20:1/24:0)		[M+H]+	1	FALSE
Cer(d20:1/24:1)	Cer(d20:1/24:1)		[M+H]+	1	FALSE
Cer(d20:1/26:0)	Cer(d20:1/26:0)		[M+H]+	1	FALSE
Cer1P(d18:1/16:0)	Cer1P(d18:1/16:0)		[M+H]+	1	FALSE

Cer(d18:1_24:2)	Cer(d18:1_24:2)		[M+H]+	1	FALSE
Cer(d27:1)	Cer(d27:1)		[M+H]+	1	FALSE
Cer(d30:0)	Cer(d30:0)		[M+H]+	1	FALSE
Cer(d31:1)	Cer(d31:1)		[M+H]+	1	FALSE
Cer(d32:0)	Cer(d32:0)		[M+H]+	1	FALSE
Cer(d33:1)	Cer(d33:1)		[M+H]+	1	FALSE
Cer(d34:0)	Cer(d34:0)		[M+H]+	1	FALSE
Cer(d34:1)	Cer(d34:1)		[M+H]+	1	FALSE
Cer(d36:0)	Cer(d36:0)		[M+H]+	1	FALSE
Cer(m18:0_16:0)	Cer(m18:0_16:0)		[M+H]+	1	FALSE
Cer(m36:0)	Cer(m36:0)		[M+H]+	1	FALSE
Cer(t20:1_16:0)	Cer(t20:1_16:0)		[M+H]+	1	FALSE
Cer(t20:1_18:0)	Cer(t20:1_18:0)		[M+H]+	1	FALSE
COH	COH	C42H82NO10P	[M+NH4]+	1	FALSE
DE(16:0)	DE(16:0)	C42H82NO10P	[M+NH4]+	1	FALSE
DE(18:1)	DE(18:1)	C42H82NO10P	[M+NH4]+	1	FALSE
DE(18:2)	DE(18:2)	C42H82NO10P	[M+NH4]+	1	FALSE
DE(20:4)	DE(20:4)	C42H82NO10P	[M+NH4]+	1	FALSE
DE(20:5)	DE(20:5)	C42H82NO10P	[M+NH4]+	1	FALSE
DE(22:6)	DE(22:6)	C42H82NO10P	[M+NH4]+	1	FALSE
DG(14:0_16:0)	DG(14:0_16:0)	C42H82NO10P	[M+NH4]+	1	FALSE
DG(14:0_16:0)	DG(14:0_16:0)	C42H82NO10P	[M+NH4]+	1	FALSE
DG(14:0_18:2)	DG(14:0_18:2)	C42H82NO10P	[M+NH4]+	1	FALSE
DG(14:0_18:2)	DG(14:0_18:2)	C42H82NO10P	[M+NH4]+	1	FALSE
DG(16:0_16:0)	DG(16:0_16:0)	C42H82NO10P	[M+NH4]+	1	FALSE
DG(16:0_16:1)	DG(16:0_16:1)	C42H82NO10P	[M+NH4]+	1	FALSE
DG(16:0_16:1)	DG(16:0_16:1)	C42H82NO10P	[M+NH4]+	1	FALSE
DG(16:0_18:1)	DG(16:0_18:1)	C42H82NO10P	[M+NH4]+	1	FALSE
DG(16:0_18:1)	DG(16:0_18:1)	C42H82NO10P	[M+NH4]+	2	FALSE
DG(16:0_18:2)	DG(16:0_18:2)	C42H82NO10P	[M+NH4]+	1	FALSE
DG(16:0_18:2)	DG(16:0_18:2)	C42H82NO10P	[M+NH4]+	1	FALSE
DG(16:0_20:4)	DG(16:0_20:4)	C42H82NO10P	[M+NH4]+	1	FALSE
DG(16:0_20:4)	DG(16:0_20:4)	C42H82NO10P	[M+NH4]+	1	FALSE
DG(16:0_22:5)	DG(16:0_22:5)	C42H82NO10P	[M+NH4]+	1	FALSE
DG(16:0_22:5)	DG(16:0_22:5)	C42H82NO10P	[M+NH4]+	1	FALSE
DG(16:0_22:6)	DG(16:0_22:6)	C42H82NO10P	[M+NH4]+	1	FALSE
DG(16:0_22:6)	DG(16:0_22:6)	C42H82NO10P	[M+NH4]+	1	FALSE
DG(16:1_18:1)	DG(16:1_18:1)	C42H82NO10P	[M+NH4]+	1	FALSE

DG(16:1_18:1)	DG(16:1_18:1)	C42H82NO10P	[M+NH4]+	1	FALSE
DG(18:0_18:1)	DG(18:0_18:1)	C42H82NO10P	[M+NH4]+	1	FALSE
DG(18:0_18:1)	DG(18:0_18:1)	C42H82NO10P	[M+NH4]+	1	FALSE
DG(18:0_18:2)	DG(18:0_18:2)	C42H82NO10P	[M+NH4]+	1	FALSE
DG(18:0_18:2)	DG(18:0_18:2)	C42H82NO10P	[M+NH4]+	1	FALSE
DG(18:0_20:4)	DG(18:0_20:4)	C42H82NO10P	[M+NH4]+	1	FALSE
DG(18:0_20:4)	DG(18:0_20:4)	C42H82NO10P	[M+NH4]+	1	FALSE
DG(18:1_18:1)	DG(18:1_18:1)	C42H82NO10P	[M+NH4]+	1	FALSE
DG(18:1_18:2)	DG(18:1_18:2)	C42H82NO10P	[M+NH4]+	1	FALSE
DG(18:1_18:2)	DG(18:1_18:2)	C42H82NO10P	[M+NH4]+	1	FALSE
DG(18:1_20:3)	DG(18:1_20:3)	C42H82NO10P	[M+NH4]+	1	FALSE
DG(18:1_20:3)	DG(18:1_20:3)	C42H82NO10P	[M+NH4]+	1	FALSE

Precurso	MS1 res	Product m/	MS2 res	Dwell (ms	Fragmen	CE (V)	Polarity
344.3	Unit	85.1	Unit	20	166	30	Positive
372.3	Unit	85.1	Unit	20	166	30	Positive
370.3	Unit	85.1	Unit	20	166	30	Positive
368.3	Unit	85.1	Unit	20	166	30	Positive
400.4	Unit	85.1	Unit	20	166	30	Positive
398.3	Unit	85.1	Unit	20	166	30	Positive
428.4	Unit	85.1	Unit	20	166	30	Positive
426.4	Unit	85.1	Unit	20	166	30	Positive
424.3	Unit	85.1	Unit	20	166	30	Positive
456.4	Unit	85.1	Unit	20	166	30	Positive
827.7	Unit	383.3	Unit	20	166	23	Positive
844.7	Unit	383.3	Unit	20	166	23	Positive
571.4		369.3	Unit	20	166	23	Positive
571.4		203.1	Unit	20	166	23	Positive
804.7		369.3	Unit	20	166	23	Positive
804.7		401.3	Unit	20	166	23	Positive
642.6	Unit	369.3	Unit	20	166	10	Positive
640.6	Unit	369.3	Unit	20	166	10	Positive
638.6	Unit	369.3	Unit	20	166	10	Positive
656.6	Unit	369.3	Unit	20	166	10	Positive
654.6	Unit	369.3	Unit	20	166	10	Positive
670.7	Unit	369.3	Unit	20	166	10	Positive
668.6	Unit	369.3	Unit	20	166	10	Positive
666.6	Unit	369.3	Unit	20	166	10	Positive
664.6	Unit	369.3	Unit	20	166	10	Positive
696.7	Unit	369.3	Unit	20	166	10	Positive
694.7	Unit	369.3	Unit	20	166	10	Positive
692.6	Unit	369.3	Unit	20	166	10	Positive
690.6	Unit	369.3	Unit	20	166	10	Positive
688.6	Unit	369.3	Unit	20	166	10	Positive
726.7	Unit	369.3	Unit	20	166	10	Positive
724.7	Unit	369.3	Unit	20	166	10	Positive
718.7	Unit	369.3	Unit	20	166	10	Positive
716.6	Unit	369.3	Unit	20	166	10	Positive
714.6	Unit	369.3	Unit	20	166	10	Positive
754.7	Unit	369.3	Unit	20	166	10	Positive
752.7	Unit	369.3	Unit	20	166	10	Positive

746.7	Unit	369.3	Unit	20	166	10	Positive
744.7	Unit	369.3	Unit	20	166	10	Positive
742.7	Unit	369.3	Unit	20	166	10	Positive
678.6	Unit	383.3	Unit	20	166	20	Positive
510.6	Unit	236.3	Unit	20	166	10	Positive
538.6	Unit	236.3	Unit	20	166	10	Positive
566.6	Unit	236.3	Unit	20	166	29	Positive
594.6	Unit	236.3	Unit	20	166	29	Positive
608.6	Unit	236.3	Unit	20	166	29	Positive
622.6	Unit	236.3	Unit	20	166	29	Positive
620.6	Unit	236.3	Unit	20	166	29	Positive
510.5	Unit	264.3	Unit	20	166	29	Positive
538.5	Unit	264.3	Unit	20	166	29	Positive
566.6	Unit	264.3	Unit	20	166	29	Positive
580.6	Unit	264.3	Unit	20	166	29	Positive
594.6	Unit	264.3	Unit	20	166	29	Positive
608.6	Unit	264.3	Unit	20	166	29	Positive
622.6	Unit	264.3	Unit	20	166	29	Positive
636.6	Unit	264.3	Unit	20	166	29	Positive
650.6	Unit	264.3	Unit	20	166	29	Positive
648.6	Unit	264.3	Unit	20	166	29	Positive
678.6	Unit	264.3	Unit	20	166	29	Positive
508.5	Unit	262.3	Unit	20	166	23	Positive
536.5	Unit	262.3	Unit	20	166	23	Positive
564.6	Unit	262.3	Unit	20	166	23	Positive
592.6	Unit	262.3	Unit	20	166	23	Positive
606.6	Unit	262.3	Unit	20	166	23	Positive
620.6	Unit	262.3	Unit	20	166	23	Positive
634.6	Unit	262.3	Unit	20	166	23	Positive
648.6	Unit	262.3	Unit	20	166	23	Positive
646.6	Unit	262.3	Unit	20	166	23	Positive
676.6	Unit	262.3	Unit	20	166	23	Positive
650.6	Unit	292.3	Unit	20	166	29	Positive
664.6	Unit	292.3	Unit	20	166	29	Positive
678.6	Unit	292.3	Unit	20	166	29	Positive
676.6	Unit	292.3	Unit	20	166	29	Positive
706.6	Unit	292.3	Unit	20	166	29	Positive
618.4	Unit	264.3	Unit	20	166	29	Positive

646.6	Unit	264.3	Unit	20	166	29	Positive
440.4	Unit	264.3	Unit	20	166	29	Positive
484.5	Unit	264.3	Unit	20	166	29	Positive
496.5	Unit	264.3	Unit	20	166	29	Positive
512.5	Unit	264.3	Unit	20	166	29	Positive
524.5	Unit	264.3	Unit	20	166	29	Positive
540.5	Unit	264.3	Unit	20	166	29	Positive
538.5	Unit	264.3	Unit	20	166	29	Positive
568.6	Unit	264.3	Unit	20	166	29	Positive
524.5	Unit	264.3	Unit	20	166	29	Positive
552.6	Unit	264.3	Unit	20	166	29	Positive
582.5	Unit	264.3	Unit	20	166	29	Positive
610.6	Unit	264.3	Unit	20	166	29	Positive
369.4	Unit	161.2	Unit	20	166	23	Positive
640.8	Unit	367.4	Unit	20	166	12	Positive
666.8	Unit	367.4	Unit	20	166	12	Positive
664.8	Unit	367.4	Unit	20	166	12	Positive
688.8	Unit	367.4	Unit	20	166	12	Positive
686.8	Unit	367.4	Unit	20	166	12	Positive
712.8	Unit	367.4	Unit	20	166	12	Positive
558.5	Unit	285.2	Unit	20	166	21	Positive
558.5	Unit	313.3	Unit	20	166	21	Positive
582.5	Unit	285.2	Unit	20	166	21	Positive
582.5	Unit	337.3	Unit	20	166	21	Positive
586.5	Unit	313.2	Unit	20	166	21	Positive
584.5	Unit	313.2	Unit	20	166	21	Positive
584.5	Unit	311.3	Unit	20	166	21	Positive
612.6	Unit	313.3	Unit	20	166	21	Positive
612.6	Unit	339.3	Unit	20	166	21	Positive
610.5	Unit	313.2	Unit	20	166	21	Positive
610.5	Unit	337.3	Unit	20	166	21	Positive
634.5	Unit	313.2	Unit	20	166	21	Positive
634.5	Unit	361.3	Unit	20	166	21	Positive
660.6	Unit	313.3	Unit	20	166	21	Positive
660.6	Unit	387.3	Unit	20	166	21	Positive
658.5	Unit	313.2	Unit	20	166	21	Positive
658.5	Unit	385.3	Unit	20	166	21	Positive
610.5	Unit	339.2	Unit	20	166	21	Positive

610.5	Unit	311.3	Unit	20	166	21	Positive
640.6	Unit	341.3	Unit	20	166	21	Positive
640.6	Unit	339.2	Unit	20	166	21	Positive
638.6	Unit	341.3	Unit	20	166	21	Positive
638.6	Unit	337.3	Unit	20	166	21	Positive
662.6	Unit	341.3	Unit	20	166	21	Positive
662.6	Unit	361.3	Unit	20	166	21	Positive
638.6	Unit	339.3	Unit	20	166	21	Positive
636.6	Unit	339.3	Unit	20	166	21	Positive
636.6	Unit	337.3	Unit	20	166	21	Positive
662.6	Unit	363.2894	Unit	20	166	21	Positive
662.6	Unit	339.3	Unit	20	166	21	Positive

Compound group	Compoun	Compoun	Ion speci	CAS	z	Monoisot	ISTD	Precurso	MS1 res	Product r	MS2 res
SQDG_NEG	SQDG 16:0/16:0		[M-H]-		1	794.5	FALSE	793.5	Unit	81	Unit
SQDG_NEG	SQDG 16:0/16:0		[M-H]-		1	794.5	FALSE	793.5	Unit	225	Unit
SQDG_NEG	SQDG 16:0/16:1		[M-H]-		1	792.5	FALSE	791.5	Unit	81	Unit
SQDG_NEG	SQDG 16:0/16:1		[M-H]-		1	792.5	FALSE	791.5	Unit	225	Unit
SQDGNH4	SQDG 16:0/16:1		[M+H]+		1	809.5	FALSE	810.5	Unit	313.3	Unit
SQDGNH4	SQDG 16:0/16:1		[M+H]+		1	809.5	FALSE	810.5	Unit	311.3	Unit
SQDG_NEG	SQDG 16:0/18:0		[M-H]-		1	822.5	FALSE	821.5	Unit	81	Unit
SQDG_NEG	SQDG 16:0/18:0		[M-H]-		1	822.5	FALSE	821.5	Unit	225	Unit
SQDGNH4	SQDG 16:0/18:0		[M+H]+		1	839.6	FALSE	840.6	Unit	313.3	Unit
SQDGNH4	SQDG 16:0/18:0		[M+H]+		1	839.6	FALSE	840.6	Unit	341.3	Unit
SQDG_NEG	SQDG 16:0/18:1		[M-H]-		1	820.5	FALSE	819.5	Unit	81	Unit
SQDG_NEG	SQDG 16:0/18:1		[M-H]-		1	820.5	FALSE	819.5	Unit	225	Unit
SQDGNH4	SQDG 16:0/18:1		[M+H]+		1	837.6	FALSE	838.6	Unit	313.3	Unit
SQDGNH4	SQDG 16:0/18:1		[M+H]+		1	837.6	FALSE	838.6	Unit	339.3	Unit
SQDG_NEG	SQDG 16:0/18:2		[M-H]-		1	818.5	FALSE	817.5	Unit	81	Unit
SQDG_NEG	SQDG 16:0/18:2		[M-H]-		1	818.5	FALSE	817.5	Unit	225	Unit
SQDGNH4	SQDG 16:0/18:2		[M+H]+		1	836.6	FALSE	837.6	Unit	313.3	Unit
SQDGNH4	SQDG 16:0/18:2		[M+H]+		1	836.6	FALSE	837.6	Unit	337.3	Unit
SQDG_NEG	SQDG 16:0/18:3		[M-H]-		1	816.5	FALSE	815.5	Unit	81	Unit
SQDG_NEG	SQDG 16:0/18:3		[M-H]-		1	816.5	FALSE	815.5	Unit	225	Unit
SQDGNH4	SQDG 16:0/18:3		[M+H]+		1	833.5	FALSE	834.5	Unit	313.3	Unit
SQDGNH4	SQDG 16:0/18:3		[M+H]+		1	833.5	FALSE	834.5	Unit	335.3	Unit
SQDG_NEG	SQDG 16:0/22:6		[M-H]-		1	866.5	FALSE	865.5	Unit	81	Unit
SQDG_NEG	SQDG 16:0/22:6		[M-H]-		1	866.5	FALSE	865.5	Unit	225	Unit
SQDGNH4	SQDG 16:0/22:6		[M+H]+		1	883.6	FALSE	884.6	Unit	313.3	Unit
SQDGNH4	SQDG 16:0/22:6		[M+H]+		1	883.6	FALSE	884.6	Unit	385.3	Unit
SQDG_NEG	SQDG 16:1/16:1		[M-H]-		1	790.5	FALSE	789.5	Unit	81	Unit
SQDG_NEG	SQDG 16:1/16:1		[M-H]-		1	790.5	FALSE	789.5	Unit	225	Unit
SQDG_NEG	SQDG 16:1/18:1		[M-H]-		1	818.5	FALSE	817.5	Unit	81	Unit
SQDG_NEG	SQDG 16:1/18:1		[M-H]-		1	818.5	FALSE	817.5	Unit	225	Unit
SQDGNH4	SQDG 16:1/18:1		[M+H]+		1	835.6	FALSE	836.6	Unit	311.3	Unit
SQDGNH4	SQDG 16:1/18:1		[M+H]+		1	835.6	FALSE	836.6	Unit	339.3	Unit
SQDG_NEG	SQDG 16:1/18:2		[M-H]-		1	816.5	FALSE	815.5	Unit	81	Unit
SQDG_NEG	SQDG 16:1/18:2		[M-H]-		1	816.5	FALSE	815.5	Unit	225	Unit
SQDGNH4	SQDG 16:1/18:2		[M+H]+		1	833.5	FALSE	834.5	Unit	311.3	Unit
SQDGNH4	SQDG 16:1/18:2		[M+H]+		1	833.5	FALSE	834.5	Unit	337.3	Unit
SQDG_NEG	SQDG 16:1/20:3		[M-H]-		1	842.5	FALSE	841.5	Unit	81	Unit

SQDG_NEG	SQDG 16:1/20:3	[M-H]-	1	842.5	FALSE	841.5	Unit	225	Unit
SQDGNH4	SQDG 16:1/20:3	[M+H]+	1	859.6	FALSE	860.6	Unit	311.3	Unit
SQDGNH4	SQDG 16:1/20:3	[M+H]+	1	859.6	FALSE	860.6	Unit	363.3	Unit
SQDG_NEG	SQDG 16:1/22:6	[M-H]-	1	864.5	FALSE	863.5	Unit	81	Unit
SQDG_NEG	SQDG 16:1/22:6	[M-H]-	1	864.5	FALSE	863.5	Unit	225	Unit
SQDGNH4	SQDG 16:1/22:6	[M+H]+	1	881.5	FALSE	882.5	Unit	311.3	Unit
SQDGNH4	SQDG 16:1/22:6	[M+H]+	1	881.5	FALSE	882.5	Unit	385.3	Unit
SQDG_NEG	SQDG 18:0/18:0	[M-H]-	1	850.6	FALSE	849.6	Unit	81	Unit
SQDG_NEG	SQDG 18:0/18:0	[M-H]-	1	850.6	FALSE	849.6	Unit	225	Unit
SQDG_NEG	SQDG 18:0/18:1	[M-H]-	1	848.6	FALSE	847.6	Unit	81	Unit
SQDG_NEG	SQDG 18:0/18:1	[M-H]-	1	848.6	FALSE	847.6	Unit	225	Unit
SQDGNH4	SQDG 18:1/18:1	[M+H]+	1	863.6	FALSE	864.6	Unit	339.3	Unit
SQDG_NEG	SQDG 18:1/18:2	[M-H]-	1	844.5	FALSE	843.5	Unit	81	Unit
SQDG_NEG	SQDG 18:1/18:2	[M-H]-	1	844.5	FALSE	843.5	Unit	225	Unit
SQDG_NEG	SQDG 18:1/20:0	[M-H]-	1	876.6	FALSE	875.6	Unit	81	Unit
SQDG_NEG	SQDG 18:1/20:0	[M-H]-	1	876.6	FALSE	875.6	Unit	225	Unit
SQDGNH4	SQDG 18:1/20:0	[M+H]+	1	893.6	FALSE	894.6	Unit	339.3	Unit
SQDGNH4	SQDG 18:1/20:0	[M+H]+	1	893.6	FALSE	894.6	Unit	369.3	Unit
SQDG_NEG	SQDG 18:1/20:1	[M-H]-	1	874.6	FALSE	873.6	Unit	81	Unit
SQDG_NEG	SQDG 18:1/20:1	[M-H]-	1	874.6	FALSE	873.6	Unit	225	Unit
SQDGNH4	SQDG 18:1/20:1	[M+H]+	1	891.6	FALSE	892.6	Unit	339.3	Unit
SQDGNH4	SQDG 18:1/20:1	[M+H]+	1	891.6	FALSE	892.6	Unit	367.3	Unit
SQDG_NEG	SQDG 18:1/20:2	[M-H]-	1	872.6	FALSE	871.6	Unit	81	Unit
SQDG_NEG	SQDG 18:1/20:2	[M-H]-	1	872.6	FALSE	871.6	Unit	225	Unit
SQDGNH4	SQDG 18:1/20:2	[M+H]+	1	889.6	FALSE	890.6	Unit	339.3	Unit
SQDGNH4	SQDG 18:1/20:2	[M+H]+	1	889.6	FALSE	890.6	Unit	365.3	Unit
SQDG_NEG	SQDG 18:1/20:3	[M-H]-	1	870.5	FALSE	869.5	Unit	81	Unit
SQDG_NEG	SQDG 18:1/20:3	[M-H]-	1	870.5	FALSE	869.5	Unit	225	Unit
SQDGNH4	SQDG 18:1/20:3	[M+H]+	1	887.6	FALSE	888.6	Unit	339.3	Unit
SQDGNH4	SQDG 18:1/20:3	[M+H]+	1	887.6	FALSE	888.6	Unit	363.3	Unit
SQDG_NEG	SQDG 18:1/20:4	[M-H]-	1	868.5	FALSE	867.5	Unit	81	Unit
SQDG_NEG	SQDG 18:1/20:4	[M-H]-	1	868.5	FALSE	867.5	Unit	225	Unit
SQDGNH4	SQDG 18:1/20:4	[M+H]+	1	885.6	FALSE	886.6	Unit	339.3	Unit
SQDGNH4	SQDG 18:1/20:4	[M+H]+	1	885.6	FALSE	886.6	Unit	361.3	Unit
SQDG_NEG	SQDG 18:1/22:1	[M-H]-	1	902.6	FALSE	901.6	Unit	81	Unit
SQDG_NEG	SQDG 18:1/22:1	[M-H]-	1	902.6	FALSE	901.6	Unit	225	Unit
SQDGNH4	SQDG 18:1/22:1	[M+H]+	1	919.6	FALSE	920.6	Unit	339.3	Unit
SQDGNH4	SQDG 18:1/22:1	[M+H]+	1	919.6	FALSE	920.6	Unit	395.3	Unit

SQDG_NEG	SQDG 18:1/22:2	[M-H]-	1	900.6	FALSE	899.6	Unit	81	Unit
SQDG_NEG	SQDG 18:1/22:2	[M-H]-	1	900.6	FALSE	899.6	Unit	225	Unit
SQDGNH4	SQDG 18:1/22:2	[M+H]+	1	917.6	FALSE	918.6	Unit	339.3	Unit
SQDGNH4	SQDG 18:1/22:2	[M+H]+	1	917.6	FALSE	918.6	Unit	393.3	Unit
SQDG_NEG	SQDG 18:1/22:3	[M-H]-	1	898.6	FALSE	897.6	Unit	81	Unit
SQDG_NEG	SQDG 18:1/22:3	[M-H]-	1	898.6	FALSE	897.6	Unit	225	Unit
SQDGNH4	SQDG 18:1/22:3	[M+H]+	1	915.6	FALSE	916.6	Unit	339.3	Unit
SQDGNH4	SQDG 18:1/22:3	[M+H]+	1	915.6	FALSE	916.6	Unit	391.3	Unit
SQDGNH4	SQDG 18:1/22:4	[M+H]+	1	913.6	FALSE	914.6	Unit	339.3	Unit
SQDGNH4	SQDG 18:1/22:4	[M+H]+	1	913.6	FALSE	914.6	Unit	389.3	Unit
SQDG_NEG	SQDG 18:1/22:5	[M-H]-	1	894.5	FALSE	893.5	Unit	81	Unit
SQDG_NEG	SQDG 18:1/22:5	[M-H]-	1	894.5	FALSE	893.5	Unit	225	Unit
SQDGNH4	SQDG 18:1/22:5	[M+H]+	1	911.6	FALSE	912.6	Unit	339.3	Unit
SQDGNH4	SQDG 18:1/22:5	[M+H]+	1	911.6	FALSE	912.6	Unit	387.3	Unit
SQDG_NEG	SQDG 18:1/22:6	[M-H]-	1	892.5	FALSE	891.5	Unit	81	Unit
SQDG_NEG	SQDG 18:1/22:6	[M-H]-	1	892.5	FALSE	891.5	Unit	225	Unit
SQDGNH4	SQDG 18:1/22:6	[M+H]+	1	909.6	FALSE	910.6	Unit	339.3	Unit
SQDGNH4	SQDG 18:1/22:6	[M+H]+	1	909.6	FALSE	910.6	Unit	385.3	Unit
SQDG_NEG	SQDG 18:2/18:2	[M-H]-	1	842.5	FALSE	841.5	Unit	81	Unit
SQDG_NEG	SQDG 18:2/18:2	[M-H]-	1	842.5	FALSE	841.5	Unit	225	Unit
SQDGNH4	SQDG 18:2/18:2	[M+H]+	1	859.6	FALSE	860.6	Unit	337.3	Unit
SQDG_NEG	SQDG 18:2/20:3	[M-H]-	1	868.5	FALSE	867.5	Unit	81	Unit
SQDG_NEG	SQDG 18:2/20:3	[M-H]-	1	868.5	FALSE	867.5	Unit	225	Unit
SQDGNH4	SQDG 18:2/20:3	[M+H]+	1	885.6	FALSE	886.6	Unit	337.3	Unit
SQDGNH4	SQDG 18:2/20:3	[M+H]+	1	885.6	FALSE	886.6	Unit	363.3	Unit
SQDG_NEG	SQDG 18:2/22:6	[M-H]-	1	890.5	FALSE	889.5	Unit	81	Unit
SQDG_NEG	SQDG 18:2/22:6	[M-H]-	1	890.5	FALSE	889.5	Unit	225	Unit
SQDGNH4	SQDG 18:2/22:6	[M+H]+	1	907.6	FALSE	908.6	Unit	337.3	Unit
SQDGNH4	SQDG 18:2/22:6	[M+H]+	1	907.6	FALSE	908.6	Unit	385.3	Unit

Dwell (m	Fragmen	CE (V)	Polarity
5	250	60	Negative
5	250	60	Negative
5	250	60	Negative
5	250	60	Negative
5	185	26	Positive
5	185	26	Positive
5	250	60	Negative
5	250	60	Negative
5	185	26	Positive
5	185	26	Positive
5	250	60	Negative
5	250	60	Negative
5	185	26	Positive
5	185	26	Positive
5	250	60	Negative
5	250	60	Negative
5	185	26	Positive
5	185	26	Positive
5	250	60	Negative
5	250	60	Negative
5	185	26	Positive
5	185	26	Positive
5	250	60	Negative
5	250	60	Negative
5	185	26	Positive
5	185	26	Positive
5	250	60	Negative
5	250	60	Negative
5	250	60	Negative
5	250	60	Negative
5	185	26	Positive
5	185	26	Positive
5	250	60	Negative
5	250	60	Negative
5	185	26	Positive
5	185	26	Positive
5	250	60	Negative

5	250	60	Negative
5	185	26	Positive
5	185	26	Positive
5	250	60	Negative
5	250	60	Negative
5	185	26	Positive
5	185	26	Positive
5	250	60	Negative
5	250	60	Negative
5	250	60	Negative
5	250	60	Negative
5	185	26	Positive
5	250	60	Negative
5	250	60	Negative
5	250	60	Negative
5	250	60	Negative
5	185	26	Positive
5	185	26	Positive
5	250	60	Negative
5	250	60	Negative
5	185	26	Positive
5	185	26	Positive
5	250	60	Negative
5	250	60	Negative
5	185	26	Positive
5	185	26	Positive
5	250	60	Negative
5	250	60	Negative
5	185	26	Positive
5	185	26	Positive
5	250	60	Negative
5	250	60	Negative
5	185	26	Positive
5	185	26	Positive
5	250	60	Negative
5	250	60	Negative
5	185	26	Positive
5	185	26	Positive

5	250	60	Negative
5	250	60	Negative
5	185	26	Positive
5	185	26	Positive
5	250	60	Negative
5	250	60	Negative
5	185	26	Positive
5	185	26	Positive
5	185	26	Positive
5	185	26	Positive
5	250	60	Negative
5	250	60	Negative
5	185	26	Positive
5	185	26	Positive
5	250	60	Negative
5	250	60	Negative
5	185	26	Positive
5	185	26	Positive
5	250	60	Negative
5	250	60	Negative
5	185	26	Positive
5	250	60	Negative
5	250	60	Negative
5	185	26	Positive
5	185	26	Positive
5	250	60	Negative
5	250	60	Negative
5	185	26	Positive
5	185	26	Positive

Compound group	Compound name	Compound formula	Ion speci	CAS	z	Monoisot	ISTD	Precurso
DG(18:2_20:4)	DG(18:2_20:4)	C42H82NO10P	[M+NH4]+		1		FALSE	658.5
MGDG(16:0_16:1)a	MGDG(16:0_16:1)a	C41H77O10	[M+H]+		1		FALSE	729.5
MGDG(16:0_16:1)b	MGDG(16:0_16:1)b	C41H77O10	[M+NH4]+		1		FALSE	746.6
MGDG(18:0/18:0)a	MGDG(18:0/18:0)a		[M+NH4]+		1		FALSE	760.6
MGDG(18:0/18:0)b	MGDG(18:0/18:0)b		[M+H]+		1		FALSE	743.6
BisMeLPA(12:0e)a	BisMeLPA(12:0e)a		[M+H]+		1		FALSE	369.2
BisMeLPA(12:0e)b	BisMeLPA(12:0e)b		[M+H]+		1		FALSE	369.2
CarE(14:0+O)a	CarE(14:0+O)a	C21 H41 N O5	[M+H]+		1		FALSE	388.3
CarE(16:1)b	CarE(16:1)b	C23 H43 N O4	[M+H]+		1		FALSE	398.3
dhCer(d18:0/16:0)	dhCer(d18:0/16:0)		[M+H]+		1		FALSE	540.5
dhCer(d18:0/18:0)	dhCer(d18:0/18:0)		[M+H]+		1		FALSE	568.6
dhCer(d18:0/20:0)	dhCer(d18:0/20:0)		[M+H]+		1		FALSE	596.6
dhCer(d18:0/22:0)	dhCer(d18:0/22:0)		[M+H]+		1		FALSE	624.6
dhCer(d18:0/24:0)	dhCer(d18:0/24:0)		[M+H]+		1		FALSE	652.7
dhCer(d18:0/24:1)	dhCer(d18:0/24:1)		[M+H]+		1		FALSE	650.6
dhCer(d18:1/18:0)	dhCer(d18:1/18:0)		[M+H]+		1		FALSE	566.6
dhCer(d18:1/20:0)	dhCer(d18:1/20:0)		[M+H]+		1		FALSE	594.6
dhCer(d18:1/22:0)	dhCer(d18:1/22:0)		[M+H]+		1		FALSE	622.6
dhCer(d18:1/24:0)	dhCer(d18:1/24:0)		[M+H]+		1		FALSE	650.7
dhCer(d18:1/24:1)	dhCer(d18:1/24:1)		[M+H]+		1		FALSE	648.6
dhCer(d18:1/24:2)	dhCer(d18:1/24:2)		[M+H]+		1		FALSE	646.6
Cer(d18:1_26:0)	Cer(d18:1_26:0)		[M+H]+		1		FALSE	678.7
Cer(d18:2_24:0)	Cer(d18:2_24:0)		[M+H]+		1		FALSE	648.6
Cer(d18:2_28:0)	Cer(d18:2_28:0)		[M+H]+		1		FALSE	704.7
Cer(d19:2_31:0+O)	Cer(d19:2_31:0+O)		[M+H]+		1		FALSE	760.8
Cer(d20:2_26:0+O)	Cer(d20:2_26:0+O)		[M+H]+		1		FALSE	704.7
Cer(t18:0_26:0)	Cer(t18:0_26:0)		[M+H]+		1		FALSE	696.7
Cer(t20:0_25:0+O)	Cer(t20:0_25:0+O)		[M+H]+		1		FALSE	710.7
GM1(d18:1/16:0)	GM1(d18:1/16:0)		[M+2H]2+		1		FALSE	760.1
GM3(d18:1/16:0)	GM3(d18:1/16:0)		[M+H]+		1		FALSE	1153.7
GM3(d18:1/18:0)	GM3(d18:1/18:0)		[M+H]+		1		FALSE	1181.8
GM3(d18:1/20:0)	GM3(d18:1/20:0)		[M+H]+		1		FALSE	1209.8
GM3(d18:1/22:0)	GM3(d18:1/22:0)		[M+H]+		1		FALSE	1237.8
GM3(d18:1/24:0)	GM3(d18:1/24:0)		[M+H]+		1		FALSE	1265.8
GM3(d18:1/24:1)	GM3(d18:1/24:1)		[M+H]+		1		FALSE	1263.8
GM3(d18:2/24:1)	GM3(d18:2/24:1)		[M+H]+		1		FALSE	1261.8
Hex2Cer(d18:1/16:0)	Hex2Cer(d18:1/16:0)		[M+H]+		1		FALSE	862.6

Hex2Cer(d18:1/18:0	Hex2Cer(d18:1/18:0)		[M+H]+	1	FALSE	890.7
Hex2Cer(d18:1/20:0	Hex2Cer(d18:1/20:0)		[M+H]+	1	FALSE	918.7
Hex2Cer(d18:1/22:0	Hex2Cer(d18:1/22:0)		[M+H]+	1	FALSE	946.7
Hex2Cer(d18:1/24:0	Hex2Cer(d18:1/24:0)		[M+H]+	1	FALSE	974.8
Hex2Cer(d18:1/24:1	Hex2Cer(d18:1/24:1)		[M+H]+	1	FALSE	972.7
Hex3Cer(d18:1/16:0	Hex3Cer(d18:1/16:0)		[M+H]+	1	FALSE	1024.7
Hex3Cer(d18:1/18:0	Hex3Cer(d18:1/18:0)		[M+H]+	1	FALSE	1052.7
Hex3Cer(d18:1/20:0	Hex3Cer(d18:1/20:0)		[M+H]+	1	FALSE	1080.7
Hex3Cer(d18:1/22:0	Hex3Cer(d18:1/22:0)		[M+H]+	1	FALSE	1108.8
Hex3Cer(d18:1/24:0	Hex3Cer(d18:1/24:0)		[M+H]+	1	FALSE	1136.8
Hex3Cer(d18:1/24:1	Hex3Cer(d18:1/24:1)		[M+H]+	1	FALSE	1134.8
HexCer(d16:1/18:0)	HexCer(d16:1/18:0)		[M+H]+	1	FALSE	700.6
HexCer(d16:1/20:0)	HexCer(d16:1/20:0)		[M+H]+	1	FALSE	728.6
HexCer(d16:1/22:0)	HexCer(d16:1/22:0)		[M+H]+	1	FALSE	756.7
HexCer(d16:1/24:0)	HexCer(d16:1/24:0)		[M+H]+	1	FALSE	784.7
HexCer(d18:1/16:0)	HexCer(d18:1/16:0)		[M+H]+	1	FALSE	700.6
HexCer(d18:1/18:0)	HexCer(d18:1/18:0)		[M+H]+	1	FALSE	728.6
HexCer(d18:1/20:0)	HexCer(d18:1/20:0)		[M+H]+	1	FALSE	756.6
HexCer(d18:1/22:0)	HexCer(d18:1/22:0)		[M+H]+	1	FALSE	784.7
HexCer(d18:1/24:0)	HexCer(d18:1/24:0)		[M+H]+	1	FALSE	812.7
HexCer(d18:1/24:1)	HexCer(d18:1/24:1)		[M+H]+	1	FALSE	810.7
HexCer(d18:2/18:0)	HexCer(d18:2/18:0)		[M+H]+	1	FALSE	726.6
HexCer(d18:2/20:0)	HexCer(d18:2/20:0)		[M+H]+	1	FALSE	754.6
HexCer(d18:2/22:0)	HexCer(d18:2/22:0)		[M+H]+	1	FALSE	782.7
HexCer(d18:2/24:0)	HexCer(d18:2/24:0)		[M+H]+	1	FALSE	810.7
LPC(12:0)	LPC(12:0)	C20H46N2O7P	[M+H]+	1	FALSE	457.3
LPC(14:0) [sn1]	LPC(14:0) [sn1]	C42H82NO10P	[M+H]+	1	FALSE	468.3
LPC(15:0) [sn1]	LPC(15:0) [sn1]	C42H82NO10P	[M+H]+	1	FALSE	482.3
LPC(15-MHDA) [sn1	LPC(15-MHDA) [sn1]	C42H82NO10P	[M+H]+	1	FALSE	510.4
LPC(15-MHDA) [sn1	LPC(15-MHDA) [sn1]	C42H82NO10P	[M+H]+	1	FALSE	510.4
LPC(15-MHDA) [sn2	LPC(15-MHDA) [sn2]	C42H82NO10P	[M+H]+	1	FALSE	510.4
LPC(16:0) [sn1]	LPC(16:0) [sn1]	C42H82NO10P	[M+H]+	1	FALSE	496.3
LPC(16:1) [sn1]	LPC(16:1) [sn1]	C42H82NO10P	[M+H]+	1	FALSE	494.3
LPC(16:2) [sn1]	LPC(16:2) [sn1]	C42H82NO10P	[M+H]+	1	FALSE	492.3
LPC(18:0) [sn1]	LPC(18:0) [sn1]	C42H82NO10P	[M+H]+	1	FALSE	524.4
LPC(18:1) [sn1]	LPC(18:1) [sn1]	C42H82NO10P	[M+H]+	1	FALSE	522.4
LPC(18:2) [sn1]	LPC(18:2) [sn1]	C42H82NO10P	[M+H]+	1	FALSE	520.3
LPC(18:3) [sn1] (b)	LPC(18:3) [sn1] (b)	C42H82NO10P	[M+H]+	1	FALSE	518.3

LPC(18:3) [sn2] (a)	LPC(18:3) [sn2] (a)	C42H82NO10P	[M+H]+	1	FALSE	518.3
LPC(18:4) [sn2] (a)	LPC(18:4) [sn2] (a)	C42H82NO10P	[M+H]+	1	FALSE	516.3
LPC(20:0) [sn1]	LPC(20:0) [sn1]	C42H82NO10P	[M+H]+	1	FALSE	552.4
LPC(20:1) [sn1]	LPC(20:1) [sn1]	C42H82NO10P	[M+H]+	1	FALSE	550.4
LPC(20:2) [sn1]	LPC(20:2) [sn1]	C42H82NO10P	[M+H]+	1	FALSE	548.4
LPC(20:3) [sn2]	LPC(20:3) [sn2]	C42H82NO10P	[M+H]+	1	FALSE	546.4
LPC(20:4) [sn1]	LPC(20:4) [sn1]	C42H82NO10P	[M+H]+	1	FALSE	544.3
LPC(20:4) [sn2]	LPC(20:4) [sn2]	C42H82NO10P	[M+H]+	1	FALSE	544.3
LPC(20:5) [sn1]	LPC(20:5) [sn1]	C42H82NO10P	[M+H]+	1	FALSE	542.3
LPC(22:0) [sn1]	LPC(22:0) [sn1]	C42H82NO10P	[M+H]+	1	FALSE	580.4
LPC(22:1) [sn1]	LPC(22:1) [sn1]	C42H82NO10P	[M+H]+	1	FALSE	578.4
LPC(22:4) [sn1]	LPC(22:4) [sn1]	C42H82NO10P	[M+H]+	1	FALSE	572.4
LPC(22:5) (n3) [sn1]	LPC(22:5) (n3) [sn1] [	C42H82NO10P	[M+H]+	1	FALSE	570.4
LPC(22:5) [sn1] (n3)/	LPC(22:5) [sn1] (n3)/L	C42H82NO10P	[M+H]+	1	FALSE	570.4
LPC(22:5) [sn1] (n6)	LPC(22:5) [sn1] (n6)	C42H82NO10P	[M+H]+	1	FALSE	570.4
LPC(22:5) [sn2] (n3)	LPC(22:5) [sn2] (n3)	C42H82NO10P	[M+H]+	1	FALSE	570.4
LPC(22:6) [sn1]	LPC(22:6) [sn1]	C42H82NO10P	[M+H]+	1	FALSE	568.3
LPC(24:0) [sn1]	LPC(24:0) [sn1]	C42H82NO10P	[M+H]+	1	FALSE	608.5
LPC(26:0) [sn1]	LPC(26:0) [sn1]	C42H82NO10P	[M+H]+	1	FALSE	636.5
LPC(32:1) [sn1]	LPC(32:1) [sn1]	C42H82NO10P	[M+H]+	1	FALSE	718.6
LPC(O-16:0)	LPC(O-16:0)	C42H82NO10P	[M+H]+	1	FALSE	482.4
LPC(O-18:0)	LPC(O-18:0)	C42H82NO10P	[M+H]+	1	FALSE	510.4
LPC(O-18:1)	LPC(O-18:1)	C42H82NO10P	[M+H]+	1	FALSE	508.4
LPC(O-20:0)	LPC(O-20:0)	C42H82NO10P	[M+H]+	1	FALSE	538.4
LPC(O-20:1)	LPC(O-20:1)	C42H82NO10P	[M+H]+	1	FALSE	536.4
LPC(O-22:0)	LPC(O-22:0)	C42H82NO10P	[M+H]+	1	FALSE	566.5
LPC(O-22:1)	LPC(O-22:1)	C42H82NO10P	[M+H]+	1	FALSE	564.4
LPC(O-24:0)	LPC(O-24:0)	C42H82NO10P	[M+H]+	1	FALSE	594.5
LPC(O-24:1)	LPC(O-24:1)	C42H82NO10P	[M+H]+	1	FALSE	592.5
LPC(O-24:2)	LPC(O-24:2)	C42H82NO10P	[M+H]+	1	FALSE	590.5
LPC(P-16:0)	LPC(P-16:0)	C42H82NO10P	[M+H]+	1	FALSE	480.3
LPC(P-17:0) (a)	LPC(P-17:0) (a)	C42H82NO10P	[M+H]+	1	FALSE	494.3
LPC(P-18:0)	LPC(P-18:0)	C42H82NO10P	[M+H]+	1	FALSE	508.3
LPC(P-18:1)	LPC(P-18:1)	C42H82NO10P	[M+H]+	1	FALSE	506.3
LPC(P-20:0)	LPC(P-20:0)	C42H82NO10P	[M+H]+	1	FALSE	536.3
MePC(29:0)	MePC(29:0)	$C_{38}H_{76}NO_8P$	[M+H]+	1	FALSE	722.5
MePC(32:1)	MePC(32:1)	$C_{41}H_{80}NO_8P$	[M+H]+	1	FALSE	762.6
MePC(35:0)	MePC(35:0)	$C_{44}H_{88}NO_8P$	[M+H]+	1	FALSE	802.6

LPE(16:0) [sn1]	LPE(16:0) [sn1]	C42H82NO10P	[M+H]+	1	FALSE	454.3
LPE(18:0) [sn1]	LPE(18:0) [sn1]	C42H82NO10P	[M+H]+	1	FALSE	482.3
LPE(18:1) [sn1]	LPE(18:1) [sn1]	C42H82NO10P	[M+H]+	1	FALSE	480.3
LPE(18:2) [sn1]	LPE(18:2) [sn1]	C42H82NO10P	[M+H]+	1	FALSE	478.3
LPE(20:4) [sn2]	LPE(20:4) [sn2]	C42H82NO10P	[M+H]+	1	FALSE	502.3
LPE(22:6) [sn2]	LPE(22:6) [sn2]	C42H82NO10P	[M+H]+	1	FALSE	526.3
LPE(P-16:0)	LPE(P-16:0)	C42H82NO10P	[M+H]+	1	FALSE	438.3
LPE(P-18:0)	LPE(P-18:0)	C42H82NO10P	[M+H]+	1	FALSE	466.3
LPE(P-18:1)	LPE(P-18:1)	C42H82NO10P	[M+H]+	1	FALSE	464.3

MS1 res	Product r	MS2 res	Dwell (m	Fragmen	CE (V)	Polarity
Unit	337.2	Unit	20	166	21	Positive
Unit	257.2	Unit	20	166	21	Positive
Unit	313.2	Unit	20	166	21	Positive
Unit	341.3	Unit	20	166	21	Positive
Unit	284.3	Unit	20	166	21	Positive
Unit	153	Unit	20	166	21	Positive
Unit	255.2	Unit	20	166	21	Positive
Unit	85.02	Unit	20	166	21	Positive
Unit	85.02	Unit	20	166	21	Positive
Unit	284.3	Unit	20	166	31	Positive
Unit	284.3	Unit	20	166	31	Positive
Unit	284.3	Unit	20	166	31	Positive
Unit	284.3	Unit	20	166	31	Positive
Unit	284.3	Unit	20	166	31	Positive
Unit	284.3	Unit	20	166	31	Positive
Unit	282.3	Unit	20	166	31	Positive
Unit	282.3	Unit	20	166	31	Positive
Unit	282.3	Unit	20	166	31	Positive
Unit	282.3	Unit	20	166	31	Positive
Unit	282.3	Unit	20	166	31	Positive
Unit	282.3	Unit	20	166	31	Positive
Unit	282.3	Unit	20	166	31	Positive
Unit	280.3	Unit	20	166	31	Positive
Unit	280.3	Unit	20	166	31	Positive
Unit	264.3	Unit	20	166	31	Positive
Unit	264.3	Unit	20	166	31	Positive
Unit	264.3	Unit	20	166	31	Positive
Unit	264.3	Unit	20	166	31	Positive
Unit	366.2	Unit	20	166	31	Positive
Unit	264.3	Unit	20	166	57	Positive
Unit	264.3	Unit	20	166	57	Positive
Unit	264.3	Unit	20	166	57	Positive
Unit	264.3	Unit	20	166	57	Positive
Unit	264.3	Unit	20	166	57	Positive
Unit	264.3	Unit	20	166	57	Positive
Unit	262.3	Unit	20	166	57	Positive
Unit	264.3	Unit	20	166	53	Positive

Unit	264.3	Unit	20	166	53	Positive
Unit	264.3	Unit	20	166	53	Positive
Unit	264.3	Unit	20	166	53	Positive
Unit	264.3	Unit	20	166	53	Positive
Unit	264.3	Unit	20	166	53	Positive
Unit	264.3	Unit	20	166	53	Positive
Unit	264.3	Unit	20	166	53	Positive
Unit	264.3	Unit	20	166	53	Positive
Unit	264.3	Unit	20	166	53	Positive
Unit	264.3	Unit	20	166	53	Positive
Unit	264.3	Unit	20	166	53	Positive
Unit	236.3	Unit	20	166	33	Positive
Unit	236.3	Unit	20	166	33	Positive
Unit	236.3	Unit	20	166	33	Positive
Unit	236.3	Unit	20	166	33	Positive
Unit	264.3	Unit	20	166	33	Positive
Unit	264.3	Unit	20	166	33	Positive
Unit	264.3	Unit	20	166	33	Positive
Unit	264.3	Unit	20	166	33	Positive
Unit	264.3	Unit	20	166	33	Positive
Unit	264.3	Unit	20	166	33	Positive
Unit	262.3	Unit	20	166	33	Positive
Unit	262.3	Unit	20	166	33	Positive
Unit	262.3	Unit	20	166	33	Positive
Unit	262.3	Unit	20	166	33	Positive
Unit	184.1	Unit	20	166	21	Positive
Unit	184.1	Unit	20	166	21	Positive
Unit	184.1	Unit	20	166	21	Positive
Unit	184.1	Unit	20	166	21	Positive
Unit	104.1	Unit	20	166	21	Positive
Unit	184.1	Unit	20	166	21	Positive
Unit	184.1	Unit	20	166	21	Positive
Unit	184.1	Unit	20	166	21	Positive
Unit	184.1	Unit	20	166	21	Positive
Unit	184.1	Unit	20	166	21	Positive
Unit	184.1	Unit	20	166	21	Positive
Unit	184.1	Unit	20	166	21	Positive
Unit	184.1	Unit	20	166	21	Positive

Unit	184.1	Unit	20	166	21	Positive
Unit	184.1	Unit	20	166	21	Positive
Unit	184.1	Unit	20	166	21	Positive
Unit	184.1	Unit	20	166	21	Positive
Unit	184.1	Unit	20	166	21	Positive
Unit	184.1	Unit	20	166	21	Positive
Unit	184.1	Unit	20	166	21	Positive
Unit	184.1	Unit	20	166	21	Positive
Unit	184.1	Unit	20	166	21	Positive
Unit	184.1	Unit	20	166	21	Positive
Unit	184.1	Unit	20	166	21	Positive
Unit	184.1	Unit	20	166	21	Positive
Unit	184.1	Unit	20	166	21	Positive
Unit	184.1	Unit	20	166	21	Positive
Unit	184.1	Unit	20	166	21	Positive
Unit	184.1	Unit	20	166	21	Positive
Unit	184.1	Unit	20	166	21	Positive
Unit	184.1	Unit	20	166	21	Positive
Unit	184.1	Unit	20	166	21	Positive
Unit	184.1	Unit	20	166	21	Positive
Unit	184.1	Unit	20	166	21	Positive
Unit	184.1	Unit	20	166	21	Positive
Unit	184.1	Unit	20	166	21	Positive
Unit	184.1	Unit	20	166	21	Positive
Unit	184.1	Unit	20	166	21	Positive
Unit	184.1	Unit	20	166	21	Positive
Unit	184.1	Unit	20	166	21	Positive
Unit	184.1	Unit	20	166	21	Positive
Unit	184.1	Unit	20	166	21	Positive
Unit	184.1	Unit	20	166	21	Positive
Unit	184.1	Unit	20	166	21	Positive
Unit	184.1	Unit	20	166	21	Positive
Unit	184.1	Unit	20	166	21	Positive
Unit	184.1	Unit	20	166	21	Positive
Unit	184.1	Unit	20	166	21	Positive
Unit	184.1	Unit	20	166	21	Positive
Unit	184.1	Unit	20	166	21	Positive
Unit	184.1	Unit	20	166	21	Positive

Unit	313.3	Unit	20	166	17	Positive
Unit	341.3	Unit	20	166	17	Positive
Unit	339.3	Unit	20	166	17	Positive
Unit	337.3	Unit	20	166	17	Positive
Unit	361.3	Unit	20	166	17	Positive
Unit	385.3	Unit	20	166	17	Positive
Unit	266.4	Unit	20	166	19	Positive
Unit	294.4	Unit	20	166	19	Positive
Unit	292.4	Unit	20	166	19	Positive

label	Compound group	Compoun	Ion speci	CAS	z	Monoisot	ISTD	Precurso
BMPNH4	BMP_14:0/18:1		[M+NH4]+				FALSE	738.6
BMPNH4	BMP_14:0/18:1		[M+NH4]+				FALSE	738.6
BMPNH4	BMP_16:0/16:0		[M+NH4]+				FALSE	740.5
BMPNH4	BMP_16:0/16:1		[M+NH4]+				FALSE	738.5
BMPNH4	BMP_16:0/16:1 dup		[M+NH4]+				FALSE	738.5
BMPNH4	BMP_16:0/18:0		[M+NH4]+				FALSE	768.5
BMPNH4	BMP_16:0/18:0		[M+NH4]+				FALSE	768.5
BMPNH4	BMP_16:0/18:1		[M+NH4]+				FALSE	766.6
BMPNH4	BMP_16:0/18:1		[M+NH4]+				FALSE	766.6
BMPNH4	BMP(16:0/18:2)		[M+NH4]+				FALSE	764.5
BMPNH4	BMP(16:0/18:2)		[M+NH4]+				FALSE	764.5
BMPNH4	BMP_16:0/18:1		[M+NH4]+				FALSE	766.6
BMPNH4	BMP_16:0/22:6		[M+NH4]+				FALSE	812.5
BMPNH4	BMP_16:0/22:6		[M+NH4]+				FALSE	812.5
BMPNH4	BMP_16:1/16:1		[M+NH4]+				FALSE	736.5
BMPNH4	BMP_16:1/18:1		[M+NH4]+				FALSE	764.5
BMPNH4	BMP_16:1/18:1		[M+NH4]+				FALSE	764.5
BMPNH4	BMP_16:1/18:2		[M+NH4]+				FALSE	762.5
BMPNH4	BMP_16:1/20:3		[M+NH4]+				FALSE	788.6
BMPNH4	BMP_16:1/22:6		[M+NH4]+				FALSE	810.5
BMPNH4	BMP_16:1/22:6		[M+NH4]+				FALSE	810.5
BMPNH4	BMP_18:0/18:0		[M+NH4]+				FALSE	796.6
BMPNH4	BMP_18:0/18:1		[M+NH4]+				FALSE	794.6
BMPNH4	BMP_18:0/18:1		[M+NH4]+				FALSE	794.6
BMPNH4	BMP_18:1/18:1		[M+NH4]+				FALSE	792.6
BMPNH4	BMP_18:1/18:2		[M+NH4]+				FALSE	790.6
BMPNH4	BMP_18:1/18:2		[M+NH4]+				FALSE	790.6
BMPNH4	BMP_18:1/20:0		[M+NH4]+				FALSE	822.6
BMPNH4	BMP_18:1/20:1		[M+NH4]+				FALSE	820.6
BMPNH4	BMP_18:1/20:1		[M+NH4]+				FALSE	820.6
BMPNH4	BMP_18:1/20:2		[M+NH4]+				FALSE	818.6
BMPNH4	BMP_18:1/20:3		[M+NH4]+				FALSE	816.6
BMPNH4	BMP_18:1/20:4		[M+NH4]+				FALSE	814.6
BMPNH4	BMP_18:1/22:1		[M+NH4]+				FALSE	848.6
BMPNH4	BMP_18:1/22:2		[M+NH4]+				FALSE	846.6
BMPNH4	BMP_18:1/22:3		[M+NH4]+				FALSE	844.6
BMPNH4	BMP_18:1/22:4		[M+NH4]+				FALSE	842.6

BMPNH4	BMP_18:1/22:5		[M+NH4]+		FALSE	840.6
BMPNH4	BMP_18:1/22:6		[M+NH4]+		FALSE	838.6
BMPNH4	BMP_18:1/22:6		[M+NH4]+		FALSE	838.6
BMPNH4	BMP_18:2/18:2		[M+NH4]+		FALSE	788.5
BMPNH4	BMP_18:2/20:3		[M+NH4]+		FALSE	814.6
BMPNH4	BMP_18:2/22:6		[M+NH4]+		FALSE	836.5
BMPNH4	BMP_18:4/22:6		[M+NH4]+		FALSE	832.5
BMPNH4	BMP_20:4/20:4		[M+NH4]+		FALSE	836.5
BMPNH4	BMP_20:4/22:6		[M+NH4]+		FALSE	860.5
BMPNH4	BMP_20:5/22:6		[M+NH4]+		FALSE	858.5
BMPNH4	BMP_22:5/22:5		[M+NH4]+		FALSE	888.6
BMPNH4	BMP_22:5/22:6		[M+NH4]+		FALSE	886.6
BMPNH4	BMP_22:6/22:6		[M+NH4]+		FALSE	884.5
HemiBMI	HB_16:0/18:1/18:1	C60H11	[M+NH4]+		FALSE	1030.8
HemiBMI	HB_18:0/18:1/18:1	C60H11	[M+NH4]+		FALSE	1058.8
HemiBMI	HB_18:1/18:1/18:1	C60H11	[M+NH4]+	1	FALSE	1056.8
LPG	LPG_16:0		[M-H]-		FALSE	483.3
LPG	LPG_18:0		[M-H]-		FALSE	511.3
LPG	LPG_18:1	C24H47(	[M-H]-	1	FALSE	509.3
LPG	LPG_18:2		[M-H]-		FALSE	507.3
LPG	LPG_20:4		[M-H]-		FALSE	531.3
LPG	LPG_20:5		[M-H]-		FALSE	529.3
LPG	LPG_22:4		[M-H]-		FALSE	559.3
LPG	LPG_22:5		[M-H]-		FALSE	557.3
LPG	LPG_22:6		[M-H]-		FALSE	555.3
PC	PC_18:1		[M+H]+		FALSE	786.6
PG NH4	PG_14:0/16:0		[M+NH4]+		FALSE	712.4
PG NH4	PG_14:0/18:1		[M+NH4]+		FALSE	738.6
PG NH4	PG_16:0/16:0		[M+NH4]+		FALSE	740.5
PG NH4	PG_16:0/16:1		[M+NH4]+		FALSE	738.5
PG NH4	PG_16:0/18:0		[M+NH4]+		FALSE	768.5
PG NH4	PG_16:0/18:1		[M+NH4]+		FALSE	766.6
PG NH4	PG_16:0/20:4 S18:2 and S16:1/20:3		[M+NH4]+		FALSE	788.6
PG NH4	PG_16:0/22:6		[M+NH4]+		FALSE	812.5
PG NH4	PG_16:1/16:1		[M+NH4]+		FALSE	736.5
PG NH4	PG_16:1/18:1		[M+NH4]+		FALSE	764.5
PG NH4	PG_16:1/18:2		[M+NH4]+		FALSE	762.5
PG NH4	PG_16:1/22:6		[M+NH4]+		FALSE	810.5

PG NH4	PG_18:0/18:0	[M+NH4]+	FALSE	796.6
PG NH4	PG_18:0/18:1	[M+NH4]+	FALSE	794.6
PG NH4	PG_18:1/18:1	[M+NH4]+	FALSE	792.6
PG NH4	PG_18:1/18:2	[M+NH4]+	FALSE	790.6
PG NH4	PG_18:1/20:0 S18:0/20:1	[M+NH4]+	FALSE	822.6
PG NH4	PG_18:1/20:1	[M+NH4]+	FALSE	820.6
PG NH4	PG_18:1/20:2	[M+NH4]+	FALSE	818.6
PG NH4	PG_18:1/20:3	[M+NH4]+	FALSE	816.6
PG NH4	PG_18:1/20:4 S18:2/20:3	[M+NH4]+	FALSE	814.6
PG NH4	PG_18:1/22:1	[M+NH4]+	FALSE	848.6
PG NH4	PG_18:1/22:2	[M+NH4]+	FALSE	846.6
PG NH4	PG_18:1/22:3	[M+NH4]+	FALSE	844.6
PG NH4	PG_18:1/22:4	[M+NH4]+	FALSE	842.6
PG NH4	PG_18:1/22:5	[M+NH4]+	FALSE	840.6
PG NH4	PG_18:1/22:6	[M+NH4]+	FALSE	838.6
PG NH4	PG_18:2/22:6	[M+NH4]+	FALSE	836.5
PG NH4	PG_18:4/22:6	[M+NH4]+	FALSE	832.5
PG NH4	PG_20:4/22:6	[M+NH4]+	FALSE	860.5
PG NH4	PG_20:5/22:6	[M+NH4]+	FALSE	858.5
PG NH4	PG_22:4/22:6/ S22:5	[M+NH4]+	FALSE	888.6
PG NH4	PG_22:5/22:6	[M+NH4]+	FALSE	886.6
PG NH4	PG_22:6/22:6	[M+NH4]+	FALSE	884.5
PC	POPC	[M+H]+	FALSE	760.6
PC	POPC	[M+H]+	FALSE	760.6

MS1 res	Product r	MS2 res	Dwell (m	Fragmen	CE (V)	Polarity
Unit	285.3	Unit	5	150	27	Positive
Unit	339.3	Unit	5	150	27	Positive
Unit	313.5	Unit	5	150	27	Positive
Unit	311.5	Unit	5	150	27	Positive
Unit	313.5	Unit	5	150	27	Positive
Unit	313.5	Unit	5	150	27	Positive
Unit	341.3	Unit	5	150	27	Positive
Unit	313.5	Unit	5	150	27	Positive
Unit	339.3	Unit	5	150	27	Positive
Unit	313.5	Unit	5	150	27	Positive
Unit	337.3	Unit	5	150	27	Positive
Unit	339.3	Unit	5	150	27	Positive
Unit	313.5	Unit	5	150	27	Positive
Unit	385.3	Unit	5	150	27	Positive
Unit	311.5	Unit	5	150	27	Positive
Unit	311.5	Unit	5	150	27	Positive
Unit	339.3	Unit	5	150	27	Positive
Unit	337.3	Unit	5	150	27	Positive
Unit	311.3	Unit	5	150	27	Positive
Unit	311.3	Unit	5	150	27	Positive
Unit	385.3	Unit	5	150	27	Positive
Unit	341.3	Unit	5	150	27	Positive
Unit	339.3	Unit	5	150	27	Positive
Unit	341.3	Unit	5	150	27	Positive
Unit	339.3	Unit	5	150	27	Positive
Unit	337.3	Unit	5	150	27	Positive
Unit	339.3	Unit	5	150	27	Positive
Unit	339.3	Unit	5	150	27	Positive
Unit	339.3	Unit	5	150	27	Positive
Unit	367.3	Unit	5	150	27	Positive
Unit	339.3	Unit	5	150	27	Positive
Unit	363.3	Unit	5	150	27	Positive
Unit	361.3	Unit	5	150	27	Positive
Unit	339.3	Unit	5	150	27	Positive
Unit	339.3	Unit	5	150	27	Positive
Unit	339.3	Unit	5	150	27	Positive
Unit	339.3	Unit	5	150	27	Positive

Unit	339.3	Unit	5	150	27	Positive
Unit	339.3	Unit	5	150	27	Positive
Unit	385.3	Unit	5	150	27	Positive
Unit	337.3	Unit	5	150	27	Positive
Unit	363.3	Unit	5	150	27	Positive
Unit	385.3	Unit	5	150	27	Positive
Unit	385.6	Unit	5	150	27	Positive
Unit	357.3	Unit	5	150	27	Positive
Unit	385.3	Unit	5	150	27	Positive
Unit	385.3	Unit	5	150	27	Positive
Unit	387.3	Unit	5	150	27	Positive
Unit	387.3	Unit	5	150	27	Positive
Unit	385.3	Unit	5	150	27	Positive
Unit	577.5	Unit	5	143	27	Positive
Unit	605.5	Unit	5	143	27	Positive
Unit	603.5	Unit	5	143	27	Positive
Unit	255.2	Unit	5	163	32	Negative
Unit	283.2	Unit	5	163	32	Negative
Unit	281.2	Unit	5	163	32	Negative
Unit	279.2	Unit	5	163	32	Negative
Unit	303.2	Unit	5	163	32	Negative
Unit	301.2	Unit	5	163	32	Negative
Unit	331.2	Unit	5	163	32	Negative
Unit	329.2	Unit	5	163	32	Negative
Unit	327.2	Unit	5	163	32	Negative
Unit	104.1	Unit	5	180	24	Positive
Unit	523.4	Unit	5	110	9	Positive
Unit	549.5	Unit	5	110	9	Positive
Unit	551.5	Unit	5	110	9	Positive
Unit	549.5	Unit	5	110	9	Positive
Unit	579.5	Unit	5	110	9	Positive
Unit	577.5	Unit	5	110	9	Positive
Unit	599.5	Unit	5	110	9	Positive
Unit	623.5	Unit	5	110	9	Positive
Unit	547.5	Unit	5	110	9	Positive
Unit	575.5	Unit	5	110	9	Positive
Unit	573.5	Unit	5	110	9	Positive
Unit	621.5	Unit	5	110	9	Positive

Unit	607.5	Unit	5	110	9	Positive
Unit	605.5	Unit	5	110	9	Positive
Unit	603.5	Unit	5	110	9	Positive
Unit	601.5	Unit	5	110	9	Positive
Unit	633.5	Unit	5	110	9	Positive
Unit	631.5	Unit	5	110	9	Positive
Unit	629.6	Unit	5	110	9	Positive
Unit	627.5	Unit	5	110	9	Positive
Unit	625.5	Unit	5	110	9	Positive
Unit	659.5	Unit	5	110	9	Positive
Unit	657.5	Unit	5	110	9	Positive
Unit	655.5	Unit	5	110	9	Positive
Unit	653.5	Unit	5	110	9	Positive
Unit	651.5	Unit	5	110	9	Positive
Unit	649.5	Unit	5	110	9	Positive
Unit	647.5	Unit	5	110	9	Positive
Unit	643.5	Unit	5	110	9	Positive
Unit	671.5	Unit	5	110	9	Positive
Unit	669.5	Unit	5	110	9	Positive
Unit	699.5	Unit	5	110	9	Positive
Unit	697.5	Unit	5	110	9	Positive
Unit	695.5	Unit	5	110	9	Positive
Unit	86.1	Unit	5	215	50	Positive
Unit	124.9	Unit	5	215	50	Positive

Compound group	Compoun	Compoun	Ion speci	CAS	z	Monoisot	ISTD	Precurso
PE(16:0_16:0)	PE(16:0_	C42H82N	[M+H]+		1		FALSE	692.5
PE(16:0_16:1)	PE(16:0_	C42H82N	[M+H]+		1		FALSE	690.5
PE(16:0_18:1)	PE(16:0_	C42H82N	[M+H]+		1		FALSE	718.5
PE(16:0_18:2)	PE(16:0_	C42H82N	[M+H]+		1		FALSE	716.5
PE(16:0_18:3) (a)	PE(16:0_	C42H82N	[M+H]+		1		FALSE	714.5
PE(16:0_20:3)	PE(16:0_	C42H82N	[M+H]+		1		FALSE	742.5
PE(16:0_20:4)	PE(16:0_	C42H82N	[M+H]+		1		FALSE	740.5
PE(16:0_20:5)	PE(16:0_	C42H82N	[M+H]+		1		FALSE	738.5
PE(16:0_22:6)	PE(16:0_	C42H82N	[M+H]+		1		FALSE	764.5
PE(16:1_18:2)	PE(16:1_	C42H82N	[M+H]+		1		FALSE	714.5
PE(16:1_20:4)	PE(16:1_	C42H82N	[M+H]+		1		FALSE	738.5
PE(17:0_18:1)	PE(17:0_	C42H82N	[M+H]+		1		FALSE	732.6
PE(17:0_18:2)	PE(17:0_	C42H82N	[M+H]+		1		FALSE	730.5
PE(17:0_20:4)	PE(17:0_	C42H82N	[M+H]+		1		FALSE	754.6
PE(18:0_18:1)	PE(18:0_	C42H82N	[M+H]+		1		FALSE	746.6
PE(18:0_18:2)	PE(18:0_	C42H82N	[M+H]+		1		FALSE	744.6
PE(18:0_20:3) (a)	PE(18:0_	C42H82N	[M+H]+		1		FALSE	770.6
PE(18:0_20:4)	PE(18:0_	C42H82N	[M+H]+		1		FALSE	768.6
PE(18:0_22:4)	PE(18:0_	C42H82N	[M+H]+		1		FALSE	796.6
PE(18:0_22:5) (n3)	PE(18:0_	C42H82N	[M+H]+		1		FALSE	794.6
PE(18:0_22:5) (n6)	PE(18:0_	C42H82N	[M+H]+		1		FALSE	794.6
PE(18:0_22:6)	PE(18:0_	C42H82N	[M+H]+		1		FALSE	792.6
PE(18:1_18:1)	PE(18:1_	C42H82N	[M+H]+		1		FALSE	744.6
PE(18:1_18:2)	PE(18:1_	C42H82N	[M+H]+		1		FALSE	742.5
PE(18:1_22:6) (a)	PE(18:1_	C42H82N	[M+H]+		1		FALSE	790.5
PE(20:0_20:4)	PE(20:0_	C42H82N	[M+H]+		1		FALSE	796.6
PE(36:0)	PE(36:0)	C42H82N	[M+H]+		1		FALSE	748.6
PE(38:5) (a)	PE(38:5)	C42H82N	[M+H]+		1		FALSE	766.5
PE(O-16:0/18:2)	PE(O-16:	C42H82N	[M+H]+		1		FALSE	702.5
PE(O-16:0/20:3)	PE(O-16:	C42H82N	[M+H]+		1		FALSE	728.6
PE(O-16:0/20:4)	PE(O-16:	C42H82N	[M+H]+		1		FALSE	726.5
PE(O-16:0/22:4)	PE(O-16:	C42H82N	[M+H]+		1		FALSE	754.6
PE(O-16:0/22:6)	PE(O-16:	C42H82N	[M+H]+		1		FALSE	750.6
PE(O-18:0/20:4)	PE(O-18:	C42H82N	[M+H]+		1		FALSE	754.6
PE(O-18:0/22:5) (a)	PE(O-18:	C42H82N	[M+H]+		1		FALSE	780.6
PE(O-18:0/22:6)	PE(O-18:	C42H82N	[M+H]+		1		FALSE	778.5
PE(O-18:1/18:2)	PE(O-18:	C42H82N	[M+H]+		1		FALSE	728.6

PE(O-18:1/22:6)	PE(O-18:	C42H82I	[M+H]+	1	FALSE	776.6
PE(O-34:1)	PE(O-34:	C42H82I	[M+H]+	1	FALSE	704.6
PE(O-36:5)	PE(O-36:	C42H82I	[M+H]+	1	FALSE	724.5
PE(O-38:5) (a)	PE(O-38:	C42H82I	[M+H]+	1	FALSE	752.6
PE(O-38:5) (b)	PE(O-38:	C42H82I	[M+H]+	1	FALSE	752.6
PE(P-15:0/20:4) (a)	PE(P-15:	C42H82I	[M+H]+	1	FALSE	710.5
PE(P-15:0/22:6) (a)	PE(P-15:	C42H82I	[M+H]+	1	FALSE	734.5
PE(P-16:0/18:1)	PE(P-16:	C42H82I	[M+H]+	1	FALSE	702.5
PE(P-16:0/18:2)	PE(P-16:	C42H82I	[M+H]+	1	FALSE	700.5
PE(P-16:0/18:3)	PE(P-16:	C42H82I	[M+H]+	1	FALSE	698.5
PE(P-16:0/20:3) (a)	PE(P-16:	C42H82I	[M+H]+	1	FALSE	726.5
PE(P-16:0/20:4)	PE(P-16:	C42H82I	[M+H]+	1	FALSE	724.5
PE(P-16:0/20:5)	PE(P-16:	C42H82I	[M+H]+	1	FALSE	722.5
PE(P-16:0/22:4)	PE(P-16:	C42H82I	[M+H]+	1	FALSE	752.6
PE(P-16:0/22:5) (n3)	PE(P-16:	C42H82I	[M+H]+	1	FALSE	750.5
PE(P-16:0/22:5) (n6)	PE(P-16:	C42H82I	[M+H]+	1	FALSE	750.5
PE(P-16:0/22:6)	PE(P-16:	C42H82I	[M+H]+	1	FALSE	748.5
PE(P-17:0/20:4) (a)	PE(P-17:	C42H82I	[M+H]+	1	FALSE	738.6
PE(P-17:0/22:6) (a)	PE(P-17:	C42H82I	[M+H]+	1	FALSE	762.6
PE(P-18:0/18:1)	PE(P-18:	C42H82I	[M+H]+	1	FALSE	730.6
PE(P-18:0/18:2)	PE(P-18:	C42H82I	[M+H]+	1	FALSE	728.6
PE(P-18:0/18:3)	PE(P-18:	C42H82I	[M+H]+	1	FALSE	726.5
PE(P-18:0/20:3) (a)	PE(P-18:	C42H82I	[M+H]+	1	FALSE	754.5
PE(P-18:0/20:4)	PE(P-18:	C42H82I	[M+H]+	1	FALSE	752.6
PE(P-18:0/20:5)	PE(P-18:	C42H82I	[M+H]+	1	FALSE	750.5
PE(P-18:0/22:4)	PE(P-18:	C42H82I	[M+H]+	1	FALSE	780.6
PE(P-18:0/22:5) (n3)	PE(P-18:	C42H82I	[M+H]+	1	FALSE	778.5
PE(P-18:0/22:5) (n6)	PE(P-18:	C42H82I	[M+H]+	1	FALSE	778.5
PE(P-18:0/22:6)	PE(P-18:	C42H82I	[M+H]+	1	FALSE	776.6
PE(P-18:1/18:1) (a)	PE(P-18:	C42H82I	[M+H]+	1	FALSE	728.6
PE(P-18:1/18:2) (a)	PE(P-18:	C42H82I	[M+H]+	1	FALSE	726.5
PE(P-18:1/18:3)	PE(P-18:	C42H82I	[M+H]+	1	FALSE	724.5
PE(P-18:1/20:3) (a)	PE(P-18:	C42H82I	[M+H]+	1	FALSE	752.5
PE(P-18:1/20:4) (a)	PE(P-18:	C42H82I	[M+H]+	1	FALSE	750.5
PE(P-18:1/20:5) (a)	PE(P-18:	C42H82I	[M+H]+	1	FALSE	748.5
PE(P-18:1/22:4)	PE(P-18:	C42H82I	[M+H]+	1	FALSE	778.5
PE(P-18:1/22:5) (a)	PE(P-18:	C42H82I	[M+H]+	1	FALSE	776.6
PE(P-18:1/22:6) (a)	PE(P-18:	C42H82I	[M+H]+	1	FALSE	774.5

PE(P-19:0/20:4) (a)	PE(P-19:	C42H82N	[M+H]+	1	FALSE	766.6
PE(P-20:0/18:1)	PE(P-20:	C42H82N	[M+H]+	1	FALSE	758.6
PE(P-20:0/18:2)	PE(P-20:	C42H82N	[M+H]+	1	FALSE	756.6
PE(P-20:0/20:4)	PE(P-20:	C42H82N	[M+H]+	1	FALSE	780.6
PE(P-20:0/22:6)	PE(P-20:	C42H82N	[M+H]+	1	FALSE	804.6
PE(P-20:1/20:4)	PE(P-20:	C42H82N	[M+H]+	1	FALSE	778.5
PE(P-20:1/22:6) (a)	PE(P-20:	C42H82N	[M+H]+	1	FALSE	802.6
PE(18:1_20:4)	PE(18:1_	C42H82N	[M+H]+	1	FALSE	766.5
PE(19:1_19:1)	PE(19:1_	C42H82N	[M+H]+	1	FALSE	772.6
PG(34:1)	PG(34:1)	C42H82N	[M+NH4]+	1	FALSE	766.6
PG(34:2)	PG(34:2)	C42H82N	[M+NH4]+	1	FALSE	792.6
PG(36:1)	PG(36:1)	C42H82N	[M+NH4]+	1	FALSE	764.6
PG(36:2)	PG(36:2)	C42H82N	[M+NH4]+	1	FALSE	794.6
PG(16:0_17:0)	PG(16:0_	C42H82N	[M+NH4]+	1	FALSE	737.5
PI(15-MHDA_18:1)/PI(17:0_18:1)	PI(15-MH	C42H82N	[M+NH4]+	1	FALSE	868.6
PI(15-MHDA_18:2)/PI(17:0_18:2)	PI(15-MH	C42H82N	[M+NH4]+	1	FALSE	866.6
PI(15-MHDA_20:4)/PI(17:0_20:4)	PI(15-MH	C42H82N	[M+NH4]+	1	FALSE	890.6
PI(16:0/16:0)	PI(16:0/1	C42H82N	[M+NH4]+	1	FALSE	828.6
PI(16:0_16:1)	PI(16:0_1	C42H82N	[M+NH4]+	1	FALSE	826.5
PI(16:0_20:3) (a)	PI(16:0_2	C42H82N	[M+NH4]+	1	FALSE	878.6
PI(16:0_20:4)	PI(16:0_2	C42H82N	[M+NH4]+	1	FALSE	876.6
PI(18:0_18:1)	PI(18:0_1	C42H82N	[M+NH4]+	1	FALSE	882.6
PI(18:0_20:2)	PI(18:0_2	C42H82N	[M+NH4]+	1	FALSE	908.6
PI(18:0_20:3) (a)	PI(18:0_2	C42H82N	[M+NH4]+	1	FALSE	906.6
PI(18:0_20:4)	PI(18:0_2	C42H82N	[M+NH4]+	1	FALSE	904.6
PI(18:0_22:4)	PI(18:0_2	C42H82N	[M+NH4]+	1	FALSE	932.6
PI(18:0_22:5) (n3)	PI(18:0_2	C42H82N	[M+NH4]+	1	FALSE	930.6
PI(18:0_22:5) (n6)	PI(18:0_2	C42H82N	[M+NH4]+	1	FALSE	930.6
PI(18:0_22:6)	PI(18:0_2	C42H82N	[M+NH4]+	1	FALSE	928.6
Sitosterolester(18:3)	Sitosterol	C29H49	[M+H]+	1	FALSE	675.61

MS1 res	Product r	MS2 res	Dwell (m	Fragmen	CE (V)	Polarity
Unit	551.5	Unit	20	166	17	Positive
Unit	549.5	Unit	20	166	17	Positive
Unit	577.5	Unit	20	166	17	Positive
Unit	575.5	Unit	20	166	17	Positive
Unit	573.5	Unit	20	166	17	Positive
Unit	601.5	Unit	20	166	17	Positive
Unit	599.5	Unit	20	166	17	Positive
Unit	597.5	Unit	20	166	17	Positive
Unit	623.5	Unit	20	166	17	Positive
Unit	573.5	Unit	20	166	17	Positive
Unit	597.5	Unit	20	166	17	Positive
Unit	591.5	Unit	20	166	17	Positive
Unit	589.5	Unit	20	166	17	Positive
Unit	613.5	Unit	20	166	17	Positive
Unit	605.6	Unit	20	166	17	Positive
Unit	603.5	Unit	20	166	17	Positive
Unit	629.6	Unit	20	166	17	Positive
Unit	627.5	Unit	20	166	17	Positive
Unit	655.6	Unit	20	166	17	Positive
Unit	653.6	Unit	20	166	17	Positive
Unit	653.6	Unit	20	166	17	Positive
Unit	651.5	Unit	20	166	17	Positive
Unit	603.5	Unit	20	166	17	Positive
Unit	601.5	Unit	20	166	17	Positive
Unit	649.5	Unit	20	166	17	Positive
Unit	655.6	Unit	20	166	17	Positive
Unit	607.6	Unit	20	166	17	Positive
Unit	625.5	Unit	20	166	17	Positive
Unit	561.5	Unit	20	166	17	Positive
Unit	587.5	Unit	20	166	17	Positive
Unit	585.5	Unit	20	166	17	Positive
Unit	613.6	Unit	20	166	17	Positive
Unit	609.5	Unit	20	166	17	Positive
Unit	613.6	Unit	20	166	17	Positive
Unit	639.6	Unit	20	166	17	Positive
Unit	637.5	Unit	20	166	17	Positive
Unit	587.5	Unit	20	166	17	Positive

Unit	635.5	Unit	20	166	17	Positive
Unit	563.5	Unit	20	166	17	Positive
Unit	583.5	Unit	20	166	17	Positive
Unit	611.5	Unit	20	166	17	Positive
Unit	611.5	Unit	20	166	17	Positive
Unit	361.3	Unit	20	166	17	Positive
Unit	385.3	Unit	20	166	17	Positive
Unit	339.3	Unit	20	166	17	Positive
Unit	337.3	Unit	20	166	17	Positive
Unit	335.3	Unit	20	166	17	Positive
Unit	363.3	Unit	20	166	17	Positive
Unit	361.3	Unit	20	166	17	Positive
Unit	359.3	Unit	20	166	17	Positive
Unit	389.3	Unit	20	166	17	Positive
Unit	387.3	Unit	20	166	17	Positive
Unit	387.3	Unit	20	166	17	Positive
Unit	385.3	Unit	20	166	17	Positive
Unit	361.3	Unit	20	166	17	Positive
Unit	385.3	Unit	20	166	17	Positive
Unit	339.3	Unit	20	166	17	Positive
Unit	337.3	Unit	20	166	17	Positive
Unit	335.3	Unit	20	166	17	Positive
Unit	363.3	Unit	20	166	17	Positive
Unit	361.3	Unit	20	166	17	Positive
Unit	359.3	Unit	20	166	17	Positive
Unit	389.3	Unit	20	166	17	Positive
Unit	387.3	Unit	20	166	17	Positive
Unit	387.3	Unit	20	166	17	Positive
Unit	385.3	Unit	20	166	17	Positive
Unit	339.3	Unit	20	166	17	Positive
Unit	337.3	Unit	20	166	17	Positive
Unit	335.3	Unit	20	166	17	Positive
Unit	363.3	Unit	20	166	17	Positive
Unit	361.3	Unit	20	166	17	Positive
Unit	359.3	Unit	20	166	17	Positive
Unit	389.3	Unit	20	166	17	Positive
Unit	387.3	Unit	20	166	17	Positive
Unit	385.3	Unit	20	166	17	Positive

Unit	361.3	Unit	20	166	17	Positive
Unit	339.3	Unit	20	166	17	Positive
Unit	337.3	Unit	20	166	17	Positive
Unit	361.3	Unit	20	166	17	Positive
Unit	385.3	Unit	20	166	17	Positive
Unit	361.3	Unit	20	166	17	Positive
Unit	385.3	Unit	20	166	17	Positive
Unit	577.5	Unit	20	166	17	Positive
Unit	591.5	Unit	20	166	17	Positive
Unit	577.5	Unit	20	166	17	Positive
Unit	603.5	Unit	20	166	21	Positive
Unit	575.5	Unit	20	166	21	Positive
Unit	605.6	Unit	20	166	21	Positive
Unit	565.5	Unit	20	166	21	Positive
Unit	591.6	Unit	20	166	17	Positive
Unit	589.6	Unit	20	166	17	Positive
Unit	613.6	Unit	20	166	17	Positive
Unit	551.6	Unit	20	166	17	Positive
Unit	549.5	Unit	20	166	17	Positive
Unit	601.6	Unit	20	166	17	Positive
Unit	599.6	Unit	20	166	17	Positive
Unit	605.6	Unit	20	166	17	Positive
Unit	631.6	Unit	20	166	17	Positive
Unit	629.6	Unit	20	166	17	Positive
Unit	627.6	Unit	20	166	17	Positive
Unit	655.6	Unit	20	166	17	Positive
Unit	653.6	Unit	20	166	17	Positive
Unit	653.6	Unit	20	166	17	Positive
Unit	651.6	Unit	20	166	17	Positive
Unit	397.4	Unit	20	166	23	Positive

Compound group	Compound name	Compou	Ion speci	CAS	z	Monoisot	ISTD	Precurso	MS1 res
Ubiquinone	Ubiquinone	C42H82N	[M+NH4]+		1		FALSE	880.7	Unit
TG(6:0_12:0_18:3)	TG(6:0_11:2_18:3)	C42H82N	[M+NH4]+		1		FALSE	650.5	Unit
TG(6:0_12:0_18:3)	TG(6:0_11:2_18:3)	C42H82N	[M+NH4]+		1		FALSE	650.5	Unit
TG(6:0_12:0_18:3)	TG(6:0_11:2_18:3)	C42H82N	[M+NH4]+		1		FALSE	650.5	Unit
TG(22:1_10:0_10:0)	TG(22:1_10:0_10:0	C42H82N	[M+NH4]+		1		FALSE	738.66	Unit
TG(22:1_10:0_10:0)	TG(22:1_10:0_10:0	C42H82N	[M+NH4]+		1		FALSE	738.66	Unit
TG(22:1_10:0_10:0)	TG(22:1_10:0_10:0	C42H82N	[M+NH4]+		1		FALSE	738.66	Unit
TG(18:4_18:2_18:2)	TG(18:4_18:2_18:2	C42H82N	[M+NH4]+		1		FALSE	892.7	Unit
TG(18:4_18:2_18:2)	TG(18:4_18:2_18:2	C42H82N	[M+NH4]+		1		FALSE	892.7	Unit
TG(18:4_18:2_18:2)	TG(18:4_18:2_18:2	C42H82N	[M+NH4]+		1		FALSE	892.7	Unit
TG(18:4_16:0_18:2)	TG(18:4_16:0_18:2	C42H82N	[M+NH4]+		1		FALSE	868.7	Unit
TG(18:4_16:0_18:2)	TG(18:4_16:0_18:2	C42H82N	[M+NH4]+		1		FALSE	868.7	Unit
TG(18:4_16:0_18:2)	TG(18:4_16:0_18:2	C42H82N	[M+NH4]+		1		FALSE	868.7	Unit
TG(18:3_18:2_18:2)	TG(18:3_18:2_18:2	C42H82N	[M+NH4]+		1		FALSE	894.8	Unit
TG(18:3_18:2_18:2)	TG(18:3_18:2_18:2	C42H82N	[M+NH4]+		1		FALSE	894.8	Unit
TG(18:3_18:2_18:2)	TG(18:3_18:2_18:2	C42H82N	[M+NH4]+		1		FALSE	894.8	Unit
TG(18:3_18:2_16:0)	TG(18:3_18:2_16:0	C42H82N	[M+NH4]+		1		FALSE	870.8	Unit
TG(18:3_18:2_16:0)	TG(18:3_18:2_16:0	C42H82N	[M+NH4]+		2		FALSE	870.8	Unit
TG(18:3_18:2_16:0)	TG(18:3_18:2_16:0	C42H82N	[M+NH4]+		3		FALSE	870.8	Unit
TG(18:2_18:2_20:4)	TG(18:2_18:2_20:4	C42H82N	[M+NH4]+		1		FALSE	920.8	Unit
TG(18:2_18:2_20:4)	TG(18:2_18:2_20:4	C42H82N	[M+NH4]+		2		FALSE	920.8	Unit
TG(18:2_18:2_20:4)	TG(18:2_18:2_20:4	C42H82N	[M+NH4]+		3		FALSE	920.8	Unit
TG(18:2_18:2_18:2)	TG(18:2_18:2_18:2	C42H82N	[M+NH4]+		1		FALSE	896.8	Unit
TG(18:2_18:2_16:0)	TG(18:2_18:2_16:0	C42H82N	[M+NH4]+		1		FALSE	872.8	Unit
TG(18:2_18:2_16:0)	TG(18:2_18:2_16:0	C42H82N	[M+NH4]+		1		FALSE	872.8	Unit
TG(18:2_18:2_16:0)	TG(18:2_18:2_16:0	C42H82N	[M+NH4]+		1		FALSE	872.8	Unit
TG(18:2_18:2_15:0)	TG(18:2_18:2_15:0	C42H82N	[M+NH4]+		1		FALSE	858.8	Unit
TG(18:2_18:2_15:0)	TG(18:2_18:2_15:0	C42H82N	[M+NH4]+		1		FALSE	858.8	Unit
TG(18:1e_16:0_16:0)	TG(18:1e_16:0_16	C42H82N	[M+NH4]+		1		FALSE	850.8	Unit
TG(18:1e_16:0_16:0)	TG(18:1e_16:0_16	C42H82N	[M+NH4]+		1		FALSE	850.8	Unit
TG(18:1_18:2_20:3)	TG(18:1_18:2_20:3	C42H82N	[M+NH4]+		1		FALSE	924.8	Unit
TG(18:1_18:2_20:3)	TG(18:1_18:2_20:3	C42H82N	[M+NH4]+		1		FALSE	924.8	Unit
TG(18:1_18:2_20:3)	TG(18:1_18:2_20:3	C42H82N	[M+NH4]+		1		FALSE	924.8	Unit
TG(18:1_18:2_18:2)	TG(18:1_18:2_18:2	C42H82N	[M+NH4]+		1		FALSE	898.8	Unit
TG(18:1_18:2_18:2)	TG(18:1_18:2_18:2	C42H82N	[M+NH4]+		1		FALSE	898.8	Unit
TG(18:1_18:2_18:2)	TG(18:1_18:2_18:2	C42H82N	[M+NH4]+		1		FALSE	898.8	Unit
TG(18:1_18:1_22:6)	TG(18:1_18:1_22:6	C42H82N	[M+NH4]+		1		FALSE	948.8	Unit

TG(18:1_18:1_22:6)	TG(18:1_18:1_22:	C42H82N	[M+NH4]+	1	FALSE	948.8	Unit
TG(18:1_18:1_22:6)	TG(18:1_18:1_22:	C42H82N	[M+NH4]+	1	FALSE	948.8	Unit
TG(18:1_18:1_18:3)	TG(18:1_18:1_18:	C42H82N	[M+NH4]+	1	FALSE	898.8	Unit
TG(18:1_18:1_18:3)	TG(18:1_18:1_18:	C42H82N	[M+NH4]+	1	FALSE	898.8	Unit
TG(18:1_18:1_18:3)	TG(18:1_18:1_18:	C42H82N	[M+NH4]+	1	FALSE	898.8	Unit
TG(18:1_18:1_18:1)	TG(18:1_18:1_18:	C42H82N	[M+NH4]+	1	FALSE	902.8	Unit
TG(18:1_18:1_18:1)	TG(18:1_18:1_18:	C42H82N	[M+NH4]+	1	FALSE	902.8	Unit
TG(18:1_18:1_18:1)	TG(18:1_18:1_18:	C42H82N	[M+NH4]+	1	FALSE	902.8	Unit
TG(18:0_18:1_22:6)	TG(18:0_18:1_22:	C42H82N	[M+NH4]+	1	FALSE	950.8	Unit
TG(18:0_18:1_22:6)	TG(18:0_18:1_22:	C42H82N	[M+NH4]+	1	FALSE	950.8	Unit
TG(18:0_18:1_22:6)	TG(18:0_18:1_22:	C42H82N	[M+NH4]+	1	FALSE	950.8	Unit
TG(18:0_18:1_18:3)	TG(18:0_18:1_18:	C42H82N	[M+NH4]+	1	FALSE	900.8	Unit
TG(18:0_18:1_18:3)	TG(18:0_18:1_18:	C42H82N	[M+NH4]+	1	FALSE	900.8	Unit
TG(18:0_18:1_18:3)	TG(18:0_18:1_18:	C42H82N	[M+NH4]+	1	FALSE	900.8	Unit
TG(18:0_16:0_18:3)	TG(18:0_16:0_18:	C42H82N	[M+NH4]+	1	FALSE	874.8	Unit
TG(18:0_16:0_18:3)	TG(18:0_16:0_18:	C42H82N	[M+NH4]+	1	FALSE	874.8	Unit
TG(18:0_16:0_18:3)	TG(18:0_16:0_18:	C42H82N	[M+NH4]+	1	FALSE	874.8	Unit
TG(18:0_16:0_16:0)	TG(18:0_16:0_16:	C42H82N	[M+NH4]+	1	FALSE	852.8	Unit
TG(18:0_16:0_16:0)	TG(18:0_16:0_16:	C42H82N	[M+NH4]+	1	FALSE	852.8	Unit
TG(18:0_16:0_16:0)	TG(18:0_16:0_16:	C42H82N	[M+NH4]+	1	FALSE	852.8	Unit
TG(16:1_18:2_18:3)	TG(16:1_18:2_18:	C42H82N	[M+NH4]+	1	FALSE	868.7	Unit
TG(16:1_18:2_18:3)	TG(16:1_18:2_18:	C42H82N	[M+NH4]+	1	FALSE	868.7	Unit
TG(16:1_18:2_18:3)	TG(16:1_18:2_18:	C42H82N	[M+NH4]+	1	FALSE	868.7	Unit
TG(16:1_18:2_15:0)	TG(16:1_18:2_15:	C42H82N	[M+NH4]+	1	FALSE	832.7	Unit
TG(16:1_18:2_15:0)	TG(16:1_18:2_15:	C42H82N	[M+NH4]+	1	FALSE	832.7	Unit
TG(16:1_18:2_15:0)	TG(16:1_18:2_15:	C42H82N	[M+NH4]+	1	FALSE	832.7	Unit
TG(16:1_14:1_15:0)	TG(16:1_14:1_15:	C42H82N	[M+NH4]+	1	FALSE	778.7	Unit
TG(16:1_14:1_15:0)	TG(16:1_14:1_15:	C42H82N	[M+NH4]+	1	FALSE	778.7	Unit
TG(16:1_14:1_15:0)	TG(16:1_14:1_15:	C42H82N	[M+NH4]+	1	FALSE	778.7	Unit
TG(16:0_18:2_22:4)	TG(16:0_18:2_22:	C42H82N	[M+NH4]+	1	FALSE	924.8	Unit
TG(16:0_18:2_22:4)	TG(16:0_18:2_22:	C42H82N	[M+NH4]+	1	FALSE	924.8	Unit
TG(16:0_18:2_22:4)	TG(16:0_18:2_22:	C42H82N	[M+NH4]+	1	FALSE	924.8	Unit
TG(16:0_18:1_22:6)	TG(16:0_18:1_22:	C42H82N	[M+NH4]+	1	FALSE	922.8	Unit
TG(16:0_18:1_22:6)	TG(16:0_18:1_22:	C42H82N	[M+NH4]+	1	FALSE	922.8	Unit
TG(16:0_18:1_22:6)	TG(16:0_18:1_22:	C42H82N	[M+NH4]+	1	FALSE	922.8	Unit
TG(16:0_18:1_18:3)	TG(16:0_18:1_18:	C42H82N	[M+NH4]+	1	FALSE	872.8	Unit
TG(16:0_18:1_18:3)	TG(16:0_18:1_18:	C42H82N	[M+NH4]+	2	FALSE	872.8	Unit
TG(16:0_18:1_18:3)	TG(16:0_18:1_18:	C42H82N	[M+NH4]+	3	FALSE	872.8	Unit

TG(15:0_18:2_18:3)	TG(15:0_18:2_18:3	C42H82N	[M+NH4]+	1	FALSE	856.7	Unit
TG(15:0_18:2_18:3)	TG(15:0_18:2_18:3	C42H82N	[M+NH4]+	1	FALSE	856.7	Unit
TG(15:0_18:2_18:3)	TG(15:0_18:2_18:3	C42H82N	[M+NH4]+	1	FALSE	856.7	Unit
TG(14:0_14:1_13:0)	TG(14:0_14:1_13:(	C42H82N	[M+NH4]+	1	FALSE	724.6	Unit
TG(14:0_14:1_13:0)	TG(14:0_14:1_13:(	C42H82N	[M+NH4]+	1	FALSE	724.6	Unit
TG(14:0_14:1_13:0)	TG(14:0_14:1_13:(	C42H82N	[M+NH4]+	1	FALSE	724.6	Unit
TG(12:1e_6:0_18:3)	TG(12:1e_6:0_18:3	C42H82N	[M+NH4]+	1	FALSE	634.5	Unit
TG(12:0_14:0_16:0)	TG(12:0_14:0_16:(	C42H82N	[M+NH4]+	1	FALSE	740.7	Unit
TG(12:0_14:0_16:0)	TG(12:0_14:0_16:(	C42H82N	[M+NH4]+	1	FALSE	740.7	Unit
TG(12:0_14:0_16:0)	TG(12:0_14:0_16:(	C42H82N	[M+NH4]+	1	FALSE	740.7	Unit
TG(12:0_12:0_14:0)	TG(12:0_12:0_14:(	C42H82N	[M+NH4]+	1	FALSE	684.6	Unit
TG(12:0_12:0_14:0)	TG(12:0_12:0_14:(	C42H82N	[M+NH4]+	1	FALSE	684.6	Unit

Product r	MS2 res	Dwell (m	Fragmen	CE (V)	Polarity
197.0	Unit	20	166	17	Positive
517.4	Unit	20	166	21	Positive
433.3	Unit	20	166	21	Positive
355.3	Unit	20	166	21	Positive
549.5	Unit	20	166	21	Positive
549.5	Unit	20	166	21	Positive
383.3	Unit	20	166	21	Positive
599.5	Unit	20	166	21	Positive
595.5	Unit	20	166	21	Positive
595.5	Unit	20	166	21	Positive
595.5	Unit	20	166	21	Positive
575.5	Unit	20	166	21	Positive
571.5	Unit	20	166	21	Positive
599.5	Unit	20	166	21	Positive
597.5	Unit	20	166	21	Positive
597.5	Unit	20	166	21	Positive
597.5	Unit	20	166	21	Positive
575.5	Unit	20	166	21	Positive
573.5	Unit	20	166	21	Positive
623.5	Unit	20	166	21	Positive
623.5	Unit	20	166	21	Positive
599.5	Unit	20	166	21	Positive
599.5	Unit	20	166	21	Positive
599.5	Unit	20	166	21	Positive
575.5	Unit	20	166	21	Positive
575.5	Unit	20	166	21	Positive
599.5	Unit	20	166	21	Positive
561.5	Unit	20	166	21	Positive
551.5	Unit	20	166	21	Positive
577.5	Unit	20	166	21	Positive
625.5	Unit	20	166	21	Positive
627.5	Unit	20	166	21	Positive
601.5	Unit	20	166	21	Positive
599.5	Unit	20	166	21	Positive
601.5	Unit	20	166	21	Positive
601.5	Unit	20	166	21	Positive
649.5	Unit	20	166	21	Positive

649.5	Unit	20	166	21	Positive
603.5	Unit	20	166	21	Positive
603.5	Unit	20	166	21	Positive
599.5	Unit	20	166	21	Positive
599.5	Unit	20	166	21	Positive
603.5	Unit	20	166	21	Positive
603.5	Unit	20	166	21	Positive
603.5	Unit	20	166	21	Positive
651.5	Unit	20	166	21	Positive
649.5	Unit	20	166	21	Positive
605.6	Unit	20	166	21	Positive
605.6	Unit	20	166	21	Positive
601.5	Unit	20	166	21	Positive
599.5	Unit	20	166	21	Positive
601.5	Unit	20	166	21	Positive
579.5	Unit	20	166	21	Positive
573.5	Unit	20	166	21	Positive
551.5	Unit	20	166	21	Positive
579.5	Unit	20	166	21	Positive
579.5	Unit	20	166	21	Positive
597.5	Unit	20	166	21	Positive
573.5	Unit	20	166	21	Positive
571.5	Unit	20	166	21	Positive
573.5	Unit	20	166	21	Positive
561.5	Unit	20	166	21	Positive
535.5	Unit	20	166	21	Positive
507.4	Unit	20	166	21	Positive
519.4	Unit	20	166	21	Positive
535.5	Unit	20	166	21	Positive
651.5	Unit	20	166	21	Positive
627.5	Unit	20	166	21	Positive
575.5	Unit	20	166	21	Positive
649.5	Unit	20	166	21	Positive
623.5	Unit	20	166	21	Positive
577.5	Unit	20	166	21	Positive
599.5	Unit	20	166	21	Positive
577.5	Unit	20	166	21	Positive
573.5	Unit	20	166	21	Positive

597.5	Unit	20	166	21	Positive
559.5	Unit	20	166	21	Positive
561.5	Unit	20	166	21	Positive
479.4	Unit	20	166	21	Positive
481.4	Unit	20	166	21	Positive
493.4	Unit	20	166	21	Positive
335.3	Unit	20	166	21	Positive
523.5	Unit	20	166	21	Positive
495.4	Unit	20	166	21	Positive
467.4	Unit	20	166	21	Positive
467.4	Unit	20	166	21	Positive
439.4	Unit	20	166	21	Positive

Compound group	Compound name	Compound fo	Ion speci	CAS	z	Monoisot	ISTD
PI(18:1_18:2)	PI(18:1_18:2)	C42H82NO1	[M+NH4]+		1		FALSE
PI(20:0_20:4)	PI(20:0_20:4)	C42H82NO1	[M+NH4]+		1		FALSE
PI(34:0)	PI(34:0)	C42H82NO1	[M+NH4]+		1		FALSE
PI(34:1)	PI(34:1)	C42H82NO1	[M+NH4]+		1		FALSE
PI(36:2)	PI(36:2)	C42H82NO1	[M+NH4]+		1		FALSE
PI(37:6)	PI(37:6)	C42H82NO1	[M+NH4]+		1		FALSE
PI(38:5) (a)	PI(38:5) (a)	C42H82NO1	[M+NH4]+		1		FALSE
PI(38:6)	PI(38:6)	C42H82NO1	[M+NH4]+		1		FALSE
PI(39:6)	PI(39:6)	C42H82NO1	[M+NH4]+		1		FALSE
PS(36:1)	PS(36:1)	C42H82NO1	[M+H]+		1		FALSE
PS(36:2)	PS(36:2)	C42H82NO1	[M+H]+		1		FALSE
PS(38:3)	PS(38:3)	C42H82NO1	[M+H]+		1		FALSE
PS(38:4)	PS(38:4)	C42H82NO1	[M+H]+		1		FALSE
PS(38:5)	PS(38:5)	C42H82NO1	[M+H]+		1		FALSE
PS(40:5)	PS(40:5)	C42H82NO1	[M+H]+		1		FALSE
PS(40:6)	PS(40:6)	C42H82NO1	[M+H]+		1		FALSE
S1P(d16:1)	S1P(d16:1)		[M+H]+		1		FALSE
S1P(d18:0)	S1P(d18:0)		[M+H]+		1		FALSE
S1P(d18:1)	S1P(d18:1)		[M+H]+		1		FALSE
S1P(d18:2)	S1P(d18:2)		[M+H]+		1		FALSE
SM(34:3)	SM(34:3)		[M+H]+		1		FALSE
SM(38:3) (a)	SM(38:3) (a)		[M+H]+		1		FALSE
SM(40:3) (a)	SM(40:3) (a)		[M+H]+		1		FALSE
SM(41:0)	SM(41:0)		[M+H]+		1		FALSE
SM(41:1) (a)	SM(41:1) (a)		[M+H]+		1		FALSE
SM(43:1)	SM(43:1)		[M+H]+		1		FALSE
SM(43:2) (c)	SM(43:2) (c)		[M+H]+		1		FALSE
SM(44:1)	SM(44:1)		[M+H]+		1		FALSE
SM(44:2)	SM(44:2)		[M+H]+		1		FALSE
SM(44:3)	SM(44:3)		[M+H]+		1		FALSE
SM(d16:1/23:0)/SM(d17:1/22	SM(d16:1/23:0)/SM(d17:1/22:0)		[M+H]+		1		FALSE
SM(d16:1/24:1)	SM(d16:1/24:1)		[M+H]+		1		FALSE
SM(d18:0/14:0)	SM(d18:0/14:0)		[M+H]+		1		FALSE
SM(d18:0/16:0)	SM(d18:0/16:0)		[M+H]+		1		FALSE
SM(d18:0/22:0)	SM(d18:0/22:0)		[M+H]+		1		FALSE
SM(d18:1/14:0)/SM(d16:1/16	SM(d18:1/14:0)/SM(d16:1/16:0)		[M+H]+		1		FALSE
SM(d18:1/16:0)	SM(d18:1/16:0)		[M+H]+		1		FALSE

SM(d18:1/18:0)/SM(d16:1/20	SM(d18:1/18:0)/SM(d16:1/20:0)		[M+H]+	1	FALSE
SM(d18:1/20:0)/SM(d16:1/22	SM(d18:1/20:0)/SM(d16:1/22:0)		[M+H]+	1	FALSE
SM(d18:1/22:0)/SM(d16:1/24	SM(d18:1/22:0)/SM(d16:1/24:0)		[M+H]+	1	FALSE
SM(d18:1/23:0)/SM(d17:1/24	SM(d18:1/23:0)/SM(d17:1/24:0)		[M+H]+	1	FALSE
SM(d18:1/24:0)	SM(d18:1/24:0)		[M+H]+	1	FALSE
SM(d18:1/24:1)	SM(d18:1/24:1)		[M+H]+	1	FALSE
SM(d18:2/14:0)	SM(d18:2/14:0)		[M+H]+	1	FALSE
SM(d18:2/16:0)	SM(d18:2/16:0)		[M+H]+	1	FALSE
SM(d18:2/17:0)	SM(d18:2/17:0)		[M+H]+	1	FALSE
SM(d18:2/18:0)	SM(d18:2/18:0)		[M+H]+	1	FALSE
SM(d18:2/18:1)	SM(d18:2/18:1)		[M+H]+	1	FALSE
SM(d18:2/20:0)	SM(d18:2/20:0)		[M+H]+	1	FALSE
SM(d18:2/22:0)	SM(d18:2/22:0)		[M+H]+	1	FALSE
SM(d18:2/23:0)	SM(d18:2/23:0)	C42H79O10I	[M+H]+	1	FALSE
SM(d18:2/24:0)	SM(d18:2/24:0)		[M+H]+	1	FALSE
SM(d19:0_23:1)	SM(d19:0_23:1)		[M+H]+	1	FALSE
SM(d20:1_20:1)	SM(d20:1_20:1)		[M+H]+	1	FALSE
SM(d31:1)	SM(d31:1)		[M+H]+	1	FALSE
SM(d34:3)	SM(d34:3)		[M+H]+	1	FALSE
SM(d38:4)	SM(d38:4)		[M+H]+	1	FALSE
SM(d39:2)	SM(d39:2)		[M+H]+	1	FALSE
SM(d40:1)	SM(d40:1)		[M+H]+	1	FALSE
SM(d40:3)	SM(d40:3)		[M+H]+	1	FALSE
SM(d41:3)	SM(d41:3)		[M+H]+	1	FALSE
SM(d42:2)	SM(d42:2)		[M+H]+	1	FALSE
SM(d43:2)	SM(d43:2)		[M+H]+	1	FALSE
SM(d44:5)	SM(d44:5)		[M+H]+	1	FALSE
Sph(d16:1)	Sph(d16:1)	C42H82NO1	[M+H]+	1	FALSE
Sph(d18:1)	Sph(d18:1)	C42H79O10I	[M+H]+	1	FALSE
Sph(d18:2)	Sph(d18:2)		[M+H]+	1	FALSE
Sulfatide (d18:1:/16:0(OH))	Sulfatide (d18:1:/16:0(OH))		[M+H]+	1	FALSE
Sulfatide (d18:1:/16:0)	Sulfatide (d18:1:/16:0)		[M+H]+	1	FALSE
Sulfatide (d18:1:/24:0(OH))	Sulfatide (d18:1:/24:0(OH))		[M+H]+	1	FALSE
Sulfatide (d18:1:/24:0)	Sulfatide (d18:1:/24:0)		[M+H]+	1	FALSE
Sulfatide (d18:1:/24:1(OH))	Sulfatide (d18:1:/24:1(OH))		[M+H]+	1	FALSE
Sulfatide (d18:1:/24:1)	Sulfatide (d18:1:/24:1)		[M+H]+	1	FALSE
TG(48:0) [NL-16:0]	TG(48:0) [NL-16:0]	C42H82NO1	[M+NH4]+	1	FALSE
TG(48:1) [NL-18:1]	TG(48:1) [NL-18:1]	C42H82NO1	[M+NH4]+	1	FALSE

TG(48:2) [NL-14:1]	TG(48:2) [NL-14:1]	C42H82NO1 [M+NH4]+	1	FALSE
TG(48:2) [NL-16:0]	TG(48:2) [NL-16:0]	C42H82NO1 [M+NH4]+	1	FALSE
TG(48:2) [NL-16:1]	TG(48:2) [NL-16:1]	C42H82NO1 [M+NH4]+	1	FALSE
TG(48:2) [NL-18:1]	TG(48:2) [NL-18:1]	C42H82NO1 [M+NH4]+	1	FALSE
TG(48:3) [NL-16:1]	TG(48:3) [NL-16:1]	C42H82NO1 [M+NH4]+	1	FALSE
TG(48:3) [NL-18:2]	TG(48:3) [NL-18:2]	C42H82NO1 [M+NH4]+	1	FALSE
TG(49:1) [NL-15:0]	TG(49:1) [NL-15:0]	C42H82NO1 [M+NH4]+	1	FALSE
TG(50:0) [NL-18:0]	TG(50:0) [NL-18:0]	C42H82NO1 [M+NH4]+	1	FALSE
TG(50:1) [NL-14:0]	TG(50:1) [NL-14:0]	C42H82NO1 [M+NH4]+	1	FALSE
TG(50:1) [NL-18:1]	TG(50:1) [NL-18:1]	C42H82NO1 [M+NH4]+	1	FALSE
TG(50:2) [NL-18:0]	TG(50:2) [NL-18:0]	C42H82NO1 [M+NH4]+	1	FALSE
TG(50:2) [NL-18:1]	TG(50:2) [NL-18:1]	C42H82NO1 [M+NH4]+	1	FALSE
TG(50:2) [NL-18:2]	TG(50:2) [NL-18:2]	C42H82NO1 [M+NH4]+	1	FALSE
TG(50:3) [NL-14:1]	TG(50:3) [NL-14:1]	C42H82NO1 [M+NH4]+	1	FALSE
TG(50:3) [NL-16:1]	TG(50:3) [NL-16:1]	C42H82NO1 [M+NH4]+	1	FALSE
TG(50:3) [NL-18:1]	TG(50:3) [NL-18:1]	C42H82NO1 [M+NH4]+	1	FALSE
TG(50:4) [NL-14:0]	TG(50:4) [NL-14:0]	C42H82NO1 [M+NH4]+	1	FALSE
TG(52:1) [NL-18:0]	TG(52:1) [NL-18:0]	C42H82NO1 [M+NH4]+	1	FALSE
TG(52:2) [NL-16:0]	TG(52:2) [NL-16:0]	C42H82NO1 [M+NH4]+	1	FALSE
TG(52:3) [NL-16:1]	TG(52:3) [NL-16:1]	C42H82NO1 [M+NH4]+	1	FALSE
TG(52:3) [NL-18:2]	TG(52:3) [NL-18:2]	C42H82NO1 [M+NH4]+	1	FALSE
TG(52:4) [NL-16:0]	TG(52:4) [NL-16:0]	C42H82NO1 [M+NH4]+	1	FALSE
TG(52:4) [NL-18:1]	TG(52:4) [NL-18:1]	C42H82NO1 [M+NH4]+	1	FALSE
TG(54:0) [NL-18:0]	TG(54:0) [NL-18:0]	C42H82NO1 [M+NH4]+	1	FALSE
TG(54:1) [NL-18:1]	TG(54:1) [NL-18:1]	C42H82NO1 [M+NH4]+	1	FALSE
TG(54:2) [NL-18:0]	TG(54:2) [NL-18:0]	C42H82NO1 [M+NH4]+	1	FALSE
TG(54:3) [NL-18:1]	TG(54:3) [NL-18:1]	C42H82NO1 [M+NH4]+	1	FALSE
TG(54:4) [NL-18:0]	TG(54:4) [NL-18:0]	C42H82NO1 [M+NH4]+	1	FALSE
TG(54:4) [NL-18:2]	TG(54:4) [NL-18:2]	C42H82NO1 [M+NH4]+	1	FALSE
TG(54:5) [NL-18:1]	TG(54:5) [NL-18:1]	C42H82NO1 [M+NH4]+	1	FALSE
TG(54:6) [NL-18:2]	TG(54:6) [NL-18:2]	C42H82NO1 [M+NH4]+	1	FALSE
TG(56:6) [NL-20:4]	TG(56:6) [NL-20:4]	C42H82NO1 [M+NH4]+	1	FALSE
TG(56:8) [NL-20:4]	TG(56:8) [NL-20:4]	C42H82NO1 [M+NH4]+	1	FALSE
TG(58:8) [NL-22:6]	TG(58:8) [NL-22:6]	C42H82NO1 [M+NH4]+	1	FALSE
TG(O-50:1) [NL-16:0]	TG(O-50:1) [NL-16:0]	C42H82NO1 [M+NH4]+	1	FALSE
TG(O-52:0) [NL-16:0]	TG(O-52:0) [NL-16:0]	C42H82NO1 [M+NH4]+	1	FALSE
TG(O-52:2) [NL-16:0]	TG(O-52:2) [NL-16:0]	C42H82NO1 [M+NH4]+	1	FALSE

Precurso	MS1 res	Product r	MS2 res	Dwell (m:	Fragmen	CE (V)	Polarity
878.6	Unit	601.6	Unit	20	166	17	Positive
932.6	Unit	655.6	Unit	20	166	17	Positive
856.6	Unit	579.6	Unit	20	166	17	Positive
854.6	Unit	577.6	Unit	20	166	17	Positive
880.6	Unit	603.6	Unit	20	166	17	Positive
886.6	Unit	609.6	Unit	20	166	17	Positive
902.6	Unit	625.6	Unit	20	166	17	Positive
900.6	Unit	623.6	Unit	20	166	17	Positive
914.6	Unit	637.6	Unit	20	166	17	Positive
790.6	Unit	605.6	Unit	20	166	25	Positive
788.5	Unit	603.5	Unit	20	166	25	Positive
814.6	Unit	629.6	Unit	20	166	25	Positive
812.5	Unit	627.5	Unit	20	166	25	Positive
810.5	Unit	625.5	Unit	20	166	25	Positive
838.6	Unit	653.6	Unit	20	166	25	Positive
836.5	Unit	651.5	Unit	20	166	25	Positive
352.2	Unit	236.3	Unit	20	166	16	Positive
382.2	Unit	284.3	Unit	20	166	11	Positive
380.2	Unit	264.3	Unit	20	166	16	Positive
378.2	Unit	262.3	Unit	20	166	16	Positive
699.5	Unit	184.1	Unit	20	166	25	Positive
755.6	Unit	184.1	Unit	20	166	25	Positive
783.6	Unit	184.1	Unit	20	166	25	Positive
803.7	Unit	184.1	Unit	20	166	25	Positive
801.7	Unit	184.1	Unit	20	166	25	Positive
829.7	Unit	184.1	Unit	20	166	25	Positive
827.7	Unit	184.1	Unit	20	166	25	Positive
843.6	Unit	184.1	Unit	20	166	25	Positive
841.6	Unit	184.1	Unit	20	166	25	Positive
839.6	Unit	184.1	Unit	20	166	25	Positive
773.7	Unit	184.1	Unit	20	166	25	Positive
785.7	Unit	184.1	Unit	20	166	25	Positive
677.6	Unit	184.1	Unit	20	166	25	Positive
705.6	Unit	184.1	Unit	20	166	25	Positive
789.7	Unit	184.1	Unit	20	166	25	Positive
675.5	Unit	184.1	Unit	20	166	25	Positive
703.6	Unit	184.1	Unit	20	166	25	Positive

731.6	Unit	184.1	Unit	20	166	25	Positive
759.6	Unit	184.1	Unit	20	166	25	Positive
787.7	Unit	184.1	Unit	20	166	25	Positive
801.7	Unit	184.1	Unit	20	166	25	Positive
815.7	Unit	184.1	Unit	20	166	25	Positive
813.7	Unit	184.1	Unit	20	166	25	Positive
673.5	Unit	184.1	Unit	20	166	25	Positive
701.6	Unit	184.1	Unit	20	166	25	Positive
715.6	Unit	184.1	Unit	20	166	25	Positive
729.6	Unit	184.1	Unit	20	166	25	Positive
727.6	Unit	184.1	Unit	20	166	25	Positive
757.6	Unit	184.1	Unit	20	166	25	Positive
785.7	Unit	184.1	Unit	20	166	25	Positive
799.7	Unit	184.1	Unit	20	166	25	Positive
813.7	Unit	184.1	Unit	20	166	25	Positive
815.7	Unit	184.1	Unit	20	166	25	Positive
785.7	Unit	184.1	Unit	20	166	25	Positive
661.5	Unit	184.1	Unit	20	166	25	Positive
699.5	Unit	184.1	Unit	20	166	25	Positive
753.6	Unit	184.1	Unit	20	166	25	Positive
771.6	Unit	184.1	Unit	20	166	25	Positive
787.7	Unit	184.1	Unit	20	166	25	Positive
783.6	Unit	184.1	Unit	20	166	25	Positive
797.7	Unit	184.1	Unit	20	166	25	Positive
813.7	Unit	184.1	Unit	20	166	25	Positive
827.7	Unit	184.1	Unit	20	166	25	Positive
835.7	Unit	184.1	Unit	20	166	25	Positive
272.3	Unit	254.3	Unit	20	166	8	Positive
300.3	Unit	282.3	Unit	20	166	8	Positive
298.3	Unit	280.3	Unit	20	166	8	Positive
796.8	Unit	264.3	Unit	20	166	56	Positive
780.8	Unit	264.3	Unit	20	166	56	Positive
908.8	Unit	264.3	Unit	20	166	56	Positive
892.8	Unit	264.3	Unit	20	166	56	Positive
906.8	Unit	264.3	Unit	20	166	56	Positive
890.8	Unit	264.3	Unit	20	166	56	Positive
824.8	Unit	551.5	Unit	20	166	21	Positive
822.8	Unit	523.5	Unit	20	166	21	Positive

820.8	Unit	577.6	Unit	20	166	21	Positive
820.8	Unit	547.5	Unit	20	166	21	Positive
820.8	Unit	549.5	Unit	20	166	21	Positive
820.8	Unit	521.5	Unit	20	166	21	Positive
818.8	Unit	547.5	Unit	20	166	21	Positive
818.8	Unit	521.5	Unit	20	166	21	Positive
836.8	Unit	577.5	Unit	20	166	21	Positive
852.8	Unit	551.5	Unit	20	166	21	Positive
850.8	Unit	605.6	Unit	20	166	21	Positive
850.8	Unit	551.5	Unit	20	166	21	Positive
848.8	Unit	547.5	Unit	20	166	21	Positive
848.8	Unit	549.5	Unit	20	166	21	Positive
848.8	Unit	551.5	Unit	20	166	21	Positive
846.8	Unit	603.6	Unit	20	166	21	Positive
846.8	Unit	575.6	Unit	20	166	21	Positive
846.8	Unit	547.5	Unit	20	166	21	Positive
844.8	Unit	599.5	Unit	20	166	21	Positive
878.8	Unit	577.5	Unit	20	166	21	Positive
876.8	Unit	603.6	Unit	20	166	21	Positive
874.8	Unit	603.6	Unit	20	166	21	Positive
874.8	Unit	577.6	Unit	20	166	21	Positive
872.8	Unit	599.6	Unit	20	166	21	Positive
872.8	Unit	573.6	Unit	20	166	21	Positive
908.9	Unit	607.6	Unit	20	166	21	Positive
906.9	Unit	607.6	Unit	20	166	21	Positive
904.9	Unit	603.6	Unit	20	166	21	Positive
902.9	Unit	603.6	Unit	20	166	21	Positive
900.8	Unit	599.5	Unit	20	166	21	Positive
900.9	Unit	603.9	Unit	20	166	21	Positive
898.9	Unit	599.6	Unit	20	166	21	Positive
896.9	Unit	599.6	Unit	20	166	21	Positive
924.9	Unit	603.6	Unit	20	166	21	Positive
920.9	Unit	599.6	Unit	20	166	21	Positive
948.9	Unit	603.7	Unit	20	166	21	Positive
836.8	Unit	563.5	Unit	20	166	21	Positive
866.8	Unit	593.6	Unit	20	166	21	Positive
862.8	Unit	589.6	Unit	20	166	21	Positive

Compou	Compou	Compou	Ion speci	CAS	z	Monoisot	ISTD	Precursor n	MS1 res	Product r	MS2 res	Dwell (m
PC(12:0_	PC(12:0_18:2)		[M+H]+		1		FALSE	702.5069	Unit	184.1	Unit	20
PC(14:0_	PC(14:0_14:0)		[M+H]+		1		FALSE	678.5069	Unit	184.1	Unit	20
PC(14:0_	PC(14:0_18:2)		[M+H]+		1		FALSE	730.5382	Unit	184.1	Unit	20
PC(14:0_	PC(14:0_18:3)		[M+H]+		1		FALSE	728.5225	Unit	184.1	Unit	20
PC(14:0_	PC(14:0_18:3)		[M+H]+		1		FALSE	728.5225	Unit	184.1	Unit	20
PC(14:0_	PC(14:0_20:4)		[M+H]+		1		FALSE	754.5382	Unit	184.1	Unit	20
PC(15:0_	PC(15:0_16:0)		[M+H]+		1		FALSE	720.5538	Unit	184.1	Unit	20
PC(15:0_	PC(15:0_16:0)		[M+H]+		1		FALSE	720.5538	Unit	184.1	Unit	20
PC(15:0_	PC(15:0_16:0)		[M+H]+		1		FALSE	720.5538	Unit	184.1	Unit	20
PC(15:0_	PC(15:0_16:1)		[M+H]+		1		FALSE	718.5382	Unit	184.1	Unit	20
PC(15:0_	PC(15:0_18:1)		[M+H]+		1		FALSE	746.5695	Unit	184.1	Unit	20
PC(15:0_	PC(15:0_18:2)		[M+H]+		1		FALSE	744.5538	Unit	184.1	Unit	20
PC(15:0_	PC(15:0_18:2)		[M+H]+		1		FALSE	744.5538	Unit	184.1	Unit	20
PC(15:0_	PC(15:0_18:3)		[M+H]+		1		FALSE	742.5382	Unit	184.1	Unit	20
PC(15:0_	PC(15:0_20:4)		[M+H]+		1		FALSE	768.5538	Unit	184.1	Unit	20
PC(15:0_	PC(15:0_22:6)		[M+H]+		1		FALSE	792.5538	Unit	184.1	Unit	20
PC(16:0_	PC(16:0_13:0)		[M+H]+		1		FALSE	692.5225	Unit	184.1	Unit	20
PC(16:0_	PC(16:0_14:0)		[M+H]+		1		FALSE	706.5382	Unit	184.1	Unit	20
PC(16:0_	PC(16:0_14:1)		[M+H]+		1		FALSE	704.5225	Unit	184.1	Unit	20
PC(16:0_	PC(16:0_16:0)		[M+H]+		1		FALSE	734.5695	Unit	184.1	Unit	20
PC(16:0_	PC(16:0_16:0)		[M+H]+		1		FALSE	734.5695	Unit	184.1	Unit	20
PC(16:0_	PC(16:0_16:1)		[M+H]+		1		FALSE	732.5538	Unit	184.1	Unit	20
PC(16:0_	PC(16:0_16:1)		[M+H]+		1		FALSE	732.5538	Unit	184.1	Unit	20
PC(16:0_	PC(16:0_17:0)		[M+H]+		1		FALSE	748.5851	Unit	184.1	Unit	20
PC(16:0_	PC(16:0_17:0)		[M+H]+		1		FALSE	748.5851	Unit	184.1	Unit	20
PC(16:0_	PC(16:0_18:1)		[M+H]+		1		FALSE	760.5851	Unit	184.1	Unit	20
PC(16:0_	PC(16:0_18:1)		[M+H]+		1		FALSE	760.5851	Unit	184.1	Unit	20
PC(16:0_	PC(16:0_18:1)		[M+H]+		1		FALSE	760.5851	Unit	184.1	Unit	20
PC(16:0_	PC(16:0_18:1)		[M+H]+		1		FALSE	760.5851	Unit	184.1	Unit	20
PC(16:0_	PC(16:0_18:2)		[M+H]+		1		FALSE	758.5695	Unit	184.1	Unit	20
PC(16:0_	PC(16:0_18:3)		[M+H]+		1		FALSE	756.5538	Unit	184.1	Unit	20
PC(16:0_	PC(16:0_18:3)		[M+H]+		1		FALSE	756.5538	Unit	184.1	Unit	20
PC(16:0_	PC(16:0_18:3)		[M+H]+		1		FALSE	756.5538	Unit	184.1	Unit	20
PC(16:0_	PC(16:0_18:3)		[M+H]+		1		FALSE	756.5538	Unit	184.1	Unit	20
PC(16:0_	PC(16:0_22:6)		[M+H]+		1		FALSE	806.5695	Unit	184.1	Unit	20
PC(16:0_	PC(16:0_8:0)		[M+H]+		1		FALSE	622.4443	Unit	184.1	Unit	20
PC(16:1_	PC(16:1_18:1)		[M+H]+		1		FALSE	758.5695	Unit	184.1	Unit	20

PC(16:1_	PC(16:1_18:1)	[M+H]+	1	FALSE	758.5695	Unit	184.1	Unit	20
PC(16:1_	PC(16:1_20:3)	[M+H]+	1	FALSE	782.5695	Unit	184.1	Unit	20
PC(16:1e	PC(16:1e_18:1)	[M+H]+	1	FALSE	744.5902	Unit	184.1	Unit	20
PC(16:1e	PC(16:1e_20:3)	[M+H]+	1	FALSE	768.5902	Unit	184.1	Unit	20
PC(16:1e	PC(16:1e_22:5)	[M+H]+	1	FALSE	792.5902	Unit	184.1	Unit	20
PC(16:1e	PC(16:1e_22:5)	[M+H]+	1	FALSE	792.5902	Unit	184.1	Unit	20
PC(17:0_	PC(17:0_18:1)	[M+H]+	1	FALSE	774.6008	Unit	184.1	Unit	20
PC(17:0_	PC(17:0_18:1)	[M+H]+	1	FALSE	774.6008	Unit	184.1	Unit	20
PC(17:0_	PC(17:0_18:2)	[M+H]+	1	FALSE	772.5851	Unit	184.1	Unit	20
PC(17:0_	PC(17:0_18:2)	[M+H]+	1	FALSE	772.5851	Unit	184.1	Unit	20
PC(17:0_	PC(17:0_18:2)	[M+H]+	1	FALSE	772.5851	Unit	184.1	Unit	20
PC(17:0_	PC(17:0_18:2)	[M+H]+	1	FALSE	772.5851	Unit	184.1	Unit	20
PC(17:0_	PC(17:0_18:3)	[M+H]+	1	FALSE	770.5695	Unit	184.1	Unit	20
PC(17:0_	PC(17:0_20:3)	[M+H]+	1	FALSE	798.6008	Unit	184.1	Unit	20
PC(17:0_	PC(17:0_20:3)	[M+H]+	1	FALSE	798.6008	Unit	184.1	Unit	20
PC(17:1_	PC(17:1_18:2)	[M+H]+	1	FALSE	770.5695	Unit	184.1	Unit	20
PC(17:1_	PC(17:1_18:2)	[M+H]+	1	FALSE	770.5695	Unit	184.1	Unit	20
PC(18:0_	PC(18:0_16:0)	[M+H]+	1	FALSE	762.6008	Unit	184.1	Unit	20
PC(18:0_	PC(18:0_18:1)	[M+H]+	1	FALSE	788.6164	Unit	184.1	Unit	20
PC(18:0_	PC(18:0_18:1)	[M+H]+	1	FALSE	788.6164	Unit	184.1	Unit	20
PC(18:0_	PC(18:0_18:3)	[M+H]+	1	FALSE	784.5851	Unit	184.1	Unit	20
PC(18:0_	PC(18:0_18:3)	[M+H]+	1	FALSE	784.5851	Unit	184.1	Unit	20
PC(18:0_	PC(18:0_20:4)	[M+H]+	1	FALSE	810.6008	Unit	184.1	Unit	20
PC(18:0_	PC(18:0_22:5)	[M+H]+	1	FALSE	836.6164	Unit	184.1	Unit	20
PC(18:0_	PC(18:0_8:0)	[M+H]+	1	FALSE	650.4756	Unit	184.1	Unit	20
PC(18:1_	PC(18:1_18:2)	[M+H]+	1	FALSE	784.5851	Unit	184.1	Unit	20
PC(18:1_	PC(18:1_20:4)	[M+H]+	1	FALSE	808.5851	Unit	184.1	Unit	20
PC(18:2_	PC(18:2_18:2)	[M+H]+	1	FALSE	782.5695	Unit	184.1	Unit	20
PC(18:2e	PC(18:2e_20:4)	[M+H]+	1	FALSE	792.5902	Unit	184.1	Unit	20
PC(18:3_	PC(18:3_18:2)	[M+H]+	1	FALSE	780.5538	Unit	184.1	Unit	20
PC(19:0_	PC(19:0_20:4)	[M+H]+	1	FALSE	824.6164	Unit	184.1	Unit	20
PC(19:1_	PC(19:1_18:1)	[M+H]+	1	FALSE	800.6164	Unit	184.1	Unit	20
PC(20:0e	PC(20:0e_18:2)	[M+H]+	1	FALSE	800.6528	Unit	184.1	Unit	20
PC(20:5_	PC(20:5_18:2)	[M+H]+	1	FALSE	804.5538	Unit	184.1	Unit	20
PC(22:3)	PC(22:3)	[M+H]+	1	FALSE	588.366	Unit	184.1	Unit	20
PC(22:5_	PC(22:5_14:1)	[M+H]+	1	FALSE	778.5382	Unit	184.1	Unit	20
PC(22:5_	PC(22:5_14:1)	[M+H]+	1	FALSE	778.5382	Unit	184.1	Unit	20
PC(26:1)	PC(26:1)	[M+H]+	1	FALSE	648.4599	Unit	184.1	Unit	20

PC(26:2)	PC(26:2)	[M+H]+	1	FALSE	646.4443	Unit	184.1	Unit	20
PC(31:0)	PC(31:0)	[M+H]+	1	FALSE	720.5538	Unit	184.1	Unit	20
PC(31:1)	PC(31:1)	[M+H]+	1	FALSE	718.5382	Unit	184.1	Unit	20
PC(31:1e	PC(31:1e)	[M+H]+	1	FALSE	718.5382	Unit	184.1	Unit	20
PC(32:1)	PC(32:1)	[M+H]+	1	FALSE	732.5538	Unit	184.1	Unit	20
PC(32:1)	PC(32:1)	[M+H]+	1	FALSE	732.5538	Unit	184.1	Unit	20
PC(32:1)	PC(32:1)	[M+H]+	1	FALSE	732.5538	Unit	184.1	Unit	20
PC(33:2e	PC(33:2e)	[M+H]+	1	FALSE	744.5538	Unit	184.1	Unit	20
PC(33:3)	PC(33:3)	[M+H]+	1	FALSE	742.5382	Unit	184.1	Unit	20
PC(33:3e	PC(33:3e)	[M+H]+	1	FALSE	742.5382	Unit	184.1	Unit	20
PC(34:0)	PC(34:0)	[M+H]+	1	FALSE	762.6008	Unit	184.1	Unit	20
PC(34:0)	PC(34:0)	[M+H]+	1	FALSE	762.6008	Unit	184.1	Unit	20
PC(34:1)	PC(34:1)	[M+H]+	1	FALSE	760.5851	Unit	184.1	Unit	20
PC(34:2)	PC(34:2)	[M+H]+	1	FALSE	758.5695	Unit	184.1	Unit	20
PC(35:2e	PC(35:2e)	[M+H]+	1	FALSE	772.5851	Unit	184.1	Unit	20
PC(36:2)	PC(36:2)	[M+H]+	1	FALSE	786.6008	Unit	184.1	Unit	20
PC(36:2)	PC(36:2)	[M+H]+	1	FALSE	786.6008	Unit	184.1	Unit	20
PC(36:2)	PC(36:2)	[M+H]+	1	FALSE	786.6008	Unit	184.1	Unit	20
PC(36:5)	PC(36:5)	[M+H]+	1	FALSE	780.5538	Unit	184.1	Unit	20
PC(36:5)	PC(36:5)	[M+H]+	1	FALSE	780.5538	Unit	184.1	Unit	20
PC(37:4e	PC(37:4e)	[M+H]+	1	FALSE	796.5851	Unit	184.1	Unit	20
PC(37:5)	PC(37:5)	[M+H]+	1	FALSE	794.5695	Unit	184.1	Unit	20
PC(37:5e	PC(37:5e)	[M+H]+	1	FALSE	794.5695	Unit	184.1	Unit	20
PC(37:5e	PC(37:5e)	[M+H]+	1	FALSE	794.5695	Unit	184.1	Unit	20
PC(37:5e	PC(37:5e)	[M+H]+	1	FALSE	794.5695	Unit	184.1	Unit	20
PC(37:5e	PC(37:5e)	[M+H]+	1	FALSE	794.5695	Unit	184.1	Unit	20
PC(38:4)	PC(38:4)	[M+H]+	1	FALSE	810.6008	Unit	184.1	Unit	20
PC(38:5)	PC(38:5)	[M+H]+	1	FALSE	808.5851	Unit	184.1	Unit	20
PC(38:5)	PC(38:5)	[M+H]+	1	FALSE	808.5851	Unit	184.1	Unit	20
PC(38:7)	PC(38:7)	[M+H]+	1	FALSE	804.5538	Unit	184.1	Unit	20
PC(38:7)	PC(38:7)	[M+H]+	1	FALSE	804.5538	Unit	184.1	Unit	20
PC(40:3e	PC(40:3e)	[M+H]+	1	FALSE	840.6477	Unit	184.1	Unit	20
PC(40:4)	PC(40:4)	[M+H]+	1	FALSE	838.6321	Unit	184.1	Unit	20
PC(44:6e	PC(44:6e)	[M+H]+	1	FALSE	890.6634	Unit	184.1	Unit	20

Fragmen	CE (V)	Polarity
166	17	Positive
166	17	Positive
166	17	Positive
166	17	Positive
166	17	Positive
166	17	Positive
166	17	Positive
166	17	Positive
166	17	Positive
166	17	Positive
166	17	Positive
166	17	Positive
166	17	Positive
166	17	Positive
166	17	Positive
166	17	Positive
166	17	Positive
166	17	Positive
166	17	Positive
166	17	Positive
166	17	Positive
166	17	Positive
166	17	Positive
166	17	Positive
166	17	Positive
166	17	Positive
166	17	Positive
166	17	Positive
166	17	Positive
166	17	Positive
166	17	Positive
166	17	Positive
166	17	Positive
166	17	Positive
166	17	Positive
166	17	Positive
166	17	Positive

166	17	Positive
166	17	Positive
166	17	Positive
166	17	Positive
166	17	Positive
166	17	Positive
166	17	Positive
166	17	Positive
166	17	Positive
166	17	Positive
166	17	Positive
166	17	Positive
166	17	Positive
166	17	Positive
166	17	Positive
166	17	Positive
166	17	Positive
166	17	Positive
166	17	Positive
166	17	Positive
166	17	Positive
166	17	Positive
166	17	Positive
166	17	Positive
166	17	Positive
166	17	Positive
166	17	Positive
166	17	Positive
166	17	Positive
166	17	Positive
166	17	Positive
166	17	Positive
166	17	Positive
166	17	Positive
166	17	Positive
166	17	Positive
166	17	Positive
166	17	Positive

166	17	Positive
166	17	Positive
166	17	Positive
166	17	Positive
166	17	Positive
166	17	Positive
166	17	Positive
166	17	Positive
166	17	Positive
166	17	Positive
166	17	Positive
166	17	Positive
166	17	Positive
166	17	Positive
166	17	Positive
166	17	Positive
166	17	Positive
166	17	Positive
166	17	Positive
166	17	Positive
166	17	Positive
166	17	Positive
166	17	Positive
166	17	Positive
166	17	Positive
166	17	Positive
166	17	Positive
166	17	Positive
166	17	Positive
166	17	Positive
166	17	Positive
166	17	Positive
166	17	Positive
166	17	Positive

Compound group	Compounc	Compounc	Ion species	CAS	z	Monoisoto|	ISTD?	Precursor n	MS1 res	Product m/
LPE(P-20:0)	LPE(P-20:0	C42H82NC	[M+H]+		1		FALSE	494.3	Unit	322.4
LPI(18:0) [sn1]	LPI(18:0) [s	C42H82NC	[M+NH4]+		1		FALSE	618.3	Unit	341.3
LPI(18:1) [sn1]	LPI(18:1) [s	C42H82NC	[M+NH4]+		1		FALSE	616.3	Unit	339.3
LPI(18:1) [sn2]	LPI(18:1) [s	C42H82NC	[M+NH4]+		1		FALSE	616.3	Unit	339.3
LPI(18:2) [sn1]	LPI(18:2) [s	C42H82NC	[M+NH4]+		1		FALSE	614.3	Unit	337.3
LPI(20:4) [sn1]	LPI(20:4) [s	C42H82NC	[M+NH4]+		1		FALSE	638.3	Unit	361.3
PC(14:0_16:0)	PC(14:0_16:0)		[M+H]+		1		FALSE	706.5	Unit	184.1
PC(14:0_20:4)	PC(14:0_20:4)		[M+H]+		1		FALSE	754.5	Unit	184.1
PC(14:0_22:6)	PC(14:0_22:6)		[M+H]+		1		FALSE	778.5	Unit	184.1
PC(15-MHDA_18:1)	PC(15-MHDA_18:1)		[M+H]+		1		FALSE	774.6	Unit	184.1
PC(15-MHDA_22:6)	PC(15-MHDA_22:6)		[M+H]+		1		FALSE	820.6	Unit	184.1
PC(16:0/16:0)	PC(16:0/16:0)		[M+H]+		1		FALSE	734.6	Unit	184.1
PC(16:0_18:0)	PC(16:0_18:0)		[M+H]+		1		FALSE	762.6	Unit	184.1
PC(16:0_18:1)	PC(16:0_18:1)		[M+H]+		1		FALSE	760.6	Unit	184.1
PC(16:0_18:2)	PC(16:0_18:2)		[M+H]+		1		FALSE	758.6	Unit	184.1
PC(16:0_18:3) (a)	PC(16:0_18:3) (a)		[M+H]+		1		FALSE	756.6	Unit	184.1
PC(16:0_18:3) (b)	PC(16:0_18:3) (b)		[M+H]+		1		FALSE	756.6	Unit	184.1
PC(16:0_20:3) (a)	PC(16:0_20:3) (a)		[M+H]+		1		FALSE	784.6	Unit	184.1
PC(16:0_20:4)	PC(16:0_20:4)		[M+H]+		1		FALSE	782.6	Unit	184.1
PC(16:0_20:5)	PC(16:0_20:5)		[M+H]+		1		FALSE	780.6	Unit	184.1
PC(16:0_22:6)	PC(16:0_22:6)		[M+H]+		1		FALSE	806.6	Unit	184.1
PC(16:1_18:2)	PC(16:1_18:2)		[M+H]+		1		FALSE	756.6	Unit	184.1
PC(16:1_20:4)	PC(16:1_20:4)		[M+H]+		1		FALSE	780.6	Unit	184.1
PC(16:1_22:6)	PC(16:1_22:6)		[M+H]+		1		FALSE	804.6	Unit	184.1
PC(18:0_18:1)	PC(18:0_18:1)		[M+H]+		1		FALSE	788.6	Unit	184.1
PC(18:0_18:2)	PC(18:0_18:2)		[M+H]+		1		FALSE	786.6	Unit	184.1
PC(18:0_20:3)	PC(18:0_20:3)		[M+H]+		1		FALSE	812.6	Unit	184.1
PC(18:0_20:4)	PC(18:0_20:4)		[M+H]+		1		FALSE	810.6	Unit	184.1
PC(18:0_22:4)	PC(18:0_22:4)		[M+H]+		1		FALSE	838.6	Unit	184.1
PC(18:0_22:5) (n3)/PC(20:1_20:4)	PC(18:0_22:5) (n3)/PC		[M+H]+		1		FALSE	836.6	Unit	184.1
PC(18:0_22:5) (n6)	PC(18:0_22:5) (n6)		[M+H]+		1		FALSE	836.6	Unit	184.1
PC(18:0_22:6)	PC(18:0_22:6)		[M+H]+		1		FALSE	834.6	Unit	184.1
PC(18:1_18:1)	PC(18:1_18:1)		[M+H]+		1		FALSE	786.6	Unit	184.1
PC(18:1_18:2)	PC(18:1_18:2)		[M+H]+		1		FALSE	784.6	Unit	184.1
PC(18:1_20:3)	PC(18:1_20:3)		[M+H]+		1		FALSE	810.6	Unit	184.1
PC(18:1_22:6) (a)	PC(18:1_22:6) (a)		[M+H]+		1		FALSE	832.6	Unit	184.1
PC(18:1_22:6) (b)	PC(18:1_22:6) (b)		[M+H]+		1		FALSE	832.6	Unit	184.1

PC(18:2_18:2)	PC(18:2_18:2)	[M+H]+	1	FALSE	782.6	Unit	184.1
PC(18:2_20:5)	PC(18:2_20:5)	[M+H]+	1	FALSE	804.6	Unit	184.1
PC(20:0_20:4)	PC(20:0_20:4)	[M+H]+	1	FALSE	838.6	Unit	184.1
PC(28:0)	PC(28:0)	[M+H]+	1	FALSE	678.5	Unit	184.1
PC(32:1)	PC(32:1)	[M+H]+	1	FALSE	732.6	Unit	184.1
PC(32:2)	PC(32:2)	[M+H]+	1	FALSE	730.5	Unit	184.1
PC(33:0) (a)	PC(33:0) (a)	[M+H]+	1	FALSE	748.6	Unit	184.1
PC(34:5)	PC(34:5)	[M+H]+	1	FALSE	752.5	Unit	184.1
PC(36:0)	PC(36:0)	[M+H]+	1	FALSE	790.6	Unit	184.1
PC(36:6) (a)	PC(36:6) (a)	[M+H]+	1	FALSE	778.5	Unit	184.1
PC(38:2)	PC(38:2)	[M+H]+	1	FALSE	814.6	Unit	184.1
PC(38:4) (b)	PC(38:4) (b)	[M+H]+	1	FALSE	810.6	Unit	184.1
PC(38:5) (a)	PC(38:5) (a)	[M+H]+	1	FALSE	808.6	Unit	184.1
PC(38:5) (b)	PC(38:5) (b)	[M+H]+	1	FALSE	808.6	Unit	184.1
PC(38:6) (a)	PC(38:6) (a)	[M+H]+	1	FALSE	806.6	Unit	184.1
PC(38:7) (c)	PC(38:7) (c)	[M+H]+	1	FALSE	804.6	Unit	184.1
PC(40:7) (a)	PC(40:7) (a)	[M+H]+	1	FALSE	832.6	Unit	184.1
PC(40:8)	PC(40:8)	[M+H]+	1	FALSE	830.6	Unit	184.1
PC(O-16:0/16:0)	PC(O-16:0/16:0)	[M+H]+	1	FALSE	720.6	Unit	184.1
PC(O-16:0/20:3)	PC(O-16:0/20:3)	[M+H]+	1	FALSE	770.6	Unit	184.1
PC(O-16:0/20:4)	PC(O-16:0/20:4)	[M+H]+	1	FALSE	768.6	Unit	184.1
PC(O-16:0/22:6)	PC(O-16:0/22:6)	[M+H]+	1	FALSE	792.6	Unit	184.1
PC(O-18:0/18:1)	PC(O-18:0/18:1)	[M+H]+	1	FALSE	774.6	Unit	184.1
PC(O-18:0/18:2)	PC(O-18:0/18:2)	[M+H]+	1	FALSE	772.6	Unit	184.1
PC(O-18:0/20:4)	PC(O-18:0/20:4)	[M+H]+	1	FALSE	796.6	Unit	184.1
PC(O-18:0/22:6)	PC(O-18:0/22:6)	[M+H]+	1	FALSE	820.6	Unit	184.1
PC(O-18:1/18:1)	PC(O-18:1/18:1)	[M+H]+	1	FALSE	772.6	Unit	184.1
PC(O-18:1/18:2)	PC(O-18:1/18:2)	[M+H]+	1	FALSE	770.6	Unit	184.1
PC(O-32:1)	PC(O-32:1)	[M+H]+	1	FALSE	718.5	Unit	184.1
PC(O-32:2)	PC(O-32:2)	[M+H]+	1	FALSE	716.6	Unit	184.1
PC(O-34:1)	PC(O-34:1)	[M+H]+	1	FALSE	746.6	Unit	184.1
PC(O-34:2)	PC(O-34:2)	[M+H]+	1	FALSE	744.6	Unit	184.1
PC(O-34:4)	PC(O-34:4)	[M+H]+	1	FALSE	740.6	Unit	184.1
PC(O-35:4)	PC(O-35:4)	[M+H]+	1	FALSE	754.5	Unit	184.1
PC(O-36:0)	PC(O-36:0)	[M+H]+	1	FALSE	776.6	Unit	184.1
PC(O-36:5)	PC(O-36:5)	[M+H]+	1	FALSE	766.5	Unit	184.1
PC(O-38:5)	PC(O-38:5)	[M+H]+	1	FALSE	794.6	Unit	184.1
PC(O-40:5)	PC(O-40:5)	[M+H]+	1	FALSE	822.6	Unit	184.1

PC(O-40:7) (a)	PC(O-40:7) (a)	[M+H]+	1	FALSE	818.6	Unit	184.1
PC(O-40:7) (b)	PC(O-40:7) (b)	[M+H]+	1	FALSE	818.6	Unit	184.1
PC(P-16:0/14:0)	PC(P-16:0/14:0)	[M+H]+	1	FALSE	690.4	Unit	184.1
PC(P-16:0/16:0)	PC(P-16:0/16:0)	[M+H]+	1	FALSE	718.5	Unit	184.1
PC(P-16:0/16:1)	PC(P-16:0/16:1)	[M+H]+	1	FALSE	716.6	Unit	184.1
PC(P-16:0/18:0)	PC(P-16:0/18:0)	[M+H]+	1	FALSE	746.6	Unit	184.1
PC(P-16:0/18:1)	PC(P-16:0/18:1)	[M+H]+	1	FALSE	744.6	Unit	184.1
PC(P-16:0/18:2)	PC(P-16:0/18:2)	[M+H]+	1	FALSE	742.5	Unit	184.1
PC(P-16:0/18:3)	PC(P-16:0/18:3)	[M+H]+	1	FALSE	740.6	Unit	184.1
PC(P-16:0/20:4)	PC(P-16:0/20:4)	[M+H]+	1	FALSE	766.5	Unit	184.1
PC(P-16:0/20:5)	PC(P-16:0/ C42H82NC	[M+H]+	1	FALSE	764.6	Unit	184.1
PC(P-16:0/22:6)	PC(P-16:0/ C42H82NC	[M+H]+	1	FALSE	790.6	Unit	184.1
PC(P-18:0/18:2)	PC(P-18:0/18:2)	[M+H]+	1	FALSE	770.6	Unit	184.1
PC(P-18:0/20:4)	PC(P-18:0/ C42H82NC	[M+H]+	1	FALSE	794.6	Unit	184.1
PC(P-18:0/22:5)	PC(P-18:0/ C42H82NC	[M+H]+	1	FALSE	820.6	Unit	184.1
PC(P-18:0/22:6)	PC(P-18:0/ C42H82NC	[M+H]+	1	FALSE	818.6	Unit	184.1
PC(P-18:1/18:1)	PC(P-18:1/18:1)	[M+H]+	1	FALSE	770.6	Unit	184.1
PC(P-18:1/22:6)	PC(P-18:1/ C42H82NC	[M+H]+	1	FALSE	816.6	Unit	184.1
PC(P-20:0/20:4)	PC(P-20:0/ C42H82NC	[M+H]+	1	FALSE	822.6	Unit	184.1
PC(P-36:3)	PC(P-36:3)	[M+H]+	1	FALSE	768.5	Unit	184.1
PC(P-38:5) (a)	PC(P-38:5) C42H82NC	[M+H]+	1	FALSE	792.6	Unit	184.1
PC(P-38:5) (b)	PC(P-38:5) C42H82NC	[M+H]+	1	FALSE	792.6	Unit	184.1
PE(15-MHDA_18:1)	PE(15-MHD C42H82NC	[M+H]+	1	FALSE	732.6	Unit	591.5
PE(15-MHDA_18:2)	PE(15-MHD C42H82NC	[M+H]+	1	FALSE	730.5	Unit	589.5
PE(15-MHDA_20:4)	PE(15-MHD C42H82NC	[M+H]+	1	FALSE	754.6	Unit	613.5
PE(15-MHDA_22:6)	PE(15-MHD C42H82NC	[M+H]+	1	FALSE	778.5	Unit	637.5

MS2 res	Dwell (ms)	Fragmentor	CE (V)	Polarity
Unit	20	166	19	Positive
Unit	20	166	17	Positive
Unit	20	166	17	Positive
Unit	20	166	17	Positive
Unit	20	166	17	Positive
Unit	20	166	17	Positive
Unit	20	166	21	Positive
Unit	20	166	21	Positive
Unit	20	166	21	Positive
Unit	20	166	21	Positive
Unit	20	166	21	Positive
Unit	20	166	21	Positive
Unit	20	166	21	Positive
Unit	20	166	21	Positive
Unit	20	166	21	Positive
Unit	20	166	21	Positive
Unit	20	166	21	Positive
Unit	20	166	21	Positive
Unit	20	166	21	Positive
Unit	20	166	21	Positive
Unit	20	166	21	Positive
Unit	20	166	21	Positive
Unit	20	166	21	Positive
Unit	20	166	21	Positive
Unit	20	166	21	Positive
Unit	20	166	21	Positive
Unit	20	166	21	Positive
Unit	20	166	21	Positive
Unit	20	166	21	Positive
Unit	20	166	21	Positive
Unit	20	166	21	Positive
Unit	20	166	21	Positive
Unit	20	166	21	Positive
Unit	20	166	21	Positive
Unit	20	166	21	Positive
Unit	20	166	21	Positive
Unit	20	166	21	Positive

Unit	20	166	21	Positive
Unit	20	166	21	Positive
Unit	20	166	21	Positive
Unit	20	166	21	Positive
Unit	20	166	21	Positive
Unit	20	166	21	Positive
Unit	20	166	21	Positive
Unit	20	166	21	Positive
Unit	20	166	21	Positive
Unit	20	166	21	Positive
Unit	20	166	21	Positive
Unit	20	166	21	Positive
Unit	20	166	21	Positive
Unit	20	166	21	Positive
Unit	20	166	21	Positive
Unit	20	166	21	Positive
Unit	20	166	21	Positive
Unit	20	166	21	Positive
Unit	20	166	21	Positive
Unit	20	166	21	Positive
Unit	20	166	21	Positive
Unit	20	166	21	Positive
Unit	20	166	21	Positive
Unit	20	166	21	Positive
Unit	20	166	21	Positive
Unit	20	166	21	Positive
Unit	20	166	21	Positive
Unit	20	166	21	Positive
Unit	20	166	21	Positive
Unit	20	166	21	Positive
Unit	20	166	21	Positive
Unit	20	166	21	Positive
Unit	20	166	21	Positive
Unit	20	166	21	Positive
Unit	20	166	21	Positive
Unit	20	166	21	Positive
Unit	20	166	21	Positive
Unit	20	166	21	Positive

Unit	20	166	21	Positive
Unit	20	166	21	Positive
Unit	20	166	21	Positive
Unit	20	166	21	Positive
Unit	20	166	21	Positive
Unit	20	166	21	Positive
Unit	20	166	21	Positive
Unit	20	166	21	Positive
Unit	20	166	21	Positive
Unit	20	166	21	Positive
Unit	20	166	21	Positive
Unit	20	166	21	Positive
Unit	20	166	21	Positive
Unit	20	166	21	Positive
Unit	20	166	21	Positive
Unit	20	166	21	Positive
Unit	20	166	21	Positive
Unit	20	166	21	Positive
Unit	20	166	21	Positive
Unit	20	166	21	Positive
Unit	20	166	21	Positive
Unit	20	166	21	Positive
Unit	20	166	17	Positive
Unit	20	166	17	Positive
Unit	20	166	17	Positive
Unit	20	166	17	Positive

Compound group	Compound formula	Ion speci	CAS	z	Monoisot	ISTD	Precurso	MS1 res	Product r
DAG 16:0/18:0		[M+H]+		1		FALSE	614.5	Unit	313
DAG 16:0/18:1		[M+H]+		1		FALSE	612.5	Unit	313
DAG 16:0/20:4		[M+H]+		1		FALSE	634.5	Unit	313
DAG 18:0/18:0		[M+H]+		1		FALSE	642.5	Unit	341
DAG 18:0/18:1		[M+H]+		1		FALSE	640.5	Unit	341
DAG 18:0/20:4-d8	C42H82NO10P	[M+NH4]+		1		FALSE	670.5	Unit	341
DAG 18:0/22:6	C42H82NO10P	[M+NH4]+		1		FALSE	686.5	Unit	341
DAG 18:0/24:0		[M+H]+		1		FALSE	662.5	Unit	341
DAG 18:1/18:1		[M+H]+		1		FALSE	638.5	Unit	339
DAG 18:1/20:4		[M+H]+		1		FALSE	660.5	Unit	339
DAG 18:1/22:6		[M+H]+		1		FALSE	684.5	Unit	339
PIP2 26:0	C42H82NO10P	[M+H]+		1		FALSE	885	Unit	401
PIP2 28:0	C42H82NO10P	[M+H]+		1		FALSE	913	Unit	401
PIP2 28:1	C42H82NO10P	[M+H]+		1		FALSE	911	Unit	401
PIP2 30:0	C42H82NO10P	[M+H]+		1		FALSE	941	Unit	401
PIP2 30:1	C42H82NO10P	[M+H]+		1		FALSE	939	Unit	401
PIP2 32:0	C42H82NO10P	[M+H]+		1		FALSE	969	Unit	401
PIP2 32:1	C42H82NO10P	[M+H]+		1		FALSE	967	Unit	401
PIP2 32:2	C42H82NO10P	[M+H]+		1		FALSE	965	Unit	401
PIP2 34:0	C42H82NO10P	[M+NH4]+		1		FALSE	997	Unit	401
PIP2 34:1	C42H82NO10P	[M+NH4]+		1		FALSE	995	Unit	401
PIP2 34:2	C42H82NO10P	[M+H]+		1		FALSE	993	Unit	401
PIP2 36:0	C42H82NO10P	[M+NH4]+		1		FALSE	1025	Unit	401
PIP2 36:1	C42H82NO10P	[M+NH4]+		1		FALSE	1023	Unit	401
PIP2 36:2	C42H82NO10P	[M+NH4]+		1		FALSE	1021	Unit	401
PIP2 36:4	C42H82NO10P	[M+NH4]+		1		FALSE	1017	Unit	401
PIP2 38:4	C42H82NO10P	[M+NH4]+		1		FALSE	1045	Unit	401
PIP2 40:6	C42H82NO10P	[M+NH4]+		1		FALSE	1069	Unit	401
MGDG(16:0_18:1)	C42H82NO10P	[M-H]-		1		FALSE	755.57	Unit	331.3
MGDG(16:0_18:1)	C42H82NO10P	[M-H]-		1		FALSE	755.57	Unit	357.3
MGDG(16:0_18:2)	C42H82NO10P	[M-H]-		1		FALSE	753.55	Unit	331.3
MGDG(16:0_18:2)	C42H82NO10P	[M-H]-		1		FALSE	753.55	Unit	355.3
MGDG(18:1_18:1)	C42H82NO10P	[M-H]-		1		FALSE	781.58	Unit	357.3
MGDG(18:1_18:1)	C42H82NO10P	[M-H]-		1		FALSE	781.58	Unit	357.3
MGDG(18:1_18:2)	C42H82NO10P	[M-H]-		1		FALSE	779.57	Unit	357.3
MGDG(18:1_18:2)	C42H82NO10P	[M-H]-		1		FALSE	779.57	Unit	355.3
MGDG(42:4)	C42H82NO10P	[M-H]-		1		FALSE	861.65	Unit	323.2
