# Supplementary material for: A lipid compendium of a metabolically compromised bacterium provides insights into lipid acquisition, biosynthesis, and metabolism
Source: bioRxiv. 2026 May 23:2026.05.22.727245. Preprint. [Version 1] doi: 10.64898/2026.05.22.727245 (PMC13228262; doi:10.64898/2026.05.22.727245)
Supplement: Supplement 5 [file NIHPP2026.05.22.727245V1-supplement-5.pdf]

bioRxiv preprint doi: https://doi.org/10.64898/2026.05.22.727245; this version posted May 23, 2026. The copyright holder for this preprint (which was not certified by peer review) is the author/funder, who has granted bioRxiv a license to display the preprint in perpetuity. It is made available under a CC-BY 4.0 International license.

Supporting Information for

A lipid compendium of a metabolically compromised bacterium provides insights into lipid acquisition, biosynthesis, and metabolism

Poulami Chatterjee,[1,2] Hyejin Esther Shin,[2,3] Miles I. Tuncel,[2,4] Isaac A. Paddy,[2,5] Alysha K. Lee,[6] Joshua McCausland,[2,3] Paula V. Welander,[6] Christine Jacobs-Wagner,[2,3,4,7] & Laura M. K. Dassama[1,2,4,*]

[1]Department of Chemistry, Stanford University, Stanford, CA

[2]Sarafan ChEM-H Institute, Stanford University, Stanford, CA

[3]Howard Hughes Medical Institute, Stanford University, Stanford, CA

[4]Department of Microbiology & Immunology, Stanford School of Medicine, Stanford, CA

[5]Department of Chemical & Systems Biology, Stanford School of Medicine, Stanford, CA

[6]Department of Earth Systems Science, Stanford Doerr School of Sustainability, Stanford, CA

[7]Department of Biology, Stanford University, Stanford, CA

*Correspondence to: dassama@stanford.edu

bioRxiv preprint doi: https://doi.org/10.64898/2026.05.22.727245; this version posted May 23, 2026. The copyright holder for this preprint (which was not certified by peer review) is the author/funder, who has granted bioRxiv a license to display the preprint in perpetuity. It is made available under a CC-BY 4.0 International license.

Supporting figures

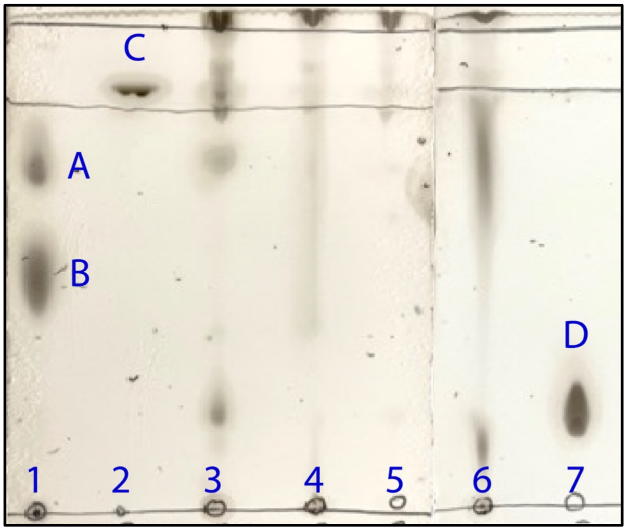

Fig. S1. Qualitative analysis of lipids was performed in rabbit serum, complete medium, spent medium, *Borrelia burgdorferi*, and lipid standards using Thin Layer Chromatography (TLC). The lanes are designated as follows: total polar lipid extract of *Escherichia coli* (standard lipids are annotated as observed in the #Avanti research (1), cholesterol standard (2), total lipid from rabbit serum (3), total lipid from complete medium (4), total lipid from spent medium (5), total lipid from *Borrelia burgdorferi* (6), and phosphatidylcholine standard (7). On the TLC plate, phosphatidylglycerol is labeled A, phosphatidylethanolamine B, cholesterol C, and phosphatidylcholine D.

bioRxiv preprint doi: https://doi.org/10.64898/2026.05.22.727245; this version posted May 23, 2026. The copyright holder for this preprint (which was not certified by peer review) is the author/funder, who has granted bioRxiv a license to display the preprint in perpetuity. It is made available under a CC-BY 4.0 International license.

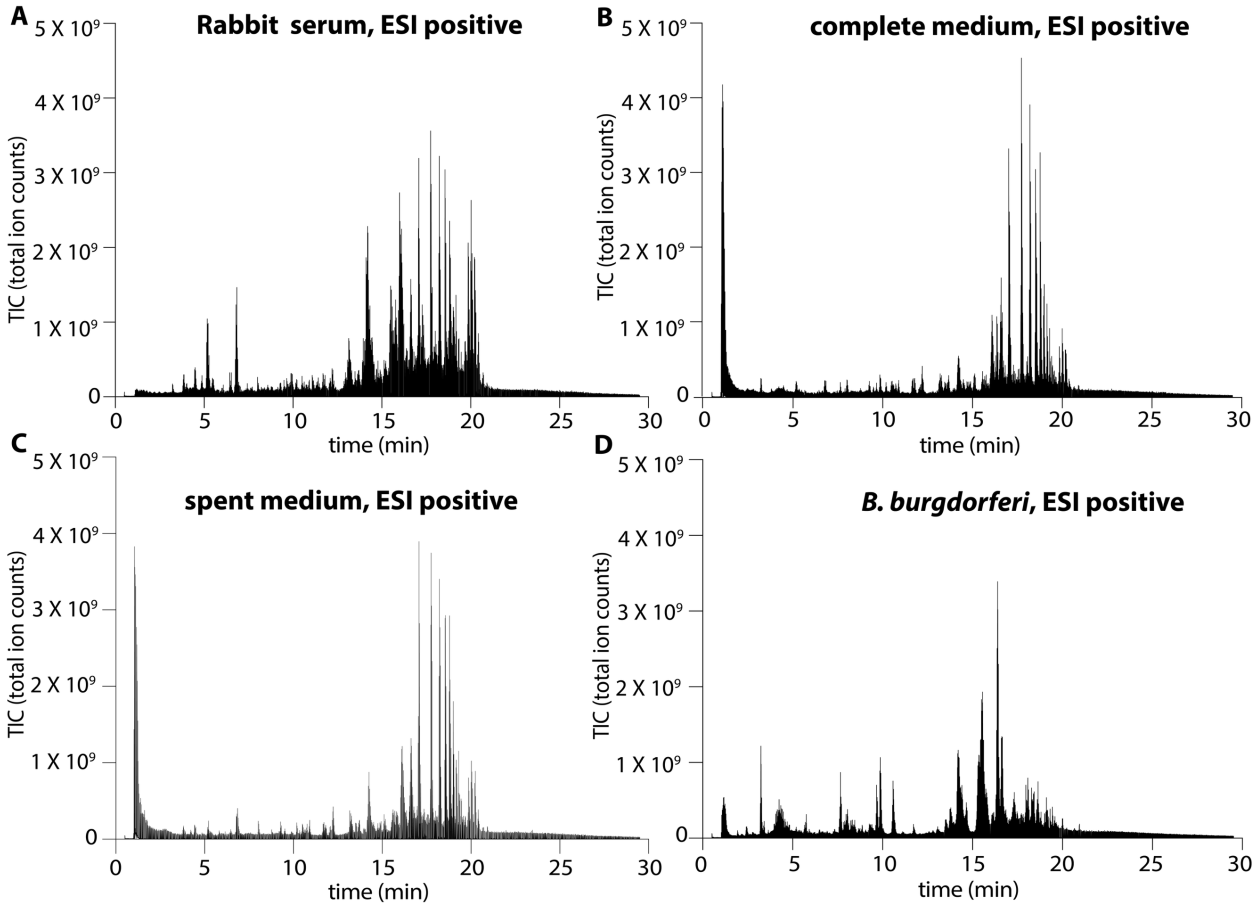

Fig. S2. Untargeted lipidomic profiling of rabbit serum, growth media, and *B. burgdorferi*. Representative total ion chromatograms acquired in positive electrospray ionization (ESI positive) mode are shown for **(A)** rabbit serum, **(B)** complete medium, **(C)** spent medium, and **(D)** *B. burgdorferi*.

bioRxiv preprint doi: https://doi.org/10.64898/2026.05.22.727245; this version posted May 23, 2026. The copyright holder for this preprint (which was not certified by peer review) is the author/funder, who has granted bioRxiv a license to display the preprint in perpetuity. It is made available under a CC-BY 4.0 International license.

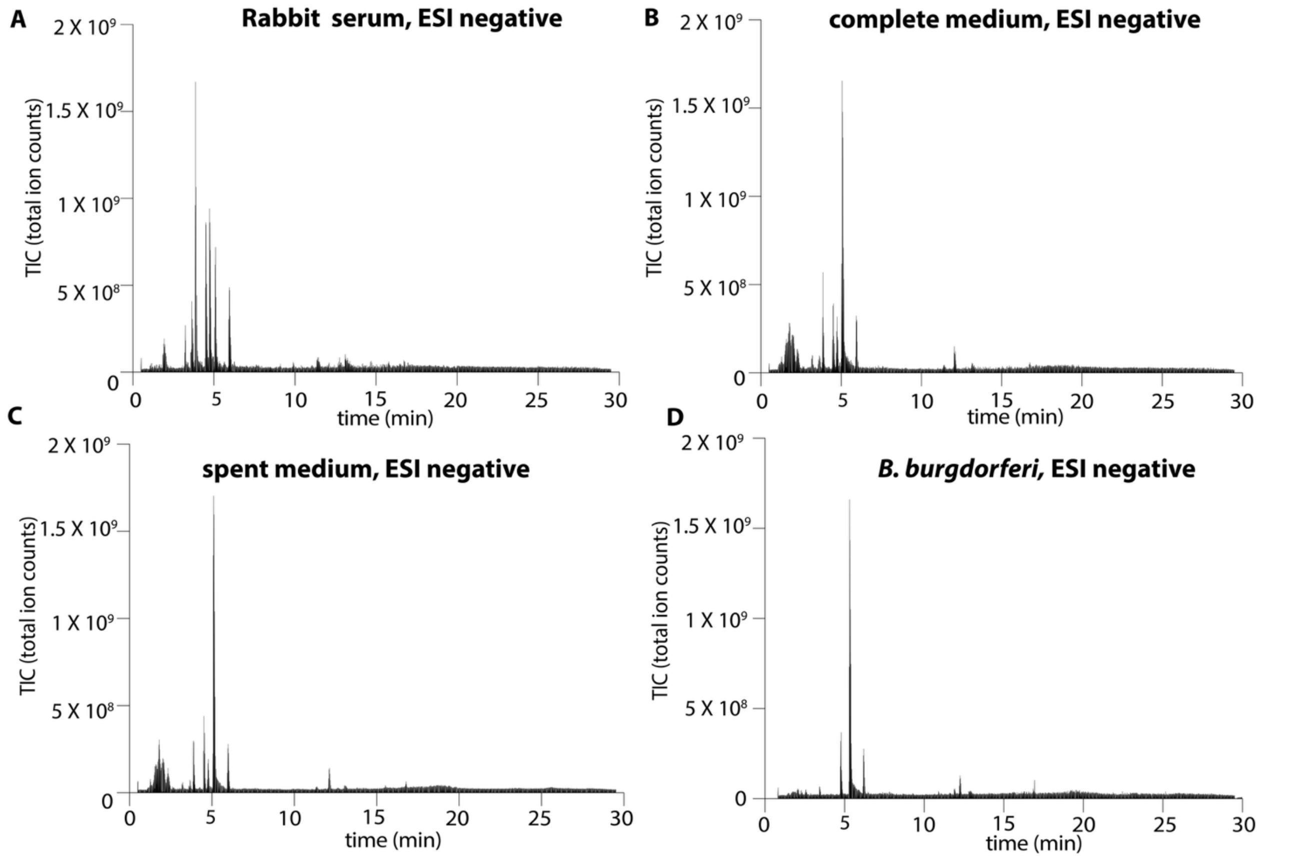

Fig. S3. Untargeted lipidomic profiling of rabbit serum, growth media, and *B. burgdorferi*. Representative total ion chromatograms acquired in negative electrospray ionization (ESI negative) mode are shown for **(A)** rabbit serum, **(B)** complete medium, **(C)** spent medium, and **(D)** *B. burgdorferi*.

bioRxiv preprint doi: https://doi.org/10.64898/2026.05.22.727245; this version posted May 23, 2026. The copyright holder for this preprint (which was not certified by peer review) is the author/funder, who has granted bioRxiv a license to display the preprint in perpetuity. It is made available under a CC-BY 4.0 International license.

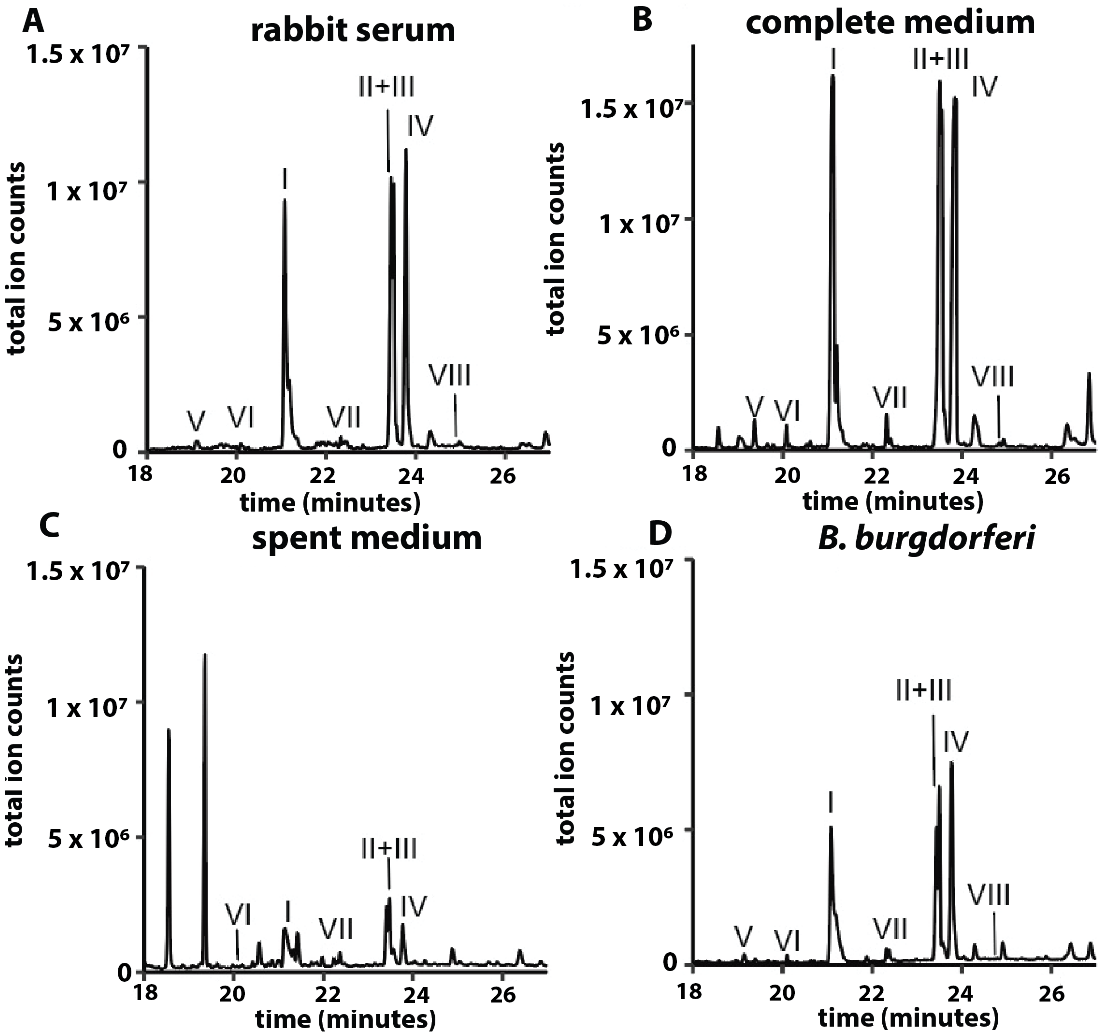

Fig. S4. Fatty acid profiling of rabbit serum, complete medium, spent medium, and *Borrelia burgdorferi*. Samples were analyzed via GC. Peaks were annotated on the basis of retention time and corresponding lipid standards. The identified fatty acids were: I: Palmitic acid, II: Stearic acid, III: Oleic acid, IV: Linoleic acid, V: Myristic acid, VI: Pentadecanoic acid, VII: Heptadecanoic acid, VIII: Arachidonic acid.

bioRxiv preprint doi: https://doi.org/10.64898/2026.05.22.727245; this version posted May 23, 2026. The copyright holder for this preprint (which was not certified by peer review) is the author/funder, who has granted bioRxiv a license to display the preprint in perpetuity. It is made available under a CC-BY 4.0 International license.

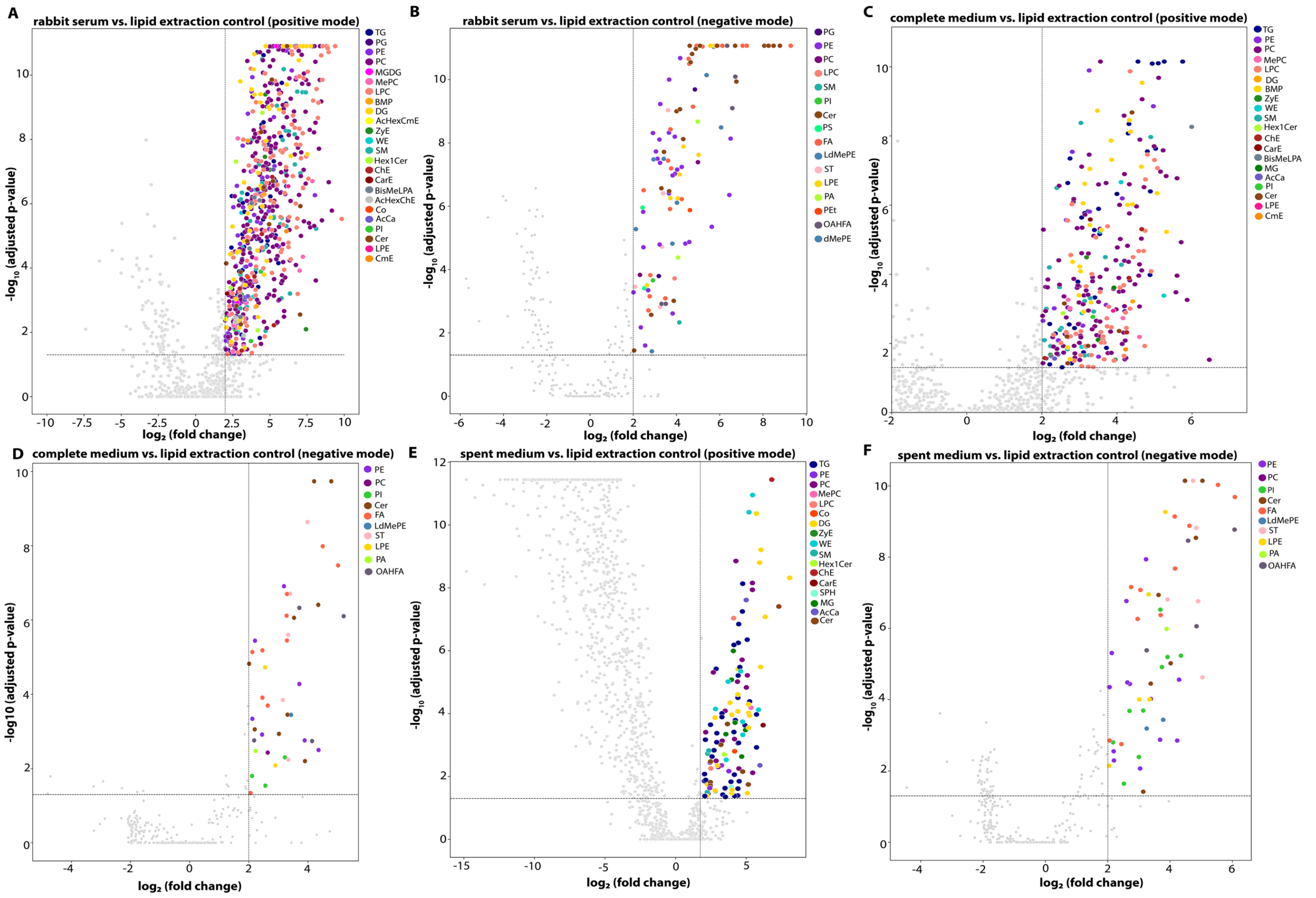

Fig. S5. Untargeted lipidomics of rabbit serum and media to assess lipid abundance and distribution. Volcano plots show lipids significantly enriched in rabbit serum **(A, B)**, complete medium **(C, D)** and spent medium **(E, F)** compared to a lipid extraction control.

bioRxiv preprint doi: https://doi.org/10.64898/2026.05.22.727245; this version posted May 23, 2026. The copyright holder for this preprint (which was not certified by peer review) is the author/funder, who has granted bioRxiv a license to display the preprint in perpetuity. It is made available under a CC-BY 4.0 International license.

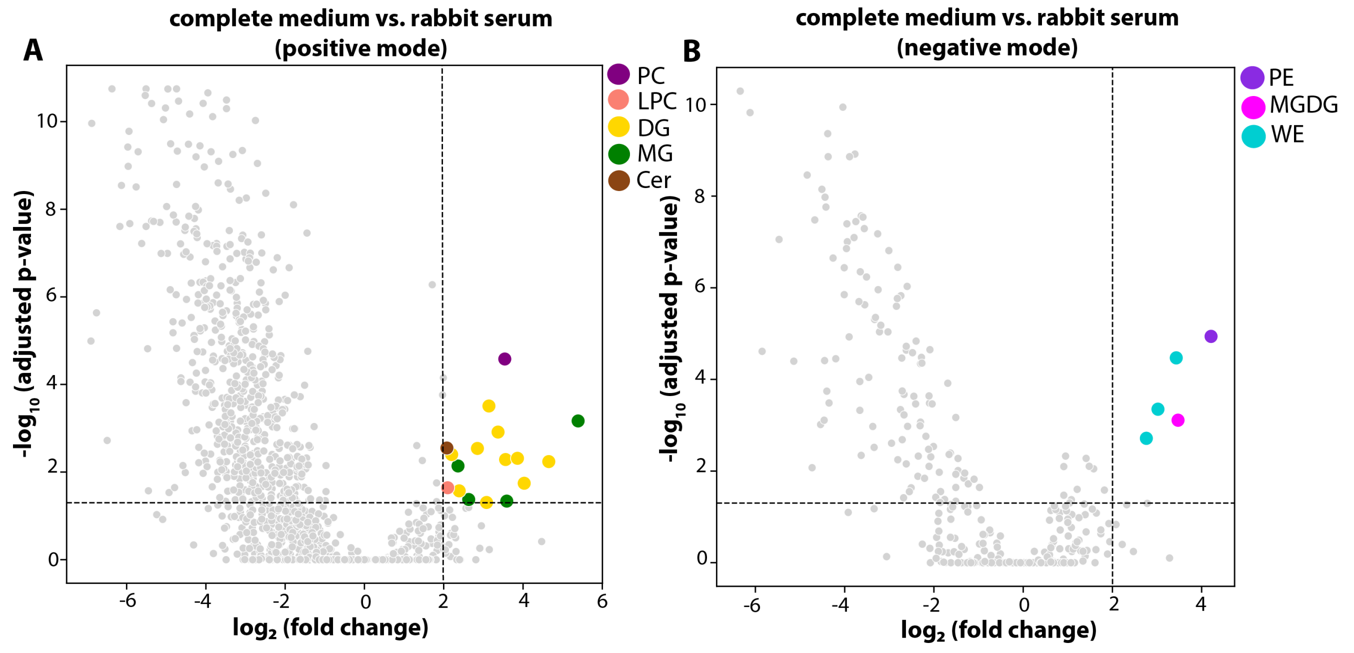

Fig. S6. Untargeted comparative lipidomics of rabbit serum and the complete medium. Volcano plots compare lipid profiles between rabbit serum and complete medium in **(A)** positive and **(B)** negative electrospray ionization modes. Most lipids in complete medium originate from rabbit serum, with limited medium-specific lipid enrichment (highlighted in colors). The plots demonstrate significant overlap in lipid composition between rabbit serum and complete medium.

bioRxiv preprint doi: https://doi.org/10.64898/2026.05.22.727245; this version posted May 23, 2026. The copyright holder for this preprint (which was not certified by peer review) is the author/funder, who has granted bioRxiv a license to display the preprint in perpetuity. It is made available under a CC-BY 4.0 International license.

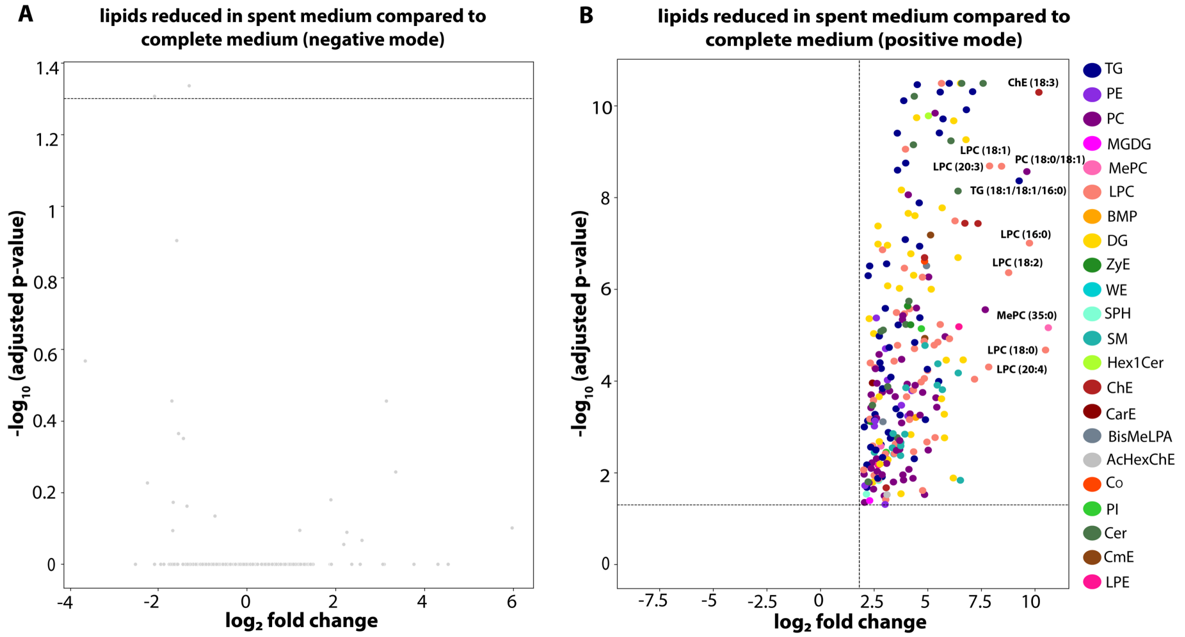

Fig. S7. Lipid depletion in the spent medium. Volcano plots showing lipids enriched in the complete medium that are absent in the spent medium. No substantial differential enrichment was observed for lipids that ionize in negative ionization mode (**A**) but a substantial number of lipids that ionize in positive ionization mode was detected (**B**); the ten most significantly reduced lipids are labeled.

bioRxiv preprint doi: https://doi.org/10.64898/2026.05.22.727245; this version posted May 23, 2026. The copyright holder for this preprint (which was not certified by peer review) is the author/funder, who has granted bioRxiv a license to display the preprint in perpetuity. It is made available under a CC-BY 4.0 International license.

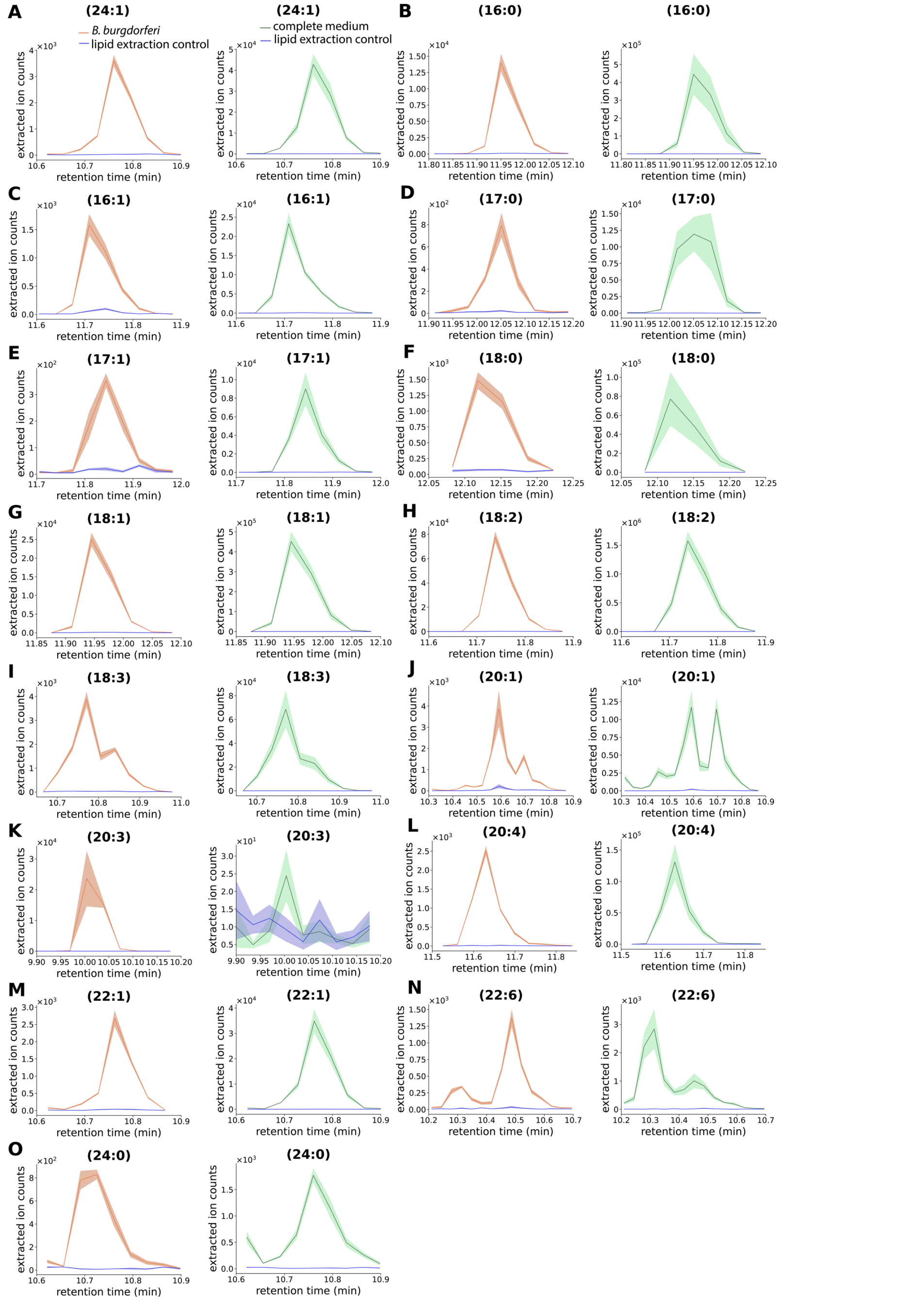

Fig. S8. **Cholesteryl esters in *B. burgdorferi* and the complete medium.** Extracted ion chromatograms (EICs) of diagnostic product ions from targeted MS/MS analysis of selected CE species are shown. Orange traces represent *B. burgdorferi* samples, green traces represent BSKII complete medium, and blue traces represent lipid extraction controls. The solid line indicates the mean of four biological replicates, and the shaded area denotes the standard error of the mean (SEM).

bioRxiv preprint doi: https://doi.org/10.64898/2026.05.22.727245; this version posted May 23, 2026. The copyright holder for this preprint (which was not certified by peer review) is the author/funder, who has granted bioRxiv a license to display the preprint in perpetuity. It is made available under a CC-BY 4.0 International license.

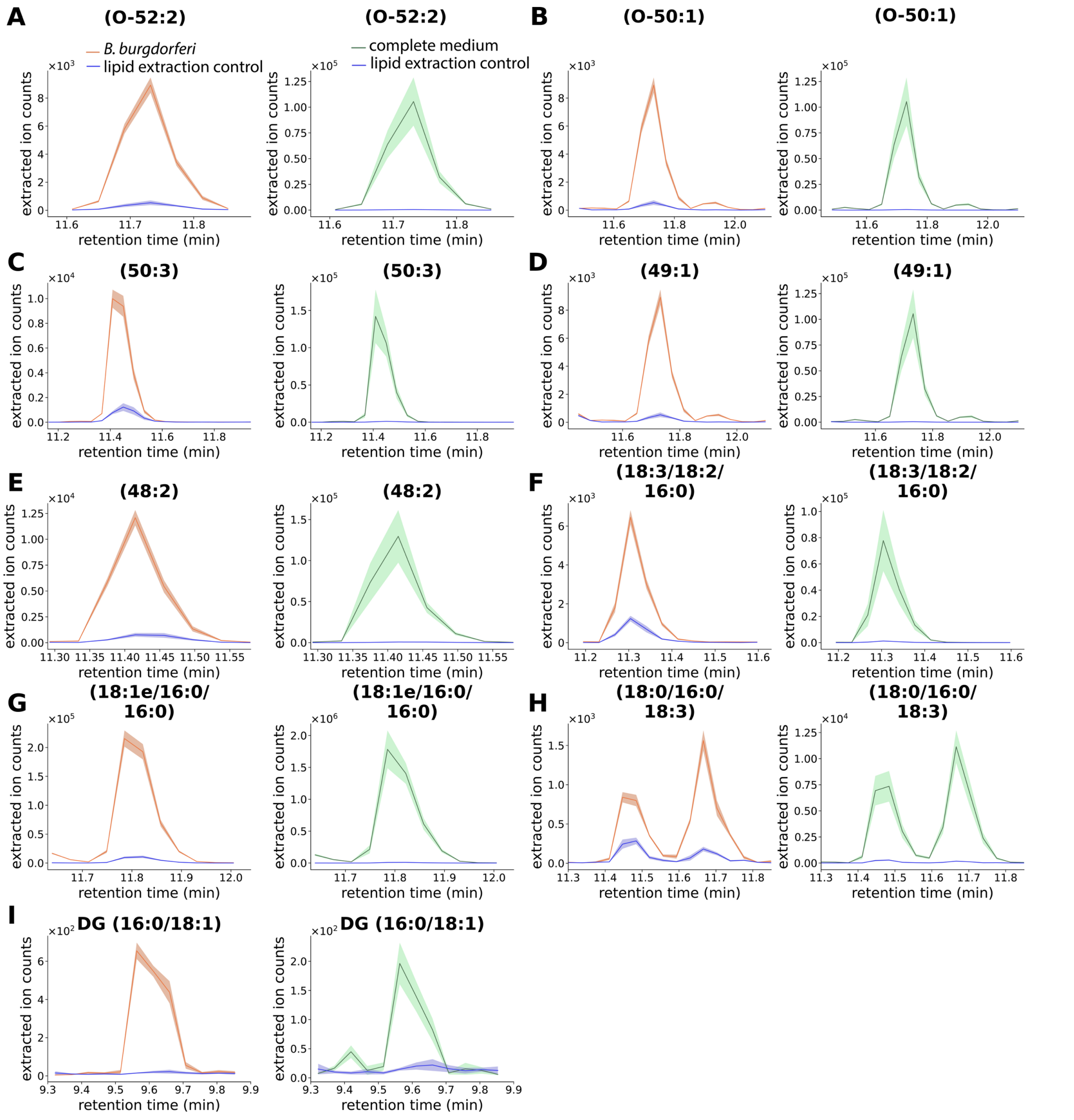

Fig. S9. Neutral lipids in *B. burgdorferi* and the complete medium. EICs of diagnostic product ions from targeted MS/MS analysis of the indicated neutral lipid species are shown. Orange traces represent *B. burgdorferi* samples, green traces represent BSKII complete medium, and blue traces represent lipid extraction controls. The solid line indicates the mean of four biological replicates, and the shaded area denotes the SEM.

bioRxiv preprint doi: https://doi.org/10.64898/2026.05.22.727245; this version posted May 23, 2026. The copyright holder for this preprint (which was not certified by peer review) is the author/funder, who has granted bioRxiv a license to display the preprint in perpetuity. It is made available under a CC-BY 4.0 International license.

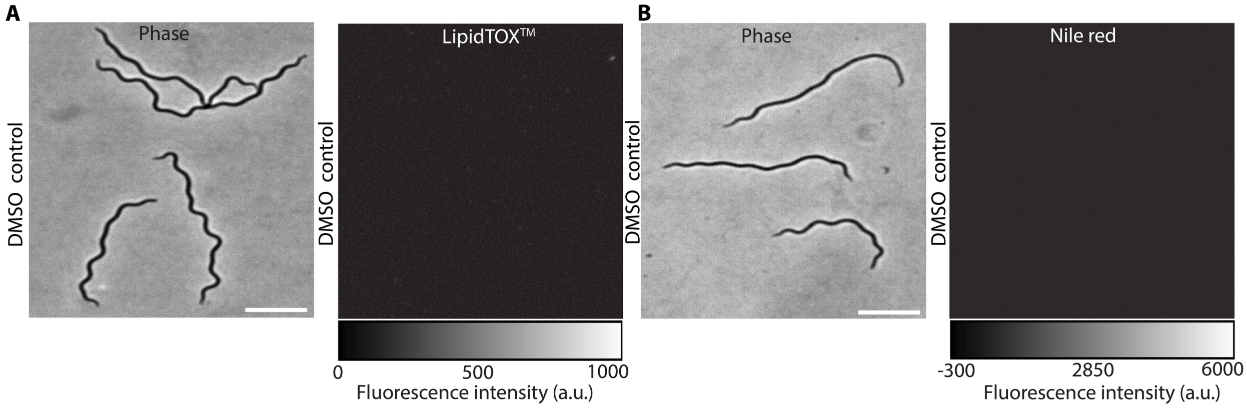

Fig. S10. Microscopy of *B. burgdorferi*. Widefield epifluorescence microscopy images of fixed *B. burgdorferi* cells treated with DMSO control at a 1:200 ratio in phosphate-buffered saline with $MgCl_2$, observed under the phase contrast and the LipidTOX™ fluorescence channel (**A**) and the phase contrast and Nile Red fluorescence channel. Scale bar = 5 μm.

bioRxiv preprint doi: https://doi.org/10.64898/2026.05.22.727245; this version posted May 23, 2026. The copyright holder for this preprint (which was not certified by peer review) is the author/funder, who has granted bioRxiv a license to display the preprint in perpetuity. It is made available under a CC-BY 4.0 International license.

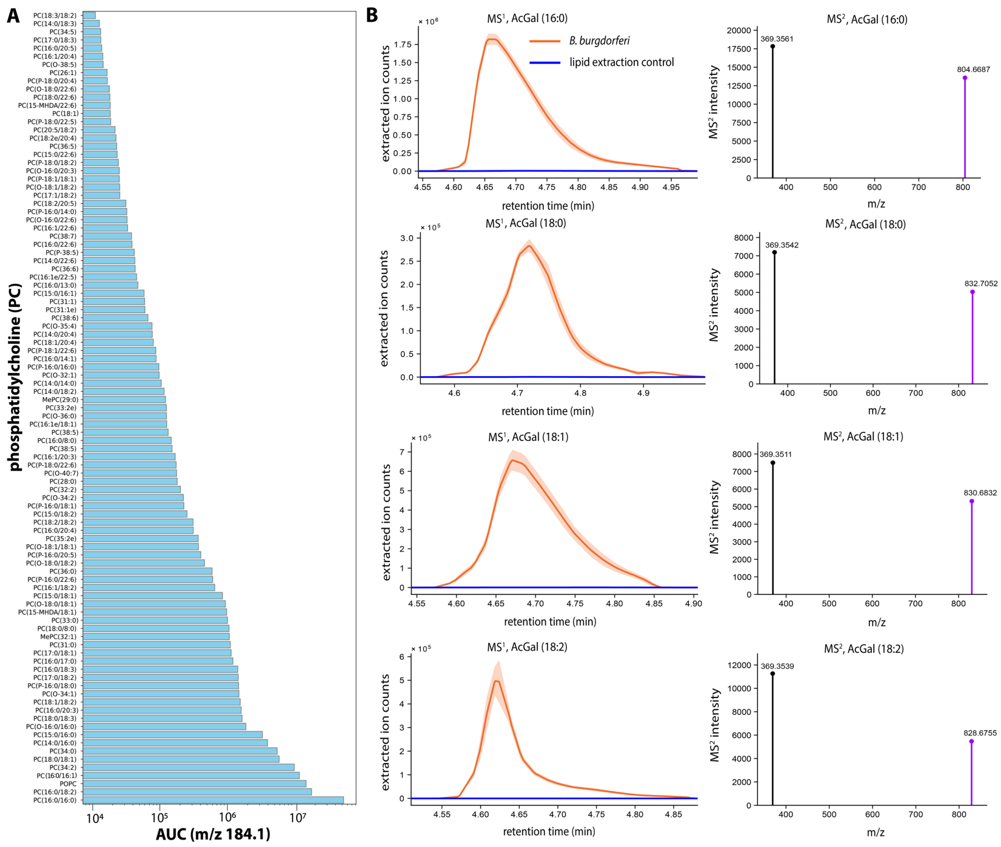

Fig. S11. Targeted profiling of phosphatidylcholines (PCs) and acylated cholesterol galactolipids (AcGals) in *B. burgdorferi*. (**A**) Ranked abundances of the area under the curve (AUC) of 97 PC species identified in *B. burgdorferi*. Abundances were calculated from the extracted ion chromatograms of the diagnostic PC product ion at m/z 184.1. (**B**) Left panel: EICs show the signal for each AcGal precursor ion in *B. burgdorferi* (orange) compared to a lipid extraction control (blue). Right panel: Corresponding mass spectra showing the intact ammonium-adducted precursor ion ($[M+NH_4]^+$, purple) and characteristic fragment ions.

bioRxiv preprint doi: https://doi.org/10.64898/2026.05.22.727245; this version posted May 23, 2026. The copyright holder for this preprint (which was not certified by peer review) is the author/funder, who has granted bioRxiv a license to display the preprint in perpetuity. It is made available under a CC-BY 4.0 International license.

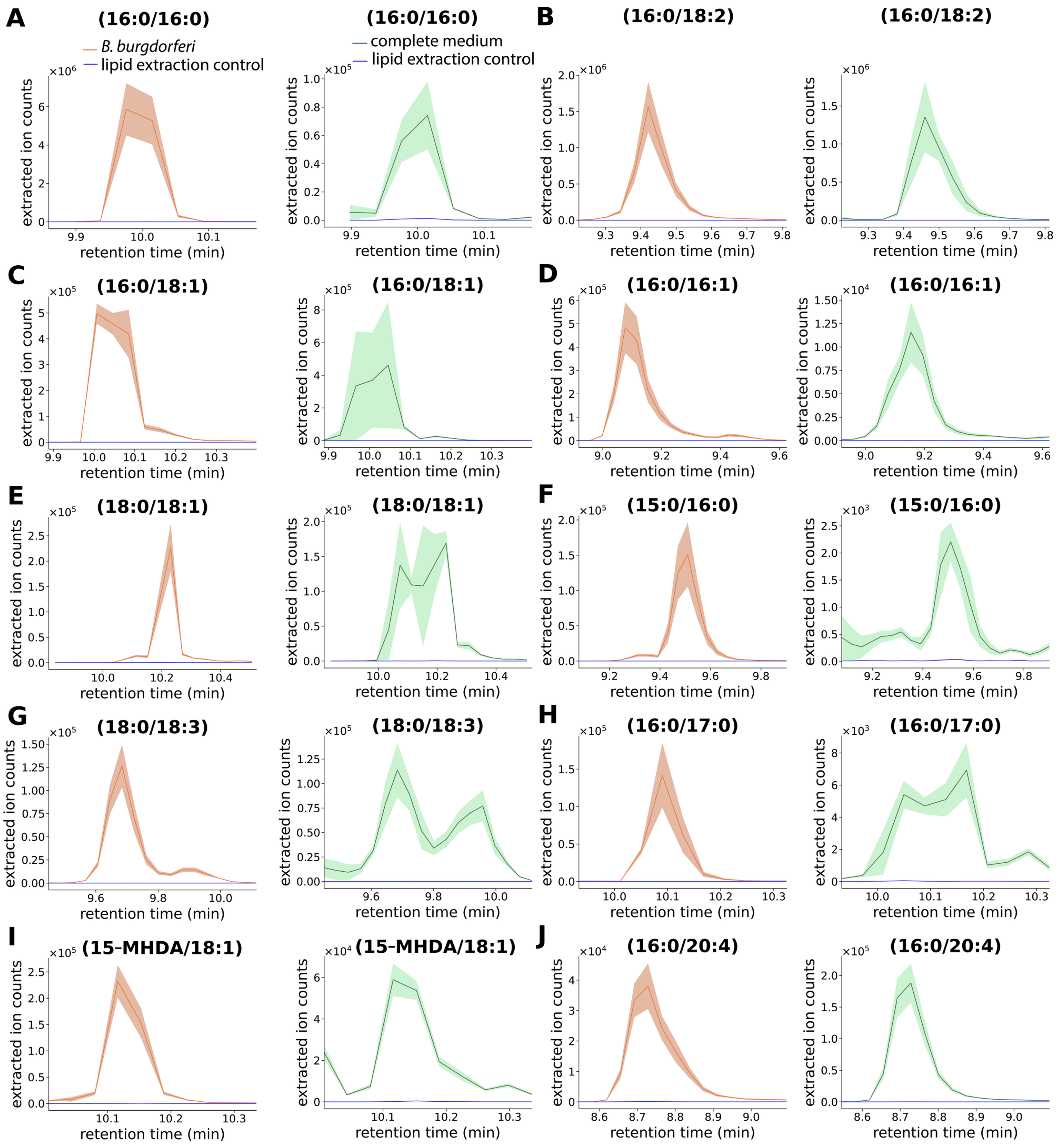

Fig. S12. Phosphatidylcholines in *B. burgdorferi* and the complete medium. EICs of diagnostic product ions from targeted MS/MS analysis of the ten selected PC species in *B. burgdorferi*. Orange traces represent *B. burgdorferi* samples, green traces represent BSKII complete medium, and blue traces represent lipid extraction controls. The solid line indicates the mean of four biological replicates, and the shaded area denotes the SEM.

bioRxiv preprint doi: https://doi.org/10.64898/2026.05.22.727245; this version posted May 23, 2026. The copyright holder for this preprint (which was not certified by peer review) is the author/funder, who has granted bioRxiv a license to display the preprint in perpetuity. It is made available under a CC-BY 4.0 International license.

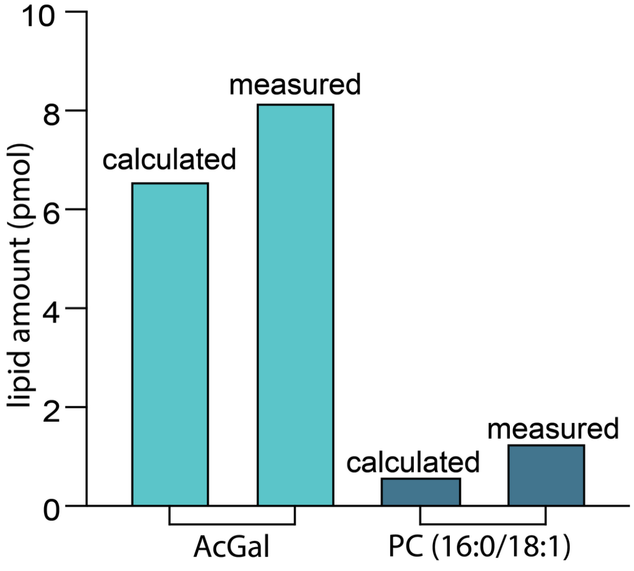

Fig. S13. Validation of the response factor calculation. The amounts of AcGal (16:0) and PC (16:0/18:1) calculated for data acquired via the QQQ (calculated) are similar to the amounts measured using a multipoint calibration curve-based performed on a Q-TOF instrument (measured).

bioRxiv preprint doi: https://doi.org/10.64898/2026.05.22.727245; this version posted May 23, 2026. The copyright holder for this preprint (which was not certified by peer review) is the author/funder, who has granted bioRxiv a license to display the preprint in perpetuity. It is made available under a CC-BY 4.0 International license.

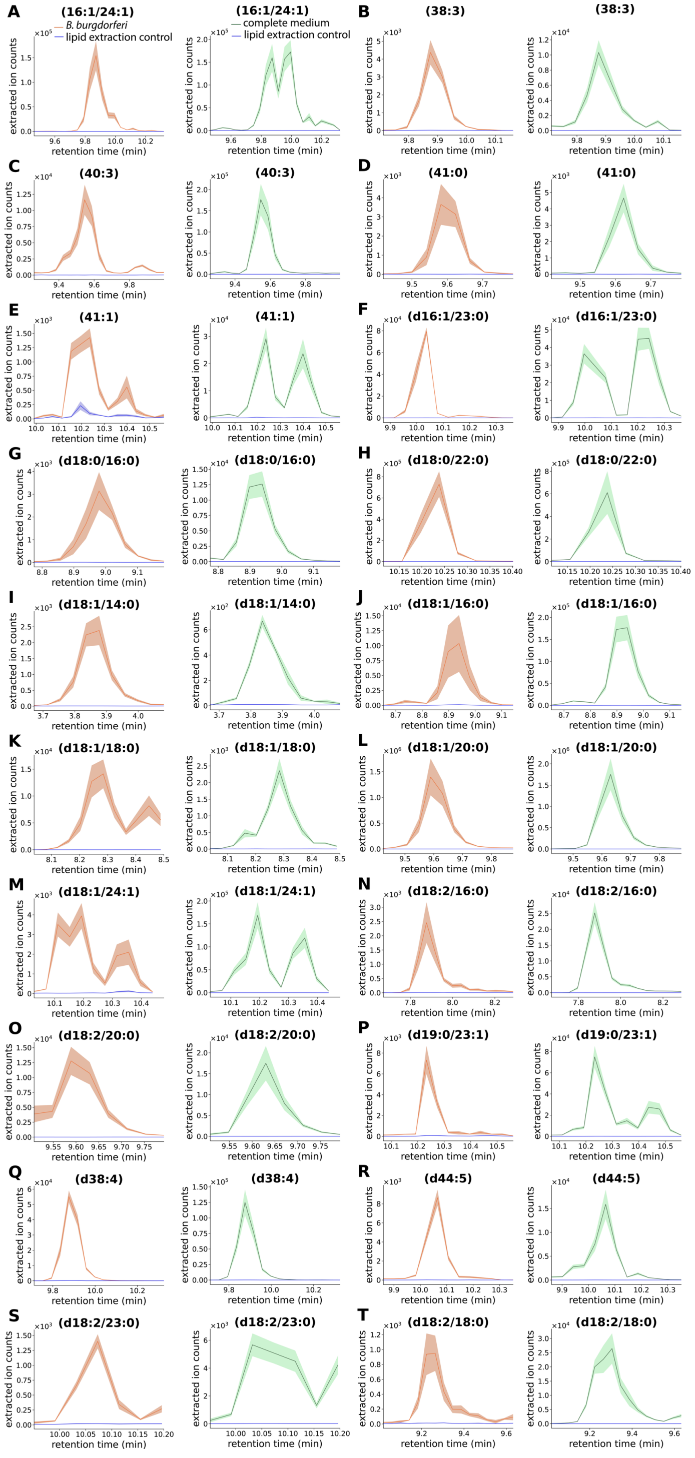

Fig. S14. Sphingomyelins in *B. burgdorferi* and the complete medium. EICs of diagnostic product ions from targeted MS/MS analysis of sphingomyelin species are shown. Orange traces represent *B. burgdorferi* samples, green traces represent BSKII complete medium, and blue traces represent lipid extraction controls. The solid line indicates the mean of four biological replicates, and the shaded area denotes the SEM.

bioRxiv preprint doi: https://doi.org/10.64898/2026.05.22.727245; this version posted May 23, 2026. The copyright holder for this preprint (which was not certified by peer review) is the author/funder, who has granted bioRxiv a license to display the preprint in perpetuity. It is made available under a CC-BY 4.0 International license.

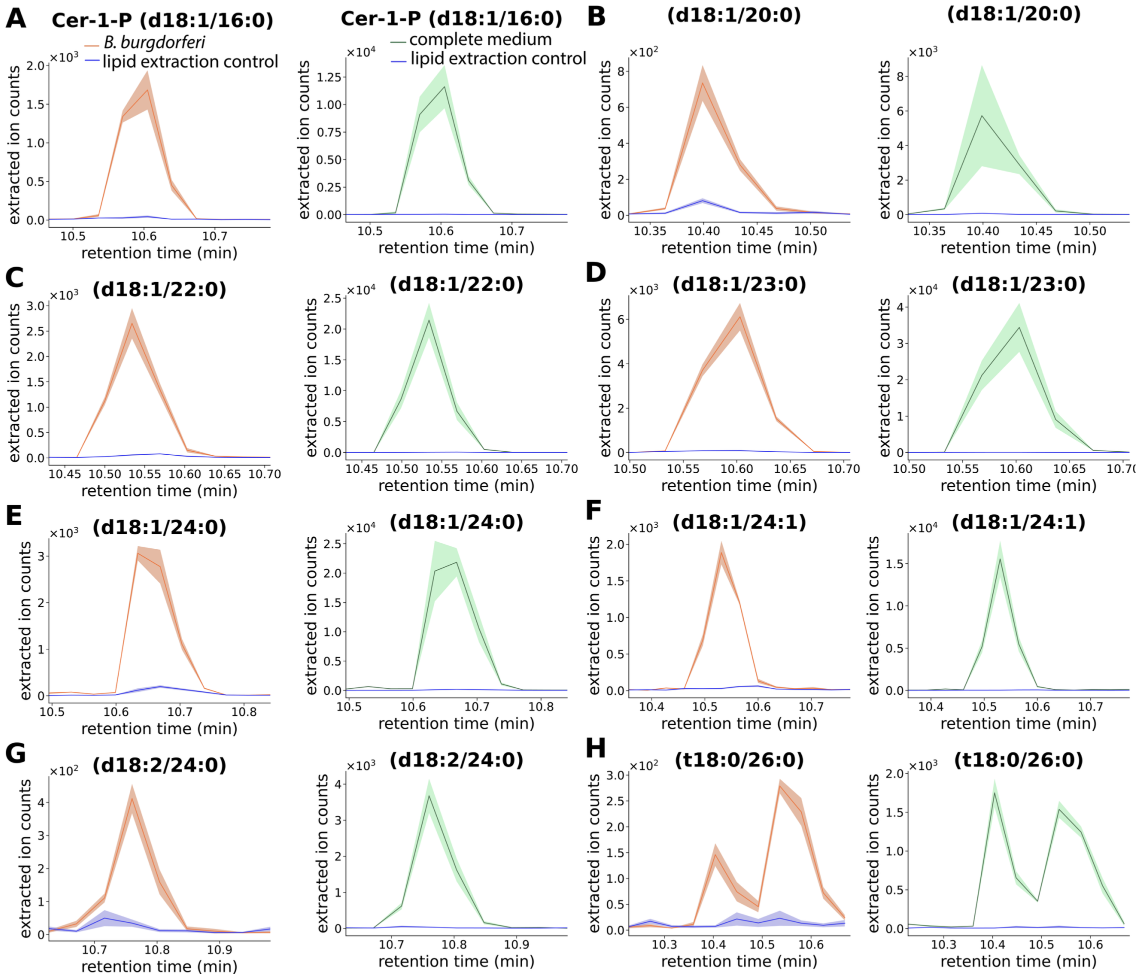

Fig. S15. Analysis of ceramides in *B. burgdorferi* and the complete medium. EICs of diagnostic product ions from targeted MS/MS analysis of ceramide species are shown. Panels correspond to individual ceramide species. Orange traces represent *B. burgdorferi* samples, green traces represent BSKII complete medium, and blue traces represent lipid extraction controls. The solid line indicates the mean of four biological replicates, and the shaded area denotes the SEM.

bioRxiv preprint doi: https://doi.org/10.64898/2026.05.22.727245; this version posted May 23, 2026. The copyright holder for this preprint (which was not certified by peer review) is the author/funder, who has granted bioRxiv a license to display the preprint in perpetuity. It is made available under a CC-BY 4.0 International license.

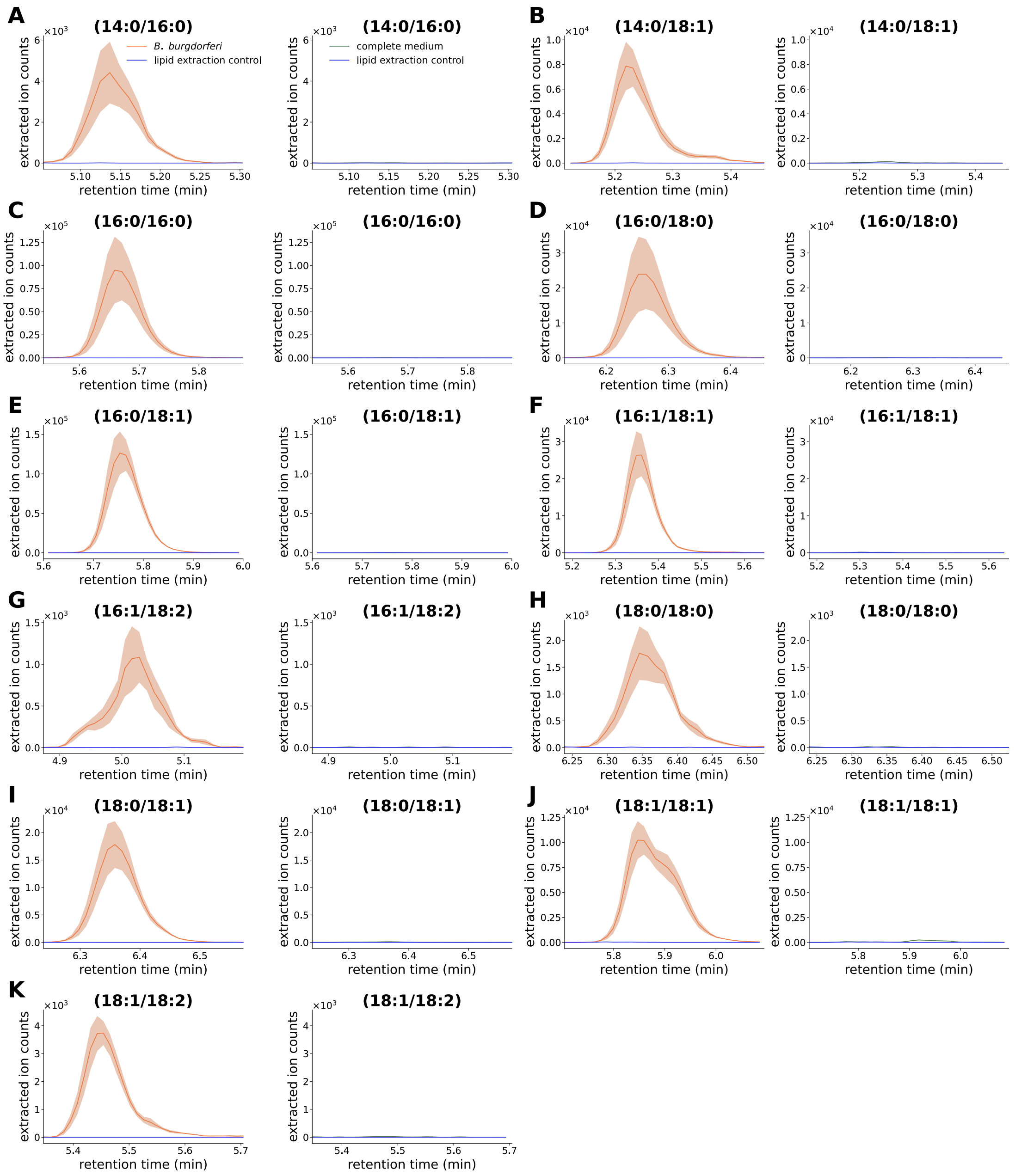

Fig. S16. Analysis of phosphatidylglycerols in *B. burgdorferi* and the complete medium. EICs of diagnostic product ions from targeted MS/MS analysis of phosphatidylglycerol species are shown. Orange traces represent *B. burgdorferi* samples, green traces represent BSKII complete medium, and blue traces represent lipid extraction controls. The solid line indicates the mean of four biological replicates, and the shaded area denotes the SEM.

bioRxiv preprint doi: https://doi.org/10.64898/2026.05.22.727245; this version posted May 23, 2026. The copyright holder for this preprint (which was not certified by peer review) is the author/funder, who has granted bioRxiv a license to display the preprint in perpetuity. It is made available under a CC-BY 4.0 International license.

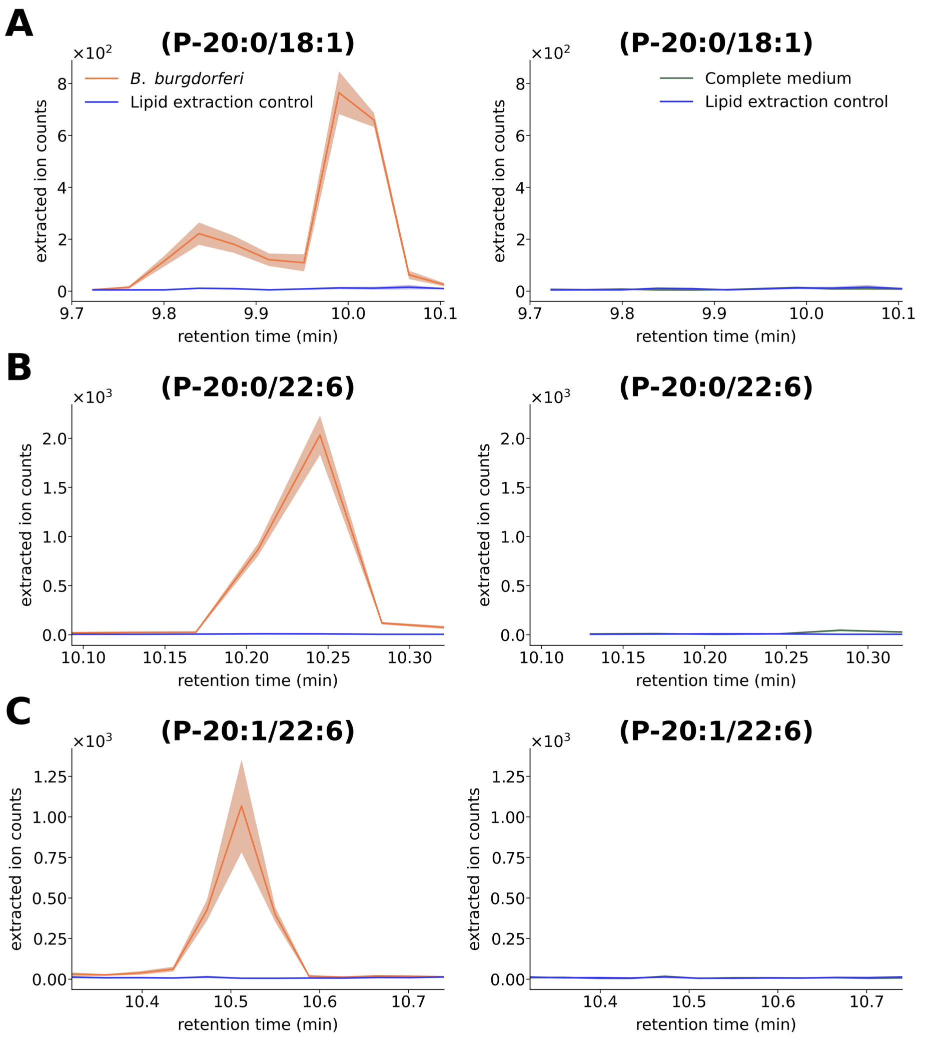

Fig. S17. Analysis of phosphatidylethanolamines in *B. burgdorferi* and the complete medium. EICs of diagnostic product ions from targeted MS/MS analysis of phosphatidylethanolamine species are shown. Orange traces represent *B. burgdorferi* samples, green traces represent BSKII complete medium, and blue traces represent lipid extraction controls. The solid line indicates the mean of four biological replicates, and the shaded area denotes the SEM.

bioRxiv preprint doi: https://doi.org/10.64898/2026.05.22.727245; this version posted May 23, 2026. The copyright holder for this preprint (which was not certified by peer review) is the author/funder, who has granted bioRxiv a license to display the preprint in perpetuity. It is made available under a CC-BY 4.0 International license.

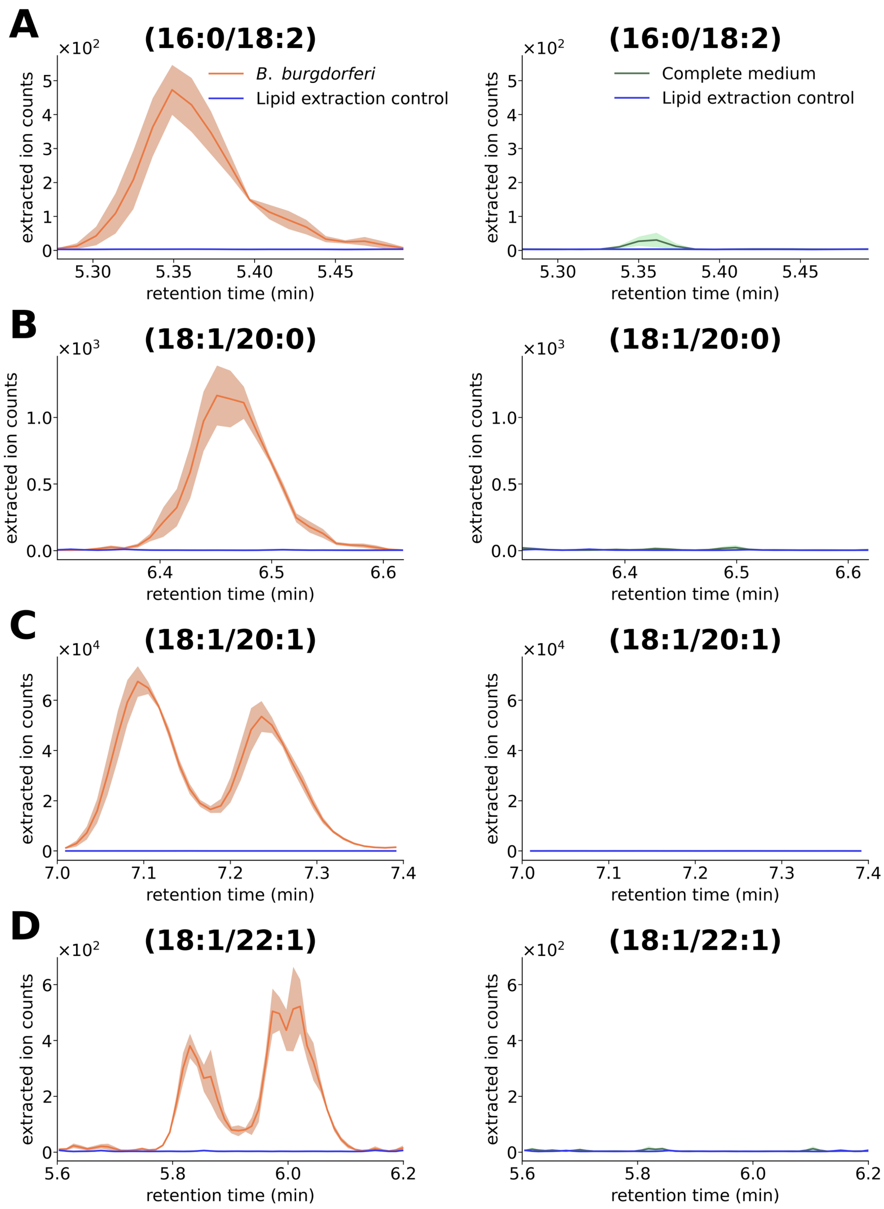

Fig. S18. Analysis of bis(monoacylglycero)phosphates (BMPs) in *B. burgdorferi* and the complete medium. EICs of diagnostic product ions from targeted MS/MS analysis of BMPs species are shown. Orange traces represent *B. burgdorferi* samples, green traces represent BSKII complete medium, and blue traces represent lipid extraction controls. The solid line indicates the mean of four biological replicates, and the shaded area denotes the SEM.

bioRxiv preprint doi: https://doi.org/10.64898/2026.05.22.727245; this version posted May 23, 2026. The copyright holder for this preprint (which was not certified by peer review) is the author/funder, who has granted bioRxiv a license to display the preprint in perpetuity. It is made available under a CC-BY 4.0 International license.

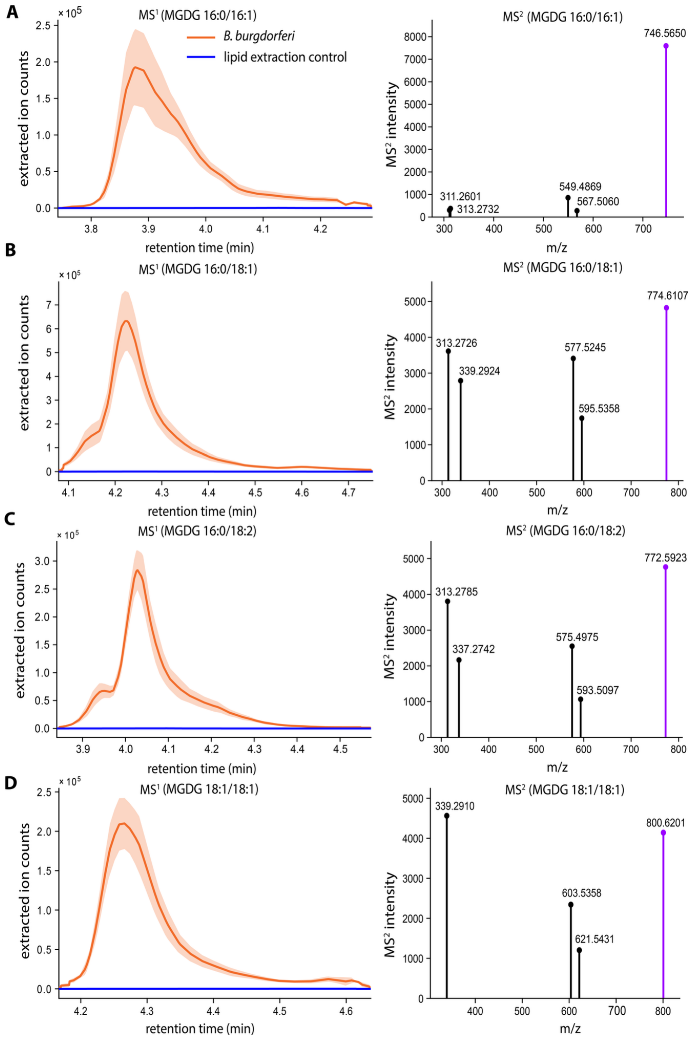

Fig. S19. Representative mass spectrometry data for four distinct monogalactosyldiacylglycerol (MGDG) species in *B. burgdorferi*. MGDGs were characterized by their different fatty acid compositions (A: 16:0/16:1, B: 16:0/18:1, C: 16:0/18:2, and D: 18:1/18:1). Left panel: EICs show the signal for each MGDG precursor ion in *B. burgdorferi* (orange) compared to a lipid extraction control (blue). Right panel: Corresponding mass spectra showing the intact ammonium-adducted precursor ion ($[M+NH_4]^+$, purple) and characteristic fragment ions.

bioRxiv preprint doi: https://doi.org/10.64898/2026.05.22.727245; this version posted May 23, 2026. The copyright holder for this preprint (which was not certified by peer review) is the author/funder, who has granted bioRxiv a license to display the preprint in perpetuity. It is made available under a CC-BY 4.0 International license.

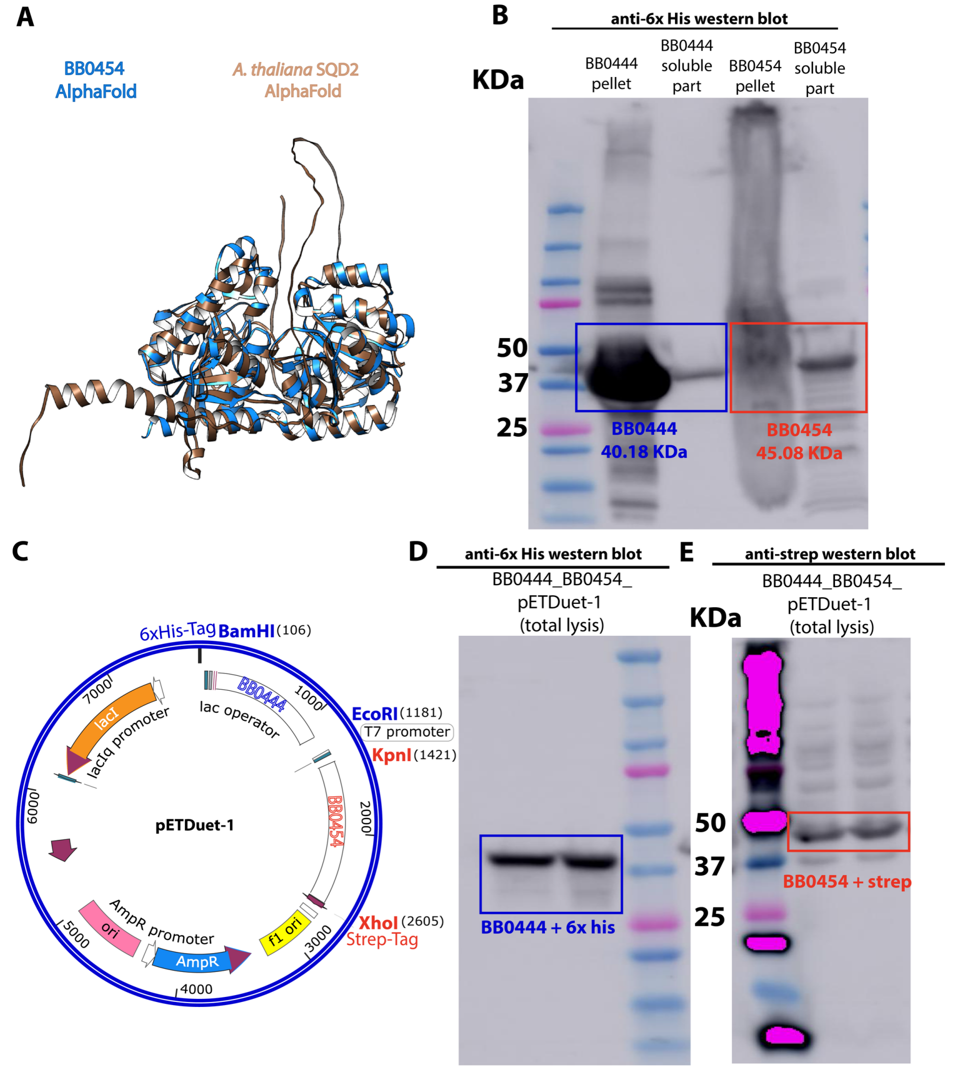

Fig. S20. SQDG synthesis in *B. burgdorferi*. (**A**) An alignment of AlphaFold2 models of BB0454 and the SQD2 protein from *A. thaliana*. (**B**) Heterologously expressed BB0444 and BB0454 cloned separately in pET28a vectors and bearing hexa-histidine affinity tags. (**C**) Plasmid map of pETDuet vector containing BB0444 and BB0454. (**D, E**) Heterologously expressed of BB0444 and BB0454 in verified through immunoblotting using affinity tags appended to each protein in pETDuet vector.

bioRxiv preprint doi: https://doi.org/10.64898/2026.05.22.727245; this version posted May 23, 2026. The copyright holder for this preprint (which was not certified by peer review) is the author/funder, who has granted bioRxiv a license to display the preprint in perpetuity. It is made available under a CC-BY 4.0 International license.

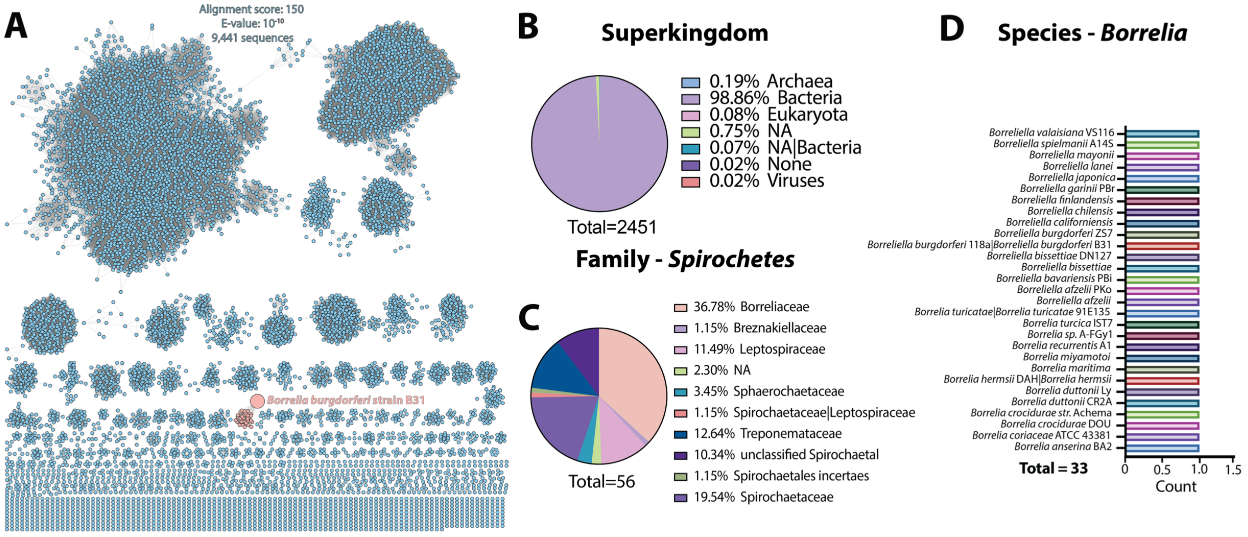

Fig. S21. Bioinformatics analyses of BB0444. (**A**) Protein sequence similarity network (SSN) the BB0444 protein from *B. burgdorferi*, showing separate clustering from homologs with a minimum of 90% identity. (**B**) Distribution of BB0444 homologs suggest they are widespread in bacteria, including in spirochetes (**C**) where a total of 33 Borrelia species encode a BB0444 homolog (**D**).

bioRxiv preprint doi: https://doi.org/10.64898/2026.05.22.727245; this version posted May 23, 2026. The copyright holder for this preprint (which was not certified by peer review) is the author/funder, who has granted bioRxiv a license to display the preprint in perpetuity. It is made available under a CC-BY 4.0 International license.

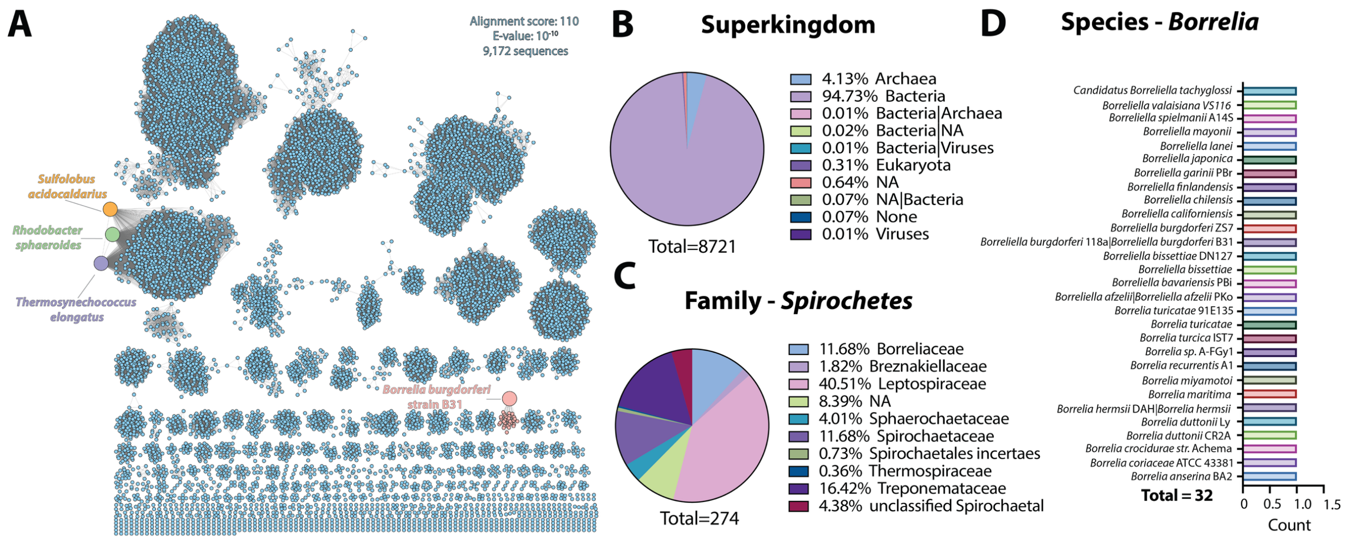

Fig. S22. Bioinformatics analyses of BB0454. (**A**) Protein sequence similarity network (SSN) the BB0454 protein from *B. burgdorferi*, showing separate clustering from homologs with a minimum of 90% identity. (**B**) Distribution of BB0454 homologs suggest they are widespread in bacteria, including in spirochetes (**C**). A total of 32 Borrelia species encode a BB0454 homolog (**D**).

bioRxiv preprint doi: https://doi.org/10.64898/2026.05.22.727245; this version posted May 23, 2026. The copyright holder for this preprint (which was not certified by peer review) is the author/funder, who has granted bioRxiv a license to display the preprint in perpetuity. It is made available under a CC-BY 4.0 International license.

Supporting table

Table S1: Amino acid sequences of *Borrelia burgdorferi* (strain ATCC 35210 / DSM 4680 / CIP 102532 / B31) protein sequences

Protein ID	Uniprot Accession	Protein sequence
BB0444	O51400	MKIFLTGIAGFIGFHVAKKLVEKGHEVLGIDVLNDYYEL<br>KFKHERLEALGFCSKDVKTHKIIKSEKYNNLSFAYLDIL<br>NKDKLLELFKDHKFTHVCHLAAQAGIRDSLENPDSYVS<br>INIVGFFNVLDVCRVYKENIEHFVYASTSSVYGINENMP<br>SSEDSITDHPLNLYAASKKSNEMMAHAYSASFNIPTTG<br>LRFFTVYGTYGRPDMALYLFSDGIKNGKAINIFNNGNM<br>ARDFTYVGDIADGVYKVLKNPAKSDCNFDVKNPNSST<br>SSFPYRIYNIGTGHATKLLDFISELEANFDKKALKNYMP<br>MQKADVVESCCDILKLKNDVGYEAKVSIKEGIKEFSQW<br>YKMLESTKKT
BB0454	O51410	MKVAIFTDTYIPEKNGVATSIKQIKEGFEKNGYEVYIFCP<br>KSKKSLNEKNVYRCSSIQINKKLDAVIAFPNKRKISKIIQ<br>SYKPDIIHTHSEFSMGKIGKQIALKHNIPIVHTSHTMWD<br>YYLHYLGIFKYFIKPDKMMRKHYNKIKHFIYPSSKAKER<br>YFQLSNNSSNYKIIPNGVDRKLFIKTLSKEKKDEILKKHN<br>IKQTDKIIIFVGRINKEKNINLLVTHLKDLLMQNNNYKLILI<br>GKGSEEKEIKNFSIKHGLEKQILLIGTIPWEEIYYYYKISD<br>IFASLSKSEVYPMTVIEALTAGIPAILINDYIYKDVIKEGIN<br>GFLIKKYENLSRYIDKVIKDDEILKKFKENAKKHSTKFSS<br>YFFTKKIKNYYSEIIARKNH
