# Supplementary material for: A lipid compendium of a metabolically compromised bacterium provides insights into lipid acquisition, biosynthesis, and metabolism
Source: bioRxiv. 2026 May 23:2026.05.22.727245. Preprint. [Version 1] doi: 10.64898/2026.05.22.727245 (PMC13228262; doi:10.64898/2026.05.22.727245)
Supplement: Supplement 2 [file media-2.pdf]

Supplementary spreadsheet 2: Untargeted lipidomics analysis of ESI-positive mode data obtaine						
Name	Formula	Annot. DeltaN	Calc. MW	m/z	RT [min]	MS Depth
ZyE(20:5)	C47 H72 O2	1.53	668.55425	669.5615	17.299	2
ZyE(18:3)	C45 H72 O2	1.36	644.55411	645.56139	18.665	2
ZyE(0:0)	C27 H44 O	1.12	384.33965	385.34692	12.666	2
ZyE()	C27 H44 O	2.88	384.34032	367.33704	24.547	2
WE(5:0_19:2)	C24 H44 O2	1.89	364.33482	365.3421	14.246	2
WE(5:0_16:1)	C21 H40 O2	0.5	324.30299	325.31027	9.722	2
WE(5:0_15:2)	C20 H36 O2	0.87	308.2718	309.27908	7.342	2
WE(4:0_22:2)	C26 H48 O2	2.41	392.36638	393.37366	16.099	2
WE(4:0_19:1)	C23 H44 O2	1.37	352.33461	353.34189	14.535	1
WE(4:0_16:3)	C20 H34 O2	1.01	306.25619	307.26346	8.581	1
WE(4:0_14:2)	C18 H32 O2	0.59	280.24039	281.24768	4.925	1
WE(3:0_20:2)	C23 H42 O2	2.14	350.31923	383.35275	8.378	2
WE(3:0_17:2)	C20 H36 O2	0.2	308.27159	309.27887	6.847	2
WE(3:0_16:3)	C19 H32 O2	0.87	292.24048	293.24775	5.284	2
WE(2:0_14:2)	C16 H28 O2	0.2	252.20898	253.21626	4.086	1
TG(9:0_9:0_9:0)	C30 H56 O6	-5.98	512.40462	513.41382	11.52	2
TG(8:0_8:0_10:3)	C29 H48 O6	-4.42	492.34291	493.35019	10.358	1
TG(8:0_10:2_18:2)	C39 H66 O6	-0.48	630.48564	631.49253	11.854	2
TG(8:0_10:2_18:1)	C39 H68 O6	1.13	632.5023	633.50956	12.965	2
TG(8:0_10:1_18:1)	C39 H70 O6	0.94	634.51784	652.55249	12.526	1
TG(6:0_6:0_13:0)	C28 H52 O6	-2.54	484.37516	485.38251	8.246	1
TG(6:0_14:4_18:2)	C41 H66 O6	-2.79	654.48411	655.49139	11.083	2
TG(6:0_14:4_18:1)	C41 H68 O6	-2.15	656.50018	657.50745	12.639	2
TG(6:0_12:2_18:2)	C39 H66 O6	0.82	630.48646	631.49352	11.541	2
TG(6:0_12:2_18:1)	C39 H68 O6	0.38	632.50183	655.49119	11.293	2
TG(6:0_11:2_18:3)	C38 H62 O6	-0.9	614.45409	615.46051	7.519	1
TG(6:0_11:2_18:2)	C38 H64 O6	-2.18	616.46894	617.47632	8.353	2
TG(6:0_11:1_18:2)	C38 H66 O6	-2.55	618.48436	619.49164	9.13	1
TG(6:0_11:1_14:2)	C34 H58 O6	-2.02	562.4222	563.42914	8.217	1
TG(6:0_10:3_10:4)	C29 H40 O6	0.08	484.28253	485.28995	19.407	2
TG(4:0_6:0_17:1)	C30 H54 O6	1.11	510.3926	511.40003	8.697	2
TG(4:0_6:0_11:1)	C24 H42 O6	0.78	426.29847	427.30575	18.413	2
TG(4:0_16:0_18:2)	C41 H74 O6	8.29	662.55403	663.56131	19.407	2

TG(4:0_16:0_18:1)	C41 H76 O6	9.03	664.57019	665.57747	19.646	2
TG(36:4)	C39 H66 O6	-2.4	630.48442	631.4917	12.386	2
TG(22:1_10:3_10:3)	C45 H72 O6	1.14	708.5337	709.54104	13.824	2
TG(20:3e_18:1_18:3)	C59 H102 O5	5.17	890.77733	891.78461	19.27	1
TG(20:3e_11:3_20:3)	C54 H88 O5	-3.71	816.66014	817.66742	18.478	3
TG(20:3e_11:2_18:3)	C52 H86 O5	-3.44	790.64481	791.6517	18.366	3
TG(20:2e_18:3_20:4)	C61 H102 O5	4.23	914.7766	915.78387	18.773	2
TG(20:2e_11:2_18:3)	C52 H88 O5	-3.07	792.66074	793.6675	18.66	3
TG(20:2e_10:2_12:3)	C45 H74 O5	0.85	694.55422	695.56039	17.33	2
TG(20:1e_18:3_20:4)	C61 H104 O5	2.21	916.7904	917.79768	18.729	1
TG(20:1e_11:3_11:3)	C45 H74 O5	0.19	694.55376	695.56104	14.644	1
TG(20:1_18:1_18:1)	C59 H108 O6	-1.27	912.81343	913.82071	21.967	1
TG(20:0e_18:2_16:0)	C57 H108 O5	0.81	872.82038	890.8542	20.931	3
TG(20:0e_18:1_16:0)	C57 H110 O5	0.63	874.83588	892.8697	21.183	3
TG(20:0e_18:0_18:2)	C59 H112 O5	0.53	900.85145	918.88526	21.226	3
TG(20:0e_18:0_18:1)	C59 H114 O5	0.74	902.86729	920.90112	21.508	3
TG(20:0e_17:0_18:1)	C58 H112 O5	0.33	888.85127	906.88503	21.344	3
TG(20:0e_16:0_18:0)	C57 H112 O5	0.86	876.85173	894.88557	21.52	3
TG(20:0e_16:0_17:0)	C56 H110 O5	1.13	862.8363	880.87012	21.342	3
TG(20:0e_16:0_16:0)	C55 H108 O5	0.69	848.82026	866.85409	21.193	3
TG(20:0e_15:0_16:0)	C54 H106 O5	3.55	834.80699	852.83847	21.045	3
TG(20:0_16:0_16:0)	C55 H106 O6	1.01	862.79981	880.83337	19.566	2
TG(19:1_18:1_18:4)	C58 H100 O6	3.09	892.75475	893.76202	19.18	1
TG(19:1_12:4_12:4)	C46 H70 O6	-3.54	718.5147	719.52197	14.627	1
TG(19:0)	C22 H40 O6	0.99	400.28288	401.29016	18.622	1
TG(18:4_6:0_18:4)	C45 H70 O6	1.15	706.51805	707.52533	12.228	1
TG(18:4_18:2_18:2)	C57 H94 O6	0.02	874.70506	875.71234	16.305	2
TG(18:4_17:1_18:1)	C56 H96 O6	4.38	864.72447	865.73175	18.747	1
TG(18:4_16:1_17:1)	C54 H92 O6	4.07	836.6928	837.70007	19.126	1
TG(18:4_16:0_18:2)	C55 H94 O6	0.86	850.70577	851.71305	16.385	1
TG(18:4_16:0_18:1)	C55 H96 O6	0.75	852.72133	853.7286	18.696	2
TG(18:3e_18:4_22:3)	C61 H100 O5	6.48	912.76299	913.77026	18.602	1
TG(18:3_18:3_16:0)	C55 H94 O6	-2.2	850.70317	851.71045	20.706	1
TG(18:3_18:2_18:2)	C57 H96 O6	-2.62	876.71839	877.72567	19.873	1
TG(18:3_18:2_16:0)	C55 H96 O6	0.58	852.72118	870.75502	20.584	3

TG(18:2_18:2_20:4)	C59 H98 O6	5.26	902.74109	903.74837	19.515	1
TG(18:2_18:2_20:1)	C59 H104 O6	-1.19	908.78221	909.78949	20.491	1
TG(18:2_18:2_18:2)	C57 H98 O6	0.7	878.73695	879.74363	19.728	2
TG(18:2_18:2_18:1)	C57 H100 O6	-1.94	880.75028	881.75728	21.165	1
TG(18:2_18:2_17:0)	C56 H100 O6	2.61	868.75426	869.76153	19.865	1
TG(18:2_18:2_16:0)	C55 H98 O6	0.6	854.73685	872.77068	20.764	3
TG(18:2_18:2_15:0)	C54 H96 O6	-4.62	840.71681	919.73597	19.632	1
TG(18:2_10:2_11:4)	C42 H64 O6	2.27	664.47179	665.47907	9.294	1
TG(18:2_10:2_10:2)	C41 H66 O6	-2.57	654.48426	655.49153	11.55	2
TG(18:2_10:1_10:4)	C41 H64 O6	-1.66	652.46921	653.47611	10.592	2
TG(18:1e_16:0_16:0)	C53 H102 O5	0.86	818.77343	836.80725	20.628	3
TG(18:1_18:2_20:3)	C59 H102 O6	5.44	906.77257	907.77985	18.41	1
TG(18:1_18:2_18:3)	C57 H98 O6	1.78	878.7379	879.74522	18.807	2
TG(18:1_18:2_18:2)	C57 H100 O6	-0.08	880.75192	881.75922	19.929	2
TG(18:1_18:2_18:1)	C57 H102 O6	-2.51	882.76543	883.77271	21.412	1
TG(18:1_18:2_16:0)	C55 H100 O6	0.7	856.75259	857.75996	20.045	3
TG(18:1_18:1_22:6)	C61 H102 O6	4.57	930.7719	931.77917	18.646	1
TG(18:1_18:1_22:4)	C61 H106 O6	3.71	934.80241	917.79919	18.728	1
TG(18:1_18:1_20:1)	C59 H108 O6	-2.23	912.81256	913.81984	20.949	1
TG(18:1_18:1_18:3)	C57 H100 O6	0.72	880.75263	881.75988	18.989	2
TG(18:1_18:1_18:2)	C57 H102 O6	0.41	882.768	883.77501	20.399	2
TG(18:1_18:1_18:1)	C57 H104 O6	1.06	884.78422	885.78947	20.256	3
TG(18:1_18:1_16:0)	C55 H102 O6	-0.23	858.76744	876.80127	20.221	3
TG(18:0e_18:2_16:0)	C55 H104 O5	0.43	844.78874	862.82265	20.636	3
TG(18:0e_18:1_16:0)	C55 H106 O5	1.07	846.80493	864.83874	20.911	3
TG(18:0e_16:0_16:0)	C53 H104 O5	0.49	820.78878	838.82256	20.891	3
TG(18:0_18:3_18:3)	C57 H98 O6	0.45	878.73673	879.74401	17.481	1
TG(18:0_18:1_22:6)	C61 H104 O6	4.67	932.78765	933.79492	19.133	2
TG(18:0_18:1_18:3)	C57 H102 O6	0.65	882.76822	883.77548	19.238	2
TG(18:0_16:0_18:3)	C55 H100 O6	0.31	856.75225	857.75954	19.2	2
TG(18:0_16:0_16:0)	C53 H102 O6	0.46	834.76803	835.7756	19.362	3
TG(17:0_18:1D7_17:1)	C55 H95 D7 O	0.96	865.81241	866.81763	20.193	3
TG(16:2e_6:0_12:2)	C37 H64 O5	0.96	588.47594	589.48322	12.206	2
TG(16:1_18:2_18:3)	C55 H94 O6	0.53	850.70549	851.71249	19.355	2
TG(16:1_18:2_16:0)	C53 H96 O6	-0.17	828.72055	829.72832	19.852	2

TG(16:1_18:2_15:0)	C52 H94 O6	-6.03	814.70013	815.71345	19.597	2
TG(16:1_18:1_20:5)	C57 H96 O6	1.13	876.72168	877.72891	16.846	2
TG(16:1_17:1_18:2)	C54 H96 O6	2.32	840.72264	841.72992	20.966	1
TG(16:1_14:1_15:0)	C48 H88 O6	2.78	760.6602	778.69409	20.264	1
TG(16:1_12:0_14:0)	C45 H84 O6	1.39	720.62779	721.63507	20.342	1
TG(16:0_8:0_10:2)	C37 H66 O6	0.98	606.48653	607.49372	12.937	2
TG(16:0_8:0_10:0)	C37 H70 O6	0.25	610.51739	628.55121	20.855	2
TG(16:0_6:0_17:0)	C42 H80 O6	-2.76	680.59361	681.60089	20.12	2
TG(16:0_6:0_12:2)	C37 H66 O6	1.85	606.48706	607.49426	12.186	2
TG(16:0_18:2_22:4)	C59 H102 O6	4.22	906.77147	907.77875	20.474	1
TG(16:0_18:2_17:1)	C54 H98 O6	-4.57	842.73249	921.75214	19.879	1
TG(16:0_18:2_16:0)	C53 H98 O6	-0.25	830.73613	831.74341	21.142	1
TG(16:0_18:1_22:6)	C59 H100 O6	3.38	904.75504	905.76093	19.13	1
TG(16:0_18:1_18:3)	C55 H98 O6	1.84	854.73791	855.74493	19.38	1
TG(16:0_16:0_18:3)	C53 H96 O6	1.03	828.72154	829.72885	18.914	2
TG(16:0_16:0_18:2)	C53 H98 O6	-3.17	830.7337	831.73721	19.777	1
TG(16:0_16:0_16:0)	C51 H98 O6	-2.23	806.73454	807.74182	19.593	1
TG(16:0_14:0_22:5)	C55 H96 O6	-2.54	852.71852	853.7258	20.92	1
TG(16:0_10:3_11:4)	C40 H62 O6	-2.67	638.45293	671.48633	11.053	2
TG(16:0_10:2_11:4)	C40 H64 O6	0.69	640.47073	658.50485	10.191	2
TG(15:0_6:0_12:1)	C36 H66 O6	1.21	594.48666	612.52032	9.251	2
TG(15:0_18:2_18:3)	C54 H94 O6	-8.21	838.69815	839.70543	19.592	1
TG(15:0_16:1_14:0)	C48 H90 O6	6.79	762.67892	763.6862	20.002	2
TG(15:0_14:0_14:0)	C46 H88 O6	-1.77	736.65679	737.66406	20.075	1
TG(14:0_18:2_18:3)	C53 H92 O6	1.01	824.69022	825.69751	18.37	2
TG(14:0_14:1_13:0)	C44 H82 O6	-1.72	706.60992	707.6172	19.549	1
TG(14:0_14:0_14:3)	C45 H80 O6	-2.87	716.59343	717.60071	23.841	2
TG(14:0_14:0_14:0)	C45 H86 O6	-2.28	722.64079	723.64807	25.703	1
TG(12:1e_6:0_18:4)	C39 H66 O5	1.33	614.49184	615.4993	11.75	2
TG(12:1e_6:0_18:3)	C39 H68 O5	1.4	616.50754	634.54137	16.253	2
TG(12:1e_12:2_12:3)	C39 H64 O5	1.5	612.47629	613.48357	11.808	1
TG(12:0e_6:0_20:5)	C41 H70 O5	-2.37	642.5208	643.52808	17.682	2
TG(12:0e_6:0_10:3)	C31 H54 O5	-3.6	506.3953	507.40251	13.294	2
TG(12:0_14:0_16:0)	C45 H86 O6	-2.36	722.64073	723.64801	19.209	1
TG(12:0_12:0_14:0)	C41 H78 O6	9.97	666.58648	667.59376	20.209	1

TG(10:0_18:1_18:3)	C49 H86 O6	5.94	770.64702	771.6543	18.719	1
SPH(t18:1)	C18 H37 N O3	2.52	315.27814	316.28522	4.534	2
SPH(t18:0)	C18 H39 N O3	-5.68	317.29119	318.30057	3.257	2
SPH(d24:0)	C24 H51 N O2	3.04	385.39315	386.40042	12.19	2
SPH(d22:0)	C22 H47 N O2	2.58	357.3616	358.36888	8.472	2
SPH(d21:1)	C21 H43 N O2	0.61	341.32959	342.33686	9.043	2
SPH(d20:1)	C20 H41 N O2	0.87	327.31402	328.32128	7.866	2
SPH(d19:1)	C19 H39 N O2	0.16	313.29813	314.3054	7.843	2
SPH(d18:1)	C18 H37 N O2	0.94	299.28271	341.3166	3.653	2
SPH(d18:0)	C18 H39 N O2	1.23	301.29845	302.30573	4.329	2
SPH(d16:1)	C16 H33 N O2	1.21	271.25146	272.25873	4.064	1
SM(t42:4)	C47 H89 N2 O	1.21	824.64173	825.64903	11.764	2
SM(t42:3)	C47 H91 N2 O	0.79	826.65704	827.66431	11.412	2
SM(t40:4)	C45 H85 N2 O	-0.43	796.6091	797.61797	9.159	2
SM(t34:2)	C39 H77 N2 O	0.91	716.54749	717.55477	11.114	2
SM(t34:1)	C39 H79 N2 O	1.18	718.56334	719.57062	12.458	2
SM(t34:0)	C39 H81 N2 O	-3.4	720.57569	762.60913	12.567	2
SM(t18:0_16:1)	C39 H79 N2 O	1.38	718.56348	719.57076	10.845	2
SM(d44:5)	C49 H91 N2 O	1.59	834.6628	835.67007	16.352	2
SM(d43:3)	C48 H93 N2 O	0.76	824.67775	825.68503	18.082	2
SM(d43:2)	C48 H95 N2 O	0.98	826.69359	827.70086	19.171	2
SM(d43:1)	C48 H97 N2 O	0.84	828.70912	829.7164	24.007	2
SM(d42:5)	C47 H87 N2 O	0.97	806.63096	807.63825	14.802	2
SM(d42:4)	C47 H89 N2 O	0.91	808.64656	809.65383	16.192	2
SM(d42:3)	C47 H91 N2 O	-1.84	810.65998	811.66852	17.957	2
SM(d42:2)	C47 H93 N2 O	0.61	812.67762	813.68489	18.455	2
SM(d41:3)	C46 H89 N2 O	0.33	796.64609	797.6534	16.445	2
SM(d41:2)	C46 H91 N2 O	-0.23	798.66129	799.66857	18.806	2
SM(d41:1)	C46 H93 N2 O	0.12	800.67722	801.6845	19.553	2
SM(d40:4)	C45 H85 N2 O	1.36	780.61559	781.62286	14.345	2
SM(d40:3)	C45 H87 N2 O	1.15	782.63108	783.63835	15.781	2
SM(d40:1)	C45 H91 N2 O	1.17	786.6624	787.66967	18.564	2
SM(d39:2)	C44 H87 N2 O	1.01	770.63096	771.63823	16.44	2
SM(d39:1)	C44 H89 N2 O	0.97	772.64658	773.65385	17.929	2
SM(d38:5)	C43 H79 N2 O	-0.27	750.56737	751.57465	13.63	2

SM(d38:4)	C43 H81 N2 O	-2.9	752.58104	753.58832	15.519	1
SM(d38:3)	C43 H83 N2 O	1.01	754.59963	755.60692	13.739	2
SM(d38:1)	C43 H87 N2 O	0.87	758.63083	759.63811	17.002	2
SM(d37:2)	C42 H83 N2 O	1.07	742.59967	743.60703	14.583	2
SM(d37:1)	C42 H85 N2 O	0.25	744.61471	745.62199	15.986	2
SM(d36:5)	C41 H75 N2 O	-1.79	722.53498	723.54226	11.855	2
SM(d36:4)	C41 H77 N2 O	-2.38	724.5502	725.55709	13.327	2
SM(d36:3)	C41 H79 N2 O	1.4	726.56859	727.57587	12.653	2
SM(d36:2D9)	C41 H72 D9 N	1	737.64046	738.64774	13.523	2
SM(d36:2)	C41 H81 N2 O	0.91	728.58389	729.59121	13.992	2
SM(d36:0)	C41 H85 N2 O	1.9	732.61592	733.62319	15.653	2
SM(d35:4)	C40 H75 N2 O	-3.05	710.53411	711.54138	12.693	1
SM(d35:2)	C40 H79 N2 O	1.45	714.56861	715.57589	12.715	2
SM(d35:1)	C40 H81 N2 O	0.85	716.58383	717.59111	14.225	2
SM(d34:3)	C39 H75 N2 O	0.95	698.53693	699.54421	10.662	2
SM(d34:0)	C39 H81 N2 O	-0.15	704.58312	705.59039	14.165	2
SM(d33:2)	C38 H75 N2 O	0.93	686.53691	687.54419	11.045	2
SM(d33:1)	C38 H77 N2 O	0.89	688.55254	689.55972	12.73	2
SM(d33:0)	C38 H79 N2 O	0.99	690.56826	691.57554	13.038	2
SM(d32:2)	C37 H73 N2 O	1.37	672.52154	673.52883	10.192	2
SM(d32:1)	C37 H75 N2 O	1.05	674.53698	675.54426	11.472	2
SM(d32:0)	C37 H77 N2 O	1.38	676.55286	677.56015	12.103	2
SM(d31:1)	C36 H73 N2 O	1.56	660.52166	661.52893	10.652	2
SM(d30:2)	C35 H69 N2 O	1.8	644.49048	645.49772	8.754	2
SM(d30:1)	C35 H71 N2 O	1.4	646.50588	647.51316	9.904	2
SM(d28:1)	C33 H67 N2 O	1.56	618.47464	619.48193	8.56	2
SM(d20:1_20:1)	C45 H89 N2 O	1.21	784.64678	785.65404	16.972	2
SM(d19:1_17:0)	C41 H83 N2 O	0.31	730.5991	731.60685	15.459	2
SM(d19:0_23:1)	C47 H95 N2 O	1.86	814.69429	815.70156	23.147	2
SM(d18:1_24:2)	C47 H91 N2 O	1	810.66228	811.66957	17.155	2
SM(d18:1_24:1)	C47 H93 N2 O	0.51	812.67754	813.68482	19.534	2
SM(d18:1_23:1)	C46 H91 N2 O	1.37	798.66257	799.66985	17.757	2
SM(d18:1_16:1)	C39 H77 N2 O	1.87	700.55324	701.56051	11.789	2
SM(d17:1_21:1)	C43 H85 N2 O	1.46	756.61563	757.62291	15.443	2
SM(d16:1_18:0)	C39 H79 N2 O	1.41	702.56857	703.57585	13.56	2

SiE(18:3)	C47 H78 O2	2.98	674.60219	675.60794	19.178	2
PI(18:1_20:4)	C47 H81 O13	0.4	884.54183	902.57568	11.183	2
PI(18:1_18:2)	C45 H81 O13	0.23	860.54168	878.57559	11.526	2
PI(18:0_22:6)	C49 H83 O13	0.99	910.55803	928.59183	12.144	2
PI(18:0_20:5)	C47 H81 O13	0.87	884.54225	885.54946	9.896	2
PI(18:0_20:4)	C47 H83 O13	0.33	886.55743	904.59116	12.728	2
PI(18:0_20:3)	C47 H85 O13	0.23	888.57298	906.60675	13.317	2
PI(18:0_20:2)	C47 H87 O13	0.7	890.58906	908.62294	14.06	2
PI(18:0_18:2)	C45 H83 O13	0.08	862.5572	863.56444	12.873	2
PI(18:0_18:1)	C45 H85 O13	0.58	864.57328	882.60713	13.771	2
PI(16:0_22:6)	C47 H79 O13	1.23	882.52692	900.56104	10.721	2
PI(16:0_20:4)	C45 H79 O13	1.09	858.52676	876.56067	10.985	2
PI(16:0_18:2)	C43 H79 O13	1.33	834.52694	852.56077	11.11	2
PG(36:2)	C42 H79 O10	1	774.54186	792.57593	13.374	1
PG(20:4D8_18:1)	C44 H69 D8 O	-1.95	804.57408	805.58136	16.285	1
PG(16:0_17:0)	C39 H77 O10	3.78	736.52822	737.5339	12.926	2
PE(62:1)	C67 H132 N O	3.02	1109.97241	1110.97969	21.79	1
PE(46:1D4)	C51 H96 D4 N	-6.78	889.73773	890.74504	18.38	2
PE(40:5p)	C45 H80 N O7	1.03	777.56804	778.57532	16.119	2
PE(37:5e)	C42 H76 N O7	1.15	737.53679	738.54407	15.222	2
PE(36:2e)	C41 H80 N O7	2.96	729.5694	730.57513	15.994	1
PE(36:1D3)	C41 H77 D3 N	-1.63	748.57977	749.58789	16.697	2
PE(35:1)	C40 H78 N O8	1.57	731.54766	732.55493	18.218	2
PE(35:0e)	C40 H82 N O7	0.97	719.58359	720.59083	17.534	2
PE(34:2e)	C39 H76 N O7	0.11	701.53602	702.54357	14.865	2
PE(33:0e)	C38 H78 N O7	4.77	691.55489	692.56219	17.053	2
PE(31:1)	C36 H70 N O8	4.56	675.48699	676.49426	20.72	3
PE(31:0D7)	C36 H65 D7 N	-3.45	684.54113	748.55774	12.833	2
PE(22:5e)	C27 H46 N O7	1.52	527.30199	528.30927	4.707	1
PE(21:4)	C26 H44 N O8	1.27	529.28113	530.2884	4.348	1
PE(20:4e)	C25 H44 N O7	0.49	501.28578	502.29306	4.467	2
PE(20:4e_18:1)	C43 H78 N O7	-1.77	751.55026	784.5838	18.064	1
PE(20:3e)	C25 H46 N O7	0.77	503.30158	504.30885	4.838	2
PE(20:0p_20:4)	C45 H82 N O7	1.12	779.58376	780.59116	16.87	2
PE(20:0p_18:2)	C43 H82 N O7	1.48	755.58401	756.59083	17.32	2

PE(19:1_19:1)	C43 H82 N O8	1.77	771.57917	772.58641	16.318	1
PE(19:1_18:1)	C42 H80 N O8	0.86	757.5628	758.57008	16.065	2
PE(18:3e)	C23 H42 N O7	1.47	475.27059	476.27786	3.497	1
PE(18:2p_18:2)	C41 H74 N O7	1.58	723.52143	724.52871	13.721	2
PE(18:2e)	C23 H44 N O7	1.26	477.28614	478.29342	4.168	2
PE(18:2_20:4)	C43 H74 N O8	1.58	763.51641	764.52369	12.57	2
PE(18:2_18:2)	C41 H74 N O8	1.53	739.51634	740.52356	12.78	2
PE(18:1p_22:6)	C45 H76 N O7	-0.56	773.53551	774.54379	14.483	2
PE(18:1p_20:5)	C43 H74 N O7	1.1	747.52112	748.52839	10.938	2
PE(18:1p_18:2)	C41 H76 N O7	1.24	725.53684	726.5442	14.818	2
PE(18:1e_20:4)	C43 H78 N O7	0.55	751.552	752.55923	14.025	2
PE(18:1D7_15:0)	C38 H67 D7 N	0.8	710.55971	711.56699	14.044	1
PE(18:1_22:6)	C45 H76 N O8	1.46	789.532	790.53928	13.454	2
PE(18:1_20:4)	C43 H76 N O8	1.21	765.53178	766.53906	13.719	2
PE(18:1_18:2)	C41 H76 N O8	0.17	741.53098	742.53675	14.43	2
PE(18:1_18:1)	C41 H78 N O8	1.21	743.54741	744.55471	14.036	2
PE(18:0p_22:6)	C45 H78 N O7	0.89	775.55228	776.55955	15.67	2
PE(18:0p_20:5)	C43 H76 N O7	1.16	749.53681	750.54408	12.576	2
PE(18:0p_20:3)	C43 H80 N O7	-0.04	753.56721	754.57449	16.794	1
PE(18:0p_18:2)	C41 H78 N O7	1.15	727.55243	728.55973	16.201	2
PE(18:0_20:5)	C43 H76 N O8	1.14	765.53173	766.53899	11.325	2
PE(18:0_20:4)	C43 H78 N O8	0.86	767.54717	768.55446	15.506	2
PE(18:0_18:3)	C41 H76 N O8	-0.31	741.53063	742.5379	11.048	1
PE(18:0_18:2)	C41 H78 N O8	1.28	743.54746	744.55475	15.694	2
PE(18:0_18:1)	C41 H80 N O8	1.77	745.56347	746.57038	16.401	2
PE(17:1_19:1)	C41 H78 N O8	1.14	743.54735	744.55463	18.039	2
PE(17:1_18:1)	C40 H76 N O8	0.92	729.53153	730.5388	14.611	2
PE(17:1_16:0)	C38 H74 N O8	4.28	703.51822	704.52549	13.868	2
PE(17:0_20:4)	C42 H76 N O8	-1.4	753.5298	754.53708	14.577	2
PE(16:0p_22:6)	C43 H74 N O7	0.68	747.5208	748.52807	14.049	2
PE(16:0p_22:5)	C43 H76 N O7	0.89	749.53661	750.54388	14.957	2
PE(16:0p_22:4)	C43 H78 N O7	0.85	751.55223	752.55952	16.018	2
PE(16:0p_20:5)	C41 H72 N O7	0.99	721.50536	722.51263	10.569	2
PE(16:0p_20:4)	C41 H74 N O7	0.57	723.5207	724.52799	14.627	2
PE(16:0p_18:2)	C39 H74 N O7	1.24	699.52116	700.52844	14.6	2

PE(16:0p_18:1)	C39 H76 N O7	0.79	701.53649	702.54381	15.769	2
PE(16:0e)	C21 H44 N O7	1.56	453.28624	454.29352	5.047	2
PE(16:0_22:6)	C43 H74 N O8	1.15	763.51608	764.52336	13.229	2
PE(16:0_20:4)	C41 H74 N O8	1.31	739.51617	740.52348	13.844	2
PE(16:0_18:2)	C39 H74 N O8	-0.03	715.51518	716.52293	14.001	2
PE(16:0_18:1)	C39 H76 N O8	1.33	717.53181	718.53907	14.9	2
PC(8:0e_16:0)	C32 H66 N O7	1.68	607.45871	608.46598	12.19	2
PC(8:0_20:3)	C36 H66 N O8	1.32	671.45349	672.46125	7.427	1
PC(44:6e)	C52 H94 N O7	0.53	875.67725	876.68453	19.211	2
PC(44:5e)	C52 H96 N O7	1.1	877.6934	878.70068	23.572	2
PC(44:5)	C52 H94 N O8	-0.07	891.67165	892.67892	12.948	2
PC(44:4e)	C52 H98 N O7	0.57	879.70859	880.71587	25.829	2
PC(44:2)	C52 H100 N O	-0.59	897.71813	898.7254	25.856	2
PC(42:6e)	C50 H90 N O7	0.61	847.64601	848.65318	17.337	2
PC(42:5e)	C50 H92 N O7	0.39	849.66147	850.66875	18.686	2
PC(42:5)	C50 H90 N O8	-8.2	863.63333	864.6406	11.221	2
PC(42:4e)	C50 H94 N O7	1.1	851.67772	852.685	23.851	2
PC(42:2e)	C50 H98 N O7	1.02	855.70897	856.71624	26.552	2
PC(42:10)	C50 H80 N O8	0.61	853.56267	854.56995	12.223	2
PC(40:9)	C48 H78 N O8	-2.48	827.54445	828.55173	13.633	1
PC(40:7)	C48 H82 N O8	-2.47	831.57575	832.58303	15.795	2
PC(40:4)	C48 H88 N O8	3.19	837.62743	838.63448	13.268	2
PC(40:3e)	C48 H92 N O7	2.19	825.66295	826.67023	19.329	2
PC(40:2e)	C48 H94 N O7	1.14	827.67773	828.685	24.741	2
PC(40:1e)	C48 H96 N O7	0.92	829.69321	830.70048	25.945	2
PC(40:1)	C48 H94 N O8	-0.32	843.67143	844.67871	23.595	2
PC(4:0_14:1)	C26 H50 N O8	1.58	535.32825	536.33552	2.863	2
PC(38:8e)	C46 H78 N O7	-2.36	787.54973	788.55701	14.831	2
PC(38:7D5)	C46 H73 D5 N	1.79	808.57933	809.58661	16.103	1
PC(38:7)	C46 H78 N O8	-2.75	803.5443	804.55157	13.148	2
PC(38:6D8)	C46 H72 D8 N	4.33	813.61589	814.62317	16.665	1
PC(38:6D3)	C46 H77 D3 N	0.05	808.58103	809.5883	13.637	1
PC(38:5)	C46 H82 N O8	0.2	807.57796	808.58524	15.368	1
PC(38:4)	C46 H84 N O8	0.78	809.59409	810.60137	16.281	2
PC(37:6)	C45 H78 N O8	1.5	791.54769	792.55469	14.864	2

PC(37:5e)	C45 H82 N O7	0.4	779.5832	780.59048	15.847	2
PC(37:5)	C45 H80 N O8	1.11	793.56304	794.57031	14.993	1
PC(37:4e)	C45 H84 N O7	0.8	781.59917	782.60647	16.105	2
PC(36:5D8)	C44 H70 D8 N	4.58	787.60032	788.6076	15.968	1
PC(36:5)	C44 H78 N O8	-2.11	779.54486	780.55213	14.139	2
PC(36:4D8)	C44 H72 D8 N	5.16	789.61644	790.62372	17.643	3
PC(36:4D3)	C44 H77 D3 N	1.15	784.58189	785.58916	15.062	2
PC(36:4)	C44 H80 N O8	1.66	781.56345	782.57073	11.258	2
PC(36:2)	C44 H84 N O8	0.05	785.59349	786.60077	17.892	2
PC(36:1)	C44 H86 N O8	1.32	787.61015	788.61742	12.548	2
PC(35:4e)	C43 H80 N O7	0.48	753.5676	754.57488	14.667	2
PC(35:4D7)	C43 H71 D7 N	-2.5	774.5885	775.59578	14.706	2
PC(35:3e)	C43 H82 N O7	-0.35	755.58263	756.5899	16.182	1
PC(35:2e)	C43 H84 N O7	0.22	757.59871	758.60598	17.857	2
PC(35:0)	C43 H86 N O8	0.65	775.60961	776.61688	18.955	1
PC(34:5D8)	C42 H66 D8 N	4.23	759.56863	760.57591	14.176	2
PC(34:3e)	C42 H80 N O7	-1.82	741.56589	742.57532	14.446	2
PC(34:2)	C42 H80 N O8	1.9	757.5636	758.57003	10.232	2
PC(34:1)	C42 H82 N O8	3.76	759.58066	760.58597	10.819	2
PC(34:0)	C42 H84 N O8	3.55	761.59616	762.60136	14.529	2
PC(33:3e)	C41 H78 N O7	-1.21	727.55071	728.55798	14.418	2
PC(33:3)	C41 H76 N O8	0.99	741.53158	742.53886	14.199	2
PC(33:2e)	C41 H80 N O7	-2.6	729.56534	730.57518	15.055	2
PC(33:1e)	C41 H82 N O7	1.18	731.58376	732.59103	15.975	2
PC(33:1D9)	C41 H71 D9 N	-4.24	754.61545	755.62272	15.005	1
PC(33:1D7)	C41 H73 D7 N	-2.23	752.60441	753.61336	15.313	2
PC(33:0)	C41 H82 N O8	-0.05	747.57777	748.58504	16.831	2
PC(32:4D8)	C40 H64 D8 N	5.01	733.55345	734.56072	14.245	1
PC(32:3e)	C40 H76 N O7	1.01	713.53666	714.54394	13.343	2
PC(32:1D3)	C40 H75 D3 N	1.95	734.56677	735.57406	15.5	2
PC(32:1)	C40 H78 N O8	1.84	731.54785	732.55517	15.5	2
PC(32:1_6:0)	C46 H90 N O8	0.95	815.64118	816.64845	19.172	2
PC(31:1e)	C39 H78 N O7	-4.33	703.54855	704.55974	15.449	2
PC(31:1)	C39 H76 N O8	3.01	717.53302	718.5394	13.598	2
PC(31:0)	C39 H78 N O8	4.12	719.54947	720.56075	15.111	1

PC(30:1)	C38 H74 N O8	1.44	703.51622	704.5236	12.879	2
PC(26:3)	C34 H62 N O8	1.93	643.42255	644.42984	6.542	2
PC(26:2)	C34 H64 N O8	0.53	645.4373	646.44457	8.126	2
PC(26:1)	C34 H66 N O8	0.95	647.45322	648.4605	9.327	2
PC(26:1_16:0)	C50 H98 N O8	-0.79	871.70232	872.70959	26.109	3
PC(24:5e)	C32 H56 N O7	0.62	597.37981	598.38708	6.254	1
PC(24:0_20:4)	C52 H96 N O8	0.41	893.68772	894.695	24.277	2
PC(24:0_18:2)	C50 H96 N O8	0.76	869.68802	870.69528	24.948	2
PC(23:1)	C31 H60 N O8	5.43	605.40894	606.41622	6.286	1
PC(22:5_18:2)	C48 H82 N O8	1.42	831.57898	832.58626	13.711	2
PC(22:5_14:1)	C44 H76 N O8	-1.95	777.52934	778.53644	13.128	2
PC(22:4)	C30 H52 N O8	1.5	585.34393	586.35121	2.991	2
PC(22:4_20:4)	C50 H84 N O8	0.7	857.59406	858.60134	13.838	2
PC(22:3)	C30 H54 N O8	1.45	587.35956	588.36684	3.164	2
PC(22:1_20:4)	C50 H90 N O8	0.59	863.64091	864.64819	17.316	2
PC(21:4)	C29 H50 N O8	1.16	571.32806	572.33534	4.384	2
PC(21:1)	C29 H56 N O8	1.58	577.37526	578.38254	5.85	1
PC(21:0)	C29 H58 N O8	1.2	579.3907	580.3979	6.159	2
PC(20:5_18:2)	C46 H78 N O8	-2.81	803.54424	804.55152	14.041	2
PC(20:4)	C28 H48 N O8	-0.45	557.3115	558.31878	2.389	2
PC(20:4_20:4)	C48 H80 N O8	1.7	829.56356	830.57084	12.545	2
PC(20:3e_18:1)	C46 H86 N O7	-0.77	795.61357	796.62085	17.339	2
PC(20:3)	C28 H50 N O8	1.15	559.32805	560.33565	2.257	2
PC(20:3_22:6)	C50 H82 N O8	0.74	855.57843	856.58571	13.292	2
PC(20:3_18:2)	C46 H82 N O8	0.75	807.57841	808.5857	13.707	2
PC(20:2)	C28 H52 N O8	1.92	561.34413	562.35107	2.514	1
PC(20:2_18:2)	C46 H84 N O8	1.03	809.59429	810.60156	15.058	2
PC(20:1)	C28 H54 N O8	1.93	563.35979	564.36678	3.098	2
PC(20:0e_20:4)	C48 H90 N O7	0.8	823.64615	824.65341	18.812	2
PC(20:0e_18:2)	C46 H90 N O7	0.42	799.64583	800.6531	19.702	2
PC(20:0e_18:1)	C46 H92 N O7	0.05	801.66118	802.66846	23.326	2
PC(20:0_18:2)	C46 H88 N O8	1.74	813.62617	814.63339	16.271	2
PC(19:3e)	C27 H50 N O7	-0.09	531.33244	532.33936	5.713	2
PC(19:2)	C27 H50 N O8	1.22	547.32807	548.33535	4.51	2
PC(19:1)	C27 H52 N O8	0.65	549.34341	550.35069	6.001	2

PC(19:1_19:1)	C46 H88 N O8	0.57	813.62522	814.6325	17.331	2
PC(19:1_18:1)	C45 H86 N O8	0.93	799.60985	800.61713	19.688	2
PC(19:0_20:4)	C47 H86 N O8	0.53	823.60954	824.61681	16.27	2
PC(19:0_20:3)	C47 H88 N O8	0.16	825.62488	826.63217	17.387	2
PC(19:0_18:1)	C45 H88 N O8	0.84	801.62543	802.6327	18.147	2
PC(18:3e_24:0)	C50 H96 N O7	0.5	853.69287	854.70014	24.578	2
PC(18:3e_20:4)	C46 H80 N O7	1.21	789.56819	790.57547	13.667	2
PC(18:3e_18:2)	C44 H80 N O7	0.65	765.56774	766.57501	13.903	2
PC(18:3_18:2)	C44 H78 N O8	1.55	779.54771	780.55499	12.055	2
PC(18:2e_20:4)	C46 H82 N O7	1.57	791.58413	792.59141	14.03	2
PC(18:2e_18:2)	C44 H82 N O7	0.58	767.58334	768.59061	14.382	2
PC(18:2)	C26 H48 N O8	1.3	533.31245	534.31971	2.399	2
PC(18:2_23:0)	C49 H94 N O8	0.47	855.67211	856.67938	23.683	1
PC(18:2_20:4)	C46 H80 N O8	1.21	805.56313	806.57041	12.887	2
PC(18:2_18:2)	C44 H80 N O8	1.03	781.56296	782.57024	14.292	2
PC(18:1e_22:6)	C48 H84 N O7	0.97	817.59933	818.60661	16.23	2
PC(18:1e_22:4)	C48 H88 N O7	0.82	821.63052	822.63779	16.996	2
PC(18:1e_20:4)	C46 H84 N O7	0.4	793.59886	794.60613	16.142	2
PC(18:1e_18:2)	C44 H84 N O7	1.66	769.59982	770.6071	16.937	2
PC(18:1e_18:1)	C44 H86 N O7	0.7	771.61473	772.62199	18.177	2
PC(18:1D7_15:0)	C41 H73 D7 N	0.68	752.6066	753.61388	14.664	2
PC(18:1)	C26 H50 N O8	1.9	535.32842	518.32513	2.065	1
PC(18:1_24:0)	C50 H98 N O8	-1.49	871.70171	872.70898	25.405	2
PC(18:1_22:0)	C48 H94 N O8	0.34	843.67199	844.67927	24.331	2
PC(18:1_20:4)	C46 H82 N O8	-2.2	807.57603	808.5833	16.127	2
PC(18:1_18:3)	C44 H80 N O8	-9.41	781.5548	782.56217	9.551	1
PC(18:1_18:2)	C44 H82 N O8	0.9	783.57851	784.58579	14.371	2
PC(18:1_18:1)	C44 H84 N O8	-0.19	785.5933	786.60058	16.494	2
PC(18:0e_22:6)	C48 H86 N O7	1.68	819.61556	820.62284	16.606	2
PC(18:0e_20:3)	C46 H88 N O7	1.24	797.63083	798.6381	17.774	2
PC(18:0e_18:2)	C44 H86 N O7	0.85	771.61485	772.62213	17.256	2
PC(18:0_8:0)	C34 H68 N O8	0.69	649.4687	650.47598	10.548	2
PC(18:0_22:6)	C48 H84 N O8	0.95	833.59424	834.60153	14.849	2
PC(18:0_22:5)	C48 H86 N O8	0.61	835.60961	836.6169	16.558	2
PC(18:0_22:4)	C48 H88 N O8	0.57	837.62523	838.63251	17.449	2

PC(18:0_20:4)	C46 H84 N O8	1.6	809.59475	810.60203	15.207	2
PC(18:0_20:3)	C46 H86 N O8	0.51	811.60952	812.61679	17.321	2
PC(18:0_18:3)	C44 H82 N O8	0.51	783.5782	784.58547	15.358	2
PC(18:0_18:2)	C44 H84 N O8	0.8	785.59408	786.60136	16.715	2
PC(18:0_18:1)	C44 H86 N O8	-0.14	787.609	788.61627	18.006	2
PC(18:0_18:0)	C44 H88 N O8	0.63	789.62525	790.63253	19.181	3
PC(18:0_16:0)	C42 H84 N O8	0.38	761.59374	762.60101	18.053	2
PC(17:1_22:6)	C47 H80 N O8	1.68	817.56353	818.57104	12.942	2
PC(17:1_20:4)	C45 H80 N O8	0.73	793.56273	794.57011	13.984	2
PC(17:1_18:2)	C43 H80 N O8	1.12	769.56302	770.57032	13.724	2
PC(17:0_22:6)	C47 H82 N O8	0.89	819.57853	820.58581	14.418	2
PC(17:0_22:5)	C47 H84 N O8	1.05	821.59432	822.60161	15.006	2
PC(17:0_20:4)	C45 H82 N O8	1.05	795.57864	796.58591	14.855	2
PC(17:0_20:3)	C45 H84 N O8	0.68	797.59399	798.60127	16.194	2
PC(17:0_18:3)	C43 H80 N O8	0.94	769.56288	770.57018	14.089	2
PC(17:0_18:2)	C43 H82 N O8	1.25	771.57877	772.58571	15.781	1
PC(17:0_18:1)	C43 H84 N O8	1.1	773.5943	774.60158	16.46	2
PC(16:1e_22:6)	C46 H80 N O7	0.89	789.56794	790.57522	14.299	2
PC(16:1e_22:5)	C46 H82 N O7	0.41	791.58322	792.59049	16.025	1
PC(16:1e_22:4)	C46 H84 N O7	0.41	793.59886	794.60614	15.933	2
PC(16:1e_20:5)	C44 H78 N O7	1.39	763.55265	764.55993	13.636	2
PC(16:1e_20:4)	C44 H80 N O7	1.13	765.5681	766.57538	14.762	2
PC(16:1e_20:3)	C44 H82 N O7	0.97	767.58364	768.59091	16.114	2
PC(16:1e_18:3)	C42 H78 N O7	0.21	739.55174	740.55902	14.087	2
PC(16:1e_18:2)	C42 H80 N O7	1.24	741.56816	742.57545	15.54	2
PC(16:1e_18:1)	C42 H82 N O7	-0.52	743.58251	744.58978	16.096	1
PC(16:1e_16:1)	C40 H78 N O7	0.83	715.55218	716.55947	14.624	2
PC(16:1e_16:0)	C40 H80 N O7	0.7	717.56775	718.5751	16.226	2
PC(16:1_22:6)	C46 H78 N O8	1.24	803.5475	804.55478	12.2	2
PC(16:1_20:3)	C44 H80 N O8	-2.43	781.56025	782.56753	15.639	2
PC(16:1_18:2)	C42 H78 N O8	0.41	755.54682	756.55409	13.909	1
PC(16:1_18:1)	C42 H80 N O8	0.64	757.56264	758.56992	14.551	2
PC(16:0e_20:4)	C44 H82 N O7	0.92	767.58359	768.59087	15.535	2
PC(16:0e_20:3)	C44 H84 N O7	1.06	769.59936	770.60663	15.797	2
PC(16:0e_18:2)	C42 H82 N O7	1.5	743.584	744.59129	15.495	2

PC(16:0e_18:1)	C42 H84 N O7	1.85	745.59992	746.60719	16.792	2
PC(16:0e_16:0)	C40 H82 N O7	1.33	719.58385	720.59111	16.605	2
PC(16:0e_14:0)	C38 H78 N O7	0.78	691.55213	692.5594	14.71	2
PC(16:0D9_22:6)	C46 H71 D9 N	0.97	814.61943	815.62671	16.95	1
PC(16:0_8:0)	C32 H64 N O8	2.96	621.43879	644.42884	8.672	1
PC(16:0_22:6)	C46 H80 N O8	-2.29	805.56031	806.56759	14.675	2
PC(16:0_22:5)	C46 H82 N O8	1.25	807.57882	808.58609	14.572	2
PC(16:0_20:5)	C44 H78 N O8	1.06	779.54733	780.55461	12.462	2
PC(16:0_19:0)	C43 H86 N O8	2.76	775.61125	776.61867	18.468	1
PC(16:0_18:3)	C42 H78 N O8	1.15	755.54737	756.55465	13.265	2
PC(16:0_18:2)	C42 H80 N O8	0.49	757.56253	758.5698	15.148	2
PC(16:0_18:1)	C42 H82 N O8	0.78	759.57839	760.58567	16.113	2
PC(16:0_17:0)	C41 H82 N O8	0.28	747.57802	748.5853	16.272	2
PC(16:0_16:1)	C40 H78 N O8	1.13	731.54733	732.55461	14.337	2
PC(16:0_16:0)	C40 H80 N O8	0.93	733.56284	734.57012	15.338	2
PC(16:0_14:1)	C38 H74 N O8	1.59	703.51632	704.5236	12.023	2
PC(16:0_14:0)	C38 H76 N O8	1.29	705.53177	706.53905	13.489	2
PC(16:0_13:0)	C37 H74 N O8	1.32	691.51612	692.52339	12.597	2
PC(15:0_22:6)	C45 H78 N O8	0.85	791.54718	792.5545	12.484	2
PC(15:0_22:5)	C45 H80 N O8	1.36	793.56323	794.57052	13.708	2
PC(15:0_20:5)	C43 H76 N O8	-4.6	765.52733	766.53461	13.219	1
PC(15:0_20:4)	C43 H78 N O8	1.58	767.54771	768.55521	12.561	2
PC(15:0_18:3)	C41 H76 N O8	1.47	741.53195	742.53922	12.234	2
PC(15:0_18:2)	C41 H78 N O8	1.49	743.54761	744.55491	13.349	2
PC(15:0_18:1)	C41 H80 N O8	1.3	745.56312	746.5704	14.64	2
PC(15:0_16:1)	C39 H76 N O8	1.06	717.53162	718.53892	12.889	2
PC(15:0_16:0)	C39 H78 N O8	1.25	719.5474	720.55468	14.731	2
PC(14:0e_18:2)	C40 H78 N O7	0.63	715.55204	716.55967	13.726	2
PC(14:0_22:6)	C44 H76 N O8	1.21	777.5318	778.53906	11.865	2
PC(14:0_20:5)	C42 H74 N O8	0.86	751.51585	752.52313	11.166	2
PC(14:0_20:4)	C42 H76 N O8	1.51	753.53199	754.53926	12.212	2
PC(14:0_18:3)	C40 H74 N O8	1.32	727.51616	728.52344	11.376	2
PC(14:0_18:2)	C40 H76 N O8	1.32	729.53182	730.5391	12.446	2
PC(14:0_14:0)	C36 H72 N O8	1.38	677.50049	678.50777	11.757	2
PC(12:0_18:2)	C38 H72 N O8	1.42	701.50055	702.50782	10.81	2

PC(11:0_23:1)	C42 H82 N O8	-5.17	759.57388	760.58567	19.081	1
MGDG(19:1_19:1)	C47 H86 O10	5.05	810.62619	811.63347	18.329	1
MGDG(18:2_18:2)	C45 H78 O10	-9.41	778.55217	779.55945	13.183	1
MGDG(16:1_15:0)	C40 H74 O10	-9.76	714.52123	715.5285	13.537	2
MGDG(16:0_16:1)	C41 H76 O10	2.5	728.54567	1480.0791	13.998	2
MGDG(16:0_15:0)	C40 H76 O10	-7.51	716.53847	717.54574	14.588	2
MG(20:4e)	C23 H40 O3	0.97	364.2981	365.3054	8.251	2
MG(18:3)	C21 H36 O4	1.62	352.26193	353.26931	3.23	2
MG(18:1)	C21 H40 O4	1.67	356.29325	357.30027	6.393	2
MG(18:0)	C21 H42 O4	0.98	358.30866	359.31586	1.004	2
MG(16:2e)	C19 H36 O3	0.76	312.26668	313.27395	5.525	2
MePC(36:4)	C45 H82 N O8	0.66	795.57833	796.5856	15.246	2
MePC(35:0)	C44 H88 N O8	1.53	789.62596	790.63324	16.88	1
MePC(34:4)	C43 H78 N O8	-1.49	767.54536	768.55438	13.412	2
MePC(34:3)	C43 H80 N O8	6.89	769.56746	770.5706	14.858	1
MePC(33:3)	C42 H78 N O8	1.52	755.54766	756.55493	10.785	1
MePC(32:1)	C41 H80 N O8	1.58	745.56334	746.57062	15.527	2
MePC(30:2)	C39 H74 N O8	1.76	715.51647	716.52374	14.489	1
MePC(29:0)	C38 H76 N O8	1.48	705.5319	706.53918	14.357	2
MePC(21:5e)	C30 H52 N O7	0.65	569.34851	570.3558	5.5	2
MePC(16:1e)	C25 H50 N O7	1.26	507.33313	508.34035	5.312	2
MePC(14:1e)	C23 H46 N O7	1.22	479.30177	480.30905	5.082	2
MePC(14:0e)	C23 H48 N O7	0.81	481.31723	482.32452	7.256	2
LPE(22:6)	C27 H44 N O7	1.5	525.28633	526.29358	3.981	2
LPE(18:0)	C23 H48 N O7	2.79	481.31818	482.3254	6.908	2
LPC(38:1)	C46 H92 N O7	1.21	801.66211	802.66919	23.951	2
LPC(32:1)	C40 H80 N O7	1.37	717.56822	718.5755	15.626	1
LPC(31:0)	C39 H80 N O7	0.99	705.56794	706.57535	15.775	2
LPC(24:1)	C32 H64 N O7	1.4	605.44288	606.45016	10.256	2
LPC(22:6)	C30 H50 N O7	1.64	567.33342	568.3407	4	2
LPC(22:5)	C30 H52 N O7	0.59	569.34848	570.35577	5.175	2
LPC(22:4)	C30 H54 N O7	0.48	571.36406	572.37134	5.211	1
LPC(22:2)	C30 H58 N O7	1.6	575.39601	576.40329	7.453	2
LPC(22:1)	C30 H60 N O7	1.99	577.41189	578.41917	8.173	1
LPC(22:0)	C30 H62 N O7	1.34	579.42717	580.43444	10.221	2

LPC(20:5)	C28 H48 N O7	-2.42	541.31553	542.3224	4.995	2
LPC(20:4e)	C28 H52 N O6	-3.63	529.3513	530.35858	7.337	1
LPC(20:4)	C28 H50 N O7	1.11	543.33309	544.34038	4.778	2
LPC(20:3e)	C28 H54 N O6	-2.63	531.36748	532.37476	8.273	1
LPC(20:3)	C28 H52 N O7	-2.7	545.34666	546.35394	7.438	2
LPC(20:2e)	C28 H56 N O6	1.31	533.38523	534.3925	6.996	1
LPC(20:2)	C28 H54 N O7	1.26	547.36448	548.37172	5.575	2
LPC(20:1e)	C28 H58 N O6	1.63	535.40105	536.40833	9.575	1
LPC(20:1)	C28 H56 N O7	1.65	549.38035	550.38764	7.017	2
LPC(20:0e)	C28 H60 N O6	1.31	537.41653	538.4238	9.531	2
LPC(20:0)	C28 H58 N O7	2.28	551.39635	552.40363	7.444	1
LPC(19:1)	C27 H54 N O7	1.42	535.36455	536.37183	6.615	2
LPC(19:0)	C27 H56 N O7	1.02	537.37999	538.38726	7.997	2
LPC(18:4)	C26 H46 N O7	1.97	515.30221	516.30948	3.257	2
LPC(18:3e)	C26 H50 N O6	1.63	503.3384	504.34567	5.049	2
LPC(18:3)	C26 H48 N O7	1.26	517.31749	518.32477	2.083	1
LPC(18:2e)	C26 H52 N O6	0.07	505.35326	506.36053	5.298	2
LPC(18:2)	C26 H50 N O7	1.17	519.3331	520.34037	5.066	2
LPC(18:1e)	C26 H54 N O6	1.8	507.36979	508.37706	6.573	2
LPC(18:1D7)	C26 H45 D7 N	0.76	528.39248	529.39976	5.639	2
LPC(18:1)	C26 H52 N O7	1.6	521.34897	522.35625	5.734	2
LPC(18:0e)	C26 H56 N O6	1.47	509.38527	510.39257	7.734	2
LPC(18:0)	C26 H54 N O7	0.95	523.36429	524.37157	7.529	2
LPC(17:1)	C25 H50 N O7	1.23	507.33311	508.34039	4.452	2
LPC(17:0)	C25 H52 N O7	1.27	509.34878	510.35612	6.552	2
LPC(16:2e)	C24 H48 N O6	6.67	477.32511	478.33239	5.152	1
LPC(16:1e)	C24 H50 N O6	1.92	479.33849	480.34577	5.832	2
LPC(16:1)	C24 H48 N O7	1.1	493.31738	494.32466	4.76	2
LPC(16:0e)	C24 H52 N O6	1.33	481.35387	482.36114	6.608	2
LPC(16:0)	C24 H50 N O7	1.43	495.3332	496.34048	5.643	2
LPC(15:0)	C23 H48 N O7	2.01	481.31781	482.32509	4.816	2
LPC(14:0)	C22 H46 N O7	2.07	467.30216	468.30944	3.6	2
LPC(12:0)	C20 H42 N O7	2.13	439.27083	440.278	2.806	2
LBPA(18:1_18:2)	C42 H77 O10	0.43	772.52576	790.55936	11.766	2
LBPA(18:0_18:1)	C42 H81 O10	0.42	776.55706	777.56402	14.141	2

LBPA(16:0_18:2)	C40 H75 O10	0.69	746.5103	747.51778	11.536	2
LBPA(16:0_18:1)	C40 H77 O10	1.57	748.52661	766.56055	16.303	2
LBPA(16:0_18:0)	C40 H79 O10	0.52	750.54147	768.57519	13.853	2
LBPA(16:0_16:1)	C38 H73 O10	0.88	720.49477	721.50228	11.181	2
LBPA(16:0_16:0)	C38 H75 O10	1.76	722.51105	740.54519	12.454	2
LBPA(14:0_16:0)	C36 H71 O10	1.5	694.47953	712.51343	10.923	2
Hex2Cer(d18:1_16:0)	C46 H87 N O1	1.85	861.61933	862.62661	12.2	2
Hex1Cer(m17:1_24:6)	C47 H79 N O7	6.14	769.59038	770.59766	15.736	1
Hex1Cer(d18:1_24:1)	C48 H91 N O8	0.44	809.67482	810.68262	17.456	2
Hex1Cer(d18:1_16:0)	C40 H77 N O8	0.87	699.56553	700.57277	13.38	2
DG(8:0_12:2)	C23 H40 O5	0.84	396.28791	397.29517	19.317	2
DG(38:5e)	C41 H72 O4	1.56	628.54404	629.55132	13.029	2
DG(38:2e)	C41 H78 O4	2.41	634.59154	635.59795	19.03	2
DG(36:6e)	C39 H66 O4	1.1	598.49677	599.50402	12.379	2
DG(36:6)	C39 H64 O5	1.66	612.47639	613.48367	7.547	1
DG(36:5e)	C39 H68 O4	1.88	600.51289	601.52016	20.063	2
DG(36:5)	C39 H66 O5	-2.64	614.4894	615.49668	16.886	2
DG(36:4e)	C39 H70 O4	1.14	602.5281	603.53538	12.842	2
DG(36:3e)	C39 H72 O4	2.22	604.5444	605.55168	13.876	1
DG(36:2e)	C39 H74 O4	0.99	606.55931	607.56659	22.2	2
DG(35:2e)	C38 H72 O4	0.85	592.54356	593.55084	18.404	2
DG(34:4e)	C37 H66 O4	1.75	574.49712	575.50439	20.046	2
DG(34:3e)	C37 H68 O4	1.06	576.51237	577.5195	17.977	1
DG(34:2e)	C37 H70 O4	0.58	578.52775	579.53502	19.319	2
DG(33:2e)	C36 H68 O4	0.72	564.51216	565.51944	13.079	2
DG(32:3e)	C35 H64 O4	0.52	548.48074	549.48802	11.182	2
DG(32:2e)	C35 H66 O4	0.98	550.49665	551.50393	18.343	2
DG(31:2e)	C34 H64 O4	0.12	536.48053	537.48835	11.557	2
DG(30:2e)	C33 H62 O4	1.05	522.46536	523.47264	10.974	2
DG(28:7e)	C31 H48 O4	-0.73	484.35491	485.36218	10.94	1
DG(28:5)	C31 H50 O5	-3.34	502.36415	503.37115	8.219	2
DG(26:4e)	C29 H50 O4	0.38	462.37108	463.37836	8.003	2
DG(25:2)	C28 H50 O5	1.39	466.36647	467.37375	8.508	2
DG(23:3e)	C26 H46 O4	-3.4	422.33817	423.34524	8.325	2
DG(23:2)	C26 H46 O5	-4.14	438.33271	439.33998	9.174	2

DG(21:2e)	C24 H44 O4	1.93	396.32472	397.332	12.539	2
DG(21:2)	C24 H42 O5	-4.26	410.30148	411.30872	7.407	2
DG(20:3e)	C23 H40 O4	-5.41	380.2906	381.29791	8.509	2
DG(20:1_18:2)	C41 H74 O5	-2.92	646.55174	647.55893	17.821	2
DG(19:3e)	C22 H38 O4	-5	366.27518	367.28268	7.523	2
DG(18:4_18:2)	C39 H64 O5	1.11	612.47605	613.48333	10.537	2
DG(18:4_18:1)	C39 H66 O5	1.46	614.49192	615.49921	11.515	2
DG(18:4_16:0)	C37 H64 O5	1.1	588.47602	589.4833	11.544	2
DG(18:3_18:2)	C39 H66 O5	1.88	614.49218	632.52601	15.006	3
DG(18:2_18:2)	C39 H68 O5	-0.91	616.50611	617.51223	18.354	2
DG(18:2_16:1)	C37 H66 O5	0.95	590.49158	591.49898	16.17	3
DG(18:2_16:0)	C37 H68 O5	0.75	592.50712	593.51431	14.433	2
DG(18:1_20:4)	C41 H70 O5	1.99	642.52361	660.55749	16.912	3
DG(18:1_20:3)	C41 H72 O5	1.71	644.53908	645.54692	17.339	3
DG(18:1_18:3)	C39 H68 O5	1.68	616.50771	617.51505	12.6	2
DG(18:1_18:2)	C39 H70 O5	0.79	618.52281	619.52994	14.682	2
DG(18:1_18:1)	C39 H72 O5	0.91	620.53854	621.54579	15.815	2
DG(18:1_18:0)	C39 H74 O5	1.16	622.55435	640.5882	23.172	3
DG(18:1_16:0)	C37 H70 O5	1.38	594.52314	595.53017	18.403	2
DG(18:1_14:0)	C35 H66 O5	1.01	566.4916	549.48834	14.001	2
DG(18:0_22:0)	C43 H84 O5	0.51	680.63222	698.66595	24.313	3
DG(18:0_20:0)	C41 H80 O5	0.7	652.60103	670.6348	19.012	3
DG(18:0_18:3)	C39 H70 O5	0.84	618.52285	619.53008	14.028	2
DG(18:0_18:1)	C39 H74 O5	1.11	622.55432	640.58813	19.092	2
DG(18:0_18:0)	C39 H76 O5	1.22	624.57004	642.60391	19.538	2
DG(18:0_17:0)	C38 H74 O5	0.87	610.55416	628.58807	22.582	3
DG(18:0_16:0)	C37 H72 O5	1.7	596.53899	597.54626	18.715	1
DG(17:0_18:2)	C38 H70 O5	1.1	606.52299	624.55682	17.476	3
DG(17:0_18:1)	C38 H72 O5	1.36	608.5388	626.57271	17.923	3
DG(17:0_16:0)	C36 H70 O5	1.35	582.52311	600.55693	17.854	3
DG(16:1_18:3)	C37 H64 O5	1.24	588.4761	606.51	14.911	3
DG(16:0_18:3)	C37 H66 O5	1.04	590.49164	591.49901	12.6	2
DG(16:0_16:1)	C35 H66 O5	0.54	566.49133	584.5252	16.765	3
DG(16:0_16:0)	C35 H68 O5	1.14	568.50732	586.54115	18.315	3
DG(14:0_18:2)	C35 H64 O5	1.65	564.47631	582.51013	15.564	3

Co(Q10)	C59 H90 O4	1.19	862.68494	880.71881	20.422	2
CmE(18:3)	C46 H76 O2	1.46	660.58549	661.59285	19.039	2
CmE(18:2)	C46 H78 O2	0.36	662.60042	680.63424	20.542	2
ChE(22:6)	C49 H76 O2	8.04	696.59014	697.59741	19.923	1
ChE(20:5)	C47 H74 O2	1.52	670.5699	671.57719	18.851	2
ChE(20:4)	C47 H76 O2	1.06	672.58524	673.59203	20.287	2
ChE(20:3)	C47 H78 O2	0.75	674.60069	675.60797	20.037	2
ChE(18:3)	C45 H74 O2	1.24	646.56969	647.57697	19.753	2
ChE(18:2)	C45 H76 O2	1.68	648.58562	649.59261	20.415	2
ChE(18:1)	C45 H78 O2	1.69	650.60128	651.60864	20.709	2
Cer(t42:0)	C42 H85 N O4	1.3	667.64873	668.65601	18.438	1
Cer(t20:0_26:0+O)	C46 H93 N O5	0.83	739.70599	740.71326	19.361	3
Cer(t20:0_26:0)	C46 H93 N O4	0.74	723.711	724.71827	20.114	1
Cer(t20:0_25:0+O)	C45 H91 N O5	0.02	725.68974	726.69702	19.346	1
Cer(t20:0_24:0+O)	C44 H89 N O5	-0.32	711.67385	712.682	19.477	3
Cer(t18:0_26:0)	C44 H89 N O4	1.33	695.68009	696.6864	19.762	1
Cer(t17:0_26:0+O)	C43 H87 N O5	0.05	697.65846	698.66554	19.042	1
Cer(m39:0+O)	C39 H79 N O3	0.88	609.60653	610.61381	20.589	2
Cer(m36:0)	C36 H73 N O2	1	551.56468	552.57196	18.117	1
Cer(m34:0)	C34 H69 N O2	0.64	523.53316	524.54056	17.444	2
Cer(m18:1_22:0)	C40 H79 N O2	1.22	605.61182	606.6191	18.312	2
Cer(m18:0_18:0)	C36 H73 N O2	1.02	551.56469	552.57202	22.455	2
Cer(m18:0_16:0)	C34 H69 N O2	0.79	523.53324	524.54053	22.003	2
Cer(d35:4)	C35 H63 N O3	2.04	545.48191	546.48919	13.999	2
Cer(d35:1)	C35 H69 N O3	0.62	551.52808	552.53534	22.138	2
Cer(d34:0)	C34 H69 N O3	2.29	539.52898	540.53625	16.635	2
Cer(d33:1)	C33 H65 N O3	0.35	523.49663	524.50391	21.723	2
Cer(d32:0)	C32 H65 N O3	0.96	511.49693	512.50421	20.902	2
Cer(d30:0)	C30 H61 N O3	1.62	483.46593	484.47321	12.933	2
Cer(d28:0)	C28 H57 N O3	2.77	455.43511	456.44238	11.263	2
Cer(d22:0_18:0)	C40 H81 N O3	1.72	623.62272	624.62998	19.341	2
Cer(d20:2_26:0+O)	C46 H89 N O4	1.14	719.67998	720.68726	19.537	2
Cer(d20:0_24:0)	C44 H89 N O3	1.29	679.68512	680.6924	20.044	2
Cer(d19:2_31:0+O)	C50 H97 N O4	1.09	775.74261	776.74991	19.342	2
Cer(d19:0_24:0)	C43 H87 N O3	1.7	665.66973	666.677	19.908	2

Cer(d18:2_28:0)	C46 H89 N O3	1.48	703.68529	704.69257	19.734	2
Cer(d18:2_26:0+O)	C44 H85 N O4	1.79	691.6491	692.65637	19.241	2
Cer(d18:2_24:0)	C42 H81 N O3	0.86	647.6222	648.62945	19.096	2
Cer(d18:2_23:0)	C41 H79 N O3	1.3	633.60682	634.61411	17.983	2
Cer(d18:2_22:0)	C40 H77 N O3	-0.68	619.58992	620.59835	17.684	2
Cer(d18:2_16:0)	C34 H65 N O3	1.37	535.49718	536.50442	13.747	2
Cer(d18:1_26:0)	C44 H87 N O3	1.95	677.66992	678.67716	19.946	2
Cer(d18:1_24:2)	C42 H79 N O3	0.84	645.60654	646.61407	17.727	2
Cer(d18:1_24:1)	C42 H81 N O3	-4.07	647.61901	648.63012	17.985	3
Cer(d18:1_24:0)	C42 H83 N O3	1.34	649.63816	650.64543	18.679	2
Cer(d18:1_23:0)	C41 H81 N O3	1.54	635.62263	636.62992	18.458	2
Cer(d18:1_22:0)	C40 H79 N O3	1.46	621.60691	622.61418	18.201	2
Cer(d18:1_20:0)	C38 H75 N O3	1.48	593.57557	594.58288	17.557	2
Cer(d18:1_19:0)	C37 H73 N O3	1.46	579.55989	580.56727	17.2	1
Cer(d18:1_18:0)	C36 H71 N O3	1.1	565.54402	566.55131	16.611	2
Cer(d18:1_16:0)	C34 H67 N O3	1.39	537.51284	538.52016	15.222	2
Cer(d18:0_16:0)	C34 H69 N O3	1.32	539.52846	540.53573	15.744	2
CarE(20:0)	C27 H53 N O4	0.44	455.39766	456.40494	8.16	1
CarE(16:1)	C23 H43 N O4	1.33	397.31974	398.32701	5.317	2
CarE(14:1)	C21 H39 N O4	2.08	369.28868	370.29595	3.231	3
CarE(14:0+O)	C21 H41 N O5	2.17	387.29931	388.30644	3.099	2
BisMePA(26:2e)	C31 H59 O7 P	5.53	574.40301	597.39185	11.136	1
BisMeLPA(12:0e)	C17 H37 O6 P	7.88	368.23568	369.24295	5.237	2
BisMeLPA(10:0e)	C15 H33 O6 P	1.67	340.20204	341.20932	3.358	2
AcHexSiE(16:0)	C51 H90 O7	-0.08	814.66859	815.67587	18.151	1
AcHexCmE(18:3)	C52 H86 O7	-3.02	822.63487	823.64215	17.61	1
AcHexCmE(18:1)	C52 H90 O7	-0.27	826.66843	844.7026	18.378	1
AcHexChE(18:2)	C51 H86 O7	-2.76	810.63512	811.6424	18.491	1
AcHexChE(17:1)	C50 H86 O7	-0.73	798.63677	799.64304	17.936	2
AcHexChE(16:1)	C49 H84 O7	0.58	784.62216	785.62872	17.845	2
AcHexChE(15:0)	C48 H84 O7	-1.61	772.62046	790.65631	17.676	2
AcHexChE(14:1)	C47 H80 O7	9.08	756.59728	757.60455	16.737	1
AcHexChE(0:0)	C33 H56 O6	0.87	548.40817	549.41544	9.38	1
AcCa(8:0)	C15 H29 N O4	6.26	287.21146	288.21731	1.43	2
AcCa(24:0)	C31 H61 N O4	1.56	511.46086	512.46813	11.275	2

AcCa(20:4)	C27 H45 N O4	1.4	447.33548	448.34276	4.054	2
AcCa(20:1)	C27 H51 N O4	0.75	453.38215	454.38941	6.089	2
AcCa(18:3)	C25 H43 N O4	1.27	421.31974	422.32702	3.615	2
AcCa(18:2)	C25 H45 N O4	2.08	423.33574	424.34302	4.134	2
AcCa(18:1)	C25 H47 N O4	1.53	425.35116	426.35844	5.023	2
AcCa(18:0)	C25 H49 N O4	1.6	427.36684	428.37412	6.286	3
AcCa(16:0)	C23 H45 N O4	1.78	399.33557	400.34285	4.73	3
AcCa(14:3)	C21 H35 N O4	1.37	365.25711	366.26443	2.46	2
AcCa(12:3)	C19 H31 N O4	0.19	337.22537	355.25931	1.528	1
AcCa(12:1)	C19 H35 N O4	1.03	341.25696	342.26438	2.203	2
AcCa(12:0)	C19 H37 N O4	2.28	343.27304	344.2803	2.583	2
AcCa(10:2)	C17 H29 N O4	1.25	311.21005	312.2173	1.522	2
AcCa(10:1)	C17 H31 N O4	1.55	313.22579	314.23307	1.623	2
AcCa(10:0)	C17 H33 N O4	0.8	315.24121	316.24845	1.691	2
			522.35423	523.36151	7.768	1

d via Orbitrap-IDX

Reference Ion	Log2 Fold Change: (Bb_pos_12022024_Newanalysis) / (CM_pos_12022024_Newanalysis)
[M+H]+1	-0.59
[M+H]+1	1.04
[M+H]+1	0.34
[M+H-H2O]+1	-5.26
[M+H]+1	0.91
[M+H]+1	-0.12
[M+H]+1	0.78
[M+H]+1	-2.12
[M+H]+1	0.36
[M+H]+1	-0.82
[M+H]+1	-1.02
[M+H+MeOH]+1	0.32
[M+H]+1	-1.95
[M+H]+1	-1.38
[M+H]+1	0.13
[M+H]+1	1.11
[M+H]+1	0.45
[M+H]+1	-4.71
[M+H]+1	-1.46
[M+NH4]+1	-1.72
[M+H]+1	0.12
[M+H]+1	-4.28
[M+H]+1	-4.08
[M+H]+1	-3.47
[M+Na]+1	-5.9
[M+H]+1	-1.42
[M+H]+1	0.17
[M+H]+1	0.84
[M+H]+1	0.64
[M+H]+1	-0.79
[M+H]+1	0.44
[M+H]+1	6.52
[M+H]+1	1.52

[M+H]+1	0.73
[M+H]+1	-5.16
[M+H]+1	1.97
[M+H]+1	-2.79
[M+H]+1	6.58
[M+H]+1	6.53
[M+H]+1	-6.08
[M+H]+1	0.21
[M+H]+1	0.42
[M+H]+1	-3.45
[M+H]+1	1.03
[M+H]+1	0.46
[M+NH4]+1	-4.22
[M+NH4]+1	-4.67
[M+NH4]+1	-4.54
[M+NH4]+1	-4.11
[M+NH4]+1	-2.78
[M+NH4]+1	-4.74
[M+NH4]+1	-3.77
[M+NH4]+1	-5.29
[M+NH4]+1	-2.74
[M+NH4]+1	1.18
[M+H]+1	-1.13
[M+H]+1	1.28
[M+H]+1	1.7
[M+H]+1	-0.61
[M+H]+1	1.39
[M+H]+1	-0.33
[M+H]+1	0.7
[M+H]+1	1.22
[M+H]+1	-5.99
[M+H]+1	-3.41
[M+H]+1	0.07
[M+H]+1	-5.06
[M+NH4]+1	-2.28

[M+H]+1	-0.42
[M+H]+1	-1.05
[M+H]+1	-4.8
[M+H]+1	0.55
[M+H]+1	-2.72
[M+NH4]+1	-3.8
[M+DMSO+H]+1	-0.36
[M+H]+1	0.81
[M+H]+1	-5.49
[M+H]+1	0.28
[M+NH4]+1	-4.7
[M+H]+1	-3.45
[M+H]+1	-4.27
[M+H]+1	-4.58
[M+H]+1	0.26
[M+H]+1	-2.96
[M+H]+1	-1.39
[M+H-H2O]+1	-2.48
[M+H]+1	0.23
[M+H]+1	-6.3
[M+H]+1	-2.36
[M+H]+1	-1.18
[M+NH4]+1	-5.34
[M+NH4]+1	-4.31
[M+NH4]+1	-2.39
[M+NH4]+1	-2.64
[M+H]+1	-2.49
[M+H]+1	-2.06
[M+H]+1	-4.98
[M+H]+1	-3.74
[M+H]+1	-0.38
[M+H]+1	-1.95
[M+H]+1	-2.05
[M+H]+1	-1.17
[M+H]+1	-2.49

[M+H]+1	-0.26
[M+H]+1	-2.29
[M+H]+1	0.76
[M+NH4]+1	-2.56
[M+H]+1	-0.45
[M+H]+1	0.87
[M+NH4]+1	-0.51
[M+H]+1	-2
[M+H]+1	-0.68
[M+H]+1	-3.86
[M+DMSO+H]+1	-2.73
[M+H]+1	0.52
[M+H]+1	-2.05
[M+H]+1	-0.91
[M+H]+1	-6.43
[M+H]+1	-4.07
[M+H]+1	-0.02
[M+H]+1	0.76
[M+H+MeOH]+1	-0.09
[M+NH4]+1	0.18
[M+NH4]+1	0.8
[M+H]+1	-0.45
[M+H]+1	-0.61
[M+H]+1	-2.65
[M+H]+1	-0.46
[M+H]+1	0.04
[M+H]+1	0.55
[M+H]+1	0.67
[M+H]+1	-1.84
[M+NH4]+1	-4.05
[M+H]+1	-1.42
[M+H]+1	-1.27
[M+H]+1	2.29
[M+H]+1	-2.4
[M+H]+1	-0.14

[M+H]+1	0
[M+H]+1	0.62
[M+H]+1	0.99
[M+H]+1	-0.4
[M+H]+1	0.74
[M+H]+1	0.84
[M+H]+1	0.5
[M+H]+1	0.11
[M+ACN+H]+1	1.87
[M+H]+1	0.14
[M+H]+1	0.99
[M+H]+1	0.74
[M+H]+1	-2.11
[M+H]+1	1.16
[M+H]+1	-0.4
[M+H]+1	-3.12
[M+ACN+H]+1	2.17
[M+H]+1	-0.23
[M+H]+1	1.43
[M+H]+1	0.43
[M+H]+1	-0.79
[M+H]+1	-0.96
[M+H]+1	-0.67
[M+H]+1	-2.76
[M+H]+1	0.18
[M+H]+1	-4.55
[M+H]+1	1.04
[M+H]+1	-1.93
[M+H]+1	-0.36
[M+H]+1	0.55
[M+H]+1	-1.57
[M+H]+1	-3.47
[M+H]+1	1.04
[M+H]+1	-1.45
[M+H]+1	-1.88

[M+H]+1	2.24
[M+H]+1	0.79
[M+H]+1	-4.43
[M+H]+1	-1.74
[M+H]+1	-3.76
[M+H]+1	-1.45
[M+H]+1	-3.94
[M+H]+1	-0.42
[M+H]+1	1.38
[M+H]+1	-0.3
[M+H]+1	1.93
[M+H]+1	0.44
[M+H]+1	-3.1
[M+H]+1	-2.37
[M+H]+1	1.1
[M+H]+1	-0.6
[M+H]+1	0.05
[M+H]+1	-2.15
[M+H]+1	-3.7
[M+H]+1	-1.29
[M+H]+1	-1.14
[M+H]+1	-1.52
[M+H]+1	0.87
[M+H]+1	0.25
[M+H]+1	0.77
[M+H]+1	0.01
[M+H]+1	-1.79
[M+H]+1	-2.33
[M+H]+1	-4.37
[M+H]+1	-4.14
[M+H]+1	-4.21
[M+H]+1	-3.9
[M+H]+1	-1.95
[M+H]+1	-1.21
[M+H]+1	-2.96

[M+H]+1	-4.11
[M+NH4]+1	-0.18
[M+NH4]+1	-0.56
[M+NH4]+1	-0.42
[M+H]+1	0.77
[M+NH4]+1	-0.05
[M+NH4]+1	-0.75
[M+NH4]+1	-0.06
[M+H]+1	0.29
[M+NH4]+1	0.8
[M+NH4]+1	0.66
[M+NH4]+1	-0.9
[M+NH4]+1	-1.79
[M+NH4]+1	-1.48
[M+H]+1	2.37
[M+H]+1	2.58
[M+H]+1	-3.71
[M+H]+1	1.46
[M+H]+1	-2.12
[M+H]+1	0.25
[M+H]+1	-2.39
[M+H]+1	0.75
[M+H]+1	-1.32
[M+H]+1	-0.77
[M+H]+1	-1.63
[M+H]+1	-1.9
[M+H]+1	0.07
[M+ACN+Na]+1	-0.09
[M+H]+1	1.01
[M+H]+1	-0.19
[M+H]+1	1.2
[M+H+MeOH]+1	0.27
[M+H]+1	0.73
[M+H]+1	0.06
[M+H]+1	-2.43

[M+H]+1	1.27
[M+H]+1	0.32
[M+H]+1	0.18
[M+H]+1	-1.04
[M+H]+1	-0.61
[M+H]+1	-0.75
[M+H]+1	-0.69
[M+H]+1	0.15
[M+H]+1	-0.48
[M+H]+1	-2.44
[M+H]+1	1.85
[M+H]+1	0.84
[M+H]+1	1.07
[M+H]+1	-1.03
[M+H]+1	4.44
[M+H]+1	2.88
[M+H]+1	2.06
[M+H]+1	3.1
[M+H]+1	-0.2
[M+H]+1	-5.58
[M+H]+1	-0.69
[M+H]+1	3.38
[M+H]+1	1.2
[M+H]+1	2.04
[M+H]+1	0.56
[M+H]+1	0.13
[M+H]+1	-1.16
[M+H]+1	1.07
[M+H]+1	1.13
[M+H]+1	0.39
[M+H]+1	-1.32
[M+H]+1	-3.48
[M+H]+1	1.25
[M+H]+1	-1.6
[M+H]+1	-2.8

[M+H]+1	-0.04
[M+H]+1	-3.17
[M+H]+1	-1.01
[M+H]+1	1.38
[M+H]+1	0.07
[M+H]+1	-1.46
[M+H]+1	-2.64
[M+H]+1	0.09
[M+H]+1	-0.67
[M+H]+1	-0.55
[M+H]+1	1.45
[M+H]+1	0.53
[M+H]+1	0.53
[M+H]+1	0.3
[M+H]+1	-1.06
[M+H]+1	1.01
[M+H]+1	-2.43
[M+H]+1	0.65
[M+H]+1	-2.41
[M+H]+1	-0.03
[M+H]+1	-5.37
[M+H]+1	2.52
[M+H]+1	-0.38
[M+H]+1	-1.23
[M+H]+1	0.29
[M+H]+1	0.15
[M+H]+1	-0.38
[M+H]+1	-1.52
[M+H]+1	-2.65
[M+H]+1	-1.84
[M+H]+1	-1.79
[M+H]+1	-2.97
[M+H]+1	3.13
[M+H]+1	-2.09
[M+H]+1	-0.91

[M+H]+1	-1.08
[M+H]+1	1.73
[M+H]+1	-2.27
[M+H]+1	-1.16
[M+H]+1	0.52
[M+H]+1	4.06
[M+H]+1	-2.83
[M+H]+1	-2.8
[M+H]+1	0.44
[M+H]+1	-1.97
[M+H]+1	-0.75
[M+H]+1	1.16
[M+H]+1	-2.05
[M+H]+1	-2.31
[M+H]+1	1.85
[M+H]+1	1.02
[M+H]+1	0.09
[M+H]+1	-3.26
[M+H]+1	-3.76
[M+H]+1	2.33
[M+H]+1	-1.84
[M+H]+1	2.94
[M+H]+1	-2.2
[M+H]+1	-2.67
[M+H]+1	-0.95
[M+H]+1	2.02
[M+H]+1	2.19
[M+H]+1	3.95
[M+H]+1	-2.87
[M+H]+1	7.19
[M+H]+1	8.35
[M+H]+1	-1.66
[M+H]+1	3.11
[M+H]+1	6.32
[M+H]+1	3.31

[M+H]+1	2.91
[M+H]+1	-0.18
[M+H]+1	3.93
[M+H]+1	4.2
[M+H]+1	-0.03
[M+H]+1	0.27
[M+H]+1	0.64
[M+H]+1	-0.95
[M+H]+1	-0.21
[M+H]+1	-1.37
[M+H]+1	2.39
[M+H]+1	-0.53
[M+H]+1	0.69
[M+H]+1	1.5
[M+H]+1	0.16
[M+H]+1	-2.31
[M+H]+1	1.34
[M+H]+1	0.39
[M+H]+1	-2.59
[M+H]+1	0.78
[M+H]+1	-3.14
[M+H]+1	-2.08
[M+H]+1	-1.08
[M+H]+1	-1.62
[M+H]+1	-2.84
[M+H]+1	0.85
[M+H]+1	-1.7
[M+H]+1	-1.72
[M+H]+1	-1.59
[M+H]+1	-1.85
[M+H]+1	-0.42
[M+H]+1	-1.14
[M+H]+1	-0.7
[M+H]+1	-4.07
[M+H]+1	0.46

[M+H]+1	0.67
[M+H]+1	-0.39
[M+H]+1	1
[M+H]+1	0.39
[M+H]+1	-0.88
[M+H]+1	-0.07
[M+H]+1	-0.99
[M+H]+1	-0.69
[M+H]+1	-1.31
[M+H]+1	1.62
[M+H]+1	-0.07
[M+H]+1	-0.12
[M+H]+1	0.29
[M+H]+1	-3.71
[M+H]+1	-4.2
[M+H]+1	-1.87
[M+H]+1	-2.57
[M+H]+1	-3.78
[M+H]+1	-4.54
[M+H]+1	-2.79
[M+H]+1	1.27
[M+H-H2O]+1	-1.32
[M+H]+1	-0.55
[M+H]+1	0.27
[M+H]+1	-1.77
[M+H]+1	2.56
[M+H]+1	1.5
[M+H]+1	-3.14
[M+H]+1	0.37
[M+H]+1	-2.65
[M+H]+1	-4.5
[M+H]+1	4
[M+H]+1	-2.45
[M+H]+1	-3.83
[M+H]+1	-2.41

[M+H]+1	-2.23
[M+H]+1	-2.42
[M+H]+1	-1.1
[M+H]+1	-6.14
[M+H]+1	-0.09
[M+H]+1	1.8
[M+H]+1	2.89
[M+H]+1	0.32
[M+H]+1	0.05
[M+H]+1	0.26
[M+H]+1	0.41
[M+H]+1	-2.77
[M+H]+1	-4.4
[M+H]+1	-2.54
[M+H]+1	1.02
[M+H]+1	-0.32
[M+H]+1	2.62
[M+H]+1	0.71
[M+H]+1	-2.81
[M+H]+1	-4.9
[M+H]+1	-1.2
[M+H]+1	-5.71
[M+H]+1	-2.39
[M+H]+1	-3.89
[M+H]+1	0.09
[M+H]+1	-2.51
[M+H]+1	0.38
[M+H]+1	-3.6
[M+H]+1	-2.09
[M+H]+1	1.87
[M+H]+1	1.13
[M+H]+1	0.63
[M+H]+1	-1.64
[M+H]+1	-4.71
[M+H]+1	-3.87

[M+H]+1	-4.1
[M+H]+1	-2.01
[M+H]+1	0.41
[M+H]+1	-2.08
[M+Na]+1	0.64
[M+H]+1	0.71
[M+H]+1	-4.21
[M+H]+1	-1.77
[M+H]+1	1.11
[M+H]+1	1.75
[M+H]+1	-0.98
[M+H]+1	3.13
[M+H]+1	5.77
[M+H]+1	6.43
[M+H]+1	6.98
[M+H]+1	6.62
[M+H]+1	8.23
[M+H]+1	7.37
[M+H]+1	-1.5
[M+H]+1	-0.36
[M+H]+1	0.97
[M+H]+1	0.19
[M+H]+1	1.98
[M+H]+1	1.19
[M+H]+1	4
[M+H]+1	6.17
[M+H]+1	7.16
[M+H]+1	-0.21
[M+H]+1	-0.68
[M+H]+1	-0.01
[M+H]+1	0.1
[M+H]+1	4.42
[M+H]+1	4.5
[M+H]+1	7.16
[M+H]+1	2.81

[M+H]+1	0.2
[M+H]+1	2.11
[M+H]+1	-3.28
[M+H]+1	-2.11
[2M+Na]+1	3.29
[M+H]+1	0.57
[M+H]+1	0.46
[M+H]+1	0.59
[M+H]+1	0.57
[M+H]+1	-1.45
[M+H]+1	0.24
[M+H]+1	-1.38
[M+H]+1	0.05
[M+H]+1	0.25
[M+H]+1	-0.4
[M+H]+1	-2.68
[M+H]+1	3.27
[M+H]+1	0.89
[M+H]+1	3.85
[M+H]+1	-0.29
[M+H]+1	-0.95
[M+H]+1	0.82
[M+H]+1	0.45
[M+H]+1	0.42
[M+H]+1	-1.4
[M+H]+1	-0.59
[M+H]+1	2.48
[M+H]+1	0.47
[M+H]+1	-0.12
[M+H]+1	0.46
[M+H]+1	-2.27
[M+H]+1	-4.19
[M+H]+1	-0.14
[M+H]+1	1.37
[M+H]+1	-1.31

[M+H]+1	0.17
[M+H]+1	0.46
[M+H]+1	-0.7
[M+H]+1	0.64
[M+H]+1	-1.3
[M+H]+1	-0.14
[M+H]+1	-2.98
[M+H]+1	0.58
[M+H]+1	-5.72
[M+H]+1	-1.55
[M+H]+1	-0.13
[M+H]+1	-0.53
[M+H]+1	0.89
[M+H]+1	0.72
[M+H]+1	-1.01
[M+H]+1	-1.3
[M+H]+1	-2.1
[M+H]+1	-3.14
[M+H]+1	-1.11
[M+H]+1	1.85
[M+H]+1	-5.92
[M+H]+1	-2.13
[M+H]+1	-5.33
[M+H]+1	1.72
[M+H]+1	-0.15
[M+H]+1	0.09
[M+H]+1	-5.31
[M+H]+1	-0.13
[M+H]+1	-0.02
[M+H]+1	-5.45
[M+H]+1	0.91
[M+H]+1	-1.04
[M+H]+1	-0.62
[M+NH4]+1	4.44
[M+H]+1	0.52

[M+H]+1	6.45
[M+NH4]+1	0.09
[M+NH4]+1	5.47
[M+H]+1	1.91
[M+NH4]+1	1.29
[M+NH4]+1	0.75
[M+H]+1	-0.46
[M+H]+1	-1.78
[M+H]+1	-0.58
[M+H]+1	1.3
[M+H]+1	0.04
[M+H]+1	-0.95
[M+H]+1	1.28
[M+H]+1	-1.78
[M+H]+1	-1.25
[M+H]+1	-3.92
[M+H]+1	-0.65
[M+H]+1	2.58
[M+H]+1	1.37
[M+H]+1	0.57
[M+H]+1	1.68
[M+H]+1	-4.88
[M+H]+1	0.99
[M+H]+1	0.01
[M+H]+1	3.28
[M+H]+1	3.74
[M+H]+1	2.01
[M+H]+1	0.51
[M+H]+1	2.28
[M+H]+1	-0.25
[M+H]+1	-0.58
[M+H]+1	-0.58
[M+H]+1	0.67
[M+H]+1	-0.13
[M+H]+1	0.59

[M+H]+1	0.93
[M+H]+1	0.34
[M+H]+1	0.86
[M+H]+1	-0.75
[M+H]+1	-0.46
[M+H]+1	1.03
[M+H]+1	-4.39
[M+H]+1	-1.95
[M+NH4]+1	-2.29
[M+H]+1	1.41
[M+H]+1	-0.21
[M+H]+1	5.39
[M+NH4]+1	-1.36
[M+H]+1	0.42
[M+H]+1	-4
[M+H]+1	1.9
[M+H]+1	4.01
[M+NH4]+1	0.39
[M+H]+1	2.12
[M+H-H2O]+1	3.68
[M+NH4]+1	0.71
[M+NH4]+1	1.28
[M+H]+1	0.14
[M+NH4]+1	-1.11
[M+NH4]+1	0.72
[M+NH4]+1	0.78
[M+H]+1	1.42
[M+NH4]+1	-1.67
[M+NH4]+1	1.07
[M+NH4]+1	0.03
[M+NH4]+1	-2.2
[M+H]+1	-0.64
[M+NH4]+1	-0.38
[M+NH4]+1	-0.06
[M+NH4]+1	0.51

[M+NH4]+1	-0.97
[M+H]+1	-4.95
[M+NH4]+1	-5.63
[M+H]+1	-2.37
[M+H]+1	-5.24
[M+H]+1	-2.98
[M+H]+1	-2.85
[M+H]+1	-2.71
[M+H]+1	-5.15
[M+H]+1	-2.68
[M+H]+1	-0.88
[M+H]+1	0.02
[M+H]+1	-2.91
[M+H]+1	-0.15
[M+H]+1	0.88
[M+H]+1	-0.05
[M+H]+1	0
[M+H]+1	4.11
[M+H]+1	0.77
[M+H]+1	1.21
[M+H]+1	0.16
[M+H]+1	0.66
[M+H]+1	0.36
[M+H]+1	0.23
[M+H]+1	0.37
[M+H]+1	0.37
[M+H]+1	0.62
[M+H]+1	0.9
[M+H]+1	-0.37
[M+H]+1	-0.37
[M+H]+1	-0.12
[M+H]+1	-0.17
[M+H]+1	-2.65
[M+H]+1	-1.24
[M+H]+1	-2.73

[M+H]+1	-0.39
[M+H]+1	0.26
[M+H]+1	0.76
[M+H]+1	0.2
[M+H]+1	-0.93
[M+H]+1	0.48
[M+H]+1	-0.91
[M+H]+1	-3.8
[M+H]+1	0.52
[M+H]+1	-5.29
[M+H]+1	-5.15
[M+H]+1	-5.18
[M+H]+1	-4.57
[M+H]+1	-1.2
[M+H]+1	-0.32
[M+H]+1	-1.24
[M+H]+1	0.12
[M+H]+1	-1.01
[M+H]+1	0.63
[M+H]+1	0.48
[M+H]+1	2.17
[M+Na]+1	0.83
[M+H]+1	2.11
[M+H]+1	-0.82
[M+H]+1	0.47
[M+H]+1	-0.66
[M+NH4]+1	6.24
[M+H]+1	2.68
[M+H]+1	0.05
[M+H]+1	-2.45
[M+NH4]+1	-1.17
[M+H]+1	-1.84
[M+H]+1	1.03
[M+H]+1	0.58
[M+H]+1	-2.05

[M+H]+1	0.67
[M+H]+1	0.89
[M+H]+1	-2.19
[M+H]+1	-1.19
[M+H]+1	-0.91
[M+H]+1	-3.63
[M+H]+1	-3.01
[M+H]+1	-0.03
[M+NH4]+1	-2.22
[M+H]+1	-1.3
[M+H]+1	0.11
[M+H]+1	1.32
[M+H]+1	1.9
[M+H]+1	1.95
[M+H]+1	-4.54

Log2 Fold Change: (Bb_pos_12022024_Newanalysis) / (Rabbitserum_12022024_Newanalysis)
-2.8
0.41
-4.78
-8.31
-0.98
0.26
2.67
-3.26
-1.6
0.89
-0.25
1.34
-4.59
-1.04
1.26
3.28
0.09
-6.81
-4.43
-5.36
0.12
-6.53
-5.44
-6.96
-7.64
-0.21
0.11
1.18
1.54
0.4
1.55
5.81
2.9

	1.85
	-6.15
	-3.96
	-6.24
	6.51
	5.6
	-7.48
	0.34
	-2.09
	-3.49
	-2.22
	1
	-5.98
	-6.69
	-6.27
	-5.38
	-3.58
	-5.81
	-4.45
	-6.91
	-4.46
	-0.05
	-1.86
	-1.7
	1.93
	-2.73
	-0.55
	-3.35
	0
	1.28
	-7.23
	-4.22
	-0.31
	-3.94
	-5.04

	-4.6
	-1.82
	-2.84
	0.17
	-4.01
	-6.51
	-3.6
	0.67
	-7.2
	-2.84
	-6.84
	-7.17
	-6.17
	-3.12
	0.86
	-3.96
	-7.56
	-2.37
	1.14
	-6.46
	-2.84
	-2.14
	-6.4
	-6.27
	-4.43
	-4.48
	-4.52
	-4.62
	-6
	-5.44
	-0.76
	-1.92
	-5.91
	1.01
	-3.44

	0.4
	-4.3
	1.51
	-3.52
	-1.01
	-0.63
	1.13
	-2.69
	-3.7
	-2.5
	-4.2
	0.57
	-3.18
	-0.99
	-7.4
	-5.55
	-1.64
	0.15
	-6.46
	-1.77
	1.69
	-1.8
	-1.77
	-3.54
	-1.07
	-2.77
	0.96
	-0.56
	-3.51
	-7.09
	-3.32
	-0.54
	1.98
	-3.67
	-1.62

	-0.34
	-1.27
	1.53
	-0.57
	1.47
	1.41
	1.04
	-0.19
	2.9
	-1.01
	0.97
	-4.09
	-7.13
	-1.53
	-4.55
	-5.71
	2.67
	-1.95
	3.04
	-1.64
	-3.62
	-4.25
	-5.08
	-5.24
	-1.53
	-7.37
	-0.4
	-2.62
	-3.89
	-1.02
	-5.11
	-4.16
	-1.21
	-2.96
	-5.72

	0.56
	-4.11
	-5.93
	-6.48
	-5.51
	-4.97
	-5.95
	-3.63
	2.1
	-7.2
	-1.21
	0.15
	-7.17
	-4.03
	2.4
	-4.06
	-3.53
	-3.63
	-6.39
	-4.25
	-3.99
	-3.75
	0.08
	0.14
	1.39
	-0.93
	-3.99
	-0.58
	-5.75
	-7.85
	-5.78
	-5.9
	-5.55
	-5.09
	-6.23

-4.98
-2.08
-1.65
-0.29
1.21
-0.47
-2.39
-2.82
-1.23
-0.71
1.33
-0.63
-2.77
-0.94
1.94
2.4
-3.09
-3.91
-3.04
-0.19
-4.54
-2.32
1.08
-4.58
-5
-6.09
-0.31
-0.52
1.59
-0.85
-1.21
0.06
-1.4
-1.17
-4.99

	0.1
	-1.9
	0.52
	-1.06
	-1.98
	-3.26
	-3.28
	-0.75
	-1.86
	-6.66
	0.16
	-1.06
	-2.1
	-3.33
	1.84
	-0.58
	0.41
	0.61
	0.6
	-7.65
	-3.37
	0.07
	-0.85
	0.14
	-0.68
	-0.13
	-2.45
	-0.22
	-1.85
	-2.6
	-2.79
	-5.59
	2.08
	-3.2
	-5.88

	-3.51
	-6.42
	-2.82
	-3.86
	-3.18
	-3.03
	-6.04
	-0.37
	-1.43
	-4.59
	0.27
	-2.74
	0.87
	-1.15
	-0.87
	0.11
	-7.02
	-2.13
	-2.21
	-2.36
	-6.19
	-2
	-1.07
	-6.29
	-3.97
	-3.74
	-3.34
	-4.11
	-1.15
	-4.39
	-4.26
	-5.53
	1.23
	-3.65
	-1.71

	-4.82
	0.86
	-3.85
	-4.61
	2.66
	2.32
	-4.89
	-6.98
	-0.48
	-5.18
	-2.05
	2.74
	-2.74
	-2.84
	1.5
	-1.25
	-2.46
	-5.62
	-6.27
	-2.49
	-1.69
	-0.22
	-2.53
	-6.5
	-4.18
	2.04
	3.14
	0.96
	-1.56
	3.6
	3.56
	-4.8
	-0.49
	4.3
	1.87

	1.28
	-1.09
	5.85
	4.55
	-4.26
	-0.58
	-2.75
	-5.24
	-2.93
	-3.64
	-0.91
	-3.76
	-3.44
	1.52
	-0.6
	-3.45
	-0.34
	-2.55
	-4.89
	-2.64
	-5.92
	-6.08
	0.6
	-4.47
	-6.04
	0.71
	-4.12
	-4.16
	-4.89
	-5.38
	-3.21
	-4.27
	-3.52
	-4.69
	-0.78

-2.16
-1.48
-1.5
-2.34
-2.49
-3.8
-3.39
-4.46
-4.32
0.46
-3.62
-5.83
-3.68
-7.2
-7.34
-2.6
-4.17
-5.46
-7.12
-4.2
1.86
-2.34
-2.24
-4.16
-1.44
1.76
-1.47
-4.66
0.9
-4.59
-7.87
5.11
-5.32
-5.69
-4.82

	-5.32
	-4.56
	-3.91
	-7.77
	-2.47
	0.15
	2.06
	-1.26
	-4.1
	-2.82
	-1.41
	-5.64
	-7.89
	-2.96
	-2.56
	-2.99
	-0.22
	-0.71
	-4.42
	-7.69
	-2.84
	-9.35
	-4.82
	-5.21
	-2.28
	-4.04
	-3.99
	-6.98
	-2.52
	1.29
	-2.11
	-1.26
	-5.34
	-8.2
	-7.38

	-7.17
	-4.9
	-3.99
	-1.75
	-0.18
	-0.67
	-6.79
	-4.56
	-1.5
	-2.85
	-4.12
	0.99
	2.38
	5.16
	3.62
	5.84
	5.39
	6.72
	-2.35
	-2.97
	-1.69
	-3.3
	-1.59
	-2.58
	0.41
	3.33
	4.76
	-3.69
	-0.63
	-1.41
	-2.45
	2.27
	1.36
	6.79
	3.11

	0.82
	2.52
	-2.54
	-4.96
	0.28
	-4.95
	3.41
	0.08
	2.6
	2.14
	2.12
	-5.4
	-5.4
	-2.68
	-3.16
	-4.52
	0.32
	-2.05
	2.6
	-2.3
	-4.33
	-1.04
	-0.81
	0.11
	-3.71
	-5.32
	0.25
	-3.73
	-3.71
	-2.14
	-4.6
	-6.16
	-5.65
	1.71
	-5.32

	-4.13
	-2.62
	-4.85
	2.74
	-7.27
	-2.31
	-6.39
	-0.58
	-9.79
	-6.92
	-3.05
	-4.45
	-2.3
	2.71
	-5.11
	-2.08
	-5.15
	-7.88
	-5.94
	2.78
	-9.28
	-6.57
	-9.37
	-2.86
	-4.64
	-2.65
	-9.61
	-2.32
	-3.3
	-8.97
	-2.37
	-2.75
	1.09
	4.36
	0.83

	1.49
	-0.63
	0.5
	2.76
	4.11
	1.51
	-0.57
	-3.26
	-3.46
	0.9
	1.38
	-1.11
	4.42
	-4.89
	-3.05
	-5.69
	-0.74
	2.81
	0.41
	1.14
	2.99
	-6.2
	-0.97
	3.57
	2.06
	3.4
	3.56
	2.67
	3.24
	-1.28
	-0.21
	-2.91
	-2.15
	0.5
	-0.2

	3.32
	0.66
	1.92
	2.4
	0.67
	-1.18
	-7.42
	-4.71
	-5.58
	0.85
	-1.87
	2.67
	-4.97
	-1.1
	-7.1
	4.98
	2.15
	0.09
	1.98
	0.67
	1.13
	4.66
	-1.37
	0.41
	2.64
	1.26
	1.98
	-4.91
	-1.34
	-0.74
	-5.07
	-2.23
	-3.27
	1.86
	-2.59

	-2.06
	-4.92
	-7.51
	-3.84
	-6.14
	-3.66
	-3.74
	-6.07
	-6.41
	-3.83
	-2.15
	1.65
	-3.46
	-0.31
	2.82
	-0.92
	0.69
	5.2
	2.85
	3.03
	0.46
	0.96
	0.93
	-2.41
	0.82
	1.13
	0.91
	1.53
	-1.28
	0.24
	1.44
	0.45
	-3.54
	-4.17
	-3.84

	-1.5
	0.35
	0.16
	-3.22
	-4.12
	-3.34
	-2.49
	-6.48
	-1.53
	-7
	-6.92
	-6.99
	-7.09
	-3.65
	-3.06
	-5.04
	-3.15
	-0.12
	0.75
	-0.08
	2.79
	-0.74
	4.33
	-0.97
	-1.26
	-3.28
	2.62
	3.18
	-2.66
	-2.46
	-5.11
	-3.56
	1.44
	0.5
	-4.49

-0.78
1.59
-0.91
-0.94
-1.62
-6.52
-5
0.46
-1.47
-2.42
0.59
1.65
1.02
1.77
-11.28

Log2 Fold Change: (CM_pos_12022024_Newanalysis) / (Rabbitserum_12022024_Newanalysis)
-2.21
-0.62
-5.13
-3.06
-1.9
0.38
1.88
-1.14
-1.97
1.71
0.77
1.01
-2.64
0.34
1.13
2.17
-0.36
-2.11
-2.97
-3.64
0
-2.25
-1.36
-3.48
-1.75
1.21
-0.06
0.35
0.9
1.2
1.1
-0.72
1.38

	1.12
	-0.99
	-5.92
	-3.45
	-0.06
	-0.93
	-1.39
	0.13
	-2.51
	-0.04
	-3.25
	0.53
	-1.76
	-2.02
	-1.73
	-1.27
	-0.81
	-1.07
	-0.68
	-1.62
	-1.72
	-1.22
	-0.74
	-2.98
	0.23
	-2.12
	-1.94
	-3.02
	-0.7
	0.06
	-1.24
	-0.81
	-0.38
	1.12
	-2.77

	-4.18
	-0.78
	1.96
	-0.38
	-1.29
	-2.7
	-3.24
	-0.14
	-1.71
	-3.12
	-2.14
	-3.72
	-1.9
	1.46
	0.61
	-1
	-6.17
	0.12
	0.91
	-0.16
	-0.48
	-0.96
	-1.05
	-1.95
	-2.04
	-1.84
	-2.03
	-2.56
	-1.02
	-1.69
	-0.38
	0.03
	-3.86
	2.19
	-0.95

	0.66
	-2.01
	0.75
	-0.96
	-0.56
	-1.5
	1.64
	-0.69
	-3.01
	1.37
	-1.47
	0.05
	-1.13
	-0.08
	-0.97
	-1.48
	-1.62
	-0.61
	-6.37
	-1.95
	0.89
	-1.36
	-1.16
	-0.89
	-0.61
	-2.81
	0.4
	-1.23
	-1.67
	-3.04
	-1.9
	0.73
	-0.31
	-1.27
	-1.48

	-0.34
	-1.89
	0.54
	-0.17
	0.73
	0.57
	0.54
	-0.31
	1.03
	-1.16
	-0.02
	-4.83
	-5.02
	-2.68
	-4.15
	-2.6
	0.51
	-1.72
	1.61
	-2.07
	-2.82
	-3.29
	-4.41
	-2.48
	-1.72
	-2.82
	-1.43
	-0.69
	-3.53
	-1.57
	-3.54
	-0.69
	-2.25
	-1.51
	-3.84

	-1.68
	-4.9
	-1.51
	-4.74
	-1.76
	-3.51
	-2.01
	-3.21
	0.72
	-6.9
	-3.14
	-0.29
	-4.07
	-1.67
	1.29
	-3.46
	-3.58
	-1.48
	-2.7
	-2.96
	-2.85
	-2.23
	-0.8
	-0.11
	0.62
	-0.95
	-2.2
	1.75
	-1.38
	-3.71
	-1.57
	-2
	-3.6
	-3.88
	-3.26

-0.87
-1.9
-1.09
0.13
0.45
-0.43
-1.64
-2.76
-1.52
-1.5
0.68
0.27
-0.98
0.54
-0.43
-0.18
0.62
-5.37
-0.92
-0.44
-2.15
-3.07
2.4
-3.81
-3.38
-4.18
-0.38
-0.43
0.58
-0.67
-2.4
-0.2
-2.13
-1.23
-2.55

-1.17
-2.22
0.34
-0.02
-1.37
-2.51
-2.59
-0.91
-1.38
-4.22
-1.69
-1.9
-3.17
-2.3
-2.6
-3.46
-1.65
-2.48
0.79
-2.07
-2.69
-3.31
-2.05
-1.89
-1.24
-0.26
-1.3
-1.3
-2.98
-2.99
-1.48
-2.12
0.83
-1.59
-3.08

	-3.47
	-3.24
	-1.81
	-5.24
	-3.25
	-1.57
	-3.39
	-0.45
	-0.76
	-4.04
	-1.18
	-3.27
	0.34
	-1.45
	0.2
	-0.9
	-4.6
	-2.78
	0.21
	-2.33
	-0.82
	-4.52
	-0.69
	-5.07
	-4.26
	-3.89
	-2.97
	-2.59
	1.5
	-2.55
	-2.47
	-2.57
	-1.9
	-1.56
	-0.79

	-3.73
	-0.88
	-1.58
	-3.45
	2.15
	-1.74
	-2.06
	-4.19
	-0.92
	-3.21
	-1.3
	1.57
	-0.69
	-0.53
	-0.35
	-2.27
	-2.55
	-2.35
	-2.52
	-4.82
	0.15
	-3.16
	-0.32
	-3.83
	-3.23
	0.03
	0.95
	-3
	1.32
	-3.59
	-4.79
	-3.13
	-3.6
	-2.02
	-1.44

	-1.63
	-0.92
	1.92
	0.35
	-4.23
	-0.85
	-3.39
	-4.29
	-2.72
	-2.27
	-3.3
	-3.22
	-4.14
	0.02
	-0.76
	-1.14
	-1.68
	-2.95
	-2.3
	-3.41
	-2.78
	-4
	1.68
	-2.85
	-3.2
	-0.15
	-2.42
	-2.43
	-3.3
	-3.53
	-2.8
	-3.14
	-2.82
	-0.62
	-1.24

	-2.83
	-1.08
	-2.5
	-2.73
	-1.61
	-3.73
	-2.4
	-3.77
	-3.01
	-1.16
	-3.55
	-5.71
	-3.97
	-3.48
	-3.15
	-0.73
	-1.6
	-1.68
	-2.58
	-1.41
	0.58
	-1.02
	-1.7
	-4.43
	0.33
	-0.79
	-2.97
	-1.52
	0.52
	-1.94
	-3.37
	1.11
	-2.88
	-1.86
	-2.41

	-3.09
	-2.14
	-2.81
	-1.64
	-2.38
	-1.65
	-0.83
	-1.58
	-4.15
	-3.08
	-1.82
	-2.87
	-3.49
	-0.42
	-3.58
	-2.67
	-2.84
	-1.42
	-1.62
	-2.79
	-1.64
	-3.64
	-2.43
	-1.32
	-2.37
	-1.52
	-4.37
	-3.39
	-0.43
	-0.58
	-3.23
	-1.9
	-3.7
	-3.49
	-3.51

	-3.07
	-2.89
	-4.4
	0.32
	-0.82
	-1.38
	-2.58
	-2.79
	-2.61
	-4.6
	-3.15
	-2.14
	-3.39
	-1.28
	-3.36
	-0.78
	-2.83
	-0.65
	-0.85
	-2.61
	-2.66
	-3.5
	-3.57
	-3.77
	-3.59
	-2.84
	-2.4
	-3.48
	0.06
	-1.4
	-2.55
	-2.15
	-3.14
	-0.37
	0.3

	0.62
	0.41
	0.75
	-2.85
	-3.01
	-5.51
	2.95
	-0.51
	2.03
	3.59
	1.88
	-4.01
	-5.45
	-2.94
	-2.76
	-1.84
	-2.95
	-2.94
	-1.25
	-2.01
	-3.38
	-1.87
	-1.27
	-0.31
	-2.31
	-4.74
	-2.23
	-4.2
	-3.59
	-2.6
	-2.33
	-1.97
	-5.51
	0.34
	-4.01

	-4.31
	-3.08
	-4.15
	2.1
	-5.97
	-2.17
	-3.41
	-1.16
	-4.07
	-5.37
	-2.92
	-3.92
	-3.2
	1.99
	-4.09
	-0.77
	-3.05
	-4.75
	-4.83
	0.93
	-3.36
	-4.44
	-4.04
	-4.58
	-4.49
	-2.74
	-4.3
	-2.19
	-3.28
	-3.53
	-3.28
	-1.71
	1.71
	-0.09
	0.32

	-4.96
	-0.72
	-4.97
	0.84
	2.81
	0.77
	-0.12
	-1.48
	-2.88
	-0.4
	1.33
	-0.16
	3.14
	-3.11
	-1.8
	-1.77
	-0.09
	0.23
	-0.95
	0.57
	1.31
	-1.32
	-1.96
	3.56
	-1.21
	-0.34
	1.55
	2.17
	0.96
	-1.03
	0.36
	-2.33
	-2.82
	0.63
	-0.79

	2.39
	0.32
	1.06
	3.15
	1.13
	-2.21
	-3.03
	-2.76
	-3.29
	-0.57
	-1.67
	-2.73
	-3.61
	-1.51
	-3.1
	3.08
	-1.86
	-0.29
	-0.15
	-3.01
	0.43
	3.37
	-1.51
	1.52
	1.92
	0.48
	0.56
	-3.24
	-2.42
	-0.77
	-2.87
	-1.59
	-2.89
	1.92
	-3.11

	-1.09
	0.04
	-1.88
	-1.47
	-0.9
	-0.68
	-0.89
	-3.36
	-1.25
	-1.15
	-1.27
	1.64
	-0.55
	-0.16
	1.94
	-0.87
	0.7
	1.1
	2.08
	1.82
	0.3
	0.31
	0.57
	-2.64
	0.44
	0.76
	0.29
	0.63
	-0.9
	0.61
	1.56
	0.61
	-0.89
	-2.93
	-1.11

	-1.11
	0.09
	-0.6
	-3.41
	-3.19
	-3.82
	-1.58
	-2.68
	-2.05
	-1.71
	-1.77
	-1.81
	-2.51
	-2.44
	-2.74
	-3.81
	-3.27
	0.89
	0.13
	-0.55
	0.61
	-1.56
	2.22
	-0.15
	-1.73
	-2.63
	-3.63
	0.5
	-2.71
	-0.02
	-3.94
	-1.72
	0.41
	-0.08
	-2.43

	-1.45
	0.7
	1.28
	0.25
	-0.71
	-2.89
	-1.99
	0.49
	0.76
	-1.12
	0.48
	0.33
	-0.88
	-0.18
	-6.73

Log2 Fold Change: (Hs_pos_12022024_Newanalysis) / (Rabbitserum_12022024_Newanalysis)
2.44
-0.59
-1.4
-0.75
-1.29
0
-7.41
-1.19
1.27
-1.08
0.39
-4.93
-0.93
0.61
0.2
0.5
1.13
-3.92
-2.84
-0.85
-2.62
-4.27
-1.76
-3.85
-2.68
5.27
5.67
7.69
4.17
-0.48
-1.21
0.84
-0.3

-0.6
-4.14
-2.08
-1.64
1.35
-0.52
0.53
-0.91
-1.7
0.01
0.45
-0.79
-5.5
-6.52
-6.01
-6.74
-5.25
-7.32
-6.12
-8.28
-5.9
-2.87
-1.01
1.31
-1.47
0.24
0.56
-3.55
-0.88
1.63
-0.25
1.87
-1.64
-0.18
1.35

	2.18
	0.02
	0.83
	0.8
	-0.18
	2.14
	-1.94
	-2.44
	-2.86
	-2.49
	-1.83
	-1.44
	0.84
	1.9
	-0.55
	0.85
	1.86
	-0.08
	-0.87
	0.99
	-1.02
	0.19
	0.15
	-1.48
	-2.78
	-4.24
	-2.42
	2.27
	-0.44
	-2.01
	-2.3
	-1.5
	-2.4
	0.03
	0.38

0.98
-0.1
-1.06
0.19
0.28
-1.43
-1.53
-0.57
-3.12
-0.87
-0.36
-1.02
0.2
-1.34
-2.51
-0.79
-1.31
-0.56
-4.34
-1.1
3.74
-0.33
-0.6
-0.6
1.65
0.27
0.71
-2.16
0.61
-0.4
1
-0.89
0.12
-4.42
-0.5

	-0.54
	1.67
	-3.26
	2.71
	0.33
	-1.56
	0.25
	-2.88
	0.34
	-2.33
	0.98
	-2.02
	-0.35
	-1.61
	2.63
	1.6
	0.59
	2.83
	2.44
	2.24
	3.33
	2.87
	2.37
	1.85
	3.37
	2.73
	1.99
	-0.29
	3.16
	-0.16
	-0.51
	2.48
	1
	3.15
	-2.83

	-0.17
	1.01
	2.96
	-1.11
	-0.18
	2.07
	-1.03
	3.27
	1.39
	-0.45
	0.57
	2.81
	2.09
	-3.85
	5.51
	-3.92
	3.98
	4.06
	-1.59
	6.33
	5.48
	4.49
	4.85
	5.74
	7.46
	6.39
	4.51
	5.62
	3.74
	1.85
	2.49
	2.49
	3.29
	0.22
	-1.77

-3.71
4.49
2.5
5.87
5.25
5.14
1.67
1
1.95
1.16
5.64
7.22
4.38
2.35
-0.04
1.32
-4.73
-1.56
3.17
1.43
-0.7
-0.54
-1.33
0.44
0.04
-7.06
-1.64
2.61
2.52
1.73
1.18
2.79
4.15
2.07
-1.13

	-0.45
	2.32
	0.1
	0.63
	3.82
	3.72
	3.42
	1.87
	4.05
	2.51
	-0.21
	1.32
	4.33
	2.1
	1.62
	-0.27
	4.86
	3.77
	1.06
	1.18
	3.27
	0.6
	2.62
	0.84
	1.39
	-2.01
	-0.52
	1.7
	0.49
	4
	4.06
	3.77
	6.12
	1.15
	1.85

	0.91
	2.49
	6.92
	-1.75
	1.02
	1.98
	1.48
	3.21
	5.36
	5.84
	3.87
	4.16
	1.76
	5.74
	4.81
	-2.13
	2.41
	3.63
	7.39
	1.42
	-2.47
	0.85
	4.46
	0.36
	1.54
	1.07
	0.13
	-1.24
	-2.54
	-1.27
	3.01
	1.91
	-0.23
	1.33
	6.58

	-1.77
	-0.41
	1.96
	0.01
	-0.66
	5.07
	-0.33
	2.41
	0.53
	0.4
	0.62
	3.36
	0.04
	-0.55
	-0.43
	-1.46
	-1.23
	2.24
	0.96
	-2.53
	0.7
	0.98
	-0.6
	-2.84
	-2.11
	2.29
	-0.53
	-2.34
	0.05
	-5.16
	-0.98
	1.31
	-0.98
	1.9
	0.77

	1.77
	3.91
	-0.88
	-0.99
	0.82
	2.31
	2.82
	1.17
	2.77
	4
	-0.34
	-1.06
	0.52
	0.08
	3.49
	1.12
	2.48
	2.08
	-4.11
	0.74
	5.04
	0.13
	4.01
	1.21
	2.24
	-0.06
	2.55
	-2.21
	1.18
	0.05
	2.74
	-5.97
	1.54
	-0.97
	0.89

	0.98
	0.55
	-0.14
	0.22
	-4.46
	3.76
	2.48
	1.81
	2.01
	-1.45
	1.94
	-2.12
	0.61
	1.96
	-1.21
	4.4
	3.55
	1.86
	1.64
	1.16
	-0.06
	0.25
	1.36
	1.26
	-1.11
	2.47
	2.21
	-2.01
	6.13
	2.74
	-0.47
	3.02
	2.74
	1.28
	1.51

	0.99
	0.85
	0.17
	-4.19
	-2.46
	0.11
	-0.23
	4.02
	-0.56
	0
	5.62
	1.77
	0.58
	0.84
	-2.53
	-1.17
	0.08
	4.7
	-1.09
	0.84
	4.34
	2.2
	-0.96
	-0.66
	-2.04
	-1.57
	1.59
	1.42
	7.85
	-1.61
	0.09
	-1.04
	-0.57
	-1.8
	-1.4

	0.54
	1.19
	0.96
	1.98
	2.31
	1.65
	3.29
	4.79
	-0.91
	-0.41
	0.17
	1.04
	-0.98
	1.53
	0.22
	6.74
	4.09
	5.96
	3.74
	2.3
	1.38
	2.69
	2.1
	0.49
	0.11
	2.87
	-0.94
	-1.69
	9.03
	6.04
	6.74
	5.44
	4.49
	7.59
	6.85

-1.22
-1.92
4.09
2.32
1.26
-1.93
-1.72
-0.73
0.64
-1.74
0.46
-0.42
-5.93
2.21
1.92
0.15
2.16
-0.45
0.16
1.15
1.85
1.79
-0.25
5.87
-0.93
1.38
0.26
-2.44
1.35
6.04
1.16
-2.33
-0.59
0.95
1.67

	0.51
	-1.29
	3.16
	0.38
	0.61
	-0.06
	0.72
	-1.05
	0.76
	-0.66
	-1.98
	-0.75
	-2.54
	1.73
	-0.35
	0.47
	-1.31
	1.51
	1.6
	0.01
	2.35
	0.51
	0.71
	1.63
	0.68
	1.66
	1.44
	3.02
	0.43
	2.79
	-0.26
	6.13
	5.84
	2.76
	-0.6

-2.76
-0.52
-0.83
0.06
-0.7
-1.27
6.54
-2.5
0.58
-0.79
-1.64
2.26
-1.03
1.07
-1.37
0.98
-3.66
2.48
1.74
-0.98
-2.29
-0.66
-0.04
-1.33
1.72
-0.1
-0.84
0.5
0.25
-1.33
-1.24
0.91
-2.97
-5.51
-3.45

	1.13
	-3.62
	-2.74
	-2.3
	-4.48
	1.99
	0.16
	-1.07
	-0.9
	-0.61
	-0.65
	-0.39
	0.95
	-0.41
	1.18
	1.86
	0.57
	-1.54
	1.56
	1.26
	0.42
	-2.18
	0.02
	-1.31
	-4.21
	-2.03
	-0.54
	-2.31
	-1.76
	-4.28
	-1.32
	-1.47
	-0.51
	-1.72
	-1.02

	-0.96
	-5.62
	-5.35
	-0.37
	1.44
	0.97
	-0.6
	-3
	-1.11
	-2.36
	-3.57
	-0.52
	-0.2
	-2.52
	-1.31
	0.71
	-1.47
	-0.69
	-1.62
	-1.87
	3.37
	-0.5
	-0.88
	0.77
	-0.68
	-0.67
	-0.54
	-1.06
	0.98
	1.35
	-0.35
	0.81
	-0.6
	-2.72
	-0.39

	0.85
	-0.4
	-0.89
	0.72
	1.78
	-1.39
	-0.45
	-0.55
	-1.34
	-2.25
	-3.46
	-2.72
	-3.02
	-2.55
	-4.21
	-3.68
	-2.16
	-3.66
	0.77
	0.46
	-3.14
	0.57
	-0.67
	2.63
	-0.56
	3.63
	0.87
	3.01
	1.98
	-0.66
	-0.77
	-2.5
	-0.72
	1.5
	1.76

3.35
-2
3.53
6.47
3.77
0.23
2.21
1.99
-3.1
4.22
5.47
-4.1
-3.79
4.48
-2

Log2 Fold Change: (SM_pos_12022024_Newanalyis) / (Rabbitserum_12022024_Newanalysis)
-1.13
-3.17
-4.59
-2.41
-4.1
-1.1
-1.54
-2.57
0.73
2.64
2.67
0.27
-1.14
1.53
1.82
0.77
1.75
-5.99
-5.64
-5.84
-1.06
-4.99
-3.1
-5.78
-5.34
2.07
0.16
1.2
-1.62
5.26
0.86
-0.03
5.44

	3.63
	-2.52
	-4.44
	-5.79
	-0.08
	-1.58
	-6.03
	4.33
	-0.18
	0.53
	-0.84
	4.27
	-3.8
	-3.62
	-5.26
	-5.65
	-1.97
	-5.96
	-2.84
	-4.15
	-2.99
	-2.83
	2.5
	-0.45
	2.45
	-1.07
	-3.67
	0.86
	4.03
	-3.2
	-3.13
	-3.7
	2.82
	-0.45
	5.76

	0.61
	0.15
	2.67
	5.23
	-0.19
	5.48
	0.19
	-1.79
	-3.51
	-3.86
	-6.74
	-9.43
	-4.77
	3.07
	3.89
	0.38
	-3.72
	1.68
	1.18
	-5.14
	3.15
	-0.67
	-10.31
	-6.58
	-2.25
	-2.27
	-3.34
	-1.29
	-3.91
	-2.01
	-1.87
	-1.1
	-4.3
	4.58
	2.85

	1.19
	-3.45
	3.69
	0.72
	1.36
	-1.03
	2.63
	1.47
	-3.98
	-1.18
	1.16
	3.71
	0.51
	3.16
	-6.97
	-6.99
	-2.35
	3.58
	-5.6
	-3.42
	-0.13
	3.02
	-2.05
	-0.21
	-3.69
	0.39
	0.47
	-1.15
	-2.5
	-9.84
	-3.51
	-4.85
	-1.17
	-1.84
	0.09

	3.88
	-0.16
	-1.61
	1.21
	0.47
	0.29
	-0.11
	1.06
	0.13
	-0.61
	0.69
	-2.12
	-4.16
	-2.69
	-3.67
	-2.31
	3.35
	0.13
	-0.89
	-1.32
	0.08
	-1.87
	-3.95
	-2.68
	3.03
	-8.5
	-5.18
	0.07
	-0.71
	-3.26
	-2.77
	-0.54
	-5.99
	0.28
	-2.57

	0.82
	-6.21
	-5.77
	-3.98
	-2.83
	-4.31
	-0.72
	-1.51
	1.44
	-2.04
	-5.38
	3.03
	-3.91
	-1.6
	0.64
	-1.65
	-2.2
	2.8
	-2.08
	-2.88
	-1.99
	-2.01
	-2.25
	1.81
	-1.27
	-1.02
	-4.09
	3.11
	-1.7
	-10.13
	0.99
	-5.92
	-2.8
	-7.22
	1.46

-1.55
-0.22
0.67
1.49
-0.39
5.46
3.11
-0.31
2.99
0.68
0.41
0.61
0.21
0.97
-0.4
-1.85
-3.64
-6.35
-2.57
-2.11
-1.54
-3.05
-0.38
0.35
-2.81
-6.47
2.13
-1.29
-0.52
-2.74
-0.99
1.11
-1.71
-1.51
-3.72

	-0.42
	-1.43
	0.76
	-0.24
	-3.93
	-0.41
	-2.04
	0.28
	0.25
	-2.79
	-1.31
	-0.77
	-2.98
	-2.35
	-0.44
	-1.12
	-3.63
	0.26
	1.47
	-7.2
	-1.25
	3.05
	-2.87
	4.09
	-4.25
	0.41
	-1.52
	-0.66
	-0.32
	-1.51
	-0.4
	-3.12
	0.34
	-1.06
	-2.33

	-2.07
	-5.76
	-0.79
	-0.99
	2.95
	-1.2
	-3.25
	-0.1
	2.27
	-3.29
	1.51
	-3.18
	0.34
	0.76
	2.96
	1.65
	-6.77
	-2.43
	0.87
	-2.19
	-4.66
	-5.57
	1.37
	-3.81
	-3.75
	-3.99
	-2.86
	-2.98
	-2.97
	-2.23
	-4.65
	-2.44
	-0.18
	2.92
	-0.21

	-2.65
	-2.56
	-2.76
	-6.91
	2.21
	-2
	-1.43
	-4.68
	5.54
	-2.07
	-0.44
	2.11
	1.04
	0.67
	4.28
	-2.03
	-2.19
	-4.38
	-4.18
	-3.06
	2.83
	-1.36
	0.84
	-4.73
	-2.97
	3.72
	2.98
	-3.37
	0.98
	-2.77
	-2.62
	-1.02
	-2.13
	-0.14
	-0.8

	-2.09
	-2.01
	-0.34
	3.18
	-4.04
	-0.35
	-3.36
	-3.16
	-2.4
	-1.91
	-3.6
	-2.84
	-3.13
	-2.59
	0.81
	-5.55
	-2.02
	-3.01
	-1.99
	-2.47
	-2.66
	-2.4
	2.66
	-3.93
	-2.89
	0.37
	-2.47
	-3.5
	-4.84
	-0.63
	-2.46
	-6.83
	-4.27
	-3.49
	3.56

	-0.06
	3.57
	-4
	-2.35
	-5.15
	-3.64
	-3.29
	-3.46
	-6.83
	-3.14
	-2.76
	-5.65
	-4.14
	-6.45
	-0.78
	-2.36
	-2.92
	-1.55
	-3.16
	1.02
	1.22
	0.21
	-2.7
	-3.72
	0.95
	0.62
	-10.65
	2.48
	0.27
	-3.15
	-7.41
	0.64
	-1.41
	-0.94
	2.02

	-3.13
	3.63
	0.38
	0.86
	3
	0.42
	5.21
	-2.1
	-3.29
	-3.76
	-2.29
	-2.58
	-3.54
	1.85
	-2.27
	0.27
	-6.06
	-3.18
	0.14
	-0.52
	-2.44
	-3.02
	-1.4
	0.47
	1.46
	2.7
	-2.16
	-7.16
	-0.24
	-6.13
	-1.08
	-1.56
	-2.27
	-4.15
	-2.77

	-7.72
	-5.47
	-3.48
	2.32
	1.54
	-0.58
	-0.82
	-0.16
	-3.06
	-3.55
	-1.32
	3.04
	-4.03
	1.81
	-2.66
	0.15
	-2.29
	2.74
	-0.71
	-2.66
	-4.55
	-0.49
	-4.12
	-3.53
	-3.26
	-2.55
	0.85
	-3.68
	-1.19
	-1.04
	-2.99
	-2.57
	-2.81
	-0.03
	2.06

	1.89
	-1.88
	1.75
	-2.56
	-1.02
	-2.78
	2.48
	-2.42
	4.2
	3.21
	3.27
	-2.09
	-4.82
	1.25
	-1.7
	-2.49
	-1.98
	-0.27
	-3.31
	1.86
	-0.88
	2.18
	5.09
	-0.21
	-0.79
	-3.89
	-0.28
	-6.97
	-3.31
	-2.69
	-0.85
	-2.94
	-4.12
	-0.95
	-2.96

	1.02
	0.1
	-0.45
	1.58
	2.19
	-2.86
	-4.28
	0.02
	-7.61
	-3.36
	-1.26
	-1.53
	-1.12
	-1.12
	-4.23
	0.46
	-2.13
	2.19
	-2.64
	1.07
	-1.59
	-10.08
	3.21
	-3.75
	2.05
	-3.44
	-7.58
	0.56
	4.54
	1.75
	-1.07
	-2.55
	1.82
	-1.33
	-4.12

	-5.18
	-1.38
	-3.33
	-0.1
	1.56
	-0.19
	1.21
	-0.86
	-1.92
	2.66
	-0.83
	-1
	-2.02
	-4
	-1.16
	-2.29
	-5.76
	2.06
	0.78
	0.75
	-3.09
	-3.35
	0.8
	3.85
	-1.7
	-0.39
	5.11
	0.55
	1.31
	3.75
	-0.67
	-0.84
	-2.36
	0.53
	1.79

	3.33
	-0.85
	1.26
	1.32
	-1.85
	-2.93
	-5.29
	-3.61
	-3.2
	5.11
	-3.42
	-2.16
	-4.66
	0.66
	-3.52
	2.44
	-1.59
	-1.21
	3.11
	-1.02
	0.49
	2.23
	-0.3
	3.86
	3.87
	-0.04
	3.88
	-4.39
	-0.56
	0.13
	-3.8
	0.13
	-3.12
	4.63
	-3.79

	3.22
	-5.09
	-6.74
	-0.73
	-8.23
	2.14
	0.65
	3.81
	0.92
	-2.26
	-5.61
	-0.09
	-0.97
	-2.58
	3.84
	2.33
	-0.94
	1.37
	-1.05
	0.6
	-1.93
	0.36
	0.25
	-1.34
	0.51
	0.76
	0.7
	3.69
	0.42
	3.14
	3.32
	2.05
	-0.88
	-5.75
	-0.46

	2.42
	0.53
	6.42
	-0.65
	-3.81
	-4.52
	-0.44
	-6.8
	-0.44
	-2.96
	-9.35
	-7.9
	-6.49
	-4.33
	-3.69
	-2.54
	-4.28
	2.54
	1.97
	-1.29
	-1.23
	-0.67
	-2.72
	-2.88
	-2.56
	-1.68
	-4.28
	0.46
	0.58
	1.71
	-7.04
	-1.32
	0.08
	1.53
	-1.46

	-1.48
	0.82
	-0.6
	1.26
	-2.5
	-3.28
	-3.51
	0.76
	1.9
	0.37
	0.53
	1.87
	-0.6
	0.6
	2.56

Log2 Fold Change: (Bb_pos_12022024_Newanalysis) / (SM_pos_12022024_Newanalyis)
-1.68
3.58
-0.19
-5.91
3.11
1.37
4.21
-0.69
-2.34
-1.75
-2.91
1.07
-3.45
-2.57
-0.55
2.5
-1.66
-0.82
1.21
0.47
1.18
-1.54
-2.35
-1.18
-2.31
-2.28
-0.06
-0.01
3.16
-4.85
0.69
5.83
-2.54

	-1.77
	-3.62
	0.48
	-0.44
	6.6
	7.19
	-1.45
	-3.99
	-1.91
	-4.02
	-1.38
	-3.28
	-2.18
	-3.07
	-1.01
	0.26
	-1.61
	0.16
	-1.61
	-2.75
	-1.47
	2.78
	-4.36
	-1.26
	-0.52
	-1.66
	3.13
	-4.21
	-4.03
	4.48
	-4.1
	-0.52
	-3.13
	-3.49
	-10.8

	-5.21
	-1.97
	-5.5
	-5.05
	-3.82
	-11.98
	-3.79
	2.45
	-3.69
	1.02
	-0.1
	2.26
	-1.4
	-6.2
	-3.02
	-4.34
	-3.84
	-4.05
	-0.03
	-1.32
	-5.99
	-1.47
	3.91
	0.31
	-2.18
	-2.2
	-1.18
	-3.32
	-2.09
	-3.43
	1.1
	-0.82
	-1.61
	-3.56
	-6.3

	-0.79
	-0.85
	-2.18
	-4.24
	-2.37
	0.39
	-1.5
	-4.16
	0.28
	-1.32
	-5.35
	-3.14
	-3.7
	-4.16
	-0.42
	1.44
	0.71
	-3.43
	-0.87
	1.65
	1.82
	-4.82
	0.29
	-3.34
	2.62
	-3.16
	0.49
	0.59
	-1.02
	2.75
	0.2
	4.3
	3.16
	-1.83
	-1.71

	-4.22
	-1.11
	3.13
	-1.78
	1
	1.12
	1.15
	-1.26
	2.77
	-0.41
	0.28
	-1.97
	-2.97
	1.16
	-0.89
	-3.41
	-0.68
	-2.08
	3.94
	-0.32
	-3.7
	-2.37
	-1.13
	-2.56
	-4.56
	1.13
	4.78
	-2.69
	-3.18
	2.23
	-2.34
	-3.62
	4.78
	-3.24
	-3.15

	-0.26
	2.1
	-0.17
	-2.5
	-2.68
	-0.66
	-5.23
	-2.12
	0.66
	-5.16
	4.16
	-2.88
	-3.25
	-2.43
	1.75
	-2.41
	-1.32
	-6.44
	-4.32
	-1.37
	-2
	-1.74
	2.33
	-1.67
	2.66
	0.08
	0.11
	-3.69
	-4.05
	2.28
	-6.77
	0.03
	-2.75
	2.13
	-7.68

	-3.43
	-1.86
	-2.32
	-1.78
	1.6
	-5.93
	-5.5
	-2.51
	-4.22
	-1.39
	0.92
	-1.24
	-2.98
	-1.91
	2.34
	4.24
	0.55
	2.43
	-0.46
	1.92
	-3
	0.73
	1.46
	-4.93
	-2.2
	0.39
	-2.44
	0.77
	2.1
	1.88
	-0.21
	-1.05
	0.31
	0.35
	-1.27

	0.52
	-0.47
	-0.23
	-0.83
	1.95
	-2.85
	-1.24
	-1.03
	-2.11
	-3.87
	1.47
	-0.3
	0.88
	-0.98
	2.28
	0.54
	4.04
	0.36
	-0.87
	-0.45
	-2.12
	-2.98
	2.02
	-3.95
	3.57
	-0.54
	-0.93
	0.43
	-1.53
	-1.1
	-2.39
	-2.47
	1.74
	-2.14
	-3.55

	-1.44
	-0.66
	-2.03
	-2.88
	-6.13
	-1.83
	-2.79
	-0.27
	-3.7
	-1.3
	-1.25
	0.44
	0.52
	-1.91
	-3.83
	-1.54
	-0.25
	0.3
	-3.08
	-0.16
	-1.53
	3.57
	-2.44
	-2.48
	-0.22
	0.25
	-0.48
	-1.13
	1.82
	-2.17
	0.39
	-3.1
	1.41
	-6.57
	-1.5

-2.17
3.42
-1.1
2.3
0.46
4.32
-3.46
-2.3
-6.02
-3.1
-1.61
0.63
-3.78
-3.51
-2.78
0.78
-0.27
-1.23
-2.1
0.56
-4.52
1.14
-3.36
-1.77
-1.21
-1.67
0.16
4.33
-2.53
6.37
6.18
-3.77
1.63
4.44
2.67

	3.37
	0.91
	6.19
	1.37
	-0.22
	-0.22
	0.61
	-2.08
	-0.54
	-1.73
	2.69
	-0.91
	-0.31
	4.11
	-1.41
	2.1
	1.68
	0.45
	-2.9
	-0.16
	-3.26
	-3.68
	-2.07
	-0.54
	-3.15
	0.34
	-1.65
	-0.66
	-0.04
	-4.75
	-0.75
	2.55
	0.75
	-1.2
	-4.33

	-2.09
	-5.05
	2.5
	0.01
	2.66
	-0.16
	-0.1
	-1
	2.51
	3.6
	-0.86
	-0.19
	0.46
	-0.75
	-6.56
	-0.25
	-1.25
	-3.91
	-3.96
	-5.22
	0.64
	-2.55
	0.45
	-0.44
	-2.39
	1.14
	9.18
	-7.14
	0.63
	-1.44
	-0.46
	4.47
	-3.91
	-4.75
	-6.84

	-2.19
	-8.19
	-4.29
	-8.63
	-5.47
	-0.27
	-3.15
	0.85
	-0.8
	0.94
	0.88
	-3.06
	-4.35
	-4.81
	-0.29
	-3.26
	5.83
	2.47
	-4.57
	-7.17
	-0.39
	-6.33
	-3.42
	-5.68
	-3.74
	-6.73
	-1.83
	0.18
	-2.28
	7.42
	-1.03
	0.3
	-3.06
	-4.05
	-4.6

	0.55
	0.57
	-0.51
	-4.07
	-1.73
	-0.09
	-5.97
	-4.4
	1.56
	0.7
	-2.81
	-2.05
	6.41
	3.35
	6.29
	5.69
	7.68
	3.98
	-1.64
	-0.31
	2.86
	-2.81
	2.53
	0.95
	3.67
	5.88
	3.91
	-0.01
	0.56
	-0.37
	0.54
	4.84
	4.17
	6.82
	1.05

	-1.07
	4.4
	-4.29
	-2.39
	1.3
	-2.16
	0.92
	2.5
	-1.6
	-1.07
	-1.15
	-3.31
	-0.58
	-3.93
	-1.46
	-2.03
	2.31
	-1.79
	5.91
	-4.16
	-3.45
	-3.22
	-5.9
	0.32
	-2.92
	-1.44
	0.53
	3.24
	-0.4
	0.56
	-3.75
	-3.22
	-1.53
	2.66
	-2.36

	-5.15
	-2.72
	-4.4
	1.17
	-9.46
	0.55
	-2.11
	-0.59
	-2.17
	-3.56
	-1.79
	-2.92
	-1.19
	3.83
	-0.87
	-2.53
	-3.02
	-10.07
	-3.3
	1.71
	-7.69
	3.52
	-12.58
	0.9
	-6.7
	0.79
	-2.03
	-2.88
	-7.83
	-10.73
	-1.3
	-0.2
	-0.73
	5.68
	4.96

	6.67
	0.75
	3.83
	2.86
	2.55
	1.7
	-1.79
	-2.4
	-1.54
	-1.76
	2.21
	-0.11
	6.44
	-0.89
	-1.88
	-3.4
	5.02
	0.76
	-0.36
	0.39
	6.08
	-2.86
	-1.78
	-0.28
	3.77
	3.79
	-1.54
	2.12
	1.92
	-5.02
	0.45
	-2.06
	0.2
	-0.03
	-1.99

	-0.01
	1.51
	0.66
	1.08
	2.52
	1.75
	-2.12
	-1.1
	-2.38
	-4.26
	1.55
	4.83
	-0.31
	-1.75
	-3.58
	2.54
	3.74
	1.3
	-1.13
	1.69
	0.64
	2.43
	-1.07
	-3.45
	-1.23
	1.3
	-1.9
	-0.52
	-0.78
	-0.87
	-1.27
	-2.37
	-0.15
	-2.76
	1.19

-5.28
0.18
-0.77
-3.12
2.09
-5.8
-4.39
-9.88
-7.33
-1.57
3.45
1.74
-2.49
2.27
-1.03
-3.25
1.63
3.84
3.9
2.43
2.39
0.6
0.69
-1.08
0.3
0.37
0.2
-2.16
-1.69
-2.9
-1.88
-1.61
-2.66
1.59
-3.37

	-3.93
	-0.18
	-6.26
	-2.56
	-0.31
	1.18
	-2.05
	0.32
	-1.09
	-4.04
	2.43
	0.9
	-0.6
	0.69
	0.63
	-2.5
	1.13
	-2.66
	-1.22
	1.22
	4.02
	-0.07
	7.05
	1.9
	1.3
	-1.6
	6.9
	2.72
	-3.24
	-4.17
	1.93
	-2.23
	1.36
	-1.02
	-3.03

	0.7
	0.77
	-0.31
	-2.19
	0.87
	-3.24
	-1.5
	-0.3
	-3.37
	-2.79
	0.07
	-0.22
	1.63
	1.17
	-13.84

Log2 Fold Change: (CM_pos_12022024_Newanalysis) / (SM_pos_12022024_Newanalyis)
-1.09
2.55
-0.54
-0.65
2.2
1.49
3.42
1.43
-2.7
-0.93
-1.9
0.75
-1.5
-1.19
-0.69
1.4
-2.11
3.88
2.67
2.2
1.06
2.74
1.74
2.29
3.59
-0.86
-0.22
-0.85
2.52
-4.06
0.24
-0.69
-4.06

	-2.5
	1.53
	-1.49
	2.34
	0.02
	0.65
	4.63
	-4.19
	-2.33
	-0.57
	-2.42
	-3.74
	2.04
	1.6
	3.52
	4.37
	1.17
	4.89
	2.16
	2.54
	1.27
	1.6
	-3.23
	-2.54
	-2.22
	-1.05
	1.73
	-3.88
	-4.73
	3.26
	1.89
	2.89
	-3.2
	1.57
	-8.52

-4.79
-0.92
-0.71
-5.6
-1.1
-8.18
-3.43
1.64
1.8
0.74
4.6
5.71
2.87
-1.62
-3.28
-1.37
-2.45
-1.57
-0.27
4.98
-3.63
-0.29
9.26
4.62
0.21
0.44
1.31
-1.27
2.89
0.32
1.48
1.13
0.44
-2.39
-3.8

	-0.53
	1.44
	-2.94
	-1.68
	-1.91
	-0.48
	-0.99
	-2.16
	0.97
	2.55
	-2.62
	-3.65
	-1.64
	-3.25
	6
	5.51
	0.73
	-4.19
	-0.77
	1.47
	1.02
	-4.38
	0.9
	-0.69
	3.08
	-3.2
	-0.06
	-0.08
	0.82
	6.8
	1.62
	5.58
	0.87
	0.57
	-1.57

	-4.21
	-1.73
	2.14
	-1.38
	0.26
	0.28
	0.65
	-1.37
	0.9
	-0.55
	-0.71
	-2.71
	-0.86
	0.01
	-0.48
	-0.29
	-2.85
	-1.84
	2.51
	-0.74
	-2.9
	-1.42
	-0.46
	0.2
	-4.74
	5.68
	3.74
	-0.76
	-2.82
	1.69
	-0.77
	-0.15
	3.74
	-1.79
	-1.27

	-2.5
	1.31
	4.26
	-0.76
	1.07
	0.8
	-1.29
	-1.7
	-0.72
	-4.86
	2.24
	-3.32
	-0.16
	-0.06
	0.65
	-1.81
	-1.37
	-4.28
	-0.62
	-0.08
	-0.86
	-0.22
	1.46
	-1.92
	1.89
	0.07
	1.9
	-1.36
	0.32
	6.42
	-2.56
	3.93
	-0.8
	3.35
	-4.72

	0.68
	-1.68
	-1.76
	-1.36
	0.83
	-5.88
	-4.76
	-2.45
	-4.5
	-2.19
	0.26
	-0.34
	-1.19
	-0.43
	-0.03
	1.66
	4.26
	0.97
	1.65
	1.67
	-0.61
	-0.02
	2.78
	-4.16
	-0.57
	2.29
	-2.51
	0.86
	1.09
	2.07
	-1.41
	-1.32
	-0.43
	0.29
	1.17

	-0.75
	-0.79
	-0.41
	0.22
	2.56
	-2.1
	-0.54
	-1.18
	-1.62
	-1.43
	-0.38
	-1.14
	-0.19
	0.05
	-2.17
	-2.34
	1.98
	-2.74
	-0.68
	5.13
	-1.44
	-6.37
	0.81
	-5.99
	3.01
	-0.68
	0.23
	-0.64
	-2.67
	-1.48
	-1.07
	1.01
	0.49
	-0.53
	-0.75

	-1.4
	2.51
	-1.02
	-4.25
	-6.21
	-0.37
	-0.14
	-0.35
	-3.02
	-0.75
	-2.7
	-0.09
	0
	-2.22
	-2.76
	-2.54
	2.18
	-0.34
	-0.67
	-0.13
	3.84
	1.05
	-2.06
	-1.26
	-0.51
	0.1
	-0.11
	0.39
	4.47
	-0.33
	2.18
	-0.13
	-1.72
	-4.48
	-0.59

	-1.08
	1.68
	1.17
	3.46
	-0.06
	0.26
	-0.62
	0.49
	-6.46
	-1.14
	-0.86
	-0.53
	-1.73
	-1.2
	-4.63
	-0.23
	-0.36
	2.03
	1.66
	-1.76
	-2.68
	-1.8
	-1.16
	0.9
	-0.26
	-3.69
	-2.04
	0.37
	0.34
	-0.81
	-2.17
	-2.11
	-1.47
	-1.88
	-0.64

	0.45
	1.09
	2.26
	-2.83
	-0.18
	-0.49
	-0.03
	-1.12
	-0.33
	-0.35
	0.3
	-0.38
	-1
	2.61
	-1.57
	4.41
	0.35
	0.06
	-0.31
	-0.94
	-0.12
	-1.6
	-0.99
	1.08
	-0.31
	-0.52
	0.05
	1.07
	1.54
	-2.9
	-0.33
	3.69
	1.45
	2.87
	-4.79

	-2.76
	-4.65
	1.5
	-0.38
	3.54
	-0.09
	0.89
	-0.31
	3.82
	1.98
	-0.79
	-0.06
	0.17
	2.97
	-2.36
	1.62
	1.32
	-0.13
	0.58
	-2.43
	-0.63
	-1.23
	1
	-0.71
	-0.62
	-1.41
	7.68
	-4
	0.25
	1.21
	4.04
	0.47
	-1.46
	-0.92
	-4.42

	0.04
	-5.77
	-3.19
	-2.49
	-5.38
	-2.07
	-6.04
	0.53
	-0.86
	0.68
	0.47
	-0.29
	0.05
	-2.28
	-1.31
	-2.94
	3.22
	1.76
	-1.76
	-2.27
	0.8
	-0.62
	-1.02
	-1.78
	-3.83
	-4.22
	-2.21
	3.78
	-0.2
	5.55
	-2.15
	-0.33
	-1.43
	0.66
	-0.73

	4.64
	2.58
	-0.92
	-1.99
	-2.36
	-0.81
	-1.76
	-2.63
	0.45
	-1.05
	-1.83
	-5.17
	0.64
	-3.08
	-0.7
	-0.93
	-0.54
	-3.39
	-0.14
	0.04
	1.88
	-3
	0.55
	-0.24
	-0.33
	-0.28
	-3.25
	0.2
	1.25
	-0.36
	0.44
	0.42
	-0.33
	-0.34
	-1.76

-1.27
2.29
-1
-0.29
-1.99
-2.73
0.47
1.91
-2.17
0.38
-1.39
-1.93
-0.63
-4.18
-1.06
0.65
-0.96
-2.67
2.06
-3.87
-2.5
-4.05
-6.35
-0.1
-1.52
-0.85
-1.95
2.77
-0.28
0.1
-1.48
0.98
-1.39
1.29
-1.05

	-5.33
	-3.18
	-3.7
	0.53
	-8.16
	0.69
	0.87
	-1.18
	3.54
	-2.01
	-1.67
	-2.39
	-2.08
	3.11
	0.14
	-1.23
	-0.92
	-6.93
	-2.19
	-0.14
	-1.77
	5.64
	-7.25
	-0.82
	-6.54
	0.7
	3.28
	-2.75
	-7.82
	-5.28
	-2.21
	0.84
	-0.11
	1.24
	4.44

	0.21
	0.66
	-1.63
	0.94
	1.26
	0.95
	-1.33
	-0.62
	-0.96
	-3.06
	2.17
	0.84
	5.16
	0.89
	-0.64
	0.52
	5.67
	-1.83
	-1.73
	-0.18
	4.4
	2.02
	-2.76
	-0.29
	0.49
	0.05
	-3.56
	1.61
	-0.35
	-4.77
	1.03
	-1.49
	-0.46
	0.1
	-2.58

	-0.94
	1.17
	-0.2
	1.83
	2.98
	0.72
	2.27
	0.85
	-0.09
	-5.68
	1.76
	-0.56
	1.06
	-2.17
	0.42
	0.64
	-0.27
	0.92
	-3.26
	-1.99
	-0.07
	1.14
	-1.21
	-2.34
	-1.95
	0.52
	-3.32
	1.15
	-1.85
	-0.91
	0.93
	-1.72
	0.23
	-2.7
	0.68

	-4.31
	5.13
	4.86
	-0.75
	7.34
	-2.83
	-1.54
	-7.17
	-2.18
	1.11
	4.33
	1.72
	0.42
	2.42
	-1.9
	-3.19
	1.64
	-0.27
	3.13
	1.22
	2.22
	-0.06
	0.33
	-1.3
	-0.07
	-0.01
	-0.41
	-3.06
	-1.32
	-2.53
	-1.75
	-1.44
	-0.01
	2.82
	-0.64

	-3.54
	-0.44
	-7.02
	-2.76
	0.62
	0.7
	-1.14
	4.12
	-1.6
	1.25
	7.58
	6.08
	3.98
	1.89
	0.95
	-1.26
	1.01
	-1.65
	-1.84
	0.74
	1.85
	-0.89
	4.94
	2.72
	0.83
	-0.94
	0.65
	0.05
	-3.28
	-1.72
	3.1
	-0.4
	0.33
	-1.6
	-0.97

	0.03
	-0.12
	1.88
	-1.01
	1.78
	0.39
	1.51
	-0.27
	-1.15
	-1.48
	-0.05
	-1.53
	-0.27
	-0.78
	-9.3

Log2 Fold Change: (Bb_pos_12022024_Newanalysis) / (Water_pos_12022024_Newanalysis)
-1.32
5.44
0.22
-0.89
2.24
2.16
6.04
-0.42
-0.28
0.26
-0.3
4.43
-1.69
1.81
-0.35
2.65
-1.03
-0.15
-1.3
1.68
0.31
-0.74
0.13
-0.72
-0.96
-0.91
-0.53
-0.67
1.92
-2.61
-0.72
3.51
-0.3

	2.61
	-0.83
	1.79
	-1.83
	5.31
	4.66
	-3.35
	-0.54
	-1.45
	-1.31
	0.75
	-1.27
	-1.26
	-1.88
	-1.43
	-1.5
	-1.35
	-1.5
	-1.35
	-1.88
	-1.36
	-0.19
	-3.11
	0.73
	0.6
	-0.73
	2.75
	-3.92
	-0.13
	4.09
	-1.11
	-2.08
	-1.12
	-3.96
	-1.36

	-1.95
	-1.55
	-4.66
	-1.88
	-1.31
	-2.74
	-2
	0.62
	-1.19
	1.87
	-2.34
	-2.06
	-3.62
	-4.63
	-1.46
	-1.71
	-2.18
	-2.74
	-2.06
	-4
	-2.09
	-2.94
	-3.24
	-1.89
	-1.26
	-0.77
	-1.37
	-2.41
	-3.06
	-3.14
	-2.59
	-1.73
	-0.65
	-2.79
	-1.31

	-2.59
	-1.48
	-1.26
	-2.18
	-1.23
	-0.23
	-1.76
	-1.3
	0.31
	-3.46
	-1.29
	-1.84
	-2.41
	-2.52
	-1.17
	-1.23
	-2.35
	-1.26
	-0.76
	-1.29
	0.48
	-2.52
	0.72
	-1.51
	-0.64
	-2.23
	-1.25
	-1.14
	0.51
	0.76
	0.17
	1.87
	1.76
	-3.39
	0.58

	-1.4
	-0.56
	1.37
	-0.82
	-0.87
	-0.85
	-0.72
	-2.31
	1.69
	-1.25
	0.12
	-0.57
	-0.99
	-0.59
	-0.8
	-1
	1.68
	-0.73
	3.95
	-0.47
	-2.41
	-1.22
	-0.6
	-1.21
	-0.12
	-4.22
	3.27
	-3.62
	-2.23
	1.96
	1.57
	-3.31
	3.27
	-0.19
	0.34

	3.57
	1.75
	-2.48
	0.66
	-0.49
	-0.24
	-1.24
	-1.12
	6.27
	-1.09
	2.86
	-1.02
	-0.89
	1.32
	2.5
	1.87
	-0.76
	-1.47
	-0.66
	-1.2
	-0.23
	-0.63
	2.61
	-1.08
	1.87
	-0.19
	-1.41
	-0.12
	-3.91
	-2.06
	-3.18
	-3.48
	0.53
	1.96
	1.89

	-3.14
	-1
	-1.3
	-0.86
	1.95
	-1.08
	-0.72
	0.05
	-0.99
	1.96
	0.46
	-0.91
	-0.8
	0.62
	1.89
	2.92
	-0.99
	-0.19
	-0.26
	-1.06
	-0.6
	-0.87
	-2.39
	-1.61
	-1.19
	-2.22
	-1.32
	-1.04
	1.11
	-1.29
	0.65
	-0.4
	0.04
	-0.89
	-1.46

	2.75
	1.49
	0.03
	-0.36
	0.61
	-0.72
	1.21
	-1.39
	-0.83
	-0.6
	4.18
	1.49
	0.47
	1.78
	5.4
	3.56
	1.92
	2.48
	-1.07
	-0.61
	-1.06
	2.66
	1.83
	0.63
	1.09
	-0.53
	0.95
	0.83
	1.14
	-0.05
	0.05
	-1.85
	2.59
	-2.28
	0.47

	1.69
	-1.14
	-1.44
	1.33
	-0.23
	0.17
	-0.82
	-0.86
	-2.7
	-1.38
	1.27
	-1.27
	-1.27
	-1.54
	-3.04
	-0.31
	-1.27
	-1.38
	-0.78
	-0.52
	-1.3
	2.3
	-2.79
	-1.27
	-1.34
	-1.14
	-0.1
	-0.63
	2.66
	2.83
	-1.38
	0.86
	4.69
	3.62
	-0.91

	1.07
	3.92
	-0.4
	3.1
	4.72
	5.98
	1.77
	-1.08
	2.84
	-0.92
	0.79
	4.01
	0.35
	-1.46
	1.3
	5.48
	1.59
	-0.32
	-0.69
	3.25
	1.28
	4.78
	-0.31
	-0.36
	-0.68
	3.01
	1.17
	5.6
	-0.03
	9.18
	9.04
	-2.49
	3.67
	5.81
	2.76

	1.59
	-0.65
	5.01
	3.08
	-1.35
	-1.05
	-1.15
	-1.42
	-1.04
	1.38
	5.02
	-0.51
	1.6
	4.19
	-1.06
	-3.46
	0.88
	-0.8
	0.42
	1.11
	-0.68
	-1.43
	-0.36
	-0.7
	1.25
	1.34
	-0.32
	-0.5
	-3.25
	-2.44
	-1.1
	1.15
	1.16
	-3.85
	-0.56

1.21
-2.27
2.83
0.48
1.75
-1.11
0.54
0.76
3.69
4.43
1.53
1.11
-1.14
0.43
1.15
-0.68
-1.85
-0.4
-1.55
-2.62
6.38
-0.5
-1.21
-1.03
3.83
2.11
5.48
2.43
-1.38
-3.48
-1.04
5.37
-0.61
-0.87
-0.42

	0.95
	-1.56
	3.39
	0.32
	3.5
	0.73
	1.99
	-0.66
	0.34
	4.98
	1.28
	0.23
	0.08
	-0.74
	4.18
	2.91
	6.94
	2.09
	-0.52
	-0.71
	0.64
	-1.74
	-0.36
	0.27
	2.54
	-0.36
	0.71
	-0.25
	-0.82
	5.89
	1.61
	4.02
	2.97
	-0.12
	0.46

	-0.55
	-1.38
	1.09
	-1.08
	-1.3
	4.62
	-1.11
	0.72
	0.42
	5.61
	3.86
	7.3
	8.7
	6.92
	9.08
	6.58
	9.24
	5.91
	-1.26
	1.56
	1.32
	-0.62
	3.15
	5.8
	7.54
	6.52
	8.41
	1.83
	-0.78
	-0.95
	1.37
	3.55
	6.32
	6.9
	2.37

	-1.01
	1.01
	-3.6
	-0.16
	3.07
	0.47
	4.41
	0.82
	-0.78
	0.36
	-0.41
	1.82
	-1.42
	0.57
	-0.59
	0.06
	4.2
	0.79
	5.27
	-0.77
	-0.94
	1.47
	-0.59
	-0.64
	-0.76
	-1.25
	3.23
	1.91
	-0.14
	-0.75
	-1.14
	-0.52
	-0.6
	1.61
	-0.76

	-0.35
	-0.77
	0.19
	2.27
	-0.81
	-0.97
	-0.42
	-0.79
	-0.95
	-0.2
	-0.86
	-1.02
	2.18
	3.63
	-0.33
	-0.46
	0.59
	-0.62
	-0.68
	4.24
	-2.07
	2.22
	-0.42
	1.18
	-0.07
	2.85
	-0.73
	1.37
	-1.02
	-1.53
	-0.31
	-0.79
	-0.32
	4.27
	4.94

	5.01
	0.3
	5.11
	-0.12
	0.32
	-0.35
	-0.83
	0.92
	-1.1
	0.78
	-1.53
	0.06
	-0.52
	-0.67
	-0.34
	-2
	3.63
	2.26
	2.02
	-1.22
	0.28
	-1.85
	-1.01
	-0.38
	3.13
	2.9
	0.89
	1.94
	2.09
	-0.77
	2.18
	1.58
	-1.74
	4.95
	-1.17

	2.14
	-1.04
	-1.03
	-1.42
	-1.61
	2.49
	-1.25
	-1
	0.82
	-0.24
	0.18
	3.87
	-1.72
	-1.45
	-0.72
	0.32
	3.8
	-0.85
	-0.27
	3.46
	-1.03
	-0.36
	-1.02
	-2.31
	-0.62
	-1.37
	-0.26
	-0.54
	2.81
	0.39
	-0.7
	-1.03
	-0.69
	-0.48
	3.54

	-1.52
	-3.09
	-1.4
	-1.12
	-3.16
	-2.3
	-1.5
	-2.48
	-2.45
	-1.3
	-0.09
	-2.15
	-1.82
	-2.11
	-0.77
	-2.48
	-1.63
	3.43
	0.78
	1.96
	-0.81
	-1.42
	-1.3
	0.64
	-1.36
	-1.55
	-1.25
	-0.97
	-0.42
	-0.12
	-2.53
	-2.56
	-1.51
	-2.79
	-1.44

	-2.49
	-2.09
	-0.17
	-0.19
	-1.62
	1.79
	-1.12
	-3
	0.02
	-2.06
	-0.75
	-2.58
	-1.89
	-0.9
	-1.42
	1.99
	1.43
	-1.12
	0.71
	0.71
	2.9
	0
	8.11
	2.05
	-0.75
	-1.25
	4.37
	1.35
	0.01
	-2.98
	-1.86
	-0.87
	0.21
	-1.52
	-1.19

	0.44
	3.24
	-0.3
	0.62
	1.74
	-1.29
	-1.49
	0.21
	-0.16
	-2.65
	-1.23
	-0.58
	-0.67
	-0.62
	-1.04

Log2 Fold Change: (CM_pos_12022024_Newanalysis) / (Water_pos_12022024_Newanalysis)
-0.74
4.4
-0.13
4.37
1.33
2.28
5.25
1.69
-0.65
1.08
0.72
4.11
0.27
3.19
-0.49
1.54
-1.48
4.56
0.16
3.4
0.18
3.54
4.21
2.75
4.94
0.51
-0.69
-1.51
1.28
-1.82
-1.16
-3.01
-1.82

	1.88
	4.32
	-0.17
	0.96
	-1.27
	-1.87
	2.73
	-0.75
	-1.87
	2.14
	-0.28
	-1.73
	2.96
	2.79
	3.11
	2.61
	1.42
	3.23
	2.42
	3.41
	1.38
	-1.37
	-1.98
	-0.55
	-1.1
	-0.11
	1.35
	-3.59
	-0.83
	2.87
	4.88
	1.33
	-1.19
	1.1
	0.92

	-1.53
	-0.5
	0.14
	-2.43
	1.41
	1.07
	-1.64
	-0.19
	4.3
	1.59
	2.36
	1.39
	0.65
	-0.05
	-1.71
	1.26
	-0.79
	-0.26
	-2.29
	2.3
	0.27
	-1.75
	2.11
	2.43
	1.13
	1.87
	1.12
	-0.35
	1.92
	0.6
	-2.2
	0.22
	1.4
	-1.62
	1.19

	-2.32
	0.81
	-2.02
	0.38
	-0.78
	-1.1
	-1.25
	0.7
	0.99
	0.41
	1.44
	-2.35
	-0.36
	-1.61
	5.26
	2.84
	-2.32
	-2.02
	-0.66
	-1.46
	-0.32
	-2.07
	1.33
	1.14
	-0.17
	-2.27
	-1.81
	-1.81
	2.35
	4.81
	1.59
	3.14
	-0.53
	-0.99
	0.72

	-1.39
	-1.18
	0.37
	-0.42
	-1.61
	-1.69
	-1.21
	-2.42
	-0.18
	-1.39
	-0.87
	-1.31
	1.12
	-1.74
	-0.39
	2.12
	-0.49
	-0.5
	2.52
	-0.9
	-1.62
	-0.26
	0.07
	1.55
	-0.31
	0.33
	2.23
	-1.69
	-1.87
	1.41
	3.14
	0.16
	2.23
	1.25
	2.23

	1.32
	0.96
	1.95
	2.4
	3.26
	1.21
	2.7
	-0.7
	4.9
	-0.8
	0.93
	-1.46
	2.2
	3.69
	1.4
	2.47
	-0.8
	0.68
	3.04
	0.09
	0.91
	0.89
	1.73
	-1.33
	1.11
	-0.2
	0.38
	2.21
	0.46
	2.07
	1.03
	0.43
	2.48
	3.17
	4.85

	0.96
	-0.82
	-0.74
	-0.44
	1.19
	-1.04
	0.03
	0.11
	-1.27
	1.16
	-0.2
	-0.01
	1
	2.1
	-0.48
	0.34
	2.72
	-1.65
	1.85
	-1.31
	1.79
	-1.63
	-1.07
	-0.84
	0.44
	-0.31
	-1.39
	-0.95
	0.1
	-1.1
	-0.54
	-0.67
	-0.69
	-0.95
	0.97

	1.48
	1.18
	-0.15
	0.69
	1.23
	0.02
	1.9
	-1.54
	-0.34
	1.84
	2.33
	0.65
	-0.61
	2.81
	0.96
	0.68
	-0.14
	-0.61
	-0.88
	4.97
	-0.37
	-0.72
	0.62
	-1.41
	0.53
	-0.67
	2.1
	-0.24
	0.01
	-0.44
	1.36
	1.63
	1.34
	-0.67
	3.26

1.73
2.03
-0.43
-0.05
-0.3
1.63
1.82
-0.95
-2.02
-0.84
-0.18
-1.8
-1.8
-1.84
-1.97
-1.32
1.16
-2.03
1.63
-0.49
4.07
-0.22
-2.41
-0.04
-1.63
-1.29
0.28
0.89
5.31
4.67
0.4
3.83
1.56
5.71
0.01

	2.16
	2.19
	1.87
	4.26
	4.2
	1.92
	4.6
	1.72
	2.4
	1.05
	1.53
	2.85
	2.4
	0.85
	-0.54
	4.47
	1.5
	2.94
	3.06
	0.92
	3.12
	1.84
	1.9
	2.31
	0.27
	0.99
	-1.02
	1.65
	2.84
	2
	0.69
	-0.83
	0.56
	-0.51
	-0.55

	-1.32
	-0.47
	1.08
	-1.12
	-1.32
	-1.32
	-1.79
	-0.47
	-0.83
	2.75
	2.62
	0.03
	0.91
	2.69
	-1.22
	-1.15
	-0.46
	-1.2
	3.01
	0.33
	2.46
	0.65
	0.72
	0.92
	4.09
	0.49
	1.37
	1.23
	-1.66
	-0.59
	-0.68
	2.29
	1.86
	0.22
	-1.02

	0.54
	-1.88
	1.83
	0.08
	2.63
	-1.04
	1.54
	1.46
	4.99
	2.81
	1.6
	1.23
	-1.43
	4.14
	5.35
	1.19
	0.72
	3.38
	2.99
	0.17
	5.11
	0.82
	-0.67
	-1.31
	5.6
	-0.45
	3.98
	5.57
	-1.76
	-0.83
	3.46
	1.38
	1.84
	2.96
	1.99

	3.18
	0.86
	4.48
	6.46
	3.58
	-1.07
	-0.9
	-0.98
	0.28
	4.72
	0.87
	2.99
	4.48
	1.79
	3.15
	3.23
	4.33
	1.37
	2.29
	4.19
	1.84
	3.97
	2.03
	4.16
	2.44
	2.15
	0.33
	3.35
	1.27
	4.02
	0.49
	3.39
	4.6
	4.58
	4.33

	3.55
	0.63
	0.67
	1
	-1.94
	3.91
	3.1
	2.5
	-0.69
	3.85
	4.84
	4.18
	2.94
	0.49
	2.1
	-0.03
	1.02
	-1.46
	0.24
	1.92
	0.34
	-0.82
	1.18
	4.61
	3.55
	0.35
	1.25
	2.04
	-0.09
	-0.94
	1.27
	-0.86
	1.82
	-0.26
	-0.44

	-1.2
	-1.1
	-0.32
	1.95
	-0.22
	-0.1
	3.95
	0.23
	-1.35
	1.8
	-0.65
	3.21
	-1.48
	0.32
	-0.19
	2.75
	0.93
	-0.1
	1.41
	-0.48
	0
	0.65
	-1.04
	-1.06
	0.64
	-0.66
	0.75
	1.44
	-0.02
	-1.21
	1.12
	3.67
	-0.47
	0.25
	0.55

	-0.52
	-1.23
	0.89
	1.63
	0.49
	-0.82
	2.56
	-1.38
	4.76
	1.36
	-0.74
	-0.49
	1.28
	2.91
	0.68
	0.85
	2.69
	2.51
	0.43
	2.39
	3.85
	4.35
	4.91
	-0.54
	0.08
	2.76
	4.58
	1.5
	-1
	3.92
	-1.22
	0.25
	0.3
	-0.17
	4.42

	-1.45
	0.21
	-0.35
	-2.03
	-0.97
	-1.09
	-0.37
	2.71
	-0.52
	-0.52
	-1.57
	1.01
	-1.8
	1.11
	0.91
	1.92
	4.28
	-0.32
	0.65
	-1.79
	-1.4
	3.03
	-2
	-0.39
	-0.14
	-0.84
	-1.12
	1.43
	-0.18
	-0.52
	2.75
	2.15
	-2.41
	5.07
	-1.76

	1.21
	-1.38
	-1.89
	-0.67
	-1.15
	1.46
	3.14
	0.94
	3.11
	-1.65
	0.38
	-1.52
	-0.35
	-1.87
	3.28
	-1.58
	-0.22
	-1.24
	-2.39
	-0.22
	-1.74
	-1.64
	-1.16
	-1.2
	-1.34
	-2.14
	-1.68
	1.13
	1.74
	0.35
	1.5
	-0.38
	-0.31
	-0.42
	3.03

	-0.55
	1.87
	4.23
	1.25
	2.08
	0.68
	1.34
	0.23
	2.71
	1.37
	0.79
	-2.17
	1.09
	-1.96
	-1.65
	-2.43
	-1.63
	-0.68
	0.01
	0.74
	-0.97
	-2.07
	-1.66
	0.41
	-1.73
	-1.93
	-1.86
	-1.87
	-0.04
	0.25
	-2.41
	-2.39
	1.14
	-1.55
	1.29

	-2.1
	-2.34
	-0.93
	-0.38
	-0.69
	1.31
	-0.2
	0.8
	-0.5
	3.23
	4.4
	2.6
	2.69
	0.31
	-1.09
	3.22
	1.31
	-0.11
	0.09
	0.23
	0.73
	-0.83
	5.99
	2.87
	-1.23
	-0.59
	-1.87
	-1.33
	-0.03
	-0.54
	-0.7
	0.97
	-0.82
	-2.1
	0.86

	-0.23
	2.35
	1.89
	1.8
	2.65
	2.34
	1.51
	0.24
	2.06
	-1.35
	-1.34
	-1.89
	-2.57
	-2.57
	3.51

Log2 Fold Change: (Hs_pos_12022024_Newanalysis) / (Water_pos_12022024_Newanalysis)
3.91
4.44
3.6
6.67
1.93
1.9
-4.04
1.65
2.59
-1.71
0.33
-1.84
1.97
3.47
-1.42
-0.13
0.01
2.74
0.29
6.2
-2.44
1.53
3.82
2.39
4
4.57
5.04
5.83
4.54
-3.49
-3.47
-1.46
-3.49

	0.16
	1.18
	3.67
	2.77
	0.14
	-1.46
	4.66
	-1.79
	-1.06
	2.19
	3.42
	-3.06
	-0.78
	-1.71
	-1.18
	-2.86
	-3.02
	-3.02
	-3.02
	-3.26
	-2.8
	-3.01
	-2.25
	3.75
	-2.81
	2.24
	3.86
	-4.13
	-1.01
	4.44
	5.87
	4.01
	-2.45
	-0.21
	5.04

	4.83
	0.3
	-0.99
	-1.26
	2.52
	5.91
	-0.34
	-2.49
	3.14
	2.21
	2.67
	3.67
	3.39
	0.39
	-2.87
	3.1
	7.24
	-0.45
	-4.07
	3.46
	-0.27
	-0.61
	3.31
	2.9
	0.39
	-0.53
	0.73
	4.48
	2.5
	0.29
	-4.12
	-1.31
	2.86
	-3.78
	2.52

	-2.01
	2.72
	-3.83
	1.53
	0.05
	-1.02
	-4.42
	0.81
	0.89
	-1.83
	2.54
	-3.43
	0.97
	-2.87
	3.72
	3.53
	-2.01
	-1.96
	1.37
	-0.61
	2.53
	-1.04
	1.89
	1.43
	2.08
	0.81
	-1.51
	-2.74
	4.64
	7.46
	4.48
	1.53
	-0.1
	-4.14
	1.69

	-1.6
	2.39
	-3.43
	2.45
	-2.01
	-3.82
	-1.5
	-5
	-0.86
	-2.56
	0.13
	1.5
	5.8
	-0.67
	6.39
	6.32
	-0.4
	4.05
	3.35
	3.41
	4.53
	5.89
	6.85
	5.88
	4.78
	5.88
	5.66
	-1.29
	4.82
	2.83
	6.17
	3.33
	5.47
	5.92
	3.23

2.84
6.87
6.41
6.03
4.84
6.79
3.68
5.78
5.57
5.66
4.64
1.64
8.36
1.51
5.62
2.02
6.76
6.22
4.15
9.38
9.24
7.61
7.38
4.52
7.95
7.13
7.09
6.07
5.58
7.64
5.09
4.91
9.38
7.27
6.34

	-1.87
	5.58
	2.86
	5.3
	5.99
	4.53
	3.34
	3.87
	2.19
	3.83
	4.76
	6.94
	6.36
	3.91
	-0.08
	1.84
	-2.63
	2.16
	5.95
	0.56
	3.24
	0.9
	-4.81
	3.41
	3.86
	-3.19
	-2.65
	2.09
	2.05
	1.3
	3.04
	2.33
	5.6
	2.34
	2.39

	2.19
	5.71
	-0.4
	1.34
	6.42
	6.25
	7.91
	1.23
	5.08
	8.56
	3.82
	3.87
	6.9
	7.21
	5.18
	3.87
	6.38
	5.64
	-0.61
	8.22
	5.58
	3.19
	5.3
	1.33
	3.16
	-2.41
	2.88
	2.75
	3.48
	6.56
	6.9
	7.52
	6.63
	2.07
	8.19

	6.11
	7.76
	8.3
	3.44
	3.97
	5.18
	6.7
	2.71
	4.09
	9.04
	4.87
	5.64
	-0.37
	5.36
	2.64
	-2.55
	8.16
	4.37
	8.81
	3.26
	2.42
	5.14
	2.74
	5.39
	4.17
	3.68
	3.37
	2.24
	1.27
	5.96
	5.88
	8.31
	3.22
	8.6
	7.38

	4.12
	2.66
	5.41
	7.72
	1.39
	8.73
	6.32
	8.32
	3.85
	4.66
	3.46
	4.63
	3.13
	0.83
	-0.62
	5.27
	2.83
	7.53
	6.54
	3.21
	3.67
	5.98
	1.62
	3.3
	1.39
	3.25
	-2.5
	2.3
	1.57
	0.42
	4.49
	3.62
	3.18
	3.42
	1.66

	2.09
	4.36
	-1.72
	-2.46
	3.73
	1.84
	4.42
	4.99
	4.66
	9.02
	5.59
	2.19
	5.56
	2.76
	3.04
	1.11
	3.7
	3.83
	1.2
	4.48
	10.27
	4.79
	3.05
	4.97
	9.53
	0.58
	6.35
	1.45
	2.81
	2.99
	4.86
	-0.54
	6.23
	-0.13
	1.11

	4.35
	-0.24
	4.2
	3.03
	-0.21
	6.45
	6.41
	7.03
	10.01
	2.52
	7.09
	4.82
	3.14
	9.59
	7.28
	6.32
	5.86
	6.92
	7.21
	2.74
	4.46
	2.09
	2.39
	4.38
	4.16
	2.82
	9.16
	5.09
	3.85
	3.85
	6.36
	3.29
	7.45
	6.1
	5.91

	7.27
	3.85
	7.46
	3.91
	3.5
	0.69
	-0.3
	4.62
	3.87
	7.79
	8.31
	7.63
	8.55
	3.05
	4.2
	4.73
	7.24
	7.49
	2.81
	7.82
	7.81
	9.8
	3.5
	4.81
	2.78
	2.11
	6.29
	8.16
	9.55
	2.99
	3.81
	4.24
	7.74
	6.27
	6.44

	7.16
	4.71
	6.03
	2.66
	1.19
	6.94
	8.97
	10.08
	1.01
	8.04
	8.15
	7.35
	5.34
	3.3
	5.68
	7.48
	7.94
	5.15
	4.83
	6.83
	4.38
	5.37
	6.84
	8.86
	7.25
	6.06
	2.71
	3.83
	8.89
	6.49
	10.55
	6.73
	9.45
	7.7
	6.11

	-3.05
	-3.43
	3.03
	7.12
	4.05
	3.49
	-0.72
	0.02
	-2.74
	-3.52
	-2.07
	6.8
	-1.95
	5.47
	4.5
	4.74
	6.04
	2.39
	2.83
	2.68
	5.23
	4.31
	-0.03
	5.12
	2.02
	5.45
	3.23
	3.21
	4.92
	7.42
	4.61
	3.31
	4.46
	0.86
	6.23

	4.3
	0.56
	8.2
	-0.1
	7.08
	1.28
	6.7
	-1.27
	9.59
	6.06
	0.21
	2.68
	1.95
	2.65
	4.43
	2.09
	4.43
	8.77
	6.86
	1.47
	9.56
	9.3
	9.67
	5.67
	5.25
	7.15
	10.33
	6.71
	2.71
	10.24
	1.8
	8.09
	4.43
	2.68
	3.51

0.75
0.42
3.79
-2.82
-4.49
-3.13
6.29
1.68
2.94
-0.91
-4.55
3.42
-5.97
5.29
1.34
4.66
0.71
1.93
3.34
-3.34
-4.99
3.7
-0.09
-5.27
2.78
-0.6
-3.51
-0.23
-0.9
-0.83
1.15
5.4
-2.56
-1.06
-4.43

	-0.06
	-5.33
	-5.69
	-6.12
	-6.76
	5.66
	6.33
	2.64
	5.5
	-1.69
	1.41
	0.82
	4.2
	-0.76
	7.57
	-2.8
	2.21
	-2.49
	-0.69
	4.05
	-1.74
	-7.19
	0.38
	-4.02
	-7.47
	-4.66
	-2.78
	2.06
	2.39
	-3.15
	3.06
	-0.27
	2.07
	-4.05
	5.11

	-0.42
	-3.79
	0.76
	2.36
	4.42
	2.33
	1.63
	0.59
	2.85
	0.16
	-1.51
	-4.32
	1.44
	-4.32
	-4.9
	-0.85
	-3.79
	-2.47
	-3.7
	-2.94
	2.1
	-2.88
	-3.12
	3.82
	-2.85
	-3.36
	-2.7
	-3.55
	1.84
	0.99
	-4.32
	-2.2
	1.43
	-1.34
	2

	-0.14
	-2.84
	-1.22
	3.75
	4.29
	3.74
	0.93
	2.93
	0.21
	2.7
	2.7
	1.69
	2.18
	0.2
	-2.56
	3.35
	2.41
	-4.65
	0.73
	1.24
	-3.03
	1.3
	3.1
	5.65
	-0.05
	5.67
	2.63
	1.18
	4.65
	-1.18
	2.47
	0.19
	-1.94
	-0.53
	5.06

	4.57
	-0.35
	4.14
	8.03
	7.13
	5.46
	5.71
	1.74
	-1.79
	3.99
	3.65
	-6.32
	-5.49
	2.09
	8.24

Log2 Fold Change: (Rabbitserum_12022024_Newanalysis) / (Water_pos_12022024_Newanalysis)
1.48
5.02
5
7.43
3.22
1.89
3.37
2.84
1.32
-0.63
-0.05
3.09
2.91
2.86
-1.62
-0.63
-1.12
6.67
3.13
7.05
0.18
5.8
5.57
6.23
6.69
-0.71
-0.63
-1.86
0.38
-3.01
-2.27
-2.3
-3.19

	0.76
	5.31
	5.75
	4.41
	-1.21
	-0.95
	4.13
	-0.88
	0.64
	2.18
	2.97
	-2.26
	4.72
	4.81
	4.84
	3.88
	2.23
	4.31
	3.1
	5.03
	3.1
	-0.14
	-1.25
	2.44
	-1.34
	2.01
	3.29
	-0.58
	-0.13
	2.81
	6.11
	2.14
	-0.81
	-0.02
	3.68

	2.65
	0.28
	-1.82
	-2.05
	2.7
	3.77
	1.6
	-0.05
	6.01
	4.71
	4.5
	5.11
	2.55
	-1.5
	-2.32
	2.25
	5.38
	-0.37
	-3.2
	2.47
	0.75
	-0.79
	3.16
	4.38
	3.17
	3.7
	3.15
	2.2
	2.94
	2.29
	-1.82
	0.19
	5.26
	-3.8
	2.14

-2.99
2.82
-2.77
1.34
-0.22
0.41
-2.89
1.39
4.01
-0.96
2.9
-2.41
0.77
-1.53
6.23
4.32
-0.71
-1.41
5.71
0.48
-1.21
-0.71
2.49
2.03
0.43
0.54
-2.21
-0.58
4.02
7.86
3.48
2.41
-0.22
0.28
2.2

	-1.06
	0.72
	-0.16
	-0.25
	-2.34
	-2.26
	-1.76
	-2.12
	-1.21
	-0.23
	-0.85
	3.52
	6.14
	0.94
	3.76
	4.72
	-0.99
	1.22
	0.91
	1.17
	1.2
	3.02
	4.48
	4.03
	1.41
	3.15
	3.67
	-1
	1.66
	2.98
	6.68
	0.85
	4.48
	2.77
	6.06

	3.01
	5.86
	3.45
	7.14
	5.02
	4.72
	4.71
	2.51
	4.18
	6.1
	4.07
	-1.17
	6.28
	5.36
	0.11
	5.93
	2.77
	2.16
	5.73
	3.05
	3.77
	3.12
	2.53
	-1.22
	0.49
	0.74
	2.58
	0.46
	1.83
	5.79
	2.6
	2.42
	6.08
	7.05
	8.11

1.83
1.08
0.36
-0.57
0.74
-0.61
1.67
2.87
0.24
2.66
-0.88
-0.28
1.97
1.56
-0.05
0.52
2.1
3.72
2.78
-0.87
3.94
1.44
-3.47
2.97
3.82
3.87
-1.01
-0.52
-0.47
-0.43
1.86
-0.47
1.45
0.28
3.52

	2.65
	3.4
	-0.5
	0.71
	2.59
	2.54
	4.49
	-0.64
	1.03
	6.05
	4.03
	2.55
	2.57
	5.11
	3.56
	4.14
	1.52
	1.87
	-1.67
	7.04
	2.31
	2.59
	2.68
	0.49
	1.77
	-0.4
	3.4
	1.05
	2.99
	2.55
	2.84
	3.75
	0.51
	0.92
	6.34

	5.2
	5.27
	1.38
	5.19
	2.96
	3.2
	5.22
	-0.5
	-1.27
	3.2
	1
	1.47
	-2.13
	-0.39
	-2.17
	-0.42
	5.76
	0.75
	1.42
	1.84
	4.9
	4.3
	-1.72
	5.02
	2.63
	2.6
	3.24
	3.48
	3.81
	7.22
	2.87
	6.39
	3.46
	7.27
	0.8

	5.89
	3.06
	3.45
	7.71
	2.05
	3.66
	6.66
	5.9
	3.32
	4.26
	2.84
	1.27
	3.08
	1.38
	-0.19
	6.73
	4.05
	5.29
	5.58
	5.74
	2.97
	5
	2.22
	6.14
	3.5
	0.96
	-1.97
	4.64
	1.53
	5.58
	5.47
	2.31
	4.16
	1.52
	0.89

	0.31
	0.45
	-0.84
	-1.47
	2.91
	-0.47
	1.6
	3.82
	1.89
	5.02
	5.93
	3.25
	5.04
	2.67
	-0.45
	-0.01
	1.21
	1.75
	5.31
	3.74
	5.24
	4.65
	-0.96
	3.77
	7.29
	0.63
	3.79
	3.66
	1.63
	2.94
	2.12
	5.43
	4.68
	0.84
	0.22

	3.37
	-0.79
	4.33
	2.82
	4.24
	2.69
	3.94
	5.23
	8
	3.97
	5.15
	6.94
	2.53
	7.63
	8.5
	1.93
	2.32
	5.06
	5.57
	1.58
	4.52
	1.84
	1.03
	3.12
	5.27
	0.34
	6.96
	7.09
	-2.28
	1.11
	6.83
	0.27
	4.71
	4.82
	4.4

	6.27
	3
	7.29
	8.09
	5.96
	0.58
	-0.07
	0.59
	4.43
	7.8
	2.69
	5.87
	7.97
	2.21
	6.74
	5.9
	7.17
	2.8
	3.91
	6.98
	3.48
	7.6
	4.46
	5.47
	4.81
	3.68
	4.69
	6.74
	1.7
	4.6
	3.72
	5.28
	8.3
	8.07
	7.84

	6.62
	3.52
	5.08
	0.68
	-1.12
	5.29
	5.68
	5.28
	1.92
	8.45
	7.98
	6.31
	6.32
	1.77
	5.46
	0.74
	3.85
	-0.81
	1.09
	4.53
	3
	2.68
	4.75
	8.38
	7.14
	3.19
	3.65
	5.52
	-0.15
	0.45
	3.81
	1.29
	4.96
	0.11
	-0.74

	-1.82
	-1.51
	-1.06
	4.8
	2.79
	5.42
	1
	0.74
	-3.38
	-1.79
	-2.53
	7.22
	3.97
	3.26
	2.57
	4.58
	3.87
	2.84
	2.66
	1.53
	3.38
	2.51
	0.22
	-0.75
	2.94
	4.07
	2.98
	5.64
	3.58
	1.38
	3.46
	5.64
	5.05
	-0.09
	4.56

	3.78
	1.85
	5.04
	-0.47
	6.46
	1.35
	5.98
	-0.22
	8.83
	6.72
	2.19
	3.43
	4.48
	0.92
	4.78
	1.62
	5.74
	7.26
	5.26
	1.46
	7.21
	8.79
	8.95
	4.04
	4.57
	5.5
	8.89
	3.69
	2.28
	7.44
	2.06
	1.96
	-1.41
	-0.08
	4.1

	3.52
	0.93
	4.62
	-2.87
	-3.79
	-1.86
	-0.25
	4.18
	2.36
	-0.12
	-2.91
	1.17
	-4.94
	4.22
	2.71
	3.68
	4.37
	-0.55
	1.6
	-2.36
	-2.7
	4.36
	-0.04
	-3.94
	1.07
	-0.49
	-2.68
	-0.73
	-1.15
	0.5
	2.39
	4.48
	0.41
	4.44
	-0.98

-1.18
-1.7
-2.95
-3.82
-2.28
3.67
6.17
3.7
6.4
-1.08
2.05
1.21
3.26
-0.35
6.38
-4.66
1.64
-0.94
-2.25
2.79
-2.16
-5.01
0.35
-2.71
-3.26
-2.63
-2.24
4.36
4.16
1.13
4.37
1.21
2.58
-2.34
6.13

	0.54
	1.83
	6.11
	2.72
	2.98
	1.37
	2.23
	3.59
	3.96
	2.52
	2.06
	-3.8
	1.64
	-1.8
	-3.59
	-1.56
	-2.32
	-1.78
	-2.08
	-1.07
	-1.27
	-2.38
	-2.24
	3.05
	-2.17
	-2.68
	-2.16
	-2.5
	0.86
	-0.36
	-3.97
	-3
	2.03
	1.38
	2.39

	-0.98
	-2.44
	-0.33
	3.03
	2.5
	5.13
	1.37
	3.48
	1.55
	4.95
	6.16
	4.41
	5.2
	2.75
	1.64
	7.03
	4.58
	-1
	-0.04
	0.79
	0.11
	0.73
	3.77
	3.02
	0.51
	2.04
	1.75
	-1.83
	2.67
	-0.52
	3.24
	2.69
	-1.23
	-2.03
	3.3

	1.22
	1.65
	0.61
	1.56
	3.37
	5.23
	3.51
	-0.26
	1.3
	-0.23
	-1.82
	-2.22
	-1.7
	-2.39
	10.24

Log2 Fold Change: (SM_pos_12022024_Newanalyis) / (Water_pos_12022024_Newanalysis)
0.35
1.86
0.41
5.02
-0.87
0.79
1.83
0.27
2.05
2.01
2.62
3.36
1.76
4.39
0.2
0.15
0.63
0.68
-2.5
1.21
-0.88
0.8
2.48
0.46
1.35
1.37
-0.47
-0.66
-1.24
2.24
-1.41
-2.32
2.24

	4.38
	2.79
	1.31
	-1.39
	-1.29
	-2.53
	-1.9
	3.45
	0.46
	2.71
	2.13
	2.01
	0.92
	1.19
	-0.42
	-1.76
	0.26
	-1.66
	0.26
	0.87
	0.11
	-2.97
	1.25
	1.99
	1.12
	0.94
	-0.38
	0.28
	3.9
	-0.39
	2.99
	-1.56
	2.01
	-0.47
	9.44

	3.26
	0.42
	0.85
	3.17
	2.52
	9.25
	1.79
	-1.84
	2.5
	0.85
	-2.24
	-4.31
	-2.22
	1.57
	1.57
	2.63
	1.66
	1.31
	-2.02
	-2.67
	3.9
	-1.47
	-7.15
	-2.2
	0.92
	1.43
	-0.19
	0.91
	-0.97
	0.29
	-3.69
	-0.91
	0.96
	0.77
	4.99

	-1.8
	-0.63
	0.92
	2.06
	1.13
	-0.62
	-0.26
	2.86
	0.02
	-2.14
	4.06
	1.3
	1.28
	1.63
	-0.74
	-2.67
	-3.05
	2.17
	0.11
	-2.93
	-1.34
	2.31
	0.44
	1.83
	-3.25
	0.93
	-1.75
	-1.73
	1.53
	-1.99
	-0.03
	-2.43
	-1.4
	-1.56
	2.29

	2.82
	0.55
	-1.77
	0.96
	-1.87
	-1.97
	-1.87
	-1.06
	-1.07
	-0.84
	-0.16
	1.4
	1.98
	-1.75
	0.09
	2.41
	2.36
	1.35
	0.01
	-0.15
	1.28
	1.15
	0.53
	1.35
	4.44
	-5.35
	-1.51
	-0.93
	0.95
	-0.27
	3.91
	0.31
	-1.51
	3.04
	3.49

	3.83
	-0.35
	-2.31
	3.16
	2.19
	0.41
	3.99
	1
	5.61
	4.06
	-1.31
	1.86
	2.36
	3.75
	0.75
	4.28
	0.57
	4.97
	3.66
	0.17
	1.77
	1.11
	0.28
	0.59
	-0.78
	-0.27
	-1.52
	3.57
	0.13
	-4.35
	3.59
	-3.5
	3.28
	-0.17
	9.57

	0.29
	0.86
	1.03
	0.92
	0.35
	4.85
	4.79
	2.56
	3.23
	3.35
	-0.46
	0.33
	2.18
	2.53
	-0.45
	-1.32
	-1.54
	-2.63
	0.2
	-2.98
	2.4
	-1.6
	-3.85
	3.32
	1.01
	-2.6
	1.12
	-1.81
	-0.99
	-3.17
	0.86
	0.65
	-0.26
	-1.24
	-0.2

	2.23
	1.96
	0.26
	0.47
	-1.33
	2.13
	2.45
	-0.36
	1.28
	3.27
	2.71
	1.78
	-0.41
	2.76
	3.12
	3.02
	-2.11
	2.13
	-0.2
	-0.16
	1.06
	5.64
	-0.19
	4.58
	-2.48
	0.01
	1.88
	0.4
	2.67
	1.04
	2.44
	0.62
	0.85
	-0.14
	4.01

3.13
-0.49
0.59
4.2
5.91
2
1.97
-0.6
1
-0.09
2.51
-1.71
-1.79
0.37
0.79
1.23
-1.02
-1.69
2.3
-0.35
0.24
-1.27
-0.35
1.21
-1.12
-1.38
0.38
0.5
0.84
5
-1.77
3.96
3.28
10.19
0.6

	3.24
	0.5
	0.7
	0.8
	4.26
	1.66
	5.22
	1.22
	8.86
	2.18
	2.39
	3.38
	4.12
	2.05
	4.08
	4.7
	1.86
	0.91
	1.4
	2.69
	5.8
	3.64
	3.05
	1.41
	0.53
	4.68
	1.01
	1.27
	2.5
	2.81
	2.86
	1.28
	2.04
	1.38
	0.09

	-1.77
	-1.56
	-1.18
	1.71
	-1.13
	-0.82
	-1.76
	0.65
	-0.5
	3.11
	2.33
	0.41
	1.91
	0.08
	0.35
	-5.56
	-0.81
	-1.25
	3.31
	1.27
	2.58
	2.25
	1.71
	-0.17
	4.4
	1
	1.33
	0.16
	-3.21
	2.31
	-0.35
	-1.4
	0.41
	-2.65
	3.78

	3.31
	2.77
	0.34
	0.46
	-0.9
	-0.95
	0.65
	1.76
	1.18
	0.83
	2.39
	1.29
	-1.6
	1.18
	7.71
	-0.43
	-0.6
	3.51
	2.41
	2.6
	5.74
	2.05
	-1.66
	-0.59
	6.21
	0.96
	-3.7
	9.57
	-2.01
	-2.03
	-0.58
	0.91
	3.3
	3.88
	6.42

	3.14
	6.63
	7.67
	8.95
	8.96
	1
	5.14
	-1.51
	1.14
	4.04
	0.4
	3.29
	4.43
	4.07
	4.46
	6.17
	1.11
	-0.39
	4.05
	6.46
	1.04
	4.58
	3.06
	5.94
	6.27
	6.37
	2.54
	-0.42
	1.46
	-1.53
	2.64
	3.72
	6.03
	3.93
	5.06

-1.09
-1.95
1.59
2.99
0.42
4.72
4.86
5.13
-1.14
4.9
6.66
9.35
2.29
3.58
2.8
0.9
1.56
1.93
0.38
1.87
-1.54
2.18
0.62
4.85
3.88
0.63
4.5
1.84
-1.34
-0.59
0.82
-1.28
2.15
0.08
1.32

	0.07
	-3.39
	0.68
	2.24
	1.77
	2.63
	3.49
	-1.68
	0.82
	1.42
	0.74
	5.13
	-0.84
	4.5
	0.87
	2.1
	1.89
	2.57
	-0.64
	3.39
	2.5
	4.69
	5.31
	-0.95
	2.16
	0.18
	2.7
	-1.33
	0.27
	-1.31
	2.61
	2.7
	0.92
	-1.04
	1.6

4.81
1.95
4.59
1.1
8.65
-1.51
1.7
-0.2
1.22
3.36
0.93
1.91
3.36
-0.2
0.54
2.08
3.61
9.45
2.62
2.53
5.62
-1.29
12.16
0.28
6.62
2.06
1.31
4.24
6.81
9.2
0.99
-0.59
0.41
-1.41
-0.02

	-1.66
	-0.45
	1.28
	-2.97
	-2.23
	-2.05
	0.96
	3.33
	0.44
	2.54
	-3.74
	0.16
	-6.96
	0.22
	1.55
	1.4
	-1.39
	1.51
	2.38
	-1.61
	-5.8
	1.01
	0.76
	-0.09
	-0.64
	-0.88
	2.43
	-0.18
	0.17
	4.25
	1.72
	3.64
	-1.95
	4.97
	0.82

	2.14
	-2.55
	-1.69
	-2.5
	-4.13
	0.73
	0.87
	0.09
	3.2
	4.03
	-1.37
	-0.96
	-1.41
	0.3
	2.86
	-2.22
	0.05
	-2.16
	0.86
	1.77
	-1.67
	-2.78
	0.05
	1.15
	0.61
	-2.66
	1.64
	-0.02
	3.59
	1.26
	0.57
	1.34
	-0.54
	2.29
	2.35

	3.76
	-3.26
	-0.63
	2
	-5.26
	3.51
	2.89
	7.4
	4.89
	0.26
	-3.54
	-3.89
	0.67
	-4.38
	0.25
	0.77
	-3.26
	-0.41
	-3.13
	-0.48
	-3.19
	-2.02
	-1.99
	1.71
	-1.66
	-1.92
	-1.45
	1.19
	1.28
	2.78
	-0.65
	-0.95
	1.15
	-4.38
	1.93

1.44
-1.91
6.09
2.38
-1.31
0.61
0.93
-3.32
1.11
1.98
-3.19
-3.48
-1.29
-1.58
-2.04
4.49
0.3
1.54
1.93
-0.51
-1.12
0.06
1.05
0.14
-2.05
0.35
-2.53
-1.37
3.25
1.19
-3.79
1.37
-1.15
-0.5
1.84

	-0.26
	2.48
	0.01
	2.81
	0.87
	1.95
	0
	0.5
	3.21
	0.13
	-1.29
	-0.36
	-2.3
	-1.79
	12.8

P-value: (Bb_pos_12022024_Newanalysis) / (CM_pos_12022024_Newanalysis)
0.870987968
0.673670258
0.999998164
0.009318927
0.96212471
0.994816111
0.849455964
0.004531536
0.943333222
0.989866628
0.988272649
0.855251059
0.419125466
0.563841336
0.999989085
0.156986726
0.772913959
4.71845E-14
0.485065133
0.000709477
0.607548181
3.67363E-11
6.40848E-05
4.70179E-13
4.60743E-14
0.884564236
0.984889361
0.979086464
0.188841878
0.996201791
0.997488276
2.96199E-08
0.429894226

0.507478462
2.42362E-13
0.101088564
3.58497E-05
0.000667007
0.00032676
1.03781E-08
0.998049356
0.627619596
0.001879044
0.032503894
0.992503921
1.23283E-10
1.43879E-08
4.00463E-08
0.002318725
0.000246192
0.00022244
3.71578E-06
1.46271E-10
0.000266485
0.851270356
0.639220761
0.006793918
0.637707033
0.913801392
0.040300262
0.828251964
0.999918356
0.207105717
1.11378E-08
0.000810236
0.998490004
6.44007E-12
0.565078953

	0.833279451
	0.784408921
	4.99158E-07
	0.995339239
	0.000407144
	0.045137496
	0.9981661
	0.545321157
	7.78266E-14
	0.984647755
	5.74619E-11
	4.94946E-08
	5.3242E-05
	3.12972E-13
	0.999974478
	4.77282E-05
	0.089385422
	0.027773786
	0.976396797
	1.38698E-07
	0.056155692
	0.105133494
	1.46044E-05
	4.76214E-09
	8.80987E-06
	1.33435E-05
	0.000104856
	0.020783888
	2.78496E-09
	0.00012872
	0.999977404
	0.25503844
	0.028820526
	0.541969219
	0.000405695

0.994536567
0.00381531
0.957445303
0.124058537
0.998019743
0.931528917
0.99999999
0.06474324
0.696928917
4.64774E-05
0.035671212
0.99333678
0.269585443
0.384683842
4.60743E-14
0.000638573
0.947331917
0.972071404
0.999998123
0.999707728
0.186245904
0.992988703
0.998679763
0.127250904
0.9441896
0.999936783
0.999755914
0.968553793
0.181303446
8.31944E-09
0.01009382
0.004971323
0.004864235
0.002554648
0.985304371

	0.999939715
	0.744779982
	0.489161973
	0.926041137
	0.996303722
	0.999976276
	0.864037057
	0.963938423
	0.001782239
	0.999999588
	0.436379439
	0.990828228
	5.64869E-07
	0.655017869
	0.4884298
	9.14293E-09
	0.999999606
	0.677642686
	0.063393824
	0.581223728
	0.477129378
	0.433294823
	0.337714634
	1.94356E-05
	0.999999998
	0.000633301
	0.200577542
	0.56711836
	0.641805252
	0.961309087
	0.180235498
	0.151694086
	0.2109265
	0.799896374
	0.075512229

	0.048259076
	0.7876729
	0.001388471
	3.2824E-06
	0.000279122
	0.538364888
	0.061517287
	0.948463169
	0.031067541
	0.844505259
	0.316864092
	0.82122231
	5.72168E-11
	0.000670394
	0.239492694
	0.363416123
	0.997976184
	0.146659298
	1.68421E-13
	0.004614732
	0.021301741
	0.007487853
	0.118990061
	0.843810523
	0.041234387
	0.991992728
	0.124969316
	0.367072781
	0.000954265
	9.46315E-05
	5.01638E-05
	0.003953654
	0.020326828
	0.194271454
	0.14698541

	7.86702E-06
	0.998972467
	0.65780379
	0.596947818
	0.227129397
	0.999976634
	0.515323112
	0.999904348
	0.998945549
	0.726352584
	0.233623729
	0.627812524
	0.132337498
	0.994637061
	0.026512218
	0.000428371
	9.25548E-05
	0.014686535
	2.51365E-05
	0.986710556
	0.003349943
	0.911160902
	0.981106035
	0.725926129
	3.97735E-05
	0.089508101
	0.999999994
	0.989322188
	0.050812752
	1
	0.728139945
	0.972829654
	0.541532707
	0.998534732
	8.37117E-07

0.127760199
0.999882891
0.99999719
0.863564262
0.999311173
0.009435034
0.119884029
0.999999924
0.524582566
1.76528E-07
0.011246097
0.773969961
0.829197582
0.003205183
5.0248E-05
0.005383935
0.000737979
0.003856581
0.999153539
6.90004E-13
0.471483691
0.007499311
0.325467992
0.174201535
0.999718452
0.989028051
0.782311375
0.565945662
0.013021094
0.977981907
0.131381446
4.50267E-08
0.241674566
0.993156357
8.75855E-13

	0.768823688
	2.96073E-07
	0.12706962
	0.969065697
	0.999994735
	0.717751782
	9.92021E-06
	0.999901693
	0.764750418
	0.975777935
	0.025763191
	0.987990757
	0.984067206
	0.070561144
	0.920595527
	0.868625777
	0.001335885
	0.90686434
	0.000789874
	0.999991291
	4.07082E-10
	6.04893E-05
	0.999993269
	0.025266074
	0.998724427
	0.999999999
	0.880460797
	6.0416E-06
	0.000530669
	5.07156E-05
	0.18320368
	0.01334276
	0.00689299
	0.086588566
	0.099380601

	0.034338204
	0.62752221
	3.56189E-05
	0.999934531
	0.984365474
	0.817093076
	0.013709214
	2.08487E-07
	0.999756955
	0.001889467
	0.427594242
	0.518154644
	0.006202138
	0.005528228
	0.261423746
	0.992402498
	0.999963112
	6.19632E-06
	1.03464E-08
	0.001726381
	0.040609746
	0.004728126
	0.225622194
	1.33107E-05
	0.200554495
	0.346742372
	0.052895714
	8.19632E-08
	1.37658E-09
	1.54869E-05
	7.77349E-05
	0.206499679
	2.73358E-05
	3.74112E-12
	0.000271288

	9.83853E-08
	0.999999814
	1.91291E-09
	1.60659E-06
	0.999886817
	0.999526196
	0.983708366
	0.443579617
	0.999564111
	0.021692333
	0.001082534
	0.303882037
	0.999986378
	0.005071336
	0.999933989
	0.052921794
	0.009600308
	0.994038917
	0.012219634
	0.402622421
	3.8024E-12
	0.025439351
	0.759451533
	0.006266264
	1.63539E-05
	0.99596561
	0.156519549
	0.18966754
	0.773625313
	0.025150262
	0.995663219
	0.502428345
	0.933036933
	6.27716E-05
	0.988278012

	0.965688214
	0.985725443
	0.14507869
	0.998475266
	0.489257551
	0.999999968
	0.599629076
	0.961413906
	0.01819431
	0.069067457
	0.967275284
	0.995382107
	0.999991902
	0.006574016
	0.008550453
	0.030225244
	0.023540306
	2.22807E-08
	5.09827E-08
	0.118889413
	0.106704731
	0.005401275
	0.959618364
	0.999135061
	0.114591842
	0.755907829
	0.847452406
	0.014689759
	0.994440689
	0.165564545
	1.2064E-07
	6.80611E-10
	3.46694E-08
	4.13102E-05
	0.084539598

	0.001770335
	0.124702316
	0.999917878
	0.036256954
	0.944767257
	0.038791027
	8.11533E-05
	0.9943873
	0.983573523
	0.971238217
	0.997016219
	4.12117E-05
	3.88175E-06
	1.65609E-05
	0.289624467
	0.971632743
	0.008906016
	0.884374353
	9.75505E-06
	1.30673E-13
	0.85494871
	1.27528E-09
	0.002435459
	0.00017248
	0.998815803
	1.12855E-06
	0.999999992
	2.88857E-06
	0.186212706
	0.253500203
	0.123310585
	0.999853028
	0.105471806
	1.75984E-06
	6.54888E-08

	6.24589E-06
	0.181317001
	0.305062935
	0.274311495
	0.104430002
	0.987138964
	1.28978E-05
	0.013186514
	0.796778952
	0.025771901
	0.999999996
	0.005469365
	5.4512E-14
	1.25111E-07
	1.00203E-06
	4.60743E-14
	4.60743E-14
	6.16174E-14
	0.473711297
	0.9997656
	0.998393621
	0.999929812
	0.004872987
	0.260918936
	6.82626E-07
	7.27196E-14
	1.6727E-10
	0.973087979
	0.736680436
	0.99997877
	0.652330355
	4.82947E-14
	3.7048E-09
	4.60743E-14
	0.000465397

	0.99882651
	0.000272472
	0.416532675
	0.645650238
	2.85521E-05
	0.999831389
	0.691464842
	0.792367993
	0.810987879
	0.399223284
	0.999889685
	0.999556711
	0.99999999
	0.991236986
	0.943294748
	3.02796E-05
	7.78559E-09
	0.24267812
	2.3288E-05
	0.995957275
	0.35336586
	0.913191155
	0.983599876
	0.908431664
	0.9655201
	0.97161148
	0.389084396
	0.985021911
	0.986448329
	0.998849675
	0.007328597
	0.00062727
	0.999758144
	0.000748616
	0.023229999

0.582626352
0.944021469
0.999999243
0.121706918
0.013304093
0.999996536
0.005395651
0.766101141
2.26519E-12
0.000317901
0.983698907
0.855544348
0.704302606
0.803201547
0.999999997
0.001440086
0.445547711
7.27396E-05
0.288788754
0.184573836
4.28723E-08
1.01828E-06
1.13079E-07
0.984019754
0.799762496
0.999910662
9.72185E-08
0.979283395
0.996794958
2.87228E-06
0.963703213
0.505009274
0.034008677
5.09925E-13
0.928968763

	4.60743E-14
	0.997619728
	9.08748E-11
	0.152180177
	0.635543825
	0.502652873
	0.44369533
	0.625197563
	0.972331024
	0.347524188
	0.999982883
	0.667733005
	0.631126572
	0.009679894
	0.34819619
	0.012226774
	0.935649835
	0.001606209
	0.058824469
	0.997732105
	0.063522776
	0.000376236
	0.470102153
	0.992039145
	1.25808E-07
	1.618E-07
	0.290395778
	0.720470975
	4.10984E-05
	0.999676879
	0.651221541
	0.954741784
	0.999276254
	0.999953465
	0.984008866

0.611713031
0.999999998
0.994219489
0.994339915
0.999988933
0.302450192
1.55542E-13
0.000834711
0.00047366
0.132706947
0.847900348
8.82218E-11
0.010825114
0.19305682
3.08342E-12
0.074117199
4.59641E-10
0.997656487
0.002083494
2.9135E-09
0.99992009
0.43901143
0.999078506
0.998985324
0.968240492
0.968016788
0.196732435
0.000293172
0.435648272
0.999999921
7.1896E-06
0.918273113
0.999807671
0.998348433
0.999724806

0.932832883
3.2362E-08
1.97844E-08
0.001184974
2.88924E-07
7.25452E-06
2.32455E-05
9.77645E-08
2.46912E-08
0.005176715
0.51314465
0.989813136
0.06429772
0.996843245
0.907205051
0.99999869
0.999025886
0.103027812
0.62209998
0.825045718
0.760222076
0.999310593
0.999857215
0.999996542
0.999982201
0.989043732
0.999735276
0.995210644
0.918312011
0.934855313
1
0.99962959
0.0096281
0.170278694
0.015592794

	0.999818636
	0.993996684
	0.999201862
	0.999963697
	0.575349968
	0.989145892
	0.601119525
	3.89142E-07
	0.655459395
	5.49255E-07
	7.56062E-14
	2.36004E-10
	2.88961E-08
	0.575141647
	0.999999818
	0.93634668
	0.987706892
	0.963298241
	0.999987726
	0.999858153
	0.027019487
	0.453164461
	0.007212798
	0.999482578
	0.861732565
	0.689565004
	1.6287E-13
	0.040995685
	0.999999662
	0.916809271
	0.932850878
	0.22027696
	0.230597758
	0.983709616
	0.06110457

0.6718452
0.814088817
0.008603546
0.872452035
0.598366185
5.50105E-05
0.004344165
0.969411432
0.001605771
0.771655252
0.999823199
0.660516025
0.417533447
0.007870032
0.032790251

P-value: (Bb_pos_12022024_Newanalysis) / (Rabbitserum_12022024_Newanalysis)
0.065877671
0.981996314
3.6749E-09
8.25374E-05
0.390766477
0.819548848
0.005111945
6.51486E-06
0.012759474
0.392537089
0.999596808
0.078165869
0.003064989
0.311945226
0.092522974
0.001234768
0.695671648
4.60743E-14
9.07714E-07
1.54732E-12
0.991914434
5.05151E-14
7.25959E-06
4.60743E-14
4.60743E-14
0.998770214
0.957793863
0.66611357
0.006219958
0.994522885
0.312977617
1.21808E-07
0.027679794

	0.054004405
	4.65183E-14
	3.83828E-07
	1.63319E-11
	0.001978994
	0.002046197
	1.05961E-10
	0.968584303
	8.36808E-05
	0.000141834
	9.53972E-05
	0.535972259
	1.06026E-13
	1.36583E-11
	5.44081E-11
	3.71761E-05
	3.46942E-06
	1.02108E-05
	1.9347E-07
	8.51874E-13
	2.94949E-07
	0.960640088
	0.386715717
	1.19951E-06
	0.406984781
	0.00211238
	0.429674431
	0.050850271
	0.999375872
	0.053437149
	7.34758E-11
	1.04863E-05
	0.999431119
	7.93829E-09
	0.007785186

	1.19541E-05
	0.074602221
	0.000290924
	0.999999709
	8.78223E-07
	5.90919E-05
	4.37044E-05
	0.713951784
	4.60743E-14
	0.000194327
	6.31717E-14
	4.62963E-14
	1.20343E-07
	6.37143E-09
	0.343299528
	7.59676E-08
	1.47293E-12
	0.01121857
	0.253669664
	2.08158E-06
	0.034391365
	0.001982084
	7.41403E-07
	9.37139E-13
	4.72313E-10
	6.79444E-09
	2.93656E-09
	5.94175E-06
	1.60937E-11
	2.89081E-08
	0.960922367
	0.268828447
	1.98313E-10
	0.839689867
	5.97357E-06

	0.837644143
	1.48566E-06
	0.013303322
	0.018305777
	0.698238546
	0.537738045
	0.744651081
	0.001437204
	5.56669E-06
	0.0214066
	0.000355967
	0.795808753
	2.11125E-05
	0.86236355
	4.60743E-14
	9.39125E-05
	0.93049689
	0.999954158
	4.60743E-14
	0.05404105
	0.000116537
	0.205855729
	0.613068034
	0.014905083
	0.009134146
	0.0002723
	0.79422474
	0.99451725
	1.61051E-06
	5.54001E-14
	1.4246E-08
	0.269487135
	0.017000947
	1.35668E-05
	0.264272463

	0.983765899
	0.312702508
	0.018287936
	0.935705905
	0.788661131
	0.598316634
	0.71144142
	0.991265333
	7.35302E-06
	0.511148622
	0.415752668
	1.77895E-06
	4.60743E-14
	0.026628649
	1.55098E-13
	4.60743E-14
	0.998131139
	0.000159245
	1.86979E-05
	0.003137654
	8.01636E-06
	6.05254E-09
	5.4956E-14
	2.61503E-10
	0.132693425
	8.03789E-06
	0.957515767
	0.334790977
	0.000982072
	0.225451653
	1.34052E-07
	0.003645925
	0.298945252
	0.001501693
	1.56195E-08

	0.9985602
	7.66911E-07
	6.77377E-07
	4.60743E-14
	7.93506E-09
	4.93142E-07
	0.000655213
	9.05888E-09
	3.09977E-05
	2.81069E-08
	0.131086051
	0.99999922
	4.60743E-14
	2.3576E-07
	0.000662184
	6.30266E-09
	3.28105E-07
	2.78437E-05
	4.60743E-14
	8.32612E-12
	5.14893E-08
	1.8613E-10
	0.989457943
	0.999999709
	0.000424621
	0.243361755
	0.007427755
	0.364105669
	6.67405E-07
	1.38814E-09
	4.17348E-08
	9.04214E-06
	2.14865E-08
	7.17496E-07
	8.22197E-06

1.34959E-07
0.000729788
0.001629137
0.778130494
0.030444447
0.431376765
0.000328695
0.00171335
0.350880135
0.93017753
0.000295109
0.541224822
0.001066147
0.999999998
0.051589495
0.000564065
0.001877474
2.15383E-10
5.91764E-09
0.99999981
6.04191E-09
0.087356798
0.996346284
5.49645E-07
4.71845E-14
4.19884E-07
0.999640794
0.480159677
0.007841664
0.979086413
0.229474672
0.953593386
0.058130734
0.114027946
5.9841E-14

0.996412188
0.047032509
1
0.968808886
0.08854588
1.34018E-10
1.20008E-07
0.899370142
0.002545663
4.65183E-14
0.98538981
0.301425895
0.095179033
9.28924E-13
0.20089004
0.313540784
0.999995586
0.954869935
0.999533383
4.60743E-14
1.23159E-06
0.316590111
0.550539374
0.736248225
0.603519754
1
0.011513511
0.99079808
0.080372866
0.008449061
9.99777E-07
2.32248E-12
0.004480363
0.923478963
4.60743E-14

	8.75741E-11
	4.02678E-13
	9.40609E-08
	0.121296597
	0.001375374
	0.02828652
	5.73985E-14
	0.799545258
	0.181763806
	4.55744E-09
	0.969664114
	4.17744E-05
	0.990643298
	1.15688E-06
	0.965077304
	0.698775301
	6.03884E-12
	0.000587734
	2.47625E-05
	0.001596835
	8.26933E-11
	0.001293065
	0.913192391
	4.76286E-14
	2.63339E-05
	9.43996E-08
	0.001660835
	5.25135E-14
	0.671376878
	3.72468E-11
	8.58219E-05
	1.26342E-06
	0.630469944
	0.001252932
	0.001880991

	1.51335E-12
	0.988649787
	5.12456E-09
	0.008489569
	0.002813478
	0.999953111
	1.75634E-07
	4.62963E-14
	0.877111375
	3.02991E-12
	0.020322715
	0.000198974
	0.000110703
	0.00040381
	0.997614521
	0.980963919
	1.10699E-05
	1.28674E-10
	5.00711E-14
	1.81388E-05
	0.006479179
	0.979882942
	0.002671642
	1.1503E-12
	8.7582E-07
	0.137196161
	0.00035214
	0.288315289
	0.000752567
	0.103102048
	0.708652714
	3.15545E-05
	0.730337499
	1.07542E-08
	0.012830296

	0.00049612
	0.489122493
	1.00486E-12
	2.00883E-07
	0.000222942
	0.357135333
	5.7216E-06
	5.90206E-12
	2.92944E-05
	7.71915E-09
	0.251358203
	2.318E-11
	2.17904E-07
	0.001635096
	0.936147625
	0.000665147
	0.999977749
	1.66458E-05
	0.000215406
	0.000158719
	4.60743E-14
	3.02723E-11
	0.871327968
	2.88617E-10
	2.82901E-11
	0.949501453
	0.001709987
	5.21672E-06
	0.000124801
	2.08474E-08
	0.344677552
	0.000122925
	9.17726E-07
	5.65753E-06
	0.699805466

	0.244091268
	0.15921725
	5.72213E-05
	1.32366E-08
	0.021788613
	4.14859E-08
	6.83596E-09
	2.27195E-07
	2.5676E-09
	0.999219842
	0.000138573
	1.41703E-11
	1.12022E-07
	1.43751E-06
	8.22433E-08
	3.1156E-05
	2.30888E-05
	2.24631E-12
	1.23346E-13
	0.00130325
	0.000826732
	5.51591E-07
	0.000110892
	6.56311E-10
	0.767476598
	0.996133696
	0.518532024
	0.00089353
	0.917008985
	0.004141089
	3.90465E-13
	4.96159E-13
	4.65183E-14
	1.24359E-08
	0.000489545

	1.18597E-08
	0.001007104
	0.00120828
	0.000324118
	0.717553499
	1
	0.02860374
	0.002872553
	1.71313E-06
	9.5449E-06
	0.023630671
	4.10283E-12
	7.9977E-11
	3.53162E-06
	1.67858E-06
	5.54066E-05
	0.99949746
	0.382574392
	4.33908E-12
	4.60743E-14
	4.84699E-05
	6.36158E-14
	1.64026E-07
	7.54951E-07
	0.12538788
	5.4016E-11
	1.93775E-08
	9.97091E-13
	0.005383558
	0.736671747
	0.006221774
	0.86586387
	9.21936E-07
	3.52031E-11
	2.37699E-13

	3.42985E-11
	1.16422E-06
	7.87245E-07
	0.087332211
	0.798445906
	0.693338904
	7.45519E-10
	7.01433E-09
	0.127516722
	0.001747308
	0.004338289
	0.70408137
	1.72881E-05
	8.96477E-05
	0.00932422
	4.60743E-14
	8.37885E-13
	1.8463E-13
	0.202979362
	8.84224E-09
	0.733857253
	0.01577027
	0.006538681
	7.43092E-05
	0.836725187
	4.33406E-08
	4.72901E-06
	1.06248E-13
	0.563351919
	0.00047602
	6.07463E-05
	4.18627E-08
	0.035508676
	4.60743E-14
	7.03086E-05

0.955355
0.000429046
0.073028154
0.039516505
0.999799203
6.11733E-14
0.001028331
0.972437488
0.012514406
0.477639404
0.037274318
0.000323515
0.005244789
0.020195709
0.000134769
1.86778E-10
0.895406912
4.01585E-07
0.015521945
4.85004E-06
2.78488E-07
0.175293135
0.341730542
0.999998679
0.001972919
7.76146E-12
0.938126239
3.35166E-07
3.01801E-07
0.239734636
1.25929E-07
3.6453E-08
2.8806E-10
0.001071506
2.76412E-12

	0.003264282
	0.064025507
	1.36515E-08
	2.90846E-06
	5.09592E-14
	0.011026978
	7.2403E-10
	0.315954809
	4.60743E-14
	6.40599E-14
	0.000808603
	3.61266E-06
	0.032072909
	0.012862313
	6.72886E-08
	4.55002E-06
	3.35078E-06
	2.50133E-13
	9.12923E-10
	0.135292574
	2.3348E-13
	4.60743E-14
	5.73985E-14
	0.00603586
	2.10685E-09
	0.006636175
	5.9619E-14
	0.085229761
	3.76024E-07
	2.5796E-12
	0.012382986
	0.000476542
	0.000105435
	1.86406E-13
	0.467126088

	0.001156998
	0.952174563
	0.671064153
	0.021551117
	0.024343232
	0.135243319
	0.50684359
	0.017464116
	3.24399E-07
	0.99928215
	0.714828119
	0.836001718
	4.08496E-07
	4.57657E-11
	5.31258E-05
	8.83736E-05
	0.946259989
	0.012969646
	0.568125977
	0.403409119
	3.53808E-06
	6.76694E-06
	0.766686076
	0.000142558
	6.67438E-05
	5.5225E-07
	0.000171631
	0.002492351
	7.85629E-08
	0.929333997
	0.751934018
	4.80795E-05
	0.036372962
	0.981016456
	0.999995203

6.20479E-05
0.779988549
0.634223884
0.488485585
0.671911363
0.066750658
4.60743E-14
5.99448E-11
4.76404E-09
0.324991673
0.001993074
7.01257E-06
3.69149E-11
0.005668919
4.60743E-14
3.96755E-06
6.75507E-05
0.999821911
0.011217411
0.321162755
0.936527083
7.84246E-07
0.628247226
0.857223871
0.013621112
0.618828763
0.016406228
1.29619E-12
0.209491352
0.8572666
1.49991E-13
0.000132531
2.92663E-05
0.085291221
1.35304E-05

	0.437664273
	3.91047E-09
	1.91812E-11
	1.04658E-06
	6.5338E-09
	1.14865E-07
	8.85466E-08
	4.85167E-14
	8.9548E-11
	1.60988E-05
	7.95566E-07
	0.200807613
	0.007867298
	0.980529852
	0.017053644
	0.713771452
	0.781529383
	0.000206749
	4.49017E-06
	0.000104174
	0.856508261
	0.900201537
	0.914312862
	0.437915399
	0.898824625
	0.525985381
	0.912661484
	0.703298172
	0.806598382
	0.999124853
	0.62918124
	0.981023461
	0.000210243
	1.72377E-11
	0.000200326

0.095615898
0.918996231
0.999985598
3.21102E-05
1.1033E-05
7.64355E-09
0.016343817
2.75399E-11
0.269693742
2.56934E-10
4.60743E-14
5.91749E-14
4.15334E-13
8.14993E-07
9.92298E-05
0.046188868
9.01102E-06
0.953281725
0.99982961
0.997414272
0.003464245
0.712599613
7.99437E-07
0.580393729
0.302717598
1.06542E-06
4.56683E-06
0.006442156
0.001104335
0.79124925
0.001220565
0.0001124
0.129412998
0.882561072
7.62601E-07

0.878361204
0.20251868
0.867105584
0.147663125
0.012299458
7.27632E-11
3.11802E-07
0.815232639
0.161183101
0.961561321
0.997463762
0.51708754
0.709891453
0.001776495
5.9773E-07

P-value: (CM_pos_12022024_Newanalysis) / (Rabbitserum_12022024_Newanalysis)
0.479404778
0.965056787
2.86548E-09
0.515686566
0.090392472
0.979820698
0.084934827
0.194269777
0.001189448
0.140046528
0.935132943
0.55033034
0.243627994
0.997718755
0.125251095
0.365979984
0.999993161
1.40815E-06
0.000124669
6.30141E-08
0.907529175
0.000582307
0.967545932
1.55098E-13
0.000108273
0.686133826
0.999972933
0.967121345
0.67163531
0.908086329
0.570778769
0.99331741
0.721243749

	0.808181952
	0.008797338
	4.57242E-10
	2.59222E-05
	0.998550106
	0.983862013
	0.3911877
	0.999163211
	1.14335E-06
	0.932656996
	1.71759E-08
	0.858949404
	0.007658036
	0.050950416
	0.085960856
	0.666621602
	0.640915796
	0.87276624
	0.875380215
	0.158780359
	0.15917358
	0.373693056
	0.998219885
	9.15942E-11
	0.998883341
	0.027542961
	0.000292088
	0.466674654
	0.993048888
	0.984539197
	0.291241692
	0.623260583
	0.976289661
	0.038490478
	0.294665576

	0.00033312
	0.627077609
	0.216577733
	0.99770644
	0.253246754
	0.152905894
	0.000140207
	0.999769876
	0.00141397
	3.08032E-05
	0.003022553
	2.4995E-09
	0.24453992
	0.000740439
	0.440522319
	0.191258471
	5.43942E-10
	0.99902888
	0.666771027
	0.907667975
	0.999932055
	0.59020104
	0.87927569
	0.005596044
	0.006676689
	0.069634065
	0.004903077
	0.053078784
	0.231913657
	0.041014312
	0.985785397
	0.999999995
	6.42491E-07
	0.070506224
	0.652200207

	0.530058412
	0.074575626
	0.093783537
	0.955720739
	0.906924091
	0.115811927
	0.723596396
	0.657561741
	0.000308368
	0.232458482
	0.503823806
	0.977517855
	0.00842769
	0.959609232
	0.00101035
	0.98141854
	0.461476651
	0.992691221
	4.60743E-14
	0.027729228
	0.061846087
	0.485023083
	0.835683378
	0.931697854
	0.07728499
	0.000486957
	0.913822125
	0.776800904
	0.001266573
	3.22108E-06
	0.000289721
	0.49217144
	0.996186736
	0.421236897
	0.635872443

	0.997063081
	0.018015686
	0.555152321
	0.999999993
	0.964872237
	0.704397893
	0.999673418
	0.999917834
	0.371646915
	0.464666168
	0.999999991
	3.65632E-07
	2.72338E-13
	0.000495352
	3.44091E-12
	8.9738E-10
	0.995908675
	0.008761149
	0.046143723
	3.47593E-05
	0.001180938
	7.46721E-07
	4.1267E-13
	0.001569389
	0.139812021
	0.6189929
	0.03426357
	0.998559894
	0.050131419
	0.041699029
	9.6101E-05
	0.61787628
	0.001331828
	0.038286111
	3.05543E-05

	0.01872685
	2.67928E-08
	0.089255306
	5.4512E-14
	0.00567648
	5.1901E-05
	0.493170518
	8.32395E-08
	0.132789353
	5.94471E-07
	0.000739847
	0.861485299
	1.93512E-13
	0.067124808
	0.168540094
	1.09011E-06
	1.07127E-07
	0.025088586
	1.05419E-11
	9.25058E-08
	0.000517893
	2.55522E-06
	0.350999474
	0.874917663
	0.504805986
	0.079737078
	0.829200089
	1
	0.117311853
	0.002348401
	0.117610079
	0.262044633
	0.000210243
	0.00050094
	0.008307622

	0.656210995
	0.002006815
	0.071587474
	0.999612672
	0.927040438
	0.535411881
	0.031895026
	0.000919821
	0.187655969
	0.206533018
	0.096826853
	0.999992033
	0.384401458
	0.995815066
	0.999716857
	0.999998442
	0.880096378
	1.96176E-13
	0.036481341
	0.979545719
	0.000357019
	0.007933915
	0.842578865
	2.49347E-05
	5.21496E-11
	0.000847083
	0.999402654
	0.838429048
	0.971011523
	0.979396634
	0.010500874
	0.999998258
	0.000757658
	0.047997472
	5.14215E-08

	0.045176238
	0.079769877
	0.99999462
	0.999199873
	0.175364283
	1.34847E-06
	0.000153172
	0.878455597
	0.156010264
	1.06038E-09
	0.002014503
	0.019358196
	0.005255737
	7.89221E-09
	0.027949713
	1.69553E-05
	0.001033387
	0.032990476
	0.983588259
	4.2404E-05
	0.000181934
	2.4544E-05
	0.008623121
	0.007364782
	0.7734068
	0.98981135
	0.206182503
	0.243968558
	5.94094E-06
	0.001251511
	0.001234823
	0.001800962
	0.507456705
	0.99824027
	4.58356E-12

	1.8349E-09
	1.72313E-05
	0.000108789
	0.021105631
	0.000971894
	0.438660549
	3.98743E-09
	0.668051333
	0.88289873
	2.81745E-08
	0.141793223
	7.36864E-06
	0.999999639
	8.06702E-10
	0.999971404
	0.999458504
	2.05242E-07
	3.45868E-05
	0.808676954
	0.001088456
	0.979566581
	5.67862E-10
	0.950921249
	6.30052E-13
	8.99549E-06
	8.85994E-08
	0.027673552
	2.77467E-09
	0.026521627
	5.3451E-05
	0.049283275
	0.021166816
	0.228456128
	0.542298263
	0.595064204

	1.52999E-09
	0.274652968
	0.023497544
	0.004897014
	0.015766169
	0.708191301
	0.003053862
	1.94485E-10
	0.739683509
	6.07127E-08
	0.646543614
	0.020394572
	0.67935788
	0.924302391
	0.499003328
	0.999994564
	1.86355E-05
	0.002057138
	6.19132E-07
	2.67686E-10
	0.974735158
	0.000707618
	0.415032639
	1.83616E-06
	0.00058611
	0.99445353
	0.400308323
	2.67948E-05
	0.000299847
	0.022982698
	0.003908094
	0.017919899
	6.12657E-07
	0.014955242
	0.702889777

	0.04028375
	0.529212975
	0.016186662
	0.968531033
	0.000115562
	0.212504991
	9.2495E-07
	3.33844E-10
	6.95826E-05
	6.2134E-05
	2.26644E-06
	2.84995E-09
	3.26965E-07
	0.998061634
	0.860708665
	0.538038796
	0.014761643
	3.78869E-06
	0.664246811
	7.86816E-07
	4.82754E-09
	7.87875E-08
	0.999906945
	5.22396E-06
	0.000116587
	0.998862811
	0.434299724
	0.00378708
	0.004535692
	0.000159428
	0.644214528
	0.014143411
	1.18105E-05
	0.950547481
	0.32923254

	0.048836497
	0.458432405
	6.17009E-08
	4.90542E-09
	0.599793166
	3.69376E-08
	4.13953E-07
	2.02495E-06
	2.15521E-05
	0.143071326
	0.001219598
	4.94349E-12
	7.82405E-08
	0.045695798
	0.002296746
	0.136352807
	0.135291787
	0.003618753
	1.15777E-05
	0.457610343
	0.391945827
	0.023484333
	0.001094099
	2.87432E-10
	0.767061894
	0.953570238
	0.067347687
	0.892509309
	0.99713583
	0.619335082
	4.24405E-05
	0.018164804
	4.62109E-09
	0.048397592
	0.348960781

	0.001452865
	0.389365747
	0.002201646
	0.478932335
	0.221314388
	0.039782082
	0.26656924
	0.000679087
	2.84128E-07
	1.1874E-06
	0.007366185
	3.57326E-06
	0.001830772
	0.992713985
	6.51495E-09
	0.00045984
	0.003859686
	0.048820053
	1.61392E-05
	6.12215E-07
	0.00117375
	7.40105E-05
	0.015862835
	0.377386593
	0.055527163
	0.003843109
	1.77536E-08
	6.82277E-06
	0.64227616
	0.955462591
	4.74118E-06
	0.739624113
	0.001527827
	0.001456114
	3.46183E-05

0.000393825
0.000911903
3.5532E-09
0.990005986
0.707872193
0.317311895
0.007767438
9.7493E-05
0.006449347
1.99505E-07
0.004693267
0.155615852
1.63863E-09
0.179711755
0.024201353
0.025490094
7.22453E-07
0.825496305
0.993598169
1.74744E-08
0.483407623
0.009169804
1.30762E-07
1.8169E-07
1.75429E-05
2.6969E-06
0.003661308
2.86327E-13
0.999724972
0.000298635
9.339E-07
5.5111E-08
1.52294E-05
0.994682538
0.982662201

	0.996940781
	0.999981464
	0.926252437
	0.598839984
	5.97366E-05
	5.58442E-14
	0.043276106
	0.99477687
	0.198138348
	0.009070886
	0.063661153
	0.000765602
	0.00476963
	0.078184015
	0.001607053
	0.000675963
	1.11315E-07
	1.39365E-09
	0.188012196
	1.89753E-05
	6.86936E-05
	0.019375702
	0.100850894
	0.938031199
	0.015784719
	3.89418E-11
	0.071925114
	6.03047E-08
	1.68796E-06
	0.431015593
	0.004177168
	0.01280474
	5.43135E-10
	0.999993946
	4.77138E-09

	0.15604422
	0.007303755
	1.69317E-08
	0.003955557
	3.72424E-12
	0.014809405
	1.83545E-05
	0.020104587
	1.68139E-08
	3.87989E-10
	0.004929651
	8.67893E-05
	0.000767585
	0.207715328
	6.24451E-08
	0.321360144
	0.000570291
	3.60236E-08
	1.90215E-07
	0.999982334
	5.30645E-05
	3.09675E-12
	2.72227E-07
	0.00100753
	4.81736E-08
	0.003671751
	4.28072E-07
	0.016437463
	1.32495E-06
	2.69813E-05
	0.00147857
	0.045367111
	1.97194E-08
	0.987066918
	0.950815608

	5.9841E-14
	0.778861286
	2.85118E-09
	0.947136892
	0.482203986
	0.963471574
	0.999998018
	0.411918561
	2.47281E-06
	0.549000318
	0.616189782
	0.999612883
	2.82242E-05
	3.57767E-07
	0.013015348
	0.465559818
	0.999999983
	0.965959361
	0.776967179
	0.670971593
	0.010595996
	0.696497966
	0.039230341
	0.000696302
	0.218693001
	0.996975778
	0.047525589
	0.080408028
	0.215769474
	0.985128734
	0.999978301
	0.000510988
	0.015770591
	0.995870069
	0.966291405

	0.004790232
	0.794270391
	0.908363045
	0.217523791
	0.758787869
	0.000280419
	2.66718E-09
	5.97451E-06
	0.001961908
	0.995594267
	0.03884235
	0.001149376
	2.45454E-07
	8.15974E-06
	1.12613E-09
	0.009854963
	0.000904018
	0.999968992
	0.986691399
	5.79141E-07
	0.979800152
	0.000132707
	0.406726014
	0.655294896
	0.084927369
	0.966594177
	0.863548082
	1.1342E-07
	0.002627209
	0.832063405
	1.19849E-07
	0.001999934
	6.08402E-05
	0.192494587
	2.96533E-05

	0.934713095
	0.95446834
	0.054942065
	0.1399867
	0.672591461
	0.636047021
	0.327864851
	2.86948E-08
	0.196415157
	0.311690967
	9.57022E-05
	0.505575732
	0.95054694
	0.999903062
	0.164382148
	0.65550775
	0.561082489
	0.175649351
	0.000347752
	0.002932943
	0.999960212
	0.980716381
	0.973400716
	0.371720717
	0.948012775
	0.87298473
	0.977477213
	0.936725366
	0.999796823
	0.992109721
	0.629222707
	0.9152115
	0.715514136
	4.54922E-09
	0.587500582

	0.160996628
	0.997552867
	0.995425981
	5.41186E-05
	0.001050681
	1.73329E-09
	0.418664001
	0.004846872
	0.983262023
	0.043260225
	1.44651E-05
	0.000363212
	0.000222234
	7.43986E-05
	0.000118844
	0.290529986
	1.61167E-06
	0.561250344
	0.998388534
	0.98065155
	0.963739958
	0.02937643
	0.025125256
	0.773889059
	0.029998822
	5.87689E-05
	2.20378E-07
	0.973796292
	0.000903491
	0.999685086
	0.014407354
	0.048735002
	0.999580464
	0.998257182
	0.002508187

0.131108841
0.868873208
0.118471429
0.716875359
0.358156375
0.000115615
0.016976547
0.997145151
0.412009367
0.99576665
0.979465791
0.999901013
0.996576803
0.992708084
0.004051719

P-value: (SM_pos_12022024_Newanalyis) / (Rabbitserum_12022024_Newanalysis)
0.967056208
0.019299354
2.51051E-08
0.804859534
5.32024E-06
0.993204626
0.309653397
0.000229072
0.358241223
0.002438739
0.001840469
0.942760159
0.99016905
0.160198455
0.017327786
0.980209689
0.495638453
4.60743E-14
1.37134E-09
2.91434E-13
0.216411128
4.51117E-12
0.049266449
4.60743E-14
4.69624E-14
0.209127162
0.63320257
0.331715684
0.003000052
0.001288384
0.68393626
0.999999992
6.75682E-05

	0.000743738
	1.7292E-08
	3.99258E-08
	7.65772E-11
	0.993095021
	0.803210857
	8.35128E-10
	0.000598051
	0.786875555
	0.760307682
	0.138711232
	2.34414E-06
	3.36881E-09
	2.8584E-06
	1.50323E-08
	8.42242E-06
	0.003279346
	2.26692E-06
	0.000151342
	4.39306E-08
	0.00039481
	7.89963E-06
	0.158677231
	0.360592761
	0.237632112
	0.16737018
	9.69815E-09
	1
	0.002316307
	0.00095194
	6.43277E-06
	6.38624E-06
	3.60805E-05
	0.565487291
	0.000158437

0.712773478
0.999811327
0.000498151
4.09978E-08
0.999999431
0.000518099
0.92848239
0.000550285
4.05652E-09
5.6284E-08
7.71605E-14
4.60743E-14
3.25251E-06
2.79313E-09
1.69533E-06
0.998883851
6.29847E-07
1
0.539331373
3.71991E-05
0.0275948
0.967376766
3.12272E-12
4.15556E-13
0.000408884
0.002075716
1.05373E-06
0.734436449
1.79389E-08
0.000585582
0.337184031
0.459792137
1.25249E-08
1.06175E-06
1.30076E-05

	0.052532732
	1.54623E-05
	4.66068E-08
	0.97158186
	0.791626429
	0.081536704
	0.171787846
	0.060433175
	3.11764E-06
	0.994263627
	0.82116809
	7.68586E-07
	0.999993926
	0.306099309
	4.60743E-14
	7.30621E-07
	0.033162229
	0.000832289
	4.65183E-14
	0.001296819
	0.849236588
	0.006748613
	0.250776112
	0.968243103
	7.4899E-12
	0.998648771
	0.973886191
	0.565857128
	0.00028065
	4.60743E-14
	5.02733E-09
	2.43083E-12
	0.19768826
	0.155429832
	0.997060646

	0.001029128
	0.998507718
	0.218285565
	0.998149205
	0.999973345
	0.994250004
	0.999905708
	0.712813951
	0.997523389
	0.568919099
	0.680298424
	0.001786783
	1.17436E-11
	0.000311672
	1.70565E-10
	9.07821E-08
	0.199382003
	0.846342501
	0.438699506
	0.001494319
	0.947115295
	0.001532938
	1.31389E-11
	0.000274085
	1.54022E-05
	1.15026E-07
	3.51395E-08
	0.958363237
	0.802150476
	8.03243E-05
	0.001437316
	0.658085894
	1.88471E-09
	0.998736576
	0.000155182

	0.999903805
	1.95023E-11
	2.23961E-05
	3.44502E-13
	0.000434417
	1.95527E-06
	0.519538803
	0.014812994
	0.003750097
	0.42507551
	2.48239E-07
	3.15115E-10
	3.052E-13
	0.046998151
	0.999628898
	0.018547595
	0.000169858
	0.372087754
	2.08708E-09
	1.91537E-08
	0.013854747
	0.000188513
	9.34794E-06
	0.000897924
	0.101555762
	0.198135888
	0.080312482
	0.390963855
	0.047269676
	7.6353E-11
	0.990852758
	2.84303E-07
	0.002701225
	8.98472E-10
	0.474578239

	0.003656263
	0.970564608
	0.97694005
	0.501460293
	0.864684998
	2.53257E-08
	4.83809E-06
	0.930216587
	5.61322E-05
	0.418024964
	0.682116572
	0.727039836
	0.764381612
	0.742045184
	0.991917251
	0.807414592
	0.000173265
	4.91829E-14
	3.21961E-07
	0.240141078
	0.038834922
	0.007889444
	0.787288213
	0.90380072
	1.31849E-09
	3.26666E-06
	0.147812677
	0.056015045
	0.498946109
	0.000403355
	0.578783805
	0.109559663
	0.010628988
	0.018076359
	2.15014E-11

	0.872736152
	0.193650822
	0.998326722
	0.889061193
	0.006451684
	0.309215684
	0.001429547
	0.999785884
	0.880149979
	1.6869E-07
	0.079828769
	0.750286559
	0.004300252
	3.95502E-10
	0.507991068
	0.095002064
	8.45731E-08
	0.994372958
	0.213290801
	4.65183E-14
	0.039429912
	0.24250738
	7.29734E-05
	0.002294956
	0.000257035
	0.994122933
	0.227826067
	0.468330113
	0.783871755
	0.028193768
	0.204329996
	5.0775E-07
	0.99140268
	0.998377657
	2.54034E-10

	2.33636E-06
	2.04045E-11
	0.074196419
	0.98718284
	0.061812513
	0.706549724
	2.08136E-08
	0.999756094
	0.229256032
	1.01583E-06
	0.034618508
	7.96567E-06
	0.999533777
	0.007863534
	0.467844157
	0.055566383
	3.59736E-11
	1.54505E-05
	0.626652115
	0.000611604
	1.50716E-08
	1.33438E-12
	0.377897658
	8.37329E-10
	3.49654E-05
	3.71835E-08
	0.034925232
	3.77823E-11
	0.000456781
	7.71849E-05
	4.82107E-05
	0.000416279
	0.927445945
	0.001908157
	0.986986893

	1.54402E-06
	0.002704047
	2.62153E-06
	1.10875E-05
	0.0147723
	0.45439703
	0.271369707
	1.51557E-12
	1.05863E-06
	3.33342E-06
	0.999715561
	0.000934471
	0.330419457
	0.963001566
	5.03904E-05
	0.999739761
	0.000333602
	1.3001E-08
	1.06878E-11
	1.10298E-05
	8.46147E-06
	0.209639794
	0.999770439
	1.42881E-08
	0.000116196
	7.79816E-06
	0.00085365
	1.88849E-05
	0.014644557
	0.0162973
	0.195025685
	0.961440262
	0.000167333
	0.929975739
	0.990462849

	0.000783509
	0.558631008
	0.97404968
	0.000634075
	0.00031292
	0.743436531
	9.2569E-08
	1.02586E-06
	0.000711462
	0.000206688
	1.33882E-07
	2.04966E-08
	1.09847E-05
	0.000143329
	0.432761064
	6.44006E-08
	0.005682451
	3.01096E-05
	0.266870546
	0.000364338
	4.79517E-09
	0.002123417
	0.631675199
	7.47553E-09
	2.34189E-06
	0.999449387
	0.352126175
	0.000181349
	0.000105075
	0.93037302
	0.867022778
	2.04807E-08
	5.46804E-08
	0.000129129
	7.25882E-10

	0.997012199
	0.000411636
	4.53648E-12
	1.72828E-08
	3.73438E-06
	5.19381E-08
	8.97768E-09
	7.81232E-06
	2.55462E-13
	0.000724981
	0.003443064
	1.38157E-11
	1.36773E-08
	8.19151E-06
	0.747149222
	9.36114E-05
	0.004043817
	0.000546595
	4.55674E-07
	0.41372951
	0.049451096
	0.990825861
	2.00211E-05
	1.49939E-08
	0.99999923
	0.99999998
	6.33047E-11
	0.027119995
	0.878467578
	0.006066745
	5.86897E-12
	0.717888322
	0.001966256
	0.787999628
	0.999991862

0.000820318
6.4963E-05
0.999999327
0.991876731
0.255768436
0.998642897
1.18289E-09
9.17921E-07
5.29358E-06
6.92556E-08
0.000803212
8.03658E-06
1.48299E-05
0.00050284
9.46225E-06
0.753431535
2.84118E-10
1.22712E-05
0.734930244
0.420785917
0.000265456
0.00059708
0.07818371
0.746770851
0.250793215
5.29571E-07
0.000416729
8.66318E-12
0.980290961
0.001508101
0.076231141
0.864405801
0.0033756
1.65679E-05
0.000560462

	1.39708E-10
	8.83491E-08
	1.30754E-06
	0.440676365
	0.008962609
	0.959773171
	0.911367424
	0.999747863
	0.001528437
	0.000367079
	0.441930304
	0.002222039
	1.31987E-11
	0.99960481
	0.011973835
	0.999989959
	4.38491E-05
	0.005230673
	0.980636608
	3.05229E-07
	0.003836403
	0.845250844
	1.08118E-08
	1.56172E-07
	3.90922E-05
	4.6242E-07
	0.992062904
	1.13798E-13
	0.005286236
	0.002119634
	3.16773E-07
	2.68212E-09
	5.68122E-05
	0.999870787
	0.018668771

0.616387566
0.004582862
0.985653
0.29407094
0.030196438
1.19926E-09
0.079024259
0.223537809
1.44983E-06
0.06862658
0.000266315
0.562899229
0.00745557
0.999038733
0.424705873
1.68017E-05
0.001160743
0.987798033
0.000277438
0.026355875
0.223313096
0.047224218
0.000113411
0.977427291
0.697667525
7.43878E-09
0.880882172
2.70195E-12
8.80839E-06
0.581564307
0.169624618
0.00031745
9.43512E-08
0.022876591
2.79658E-08

0.904305983
0.9945672
0.807857041
0.02851193
0.000469403
0.005687549
9.88934E-08
0.834794223
4.76286E-14
3.44309E-07
0.504085355
0.360645462
0.871058051
0.188601784
5.56127E-08
0.225843683
0.034600333
0.00880144
0.000196471
0.993304285
0.129817912
4.60743E-14
3.88524E-05
0.000143929
0.000310157
3.36323E-05
2.13263E-12
0.992710948
3.06812E-10
0.280102573
0.380451162
0.000911717
1.69777E-08
0.002064598
0.000471234

	4.80727E-14
	0.349023632
	4.84548E-07
	0.999738835
	0.999411207
	0.998795014
	0.978237012
	0.951819229
	0.001399395
	0.079059435
	0.984041164
	0.868866952
	0.09760254
	1.32219E-09
	0.10075001
	0.122186405
	1.72951E-10
	0.049726728
	0.320659696
	0.891148161
	2.51592E-06
	0.005219202
	0.955810559
	0.001040585
	0.015162648
	0.999973483
	8.51079E-09
	0.999999852
	0.070940442
	0.212384035
	0.351819651
	0.06796718
	0.002555207
	0.998304216
	0.292926341

	0.000120166
	0.978468337
	0.941136024
	0.999769901
	0.460488331
	4.01696E-08
	4.67404E-14
	3.6421E-08
	0.006105905
	2.48314E-11
	6.83281E-06
	0.093535651
	1.55446E-10
	0.439906375
	4.88229E-11
	0.046773155
	0.007243188
	0.994799759
	2.03416E-06
	0.002282007
	0.996806967
	0.006143713
	0.92298541
	0.001330836
	0.000112869
	0.999841805
	1.06989E-05
	3.38228E-11
	0.347310311
	0.970755982
	2.68438E-10
	0.608868322
	0.000986337
	5.05595E-06
	6.58923E-09

	0.005217201
	1.13605E-10
	2.24387E-10
	0.907289008
	1.49495E-11
	0.000105012
	0.437401489
	2.32813E-11
	0.885560527
	0.016475436
	4.62963E-14
	1
	0.906724101
	3.76166E-05
	0.002022493
	0.003887669
	0.556472135
	0.662937427
	0.70536396
	0.6721199
	0.001313314
	0.999042294
	0.999953126
	0.608038266
	0.947901546
	0.953160848
	0.979401198
	0.020570555
	0.774159379
	0.0091755
	0.002159999
	0.045532296
	0.405691699
	4.71845E-14
	0.976357174

	0.006496167
	0.966139484
	2.17874E-08
	0.241597058
	3.73128E-06
	3.68593E-11
	0.995451197
	4.32998E-12
	0.982661236
	9.26655E-06
	4.60743E-14
	4.65183E-14
	1.34526E-12
	3.76877E-07
	2.36876E-06
	0.963072575
	1.28687E-07
	0.039172869
	0.259911442
	0.033339454
	0.688930755
	0.685487429
	3.20131E-05
	0.003696859
	0.000830744
	0.003527485
	6.32406E-09
	0.999999936
	0.999830926
	0.649788758
	2.94083E-07
	0.19457261
	0.999978939
	0.005770707
	0.033040818

	0.905734031
	0.927700993
	0.942659933
	0.742537394
	0.037442327
	3.08229E-06
	8.572E-05
	0.912391723
	0.005591869
	0.789372834
	0.99393126
	0.298115804
	0.999996011
	0.283689931
	0.250612245

P-value: (Bb_pos_12022024_Newanalysis) / (SM_pos_12022024_Newanalyis)
0.300741659
0.003304043
0.969053469
0.002612076
0.001177524
0.491276694
1.57049E-05
0.79031936
5.47958E-05
0.227901492
0.004174453
0.400728421
0.014518949
0.000954698
0.976951091
0.007979153
0.999479602
0.059472499
0.136157033
0.931228793
0.068727059
0.008512018
0.026861986
0.001867856
2.15001E-07
0.389757304
0.978431925
0.992638168
7.74604E-08
0.005298721
0.987746691
1.11254E-07
0.244088151

0.557805245
7.11728E-09
0.950153584
0.978485613
0.000433232
6.35307E-05
0.942000663
0.004920466
0.002902179
0.005453497
0.074154996
0.000263052
0.000161402
0.000262712
0.186447915
0.994119501
0.158741509
0.993416367
0.172756845
0.000437287
0.120439772
7.73238E-05
0.001390064
0.000303449
0.999295335
0.459747804
1.27036E-06
0.052685923
0.000933068
2.52053E-07
0.000998984
0.999970424
1.4506E-05
5.62379E-07
6.48209E-09

	2.56423E-07
	0.128825119
	9.42325E-10
	4.91666E-08
	1.09311E-06
	2.58838E-10
	3.15394E-06
	1.07167E-05
	5.0726E-10
	0.053193864
	0.999435483
	0.000316416
	0.814497696
	4.60743E-14
	0.000473645
	2.89679E-08
	5.77264E-05
	0.011021141
	0.994642916
	0.896999059
	4.88961E-06
	0.015449407
	0.000142367
	0.997130859
	0.000155659
	0.000667209
	0.222737181
	0.000276087
	0.049772214
	0.010820426
	0.817949454
	0.999100425
	0.511448079
	2.72204E-05
	2.60203E-12

0.463487623
0.953664177
0.000784393
0.002548244
0.0982952
0.872371907
0.885084622
4.34363E-07
0.999933819
0.0736549
1.19202E-05
2.49106E-05
1.46894E-05
0.030607419
0.793355163
0.497750608
0.236965294
0.001424468
0.019772941
0.683972585
4.64382E-06
1.08171E-05
0.98584471
0.091650528
4.13542E-08
9.13112E-05
0.99449791
0.863670233
0.437733264
0.000845551
0.998090077
2.59922E-10
2.78174E-05
0.012261536
0.11143748

	0.00016145
	0.541496425
	3.5199E-05
	0.75541798
	0.687838353
	0.887097275
	0.570999855
	0.957796666
	2.52322E-05
	0.999998912
	0.99788477
	0.152906647
	3.52429E-09
	0.55039281
	0.012426201
	1.12474E-10
	0.394606614
	6.20821E-06
	1.21869E-07
	0.999759184
	9.29933E-05
	0.000797437
	0.00695272
	0.000113539
	1.66013E-08
	0.613135109
	3.07944E-07
	0.069351164
	0.026190223
	0.035754444
	0.021212668
	0.133653551
	4.03066E-07
	0.004244202
	0.019447491

	0.999978086
	0.001592817
	0.791148452
	1.83823E-08
	0.003726452
	0.99522643
	0.05590139
	0.000114411
	0.513696355
	4.12345E-06
	0.000292939
	3.84478E-10
	2.96346E-11
	0.001036461
	0.001507958
	5.89818E-05
	0.230539546
	1.3037E-07
	4.80727E-14
	0.022246732
	0.000835083
	0.000108844
	1.76875E-06
	0.001091112
	2.5382E-07
	0.999996634
	0.915766947
	0.005227194
	0.002976887
	0.798480126
	1.91819E-07
	0.791660139
	0.001808911
	0.113287517
	6.60201E-08

0.008935075
0.005767116
0.000221075
0.046378769
0.001669565
2.77556E-10
2.43937E-11
0.020082761
2.27255E-07
0.075581851
0.015096638
0.043636431
2.66331E-05
0.726334349
0.012982046
1.75165E-05
0.951284693
3.19612E-05
0.622208417
0.268253834
2.3894E-05
0.910423238
0.495053285
3.76956E-08
8.14729E-07
0.971573683
0.08012005
0.838034907
6.34774E-05
0.002798478
0.986153597
0.468071016
0.979522806
0.963187396
0.007317427

	0.987969482
	0.982458233
	0.998000202
	0.999690755
	0.879214691
	1.99199E-08
	0.019204238
	0.775909464
	0.000126842
	1.10466E-09
	0.279239511
	0.970897699
	0.790623069
	0.071127105
	0.003403071
	0.986350669
	6.16459E-08
	0.735910374
	0.357709263
	0.667644982
	0.006686657
	0.001823271
	0.00719722
	5.08859E-05
	0.018284677
	0.99461248
	0.751292212
	0.819504104
	0.649146212
	0.996355121
	0.000652521
	0.0001393
	0.000933459
	0.738141557
	7.24976E-14

0.003369282
0.355917509
0.000222821
0.029688932
4.27473E-07
0.4498249
1.59334E-06
0.917150807
0.000690425
0.308747442
0.179683851
0.990261105
0.940808327
1.00773E-10
0.122734259
0.6375709
0.955464464
0.775657837
3.53752E-07
0.999216619
0.260319068
2.96174E-08
0.057285299
2.44584E-06
0.999998247
0.999073819
0.837699778
0.001099155
0.023259229
3.51327E-05
0.999940833
0.311854648
0.990167585
1.10996E-08
0.010103966

	2.37423E-05
	0.013548942
	0.183513584
	0.177839941
	0.986930389
	0.576055413
	6.71302E-05
	0.000112587
	5.88938E-08
	2.90576E-05
	0.009953724
	0.992818509
	3.89757E-07
	4.24107E-05
	0.0001727
	0.921768345
	0.819502243
	0.389089381
	0.002424045
	0.999970606
	4.446E-10
	0.582346837
	0.005505328
	0.002287031
	0.492353614
	0.008656563
	0.999490364
	5.92513E-08
	4.94534E-08
	1.05241E-05
	0.007634764
	0.000307527
	0.007302118
	1.08821E-09
	0.002715779

	2.19879E-09
	0.999997184
	2.77445E-13
	0.061343437
	0.999995702
	0.986407342
	0.637979342
	0.000244315
	0.855685358
	0.007306698
	5.70591E-05
	0.064913356
	0.694099558
	1.43851E-09
	0.083532168
	0.021118804
	0.003629466
	0.999931035
	0.064111531
	0.999638576
	3.8235E-12
	1.00039E-06
	0.111090841
	0.740902079
	0.000827964
	0.993531944
	0.205876223
	0.791963506
	0.999999857
	2.37694E-07
	0.93961294
	0.030932688
	0.886739645
	0.864748996
	2.85804E-11

0.487452256
4.47034E-07
2.96848E-06
0.999997797
0.034323082
0.999999162
0.999997372
0.774437229
0.001447989
0.000275188
0.845127373
1
0.959825
0.987016135
3.06726E-06
0.998607116
0.432639006
1.32465E-07
1.16902E-06
6.58826E-06
0.606342714
1.16593E-07
0.988925347
0.782837374
0.718086609
0.997461729
3.46733E-09
1.10952E-07
0.319638721
0.999989799
0.704004093
7.51121E-12
7.41296E-13
3.38899E-07
0.000717774

	0.003107178
	4.87441E-10
	0.000959924
	6.54857E-05
	0.011773221
	0.998813539
	5.25494E-06
	0.063665427
	0.998265117
	0.453277572
	0.785981375
	1.81424E-05
	0.000482742
	2.64996E-11
	0.987468342
	1.35045E-06
	5.93865E-10
	0.002784522
	8.14114E-11
	4.60743E-14
	0.989275605
	2.23297E-10
	0.000377079
	2.18926E-08
	0.000469139
	4.60743E-14
	0.009883066
	0.879105322
	0.03122497
	3.3103E-05
	0.900618469
	1
	0.055835152
	0.000152599
	5.31085E-09

0.987001767
0.921218084
0.999962698
0.000802516
0.162458295
0.988009855
8.35503E-09
1.39581E-08
0.471871238
0.992266742
0.284056892
0.07785063
4.62963E-14
0.000209362
4.69018E-07
4.60743E-14
4.60743E-14
4.90963E-10
0.567273815
0.73348173
0.107634766
0.205913598
0.000339817
0.236380754
1.48307E-06
5.42899E-14
2.29227E-05
0.999999818
0.230644096
0.993577127
0.404336328
4.62963E-14
1.18556E-08
4.60743E-14
0.325522252

	0.976308928
	9.51822E-09
	0.015819933
	0.913562139
	0.015809156
	6.33958E-05
	0.523856015
	0.638003236
	0.025484322
	0.879810372
	0.429788387
	0.026213527
	0.999992938
	0.008031244
	0.021174656
	0.001209352
	6.3122E-05
	2.17281E-06
	2.09962E-08
	1.01881E-09
	0.000150315
	7.6263E-05
	4.22801E-07
	0.988053159
	0.072415315
	0.050386272
	0.999977268
	0.000266338
	0.808318634
	0.988044194
	9.83684E-05
	0.023566939
	0.194468337
	1.09798E-07
	0.003769266

	0.000196307
	0.018579564
	3.38287E-07
	0.021083664
	4.60743E-14
	0.99982982
	0.365502693
	0.943548722
	0.001000366
	1.77162E-07
	0.070021061
	0.000930173
	0.305739715
	1.90062E-05
	0.999999641
	1.11797E-08
	0.019606003
	4.65183E-14
	0.000708538
	0.354578567
	3.37822E-11
	1.74698E-10
	4.60743E-14
	0.741351251
	1.10467E-13
	0.402003665
	0.104428636
	0.02356555
	4.62963E-14
	8.84848E-14
	0.574218604
	0.999887895
	0.029517055
	4.60743E-14
	3.06291E-06

	4.60743E-14
	0.848289545
	1.05077E-08
	0.010714976
	0.052857869
	0.060271081
	0.163725904
	0.123671351
	0.048192566
	0.159500289
	0.319598678
	0.999999661
	4.64316E-10
	0.677081644
	0.066150493
	0.080200638
	1.34308E-09
	0.992891308
	0.997972776
	0.951513458
	5.89417E-13
	0.180498545
	0.27778785
	0.977873941
	5.75979E-09
	3.45937E-07
	0.00961043
	0.002108331
	0.000194402
	0.028325705
	0.983886978
	0.09107753
	0.897551884
	0.999727696
	0.237718033

	0.999884082
	0.354651675
	0.986563917
	0.658328083
	0.025727777
	0.00010137
	8.73419E-06
	0.102033356
	0.000144787
	2.77808E-09
	0.335663509
	5.37383E-09
	0.985718138
	3.41328E-05
	5.59029E-11
	0.016574878
	2.87738E-09
	0.968353228
	0.038004035
	7.25193E-06
	0.99731096
	0.028645112
	0.990970637
	0.025972777
	0.492830299
	0.769538614
	0.103806566
	0.604502875
	0.999598876
	0.998870307
	0.006860642
	0.009833086
	0.797943814
	0.010457428
	0.067007288

3.09252E-05
0.652416856
0.868703469
1.69486E-05
0.111444927
6.6136E-13
8.82977E-10
4.60743E-14
8.85481E-12
0.139395467
1.50151E-10
0.206355915
0.089167272
5.45531E-06
0.961344454
7.91902E-05
0.057503123
0.011835446
9.79895E-08
0.006043025
5.48966E-05
0.983487603
0.964951437
0.999754574
0.99998245
0.951509275
0.999660849
0.377771972
0.136295093
0.003591243
0.098705333
0.1929786
0.033675425
0.000188447
0.001496519

3.53502E-06
0.999961716
1.47468E-08
0.014579806
0.998619898
0.220948948
0.054584577
0.946307263
0.658780607
0.003160933
8.56567E-06
0.327774945
0.985558186
0.999693223
0.752384077
0.239756525
0.614196126
0.229860363
0.164010302
0.091460863
6.2504E-05
0.99999997
1.16784E-12
0.171714452
0.150697748
0.060687747
5.58442E-14
0.005616393
0.000546369
0.082983267
0.049648106
0.056969997
0.091653217
0.07938733
0.00510865

	0.999999682
	0.724902475
	0.999917855
	0.006050278
	0.997230245
	0.002048892
	0.334255767
	0.999898748
	6.16701E-06
	0.310831154
	0.999998978
	0.998665575
	0.636525002
	0.267945233
	2.08062E-09

P-value: (CM_pos_12022024_Newanalysis) / (SM_pos_12022024_Newanalyis)
0.910239641
0.117943683
0.947943121
0.996343532
0.010225575
0.803716856
0.000397609
0.099329698
4.56388E-06
0.549251559
0.020540129
0.96808641
0.570848224
0.064763036
0.949609067
0.784066546
0.579034582
2.33924E-13
0.001773192
5.17679E-05
0.780989468
3.126E-07
0.241370539
5.03921E-09
1.63397E-11
0.949812368
0.739790527
0.79570249
4.96898E-05
0.018408844
0.999968712
0.995067267
0.003230313

	0.019890694
	0.00041673
	0.453729748
	0.000261679
	0.999985292
	0.990944343
	1.02604E-07
	0.001582518
	3.9378E-05
	0.998527922
	1.5029E-05
	5.46018E-05
	7.63153E-05
	0.011154251
	2.4762E-05
	0.000536985
	0.130486758
	4.82139E-05
	0.003113077
	3.4731E-05
	0.180642966
	0.00189298
	0.067268969
	1.01345E-08
	0.426689916
	0.958748909
	0.00672328
	0.476112792
	0.00050797
	0.000152208
	0.002395027
	0.000493503
	4.7936E-06
	0.000512294
	4.52368E-07

	6.4643E-06
	0.781737886
	0.154447628
	1.3571E-08
	0.289871987
	5.27441E-07
	9.95E-06
	0.001163759
	0.000550946
	0.205966443
	1.18721E-10
	7.51288E-13
	0.001622979
	0.00069102
	0.000291938
	0.090622761
	0.079922908
	0.998936272
	0.999944793
	2.25748E-06
	0.016398475
	0.958463982
	3.02339E-11
	1.57412E-09
	0.901743224
	0.719502184
	0.045372905
	0.586995336
	7.34544E-06
	0.578758951
	0.725155861
	0.441174703
	0.649456098
	0.002932709
	2.13683E-07

0.782691822
0.033098449
7.67901E-05
0.597776649
0.223490636
0.999979052
0.898877234
0.001320737
0.565619985
0.08207035
0.05648625
5.44313E-06
0.00600176
0.789396346
4.60743E-14
4.75124E-06
0.734308376
0.000175873
0.01530454
0.840547103
0.003473347
5.06988E-05
0.899342465
0.999983685
4.44829E-09
0.000163801
0.999843456
0.999218756
0.993530899
4.06009E-13
0.003351197
1.26454E-13
0.430960234
0.99010381
0.358605839

	0.000287693
	0.046878234
	0.004703691
	0.998756436
	0.921039401
	0.942668686
	0.994863486
	0.576381084
	0.640724544
	0.999978372
	0.701747085
	0.043480696
	0.367058344
	0.999979385
	0.460806523
	0.435791896
	0.438024302
	0.000376041
	0.000358703
	0.748646368
	0.012199136
	0.089256554
	0.484205261
	0.987279787
	1.77392E-08
	7.83325E-06
	0.000197532
	0.816599622
	0.494523769
	0.20041868
	0.920347526
	0.999999819
	0.000242464
	0.090744084
	0.991285929

	0.032539615
	0.042501094
	0.03721651
	0.377042112
	0.929198093
	0.836831924
	0.999999977
	0.001294036
	0.666622049
	0.000106287
	0.064765197
	2.54329E-11
	0.999617144
	0.999984137
	0.286368014
	0.013309995
	0.10157634
	0.000126121
	0.200723522
	0.988718103
	0.815355596
	0.630883869
	0.002501132
	4.20215E-05
	0.001306583
	0.997221424
	0.593439771
	0.387898921
	0.998134928
	2.84916E-06
	0.338248787
	0.000118583
	0.93453702
	0.000273451
	6.04356E-05

	0.13474444
	0.014913789
	0.012846588
	0.689971097
	0.319187257
	4.11401E-10
	1.22727E-09
	0.011357367
	8.59669E-08
	0.002370611
	0.801810621
	0.643490344
	0.027379644
	0.949869024
	0.99969163
	0.85506122
	8.37421E-06
	0.239695124
	0.001924259
	0.632394172
	0.482274701
	1
	0.164813303
	1.48308E-06
	0.713717086
	0.359185167
	0.074627333
	0.479651102
	0.145347129
	0.002780448
	0.341795762
	0.134534344
	0.916454178
	0.998341075
	0.025839812

	0.379755319
	0.997570479
	0.993996245
	0.71271597
	0.704239321
	0.000451689
	0.963160452
	0.74560183
	0.013295656
	0.343007236
	0.675088757
	0.322376039
	0.999999591
	0.803836931
	0.644989032
	0.027346779
	0.018458714
	0.107286073
	0.568538899
	1.31702E-11
	0.340809331
	5.78984E-08
	0.507055681
	7.07988E-08
	0.008930681
	0.859460284
	0.99999991
	0.997854711
	0.000215816
	0.831087484
	0.294147253
	0.057218187
	0.210098168
	0.960505558
	0.440921089

	0.085345593
	7.23047E-05
	0.151991532
	0.004159992
	3.05219E-07
	0.997785854
	0.98390218
	0.823073084
	0.022728357
	0.740717112
	3.86159E-05
	0.999999997
	0.999875157
	3.23186E-13
	0.57837738
	0.111783204
	0.012481653
	0.999687694
	0.082344576
	0.999936568
	9.0677E-08
	0.076979077
	0.076883872
	0.020405914
	0.996129228
	0.999339759
	0.999998569
	0.431602104
	5.77663E-08
	0.999993685
	0.122735647
	0.674491595
	0.030785476
	1.74949E-05
	0.921860827

	0.100245003
	0.353799372
	0.023363166
	0.258915493
	0.999999998
	0.998307714
	0.382314193
	0.220866873
	2.91562E-08
	0.658804443
	0.469615742
	0.843669285
	0.014939563
	0.49253158
	4.04883E-07
	0.998381672
	0.900522908
	0.001381127
	0.000909661
	0.002801835
	1.12184E-06
	0.202693379
	0.580957122
	0.441229955
	0.991321654
	3.37598E-05
	0.105998323
	0.999994964
	0.692944874
	0.999991596
	0.550474577
	0.115302482
	0.341516174
	0.129115382
	0.956665221

0.649721812
0.999999963
0.002327353
0.005206988
0.9991319
0.926119127
0.949682146
0.032782392
0.957471126
0.997875412
0.8913747
0.964656211
0.782295706
4.40741E-05
0.052410049
6.02758E-06
0.998951545
0.973212777
0.980881576
0.252878591
1
0.008829676
0.766654374
0.152399583
0.716538405
0.999999935
0.999989075
0.871809982
0.738987154
0.002150227
0.998328036
0.000299267
0.357591019
0.001422034
7.68263E-12

	0.131815795
	2.57691E-06
	0.003188351
	0.995354101
	0.000318474
	0.99999328
	0.66841289
	0.995993691
	1.14765E-07
	0.291797681
	0.998774149
	0.995872888
	0.981787924
	0.032263604
	0.068088589
	0.074464715
	0.678245559
	0.980790525
	0.836060503
	0.008891644
	0.87989922
	0.005259675
	0.699433642
	0.568197092
	0.812779372
	0.943579084
	6.37643E-08
	0.001765288
	0.630794228
	0.216225491
	5.62886E-06
	0.337773557
	0.000456534
	0.501607419
	0.425865815

	0.999937764
	3.14644E-07
	0.001753883
	0.195622812
	0.001107455
	0.015395638
	9.00069E-12
	0.187796545
	0.882218688
	0.885284422
	0.95970005
	0.999660356
	0.514733897
	0.000105776
	0.087768939
	1.08145E-05
	8.50797E-06
	0.042481391
	0.000749442
	0.000111887
	0.993949779
	0.973100026
	0.982425394
	0.02461883
	0.000162503
	2.9258E-11
	0.009038197
	5.91702E-05
	0.957806326
	0.013964696
	0.011253392
	0.999862655
	0.999664724
	0.592484592
	0.912848996

3.64508E-05
0.021524942
0.222270065
0.165241155
0.000187672
0.800921191
0.085732765
0.000211331
0.993901029
0.093602339
0.298099565
2.27031E-06
0.287436563
0.098361508
0.999720487
0.035984652
0.665479435
0.000191776
0.999987493
0.871841793
0.233716237
0.137403905
0.919857557
0.999999896
0.99969719
0.98533061
0.000778459
0.981841667
0.010983432
0.978423978
0.99842054
0.823993383
0.996693458
0.999729825
0.089568891

	0.871638151
	0.00703556
	0.595234308
	0.994370058
	0.211940228
	3.10878E-05
	0.999782685
	0.080032784
	0.000998228
	0.956313479
	0.303840705
	0.054298847
	0.999977042
	0.034174761
	0.154599416
	0.764400235
	0.021576907
	6.34605E-09
	0.118724683
	3.31511E-09
	0.031719072
	4.77091E-06
	7.29661E-08
	0.57692387
	0.326237154
	0.235466582
	0.489063144
	0.001626715
	0.989792151
	0.999861542
	0.613374177
	0.736618952
	0.311174396
	0.031331612
	0.978759363

	0.015611338
	0.001804238
	4.25119E-07
	0.968707031
	4.67404E-14
	0.99893861
	0.396277675
	0.254204139
	7.57527E-08
	0.094901874
	0.259474112
	0.019043506
	0.014635735
	0.000625785
	0.99999997
	0.001674596
	0.618360088
	6.50202E-12
	0.142528192
	0.998709763
	0.050010389
	4.65183E-14
	6.29385E-13
	0.979856468
	7.27307E-13
	0.536842935
	0.00014746
	0.003870107
	4.67404E-14
	7.88093E-08
	0.169283719
	0.652065252
	0.999999879
	0.000360672
	4.22243E-05

	0.849694487
	0.977315937
	0.350957922
	0.84917732
	0.68738726
	0.836102784
	0.989230925
	0.896263807
	0.225778993
	0.001148907
	0.24539571
	0.712009262
	1.9668E-08
	0.251708134
	0.947708088
	0.965368188
	1.57855E-10
	0.007090968
	0.021599116
	0.997982799
	2.49668E-10
	0.15407073
	0.00474106
	0.999989384
	0.821686178
	0.999695138
	2.75961E-05
	0.070266521
	0.992908585
	0.055874222
	0.269197751
	0.412873838
	0.980246428
	0.999999291
	0.062937005

	0.754608534
	0.36902758
	0.999997643
	0.341201697
	0.036555373
	0.028774998
	1.08842E-07
	0.406109932
	0.997982428
	8.58547E-12
	0.032598806
	0.500107209
	0.052185013
	5.64658E-08
	0.735105289
	0.986793349
	0.968967569
	0.999323959
	3.64526E-07
	0.052483869
	0.999892776
	0.720495786
	0.928849828
	0.061309886
	0.137994018
	0.994008302
	0.000247693
	0.020467015
	0.27536404
	0.997841298
	0.154345338
	0.099521233
	0.911823981
	0.003596177
	0.035165902

	0.000419117
	7.95556E-10
	3.54864E-07
	0.6448746
	3.9809E-10
	1.47764E-06
	0.005390609
	4.60743E-14
	0.01868978
	0.725147319
	3.65008E-12
	0.514887362
	0.999987635
	1.99213E-05
	0.455797728
	6.05726E-05
	0.024414714
	0.935116531
	6.51848E-06
	0.108232107
	0.00220413
	0.999408809
	0.99323963
	0.998638587
	1
	0.999847671
	1
	0.158506554
	0.615846736
	0.037413084
	0.098691642
	0.321534509
	0.995433922
	2.33193E-07
	0.944447621

	7.20975E-06
	0.999238187
	6.17209E-09
	0.023165542
	0.343390475
	0.543993219
	0.72857127
	3.88427E-08
	1
	0.037909031
	4.60743E-14
	2.84628E-12
	1.54772E-07
	0.400299865
	0.714949545
	0.76220727
	0.928705384
	0.680165316
	0.122076882
	0.150874581
	0.236665747
	0.480352339
	4.99205E-09
	0.090121046
	0.737691616
	0.671338172
	0.726195333
	0.964964168
	0.000446166
	0.46916585
	0.004874147
	0.983891228
	0.996605362
	0.016616484
	0.909567554

0.627022098
0.999981812
0.015082065
0.087031605
0.855063361
0.775558518
0.383498535
0.99370843
0.357931543
0.96768136
0.999979617
0.419934246
0.999145421
0.604105303
8.62467E-06

P-value: (Bb_pos_12022024_Newanalysis) / (Water_pos_12022024_Newanalysis)
0.11840703
0.012247997
0.999971637
0.859254047
0.452725191
0.19573896
5.94605E-07
0.28811344
0.616250343
0.897366228
0.999654706
9.46455E-11
0.388836719
0.864964333
0.999391637
0.03617144
0.999999999
0.998957292
0.991873322
0.019574989
0.361743789
0.028738396
0.99999977
0.05727081
0.001274598
0.979530049
0.983272604
0.828682418
0.00084606
0.013890989
0.999881352
0.016835495
0.89348488

	0.195434398
	0.30621409
	0.809456516
	0.001062888
	0.11797765
	0.162769765
	0.012209083
	0.553574999
	0.002339081
	0.526504635
	0.054378332
	0.003514362
	0.002732423
	0.000570569
	0.004851838
	0.043353202
	0.01264193
	0.087039406
	0.014873768
	0.000920153
	0.005832327
	0.686236483
	0.017832229
	0.636447808
	0.999911558
	0.480410179
	1.23098E-05
	0.03460508
	0.717584109
	3.68888E-07
	0.327470324
	0.320292183
	0.00370211
	3.26574E-09
	0.027282643

	0.003228629
	0.003910906
	2.9175E-08
	0.000678415
	0.001944402
	0.015229727
	0.000166837
	0.627404044
	0.017938524
	0.001657818
	1.28295E-05
	0.000405166
	0.019570079
	4.85945E-13
	0.009798716
	0.000392787
	0.007933055
	0.026871545
	0.054075923
	0.001043072
	0.011458376
	0.000153191
	0.376806793
	0.000395527
	0.001353377
	0.035149849
	0.342876701
	0.006552034
	7.40992E-05
	0.002205868
	0.000852111
	0.07021431
	0.969858701
	0.000292358
	0.002734768

	1.67289E-07
	0.663251887
	0.004786757
	0.003006358
	0.029683354
	0.998072286
	0.159860382
	0.016600114
	0.999507918
	0.002230607
	0.010487263
	0.001089925
	0.007027816
	0.139870683
	0.000681986
	0.738740199
	0.139002781
	0.032713362
	0.008659663
	0.415537007
	0.736512217
	0.001545757
	0.999823701
	0.008920405
	0.041862886
	0.000179923
	0.122282071
	0.110355977
	0.999389704
	0.46196173
	0.999998554
	0.000685764
	0.039557939
	0.002811158
	0.788289195

	0.184109038
	0.495514418
	0.080253407
	0.244056846
	0.998527926
	0.999880217
	0.95956
	0.999574267
	0.001832699
	0.747002004
	0.999993006
	0.90585789
	0.004472702
	0.957318715
	0.010311128
	0.035426098
	0.943758551
	0.419657455
	7.91902E-08
	0.973113524
	0.005638077
	0.053299429
	0.546072435
	0.588004095
	0.999997497
	0.004926384
	1.58706E-06
	0.107548974
	0.023417844
	0.039724982
	0.483033068
	0.021037232
	1.78021E-06
	0.999999464
	0.983928745

	0.000913501
	0.024700466
	0.278714483
	0.072119822
	0.965139865
	0.998103955
	0.997518462
	0.039185447
	1.06692E-13
	0.95183198
	0.01130029
	0.001438908
	0.00353075
	0.131947763
	0.002714105
	0.001747099
	0.09909997
	0.496765121
	0.213936069
	0.015554191
	0.897974773
	0.434868106
	7.1564E-06
	0.137421917
	3.83155E-06
	0.994046786
	0.863434238
	0.974131574
	0.006775634
	0.458554112
	0.005349503
	0.092180137
	0.58563661
	0.299757395
	0.834486178

	0.002113312
	0.032034392
	0.024776083
	0.460298716
	0.000140664
	0.081702431
	0.845898635
	0.917053692
	0.254999043
	0.023374499
	0.794975348
	0.193213758
	0.443356411
	0.799170933
	0.040244784
	0.00025929
	0.091645986
	0.879887279
	0.999976926
	0.975024862
	0.867037442
	0.978948405
	0.192680043
	0.288815403
	0.0072054
	0.613551906
	0.034694535
	0.421275353
	0.030686258
	0.714903993
	0.87436983
	0.845828882
	0.999963645
	0.608481939
	0.006881337

	0.00103089
	0.031285802
	0.999999692
	0.999992715
	0.974560073
	0.154728233
	0.781180789
	0.961823221
	0.110259503
	0.730370803
	2.81234E-09
	0.057828452
	0.999999571
	3.52047E-06
	1.79367E-07
	5.32474E-05
	0.001248576
	0.03398447
	0.746340755
	0.966677681
	0.043320355
	0.041544379
	0.081931851
	0.911706152
	0.514110152
	0.64409141
	0.904978575
	0.999468609
	0.00275846
	0.977450744
	0.999941578
	0.006700548
	0.000154884
	0.998858145
	0.885549655

	0.000131306
	0.266392722
	0.006627046
	0.981155975
	0.999995883
	0.973723379
	0.599461261
	0.508411351
	0.001798199
	0.017375359
	0.102489532
	0.04819147
	0.131977132
	4.50347E-08
	0.089591404
	0.999726057
	0.089863759
	0.013947526
	0.458352765
	0.765090136
	0.803024086
	0.000525257
	0.003152647
	0.006442221
	0.17260323
	0.025031402
	0.993857797
	0.369761936
	0.000224562
	8.88467E-07
	0.999999828
	0.72143609
	0.000161592
	0.000341678
	0.449545805

0.021399736
0.000874849
0.975694644
0.008295314
1.29356E-07
0.030866808
0.016817275
0.013376924
0.001036492
0.427990918
0.685952203
3.5322E-08
0.993184297
0.548909506
0.984220702
0.006166919
0.024374605
0.99999988
0.709060291
7.46471E-06
0.056250898
6.28438E-08
0.999981239
0.595167486
0.711194641
0.013726757
0.050222885
6.25441E-11
0.987232404
9.96328E-08
2.12736E-05
0.013337612
4.34477E-07
4.96115E-11
0.000383576

	0.002024064
	0.90431215
	4.84629E-11
	0.000137472
	0.304796589
	0.414845022
	0.013443764
	0.012338334
	0.032094782
	0.224758515
	2.34297E-09
	0.321725019
	0.154013122
	1.2708E-09
	0.013902028
	0.0722624
	0.071271669
	0.19220092
	0.997759144
	0.068601768
	0.086944565
	0.038510633
	0.980383097
	0.403906727
	0.484146315
	0.616723026
	0.767265489
	0.98889624
	0.023911425
	0.000263172
	0.430814096
	0.827996605
	0.571569439
	0.025273167
	0.748503103

	0.348511237
	0.000106903
	1.15429E-08
	0.465429529
	0.491923944
	0.025633515
	0.602432687
	0.796016894
	3.30823E-07
	1.01589E-05
	0.353140281
	0.081454285
	0.025241329
	0.975764782
	0.999818954
	0.981608289
	0.461673317
	0.990654664
	0.221549342
	0.223598952
	4.85167E-14
	0.157114055
	0.068440459
	0.016838253
	0.003871322
	0.996899692
	1.80732E-06
	0.113167037
	0.999975068
	0.198325195
	0.055150378
	2.67453E-13
	0.309983002
	0.980519161
	0.92085665

	0.416907817
	0.106418237
	0.031548725
	0.945493417
	0.016704901
	0.877831588
	0.001838743
	0.028221323
	0.542514286
	3.23543E-10
	0.060976132
	0.996641394
	0.999995299
	0.836356137
	1.10965E-10
	0.000108026
	3.46512E-12
	0.028009441
	0.713479745
	0.239547527
	0.383511974
	0.152536018
	0.999665369
	0.902258728
	0.099786602
	0.983553747
	0.619809023
	0.999992153
	0.854876749
	2.17338E-05
	0.013190119
	0.000551359
	0.04509763
	0.99998928
	0.656254076

0.999751658
0.998215482
0.040812107
0.9610184
0.941925552
1.40784E-06
0.999706961
0.999991908
0.994863074
2.31871E-07
0.014305554
9.08303E-09
4.60743E-14
1.27865E-08
1.30373E-10
4.60743E-14
4.60743E-14
6.28086E-12
0.148365416
0.00035558
0.743649516
0.999983348
3.16349E-05
5.30798E-13
5.89528E-14
5.76206E-14
4.68836E-12
4.37624E-07
0.378279957
0.003600318
0.069565027
2.21345E-12
2.85438E-13
4.62963E-14
0.004298348

	0.446695259
	0.789291354
	0.174421207
	0.999428648
	8.70348E-05
	0.952404702
	7.11398E-05
	0.999999986
	0.957453818
	0.94497764
	0.999992933
	0.582847347
	0.883195245
	0.999155944
	0.956438969
	0.999998115
	2.17138E-10
	0.038123799
	7.10585E-08
	0.693412632
	0.190481291
	0.847777172
	0.992368572
	0.952968152
	0.999999742
	0.020771906
	0.000897168
	0.027635617
	0.999512573
	0.999820536
	0.741462716
	0.843995054
	0.39598439
	0.000376326
	0.647279345

0.991489737
0.932900232
0.861556569
0.000212884
0.672212756
0.823152528
0.941826784
0.244945905
0.078913979
0.999985058
0.753261539
0.579772368
0.010858137
1.13999E-05
0.943300463
0.096738986
0.999994096
0.946942374
0.65824949
0.00072487
0.03889719
1.022E-06
0.898022775
0.660646087
0.999999904
0.000249257
0.996493715
0.15700725
0.197446251
0.115261784
0.997716372
0.397154463
0.4719131
8.3944E-13
1.66604E-06

	7.16094E-14
	0.962027457
	5.07079E-10
	0.999992448
	0.258732188
	0.944044444
	0.6017807
	0.962621935
	0.444199331
	0.838441842
	0.083146005
	0.999985689
	0.950832432
	0.461055872
	0.999390409
	0.021280378
	8.52415E-08
	0.057439153
	0.012807565
	0.07735793
	0.550752838
	0.031703624
	0.991494949
	0.78695487
	1.28441E-07
	1.64269E-05
	0.998895904
	0.037033573
	8.98823E-05
	0.907362483
	0.136483797
	0.139894741
	0.935112907
	1.99377E-09
	0.864328551

0.128264578
0.999745463
0.999999996
0.999196899
0.999696362
0.000144434
0.001038037
0.081197311
0.969941863
0.994919745
0.99999973
1.54375E-08
0.240560824
0.001693863
0.213926872
0.290726571
2.62909E-09
0.152479538
0.805035332
1.05658E-08
0.131900387
0.890789402
0.999150584
0.400688321
1
0.156285538
0.970060351
0.924197209
0.001316811
0.78070318
0.394553061
0.712908539
0.999999915
0.99999643
3.92302E-06

0.010147287
0.0001357
0.002995682
0.008518225
0.020516041
9.95703E-05
0.000862603
2.61769E-07
0.00043195
0.013372055
0.972630454
0.027174246
0.002491765
0.000309056
0.685646275
1.82824E-05
0.085160374
0.44282237
0.035268624
0.024289352
0.685412056
0.09958539
0.079950643
0.982427547
0.122107888
0.07403587
0.094587404
0.182616537
0.195097451
0.581988598
0.000533369
2.28213E-05
0.004900648
1.34467E-07
0.00427582

	1.46352E-05
	0.019694258
	0.229710708
	0.999478239
	0.097734229
	0.005417782
	0.006901034
	0.002035143
	0.999993912
	0.091426609
	0.097564545
	0.001009233
	0.001235144
	0.326169815
	0.916830587
	0.523524269
	0.132827197
	0.991890138
	0.998820865
	0.57086259
	0.006093579
	0.956022096
	5.71765E-14
	0.044634504
	0.507160833
	0.185999263
	1.59465E-09
	0.958677555
	0.999976687
	0.631209657
	0.999051013
	0.966955258
	0.998919375
	0.031965068
	0.411636429

0.788575425
0.000516594
0.999987395
0.84713454
0.524145731
0.661111866
0.296673819
0.951008153
0.401183641
0.259399161
0.557885655
0.999826743
0.998907594
0.537500264
0.999997325

P-value: (CM_pos_12022024_Newanalysis) / (Water_pos_12022024_Newanalysis)
0.651628913
0.296072658
0.999999608
0.130794982
0.90400668
0.447232801
1.40893E-05
0.44653527
0.161520721
0.571435565
0.937759245
1.32264E-09
0.99999993
0.085865147
0.996481581
0.982310834
0.785591748
4.80727E-14
0.825316802
6.31705E-08
0.998318752
8.70984E-08
5.3042E-05
2.05822E-10
8.16014E-14
0.998938754
0.762422455
0.40959597
0.251330904
0.045064043
0.982026794
0.000369892
0.062117167

0.988362374
1.28709E-11
0.685590631
0.821108677
0.324271305
0.155277362
0.000165133
0.310851807
3.14868E-05
0.124260091
0.999915521
0.000760824
4.40096E-06
0.005409841
0.00193808
0.85187323
0.685380285
0.213458252
0.048900153
1.64081E-05
0.860977933
0.121102788
0.403903475
0.222448826
0.497582495
0.965441705
0.051543709
0.371144873
0.581630522
0.000227311
2.45312E-06
0.138061505
0.001254042
0.086787687
0.58135463

	0.06279326
	0.090454922
	0.879241547
	0.000165219
	0.992134812
	0.997355469
	0.000532614
	0.999993899
	2.69521E-11
	0.009535106
	0.000368386
	0.02545749
	0.268841438
	0.999805008
	0.00623993
	0.972172499
	0.90744261
	1
	0.00922299
	0.029092002
	0.984696876
	0.140193752
	0.003387635
	0.002337693
	0.467623209
	0.062739887
	0.024405237
	0.997238781
	0.006414479
	0.902383065
	0.001357936
	0.984905098
	0.154899155
	0.026021643
	0.980435167

	6.75179E-07
	0.135976709
	0.000494792
	0.636377055
	0.078190825
	0.994943448
	0.171296325
	0.991706856
	0.864621525
	0.72347187
	0.995801585
	0.00023854
	0.574369278
	0.990858083
	4.76286E-14
	0.023727542
	0.019267654
	0.004862516
	0.006627339
	0.26837449
	0.90641312
	0.006805931
	0.98589632
	0.857821468
	0.257744823
	0.000322294
	0.068346496
	0.018689692
	0.093596239
	1.13976E-10
	0.0129473
	1.56849E-08
	0.957134174
	0.999999991
	0.987582177

	0.265575766
	0.039487831
	0.900117908
	0.787276275
	0.946662995
	0.99999979
	0.999622663
	0.870291168
	1
	0.702448864
	0.360729746
	0.594479826
	0.028523239
	0.202714995
	0.417638209
	4.25499E-05
	0.922390118
	0.998133178
	0.000226023
	0.19138944
	0.303358045
	0.863537791
	0.999127321
	0.00173576
	0.99999937
	0.971945184
	0.001073861
	0.9049229
	0.466461561
	0.217286717
	0.00260601
	0.945107879
	0.001117174
	0.756400839
	0.015765332

	0.635765728
	0.338590875
	0.23383449
	2.05133E-09
	0.002490344
	0.791258721
	0.155547937
	0.238349033
	3.6218E-11
	0.999570109
	0.62760315
	4.88659E-05
	1.32034E-06
	5.53624E-07
	0.398895113
	6.90996E-06
	0.038458894
	0.972318904
	1.17976E-11
	0.996732323
	0.203864827
	0.397733806
	0.009593982
	0.009098143
	0.018389654
	0.872078359
	0.67897426
	0.095582608
	0.976062462
	0.013397487
	0.537547238
	0.781806724
	0.000267607
	0.00118193
	0.009441931

	0.351019138
	0.07450583
	0.464903807
	0.999918453
	0.056221577
	0.116919014
	0.99224535
	0.971684939
	0.127868426
	0.385117997
	0.913380507
	0.960901659
	0.977648845
	0.483590818
	0.99997324
	0.999969505
	0.111282457
	0.000817433
	4.04581E-05
	0.999998536
	0.054798667
	0.999655501
	0.546925275
	0.973397925
	0.4183243
	0.838126579
	0.032101895
	0.788640349
	0.999925736
	0.71632883
	0.999694072
	0.405244546
	0.427773425
	0.368810985
	0.027355379

	0.387382545
	0.017567162
	0.999966918
	0.798797544
	0.88291663
	0.821501742
	0.005512128
	0.950101109
	0.930684751
	7.43392E-06
	4.08177E-05
	0.566792277
	0.79022139
	1.1739E-10
	0.324868014
	0.5493959
	0.999959246
	0.952051034
	0.911448132
	3.1094E-12
	0.788621447
	0.980968422
	0.975154473
	0.706387778
	0.34967219
	0.289069089
	0.214551679
	0.762175251
	0.990460295
	1
	0.085851477
	0.001571483
	0.056077459
	0.999974562
	1.54432E-13

	3.37641E-06
	0.000120426
	0.803107166
	0.999999343
	1
	0.283314732
	0.00085105
	0.374079023
	0.051860207
	0.094377431
	0.989167817
	0.010542483
	0.030441595
	4.92583E-11
	0.483800122
	0.957827416
	0.546061806
	0.000940985
	0.08093905
	0.683046016
	8.1716E-09
	0.968881515
	0.004494573
	0.993755502
	0.078734828
	0.023588421
	0.993432759
	0.00148222
	7.99067E-10
	1.72473E-12
	0.162427762
	0.000308966
	0.735942882
	1.6929E-07
	0.949884532

	3.66174E-06
	0.047980036
	0.000275046
	0.014198858
	7.47965E-07
	0.357383645
	1.01149E-06
	0.003730803
	0.002192473
	0.171375726
	0.023771565
	3.45611E-06
	0.001415926
	0.24720316
	0.638402521
	0.025685351
	0.015341296
	7.3011E-06
	4.03175E-07
	0.381077781
	1.3827E-05
	0.003193286
	0.297406657
	0.00116031
	0.932913234
	0.639100422
	0.999999999
	0.053537258
	6.36402E-09
	0.433070946
	0.996949588
	0.812209169
	0.652690643
	0.81687763
	0.999995187

	0.011505752
	0.925662463
	0.596940448
	0.594902775
	0.204009474
	0.254764567
	0.002332808
	0.504467362
	0.06543458
	4.47226E-05
	0.000385222
	0.99999999
	0.11347137
	3.81023E-05
	0.008109829
	0.999991432
	0.957349272
	0.064471685
	0.003939014
	0.92497309
	1.7477E-09
	0.999974553
	0.986783821
	0.389439116
	1.3095E-07
	0.883325441
	0.007336981
	0.493493985
	0.34516662
	0.527165372
	0.737728038
	0.057987262
	0.130495506
	0.248875519
	0.373144106

	0.816367969
	0.000650515
	9.08273E-06
	0.713874913
	0.014192055
	0.022952049
	0.033695171
	0.316691971
	7.4361E-11
	0.025035946
	0.082748813
	0.221413699
	0.017984044
	0.000919108
	0.016502117
	0.137021668
	0.648087064
	1.01301E-07
	2.46965E-05
	0.999390073
	3.02092E-13
	0.698236058
	0.328605985
	0.00677379
	2.62344E-06
	0.948363137
	4.49207E-05
	1.06314E-05
	0.99916265
	0.999998622
	0.000421756
	0.006974961
	9.32566E-06
	5.98226E-06
	0.46678797

	9.12588E-06
	0.999999542
	0.018459081
	0.003857167
	0.126539832
	0.338038255
	0.863657079
	0.007495593
	0.905355196
	1.93054E-09
	0.15969374
	0.000154901
	5.45727E-06
	0.000453376
	1.78575E-08
	1.30223E-05
	1.62761E-08
	0.264134552
	0.000502008
	6.7909E-12
	0.041716906
	7.15314E-07
	0.005325443
	9.79182E-06
	0.209628242
	7.03898E-06
	0.64155508
	4.209E-06
	0.807174514
	0.009510832
	0.921783869
	0.001092626
	3.51892E-05
	2.62162E-06
	1.54779E-09

	2.92524E-06
	0.07843088
	0.910528953
	0.748381939
	0.012827742
	7.98954E-06
	2.8549E-05
	0.009270416
	0.483739823
	0.002039808
	0.013273582
	0.000337945
	5.41809E-08
	0.943961625
	0.012104989
	0.995443124
	0.098408646
	0.036852031
	0.97863554
	0.0001662
	0.926155409
	0.999999927
	0.457869747
	4.42558E-11
	5.73056E-08
	0.996614133
	0.560541976
	6.13506E-08
	0.99063534
	0.002294815
	0.744147794
	0.007150684
	0.00115957
	0.999226693
	0.961261171

	0.250281304
	0.008737621
	0.994350842
	0.442662698
	0.998515963
	0.989765017
	0.003875848
	0.771622756
	0.321547317
	0.07919571
	0.999998414
	0.394469857
	0.868405973
	0.931867552
	0.999999912
	4.03782E-05
	0.656288931
	0.942949516
	0.286047399
	0.926754038
	0.998991335
	0.999980635
	0.812961191
	0.434543003
	0.951131839
	0.116029417
	0.114621884
	0.11947828
	0.999455852
	0.999995988
	0.170645035
	0.014206303
	0.561734078
	0.999852424
	0.459185325

	0.895585975
	0.458501623
	0.821553752
	0.153484417
	0.31312359
	0.875897798
	0.050296075
	0.013680455
	1.04798E-09
	0.000490766
	0.982710792
	0.996234182
	0.25167098
	0.000375704
	0.950408899
	0.541421265
	0.371562105
	0.000843938
	0.987001666
	0.233053976
	0.00021118
	1.68421E-13
	1.81053E-06
	0.957237394
	0.828350994
	0.000465099
	3.41471E-07
	0.488486132
	0.420305813
	0.004195885
	0.809818086
	0.999968509
	0.730432045
	0.999747674
	2.26537E-05

	0.000446385
	0.803413034
	0.972080843
	0.116044975
	0.984055793
	0.951592303
	0.999829613
	0.194830942
	0.876408398
	0.956262973
	0.116385528
	0.570767949
	0.981922968
	0.429539717
	0.542672838
	0.999915149
	8.19904E-09
	0.71486304
	0.986966396
	0.028485001
	0.809693811
	0.547218395
	0.188444974
	0.44160984
	1
	0.533113888
	0.497746962
	0.504409706
	0.999733919
	0.976942983
	0.003640892
	0.021007836
	0.794670574
	3.20281E-09
	0.480265277

	0.910383696
	0.999845194
	0.992579484
	0.99997122
	0.999991202
	0.038851601
	1.50028E-09
	0.469524603
	5.54631E-05
	0.043333097
	0.814292284
	0.269003843
	0.719220685
	2.36113E-06
	4.65673E-10
	0.978585255
	0.974909942
	0.060836727
	6.53697E-05
	0.994567066
	0.083765455
	0.96456028
	1
	0.626156802
	0.965329336
	0.028398362
	0.039065624
	0.00408162
	0.129079474
	0.809384306
	0.001557761
	0.997804384
	0.99950877
	0.999679824
	1.81542E-06

	0.092338795
	0.043514336
	0.00149011
	0.974715484
	0.0032821
	0.930898919
	0.779899942
	0.998889636
	0.012214638
	0.999002725
	0.917430547
	0.005943735
	0.778418777
	8.37735E-05
	0.159155493
	1.40058E-05
	0.177680674
	0.956351414
	0.592073281
	0.298693182
	0.083165769
	0.047134814
	0.046102699
	0.965122895
	0.087223619
	0.017654303
	0.051194943
	0.064038281
	0.730594395
	0.979993636
	0.000533271
	5.2652E-05
	0.999817895
	0.000104923
	0.99564123

	3.00941E-05
	0.004989612
	0.118021752
	0.995325337
	0.882726763
	0.025469672
	0.247951819
	0.041072367
	0.573656679
	0.001086592
	5.33695E-12
	2.68837E-05
	0.005352992
	0.997967668
	0.936256719
	0.114466597
	0.39225049
	0.99989167
	0.99989857
	0.722898728
	0.9906965
	0.106477736
	1.64609E-11
	0.0207552
	0.069396571
	0.931468178
	0.00106267
	0.227694448
	0.999998548
	0.992553125
	0.99204313
	0.653147015
	0.415228598
	0.005968057
	0.902374145

0.999947138
0.013911121
0.012660601
0.232338711
0.024867209
0.003461794
0.426542122
0.999998946
0.167125199
0.944026869
0.717390178
0.500031791
0.231874978
7.58027E-05
0.042513634

P-value: (Rabbitserum_12022024_Newanalysis) / (Water_pos_12022024_Newanalysis)
0.999752526
0.062918716
2.38945E-09
0.001921105
0.007841047
0.856024227
0.026446179
0.002468685
0.350736766
0.942757214
1
8.5445E-08
0.266709456
0.031943621
0.044361446
0.774556209
0.709690464
4.60743E-14
4.31794E-06
4.94049E-14
0.707839108
1.71585E-12
6.02297E-06
4.60743E-14
4.60743E-14
0.879947306
0.658020342
0.102537849
0.974457665
0.003538393
0.209383651
0.001677782
0.001819128

	0.988026899
	5.65104E-14
	1.54982E-08
	9.12746E-07
	0.554507748
	0.461473047
	7.74022E-07
	0.167395691
	0.827830851
	0.014590673
	3.00845E-08
	3.20013E-05
	2.98482E-10
	1.33663E-06
	8.98142E-07
	0.113762483
	0.053497793
	0.019366571
	0.003089977
	2.21521E-08
	0.012161766
	0.986562976
	0.658196671
	2.12675E-08
	0.289853378
	0.156780192
	7.91912E-08
	0.999979996
	0.885431373
	0.001411109
	9.27067E-09
	0.003280789
	0.008737301
	0.999063341
	0.99567219

	0.347665192
	0.830197756
	0.020541022
	0.000557592
	0.08422655
	0.335519784
	0.996452028
	0.999990959
	5.20695E-14
	1.88902E-09
	5.65216E-09
	1.76581E-12
	0.001404126
	0.001522635
	3.79151E-05
	0.038846941
	6.16003E-09
	0.999192755
	0.000160237
	0.243856833
	0.997459121
	0.934888884
	0.000169765
	5.49579E-08
	4.62645E-05
	3.07174E-05
	5.21594E-07
	0.139596863
	1.23434E-05
	0.003039498
	0.00766702
	0.981444301
	1.18189E-09
	1.08975E-05
	0.256677065

	8.17151E-09
	9.34255E-05
	2.65602E-07
	0.980324875
	0.470198768
	0.29905993
	0.006800535
	0.933761829
	1.33992E-05
	0.949152604
	0.802404526
	3.20261E-05
	0.302293118
	0.71452894
	4.60743E-14
	0.004081932
	0.597150679
	0.02036357
	4.67404E-14
	0.87889566
	0.00505904
	0.33280476
	0.453308356
	0.999947024
	0.988429942
	0.999988391
	0.006030178
	0.292139588
	6.57326E-07
	4.65183E-14
	1.82092E-08
	1.61459E-06
	0.999216225
	0.400851026
	0.938883471

0.517022608
0.999445743
0.987059814
0.769205911
0.548859236
0.743387911
0.990500919
0.944582414
0.366060676
0.998775051
0.341982211
2.95685E-05
4.67404E-14
0.165096106
2.0138E-10
5.56222E-14
0.996609162
0.025023367
0.346012104
0.021633329
0.191524363
1.63325E-05
2.3237E-13
1.24991E-08
0.164164161
0.210595451
1.70729E-07
0.987938592
0.825379751
9.10654E-05
1.54196E-09
0.982212588
7.21142E-09
0.001206331
3.02404E-09

0.000304688
1.99225E-10
0.00026545
4.60743E-14
5.91989E-08
1.52819E-06
0.002210453
3.73509E-05
1.46735E-08
2.61283E-07
9.75678E-06
0.001136092
4.60743E-14
4.04158E-10
0.994955656
5.25247E-13
0.000573872
0.003641163
4.60743E-14
2.76056E-08
7.95636E-07
1.60332E-08
3.86009E-05
0.119210775
0.540600387
0.531707399
0.107330977
0.094484801
0.022618227
1.32777E-07
0.001831317
0.016239593
4.25855E-10
3.18193E-09
3.25095E-07

	0.01501394
	0.693593356
	0.895462721
	0.994685906
	0.349894178
	0.933926373
	0.007749773
	0.000111837
	0.999950631
	0.002026234
	0.009150385
	0.982353901
	0.107451806
	0.784839781
	0.999997649
	0.999735169
	0.61884196
	2.76064E-09
	9.01698E-09
	0.964117127
	1.0243E-07
	0.016808638
	0.072715131
	0.000201022
	1.0637E-12
	3.14108E-05
	0.068511717
	0.999998466
	0.993469789
	0.979411766
	0.021618
	0.34949185
	0.08754073
	0.891238219
	2.26142E-11

	0.000272606
	8.18266E-06
	0.999999909
	0.939236845
	0.015787232
	5.40471E-08
	4.54965E-09
	0.999912415
	0.633886883
	4.9849E-14
	5.97855E-10
	0.000232968
	0.111627776
	4.60743E-14
	0.000111214
	1.78486E-07
	0.001743042
	0.20380059
	0.553664011
	4.62963E-14
	0.006015378
	0.000169604
	0.001210287
	0.191453279
	0.024391116
	0.637917972
	0.000752631
	0.93910708
	1.17467E-06
	0.001238171
	5.74979E-07
	1.29459E-08
	0.816635489
	0.990878621
	4.60743E-14

	4.9627E-14
	3.02091E-11
	0.003435439
	0.025869564
	0.000988341
	0.004256964
	2.50577E-13
	0.996290385
	0.399482808
	4.38748E-05
	0.401103435
	0.112795762
	0.036284992
	0.814278848
	0.379834357
	0.850096923
	2.90768E-09
	0.836381528
	0.003852229
	0.046868401
	1.48099E-09
	3.59206E-09
	0.039370114
	1.73539E-12
	0.019410325
	0.000825041
	0.006998628
	2.72338E-13
	3.62733E-06
	4.60743E-14
	7.17972E-05
	3.17951E-08
	0.010796913
	2.40132E-09
	0.15934331

4.94049E-14
0.000157927
3.18719E-08
2.90966E-07
0.011016701
0.019131716
4.0936E-11
9.91429E-14
4.93517E-05
1.67421E-10
0.000418854
0.034217068
2.3736E-05
0.033465125
0.999913862
0.034849535
1.92091E-09
1.47094E-10
9.31477E-14
3.88567E-12
1.81397E-06
1.06447E-08
0.004137802
3.04171E-11
4.33822E-05
0.90942957
0.413309434
9.4371E-09
0.004188224
0.000154024
0.001113488
0.253951247
1.21394E-08
0.220320977
0.774500905

	0.995151187
	0.972576175
	0.420265425
	0.189186434
	0.054942529
	0.999998045
	0.075925516
	2.28417E-08
	0.124066159
	3.79711E-11
	1.52637E-11
	2.61205E-09
	4.52571E-10
	0.000123896
	0.116546625
	0.452342917
	0.102360993
	0.011101955
	6.38894E-05
	6.26044E-08
	4.60743E-14
	5.03059E-08
	0.998281221
	2.98401E-08
	7.76601E-13
	0.97981563
	4.37823E-05
	2.85848E-05
	0.380883016
	0.016160759
	0.99998569
	4.32334E-06
	1.27932E-08
	0.042400074
	0.99999936

	0.002180538
	0.06943008
	1.21347E-13
	1.75807E-10
	0.00019052
	0.000331917
	1.58497E-10
	8.86728E-09
	4.71845E-14
	2.68138E-05
	5.42525E-07
	1.05138E-13
	0.00097941
	2.03429E-07
	1.61503E-07
	0.000212164
	0.00355005
	7.40263E-12
	6.73117E-12
	0.279779981
	1.2233E-11
	0.000521956
	0.165306562
	4.78874E-06
	0.000102383
	0.999999999
	1.93814E-08
	5.97083E-07
	0.962018148
	0.558393995
	1.67027E-10
	0.998884805
	5.82867E-14
	2.25995E-09
	0.006823934

	1.32248E-10
	0.433879304
	1.74472E-07
	2.7266E-05
	0.000389171
	0.882186236
	0.889786694
	0.944252814
	2.03194E-08
	4.78506E-14
	8.14175E-06
	1.08819E-11
	1.05383E-10
	9.52915E-05
	4.62963E-14
	6.93462E-11
	1.84686E-12
	0.0001511
	8.82988E-11
	4.60743E-14
	2.31917E-07
	1.91924E-12
	3.55955E-07
	5.04768E-08
	0.000122118
	2.4198E-10
	4.39235E-10
	1.29174E-12
	0.086332062
	0.000997766
	3.42836E-07
	2.41741E-05
	4.17209E-10
	4.84305E-11
	5.87308E-14

	1.89334E-11
	3.82712E-07
	3.29956E-10
	0.384852968
	0.278152401
	3.12239E-08
	1.46408E-09
	4.99481E-09
	0.32338287
	8.14115E-12
	2.59804E-07
	3.58543E-07
	4.62963E-14
	0.025847826
	1.32075E-06
	0.081380025
	7.70403E-10
	0.388992774
	0.999979523
	2.5413E-13
	0.092934552
	0.010527554
	1.36524E-09
	4.60743E-14
	1.56208E-13
	7.4485E-07
	3.72614E-05
	4.60743E-14
	0.999569569
	0.974275417
	2.64299E-08
	0.001818993
	1.66515E-10
	0.94925891
	0.665098093

	0.103446428
	0.013256519
	0.997952298
	0.017938575
	0.000182198
	4.9849E-14
	0.924946152
	0.96590544
	0.001373907
	0.940569939
	0.050877005
	3.52281E-06
	0.073107438
	0.008234953
	0.00138032
	2.36153E-10
	2.52855E-09
	1.73904E-10
	0.001039961
	0.000272631
	0.000192232
	0.012835195
	0.680695766
	0.927767289
	0.001634767
	1.88753E-08
	7.03549E-05
	1.10016E-10
	7.02999E-07
	0.363433442
	4.9412E-06
	7.84343E-07
	3.08551E-08
	0.99884597
	6.82334E-11

0.014700689
0.3703354
8.76409E-10
0.633256862
1.12688E-13
0.172689873
6.30275E-09
0.999985936
4.60743E-14
6.9611E-14
0.027446999
0.000336427
1.61594E-06
0.132446844
7.02129E-09
0.00813208
2.35026E-06
1.15163E-12
3.66749E-08
0.305385092
1.14307E-10
4.60743E-14
1.19571E-13
9.87612E-05
2.41521E-09
8.31795E-09
7.89369E-14
0.000136425
0.00024958
8.01227E-10
0.036106125
0.068333419
7.28809E-07
0.939031809
0.000253293

	2.09769E-10
	0.999999965
	1.84018E-08
	0.015373566
	0.865508478
	0.557169069
	0.999987202
	0.002112387
	5.14195E-05
	0.956588039
	0.002533074
	0.755081262
	4.24303E-06
	3.04351E-09
	0.000134331
	0.34023675
	9.0621E-09
	0.988346137
	0.393249982
	0.000575025
	0.000374471
	0.039593786
	0.974065832
	4.01098E-06
	0.221791476
	0.809442931
	0.000487812
	0.891208337
	0.126779146
	0.999999757
	0.005687286
	5.01191E-08
	0.246545769
	1.08091E-08
	0.9121628

0.057547996
0.905507082
0.615715459
0.294587538
0.832248642
5.55871E-08
4.60743E-14
4.7988E-08
7.55382E-10
0.128650497
0.001648609
0.214944866
6.79185E-09
0.997331162
4.60743E-14
0.001491878
0.006571419
0.090264746
0.000388495
2.39554E-06
0.016272483
1.42699E-05
0.410176981
0.045316897
0.013073493
0.003814265
0.002206637
8.89699E-12
2.07484E-06
0.171216578
4.45344E-12
0.006345438
2.50794E-05
0.10910249
1.9803E-12

	0.463784925
	0.005247301
	4.01926E-07
	0.027393682
	5.48116E-05
	0.157275823
	0.022663436
	1.11545E-08
	1.90962E-05
	0.163802756
	6.18215E-06
	4.86034E-05
	0.997775032
	4.4294E-05
	0.000343519
	0.000936489
	0.003575483
	0.028502355
	0.024952166
	0.342679582
	0.123657307
	0.008592889
	0.007303395
	0.838894887
	0.010976317
	0.000955835
	0.008842827
	0.006799176
	0.866428585
	0.794961401
	7.0824E-06
	3.3713E-06
	0.85259772
	0.007848532
	0.867585993

	0.02387797
	0.001517792
	0.298238494
	1.31257E-05
	0.018121703
	1.31617E-12
	0.999337378
	9.27325E-07
	0.215266112
	2.244E-07
	4.62963E-14
	1.02456E-10
	6.13857E-09
	0.000244646
	0.001527818
	0.00052896
	1.02539E-08
	0.70606084
	0.986924824
	0.311691781
	0.999930013
	0.991797783
	3.84404E-08
	0.000652413
	0.999037046
	0.000802262
	0.043386227
	0.049789836
	0.00068986
	0.999768234
	0.003267812
	0.000998069
	0.258433114
	0.001984007
	0.000147016

	0.193015605
	0.170085963
	0.922901162
	0.010181433
	0.000116162
	2.32854E-09
	0.000105911
	0.999021236
	0.99358414
	0.727529308
	0.302495005
	0.366513137
	0.477652408
	1.59169E-05
	8.04427E-07

P-value: (SM_pos_12022024_Newanalyis) / (Water_pos_12022024_Newanalysis)
0.995272109
0.995600677
0.927567352
0.046207042
0.11207904
0.990504483
0.831147098
0.951015655
0.004177255
0.024710287
0.001888668
8.77615E-09
0.60440149
4.18667E-05
0.998650398
0.989262511
0.999268002
0.130675659
0.039261782
0.158301635
0.945706008
0.996170584
0.0227927
0.747923292
0.036354006
0.809524149
0.999999984
0.986387553
0.020316203
0.998918181
0.950821702
0.001526276
0.835691609

0.004043563
1.6845E-06
0.998916886
0.007168299
0.251401846
0.044456418
0.10021385
0.225977009
0.999999465
0.260434507
2.78606E-05
0.929401577
0.902949746
0.999737774
0.617173162
0.011799658
0.868165684
0.024863154
0.876522444
0.9997807
0.791465793
1.36907E-06
0.921511122
4.18E-06
0.999996295
0.999999992
0.958920039
0.999968753
0.035981806
0.999990169
0.155799883
0.238158771
0.357458562
0.351132236
3.91708E-05

	0.018635193
	0.68556693
	0.720785479
	0.015999475
	0.100003571
	1.77733E-06
	0.705007775
	0.000812988
	3.29656E-06
	0.741513687
	3.18445E-05
	8.49003E-10
	0.267611989
	0.00033346
	0.864003311
	0.015605864
	0.475437195
	0.999112442
	0.015462384
	0.016413761
	0.076915624
	0.529481441
	6.26045E-07
	0.000110197
	0.967902319
	0.649650288
	0.999809045
	0.84675345
	0.164602494
	0.989700598
	2.81423E-05
	0.148175825
	0.922010185
	0.954107189
	3.51231E-08

	2.33262E-05
	0.986110994
	0.983710573
	0.999999857
	0.99426104
	0.981887758
	0.722704847
	0.00617861
	0.994391075
	0.720074103
	0.159898027
	0.746321149
	0.243330645
	0.980990538
	0.019750778
	0.038858486
	0.000494252
	0.823859047
	0.999433813
	0.022416546
	0.000212977
	0.483043948
	0.998671143
	0.918683061
	2.77262E-11
	0.99986809
	0.038595428
	0.007703276
	0.264618755
	0.084259632
	0.9948778
	4.47468E-05
	0.099541575
	0.992525165
	0.737669301

	0.080970209
	0.999999639
	0.06049032
	0.942338163
	0.441298602
	0.958191312
	0.960610582
	0.994575075
	0.634308598
	0.797618395
	0.992438736
	0.675319606
	0.000142588
	0.148092908
	0.999999658
	2.61051E-07
	0.076997015
	0.001194597
	0.999979954
	0.90416312
	0.663399485
	0.577364732
	0.28839335
	0.009288135
	2.18553E-08
	6.43214E-05
	0.989105687
	0.999941782
	0.999999966
	0.99999997
	0.000177448
	0.959763434
	0.99315819
	0.003428555
	0.003488857

0.000573276
0.89282302
0.94576759
2.34101E-11
0.028345193
0.999999009
0.143152466
0.262145194
1.66922E-12
4.48263E-05
0.748304476
3.38999E-05
5.85887E-07
8.4651E-07
0.999924095
7.19587E-10
0.997694377
1.53713E-05
1.60427E-13
0.999989863
0.013308612
0.016789048
0.995261492
0.3785646
0.909995939
0.985459718
0.99999216
0.000700641
0.99955763
0.043042743
0.008717362
0.004152059
2.00641E-05
0.994362203
3.34943E-09

	0.993563058
	0.982144195
	0.492304175
	0.813589652
	0.943626927
	2.81959E-07
	3.26465E-10
	0.001525728
	9.78811E-05
	1.05306E-05
	0.23900122
	0.978613297
	0.004414548
	0.111700409
	0.996944906
	0.92346794
	0.012062642
	1.59181E-06
	0.726348933
	0.69281704
	0.000540881
	0.999642953
	0.00302649
	1.15365E-05
	0.025582149
	0.960970343
	0.9989743
	0.044692915
	0.217694433
	5.64615E-05
	0.507813091
	0.986178368
	0.944475206
	0.186506648
	0.999999999

	0.005542593
	0.005659118
	0.999178046
	0.999986366
	0.999215881
	1.51517E-05
	0.000620554
	0.99601755
	0.116918235
	3.32107E-08
	7.03288E-07
	0.009073298
	0.748385958
	2.14823E-09
	0.012629733
	0.000319973
	0.011412825
	0.466033431
	0.986023206
	0.979723665
	0.97232504
	3.53972E-07
	0.908561292
	3.16795E-06
	0.529758937
	0.91104106
	0.193852679
	0.942460791
	4.11309E-05
	0.829195571
	0.000368836
	0.711114425
	0.985889503
	0.914279209
	5.02931E-14

2.53586E-09
0.999967784
0.807239334
0.005188127
3.10202E-07
0.128083256
0.000133576
0.971552892
0.99921875
0.741026077
0.000212363
0.011328966
0.016942349
0.138668126
0.999987055
0.46224301
0.407369457
0.000422957
5.27103E-05
0.552354936
0.928408246
0.0122517
0.848316346
0.072114672
0.208186027
0.01014258
0.986125836
0.142596739
0.511773247
1.33793E-12
0.999990565
0.016231173
0.000848803
2.86993E-13
0.450766398

3.27466E-09
0.901546811
0.556062243
0.756173742
6.97541E-07
0.602784092
6.41012E-09
0.496683167
1.92146E-12
0.005137628
0.000190006
1.49282E-07
9.14676E-08
0.004350592
2.70694E-05
0.065974914
0.000994391
0.42295601
0.081971519
1.22577E-05
8.40994E-13
4.8356E-06
0.008434626
0.115332649
0.999183338
5.01592E-07
0.101095162
0.070843751
2.57104E-07
0.517387533
0.288391607
0.720537705
0.014142991
0.747624881
0.97817312

	0.000188846
	0.939104034
	0.116196227
	0.208872477
	0.367476953
	0.800374038
	0.000212655
	0.689536806
	0.323518919
	1.34939E-05
	0.006790946
	0.957460558
	0.005162201
	0.999999937
	0.970909547
	8.86761E-06
	0.826735289
	0.278693184
	0.023215812
	0.034712675
	1.75923E-09
	0.005612993
	0.381692018
	0.993123847
	5.79186E-06
	0.903138433
	0.010710524
	0.984435032
	0.020568395
	0.136952742
	0.924050988
	0.346146117
	0.991861451
	0.266336928
	5.97766E-10

0.007439043
0.364034435
0.300020565
0.403049893
0.713402303
0.031639956
0.533145276
0.131012197
0.047630403
0.837995762
0.034781956
0.08358686
0.003046628
0.741269963
6.24761E-06
0.999668402
0.999999955
6.32793E-07
0.000675833
0.003722551
8.13793E-14
0.000101329
0.016087228
0.267646301
8.04632E-05
1
0.175547692
1.76002E-10
0.413113519
0.255922674
0.630048253
0.484884232
5.28771E-11
5.49861E-08
0.009760331

	1.62699E-05
	3.86738E-07
	1.40037E-07
	5.56879E-06
	8.53912E-07
	0.677638374
	1.02567E-10
	1.05245E-05
	0.307222629
	2.73123E-08
	0.567726458
	6.80405E-05
	0.000680178
	3.73884E-10
	2.77766E-11
	4.00335E-12
	0.196779294
	0.942083497
	2.13698E-09
	4.65183E-14
	0.133618679
	9.87005E-08
	0.000849397
	1.74474E-09
	2.72972E-07
	4.62963E-14
	0.000139911
	0.927519718
	0.318632975
	0.999987441
	0.000843632
	0.000556562
	1.55348E-05
	0.000229503
	1.52669E-10

	0.93908244
	0.992805184
	0.026119536
	0.007236426
	0.621570257
	2.64635E-07
	1.70341E-08
	9.88982E-09
	0.211254647
	1.05304E-06
	4.11219E-05
	1.38366E-11
	1.72337E-05
	0.012169043
	0.024455038
	0.110275457
	0.002519108
	0.366147719
	0.95157151
	7.60184E-06
	0.773556508
	0.152670056
	0.954071815
	5.02144E-11
	2.74856E-08
	0.999971511
	7.64495E-06
	3.68525E-07
	0.002317901
	0.014788084
	0.925985751
	0.000265279
	0.000314332
	0.987527269
	0.390799803

	0.867053922
	2.53356E-07
	0.884382045
	0.765460049
	0.402573699
	5.84076E-06
	0.007852758
	0.661880724
	0.159266734
	0.372229502
	0.354571067
	0.000353324
	0.92969014
	0.003151016
	0.138642829
	0.001602673
	0.000391904
	7.36211E-10
	0.996520393
	3.56655E-08
	0.073605904
	3.03619E-06
	1.93788E-06
	0.999895
	0.062153866
	0.998921932
	0.001429961
	0.505620351
	0.934958483
	0.999031673
	0.004212463
	0.27429352
	0.997762857
	0.055764345
	0.141795166

	0.000976915
	0.150274721
	1.81316E-08
	0.524810015
	4.60743E-14
	0.680070218
	0.879783316
	0.75395458
	0.518013159
	2.67489E-07
	0.644230518
	0.059936401
	3.44609E-05
	0.999966414
	0.960243485
	1.52474E-05
	0.014190943
	4.74065E-14
	0.03581006
	0.111352158
	5.66729E-08
	0.016473953
	4.60743E-14
	0.999992856
	1.17573E-13
	0.03947378
	0.258139314
	2.85828E-05
	5.87308E-14
	6.80001E-12
	0.828755551
	0.538089642
	0.695116365
	0.000166656
	0.999912683

	1.76598E-05
	0.373923913
	0.799626289
	0.007543053
	0.965797006
	0.334080517
	0.950709365
	0.019810575
	0.835031299
	0.010747886
	0.000408931
	0.999902962
	3.61666E-09
	0.999237502
	0.133014199
	0.991872779
	0.55495791
	0.181506692
	0.004238672
	0.009882486
	1.6198E-11
	0.962498868
	0.608062443
	0.359188877
	0.817746572
	0.711907039
	0.003594852
	0.866104755
	0.999751628
	0.239581434
	0.422352012
	0.000126135
	0.377012138
	3.93556E-09
	0.861578771

	0.20158937
	0.516357912
	0.983563184
	0.440668806
	0.050618624
	0.999994748
	0.525204334
	0.999997648
	1.69312E-05
	9.87496E-09
	0.372789957
	0.997982568
	0.598659382
	0.718146729
	1.28335E-08
	0.751580221
	0.999999988
	0.027668596
	0.419618637
	0.158586035
	0.050021966
	0.260973315
	0.93062365
	0.73547
	0.502827342
	0.007400892
	0.40324646
	0.987662465
	0.000569592
	0.552953678
	0.418568489
	0.22955349
	0.76813899
	0.007731723
	0.010968673

	0.299767039
	2.0367E-06
	0.04939667
	0.233947057
	1.5118E-05
	1.2071E-07
	0.000154732
	4.60743E-14
	1.003E-06
	0.902975358
	2.85364E-11
	5.06895E-05
	0.691557748
	2.55486E-11
	0.986134602
	0.994553815
	3.56967E-05
	0.495161029
	0.000577965
	0.993305367
	9.7928E-07
	0.021520855
	0.012112586
	0.998421223
	0.087309018
	0.009382767
	0.0495265
	0.997730144
	0.999962648
	0.167411371
	0.327107084
	0.01471034
	0.97057206
	1.08769E-12
	0.998671336

	0.995053292
	0.012273711
	6.01181E-06
	0.0063912
	0.040876779
	0.584577459
	0.955676074
	0.000177663
	0.577036137
	0.737037559
	6.74486E-09
	3.29868E-06
	0.007048451
	0.200351965
	0.207286964
	0.00473997
	0.914932904
	0.534383088
	0.075284724
	0.872803545
	0.555792908
	0.945511867
	0.151887352
	0.98679006
	0.002248694
	0.993538822
	2.2036E-05
	0.04414801
	0.000339933
	0.806791465
	0.020939619
	0.270612602
	0.192253446
	0.998501613
	0.340169146

0.74873345
0.020852093
0.999999738
0.00025421
0.273273371
0.084958218
0.999999598
0.987143947
0.001296235
0.999997348
0.615296825
0.999993125
0.406880005
0.00597117
2.71581E-09

Adj. P-value: (Bb_pos_12022024_Newanalysis) / (CM_pos_12022024_Newanalysis)
1
1
1
0.087855222
1
1
1
0.049246863
1
1
1
1
1
1
1
0.730117293
1
3.41414E-11
1
0.010281558
1
6.88319E-09
0.001311584
1.64844E-10
3.41414E-11
1
1
1
0.828833146
1
1
2.00712E-06
1

	1
	9.25133E-11
	0.536991378
	0.00079473
	0.009774792
	0.005287
	8.32703E-07
	1
	1
	0.023537311
	0.232464102
	1
	1.93975E-08
	1.09196E-06
	2.5962E-06
	0.028048176
	0.004136976
	0.003787174
	0.000112637
	2.236E-08
	0.004432949
	1
	1
	0.0681081
	1
	1
	0.273228579
	1
	1
	0.881950486
	8.82681E-07
	0.01154846
	1
	1.53302E-09
	1

1
1
2.11014E-05
1
0.006392027
0.297268807
1
1
3.92234E-11
1
9.99156E-09
3.09689E-06
0.001117683
1.14067E-10
1
0.001017834
0.491497917
0.205894201
1
7.13509E-06
0.349939462
0.552518265
0.000365308
4.29658E-07
0.000236408
0.000337432
0.001989496
0.165025963
2.70569E-07
0.0023725
1
1
0.211466347
1
0.006370835

	1
	0.042737567
	1
	0.621593492
	1
	1
	1
	0.38891763
	1
	0.000995768
	0.249741422
	1
	1
	1
	3.41414E-11
	0.009418424
	1
	1
	1
	1
	0.821126521
	1
	1
	0.63163488
	1
	1
	1
	1
	0.805721289
	6.90717E-07
	0.093731442
	0.053079587
	0.052133542
	0.030462166
	1

	1
	1
	1
	1
	1
	1
	1
	1
	0.022503865
	1
	1
	1
	2.33438E-05
	1
	1
	7.49206E-07
	1
	1
	0.382898089
	1
	1
	1
	1
	0.000467136
	1
	0.009355225
	0.863019893
	1
	1
	1
	0.802497902
	0.711952381
	0.892195167
	1
	0.435598131

0.312569576
1
0.018170581
0.000101412
0.004615778
1
0.375021755
1
0.224330318
1
1
1
9.97582E-09
0.00981996
0.969969467
1
1
0.696224249
6.87647E-11
0.049973545
0.168177129
0.073791839
0.603320668
1
0.27799717
1
0.624459377
1
0.013230092
0.001823926
0.001063096
0.043989344
0.162227348
0.844504817
0.696972583

	0.000214407
	1
	1
	1
	0.935400752
	1
	1
	1
	1
	1
	0.952719308
	1
	0.649406057
	1
	0.198941865
	0.006673324
	0.001793721
	0.125937571
	0.000583293
	1
	0.038336845
	1
	1
	1
	0.000870939
	0.491837106
	1
	1
	0.324538249
	1
	1
	1
	1
	1
	3.23756E-05

0.633303397
1
1
1
1
0.088686293
0.606661334
1
1
8.79366E-06
0.101915013
1
1
0.036962169
0.001064181
0.05661249
0.010645578
0.043102575
1
2.23679E-10
1
0.073893475
1
0.783500034
1
1
1
1
0.114382101
1
0.645696869
2.85332E-06
0.975615224
1
2.76968E-10

1
1.35972E-05
0.631141147
1
1
1
0.000261525
1
1
1
0.194766793
1
1
0.4141779
1
1
0.017576568
1
0.011288165
1
5.29453E-08
0.001253103
1
0.19200311
1
1
1
0.000170194
0.008022841
0.00107338
0.811955802
0.116613983
0.068925519
0.480336088
0.530584385

	0.242464701
	1
	0.000790429
	1
	1
	1
	0.119156751
	1.00972E-05
	1
	0.023644478
	1
	1
	0.063518007
	0.057818736
	1
	1
	1
	0.000174096
	8.31194E-07
	0.02187563
	0.275008895
	0.050988199
	0.931214827
	0.000336867
	0.863000538
	1
	0.334539461
	4.6833E-06
	1.485E-07
	0.000383935
	0.001551571
	0.880275279
	0.000628452
	9.53911E-10
	0.004501236

5.42889E-06
1
1.965E-07
5.52376E-05
1
1
1
1
1
0.170551174
0.014720546
1
1
0.053950483
1
0.334540413
0.089964536
1
0.108789483
1
9.65721E-10
0.193001592
1
0.064022285
0.000401595
1
0.728504769
0.830778674
1
0.191316399
1
1
1
0.001291262
1

	1
	1
	0.690820544
	1
	1
	1
	1
	1
	0.148891913
	0.408065767
	1
	1
	1
	0.06643008
	0.082081803
	0.219649712
	0.181583173
	1.58646E-06
	3.17155E-06
	0.603057369
	0.558410173
	0.056730096
	1
	1
	0.587297982
	1
	1
	0.125948482
	1
	0.757433431
	6.42648E-06
	8.23757E-08
	2.29387E-06
	0.000899704
	0.472048613

	0.022375455
	0.623559912
	1
	0.252803299
	1
	0.265874327
	0.001611823
	1
	1
	1
	1
	0.000898164
	0.000116742
	0.000405597
	1
	1
	0.084926402
	1
	0.000257592
	5.77379E-11
	1
	1.40389E-07
	0.029260253
	0.003050551
	1
	4.1085E-05
	1
	9.07657E-05
	0.821092391
	1
	0.618903436
	1
	0.553709813
	5.98772E-05
	3.89013E-06

0.00017526
0.805721289
1
1
0.549929609
1
0.000328478
0.115563377
1
0.194791488
1
0.057314613
3.41414E-11
6.6101E-06
3.756E-05
3.41414E-11
3.41414E-11
3.54905E-11
1
1
1
1
0.052192656
1
2.73178E-05
3.82558E-11
2.50361E-08
1
1
1
1
3.41414E-11
3.47379E-07
3.41414E-11
0.007155086

1
0.004517387
1
1
0.000651537
1
1
1
1
1
1
1
1
1
1
0.000686103
6.53969E-07
0.978325555
0.000546647
1
1
1
1
1
1
1
1
1
1
1
0.072510399
0.009272503
1
0.010779734
0.179900032

	1
	1
	1
	0.613192229
	0.116404043
	1
	0.05670796
	1
	6.27156E-10
	0.005170904
	1
	1
	1
	1
	1
	0.018741165
	1
	0.00145909
	1
	0.816065992
	2.75468E-06
	3.79925E-05
	6.09293E-06
	1
	1
	1
	5.39257E-06
	1
	1
	9.02978E-05
	1
	1
	0.240567323
	1.74975E-10
	1

3.41414E-11
1
1.50316E-08
0.713574391
1
1
1
1
1
1
1
1
1
0.090565618
1
0.108823009
1
0.02055068
0.362580401
1
0.383407025
0.005972183
1
1
6.63062E-06
8.16723E-06
1
1
0.000896301
1
1
1
1
1
1

	1
	1
	1
	1
	1
	1
	6.59001E-11
	0.011839755
	0.007264819
	0.650479851
	1
	1.47059E-08
	0.098927343
	0.840774286
	8.0531E-10
	0.429548153
	5.85997E-08
	1
	0.025630602
	2.82632E-07
	1
	1
	1
	1
	1
	1
	0.851816189
	0.004823401
	1
	1
	0.000198121
	1
	1
	1
	1

	1
	2.16564E-06
	1.43565E-06
	0.01586936
	1.33418E-05
	0.000199739
	0.000545892
	5.40582E-06
	1.72384E-06
	0.054835773
	1
	1
	0.386745542
	1
	1
	1
	1
	0.544289917
	1
	1
	1
	1
	1
	1
	1
	1
	1
	1
	1
	1
	1
	1
	0.090146407
	0.771666917
	0.13192015

	1
	1
	1
	1
	1
	1
	1
	1.71473E-05
	1
	2.28155E-05
	3.90188E-11
	3.39836E-08
	1.96841E-06
	1
	1
	1
	1
	1
	1
	1
	0.201739251
	1
	0.071496253
	1
	1
	1
	6.73508E-11
	0.276871397
	1
	1
	1
	0.916896804
	0.945039158
	1
	0.373211115

1
1
0.082444255
1
1
0.001149572
0.047592195
1
0.020549158
1
1
1
1
0.076807227
0.234070938

Adj. P-value: (Bb_pos_12022024_Newanalysis) / (Rabbitserum_12022024_Newanalysis)
0.179043165
1
1.09399E-07
0.000629595
0.719313609
1
0.021444375
7.21939E-05
0.046143831
0.721707973
1
0.205287635
0.013924117
0.60814707
0.235171673
0.006438322
1
8.4353E-12
1.32902E-05
1.18127E-10
1
8.4353E-12
7.91209E-05
8.4353E-12
8.4353E-12
1
1
1
0.025291428
1
0.609551854
2.34986E-06
0.08782126

	0.151979416
	8.4353E-12
	6.37014E-06
	9.0591E-10
	0.009644549
	0.009927727
	4.77345E-09
	1
	0.000637638
	0.001001942
	0.0007117
	0.900085804
	1.26459E-11
	7.79042E-10
	2.67521E-09
	0.000318194
	4.21254E-05
	0.000105866
	3.53161E-06
	7.01844E-11
	5.07797E-06
	1
	0.713792015
	1.69286E-05
	0.741257755
	0.010196769
	0.771066473
	0.144783112
	1
	0.150705187
	3.48744E-09
	0.000108276
	1
	2.14717E-07
	0.030441409

	0.000121061
	0.197731594
	0.001864077
	1
	1.29112E-05
	0.000473014
	0.000365204
	1
	8.4353E-12
	0.001315154
	8.71968E-12
	8.4353E-12
	2.32645E-06
	1.76631E-07
	0.653493834
	1.55775E-06
	1.13253E-10
	0.041456721
	0.519730358
	2.71388E-05
	0.105136336
	0.009657422
	1.1161E-05
	7.59483E-11
	1.76121E-08
	1.86992E-07
	9.03375E-08
	6.68822E-05
	8.95003E-10
	6.72992E-07
	1
	0.543827749
	8.24836E-09
	1
	6.71818E-05

	1
	2.02921E-05
	0.047778495
	0.062238099
	1
	0.901915691
	1
	0.007333812
	6.33032E-05
	0.07087194
	0.002220189
	1
	0.000196475
	1
	8.4353E-12
	0.000702429
	1
	1
	8.4353E-12
	0.152062643
	0.000844601
	0.442127881
	0.985424301
	0.052516626
	0.034856181
	0.001761012
	1
	1
	2.1758E-05
	8.48898E-12
	3.58989E-07
	0.544784402
	0.058670684
	0.000134957
	0.536748838

1
0.609199976
0.062180718
1
1
0.969981929
1
1
7.99705E-05
0.870858799
0.753219325
2.37059E-05
8.4353E-12
0.085044514
1.68158E-11
8.4353E-12
1
0.001107468
0.000177383
0.014206209
8.62179E-05
1.69245E-07
8.48898E-12
1.05633E-08
0.313853215
8.64207E-05
1
0.641251958
0.005289154
0.474619663
2.55321E-06
0.016134301
0.588818162
0.0076153
3.89342E-07

1
1.14894E-05
1.03206E-05
8.4353E-12
2.14717E-07
7.88611E-06
0.003745485
2.41283E-07
0.00027262
6.55825E-07
0.310861343
1
8.4353E-12
4.18631E-06
0.003777636
1.75252E-07
5.56416E-06
0.000249333
8.4353E-12
5.06715E-10
1.1135E-06
7.81212E-09
1
1
0.002583933
0.503193006
0.029308489
0.682991593
1.02E-05
4.6088E-08
9.27105E-07
9.5593E-05
5.16043E-07
1.08296E-05
8.80826E-05

	2.56821E-06
	0.004102712
	0.008171016
	1
	0.094965006
	0.773325416
	0.002071525
	0.008534996
	0.664297632
	1
	0.00188714
	0.90593765
	0.005687209
	1
	0.146454777
	0.00329114
	0.009220992
	8.88955E-09
	1.65977E-07
	1
	1.69022E-07
	0.224660622
	1
	8.64398E-06
	8.4353E-12
	6.86609E-06
	1
	0.834565988
	0.030629774
	1
	0.480911282
	1
	0.161589763
	0.278117994
	8.59765E-12

	1
	0.135813212
	1
	1
	0.227063271
	5.88532E-09
	2.32136E-06
	1
	0.011912139
	8.4353E-12
	1
	0.592517398
	0.240397769
	7.55443E-11
	0.433613829
	0.610353843
	1
	1
	1
	8.4353E-12
	1.73058E-05
	0.6146761
	0.916878663
	1
	0.975350335
	1
	0.042364623
	1
	0.210176887
	0.032654342
	1.44577E-05
	1.67791E-10
	0.019165058
	1
	8.4353E-12

	4.04395E-09
	3.67422E-11
	1.86991E-06
	0.292124374
	0.007061873
	0.089444803
	8.53175E-12
	1
	0.400874636
	1.32903E-07
	1
	0.000351672
	1
	1.64023E-05
	1
	1
	3.84566E-10
	0.003412357
	0.000225212
	0.008033362
	3.86002E-09
	0.006705313
	1
	8.4353E-12
	0.000237528
	1.87549E-06
	0.00830618
	8.4353E-12
	1
	1.91001E-09
	0.000651108
	1.77028E-05
	1
	0.006524067
	0.009235311

	1.15808E-10
	1
	1.46536E-07
	0.032780398
	0.012964106
	1
	3.24739E-06
	8.4353E-12
	1
	2.11079E-10
	0.067824232
	0.001342937
	0.000809323
	0.002475036
	1
	1
	0.000113371
	5.67766E-09
	8.4353E-12
	0.000172918
	0.026162483
	1
	0.012410717
	9.07164E-11
	1.28876E-05
	0.322069181
	0.002199676
	0.573041849
	0.00421388
	0.256392215
	1
	0.00027646
	1
	2.81442E-07
	0.046345395

	0.002954646
	0.845592712
	8.00293E-11
	3.64939E-06
	0.001479173
	0.673351186
	6.47658E-05
	3.76972E-10
	0.000260121
	2.09316E-07
	0.516257049
	1.24301E-09
	3.91124E-06
	0.008195171
	1
	0.003790181
	1
	0.000160703
	0.001436855
	0.00110429
	8.4353E-12
	1.57999E-09
	1
	1.14859E-08
	1.48615E-09
	1
	0.008520875
	5.99022E-05
	0.000897336
	5.02002E-07
	0.655419409
	0.000885431
	1.34094E-05
	6.41757E-05
	1

	0.504221322
	0.361875236
	0.00046021
	3.37107E-07
	0.071938771
	9.2221E-07
	1.87974E-07
	4.06106E-06
	8.01335E-08
	1
	0.000980837
	8.0469E-10
	2.18127E-06
	1.97096E-05
	1.66893E-06
	0.000273675
	0.000212688
	1.63925E-10
	1.41333E-11
	0.006750111
	0.004561214
	8.67036E-06
	0.000810342
	2.36926E-08
	1
	1
	0.879743862
	0.004878681
	1
	0.017966351
	3.58306E-11
	4.38455E-11
	8.4353E-12
	3.20129E-07
	0.002920609

3.06887E-07
0.005406812
0.006326286
0.002046674
1
1
0.090248815
0.013186812
2.29472E-05
0.000100039
0.077049005
2.73423E-10
3.74406E-09
4.27359E-05
2.25512E-05
0.0004474
1
0.708275908
2.86797E-10
8.4353E-12
0.000398707
8.7316E-12
3.05288E-06
1.13445E-05
0.299939637
2.65998E-09
4.71537E-07
7.96069E-11
0.022394462
1
0.025295685
1
1.34679E-05
1.81683E-09
2.35544E-11

	1.77576E-09
	1.64846E-05
	1.17477E-05
	0.224615299
	1
	1
	2.65416E-08
	1.92469E-07
	0.304141078
	0.00867535
	0.018668738
	1
	0.000166084
	0.000675841
	0.035430609
	8.4353E-12
	6.91202E-11
	1.92726E-11
	0.437333376
	2.35903E-07
	1
	0.055050871
	0.026369737
	0.000575265
	1
	9.55538E-07
	5.5166E-05
	1.26459E-11
	0.931077892
	0.002853528
	0.000484513
	9.28666E-07
	0.107785841
	8.4353E-12
	0.000548441

1
0.002606925
0.194438326
0.117827624
1
8.61635E-12
0.005506114
1
0.045390577
0.831579573
0.112137585
0.002043858
0.021917557
0.067475926
0.000959811
7.83165E-09
1
6.61658E-06
0.054309364
5.6325E-05
4.84365E-06
0.389761836
0.65127588
1
0.009617852
4.76804E-10
1
5.66454E-06
5.1656E-06
0.497740058
2.42208E-06
8.25697E-07
1.14708E-08
0.005711218
1.94665E-10

	0.014676127
	0.174878712
	3.4617E-07
	3.6221E-05
	8.4353E-12
	0.040856376
	2.5845E-08
	0.613904544
	8.4353E-12
	8.7553E-12
	0.004475205
	4.35613E-05
	0.099233734
	0.046442843
	1.4098E-06
	5.32901E-05
	4.0903E-05
	2.45821E-11
	3.18683E-08
	0.318460335
	2.3172E-11
	8.4353E-12
	8.53175E-12
	0.024673552
	6.70182E-08
	0.026691168
	8.59765E-12
	0.220429455
	6.25833E-06
	1.83271E-10
	0.044999237
	0.002855709
	0.00077626
	1.93953E-11
	0.818688978

	0.006100364
	1
	1
	0.071269933
	0.078989078
	0.318379246
	0.865787899
	0.059954772
	5.50855E-06
	1
	1
	1
	6.70536E-06
	2.29398E-09
	0.000431188
	0.000667562
	1
	0.046778032
	0.936425131
	0.736616442
	4.2806E-05
	7.45777E-05
	1
	0.001006282
	0.000524454
	8.67648E-06
	0.001181005
	0.011700861
	1.60419E-06
	1
	1
	0.00039622
	0.109912861
	1
	1

0.000492944
1
1
0.844808587
1
0.180986089
8.4353E-12
2.91632E-09
1.37789E-07
0.626893903
0.009701444
7.69224E-05
1.896E-09
0.023400434
8.4353E-12
4.7214E-05
0.000529543
1
0.041455772
0.621396512
1
1.17165E-05
1
1
0.048713712
0.991201577
0.056947312
1.00865E-10
0.448324281
1
1.63964E-11
0.000946162
0.000259907
0.22055304
0.000134674

	0.781556326
	1.15771E-07
	1.04332E-09
	1.50434E-05
	1.80757E-07
	2.23257E-06
	1.77782E-06
	8.4353E-12
	4.12036E-09
	0.000156311
	1.18554E-05
	0.433450416
	0.03070583
	1
	0.058811641
	1
	1
	0.001387431
	5.26944E-05
	0.00076803
	1
	1
	1
	0.781942678
	1
	0.888424057
	1
	1
	1
	1
	1
	1
	0.001407801
	9.52663E-10
	0.00135023

	0.24135943
	1
	1
	0.000280529
	0.000113106
	2.07353E-07
	0.056776502
	1.45384E-09
	0.545080044
	1.03917E-08
	8.4353E-12
	8.59765E-12
	3.77369E-11
	1.20807E-05
	0.000736969
	0.133760275
	9.53265E-05
	1
	1
	1
	0.015451735
	1
	1.19007E-05
	0.950115448
	0.594360611
	1.52767E-05
	5.34579E-05
	0.026035802
	0.005860049
	1
	0.006379912
	0.000819211
	0.307762705
	1
	1.14434E-05

1
0.436489058
1
0.341473622
0.044748635
3.46417E-09
5.32116E-06
1
0.36527513
1
1
0.878078318
1
0.00879792
9.28474E-06

Adj. P-value: (CM_pos_12022024_Newanalysis) / (Rabbitserum_12022024_Newanalysis)
1
1
1.02298E-07
1
0.30301488
1
0.288057708
0.540909978
0.008162035
0.423847901
1
1
0.637661456
1
0.388830912
0.847459304
1
2.19578E-05
0.001154752
1.4359E-06
1
0.004430312
1
3.21604E-11
0.001020706
1
1
1
1
1
1
1
1

1
0.044816892
2.12972E-08
0.00028792
1
1
0.886420968
1
1.82522E-05
1
4.71096E-07
1
0.039946627
0.192546222
0.290744162
1
1
1
1
0.466693829
0.467547929
0.8596726
1
5.54279E-09
1
0.11659534
0.002427545
0.99141528
1
1
0.724035668
1
1
0.153235048
0.73018584

	0.002722959
	1
	0.586049899
	1
	0.656339565
	0.453286119
	0.001278768
	1
	0.009480845
	0.000335265
	0.018129698
	9.17195E-08
	0.63945157
	0.005451462
	0.955506365
	0.534648524
	2.47459E-08
	1
	1
	1
	1
	1
	1
	0.030627031
	0.035540326
	0.246859016
	0.027361375
	0.199098869
	0.615111835
	0.161360814
	1
	1
	1.09388E-05
	0.249073185
	1

1
0.260145657
0.312015264
1
1
0.366622212
1
1
0.002548327
0.616202197
1
1
0.043261738
1
0.00710771
1
0.984420447
1
1.78878E-11
0.117190177
0.22468272
1
1
1
0.267528156
0.003792084
1
1
0.008615208
4.54787E-05
0.002410356
1
1
0.929088902
1

1
0.081954159
1
1
1
1
1
1
0.856615068
0.988719077
1
6.60513E-06
4.8881E-11
0.003850019
3.5687E-10
3.85163E-08
1
0.044674867
0.177482205
0.00037285
0.008114859
1.25087E-05
6.70563E-11
0.010344465
0.423380437
1
0.139524234
1
0.190178056
0.163639526
0.000918441
1
0.008997405
0.152534401
0.000333118

	0.08471304
	6.87785E-07
	0.299826067
	1.78878E-11
	0.030996251
	0.00053218
	1
	1.81045E-06
	0.407024667
	1.02074E-05
	0.005447723
	1
	3.83509E-11
	0.239808461
	0.487666359
	1.74977E-05
	2.25548E-06
	0.107929905
	8.99548E-10
	1.9806E-06
	0.00400159
	3.71423E-05
	0.824371176
	1
	1
	0.273976377
	1
	1
	0.370244014
	0.014590711
	0.370840521
	0.672708794
	0.001824428
	0.003887365
	0.042742462

1
0.01278614
0.252132652
1
1
1
0.131598855
0.006563902
0.52773384
0.565679319
0.319503849
1
0.876152283
1
1
1
1
3.85834E-11
0.14687707
1
0.002887573
0.041156066
1
0.000278245
3.47538E-09
0.006115871
1
1
1
1
0.052100553
1
0.005556678
0.183594244
1.20713E-06

0.174614684
0.274012416
1
1
0.502074821
2.11603E-05
0.001382954
1
0.460430479
4.43326E-08
0.012824985
0.087060598
0.029013144
2.4187E-07
0.117968854
0.000198509
0.007249214
0.135245017
1
0.000445131
0.001607966
0.000274503
0.044100011
0.038664981
1
1
0.564959149
0.638267973
7.85993E-05
0.00852624
0.008424112
0.011635461
1
1
4.5701E-10

	7.04159E-08
	0.000201339
	0.001025267
	0.093620591
	0.00687692
	0.952954774
	1.35885E-07
	1
	1
	7.18679E-07
	0.427932454
	9.54137E-05
	1
	3.49969E-08
	1
	1
	3.95702E-06
	0.000371277
	1
	0.007576211
	1
	2.56353E-08
	1
	9.00168E-11
	0.000113695
	1.91283E-06
	0.117022343
	9.97737E-08
	0.11297126
	0.000545934
	0.187592847
	0.093854705
	0.608618824
	1
	1

	6.06263E-08
	0.695199834
	0.102310109
	0.027332267
	0.073397961
	1
	0.018290654
	1.0386E-08
	1
	1.3933E-06
	1
	0.090910298
	1
	1
	1
	1
	0.000215792
	0.013056472
	1.05858E-05
	1.35758E-08
	1
	0.005238515
	0.920313335
	2.77143E-05
	0.004456095
	1
	0.899485698
	0.00029654
	0.002484346
	0.100542448
	0.022546156
	0.081598977
	1.04834E-05
	0.070154353
	1

	0.158968927
	1
	0.075025258
	1
	0.001081829
	0.577707047
	1.51366E-05
	1.62661E-08
	0.000689943
	0.000624437
	3.34113E-05
	1.019E-07
	5.97859E-06
	1
	1
	1
	0.069392524
	5.24031E-05
	1
	1.31122E-05
	1.59951E-07
	1.72525E-06
	1
	7.01057E-05
	0.00109
	1
	0.947309391
	0.021944179
	0.025628228
	0.001432407
	1
	0.066939943
	0.000144518
	1
	0.78974501

	0.186163353
	0.980065433
	1.41146E-06
	1.62198E-07
	1
	9.12268E-07
	7.38498E-06
	3.02243E-05
	0.000244603
	0.430743046
	0.008337088
	4.8806E-10
	1.71573E-06
	0.176095334
	0.014319427
	0.415353522
	0.41282384
	0.021099578
	0.000142155
	0.978859448
	0.887577678
	0.102273295
	0.007609735
	1.44298E-08
	1
	1
	0.240395059
	1
	1
	1
	0.000445394
	0.082527749
	1.5422E-07
	0.184839777
	0.821562264

	0.009699299
	0.883533867
	0.013822695
	1
	0.595614066
	0.157368989
	0.680721308
	0.005058066
	5.29436E-06
	1.88667E-05
	0.038664981
	4.97863E-05
	0.011803226
	1
	2.05694E-07
	0.003604843
	0.022298795
	0.186122667
	0.000190023
	1.04814E-05
	0.008075695
	0.00072903
	0.073778766
	0.865484345
	0.206268414
	0.022226912
	4.8488E-07
	8.88448E-05
	1
	1
	6.41741E-05
	1
	0.010115993
	0.009716969
	0.000371461

	0.003146214
	0.006516768
	1.23103E-07
	1
	1
	0.769424018
	0.040425769
	0.000930228
	0.03450957
	3.85908E-06
	0.02637535
	0.459503759
	6.41431E-08
	0.511029062
	0.104794553
	0.109340115
	1.21813E-05
	1
	1
	4.78673E-07
	1
	0.046377425
	2.67708E-06
	3.5604E-06
	0.000204314
	3.89036E-05
	0.021295731
	5.06053E-11
	1
	0.002476471
	1.52637E-05
	1.28162E-06
	0.000180763
	1
	1

1
1
1
1
0.000603352
1.78878E-11
0.168502027
1
0.548744464
0.045974454
0.229930072
0.005606016
0.026726973
0.269959365
0.010559277
0.005036538
2.33234E-06
5.60154E-08
0.528252036
0.000219294
0.000682178
0.087127183
0.329462255
1
0.073468414
2.71583E-09
0.253089462
1.38659E-06
2.57618E-05
0.942791804
0.02387852
0.061547856
2.47267E-08
1
1.58334E-07

	0.460490037
	0.038399772
	4.65785E-07
	0.022785336
	3.80746E-10
	0.069571417
	0.000212806
	0.089878512
	4.63137E-07
	1.85127E-08
	0.027471645
	0.000839271
	0.005617347
	0.568109775
	1.42445E-06
	0.776037441
	0.004351721
	8.94489E-07
	3.70159E-06
	1
	0.00054233
	3.28571E-10
	5.09617E-06
	0.007090188
	1.14295E-06
	0.021346852
	7.61372E-06
	0.075969391
	2.08368E-05
	0.000298246
	0.009836296
	0.175176136
	5.2859E-07
	1
	1

	1.80046E-11
	1
	1.019E-07
	1
	1
	1
	1
	0.915980125
	3.60255E-05
	1
	1
	1
	0.000310019
	6.47392E-06
	0.062374272
	0.989857416
	1
	1
	1
	1
	0.0524837
	1
	0.155566098
	0.005168387
	0.590164631
	1
	0.18212402
	0.275587409
	0.584524787
	1
	1
	0.003958668
	0.073413252
	1
	1

	0.026816792
	1
	1
	0.587912437
	1
	0.002343549
	9.6392E-08
	7.89787E-05
	0.012533443
	1
	0.154369146
	0.00793178
	4.65575E-06
	0.000104139
	4.66881E-08
	0.049405012
	0.006474043
	1
	1
	9.96011E-06
	1
	0.00121951
	0.908671695
	1
	0.28804756
	1
	1
	2.36988E-06
	0.01606839
	1
	2.4828E-06
	0.012748588
	0.000612771
	0.537149658
	0.000324116

	1
	1
	0.204672439
	0.423730387
	1
	1
	0.787261281
	7.29068E-07
	0.545611754
	0.760167262
	0.000915443
	1
	1
	1
	0.478770706
	1
	1
	0.50268485
	0.002822272
	0.017669944
	1
	1
	1
	0.856632138
	1
	1
	1
	1
	1
	1
	1
	1
	1
	1.52215E-07
	1

	0.471571579
	1
	1
	0.000552054
	0.007358532
	6.748E-08
	0.925550309
	0.027096953
	1
	0.168474764
	0.000173093
	0.002929953
	0.001913804
	0.000732182
	0.001107033
	0.722722437
	2.47427E-05
	1
	1
	1
	1
	0.122849313
	0.108040943
	1
	0.12514382
	0.000594788
	4.22609E-06
	1
	0.006470988
	1
	0.067964783
	0.185853332
	1
	1
	0.015431945

0.402888171
1
0.372738552
1
0.835490987
0.00108201
0.077991527
1
0.916128646
1
1
1
1
1
0.023260335

Adj. P-value: (SM_pos_12022024_Newanalyis) / (Rabbitserum_12022024_Newanalysis)
1
0.057207246
3.21527E-07
1
3.75341E-05
1
0.605480799
0.001104524
0.678572431
0.009155739
0.007131322
1
1
0.354075524
0.052042248
1
0.86623599
6.47189E-12
2.49127E-08
1.90867E-11
0.453203711
1.78209E-10
0.129689814
6.47189E-12
6.47189E-12
0.441051346
1
0.638889914
0.010991217
0.005198503
1
1
0.000369817

0.003174444
2.3124E-07
4.81423E-07
2.03125E-09
1
1
1.61784E-08
0.002609242
1
1
0.313819933
1.80174E-05
5.52E-08
2.15064E-05
2.0437E-07
5.6738E-05
0.011897978
1.75003E-05
0.000764078
5.22775E-07
0.001798544
5.35637E-05
0.351182522
0.682085358
0.488843635
0.367184162
1.39369E-07
1
0.008740858
0.003965239
4.45239E-05
4.42485E-05
0.000210524
0.951202972
0.000795823

	1
	1
	0.002214424
	4.92875E-07
	1
	0.002292986
	1
	0.002419995
	6.48861E-08
	6.49716E-07
	7.22442E-12
	6.47189E-12
	2.41672E-05
	4.6777E-08
	1.34786E-05
	1
	5.59584E-06
	1
	0.920673905
	0.000216541
	0.078261697
	1
	1.31493E-10
	2.55067E-11
	0.001855975
	0.007936833
	8.83381E-06
	1
	2.38655E-07
	0.002559342
	0.647264635
	0.819829145
	1.74172E-07
	8.88831E-06
	8.38111E-05

	0.137163013
	9.80416E-05
	5.50807E-07
	1
	1
	0.200347863
	0.375072973
	0.154618232
	2.32804E-05
	1
	1
	6.68664E-06
	1
	0.599959481
	6.47189E-12
	6.39169E-06
	0.091929673
	0.003508527
	6.47189E-12
	0.005230247
	1
	0.02255293
	0.510937137
	1
	2.73443E-10
	1
	1
	0.951552144
	0.001323253
	6.47189E-12
	7.8242E-08
	1.06531E-10
	0.421331759
	0.344965886
	1

	0.004249694
	1
	0.456276384
	1
	1
	1
	1
	1
	1
	0.954859275
	1
	0.006944115
	4.01261E-10
	0.001454651
	4.02636E-09
	9.97166E-07
	0.424352787
	1
	0.79237611
	0.005927109
	1
	0.00605987
	4.41455E-10
	0.001296371
	9.77084E-05
	1.22935E-06
	4.30198E-07
	1
	1
	0.000431846
	0.005722121
	1
	3.30658E-08
	1
	0.000781542

1
6.2251E-10
0.000136867
2.19386E-11
0.001961518
1.5318E-05
0.896446577
0.045254131
0.013392314
0.774031704
2.43948E-06
6.88854E-09
1.97675E-11
0.124393825
1
0.055269985
0.000846336
0.699093666
3.62027E-08
2.53094E-07
0.042670185
0.000928886
6.22988E-05
0.003758928
0.241617602
0.422188147
0.197640886
0.726853173
0.12502529
2.02614E-09
1
2.75298E-06
0.010019322
1.72347E-08
0.838893404

	0.013091277
	1
	1
	0.873428755
	1
	3.23575E-07
	3.44183E-05
	1
	0.000312837
	0.764473151
	1
	1
	1
	1
	1
	1
	0.00086132
	6.47189E-12
	3.07199E-06
	0.493077247
	0.105412809
	0.025899345
	1
	1
	2.40747E-08
	2.42611E-05
	0.330802976
	0.144900575
	0.87020151
	0.001833071
	0.965562945
	0.257362678
	0.033715692
	0.054013892
	6.73981E-10

	1
	0.414397086
	1
	1
	0.021665767
	0.604818041
	0.005696465
	1
	1
	1.7309E-06
	0.196613259
	1
	0.01512839
	8.3927E-09
	0.881330279
	0.228278137
	9.37278E-07
	1
	0.447911375
	6.47189E-12
	0.106812112
	0.497065774
	0.000395955
	0.008672462
	0.001223714
	1
	0.472437544
	0.83100384
	1
	0.079765795
	0.432836484
	4.61988E-06
	1
	1
	5.70405E-09

	1.79684E-05
	6.45874E-10
	0.184568349
	1
	0.157734383
	1
	2.72375E-07
	1
	0.474914313
	8.54498E-06
	0.095417215
	5.39491E-05
	1
	0.025824803
	0.83032427
	0.143866888
	1.05388E-09
	9.79763E-05
	1
	0.002662431
	2.04818E-07
	6.50156E-11
	0.707605127
	1.62113E-08
	0.000204554
	4.51904E-07
	0.096135294
	1.09889E-09
	0.002050871
	0.000416495
	0.000273461
	0.001887285
	1
	0.007357751
	1

	1.23948E-05
	0.010028635
	1.99122E-05
	7.26149E-05
	0.045142239
	0.812561398
	0.545292168
	7.25829E-11
	8.8691E-06
	2.46972E-05
	1
	0.003898514
	0.636963876
	1
	0.000284449
	1
	0.001545248
	1.80287E-07
	3.70392E-10
	7.22799E-05
	5.69483E-05
	0.441884108
	1
	1.9549E-07
	0.000602024
	5.29369E-05
	0.003590126
	0.000117423
	0.044803204
	0.049291521
	0.416758544
	1
	0.000835113
	1
	1

	0.003324786
	0.943186932
	1
	0.002749492
	0.001459825
	1
	1.0142E-06
	8.62033E-06
	0.003050203
	0.001008503
	1.40925E-06
	2.68533E-07
	7.20361E-05
	0.000727814
	0.784197086
	7.34397E-07
	0.019352493
	0.000178444
	0.537780372
	0.001672928
	7.50452E-08
	0.008097277
	1
	1.10843E-07
	1.80066E-05
	1
	0.669979636
	0.000896944
	0.000549834
	1
	1
	2.68429E-07
	6.33405E-07
	0.000662331
	1.42895E-08

1
0.001867282
1.7899E-10
2.31167E-07
2.73569E-05
6.05883E-07
1.30263E-07
5.30217E-05
1.72384E-11
0.003100949
0.012425155
4.62028E-10
1.88619E-07
5.53045E-05
1
0.000495924
0.014316027
0.002405569
4.19397E-06
0.758747177
0.130136918
1
0.000123689
2.03933E-07
1
1
1.72457E-09
0.07707436
1
0.02050968
2.23104E-10
1
0.007558286
1
1

	0.003463265
	0.000356934
	1
	1
	0.51931329
	1
	2.19214E-08
	7.82337E-06
	3.73743E-05
	7.84079E-07
	0.003399278
	5.43609E-05
	9.44583E-05
	0.002232652
	6.29223E-05
	1
	6.28118E-09
	7.95435E-05
	1
	0.768150418
	0.001259716
	0.002605183
	0.193087215
	1
	0.510948406
	4.79543E-06
	0.001888831
	3.09276E-10
	1
	0.00597296
	0.188821644
	1
	0.012212438
	0.000104303
	0.002460219

	3.39711E-09
	9.7409E-07
	1.07015E-05
	0.794589606
	0.028974087
	1
	1
	1
	0.006044967
	0.001683989
	0.796205214
	0.008427706
	4.43127E-10
	1
	0.037510104
	1
	0.000250995
	0.017994384
	1
	2.93098E-06
	0.013666485
	1
	1.53189E-07
	1.61246E-06
	0.000226275
	4.24757E-06
	1
	9.30431E-12
	0.018159384
	0.00808524
	3.02831E-06
	4.51994E-08
	0.000316053
	1
	0.055585822

1
0.016005654
1
0.580901992
0.084668674
2.21865E-08
0.194945116
0.465235001
1.17174E-05
0.172663833
0.001263233
0.948308505
0.024631713
1
0.773467785
0.000105652
0.004730229
1
0.001309736
0.075208645
0.464887306
0.12492042
0.000589059
1
1
1.10392E-07
1
1.16202E-10
5.90429E-05
0.968973434
0.371145546
0.001477645
1.03163E-06
0.066440163
3.51397E-07

	1
	1
	1
	0.080537767
	0.002100673
	0.019365766
	1.07473E-06
	1
	6.47189E-12
	3.26254E-06
	0.876581563
	0.682145041
	1
	0.405462094
	6.42589E-07
	0.469080653
	0.095371196
	0.028510213
	0.000963755
	1
	0.296934732
	6.47189E-12
	0.000225089
	0.000730632
	0.001448507
	0.000197418
	9.58716E-11
	1
	6.72754E-09
	0.559009312
	0.711264837
	0.003812217
	2.27794E-07
	0.007899599
	0.002107787

	6.47189E-12
	0.665155525
	4.42688E-06
	1
	1
	1
	1
	1
	0.005592551
	0.195011056
	1
	1
	0.23356109
	2.41354E-08
	0.240024489
	0.282305254
	4.07489E-09
	0.130741408
	0.622315192
	1
	1.91869E-05
	0.017958756
	1
	0.004291707
	0.046186724
	1
	1.24243E-07
	1
	0.177647259
	0.446309749
	0.669455657
	0.171205107
	0.00953702
	1
	0.579180587

	0.000620402
	1
	1
	1
	0.820859991
	4.84001E-07
	6.47189E-12
	4.43778E-07
	0.020620456
	7.62796E-10
	4.69719E-05
	0.225199107
	3.71951E-09
	0.79373368
	1.37837E-09
	0.123856431
	0.023995177
	1
	1.5879E-05
	0.008630607
	1
	0.020736197
	1
	0.005353051
	0.000586483
	1
	7.03266E-05
	9.99501E-10
	0.662620342
	1
	5.98167E-09
	0.998629499
	0.004093186
	3.58377E-05
	9.9038E-08

	0.017952828
	2.84049E-09
	5.12034E-09
	1
	4.94813E-10
	0.000549549
	0.790518576
	7.20752E-10
	1
	0.049769628
	6.47189E-12
	1
	1
	0.000218709
	0.007755053
	0.013826968
	0.940596836
	1
	1
	1
	0.005289169
	1
	1
	0.997648563
	1
	1
	1
	0.060535857
	1
	0.029589658
	0.008217831
	0.120965671
	0.747617485
	6.47189E-12
	1

	0.021795795
	1
	2.83711E-07
	0.49553053
	2.73399E-05
	1.07641E-09
	1
	1.72318E-10
	1
	6.17947E-05
	6.47189E-12
	6.47189E-12
	6.53975E-11
	3.53377E-06
	1.81937E-05
	1
	1.35981E-06
	0.106236506
	0.525838523
	0.09235746
	1
	1
	0.00018872
	0.013225621
	0.003503385
	0.012684397
	9.55646E-08
	1
	1
	1
	2.83408E-06
	0.415984815
	1
	0.019615783
	0.091624605

	1
	1
	1
	1
	0.102161507
	2.30457E-05
	0.000457764
	1
	0.019076229
	1
	1
	0.587363342
	1
	0.564492211
	0.510692608

Adj. P-value: (Bb_pos_12022024_Newanalysis) / (SM_pos_12022024_Newanalyis)
0.581839143
0.013131087
1
0.010730755
0.005421576
0.848748247
0.000134473
1
0.00039272
0.465162478
0.016021774
0.728689549
0.046334706
0.004538509
1
0.027870223
1
0.152559682
0.305448957
1
0.172153945
0.02944133
0.077933027
0.00802714
3.41871E-06
0.713789742
1
1
1.44737E-06
0.019656152
1
1.96362E-06
0.491953195

	0.929942821
	1.90321E-07
	1
	1
	0.002311836
	0.000444653
	1
	0.018448171
	0.01175338
	0.020139977
	0.183397439
	0.001502392
	0.00098811
	0.001500849
	0.395052058
	1
	0.346310947
	1
	0.371178266
	0.002328694
	0.276035445
	0.00052651
	0.006242901
	0.001699556
	1
	0.808679778
	1.55004E-05
	0.137814001
	0.004453402
	3.91712E-06
	0.004716367
	1
	0.000125575
	7.71403E-06
	1.7616E-07

3.97193E-06
0.291965585
3.47932E-08
9.80141E-07
1.36528E-05
1.17589E-08
3.37806E-05
9.70603E-05
2.06979E-08
0.138945466
1
0.001763454
1
1.40652E-11
0.002495699
6.20836E-07
0.000410125
0.036701104
1
1
4.92472E-05
0.048874128
0.000887608
1
0.000958804
0.003341224
0.456717064
0.001566987
0.131369646
0.036140918
1
1
0.873874587
0.000216281
2.59038E-10

	0.813837789
	1
	0.003841866
	0.01050599
	0.232672488
	1
	1
	6.20615E-06
	1
	0.182335378
	0.000106447
	0.000200097
	0.000126856
	0.087050726
	1
	0.856857867
	0.480047453
	0.006374336
	0.060181762
	1
	4.7105E-05
	9.78564E-05
	1
	0.219373293
	8.44228E-07
	0.00060827
	1
	1
	0.779285044
	0.004094777
	1
	1.17915E-08
	0.000220212
	0.040190623
	0.258433039

	0.00098811
	0.910800574
	0.00026888
	1
	1
	1
	0.945067665
	1
	0.000202368
	1
	1
	0.335967568
	1.05353E-07
	0.921573396
	0.040662179
	5.95704E-09
	0.720220149
	6.07082E-05
	2.11679E-06
	1
	0.000618071
	0.003896285
	0.024779671
	0.000730977
	3.87522E-07
	0.993767579
	4.6567E-06
	0.173505666
	0.076300919
	0.099235005
	0.063840878
	0.300942079
	5.82086E-06
	0.016244049
	0.059342399

1
0.007000928
1
4.23061E-07
0.014541977
1
0.144792366
0.000735491
0.876820444
4.25198E-05
0.001648565
1.64147E-08
1.95074E-09
0.004866589
0.006683503
0.000417801
0.469477432
2.24152E-06
1.40652E-11
0.066423061
0.004050467
0.000705541
2.0596E-05
0.005082506
3.93793E-06
1
1
0.019436642
0.012011093
1
3.10442E-06
1
0.007808676
0.262054566
1.2593E-06

	0.030674349
	0.021140847
	0.001294035
	0.12377521
	0.007293041
	1.24428E-08
	1.67587E-09
	0.060983755
	3.5888E-06
	0.186440241
	0.047891677
	0.117575231
	0.000212165
	1
	0.042175141
	0.000147718
	1
	0.000247755
	1
	0.5309602
	0.000192988
	1
	0.853489963
	7.80907E-07
	1.06157E-05
	1
	0.195867228
	1
	0.000444425
	0.011381994
	1
	0.819594915
	1
	1
	0.025865601

	1
	1
	1
	1
	1
	4.52352E-07
	0.058725134
	1
	0.000804661
	3.94148E-08
	0.548597298
	1
	1
	0.177158671
	0.013458321
	1
	1.18852E-06
	1
	0.668264011
	1
	0.023979391
	0.007860144
	0.025503581
	0.000368671
	0.056372801
	1
	1
	1
	1
	1
	0.003279219
	0.000871903
	0.004454941
	1
	1.58001E-11

0.013350063
0.665475581
0.001302372
0.084864987
6.11719E-06
0.795542393
1.88322E-05
1
0.003438267
0.594439726
0.383301169
1
1
5.43098E-09
0.280566516
1
1
1
5.22568E-06
1
0.518196164
6.33074E-07
0.147813074
2.72083E-05
1
1
1
0.005114801
0.069030773
0.000268485
1
0.599188186
1
2.76782E-07
0.03407606

	0.00019198
	0.043701771
	0.39002607
	0.380083971
	1
	0.951342345
	0.000466054
	0.000726154
	1.14573E-06
	0.00022857
	0.033644994
	1
	5.66035E-06
	0.000315416
	0.00104629
	1
	1
	0.712819767
	0.010060807
	1
	1.85879E-08
	0.958787057
	0.020297692
	0.009573444
	0.850130662
	0.029861231
	1
	1.15093E-06
	9.85249E-07
	9.55131E-05
	0.026821252
	0.001720303
	0.02582139
	3.89139E-08
	0.0110932

7.04289E-08
1
4.09564E-11
0.156599333
1
1
1
0.001408969
1
0.025836171
0.000406099
0.164115088
1
4.94926E-08
0.20289394
0.063596958
0.014219413
1
0.1624189
1
3.58508E-10
1.26614E-05
0.257842346
1
0.004019867
1
0.428601515
1
1
3.73172E-06
1
0.087820954
1
1
1.89417E-09

0.843949142
6.35425E-06
3.21843E-05
1
0.09583959
1
1
1
0.006463896
0.001562626
1
1
1
1
3.30443E-05
1
0.772710825
2.27088E-06
1.44112E-05
6.38918E-05
0.986249816
2.03888E-06
1
1
1
1
1.03891E-07
1.96043E-06
0.611140897
1
1
6.25224E-10
8.98891E-11
5.04439E-06
0.003557162

	0.012462325
	2.00797E-08
	0.004559711
	0.000456158
	0.038844646
	1
	5.24762E-05
	0.161472644
	1
	0.800310266
	1
	0.000152154
	0.002536587
	1.78817E-09
	1
	1.63221E-05
	2.37215E-08
	0.011336687
	4.52356E-09
	1.40652E-11
	1
	1.04383E-08
	0.002051215
	4.88851E-07
	0.002474382
	1.40652E-11
	0.033444713
	1
	0.088514428
	0.000255348
	1
	1
	0.144655649
	0.000943015
	1.49347E-07

	1
	1
	1
	0.003916948
	0.353022691
	1
	2.17507E-07
	3.34617E-07
	0.824250996
	1
	0.55604643
	0.191152291
	1.40652E-11
	0.001233534
	6.61486E-06
	1.40652E-11
	1.40652E-11
	2.01861E-08
	0.940668685
	1
	0.251003822
	0.428651657
	0.001877654
	0.479088958
	1.76584E-05
	1.40652E-11
	0.000186193
	1
	0.469618283
	1
	0.73359592
	1.40652E-11
	2.918E-07
	1.40652E-11
	0.620495833

	1
	2.42888E-07
	0.049799632
	1
	0.049772996
	0.000443949
	0.888963606
	1
	0.074554153
	1
	0.769065764
	0.076365363
	1
	0.028026376
	0.06374432
	0.005549136
	0.000442272
	2.46082E-05
	4.72763E-07
	3.70691E-08
	0.000930594
	0.000520386
	6.05839E-06
	1
	0.179865635
	0.132664723
	1
	0.001518467
	1
	1
	0.000648054
	0.069789903
	0.409140578
	1.94429E-06
	0.014673547

	0.001168243
	0.057129061
	5.03877E-06
	0.06350596
	1.40652E-11
	1
	0.679194347
	1
	0.004722203
	2.89921E-06
	0.17491708
	0.004443086
	0.589881236
	0.00015841
	1
	2.78207E-07
	0.059738506
	1.40652E-11
	0.003517067
	0.663375869
	2.17929E-09
	8.53772E-09
	1.40652E-11
	1
	2.03027E-11
	0.730431963
	0.244712533
	0.069788993
	1.40652E-11
	1.78827E-11
	0.949062083
	1
	0.084456011
	1.40652E-11
	3.3025E-05

	1.40652E-11
	1
	2.63961E-07
	0.035846244
	0.138179728
	0.15436832
	0.355298485
	0.282348488
	0.127838415
	0.347648872
	0.611085927
	1
	1.9337E-08
	1
	0.166720125
	0.196027097
	4.65566E-08
	1
	1
	1
	7.45555E-11
	0.384670008
	0.546161172
	1
	1.60019E-07
	5.13208E-06
	0.032640248
	0.008923852
	0.001158833
	0.081539101
	1
	0.218189672
	1
	1
	0.481239888

1
0.663454922
1
1
0.075191361
0.000664418
8.13753E-05
0.240198948
0.000900956
8.5954E-08
0.635563633
1.50856E-07
1
0.000261852
3.3055E-09
0.051817075
8.84757E-08
1
0.104500615
6.94201E-05
1
0.082315299
1
0.075780365
0.850641528
1
0.243475915
0.984303613
1
1
0.02450609
0.033298795
1
0.035117577
0.168537125

	0.000240766
	1
	1
	0.000143692
	0.258441018
	8.23636E-11
	3.29442E-08
	1.40652E-11
	7.16717E-10
	0.311646167
	7.55555E-09
	0.429350752
	0.214433578
	5.41835E-05
	1
	0.00053746
	0.148280227
	0.039024003
	1.77018E-06
	0.022028341
	0.000393181
	1
	1
	1
	1
	1
	1
	0.696566338
	0.305726779
	0.014085919
	0.233574742
	0.406552676
	0.094300545
	0.001128864
	0.00664256

	3.72865E-05
	1
	3.51427E-07
	0.046492502
	1
	0.45368484
	0.14193125
	1
	1
	0.012648046
	8.0048E-05
	0.623849699
	1
	1
	1
	0.484727762
	0.994907825
	0.468346386
	0.355831862
	0.218977889
	0.000438609
	1
	1.3194E-10
	0.369329487
	0.331928152
	0.15522646
	1.41411E-11
	0.020646996
	0.002816097
	0.201765813
	0.131079615
	0.147134858
	0.219373293
	0.194363225
	0.019051856

	1
	1
	1
	0.022047304
	1
	0.008714581
	0.633642067
	1
	6.03602E-05
	0.597755784
	1
	1
	1
	0.530574977
	6.74139E-08

Adj. P-value: (CM_pos_12022024_Newanalysis) / (SM_pos_12022024_Newanalyis)
1
0.380085273
1
1
0.05454373
1
0.003873774
0.333199459
9.78775E-05
1
0.095416123
1
1
0.238383708
1
1
1
7.6992E-11
0.013126995
0.000732679
1
1.04491E-05
0.650274105
3.09895E-07
2.52171E-09
1
1
1
0.000708552
0.087281273
1
1
0.021412605

	0.092961577
	0.004021433
	1
	0.002750229
	1
	1
	4.14816E-06
	0.011975162
	0.000586804
	1
	0.000266133
	0.000766564
	0.001000021
	0.058548471
	0.000401457
	0.004934026
	0.410259323
	0.000693021
	0.020787141
	0.000529542
	0.525514215
	0.013859509
	0.245698176
	5.6022E-07
	0.965808191
	1
	0.03883933
	1
	0.004718383
	0.001761881
	0.016793826
	0.004609884
	0.000101856
	0.004747605
	1.42361E-05

0.000130316
1
0.46675801
7.22926E-07
0.740403939
1.61717E-05
0.000188233
0.009323658
0.005040436
0.578722735
1.3025E-08
1.92324E-10
0.012218122
0.006064997
0.00301038
0.31071349
0.281893208
1
1
5.49754E-05
0.079514855
1
4.31502E-09
1.15002E-07
1
1
0.180541903
1
0.00014531
1
1
0.987377882
1
0.019760714
7.62006E-06

1
0.1403155
0.001004813
1
0.614682623
1
1
0.010334879
1
0.287987285
0.214550637
0.000112869
0.03541977
1
3.21958E-11
0.000101123
1
0.001984187
0.075113807
1
0.022752396
0.00072055
1
1
2.78331E-07
0.001869454
1
1
1
1.21359E-10
0.022048518
4.9916E-11
0.971942391
1
0.859412388

	0.002972305
	0.185165007
	0.029117655
	1
	1
	1
	1
	1
	1
	1
	1
	0.174458247
	0.873471311
	1
	1
	0.979305927
	0.982781988
	0.003704701
	0.003566022
	1
	0.062806566
	0.307221229
	1
	1
	9.09105E-07
	0.000153454
	0.002180119
	1
	1
	0.567012062
	1
	1
	0.002578532
	0.31103742
	1

	0.13850162
	0.171252067
	0.153889946
	0.889420655
	1
	1
	1
	0.010160454
	1
	0.001315284
	0.238383708
	3.71193E-09
	1
	1
	0.734303226
	0.06725895
	0.338885482
	0.001508227
	0.567725115
	1
	1
	1
	0.017401083
	0.000618524
	0.010244006
	1
	1
	0.906150982
	1
	6.63775E-05
	0.826374901
	0.001437114
	1
	0.002851188
	0.000830041

0.420469396
0.073559795
0.065414272
1
0.793417462
3.74852E-08
9.34722E-08
0.059377888
3.5618E-06
0.016666098
1
1
0.120284719
1
1
1
0.000162034
0.647138713
0.014029604
1
1
1
0.490207303
3.86403E-05
1
0.860545017
0.266783293
1
0.445516645
0.018968559
0.832581074
0.419976118
1
1
0.114833721

0.893468602
1
1
1
1
0.00430088
1
1
0.067212818
0.834430611
1
0.798789256
1
1
1
0.120156703
0.087473125
0.352897439
1
2.09823E-09
0.830921902
2.55296E-06
1
3.02867E-06
0.048894017
1
1
1
0.002342246
1
0.748242873
0.216730976
0.587288015
1
0.986971698

0.29673624
0.000955815
0.460958685
0.026346074
1.02555E-05
1
1
1
0.103648122
1
0.00057718
1
1
9.88091E-11
1
0.364364332
0.063970082
1
0.288620596
1
3.73298E-06
0.273546688
0.273281094
0.094929365
1
1
1
0.973008972
2.54891E-06
1
0.391886587
1
0.132371597
0.000301603
1

	0.335645202
	0.852454037
	0.106023215
	0.684338037
	1
	1
	0.897557631
	0.609362842
	1.4005E-06
	1
	1
	1
	0.073664388
	1
	1.3053E-05
	1
	1
	0.010719022
	0.007622057
	0.019092252
	3.04458E-05
	0.571867479
	1
	0.987467282
	1
	0.000517179
	0.349695806
	1
	1
	1
	1
	0.373533025
	0.832088543
	0.407138201
	1

1
1
0.016417447
0.031637743
1
1
1
0.139296014
1
1
1
1
1
0.000641955
0.202332273
0.000122973
1
1
1
0.672625981
1
0.048476798
1
0.461845123
1
1
1
1
1
0.015373062
1
0.00307318
0.85815285
0.010967885
1.35043E-09

0.413510841
6.10646E-05
0.021197057
1
0.003229801
1
1
1
4.57006E-06
0.743938205
1
1
1
0.137553705
0.248003775
0.266356837
1
1
1
0.048729924
1
0.031880263
1
1
1
1
2.77373E-06
0.013081994
1
0.599892759
0.000116159
0.82546396
0.004334859
1
0.964729561

	1
	1.05115E-05
	0.013012418
	0.557182551
	0.008955992
	0.075446111
	1.54424E-09
	0.541006346
	1
	1
	1
	1
	1
	0.001310473
	0.303221017
	0.000202098
	0.000164031
	0.171194063
	0.006486849
	0.001371426
	1
	1
	1
	0.110596149
	0.001857048
	4.20364E-09
	0.049361166
	0.000816137
	1
	0.06983972
	0.058963312
	1
	1
	1
	1

	0.000551205
	0.099198027
	0.612082381
	0.491185463
	0.002089524
	1
	0.297873681
	0.002305198
	1
	0.318323945
	0.755615046
	5.52046E-05
	0.736106894
	0.330742814
	1
	0.149756139
	1
	0.002126778
	1
	1
	0.635149016
	0.426942186
	1
	1
	1
	1
	0.006690429
	1
	0.057844821
	1
	1
	1
	1
	1
	0.308033329

	1
	0.040305904
	1
	1
	0.591181912
	0.000482433
	1
	0.282234454
	0.008238955
	1
	0.766553143
	0.208265569
	1
	0.144027442
	0.46708544
	1
	0.099380711
	3.75926E-07
	0.382007008
	2.14716E-07
	0.135644504
	0.000101508
	3.10653E-06
	1
	0.805313846
	0.638581965
	1
	0.012240684
	1
	1
	1
	1
	0.779053064
	0.134281312
	1

0.07634077
0.013312527
1.3563E-05
1
3.21958E-11
1
0.919200058
0.675215378
3.20446E-06
0.32189494
0.685505567
0.089677828
0.072498754
0.005579733
1
0.012533726
1
1.17492E-09
0.438836086
1
0.195029029
3.21958E-11
1.70595E-10
1
1.87674E-10
1
0.001717704
0.024819627
3.21958E-11
3.30559E-06
0.500228699
1
1
0.003582289
0.000620209

	1
	1
	0.847665062
	1
	1
	1
	1
	1
	0.619155028
	0.009222997
	0.657739623
	1
	9.92014E-07
	0.670429374
	1
	1
	1.67081E-08
	0.040578225
	0.099447504
	1
	2.47474E-08
	0.465823942
	0.029301185
	1
	1
	1
	0.000438458
	0.254214194
	1
	0.21270188
	0.703473503
	0.945222915
	1
	1
	0.23349817

1
0.876218087
1
0.831573594
0.151720201
0.125254732
4.35569E-06
0.934880338
1
1.48088E-09
0.138689505
1
0.201697644
2.50008E-06
1
1
1
1
1.19429E-05
0.202532414
1
1
1
0.228936139
0.428270251
1
0.002623757
0.095158714
0.714924707
1
0.466531955
0.333720489
1
0.023369535
0.147193895

0.004041927
6.54609E-08
1.16917E-05
1
3.66868E-08
3.85297E-05
0.032515024
3.21958E-11
0.08836688
1
7.0499E-10
1
1
0.000335542
1
0.000831567
0.109908808
1
0.000131244
0.355411211
0.015706223
1
1
1
1
1
1
0.476246015
1
0.154544282
0.33162818
0.7977459
1
8.21587E-06
1

0.00014302
1
3.66631E-07
0.105321664
0.835109687
1
1
1.80399E-06
1
0.156252738
3.21958E-11
5.77401E-10
5.81838E-06
0.925833368
1
1
1
1
0.390362826
0.458451211
0.641140163
1
3.07581E-07
0.309453807
1
1
1
1
0.004258245
1
0.029971518
1
1
0.080366574
1

	1
	1
	0.074214077
	0.301299175
	1
	1
	0.899421599
	1
	0.858634941
	1
	1
	0.95620606
	1
	1
	0.000165885

Adj. P-value: (Bb_pos_12022024_Newanalysis) / (Water_pos_12022024_Newanalysis)
0.316108156
0.061679804
1
1
0.834461044
0.458187388
1.90457E-05
0.607035013
1
1
1
1.10209E-08
0.750249948
1
1
0.133789322
1
1
1
0.086367727
0.71353285
0.113486261
1
0.18631824
0.011310085
1
1
1
0.00817435
0.067417069
1
0.077597725
1

	0.457671362
	0.633908958
	1
	0.009779899
	0.315235813
	0.400254536
	0.061531868
	0.953175652
	0.018276904
	0.921554537
	0.179793255
	0.02500678
	0.020625875
	0.005925213
	0.031650525
	0.152359721
	0.063092285
	0.252568472
	0.070803006
	0.00873056
	0.036285408
	1
	0.080845953
	1
	1
	0.869027554
	0.000243069
	0.129758992
	1
	1.26579E-05
	0.664743947
	0.654587319
	0.026004262
	2.23882E-07
	0.109500612

	0.023489216
	0.02703907
	1.4949E-06
	0.006810545
	0.015869608
	0.07205212
	0.002151239
	1
	0.081156756
	0.013963417
	0.000251465
	0.004461039
	0.086367727
	1.20383E-10
	0.052553637
	0.004351243
	0.045184655
	0.10835337
	0.179087108
	0.009638179
	0.05880559
	0.00200698
	0.734463566
	0.004375144
	0.011869647
	0.131222033
	0.686732776
	0.039520497
	0.001091356
	0.017477345
	0.00822161
	0.215959208
	1
	0.003417284
	0.020641144

	6.55241E-06
	1
	0.031333707
	0.022268937
	0.11626431
	1
	0.394770634
	0.076853261
	1
	0.017617098
	0.055162004
	0.009974618
	0.04151689
	0.357950595
	0.006838942
	1
	0.356220301
	0.124702414
	0.04817479
	0.785877816
	1
	0.01321107
	1
	0.049180011
	0.148644581
	0.002291342
	0.323558373
	0.300088541
	1
	0.845926685
	1
	0.006872559
	0.14266689
	0.021091855
	1

	0.438293381
	0.886284834
	0.238168362
	0.53785278
	1
	1
	1
	1
	0.015143768
	1
	1
	1
	0.029828802
	1
	0.05444188
	0.131915706
	1
	0.79116554
	3.54023E-06
	1
	0.035363379
	0.177155431
	0.944325884
	0.989992322
	1
	0.032026708
	4.3382E-05
	0.294257647
	0.098216167
	0.143101328
	0.871897182
	0.090983608
	4.79304E-05
	1
	1

	0.008678928
	0.102136214
	0.592690905
	0.220005188
	1
	1
	1
	0.141656105
	3.42424E-11
	1
	0.058318536
	0.012471308
	0.025098479
	0.342588353
	0.020519728
	0.014587803
	0.277146532
	0.887832608
	0.488946922
	0.073224905
	1
	0.811353005
	0.00015307
	0.353162612
	9.04734E-05
	1
	1
	1
	0.040535673
	0.841541963
	0.03404332
	0.263080559
	0.98750171
	0.624470372
	1

	0.016908067
	0.122774397
	0.102363593
	0.843856428
	0.001867506
	0.241229521
	1
	1
	0.555517637
	0.098112367
	1
	0.454020309
	0.822409124
	1
	0.144368059
	0.00309813
	0.261944286
	1
	1
	1
	1
	1
	0.452997178
	0.608077078
	0.042267924
	1
	0.1300264
	0.793270869
	0.11907191
	1
	1
	1
	1
	1
	0.040951571

	0.009556079
	0.120694121
	1
	1
	1
	0.385580342
	1
	1
	0.299892088
	1
	1.98277E-07
	0.187642143
	1
	8.42379E-05
	6.97046E-06
	0.000823936
	0.011129695
	0.128087062
	1
	1
	0.152269171
	0.14781159
	0.241663558
	1
	0.907732592
	1
	1
	1
	0.020778639
	1
	1
	0.040210108
	0.002025449
	1
	1

	0.00176691
	0.57327628
	0.039846281
	1
	1
	1
	1
	0.901132439
	0.014914094
	0.079320953
	0.28384492
	0.164715043
	0.342650815
	2.18271E-06
	0.257831078
	1
	0.258338983
	0.067625323
	0.841252329
	1
	1
	0.005549347
	0.02307958
	0.039022316
	0.418060913
	0.103167376
	1
	0.724827326
	0.002744699
	2.65347E-05
	1
	1
	0.002096606
	0.003880569
	0.830095028

0.09205768
0.008392045
1
0.046719984
5.25489E-06
0.119635772
0.077546989
0.065653177
0.009595881
0.802677388
1
1.77739E-06
1
0.947674473
1
0.037773256
0.101145362
1
1
0.000158456
0.184157281
2.90613E-06
1
0.997408805
1
0.066841267
0.169564994
7.74418E-09
1
4.25932E-06
0.000385062
0.065520164
1.4545E-05
6.4525E-09
0.004267765

	0.016356299
	1
	6.33701E-09
	0.001834195
	0.631887006
	0.784916991
	0.065890988
	0.061975076
	0.12297662
	0.506804566
	1.69255E-07
	0.656423262
	0.384213871
	9.92485E-08
	0.067445275
	0.220263062
	0.218264199
	0.452200348
	1
	0.212324182
	0.252352826
	0.139875059
	1
	0.770315787
	0.872865258
	1
	1
	1
	0.09972941
	0.003134646
	0.806269345
	1
	0.973385801
	0.103746231
	1

0.694326484
0.001485314
6.69033E-07
0.850487739
0.882137478
0.104758825
1
1
1.15172E-05
0.000206475
0.701064149
0.240706181
0.103667187
1
1
1
0.845518558
1
0.501514074
0.504931157
2.45358E-11
0.389757965
0.211949796
0.077604893
0.026859431
1
4.85388E-05
0.305706481
1
0.462653653
0.181684755
7.18891E-11
0.639453935
1
1

	0.787662126
	0.292005125
	0.121473604
	1
	0.077183295
	1
	0.015176214
	0.112054997
	0.940290076
	3.11055E-08
	0.194885566
	1
	1
	1
	1.26472E-08
	0.001498877
	6.67268E-10
	0.111426309
	1
	0.530691677
	0.742845844
	0.381555343
	1
	1
	0.278415021
	1
	1
	1
	1
	0.000392269
	0.065024895
	0.005770405
	0.156782071
	1
	1

1
1
0.14586897
1
1
3.93842E-05
1
1
1
8.62737E-06
0.068884923
5.43046E-07
2.45358E-11
7.33761E-07
1.45257E-08
2.45358E-11
2.45358E-11
1.11008E-09
0.373854654
0.004011627
1
1
0.000535353
1.29214E-10
2.45358E-11
2.45358E-11
8.66608E-10
1.46275E-05
0.736111732
0.025480696
0.214483578
4.50441E-10
7.60894E-11
2.45358E-11
0.029013961

	0.826179396
	1
	0.421292287
	1
	0.001248966
	1
	0.001054026
	1
	1
	1
	1
	0.984516704
	1
	1
	1
	1
	2.21639E-08
	0.13884919
	3.22589E-06
	1
	0.44925227
	1
	1
	1
	1
	0.090095855
	0.008555252
	0.110434314
	1
	1
	1
	1
	0.759814189
	0.004205956
	1

	1
	1
	1
	0.002627342
	1
	1
	1
	0.539372621
	0.235159866
	1
	1
	0.981836965
	0.056648478
	0.000228105
	1
	0.27235018
	1
	1
	1
	0.007186317
	0.140977454
	2.99133E-05
	1
	1
	1
	0.003004406
	1
	0.389634071
	0.46106051
	0.309851357
	1
	0.761470439
	0.858205229
	1.95495E-10
	4.52152E-05

	2.63287E-11
	1
	4.6008E-08
	1
	0.561469823
	1
	1
	1
	0.823404466
	1
	0.244071624
	1
	1
	0.844771481
	1
	0.091690969
	3.75097E-06
	0.186707637
	0.063682443
	0.231818649
	0.949656563
	0.121833357
	1
	1
	5.22757E-06
	0.000309401
	1
	0.13605834
	0.001283663
	1
	0.351340677
	0.357977379
	1
	1.4716E-07
	1

	0.335455604
	1
	1
	1
	1
	0.00191166
	0.009606046
	0.240144807
	1
	1
	1
	8.59997E-07
	0.532201372
	0.014220601
	0.488943224
	0.61101232
	1.87406E-07
	0.381479737
	1
	6.19075E-07
	0.342520488
	1
	1
	0.76606021
	1
	0.388257799
	1
	1
	0.011600093
	1
	0.757631134
	1
	1
	1
	9.22281E-05

	0.053810233
	0.001815425
	0.022200049
	0.04759729
	0.089280906
	0.001398188
	0.008298021
	9.51902E-06
	0.004706143
	0.065634276
	1
	0.109170795
	0.019195574
	0.003571821
	1
	0.00033813
	0.248628519
	0.82163113
	0.13149257
	0.100888939
	1
	0.27804603
	0.237513977
	1
	0.323272454
	0.224312133
	0.268047508
	0.43549565
	0.457147818
	0.98365406
	0.005619613
	0.000407586
	0.031892232
	5.42153E-06
	0.028901212

0.000280654
0.086721951
0.514553901
1
0.274469335
0.034342253
0.041034195
0.01641704
1
0.261572226
0.274136017
0.009413765
0.011038161
0.662945108
1
0.918281325
0.344123794
1
1
0.97251664
0.037427094
1
2.45358E-11
0.155654021
0.899768607
0.441506143
1.21167E-07
1
1
1
1
1
1
0.122603397
0.780578882

1
0.005473726
1
1
0.918946627
1
0.619767749
1
0.766848104
0.562595477
0.958000469
1
1
0.934915391
1

Adj. P-value: (CM_pos_12022024_Newanalysis) / (Water_pos_12022024_Newanalysis)
1
0.702632056
1
0.38345684
1
0.929807213
0.000415422
0.928806032
0.449687183
1
1
2.17109E-07
1
0.279290104
1
1
1
7.0709E-11
1
5.25146E-06
1
6.84375E-06
0.001177473
4.48568E-08
8.09862E-11
1
1
0.876938568
0.624166793
0.17201911
1
0.005211109
0.218768272

1
4.48813E-09
1
1
0.74907763
0.436845839
0.002821911
0.72738756
0.000786681
0.368789957
1
0.008982566
0.000164583
0.037683708
0.018059441
1
1
0.553686846
0.18292542
0.000469395
1
0.361682446
0.868700846
0.570379026
0.994055518
1
0.190200475
0.821836259
1
0.003602909
0.000104491
0.399516023
0.0130762
0.281467684
1

	0.220499329
	0.290469762
	1
	0.002822643
	1
	1
	0.006878668
	1
	8.4402E-09
	0.056189796
	0.005195583
	0.113627807
	0.655415939
	1
	0.041653341
	1
	1
	1
	0.054927538
	0.124954373
	1
	0.404250801
	0.027117053
	0.020720606
	0.956023743
	0.220359891
	0.110127436
	1
	0.042466533
	1
	0.013876197
	1
	0.436012938
	0.115482677
	1

	3.75481E-05
	0.395039743
	0.006507443
	1
	0.260152153
	1
	0.469866738
	1
	1
	1
	1
	0.00374048
	1
	1
	7.0709E-11
	0.107876786
	0.092796173
	0.034911622
	0.043395215
	0.654425943
	1
	0.044241293
	1
	1
	0.635909226
	0.00468839
	0.234985473
	0.090754387
	0.298093971
	2.81036E-08
	0.069614731
	1.69203E-06
	1
	1
	1

	0.649368633
	0.15614564
	1
	1
	1
	1
	1
	1
	1
	1
	0.80652535
	1
	0.123235828
	0.533174978
	0.888759018
	0.000994858
	1
	1
	0.003590687
	0.511053138
	0.714689485
	1
	1
	0.016640493
	1
	1
	0.011674215
	1
	0.954712758
	0.560522265
	0.022439098
	1
	0.011997487
	1
	0.080238386

	1
	0.771567606
	0.591764267
	3.18104E-07
	0.02173429
	1
	0.437362045
	0.600592126
	1.07525E-08
	1
	1
	0.001107249
	6.32804E-05
	3.19478E-05
	0.861484003
	0.000235106
	0.153260642
	1
	4.20476E-09
	1
	0.535455395
	0.859922744
	0.05639984
	0.05438366
	0.089749223
	1
	1
	0.302820649
	1
	0.071368444
	1
	1
	0.00407442
	0.01250616
	0.055810473

	0.791535394
	0.251037871
	0.952698368
	1
	0.203322506
	0.35231902
	1
	1
	0.377156607
	0.841597627
	1
	1
	1
	0.977362072
	1
	1
	0.339328385
	0.009455372
	0.000960246
	1
	0.19919209
	1
	1
	1
	0.889690776
	1
	0.134255639
	1
	1
	1
	1
	0.870830302
	0.903117675
	0.818212966
	0.119664711

	0.844629329
	0.08684632
	1
	1
	1
	1
	0.038140875
	1
	1
	0.000249355
	0.000967004
	1
	1
	2.87941E-08
	0.749999485
	1
	1
	1
	1
	1.38336E-09
	1
	1
	1
	1
	0.78933956
	0.690430855
	0.555607114
	1
	1
	1
	0.279287887
	0.015467859
	0.202967247
	1
	1.27723E-10

	0.000133627
	0.002210771
	1
	1
	1
	0.680830672
	0.009748088
	0.825977759
	0.191052848
	0.300017378
	1
	0.060269436
	0.129145175
	1.40604E-08
	0.977540823
	1
	1
	0.010540535
	0.267078164
	1
	9.85327E-07
	1
	0.033034622
	1
	0.261610882
	0.107425983
	1
	0.014810716
	1.44797E-07
	8.69238E-10
	0.451685121
	0.004550552
	1
	1.18182E-05
	1

0.000142129
0.180267451
0.004165069
0.074438713
4.07872E-05
0.801432855
5.16395E-05
0.028941717
0.019731647
0.470024578
0.108023643
0.000135864
0.014345677
0.616717297
1
0.114431076
0.078669872
0.000246465
2.4583E-05
0.835798037
0.000409416
0.026016531
0.704967974
0.012339527
1
1
1
0.195679878
8.03411E-07
0.910339428
1
1
1
1
1

	0.064196163
	1
	1
	1
	0.535804465
	0.630340284
	0.020697213
	1
	0.227294101
	0.001033394
	0.005381706
	1
	0.344407552
	0.000915788
	0.050102298
	1
	1
	0.224887446
	0.030103754
	1
	2.76333E-07
	1
	1
	0.847881113
	9.56694E-06
	1
	0.046677384
	0.988919513
	0.782248328
	1
	1
	0.207866098
	0.382905639
	0.619547723
	0.824875824

	1
	0.007997854
	0.000291651
	1
	0.074415142
	0.105196211
	0.138981809
	0.737058917
	1.99047E-08
	0.112297932
	0.271743328
	0.568324338
	0.088298249
	0.01034072
	0.083012445
	0.397132005
	1
	7.72452E-06
	0.000654035
	1
	2.08538E-10
	1
	0.755600501
	0.044099021
	0.000109752
	1
	0.001036791
	0.000331478
	1
	1
	0.00577564
	0.045054816
	0.000297259
	0.000209965
	0.95504256

	0.000292891
	1
	0.089959605
	0.029629177
	0.373870319
	0.770723397
	1
	0.047424552
	1
	3.02279E-07
	0.445891267
	0.002690537
	0.000195041
	0.006086845
	1.88233E-06
	0.000391459
	1.74116E-06
	0.646530828
	0.006579544
	2.5922E-09
	0.162607709
	3.94064E-05
	0.037252691
	0.000309188
	0.546361908
	0.000238418
	1
	0.000158785
	1
	0.056082613
	1
	0.011821264
	0.000855334
	0.000109747
	2.49619E-07

	0.00011936
	0.260816333
	1
	1
	0.069184653
	0.000264582
	0.000734037
	0.055098079
	0.977480218
	0.01874206
	0.07089574
	0.004857461
	4.64171E-06
	1
	0.066464624
	1
	0.309433697
	0.148647817
	1
	0.002836511
	1
	1
	0.943649641
	1.28024E-08
	4.85142E-06
	1
	1
	5.12945E-06
	1
	0.02043602
	1
	0.045835712
	0.012333695
	1
	1

	0.622278846
	0.052896389
	1
	0.92343069
	1
	1
	0.029737265
	1
	0.745026126
	0.262696176
	1
	0.85483296
	1
	1
	1
	0.000959411
	1
	1
	0.685455559
	1
	1
	1
	1
	0.912515641
	1
	0.350225092
	0.347001629
	0.357924392
	1
	1
	0.46845871
	0.074459589
	1
	1
	0.945273808

1
0.944529449
1
0.432880676
0.730810521
1
0.186804065
0.07246294
1.80762E-07
0.006465037
1
1
0.624722752
0.00527343
1
1
0.822506052
0.009682094
1
0.590066799
0.00340665
1.35018E-10
8.17337E-05
1
1
0.006203484
2.13453E-05
0.982914547
0.892353288
0.031510659
1
1
1
1
0.000610693

	0.006013011
	1
	1
	0.350225092
	1
	1
	1
	0.517988781
	1
	1
	0.351055802
	1
	1
	0.905701282
	1
	1
	9.86791E-07
	1
	1
	0.123144846
	1
	1
	0.504988379
	0.9219797
	1
	1
	0.994322501
	1
	1
	1
	0.028445437
	0.098742604
	1
	4.54094E-07
	0.972897254

	1
	1
	1
	1
	1
	0.154367786
	2.42565E-07
	0.958422685
	0.001217139
	0.167187739
	1
	0.655638428
	1
	0.000101461
	8.96733E-08
	1
	1
	0.215340317
	0.001376306
	1
	0.274188628
	1
	1
	1
	1
	0.122902074
	0.154932186
	0.030889875
	0.379773643
	1
	0.015377378
	1
	1
	1
	8.18972E-05

	0.29515674
	0.167707549
	0.01487343
	1
	0.02652572
	1
	1
	1
	0.0668845
	1
	1
	0.040246703
	1
	0.001672618
	0.444881605
	0.000413697
	0.482801073
	1
	1
	0.706996544
	0.272668415
	0.177889597
	0.174977062
	1
	0.282611534
	0.087216963
	0.189281622
	0.223787082
	1
	1
	0.006885771
	0.001172043
	1
	0.001987837
	1

	0.000762218
	0.035551122
	0.354813274
	1
	1
	0.113666448
	0.617939805
	0.160697379
	1
	0.011770961
	2.0957E-09
	0.000699951
	0.037393364
	1
	1
	0.346677943
	0.851989196
	1
	1
	1
	1
	0.328042349
	5.46494E-09
	0.097917068
	0.237671099
	1
	0.011572048
	0.580484068
	1
	1
	1
	1
	0.88506185
	0.040352097
	1

	1
	0.073347327
	0.068565731
	0.58874457
	0.111735298
	0.027503962
	0.901540028
	1
	0.46164454
	1
	1
	0.997219243
	0.587883665
	0.00154993
	0.164906169

Adj. P-value: (Rabbitserum_12022024_Newanalysis) / (Water_pos_12022024_Newanalysis)
1
0.147274541
8.78311E-08
0.008004295
0.025733921
1
0.07138554
0.00985366
0.601836115
1
1
1.90795E-06
0.482552995
0.08349607
0.110008338
1
1
1.25336E-11
5.29765E-05
1.25336E-11
1
1.73767E-10
7.00916E-05
1.25336E-11
1.25336E-11
1
0.966064913
0.220652367
1
0.013271801
0.397204592
0.007167795
0.00765555

	1
	1.26177E-11
	4.39022E-07
	1.43159E-05
	0.855396772
	0.744129037
	1.24178E-05
	0.329841963
	1
	0.043398725
	7.79106E-07
	0.000277922
	1.46093E-08
	1.97359E-05
	1.41349E-05
	0.240317565
	0.128687546
	0.055073286
	0.011850023
	5.94192E-07
	0.037200357
	1
	0.966191854
	5.75971E-07
	0.516826508
	0.31259126
	1.78748E-06
	1
	1
	0.006228576
	2.84245E-07
	0.012472812
	0.028156824
	1
	1

	0.597789901
	1
	0.057824286
	0.002921322
	0.18764521
	0.581041617
	1
	1
	1.25336E-11
	7.11388E-08
	1.86221E-07
	1.78267E-10
	0.006203694
	0.006635718
	0.000320061
	0.098325269
	1.99891E-07
	1
	0.001057696
	0.449270435
	1
	1
	0.001109802
	1.29542E-06
	0.000377263
	0.000268908
	8.91102E-06
	0.284189397
	0.000126977
	0.011693582
	0.025261734
	1
	4.76224E-08
	0.000114569
	0.467931878

	2.54905E-07
	0.000676417
	4.99825E-06
	1
	0.755312233
	0.530009782
	0.022837194
	1
	0.00013597
	1
	1
	0.00027809
	0.534652877
	1
	1.25336E-11
	0.014943842
	0.902624075
	0.057424983
	1.25336E-11
	1
	0.017858202
	0.577410688
	0.733990664
	1
	1
	1
	0.020674254
	0.520275097
	1.0845E-05
	1.25336E-11
	5.02856E-07
	2.30539E-05
	1
	0.667964241
	1

0.811788163
1
1
1
0.848821933
1
1
1
0.622336914
1
0.589952465
0.000260904
1.25336E-11
0.326147597
1.04936E-08
1.25901E-11
1
0.068212864
0.595439508
0.060346192
0.369055399
0.000160244
3.17588E-11
3.65771E-07
0.324505287
0.399209912
3.45133E-06
1
1
0.000661557
5.99952E-08
1
2.2928E-07
0.005477611
1.07839E-07

	0.001785388
	1.04377E-08
	0.001599195
	1.25336E-11
	1.38179E-06
	2.20173E-05
	0.0089954
	0.000316167
	4.18474E-07
	4.9256E-06
	0.000104588
	0.005210014
	1.25336E-11
	1.89892E-08
	1
	6.50358E-11
	0.002989871
	0.013584607
	1.25336E-11
	7.2333E-07
	1.27014E-05
	4.51265E-07
	0.000324957
	0.249887476
	0.839430279
	0.829174084
	0.229104033
	0.206232939
	0.062665428
	2.78009E-06
	0.007701323
	0.047508783
	1.98067E-08
	1.12846E-07
	5.98341E-06

	0.044453335
	1
	1
	1
	0.600741847
	1
	0.025478536
	0.000785563
	1
	0.008362917
	0.029239713
	1
	0.229281646
	1
	1
	1
	0.925396104
	9.9658E-08
	2.77125E-07
	1
	2.22708E-06
	0.048845679
	0.166289238
	0.001272986
	1.15716E-10
	0.000273974
	0.158094536
	1
	1
	1
	0.060308647
	0.600338682
	0.193783972
	1
	1.58055E-09

0.001634977
9.05737E-05
1
1
0.046389466
1.27901E-06
1.54717E-07
1
0.941150379
1.25336E-11
2.62722E-08
0.001438384
0.236551733
1.25336E-11
0.000782081
3.58584E-06
0.007391773
0.388326326
0.854511349
1.25336E-11
0.020633382
0.001109012
0.00549171
0.368962509
0.066765853
0.945459918
0.003728362
1
1.76935E-05
0.005596188
9.67951E-06
3.76189E-07
1
1
1.25336E-11

	1.25336E-11
	2.02156E-09
	0.012951157
	0.070075398
	0.004648432
	0.015459567
	3.41028E-11
	1
	0.666217774
	0.000361615
	0.668215924
	0.238603574
	0.092916197
	1
	0.640654545
	1
	1.04498E-07
	1
	0.014233764
	0.115184599
	5.79725E-08
	1.25392E-07
	0.099434894
	1.7547E-10
	0.055173389
	0.004015345
	0.023406164
	3.62237E-11
	4.58282E-05
	1.25336E-11
	0.000545412
	8.15872E-07
	0.033644519
	8.81668E-08
	0.316712993

1.25336E-11
0.001045342
8.17193E-07
5.42973E-06
0.034212073
0.054515968
2.61982E-09
1.67427E-11
0.000398507
8.95548E-09
0.002315161
0.088495491
0.000217625
0.086878152
1
0.08981796
7.2171E-08
8.03473E-09
1.59813E-11
3.5009E-10
2.54943E-05
3.20434E-07
0.015104662
2.03338E-09
0.000358026
1
0.683979421
2.88525E-07
0.015258504
0.001023708
0.005121923
0.463998497
3.57748E-07
0.413839248
1

1
1
0.692834903
0.365336074
0.131510613
1
0.172390364
6.10659E-07
0.258127714
2.45935E-09
1.11893E-09
9.48244E-08
2.08687E-08
0.000855737
0.24517129
0.732832104
0.220382059
0.034428762
0.000493298
1.45274E-06
1.25336E-11
1.20372E-06
1
7.73709E-07
8.8985E-11
1
0.000360949
0.000253506
0.641919779
0.047323222
1
5.30225E-05
3.72422E-07
0.10587709
1

	0.0088945
	0.159944809
	1.93287E-11
	9.3344E-09
	0.001218687
	0.001910589
	8.56337E-09
	2.73436E-07
	1.25336E-11
	0.00024088
	9.20038E-06
	1.75711E-11
	0.004615108
	3.99124E-06
	3.28661E-06
	0.001331777
	0.01330856
	6.05252E-10
	5.57619E-10
	0.501740425
	9.29506E-10
	0.00276812
	0.32646338
	5.78288E-05
	0.000729565
	1
	5.31589E-07
	9.98907E-06
	1
	0.859905145
	8.94181E-09
	1
	1.26177E-11
	8.35949E-08
	0.022907209

7.30423E-09
0.710184054
3.51945E-06
0.00024416
0.002177966
1
1
1
5.52596E-07
1.25336E-11
9.02301E-05
8.45769E-10
6.02148E-09
0.000687381
1.25336E-11
4.14215E-09
1.84428E-10
0.001008429
5.16424E-09
1.25336E-11
4.44869E-06
1.90771E-10
6.46518E-06
1.20647E-06
0.000845623
1.22433E-08
2.03703E-08
1.36161E-10
0.191509279
0.00468286
6.26198E-06
0.00022092
1.94326E-08
3.01606E-09
1.26713E-11

	1.3511E-09
	6.88473E-06
	1.58847E-08
	0.647122767
	0.499307437
	8.04094E-07
	5.74149E-08
	1.67298E-07
	0.56409683
	6.58953E-10
	4.90774E-06
	6.50334E-06
	1.25336E-11
	0.070025334
	1.95506E-05
	0.182253964
	3.29349E-08
	0.652654785
	1
	3.43688E-11
	0.203510232
	0.032943608
	5.37351E-08
	1.25336E-11
	2.32725E-11
	1.20216E-05
	0.000315658
	1.25336E-11
	1
	1
	6.95063E-07
	0.007655482
	8.92268E-09
	1
	0.973531448

	0.222281295
	0.040025182
	1
	0.05163844
	0.0011753
	1.25336E-11
	1
	1
	0.006095228
	1
	0.12339094
	4.46476E-05
	0.167020606
	0.026800364
	0.006119893
	1.20253E-08
	9.22607E-08
	9.26387E-09
	0.004844241
	0.001634977
	0.001227687
	0.038949968
	0.988511412
	1
	0.007018891
	5.20138E-07
	0.000536033
	6.22555E-09
	1.1455E-05
	0.618750926
	5.92862E-05
	1.25522E-05
	7.96504E-07
	1
	4.08704E-09

	0.04366816
	0.627809853
	3.66684E-08
	0.940503965
	1.83471E-11
	0.33869098
	2.03805E-07
	1
	1.25336E-11
	1.35668E-11
	0.07361766
	0.001931379
	2.3068E-05
	0.272227934
	2.2383E-07
	0.0265164
	3.17253E-05
	1.2382E-10
	9.2454E-07
	0.539027916
	6.42892E-09
	1.25336E-11
	1.91879E-11
	0.00070782
	8.84754E-08
	2.58973E-07
	1.45894E-11
	0.00092679
	0.001519564
	3.40271E-08
	0.092520602
	0.157722597
	1.17868E-05
	1
	0.001537732

	1.08518E-08
	1
	5.07959E-07
	0.045368194
	1
	0.858483249
	1
	0.008661466
	0.000412566
	1
	0.010059628
	1
	5.22232E-05
	1.08413E-07
	0.000915256
	0.587444069
	2.78379E-07
	1
	0.658325063
	0.002994175
	0.002109425
	0.099928394
	1
	4.98264E-05
	0.416117034
	1
	0.002617596
	1
	0.262950014
	1
	0.019706494
	1.20103E-06
	0.453163164
	3.23728E-07
	1

	0.136591531
	1
	0.922065238
	0.523622795
	1
	1.30778E-06
	1.25336E-11
	1.16075E-06
	3.23785E-08
	0.266095212
	0.00706581
	0.405690433
	2.16902E-07
	1
	1.25336E-11
	0.006522166
	0.022205345
	0.198797541
	0.002174749
	3.22287E-05
	0.047579109
	0.000143432
	0.679799534
	0.111973074
	0.039539194
	0.01411616
	0.008982783
	7.08336E-10
	2.84844E-05
	0.336160842
	3.91405E-10
	0.021576813
	0.000228094
	0.232092387
	1.95935E-10

	0.746904799
	0.018415941
	7.1625E-06
	0.073512476
	0.000433799
	0.31337606
	0.062761032
	3.32521E-07
	0.000182557
	0.323949715
	7.15869E-05
	0.000393056
	1
	0.000364653
	0.00196423
	0.004454242
	0.013384462
	0.075950552
	0.068067668
	0.590886435
	0.257501303
	0.027754091
	0.024270659
	1
	0.034112935
	0.004527564
	0.028431556
	0.022836156
	1
	1
	8.02539E-05
	4.30573E-05
	1
	0.025751934
	1

	0.065581056
	0.006617518
	0.528830027
	0.000133693
	0.052095859
	1.38129E-10
	1
	1.45058E-05
	0.406201682
	4.32766E-06
	1.25336E-11
	5.86981E-09
	1.99496E-07
	0.001494945
	0.006654229
	0.002798027
	3.10407E-07
	1
	1
	0.547776632
	1
	1
	9.60787E-07
	0.003316039
	1
	0.003926699
	0.107928024
	0.12115965
	0.003473455
	1
	0.012434762
	0.004683937
	0.470521567
	0.008226526
	0.000985738

0.37147399
0.334336202
1
0.032039232
0.000810822
8.58365E-08
0.00075072
1
1
1
0.534936615
0.62284351
0.764656348
0.00015708
1.28227E-05

Adj. P-value: (SM_pos_12022024_Newanalyis) / (Water_pos_12022024_Newanalysis)				
1				
1				
1				
0.136715876				
0.277466377				
1				
1				
1				
0.020620959				
0.082998055				
0.011067123				
6.74791E-07				
1				
0.00059086				
1				
1				
1				
0.313227317				
0.120032448				
0.364937229				
1				
1				
0.077879083				
1				
0.112950679				
1				
1				
1				
0.071008196				
1				
1				
0.009353986				
1				

0.020083945				
4.70828E-05				
1				
0.031404209				
0.525522324				
0.132618891				
0.253889779				
0.483444214				
1				
0.540116116				
0.000429059				
1				
1				
1				
1				
0.046323996				
1				
0.083441995				
1				
1				
1				
3.98909E-05				
1				
9.7032E-05				
1				
1				
1				
1				
0.112030938				
1				
0.360445075				
0.504033755				
0.687351302				
0.678302427				
0.000562035				

0.066413805				
1				
1				
0.058867627				
0.253439106				
4.91239E-05				
1				
0.005791384				
8.03708E-05				
1				
0.000476633				
1.05732E-07				
0.551307704				
0.002907605				
1				
0.057682594				
0.850115124				
1				
0.057270391				
0.060027827				
0.205808075				
0.917429032				
2.17481E-05				
0.001244542				
1				
1				
1				
1				
0.376502745				
1				
0.000432252				
0.346876086				
1				
1				
2.073E-06				

0.00037256				
1				
1				
1				
1				
1				
1				
0.027970674				
1				
1				
0.367854987				
1				
0.512479919				
1				
0.069510239				
0.119106885				
0.003949971				
1				
1				
0.076843079				
0.002061078				
0.859784374				
1				
1				
7.43513E-09				
1				
0.118476851				
0.03322891				
0.546747675				
0.221138683				
1				
0.000622652				
0.252573436				
1				
1				

0.2143984				
1				
0.169566355				
1				
0.804514262				
1				
1				
1				
1				
1				
1				
1				
0.001513512				
0.346730791				
1				
1.06585E-05				
0.205974597				
0.007770059				
1				
1				
1				
0.974102909				
0.583809426				
0.038406358				
1.40427E-06				
0.000826404				
1				
1				
1				
1				
0.001786111				
1				
1				
0.017620784				
0.017855309				

0.004434301				
1				
1				
6.56601E-09				
0.092791456				
1				
0.337441649				
0.542732002				
8.09635E-10				
0.000623624				
1				
0.000499859				
2.06596E-05				
2.74001E-05				
1				
9.35898E-08				
1				
0.000268145				
1.49988E-10				
1				
0.050900488				
0.061100162				
1				
0.717764001				
1				
1				
1				
0.005166119				
1				
0.129238417				
0.036557046				
0.02051542				
0.000330947				
1				
3.09559E-07				

1				
1				
0.87148187				
1				
1				
1.12906E-05				
5.05042E-08				
0.009351515				
0.001140384				
0.000199392				
0.505457454				
1				
0.021519002				
0.276703837				
1				
1				
0.047115096				
4.50669E-05				
1				
1				
0.004236554				
1				
0.016015003				
0.000214659				
0.085367304				
1				
1				
0.133158134				
0.469524168				
0.000747144				
0.89086839				
1				
1				
0.415866077				
1				

0.025702875				
0.026096815				
1				
1				
1				
0.000265824				
0.004711109				
1				
0.286797891				
1.97095E-06				
2.37014E-05				
0.037745434				
1				
2.16034E-07				
0.0488163				
0.002811941				
0.045133662				
0.837767828				
1				
1				
1				
1.36582E-05				
1				
7.81508E-05				
0.917787867				
1				
0.428873221				
1				
0.000583155				
1				
0.003142733				
1				
1				
1				
7.74934E-11				

2.45628E-07				
1				
1				
0.024440345				
1.22843E-05				
0.308232971				
0.0014439				
1				
1				
1				
0.002056992				
0.044899648				
0.061564294				
0.3286484				
1				
0.83266214				
0.758937582				
0.003500484				
0.000707815				
0.944738291				
1				
0.047706715				
1				
0.195388416				
0.45367296				
0.04114822				
1				
0.336389805				
0.895624008				
6.79605E-10				
1				
0.059499542				
0.005988876				
2.12803E-10				
0.817444138				

3.04831E-07				
1				
0.949008579				
1				
2.3646E-05				
1				
5.24103E-07				
0.877019519				
8.66908E-10				
0.024255592				
0.001885448				
6.71093E-06				
4.47691E-06				
0.021276377				
0.000419475				
0.18185881				
0.006743919				
0.779902593				
0.216489915				
0.00022527				
4.80674E-10				
0.000108427				
0.035619121				
0.283670473				
1				
1.82065E-05				
0.255660705				
0.192588723				
1.05373E-05				
0.902486811				
0.583809426				
1				
0.053298537				
1				
1				

0.00187567				
1				
0.285413647				
0.454830835				
0.701753596				
1				
0.002058886				
1				
0.637836253				
0.000242999				
0.030110932				
1				
0.024346656				
1				
1				
0.000173268				
1				
0.568669849				
0.07902343				
0.108836678				
1.86352E-07				
0.02592869				
0.72225015				
1				
0.000124544				
1				
0.042979341				
1				
0.071672185				
0.325323909				
1				
0.671151634				
1				
0.549277341				
8.10124E-08				

0.032302954				
0.696933209				
0.601645269				
0.753361011				
1				
0.101330168				
0.921921454				
0.313835754				
0.14010475				
1				
0.109006218				
0.219798526				
0.016088571				
1				
0.000132185				
1				
1				
2.19188E-05				
0.005028604				
0.018796318				
9.38605E-11				
0.001171383				
0.059119693				
0.551325569				
0.000979841				
1				
0.396144458				
3.19828E-08				
0.767059641				
0.532687113				
1				
0.862162087				
1.24949E-08				
2.94614E-06				
0.039936508				

0.000280859				
1.47102E-05				
6.34843E-06				
0.000121202				
2.75525E-05				
1				
2.08069E-08				
0.000199335				
0.612659799				
1.68443E-06				
0.962751606				
0.000861997				
0.005049866				
5.50669E-08				
7.43513E-09				
1.58915E-09				
0.434035364				
1				
2.154E-07				
7.74934E-11				
0.318812774				
4.73395E-06				
0.005991753				
1.85121E-07				
1.10196E-05				
7.74934E-11				
0.001492502				
1				
0.630463859				
1				
0.005958788				
0.00433404				
0.000270413				
0.0021856				
2.89667E-08				

1				
1				
0.086789756				
0.031621071				
1				
1.07504E-05				
1.15134E-06				
7.44448E-07				
0.458609411				
3.23023E-05				
0.000583155				
4.333E-09				
0.000293105				
0.047436398				
0.08229926				
0.273989745				
0.013887167				
0.699774527				
1				
0.000153729				
1				
0.354935322				
1				
1.19532E-08				
1.69189E-06				
1				
0.000154455				
1.41341E-05				
0.013009206				
0.055223113				
1				
0.002438814				
0.002775087				
1				
0.735657228				

1				
1.04102E-05				
1				
1				
0.752710838				
0.000125262				
0.033745068				
1				
0.366638011				
0.708633438				
0.683174922				
0.003038501				
1				
0.016516783				
0.328624664				
0.009735256				
0.003296611				
9.51763E-08				
1				
2.09454E-06				
0.198682658				
7.54778E-05				
5.28372E-05				
1				
0.173363277				
1				
0.008892116				
0.888253828				
1				
1				
0.020755061				
0.561893436				
1				
0.15916194				
0.334804625				

0.006648466				
0.350630145				
1.20709E-06				
0.911445086				
7.74934E-11				
1				
1				
1				
0.903114126				
1.0839E-05				
1				
0.168335462				
0.000506857				
1				
1				
0.000266259				
0.05343047				
7.74934E-11				
0.111599371				
0.275995225				
3.02146E-06				
0.060201367				
7.74934E-11				
1				
1.2641E-10				
0.120536473				
0.53634911				
0.000437041				
7.74934E-11				
2.41735E-09				
1				
0.92774682				
1				
0.001697075				
1				

0.00029873				
0.711243745				
1				
0.032655682				
1				
0.653492652				
1				
0.069678872				
1				
0.04309174				
0.003410046				
1				
3.28144E-07				
1				
0.317652462				
1				
0.947750695				
0.40690147				
0.020849158				
0.040321243				
4.83767E-09				
1				
1				
0.690052546				
1				
1				
0.01827886				
1				
1				
0.506388306				
0.779067891				
0.001379383				
0.715556593				
3.4922E-07				
1				

0.442361054				
0.901153796				
1				
0.803810006				
0.147189877				
1				
0.911954447				
1				
0.000289214				
7.44198E-07				
0.709379668				
1				
0.997585222				
1				
9.26053E-07				
1				
1				
0.090968916				
0.775833261				
0.365396996				
0.145757127				
0.540861264				
1				
1				
0.884575724				
0.03218061				
0.753531926				
1				
0.004410561				
0.945429148				
0.774379914				
0.489617974				
1				
0.033315974				
0.043776525				

0.601211617				
5.48973E-05				
0.144267437				
0.496754195				
0.000265449				
5.58609E-06				
0.00160582				
7.74934E-11				
3.11226E-05				
1				
7.57565E-09				
0.000687982				
1				
7.01335E-09				
1				
1				
0.000520553				
0.874757626				
0.004463072				
1				
3.05776E-05				
0.074364473				
0.047259777				
1				
0.227430033				
0.038700915				
0.144581162				
1				
1				
0.38145336				
0.643293508				
0.054999654				
1				
5.75136E-10				
1				

1				
0.047757831				
0.000128163				
0.028723442				
0.123899874				
0.982217783				
1				
0.001787713				
0.973701313				
1				
5.43209E-07				
8.0392E-05				
0.030984367				
0.440154791				
0.452202565				
0.022762355				
1				
0.923393593				
0.202167398				
1				
0.948692853				
1				
0.353523347				
1				
0.012717039				
1				
0.000356454				
0.131872689				
0.002946355				
1				
0.072757528				
0.5559978				
0.425960633				
1				
0.662529788				

1				
0.072488469				
1				
0.002360261				
0.560162218				
0.222646029				
1				
1				
0.008266939				
1				
1				
1				
0.758344768				
0.027205324				
2.59551E-07				

